# International Conference on Emerging Infectious Diseases 2012 Poster and Oral Presentation Abstracts

**DOI:** 10.3201/iceid2012

**Published:** 2012-03

**Keywords:** ICEID 2012, Conference, abstracts

Emerging Infectious Diseases is providing access to these abstracts on behalf of the ICEID 2012 program committee (www.iceid.org), which performed peer review. Emerging Infectious Diseases has not edited or proofread these materials and is not responsible for inaccuracies or omissions. All information is subject to change. Comments and corrections should be brought to the attention of the authors.

Download PDF (316 pages, 2.16 MB)

## Supplementary Material

Technical Appendix ICEID 2012 Conference abstracts.

