# Supplementary material for: International Conference on Emerging Infectious Diseases 2012 Poster and Oral Presentation Abstracts
Source: Emerg Infect Dis. 2012 Mar;18(3):e1. doi: 10.3201/iceid2012 (PMC3306230; doi:10.3201/iceid2012)
Supplement: Technical Appendix — ICEID 2012 Conference abstracts. [file ICEID2012.pdf]

# International Conference on Emerging Infectious Diseases 2012 Poster and Oral Presentation Abstracts

Emerging Infectious Diseases is providing access to these abstracts on behalf of the ICEID 2012 program committee (www.iceid.org), which performed peer review. Emerging Infectious Diseases has not edited or proofread these materials and is not responsible for inaccuracies or omissions. All information is subject to change. Comments and corrections should be brought to the attention of the authors.

*Suggested citation for this article:* Authors. Title [abstract]. International Conference on Emerging Infectious Diseases 2012: poster and oral presentation abstracts. Emerg Infect Dis [serial on the Internet]. 2012 Mar [*date cited*]. http://www.cdc.gov/EID/pdfs/ICEID2012.pdf

## Influenza Preparedness: Lessons Learned

Monday, March 12
12:30 PM – 1:30 PM
Grand Hall

**Board 1. Virological Surveillance of Influenza Virus in Mainland of China**

**D. Wang**, Weijuang Huang, Yanhui Cheng, Minju Tan, Xiyan Li,Junfeng Guo, Xiang Zhao, Baohui Shen, Yao Chen, Zhao Wang, Ning Xiao, Hongtao Sui, Bin Liu, Hejiang Wei, Yu Lan, Lei Yang, Ming Li, Yuelong Shu;
National Institute for Viral Disease Control and Prevention, China CDC, Beijing, China.

**Background:** It is necessary to continuously monitor the antigenic and genetic properties of influenza viruses in order to detect any changes and select further vaccine candidates. To determine the effectiveness of antiviral is also one of the essential role for virological surveillance. **Methods:** Clinical throat swab samples from ILI cases were obtained from sentinel hospitals in mainland China. Viruses

were isolated with MDCK cells or embryonated chicken eggs. The viruses were analyzed antigenically by haemagglutination inhibition (HI) testing with ferret antisera. Viral RNA was extracted and complementary DNA was synthesized by reverse transcription reaction and then the genes were sequenced, then phylogenetic and anti-viral resistance analysis were conducted based on the sequence. **Results:** Since April, 2011, the percentage of visits with ILI (ILI%) of national sentinel hospitals in China remained at low level. In recent 20 weeks, the percentage of tests that were positive for influenza virus was lower than 5%, with influenza B virus as the predominant strain. Since April, 2011, 99% A(H1N1)pdm09viruses were antigenically similar to the vaccine virus A/California/07/2009 (H1N1), and share an AA change of S203T in the HA gene. For A(H3N2) viruses, 91.7% (33/36) were antigenically similar with the vaccine virus A/Perth/16/2009, and the HA gene formed two genetic groups which were antigenically undistinguishable. 93.2% of the B Victoria lineage viruses were antigenically similar to vaccine virus B/Brisbane/60/2009. Since April, 2011, all A(H1N1)pdm09 and A(H3N2) viruses tested were resistant to adamantine; only 1 A(H1N1)pdm09 virus was resistant to the neuraminidase inhibitors, others are still sensitive to the neuraminidase inhibitors; all influenza A(H3N2) and B viruses are sensitive to neuraminidase inhibitors. **Conclusions:** Enhanced surveillance in Mainland China during the fight with pandemic A (H1N1) will continue to play a key role in monitoring influenza virus. The national wide influenza surveillance system is also the bases for capacity building of other respiratory disease surveillance and is essential for timely information sharing of public concern.

**Board 2. Divergent Patterns of Seasonality of Circulating Influenza Viruses in Two Regions of Northern India**

**S. Broor**[1], P. Koul[2], M.A. Mir[1], M.S. Chadha[3], A.C. Mishra[3], R.B. Lal[4];
[1]All India Institute of Medical Sciences, New Delhi, India, [2]2SheriKashmir Institute of Medical Sciences, Srinagar,, Srinagar, India, [3]National Institute of Virology, Pune, India, [4]Centers for Disease Control and Prevention, Atlanta, GA, USA.

**Background:** Distinct patterns of pandemic 2009A/H1N1 (pH1N1) influenza were observed in the two northern regions of India (500 miles apart), with Srinagar having peaks of influenza activity in the winter season (Jan-Feb, 2010) and Delhi region in monsoon times (July-Aug, 2010). We performed systemic surveillance to determine trends of circulating influenza viruses in these two regions of north India in 2011. **Methods:** A total of 1,086 patients presenting with influenza like illness (ILI) at the tertiary care centers of SKIMS, Srinagar (n=556) or AIIMS, New Delhi (n=530) were enrolled from January 1-September 15, 2011. Throat and nasal swabs were collected from all patients and tested by real time reverse transcribed Polymerase Chain reaction (rRT,PCR) for seasonal and pandemic influenza viruses. **Results:** One hundred and twenty one (22%) of 556 specimens from Srinagar were influenza positive; of these 93 (77%) were pH1N1, 1 (<0.1%) was A/H3N2 and 27 (22%) were Influenza B. In contrast, only 50 (9.4%) of 530 specimens from New Delhi were influenza positive; of which, 38 (76%) were A/H3N2 and 12 (24%) were Influenza B, suggesting a remarkable difference in the prevalence of influenza and distribution of circulating influenza A viruses at these two centers. In addition the seasonality of influenza has distinct pattern. In Srinagar, during Jan-Feb, 2011, there was high positivity for pH1N1 (33% of specimens positive) with co-circulation of Influenza B (monthly positivity 2-8%) with no influenza positivity since May, 2011. In contrast, in Delhi there was high positivity for A/H3N2) in July (34% of all ILI cases tested for the month) and Aug (15%), 2011 with sporadic influenza B positivity during Aug-Sept, 2011 and no influenza circulation was seen from Jan-June, 2011. **Conclusions:** Our data shows that two regions in Northern India have distinct influenza seasonality and circulating viruses. Srinagar had peak activity in Jan-Feb and a predominance of pH1N1, whereas Delhi had peak activity in Jul-Aug with emergence of A/H3N2 in 2011. Continued global surveillance will define regional differences in influenza seasonality and types and subtypes, as well as optimal periods for implementation of influenza vaccination programs among priority populations. *Study supported in part*

*by cooperative agreement 5U51IP 000333 from the Centers for Disease Control and Prevention and Indian Council of Medical Research, New Delhi, India.*

**Board 3. Introducing a Novel Framework to Assess the Impact of an Emerging Influenza Pandemic in the United States**

**C. Reed**, M. Biggerstaff, L. Finelli, L.M. Koonin, D. Beauvais, A. Uzicanin, A.D. Plummer, J.S. Bresee, S.C. Redd, D.B. Jernigan;
Centers for Disease Control and Prevention, Atlanta, GA, USA.

**Background:** The overall impact of influenza in the population may be primarily attributable to a combination of both the clinical severity of illness in infected individuals and the extent of transmission of the infection. Because of variability in both severity and transmission among influenza viruses, the resulting impact of influenza on public health, healthcare, and society can vary greatly between seasons and between pandemics. **Methods:** To create a standardized and systematic approach to assessing the impact of an emerging pandemic, we examined published data from past influenza seasons and pandemics to evaluate epidemiologic measures of transmission and severity, characterized the range of values seen from influenza historically in the US, and outlined a conceptual framework to synthesize measures of transmission and severity of an emerging pandemic. **Results:** We divided the framework into two periods. In the initial assessment, during the early stages of a pandemic, few epidemiologic data may be available and early indicators of transmission and severity can be variable; therefore, they were categorized using a broad dichotomous scale that divided the range of historic values. In the refined assessment, as increased data become available later in a pandemic, the ranges of transmission and severity measures were more finely categorized using an ordinal scale. We then used historic data to place past pandemics and selected seasons in the framework to illustrate the range of impact seen with influenza. Because transmission and severity may differ by age or other risk groups, we show how age-stratified data may be incorporated into the framework. **Conclusions:** This tool provides an evidence-based foundation upon which strategic decisions and operational plans can be developed to guide and communicate the pandemic influenza response. Such an approach may help inform the assessment of pandemic impact by organizing available epidemiologic information in a way that can facilitate decision making and prioritize data collection by providing public health officials a set of key parameters to work toward characterizing. While we used data from the United States, the framework provides a basic structure to synthesize epidemiologic data that may be useful in other settings as well.

**Board 4. Sentinel Surveillance of Severe Acute Respiratory Infections in the 2008-2011 Epidemic Seasons in the Republic of Kazakhstan**

**G.U. Makhmetova**;
Astana Department of the Committee for State Sanitary and Epidemiological Surveillance, Astana, Kazakhstan.

**Background:** Determining the burden of influenza helps a country plan health care needs, determine immunization need, detect pandemics and contribute to global influenza knowledge. In 2008, sentinel surveillance for severe acute respiratory infections (SARI) was established in 19 sentinel hospitals in 7 large cities, mostly on the border with neighboring countries such as Russia and China, of the Republic of Kazakhstan. Our principal goal was to establish a system that monitors types of influenza virus with a secondary goal of establishing a system that can accurately determine morbidity. We analyzed data for the last three seasons, 2008-2011. **Methods:** We taught clinicians to recognize SARI patients by WHO standard case definitions and to take specimens for testing, and laboratory specialists to do PCR typing and subtyping for influenza virus. Individual, clinical, epidemiological and laboratory data were collected. Samples for virus testing were collected from patients >1 year-of-age who met the WHO standard SARI case definition. Data were analyzed in Epi-info 2000. **Results:** We tested 454 and 500

patients in the 2008-2009 and 2009-2010 seasons, respectively, and increased this number to 1279 in 2010-2011 by extending sample collection from 3 to 7 days from the onset of disease. Weekly distribution of the SARI patients reflects the flu season in the country. The first and the last season started in the second week of January and lasted until week 17, while the pandemic season of 2009-2010 started unusually early, in the 44$^{th}$ week. The predominant virus in 2008-2009 was seasonal A/H1N1 (78.5%), with type A causing 77% of infections in the pandemic year (subtyping was not performed), with the percentage of influenza A virus in 2010-2011 decreasing to 68% (230 cases), when a variety of type A viruses appeared; seasonal influenza H1N1- 30% ( 68 cases), pandemic influenza H1N1- 15% (34 cases), H3N2 - 33% (75 cases), and untypeable influenza A - 22% (53 cases). The 2010-2011 season was unusual, with influenza B virus occurring the entire season (32% of all PCR positive results). **Conclusions:** The goal of implementing of laboratory SARI surveillance was achieved in Kazakhstan. We will next establish comprehensive SARI surveillance for the next flu season to enable us to determine national SARI morbidity.

## Policy Implications and Infectious Diseases

Monday, March 12
12:30 PM – 1:30 PM
Grand Hall

### Board 5. Changing Trends of Invasive Group B Streptococcal Infections among Non-Pregnant Adults, Minnesota (MN), 2000-2010

**C. Holtzman**, R. Lynfield, R. Danila, C. Lexau;
Minnesota Department of Health, St. Paul, MN, USA.

**Background:** Although invasive GBS disease (iGBS) incidence is highest in infants, the burden of disease is greatest in adults $\geq$ 50 years of age. Diabetes mellitus (DM) is a risk factor for iGBS. The prevalence of diagnosed DM among U.S. adults has risen sharply from 5% in 1997 to 9% in 2009. We examine epidemiologic and clinical characteristics of iGBS infections occurring in non-pregnant adults over the past 11 years. **Methods:** As part of the CDC Active Bacterial Core Surveillance program (ABCs), the MN Department of Health conducts active, population-based surveillance for iGBS. Cases included non-pregnant adults ≥ 18 years of age, with GBS isolated from a normally sterile body site. Recurrent cases were those occurring ≥ 30 days after initial iGBS culture. The Cochran-Armitage test for trend was used to assess changes in proportions by 1-year intervals. **Results:** 3,305 cases of iGBS in non-pregnant adults were reported during 2000-2010 (median age: 64 years). The age-adjusted rate of iGBS increased 53% from 6.51 to 9.95 cases/100,000 from 2000-2010. Overall, 43% of iGBS cases had DM, and there was an increase in DM cases from 35% in 2000 to 49% in 2010 (trend: $p < .001$). Bacteremia without focus (41%), invasive cellulitis (19%), and osteomyelitis/arthritis (17%) were the most common iGBS syndromes. Between 2000 and 2010 the proportion of cases with bacteremia without focus decreased 33% (trend: $p=0.003$), osteomyelitis/arthritis increased 85% (trend: $p<0.001$), with no significant change in proportion of invasive cellulitis cases. A total of 178 cases had iGBS recurrence, 38% < 6 months, 52% <1 year, 72% <2 years, and 96% <5 years of initial infection. There was no change over time in proportion of iGBS cases who were hospitalized, 92% overall, however, the proportion of iGBS cases who died, 6.2% overall, decreased by 20% (trend: $p=.041$). **Conclusions:** Over these years the incidence of iGBS in non-pregnant adults has significantly increased in MN. The associated increases in the prevalence of DM among these cases suggest that DM has played a role in this increase. However, this analysis cannot rule out other causes for the increase, such as changes in diagnostic practices. Due to

the increasing prevalence of DM and the growing burden of GBS disease we have reported, further analytic studies are warranted.

**Board 6. Pandemic Influenza H1N1 Vaccination Intention, Associated Factors, and Implications from Two National Surveys in Taiwan: A Comparison between Phase-6 Pandemic and Post-Pandemic Phase**

**J.-H. Huang**, Y.-Y. Miao, P.-C. Kuo;
Institute of Health Policy and Management, College of Public Health, National Taiwan University, Taipei, Taiwan.

**Background:** This study aimed to investigate and compare intention to receive vaccination against pandemic influenza A/H1N1 (pH1N1) and its associated factors in the Taiwanese general population during phase-6 pandemic vs. post-pandemic phase. **Methods:** Two national surveys using random digit dialing were conducted in Taiwan during October 28-30, 2009 and October 27-29, 2010, representing the pandemic and post-pandemic phase, respectively. **Results:** Seventy percent (756/1,079) of the participants intended to receive vaccination during the pandemic phase, whereas only 54% (580/1,067) intended to do so during the post-pandemic phase. Interestingly, multivariate logistic regression indicated that factors associated with vaccination intention were different across these two phases. During the pandemic phase, participants who perceived pH1N1 in Taiwan to be much more severe than that in other countries (adjusted odds ratio [AOR]=1.94), who agreed (AOR=2.44) or strongly agreed (AOR=2.53) that contracting pH1N1 would have a great impact on their daily life, who perceived pH1N1 vaccination to be very effective in preventing pH1N1 (AOR=2.64), and who considered receiving vaccination not very difficult (AOR=8.97) or not at all difficult (AOR=30.72) were more inclined toward getting vaccinated against pH1N1. By contrast, during the post-pandemic phase, participants who perceived vaccination to be effective (AOR=1.95) or very effective (AOR=5.34), and who considered receiving vaccination to be not very difficult (AOR=11.47) or not at all difficult (AOR=34.92) were more inclined to be vaccinated. **Conclusions:** Perceived risk of pH1N1 was significantly associated with vaccination intention during the pandemic phase; however, the effect of perceived risk disappeared in the post-pandemic phase. On the other hand, perceived effectiveness and perceived difficulty regarding pH1N1 vaccination were significantly associated with vaccination intention during both pandemic and post-pandemic phases. These findings suggest that to enhance preparedness toward future pandemics, evidence-based information regarding the effectiveness of protective behaviors should be emphasized and communicated to the public, while perceived barriers should be better understood, addressed, and hence minimized.

**Board 7. Community Associated *Clostridium difficile* Infection in Select New Haven, Connecticut, Area Towns: 2010.**

**C. Lyons**, J. Meek, H. Fowler, B. Paccha, J. Hadler, R. Heimer;
Connecticut Emerging Infections Program, Yale School of Public Health, New Haven, CT, USA.

**Background:** The emergence of more toxigenic strains of *C. difficile* has changed the epidemiology of *C. difficile* infection (CDI) in the past decade, particularly in healthcare settings. The recognition of these strains in the community led CT Department of Public Health to make community-onset CDI physician reportable in 2006, which yielded an incidence of community associated (CA) CDI of ~7/100,000. In 2009, the CT Emerging Infections Program, in collaboration with CDC, established an active, laboratory-based surveillance system for CDI to further understand the epidemiology of CA CDI. **Methods:** Surveillance occurred in a 23-town area around New Haven CT (2010 population ≥ 1 year of age: 643,934). Line lists of all *C. difficile* toxin positive laboratory specimens from residents of the 23-town area were collected. An incident CDI case was a resident of the 23-town area ≥ 1 year of age with an initial toxin positive *C. difficile* stool specimen or a subsequent positive specimen > 8 weeks after the last positive specimen. Medical charts were reviewed to gather epidemiological and clinical data. CDI cases

were classified as healthcare facility onset (HCFO), community onset healthcare facility associated (CO-HCFA) or community associated (CA) based on location of disease acquisition and healthcare exposures. **Results:** In 2010, 887 incident CDI cases were identified (138 cases per 100,000 persons); 553 were HCFO, 151 CO-HCFA, and 183 CA (28/100,000). CA cases were significantly younger than both HCFO and CO-HCFA cases (58.3 ± 21.7 years vs 76.1 ± 15.7 and 67.4 ±18.3, p≤0.001). Thirty-four percent (34%, n=62) of CA cases were hospitalized. Of those, 46 (74%) had at least one underlying medical condition. In the 14 days prior to stool collection 34(55%) took antibiotics, 1(2%) took H2 blockers, 17(27%) took proton pump inhibitors and 16(6%) received immunosuppressive therapy. **Conclusions:** CDI is a substantial burden in the community; the observed rate of 28 CA CDI cases per 100,000 population is twice that of other enteric pathogens (Salmonella and Campylobacter) in Connecticut and 4 fold higher than the 2006 CA CDI rate obtained though passive surveillance. More than half of hospitalized CA CDI cases had recent antimicrobial exposure emphasizing the importance of outpatient antimicrobial exposure on the development of CA CDI.

**Board 8. Strengthening the Department of Defense Global, Lab-Based, Influenza Surveillance Program: A Focus on United States Coast Guard**

**L.V. Lloyd**[1,2], E. Schwartz[3], S.A. Bisaha[4,5], K.J. Tastad[1,6], J.D. Escobar[1], J.P. Abshire[1,6], C.A. Schlorman[1,6], V.H. MacIntosh[1];
[1]United States Air Force School of Aerospace Medicine, Wright-Patterson Air Force Base, OH, USA, [2]Oak Ridge Institute for Science and Education, Oak Ridge, TN, USA, [3]United States Coast Guard Headquarters, Washington, DC, USA, [4]United States Air Force School of Aerospace Medicine, WPAFB, OH, USA, [5]Henry M. Jackson Foundation for the Advancement of Military Medicine, Inc.,, Bethesda, MD, USA, [6]Henry M. Jackson Foundation for the Advancement of Military Medicine, Inc., Bethesda, MD, USA.

**Background:** The maritime missions of the U.S. Coast Guard (USCG), to serve in the frontline for federal regulatory operations related to international commerce and law enforcement, place USCG personnel at increased risk for various communicable diseases. USCG units, either in littoral zones or port locations, often require personnel to live and operate in close quarters (e.g., USCG cutters). Crowded living conditions can contribute to infectious disease transmission. However, establishing routine disease surveillance in the smallest military service has been challenging. One way the USCG monitors viral respiratory disease is through participation in the Department of Defense (DoD) Global, Lab-Based, Influenza Surveillance Program. **Methods**: We reviewed USCG specimen submissions to the program for the previous three influenza seasons. This was compared to the number of influenza-like illness (ILI) visits for USCG personnel captured in DoD ambulatory medical care data records using ICD-9 codes. **Results**: During the three previous seasons, 43 USCG clinics treated patients presenting with ILI. There were 19,646 ILI visits during the 2008-09 season, 16,229 ILI visits in 2009-10, and 11,790 visits in 2010-11. The number of specimens submitted to the DoD surveillance program was much lower: 264, 161, and 17, respectively. USCG lab specimens submitted during the three seasons made up 1.5% of all specimens submitted by participating services (Army, Navy, Air Force, Marines). **Conclusions:** In prior seasons, a small number of USCG specimens were submitted because the USCG only had two sites assigned as sentinel sites. However, based on ILI incidence, the USCG should increase its participation in the DoD influenza surveillance program. With unique work environments, large geographic coverage, and frequent interaction with foreign populations, the USCG is in position to capture respiratory data not previously available. For the 2011-2012 influenza season, all USCG clinics were selected as sentinel sites. We expect this will strengthen the DoD influenza surveillance program's ability to quickly identify and respond to new circulating strains, and to contribute data for vaccine development. Increased USCG participation will also enhance the efforts of global disease surveillance.

## Improving Preparedness for Infectious Diseases

Monday, March 12
12:30 PM – 1:30 PM
Grand Hall

**Board 9. A Nigerian Survey on Clinical Laboratory Diagnosis of Anaerobic Infections**

**F.O. Nwaokorie**, N.N. Nwokoye, J.I. Yisau;
Nigerian Institute of Medical Research, Yaba, Lagos, Nigeria.

**Background**: Anaerobic species of the Genus *Bacteriodes, Prevotella, Porphyromonas, Fusobacterium*, *Peptostreptococccus* and *Clostridium* are common pathogens in variety of human infections. Their identification depends to a large extent on the use of appropriate anaerobic techniques in the laboratory. In Nigeria, much emphasis are laid on diagnosis of aerobic than anaerobic species. This survey looked at the level of involvement of clinical Laboratory personnel in anaerobic diagnosis of human infections in Nigeria. **Methods**: Questionnaires were distributed by non-random sampling to Medical Laboratory Scientist in a conference in Lagos, Nigeria and analyzed at the Nigerian Institute of Medical Research using EPI INFO. **Results**: Of the 160 questionnaires distributed, 152 (95%) were returned. Most respondents were from privately owned diagnostic centres (26.9%) and University Teaching Hospitals (25%). We observed that 50.7% practices at least one form of anaerobic technique in their respective laboratories while 49.3% do not. Most frequently used technique was anaerobic culture (54.5%) but lapses was observed in the procedure and materials used to maintain anaerobiosis. Few respondents (2.6%) agreed to have used molecular methods. Among the 50.7% that perform one form of anaerobic technique or another 57.1% do so routinely, 37.7% on demand from physicians and 2.6% quarterly. In addition, 13% perform antibiotics susceptibility testing out of which 10% use agar dilution method, disc diffusion 80% and E-test 10%. Major reason (53.0%) for nonperformance of anaerobic diagnosis was lack of fund to purchase equipment and reagents. All participants (100%) showed their interest and willingness in laboratory diagnosis of anaerobes if given necessary training and materials. **Conclusions**: Lapses in clinical diagnosis of anaerobic infections in Nigeria is a serious issue that could lead to misdiagnosis of many infections, emergence of resistant strains, treatment failure and deaths. There is an urgent need to create awareness on anaerobic infections among the medical professionals, provide materials and train laboratory personnel on anaerobic techniques. This will help in promoting more accurate diagnosis of human infections.

**Board 10. Assessment of Laboratory Capacity on Infectious Diseases Detection among Public Health and Clinical Laboratories in Guangdong Province, China**

**H. Li**[1], W. Su[1], H. Zhan[1], W. Li[1], C. Ke[1], B. Li[1], L. Fang[1], C. Ma[1], R. Cao[1], A. Hao[1], M. Chan[2], D. Mills[2], S. Granade[3], M. Rayfield[3], X. Liu[3];
[1]Center for Diseases Control and Prevention of Guangdong Province, Guangzhou, China, [2]Association of Public Health Laboratories, Silver Spring, MD, USA, [3]Centers for Diseases Control and Prevention, Atlanta, GA, USA.

**Background:** To better understand the diagnostic capabilities of infectious diseases at the public health (PH) and clinical labs (CL) in Guangdong Province (GD), China; to provide evidence of the need to improve lab services to policy makers, and promote the development of an advanced infectious diseases lab system that supports PH surveillance and emergency response. **Methods:** Thirty-one labs including 15 PHLs and 16 CLs in five cities of GD were selected to represent each county, city level PHL and CL. An approved version of WHO Lab Assess Tool (LAT) was customized to China's PH policy and regulations, translated to Chinese by Chinese Center for Disease Control and Prevention (China CDC). GD CDC jointly worked with US-CDC, the Association of Public Health Laboratories (APHL) to revise the LAT according to

local context to include 12 modules ranging from adequacy of facility, specimen management, testing performed to reporting then provided training to local assessors this June. Data were analyzed by SPSS 13.0. **Results:** Thirty-one questionnaires were given out, and 29 responses were returned (93.5%) with on-site assessment. The findings revealed that across sites adequacy of facility scored the highest (>85%) while testing availability & competence scored the lowest (<60%). Significant gaps were identified in the following areas comparing the labs in the Pearl River Delta Economic Zone versus non-delta regions: Reagents supplies (p=0.001); Quality (p=0.007) and Biosafety (p=0.004). We also found statistically significant differences on testing availability & competence (p=0.004) at PHLs (66.4±18.1%) versus CLs (46.0 ±19.6%) based on 50 infectious pathogens surveyed as well as for quality assurance practice (p=0.037): PHLs (82.1±11.1%) versus CLs (70.7±17.1%). **Conclusions:** Facility conditions at GD labs have been improved since 2003 SARS. Most labs have the basic diagnostic capabilities, but the abilities of lab surveillance, identification and response to outbreaks and emerging infectious diseases need to be improved. The testing capacity difference between PHLs and CLs may not be an issue if specimens can be referred regularly to fulfill their different mission/roles. The modified LAT can be used by other provinces leading to a better understanding on the development of an efficient lab network in China.

**Board 11. *Clostridium difficile* Infections among Children in a Beijing Community: Toxin Profiles, Ribotypes and Antibiotic Resistance**

**Y. Cheng**[1], Q. Yan[1], H. Jia[2], L. He[3], J. Lu[1];
[1]Department of Hospital Acquired Infection Control and Prevention, SKLID, ICDC.China CDC, BeiJing, China, [2]Department of Clinic Laboratory; China-Japan Friendship Hospital, BeiJing, China, [3]Shenzhen Center for Disease Control and Prevention, Shenzhen, China. *Granted by the Young Scholar Scientific Research Foundation of China CDC (NO.2011A101) and NSFC (No.81101218).*

**Background:** C. difficile, is a well known cause of adult healthcare-associated (HA) diarrhea. Recently, reports of community-associated (CA) C. difficile infections (CDI) among children are increasing worldwide. The data from Asia and China on CA-CDI, however, are quite limited. To characterize the different molecular characteristics of children with CA-CDI , we conducted a 3-month survey in a Beijing community intestine clinic. **Methods:** We collected diarrheal stool specimens from 95 children (0-14 years old) at an intestine clinic of a hospital. We performed conventional PCR to detect the gene tcdA, tcdB of toxin A and B, cdtA, cdt B of binary-toxin. Commercially-available toxin detection kits were used to screen toxin A/B the same time on the C. difficile isolates. Then, we used PCR ribotyping to examine the association among them. We use PCR to detect erm B, gryA and gryB, and e-test to detect antimicrobial susceptibility (moxifloxacin, levofloxacin, erythromycin, clindamycin, tetracycline, rifampicin and vancomycin) of the isolated C. difficile. **Results:** We isolated 16 C. difficile strains, and the isolate rate is 16.8%(16/95),including 8 A-B+ strains and 8 A+B+ strains.2 out of 8 A-B+ strains(25%, 2/8) have positive kit toxin results, while 5 out of 8 A+B+ strains(62.5%, 5/8) have positive kit toxin results. 16 strains may be divided into 10 different types, and the ribotyping show certain gene polymorphism. 3 A-B+ strains belong to the type I, and 3 A+B+ strains belong to type II . 2 A-B+ strains, and 2 A+B+ strains belong to type III and IV respectively. The other 6 strains belonged to 6 different types. We found no isolates belonged to ribotype 027 or 078. Resistance rate against moxifloxacin, levofloxacin, erythromycin, clindamycin, tetracycline, rifampicin and vancomycin was 0, 0, 62.5%, 62.5%, 0, 0 and 0, respectively. **Conclusions:** Our results reveal that C. difficile may be the possible cause for children bacterial diarrhea; there is gene polymorphism in the isolates; high clindamycin resistance and low quinolone resistance highlight the antibiotic resistance difference between childhood CA-CDI and adult HA-CDI.

**Board 12. Seasonal Patterns of Common Infectious Diseases in Children under 5 years of Age in Rural Western Kenya, 2007-2010.**

**A.O. Audi**[1,2], D.R. Feikin[2,1], G.M. Bigogo[2,1], K. Mutai[1,2], J. Williamson[1], R.F. Breiman[2,1], D.C. Burton[2,1];
[1]Kenya Medical Research Institute/CDC Research and Public Health Collaboration, Kisumu and Nairobi, Kenya, Kisumu, Kenya, [2]International Emerging Infections Program-Kenya, Division of Global Disease Detection and Emergency Response, Centers for Disease Control and Prevention (CDC)-Kenya, Kisumu and Nairobi, Kenya, Kisumu, Kenya.

**Background:** While seasonality of common infectious disease syndromes may have implications for optimizing preventive and treatment services, it has not been fully characterized for young children in sub-Saharan Africa. **Methods:** As part of longitudinal population-based surveillance among approximately 25,000 participants in rural western Kenya, illness symptoms were solicited at regular (weekly or biweekly) home visits using standard questionnaires. We calculated monthly rates of diarrhea, acute febrile illness (AFI) and acute respiratory illness (ARI) from January 1, 2007, to December 31, 2010, among children <5 years. Months were grouped into 4 seasons as per local rainfall and temperature patterns: December-February, hotter dry season; March-May, long rains; June-August, cooler dry season; and September-November, short rains. We used Poisson regression to compare longitudinal prevalence rates (defined as total number of days of a syndrome over total number of eligible observation days, converted to person-years of observation [Pyo]) for different seasons. **Results:** There were 375,803 household visits conducted among 11,363 children < 5 years. Overall longitudinal prevalences of AFI, ARI and diarrhea were 33.9 (95% CI 33.2 - 34.7), 57.6 (95% CI 56.3 - 59.0), and 7.5 (95% CI 7.2 - 7.8) days per Pyo, respectively. Significant seasonal trends in prevalence were observed for each illness syndrome. For diarrhea, the prevalence was highest in the hotter dry season (8.6 days per Pyo, 95% CI 8.2 - 9.1) and lowest in the short rains season (6.2 days per Pyo, 95% CI 5.9 - 6.6) (rate ratio [RR] = 1.39, 95% CI 1.30 - 1.48). For AFI, the prevalence peaked twice during the hotter (36.8 days per Pyo, 95% CI 35.8 - 37.8) and cooler (36.2 days per Pyo, 95% CI 35.2 - 37.2) dry seasons compared to the short rains season (30.4 days per Pyo, 95% CI 29.6 - 31.3) (respective RRs 1.21, 95% CI 1.18 - 1.24, and 1.19, 95% CI 1.16 - 1.22). For ARI, the prevalence was highest during the cooler dry season (70.4 days per Pyo, 95% CI 68.6 - 72.1) and lowest in the long rains season (52.4 days per Pyo, 95% CI 50.9 - 53.9) (RR = 1.34, 95% CI 1.31 - 1.38). **Conclusions:** Among children <5 years in rural western Kenya, the burden of common infectious disease syndromes varies significantly by season. Etiology-specific data will be examined to explain some of these seasonal trends.

**Board 13. Use of Population-Based Survey Data to Assist during the Response to an Influenza Pandemic**

**D. Drociuk**[1], A. Alianell[1], R. Oldendick[2];
[1]South Carolina Department of Health and Environmental Control, Columbia, SC, USA, [2]University of South Carolina, Institute for Public Service and Policy Research, Columbia, SC, USA.

**Background:** This presentation will describe the establishment of a monthly population-based survey tool which collected data on: a) influenza-like illness (ILI) prevalence, b) impact on healthcare facilities and c) H1N1 vaccine acceptability/uptake and the use of these data in our response to pandemic H1N1 influenza. **Methods:** A partnership was created between the South Carolina Department of Health and Environmental Control (SC DHEC) Division of Acute Disease Epidemiology (DADE) and the University of South Carolina's Institute for Public Service and Policy Research (IPSPR). A survey instrument was created and administered by phone monthly. The purpose of this survey was to develop measures of H1N1 influenza severity not related to laboratory or hospital reports as another method to monitor ILI prevalence H1N1 vaccination activities. The survey was performed each month, beginning in November 2009, and extended for six months. Data was provided to SC DHEC each month to allow for rapid analysis and interpretation of results to direct our influenza surveillance vaccination campaigns. **Results:**

Detailed discussion and statistics regarding monthly variations will be described during the accepted presentation. Between Nov 2009 - April 2010: a) ILI prevalence statewide averaged 8.6% (range: 5%-11%), b) an average of 2.5% of individuals reporting ILI symptoms required at least an overnight stay at a hospital (range: 2%-4%), c) an average of 13.5% reported seeking care at an emergency department due to ILI illness (range: 10%-27%), d) laboratory tests for H1N1 averaged 12.7% (8%-27%), e) H1N1 vaccine coverage showed an increasing trend from 8% in November 2009, to 23% in April 2010. Among individuals who did not receive H1N1 vaccine, "Not sure if I need it" and "Not sure if it was safe" remained the top two reasons stated by individuals for not receiving the H1N1 influenza vaccine, throughout the six-month survey period. **Conclusions:** The results provided via this monthly survey tool greatly assisted in communication both internally within Public Health and to our external partners. In addition, having repeated "snapshots" of monthly data aided in identifying the need to craft targeted messages to risk groups and provide situational awareness of the progression of ILI illness within the State.

## New or Rapid Diagnostics

Monday, March 12
12:30 PM – 1:30 PM
Grand Hall

**Board 14. Building a Better Staphylococcal Enterotoxin Mousetrap**

**S.M. Tallent**[1], J.A. DeGrasse[1], N. Wang[2], D.M. Mattis[2], D.M. Kranz[2];
[1]US FDA/CFSAN/ORS, College Park, MD, USA, [2]University of Illinois, Urbana-Champaign, IL, USA.

**Background:** *Staphylococcus aureus*, a common foodborne pathogen, secretes a family of small (25-30 kDa) exotoxins known as staphylococcal enterotoxins (SEs). Since they are heat- and pH-resistant, the SEs persist long after the microbe has been eradicated. SEs are also superantigens that induce non-specific T-cell activation which can rapidly cascade to a massive release of inflammatory mediators and may lead to toxic shock. Staphylococcal food poisoning (SFP) occurs when improperly handled food contaminated with as little as 100 ng of SE is consumed. SFP is marked by severe gastrointestinal symptoms such as emesis, diarrhea, and/or abdominal pain after a 4 hour incubation period. **Methods:** Previous work successfully engineered soluble receptors that contain the Vβ domain of T-cell receptor (Vβ-TCR) with specificity and high-affinity for three staphylococcal superantigens including SEB, SEC3, and TSST. In this work, the sensitivity and specificity of the Vβ-TCR G5-8 was compared to several commercially available monoclonal antibodies (mAbs) for the detection of SEB in cell-free culture supernatants and spiked food matrices. The limit of detection and specificity of the Vβ-TCRs and mAbs were compared using two assays: a multiplex fluorescence bead-based assay and immunoprecipitation followed by liquid chromatography-mass spectrometry. **Results:** Preliminary results indicate that in these assays the engineered Vβ-TCRs and mAbs share similar levels of sensitivity and specificity, suggesting that the Vβ-TCRs could potentially supplant mAbs in SE detection assays. Bead-based assays were used to test PBS and milk samples spiked with purified SEB (0.1-7.0 ng/ml) from two different manufacturers. The enterotoxin was detected at all levels even at the lowest level tested, indicating that the sensitivity was lower than commercially available ELISA assays. **Conclusions:** Identification of contaminated food is essential for food safety. A critical element toward this goal is the availability of reliable and sensitive assays that can rapidly detect bacterial metabolites such as SEs. The use of monoclonal antibodies, and now, the engineered Vβ-TCRs allows for low level detection in a multiplex fluorescent bead-based assay to enable accurate identification and quantification of the staphylococcal enterotoxins.

**Board 15. Development and Application of a Multiplex Serological Assay for Avian Influenza with Microsphere Suspension Array System**

**Y. Wang**, W. Wong, M. Ng, C. Lim, Y. Lin, T. Huangfu;
Agri-Food&Veterinary Authority of Singapore, Singapore, Singapore.

**Background:** Avian Influenza (AI) is a highly contagious and economically significant disease in poultry. The spread of Highly Pathogenic Avian Influenza viruses further implicates its threats to public health. It is therefore critical to develop rapid and sensitive assays for effective AI surveillance. Microsphere-based immunoassays could potentially detect up to 500 analytes in one sample in one test. This multiplex capacity allows for high-throughput detecting and simultaneous subtyping of influenza virus infection. It is our aim to develop such an assay in this study. **Methods:** Competitive immunoassay format was adopted to allow testing of serum from different animal species. Recombinant AI virus proteins (antigens) were coupled to microspheres of different spectral addresses. The antigen coupled microspheres were then incubated with test serum samples, and subsequently with antigen specific monoclonal antibodies, biotinylated secondary antibody and phycoerythrin conjugated streptavaidin. The fluorescence intensity obtained is reversely proportional to the amount of specific AI antibodies in the samples. **Results:** Influenza A nucleoprotein (NP), and the H5, H7 and H9 haemagglutinin (HA) proteins were selected as our primary targets. Antigen coupling and assay conditions for each of the targets were optimized, and combinations of various antigen-antibody pairs were screened for minimal interference between different targets, broader coverage within each subtype, and better specificity. Assay validation was carried out with full panel of international reference serum samples. The developed assay requires only 5 µl of the test sample, and up to 500 samples can be tested within one day by a single operator. We are currently using this assay to screen serum samples from migratory birds and potentially wild urban birds. Results will be presented during the meeting. **Conclusions:** A multiplex serological assay for simultaneous detection of Influenza A NP, H5, H7 and H9 HA antibodies has been developed. It provides us with a more economical and efficient way for disease surveillance in poultry farms, as well as in migrate birds and wild urban birds. The method developed could also be useful for AI endemic countries in South East Asia and other regions for more effective monitoring and control of avian influenza.

**Board 16. A Modified Molecular Beacon and Fluorescent Signal Combination Assay for Detection of Multiple Foodborne Pathogens**

**Q. Hu**[1,2], D. Lv[2], Q. Li[3], X. Shi[1], Y. li[1], Y. li[1], Y. qiu[1];
[1]Shenzhen centre for disease control and prevention, Shenzhen, China, [2]School of life science, Shenzhen University, Shenzhen, China, [3]School of life science, Xiamen university, xiamen, China.

**Background:** To develop a set of modified molecular beacon coding/labeling and multicolor fluorescent signal combinational probes to detect seven foodborne pathogens, including *Salmonella*, *Escherichia coli* O157:H*7, Vibrio* parahaemolyticus*, Vibrio* vulnificus , *Campylobacter* jejuni, *Listeria* monocytogenes and *Staphyloccocus* aureus in a single real-time PCR assay. **Methods:** Based on the principle of the Homo-Tag Assisted Non-Dimer System and the fluorescent signal combination, a universal primer containing nucleotides at the 5′ end was designed to reduce the formation of dimers and used in combination with **s**even pairs of species-specific tagged primers and seven modified molecular beacon probes. Species-specific reagents were designed based on sequences of targeted genes obtained from GenBank. Four single-labeled probes, including *ssaR* gene, *hlyA* gene, *nuc* gene and *rfbE* gene and three dual-labeled probes, including *toxR* gene, *vvh* gene and *gyrA* gene and primers were added to a single reaction tube to detect up to seven foodborne pathogens. The assay was validated by detecting 435 purified bacterial strains previously identified by traditional culture methods. The sensitivity and specificity of the assay were evaluated by testing 23, 638 food and diarrheal stool samples from foodborne disease outbreak and compared the results to conventional microbial culture. **Results:** No false-negative results for

detection of the target genes were observed during the validation study. The limit of the detection of the assay was 1 - 100fg or 1 - 6.25 colony forming units/reaction depending on the organism. Compared with culture methods, sensitivity and specificity of the multiplex real-time PCR assay were 100% and more than 99% respectively. The rapid assay could be finished within two hours, providing a significant testing advance for foodborne pathogens detection. Conclusions: The highly sensitive, specific, and rapid multiplex real-time PCR assay developed and validated in this study was successfully applied to the rapid diagnosis of foodborne pathogens from food and human sources. We anticipate that the use of this methodology would greatly enhance public health investigation of causes of foodborne outbreaks and will be a valuable tool for laboratory surveillance of diarrheal diseases.

**Board 17. *Rickettsia parkeri* Infection Detected by Polymerase Chain Reaction Amplification from Eschar Swab Specimens: A Novel Diagnostic Approach for a Rare Condition**

**T. Myers**[1], T. Lalani[2], M. Dent[3], J. Jiang[4], P. Daly[2], J. Maguire[2], A. Richards[4];
[1]AFHSC/NMRC, Silver Spring, MD, USA, [2]Naval Medical Center, Portsmouth, Portsmouth, VA, USA, [3]Naval Air Station, Pensacola, FL, USA, [4]Naval Medical Research Center, Silver Spring, MD, USA.

**Background:** Until 2004, all confirmed cases of tick-borne spotted fever in North, Central, and South America were attributed to, *Rickettsia rickettsii*, the cause of Rocky Mountain spotted fever. However, in 2004, an US serviceman living in the Tidewater region of Virginia presented to an acute care clinic with fever, mild headache, and multiple eschars. *Rickettsia parkeri,* a tick-associated rickettsia was subsequently isolated in cell culture from an eschar biopsy, documenting the first recognized case of Tidewater spotted fever, also known as *R. parkeri* rickettsiosis. To date there have been over 25 cases diagnosed in the United States as well as South America. Laboratory diagnosis of *R. parkeri* infection is generally performed via serology or real-time PCR on serum and whole blood. Here we describe a novel, less invasive approach consisting of a real time *R. parkeri* specific PCR assay performed on a swab of an unroofed eschar. *R. parkeri* infection was diagnosed in two patients participating in an ongoing prospective study to determine the prevalence of *R. parkeri* among individuals with tick bite eschars or clinically diagnosed rickettsial illness. **Methods:** Both patients (one from Virginia and the other from Florida) presented with an eschar 8-10 days following a tick bite, associated with systemic symptoms including fever, chills and a diffuse maculopapular rash. The eschars were unroofed and swabs of the lesions were assayed via quantitative real-time PCR (qPCR) for *R. parkeri*. Interestingly, the patient's sample from Florida was obtained on a healing eschar 14 days post doxycycline treatment. **Results:** Both patients' swab samples were positive for *R.parkeri* DNA, and a 4 fold rise in antibodies (IgG) between acute and convalescent samples against spotted fever group rickettsia supported the diagnoses. **Conclusions:** To our knowledge this is the first report of diagnosing acute *R. parkeri* infection through less-invasive means, utilizing an eschar swab. This also represents the first report of obtaining positive PCR results from a healing eschar 14 days post antibiotic treatment.

**Board 18. Discovery and Validation of Prognostic Biomarkers for Sever Dengue by Proteomic Screening**

**B.K. Poole-Smith**[1], C. Straccini[2], A. Gilbert[2], B. Ward[2], M. Ndao[2], E. Hunsperger[1];
[1]Centers for Disease Control and Prevention, San Juan, PR, USA, [2]McGill University, Montreal, QC, Canada.

**Background:** Dengue is the most important viral vector-borne disease with more than 2.5 billion people at risk for dengue virus infection in over 100 tropical and sub-tropical countries. At least 500,000 people are hospitalized annually for dengue hemorrhagic fever (DHF), a more severe form of the disease, with fatality rates exceeding 5% without appropriate treatment. The onset of DHF occurs after the patient's fever subsides and the patient appears be recovering. Current diagnostic methods cannot predict which dengue patients develop DHF. A prognostic diagnostic test that identifies patients at risk for developing

DHF could significantly reduce dengue mortality and morbidity. We used surface enhanced laser desorption/ ionization time of flight (SELDI TOF/TOF) mass spectrometry to identify unique host biomarkers in patients with severe dengue. **Methods:** SELDI TOF/TOF assesses differences in proteins expression in healthy and diseased patients. In order to determine if there are unique biomarkers that can differentiate between dengue fever (DF) and DHF, we developed a panel of serum specimens that included patients with DHF, uncomplicated DF, other febrile illnesses, and healthy persons. These samples were analyzed using SELDI-TOF/TOF to identify variations in biomarkers. In addition, we tested serum from laboratory-confirmed cases of DENV serotypes 1-4 to determine if there was serotype-specific variation in biomarkers. Candidate biomarkers were further characterized by two-dimensional gel electrophoresis and mass spectrometry. **Results:** We identified 25 candidate biomarkers which could distinguish between DF and DHF and found no serotype-specific variation in DF biomarkers. To validate the most promising candidate biomarker vitronectin, we evaluated serum concentrations via ELISA. Vitronectin was found at significantly lower levels in serum from DHF cases compared to DF cases. **Conclusions:** The use of SELDI TOF/TOF screening for unique host biomarkers successfully identified vitronectin as a candidate. Vitronectin has the potential to differentiate between DF and DHF; with a significant reduction in DHF cases compared to DF. Further studies to determine the dynamics of this biomarker over the progression of DF to DHF will determine its utility as a prognostic marker for severe disease.

## Foodborne and Waterborne Infections

Monday, March 12
12:30 PM – 1:30 PM
Grand Hall

**Board 19. The Global Seasonality of Norovirus Gastroenteritis**

**S. Ahmed**[1], B. Lopman[2], K. Levy[1];
[1]Center for Global Safe Water, Department of Epidemiology, Rollins School of Public Health, Emory University, Atlanta, GA, USA, [2]Centers for Disease Control and Prevention, Atlanta, GA, USA.

**Background:** Norovirus is the leading cause of epidemic, acute gastroenteritis in industrialized countries, and may be a cause of severe disease in certain populations (e.g. children, elderly, and hospitalized patients). Norovirus is generally recognized to have an irregular wintertime seasonality in temperate climates, but these patterns have not been systematically described across geographic areas in the era of modern diagnostics. **Methods:** We present the results of a systematic review and meta-analysis of norovirus gastroenteritis across various geographic regions. In the systematic review we searched for publications that reported at least one full year of monthly data on norovirus outbreaks or cases in a specified geographic region. The initial literature search identified 287 potential publications, with 78 meeting the inclusion criteria. Data were then extracted from each publication using Plot Digitizer software. **Results:** The proportion of cases/outbreaks of norovirus per calendar month was calculated from studies representing twenty-two countries on four continents (34 case-based studies, 27 outbreak-based studies, representing a total of 12 years of data from temperate, Northern climates). When averaged over their entire study period, norovirus peaked most frequently in December (25% of case-based, 42% of outbreak-based) and January (33% of case-based, 17% of outbreak-based), with some studies showing peaks before December (8% of both case-based and outbreak-based) and others peaking after January (25% of both case-based and outbreak-based). All seasonal peaks occurred between November and March. We report results of regression analysis of the timing and intensity of seasonality as predicted by latitude, GDP, emergence of epidemic strains (2002 & 2006), and magnitude

of the previous norovirus season. **Conclusions:** Determinants of norovirus seasonality are likely to be multi-factorial, and this research provides a broad description of these patterns and the factors that may underpin them at a global level.

**Board 20. Nipah Transmission from Bats to Humans Associated with Drinking Traditional liquor (Tari) in Northern Bangladesh, 2011**

**M. Islam**[1], H. Sazzad[1], E. Gurley[1], S. Hasan[1], M. Rahman[2], S.P. Luby[1,3], M. Hossain[1];
[1]International Centre for Diarrheal Disease Research, Bangladesh, Dhaka, Bangladesh, [2]Institute of Epidemiology Disease Control and Research, Dhaka, Bangladesh, [3]Centers for Disease Control and Prevention, Atlanta, GA, USA.

**Background:** Nipah Virus (NiV) is a bat-borne emerging infection that causes fatal outbreaks of encephalitis almost yearly in Bangladesh. The most commonly described transmission pathway of NiV from fruit bats to humans in Bangladesh is through consumption of raw date palm sap, a national delicacy consumed during the cool, winter months. During January to March 2011, national encephalitis surveillance identified a cluster of 8 NiV cases in northern Bangladesh; 4 had a common exposure to traditional liquor (tari) produced from fermenting date palm sap. The aims of our investigation were to explore in detail case exposures and to understand tari production, consumption and selling practices. **Methods:** In July 2011, we conducted in-depth interviews and group discussions with family members and friends of the cases. We explored the tari making process and bats' access to the sap during fermentation through interviewing tari producers. **Results:** The four NiV cases regularly purchased and drank tari in the evenings from a common source prior to their illness. None of them had either a history of drinking fresh date palm sap or any exposure to sick humans or animals. Tari was produced commercially in the area and the producers harvested date palm trees throughout the year. They did not regularly clean sap collection pots but kept contents at the bottom of the collection pots to ferment the sap as it was collected in the tree overnight. The tari was ready for sale the next morning but producers reported filtering it with a net before selling. They occasionally found dead bats inside the tari pot. **Conclusions:** The process of harvesting date palm trees for tari was nearly identical to the process for fresh date palm sap collection. The tari was likely contaminated with bats' urine or saliva during collection and was the most likely source of NiV infection for these cases. The alcohol concentration may not have been high enough or sufficiently evenly distributed throughout the tari to sterilize the NiV. Date palm sap is collected for raw consumption only during the cool winter months but tari is collected year-round, possibly posing a risk for NiV transmission outside of the previously noted NiV season. Future research should look for tari exposures among suspected NiV cases, and evaluate the alcohol concentration in tari and its impact on NiV survival.

**Board 21. Toxigenic *Vibrio cholerae* O75 Infections in the United States, 2000-2011**

**A.E. Newton**[1,2], M. Parsons[1], C.A. Bopp[1], M. Freeman[1], S. Stroika[1], B.E. Mahon[1];
[1]Centers for Disease Control and Prevention, Atlanta, GA, USA, [2]Atlanta Research and Education Foundation, Decatur, GA, USA.

**Background:** Toxigenic *Vibrio cholerae* O75 was first reported as a cause of human illness in the United States in 2000. The first reported outbreak of toxigenic *V. cholerae* O75 infection occurred in 2011 and was associated with oyster consumption. **Methods:** State and local public health departments report demographic, clinical, and exposure information on toxigenic *V. cholerae* O75 infections to the Centers for Disease Control and Prevention (CDC) through the Cholera and Other *Vibrio* Illness Surveillance system (COVIS). Confirmation of species, serotyping, presence of cholera toxin, molecular subtyping by pulsed-field gel electrophoresis (PFGE), and antimicrobial susceptibility testing were performed at CDC. We reviewed data from January 2000 to September 2011. **Results:** From January 2000 to September 2011, 28 confirmed cases of toxigenic *V. cholerae* O75 infection were reported to CDC from 10 states:

Alabama (3), Florida (7), Georgia (5), Indiana (1), Kentucky (1), Louisiana (5), Ohio (1), Pennsylvania (3), South Carolina (1), and Tennessee (1). On average, 1-2 cases were reported annually from 2000-2009; 6 were reported in 2010 and 11 in 2011. Patients ranged in age from 12 to 80 years (median age 50 years); 46% were female, and 79% were white. All presented with diarrhea, 38% were hospitalized, and none died. Fifty-seven percent had at least one pre-existing condition. Seafood consumption in the 7 days before illness onset was reported for 93%, of whom 81% consumed oysters (86% were reported to be consumed raw). Twenty-nine percent had sufficient information to determine oyster harvest site; all were harvested in Apalachicola Bay, FL. All isolates were susceptible to all antimicrobials tested; 9 *sfi* and *not*1 unique PFGE pattern combinations were observed. **Conclusions:** Toxigenic *V. cholerae* O75 is an emerging cause of vibriosis in the United States. Despite the presence of cholera toxin, less than half of all patients were hospitalized, suggesting that relatively mild illness is not uncommon. Illness is associated with consumption of seafood, principally raw oysters, harvested from the Gulf Coast.

**Board 22. Forty-two Years of Laboratory-Based Surveillance for *Salmonella* Serotypes Causing Human Illness in the United States**

**K.E. Fullerton**, R. Bishop, P. Fields;
Centers for Disease Control and Prevention, Atlanta, GA, USA.

**Background:** *Salmonella* serotyping provides a consistent subtyping scheme that permits analysis of trends in human illness. Laboratory-based surveillance for culture-confirmed *Salmonella* infection started in the United States in the 1960s; information collected includes serotype, date of isolation, and patient's county and state of residence. Paper-based reporting was used from 1968 to 1994; electronic reporting was implemented in 1995. We summarize 42 years of national *Salmonella* surveillance data, including a geographic analysis of serotype distribution. **Methods:** We described the national distribution of *Salmonella* serotypes causing human illness reported from 1968 to 2009, using geographic analysis and standardized county-specific incidence rates (per 100,000 population) for 4 intervals: 1968-1994, 1995-1999, 2000-2004, and 2005-2009. **Results:** The top 5 *Salmonella* serotypes from 1968-2009 were Typhimurium (26%, var. 5-; included), Enteritidis (15%), Heidelberg (7%), Newport (7%), and Infantis (3%). Incidence of Typhimurium sharply declined starting in 1986 and continued through 2009. Conversely, Enteritidis incidence started to increase in the late 1980s, reflecting the national epidemic. Heidelberg incidence had a large increase in the early 1980s, with a peak in 1987; since then it has continually declined. Newport incidence rose dramatically in the late 1990s, with a peak in 2002. While Infantis is in the top 5 overall, it has steadily decreased since 1991. On an annual basis, the top 5 serotypes vary; notable recent increases include Javiana (since 1998) and I 4,[5],12:i:- (since 2004). Marked increases focused in specific geographic areas were seen for serotypes Enteritidis (in the northeast), Newport (in the southeast), and Typhimurium (in the northwest and the east). **Conclusions:** Laboratory-based surveillance for serotypes is a valuable epidemiologic tool for the understanding and control of salmonellosis. Geospatial analysis facilitates understanding of the changing epidemiology of different serotypes. While the top 5 reported serotypes shift each year, Typhimurium and Enteritidis remain the 2 most commonly reported serotypes in the United States, and the emergence of Javiana and I 4,[5],12:i:- are of particular note.

**Board 23. Cultivation of Mussels (*Perna perna*): A Possible Source of *Cryptosporidium* Transmitted to Human Beings**

**T.C. Bomfim**, G.F. Marine, M.C. do Couto, A.P. Sudré, M.D. Lima;
Universidade Federal Rural do Rio de Janeiro, Rio de Janeiro, Brazil.

**Background:** The ability to filter large amounts of water during their feeding process makes shellfish such as oysters and mussels capable of concentrating *Cryptosporidium* spp in their tissues. Thus, they constitute a source of infection for human beings, becoming bio-indicators of fecal contamination in

aquatic environments. Therefore, the concern about using marine mollusks as food is that the product quality is directly related to the environment where they are grown. **Methods:** The aim of the present study was to verify the presence of *Cryptosporidium* spp., by molecular techniques, in mussels (*Perna perna*) cultivated on the Guaíba Island, City of Mangaratiba, Rio de Janeiro state, Brazil, establishing potential risk factors for the intake of infected shellfish in the studied area. During 12 months were analyzed a total of 150 adult mussels, in size of slaughter and with 6 cm valve length in average. After sampling, each mussel was removed from its valve and had the digestive glands and gills crushed and centrifuged. Molecular techniques were applied in the sediment obtained from centrifugation. The genomic DNA was obtained from the samples sediment and was submitted to the primary PCR and the Nested-PCR using the gene SSUrRNA as a target. Positive samples were submitted to sequencing and phylogenetic analysis. **Results:** Samples sequenced and identified as *Cryptosporidium parvum* were submitted to a new Nested-PCR reaction using the gene GP60 (60 kDa glycoprotein) as a target, followed by sequencing and phylogenetic analysis. **Conclusions:** The profile obtained by the sequencing of mussels (*Perna perna*) samples containing *Cryptosporidium* collected in the State of Rio de Janeiro, Brazil, demonstrate that those findings sets a foodborne zoonosis, denoting risk of infection for consumers and for people who work with the cultivation in the aquatic environment and with processing the product.

**Board 24. Description of *Campylobacter* Cases Identified through Culture-Independent Methods and Their Impact on the Incidence of *Campylobacter* Infections, Foodborne Diseases Active Surveillance Network (FoodNet), 2010-2011**

**M.E. Patrick**[1], A. Cronquist[2], K. Wymore[3], J. Hatch[4], S. Solghan[5], T. Robinson[6], M. Tobin-D'Angelo[7], C. Nicholson[8], S. Hurd[9], O. Henao[1];
[1]Centers for Disease Control and Prevention, Atlanta, GA, USA, [2]Colorado Department of Public Health and Environment, Denver, CO, USA, [3]California Emerging Infections Program, Oakland, CA, USA, [4]Oregon Public Health Division, Portland, OR, USA, [5]New York State Department of Health, Albany, NY, USA, [6]Minnesota Department of Health, St. Paul, MN, USA, [7]Georgia Department of Public Health, Atlanta, GA, USA, [8]New Mexico Emerging Infections Program, Albuquerque, NM, USA, [9]Connecticut Emerging Infections Program, New Haven, CT, USA.

**Background:** *Campylobacter* is a common cause of gastrointestinal illness in the U.S. Current confirmed case definition requires culture; however, due to faster results and ease of testing, use of culture-independent tests is increasing. Some data suggest that culture-confirmed (CC) and culture-independent (CI) cases differ in their clinical spectrum of disease. We examined surveillance data to understand characteristics of CI cases and assess their impact on incidence rates. **Methods:** FoodNet has conducted active surveillance for CC *Campylobacter* infections in selected states since 1996. Since mid 2009, FoodNet has also ascertained cases diagnosed through culture-independent methods. In 2009 and 2010, FoodNet surveyed laboratories in the catchment area about the use of and types of culture-independent tests. We examined data from 2010 and 2011 and used US Census data to calculate incidence rates (IR). We assumed the sensitivity of culture-independent tests to be 98%. **Results:** A total of 6,372 CC and 530 CI cases were reported in 2010; as of September 2011, 4,576 CC and 432 CI cases had been reported. CI cases represented 8.3% of total cases in 2010 and 9.4% in 2011. CI cases were significantly more likely than CC cases to be older (median 48 vs. 37 years; p <0.0001), female (56% v. 45%; p<0.0001), and to have been hospitalized (36% vs. 17%; p<0.0001). The overall crude IR of CC cases in 2010 was 13.6 per 100,000; including CI cases increased the rate to 14.7. The change in IR was higher among females (12.0 to 13.2) than males (14.8 to 15.6), and highest among infants <1 year (23.6 to 27.0) and persons >80 years old (11.9 to 16.3). The use of culture-independent tests increased from 3.3% of labs surveyed in 2009 to 6.2% in 2010. **Conclusions:** The percentage of cases diagnosed through culture-independent methods appears to be increasing and this has an impact on incidence rates. A better understanding of

the reasons for differences in demographics and illness severity between CI and CC cases would help in interpreting surveillance data. Continued collection of surveillance data that include type of test used, outcome, and clinical presentation is important to better understand differences between CI and CC cases and inform decisions about possible changes in case definition.

## Effective and Sustainable Surveillance Platforms

Monday, March 12
12:30 PM – 1:30 PM
Grand Hall

**Board 25. National Hospital-Based Influenza Surveillance in Bangladesh, 2008–2011**

**A.A. Mamun**[1], R.U. Zaman[1], A.S. M. Alamgir[2], E. Azziz-Baumgartner[3], M. Rahman[2], M.S. Haider[1], M.Z. Rahman[2], M.A. Islam[1], T. Azim[1], K. Sturm-Ramirez[1,3], M. Rahman[2], S.P. Luby[1,3];
[1]International Centre for Diarrhoeal Diseases Research, Bangladesh, Dhaka, Bangladesh, [2]Institute of Epidemiology, Disease Control and Research, Bangladesh, Dhaka, Bangladesh, [3]Centers for Disease Control and Prevention, Atlanta, GA, USA.

**Introduction:** In 2007, IEDCR, ICDDR,B and CDC established national hospital based influenza surveillance in Bangladesh to characterize the epidemiology and diversity of circulating influenza strains and to identify clusters of people with severe influenza infection. Here we report surveillance data collected during January 2008-July 2011. **Methods:** Staff in each of the 12 participating hospitals identified outpatients with influenza-like illness (ILI, defined by WHO) and hospitalized persons with severe acute respiratory illness (SARI, aged ≥5 years with history of fever within 21 days and cough or sore throat) and severe pneumonia of aged <5 years (defined by WHO). Staff collected demographics and clinical illness information in hand held computers, and obtained nasal and throat swabs for rRT-PCR influenza testing and subtyping. **Results:** During January 2009-July, 2011, we collected specimens from 10,987 case-patients: 5,699 (52%) from ILI, 3,597 (33%) from SARI and 1,691 (15%) from severe pneumonia case-patients. Of all case-patients, 1,547 (14%) tested positive for influenza. Among the 5,699 ILI case-patients, 826 (17%) tested positive for influenza, of which 280 (34%) were influenza A/H3, 156 (19%) 2009 pandemic influenza A/H1 (pH1N1), 107 (13%) seasonal influenza A/H1 and 283 (34%) were influenza B. Among the 5,288 hospitalized case-patients, 721 (14%) tested positive for influenza, of which 297 (41%) were influenza A/H3, 266 (37%) pH1N1 and 158 (22%) were influenza B. No novel influenza strains were detected. The median age of the influenza positive case-patients was 18 years (range: 1 month - 100 years). There were 7 deaths among the 721 hospitalized influenza positive case-patients. In 2008, we observed influenza circulation from April to October. Although the influenza season started in April 2009 as expected, following the introduction of pH1N1, we did not see any distinct seasonality of influenza during 2009-2010. **Conclusions:** Both influenza A and B are circulating in Bangladesh, affecting all age groups. Despite an ongoing epidemic of H5N1 in poultry in Bangladesh, we have not detected any human cases through this national surveillance platform. Continued hospital surveillance is recommended to monitor influenza circulation and seasonality in Bangladesh.

**Board 26. Establishing Sustainable Influenza Surveillance in a Limited Resource Country, Bangladesh**

**M.S. Haider**[1], A. Alamgir[2], A. Chakraborty[3], K.M. Jamil[1], A. Al Mamun[3], K. Sturm-Ramirez[3,4], E. Azziz-Baumgartner[4], S.P. Luby[3,4], M. Rahman[1];
[1]Institute of Epidemiology, Disease Control and Research (IEDCR), Dhaka, Bangladesh, [2]World Health Organization (WHO), Dhaka, Bangladesh, [3]International Centre for Diarrhoeal Diseases Research (ICDDR,B), Dhaka, Bangladesh, [4]Centers for Disease Control and Prevention (CDC), Atlanta, GA, USA.

**Background:** Bangladesh was preparing for a potential influenza pandemic since 2005. With avian influenza infection in poultry and high human population density, characterizing circulating influenza virus was a priority. Since 2007, hospital based influenza surveillance started in Bangladesh jointly coordinated by IEDCR, ICDDR,B and CDC in 12 tertiary care hospitals across the country. Inspired by the successful use of this surveillance data to monitor the progression of the 2009 H1N1 Pandemic, the Government of Bangladesh committed to develop a sustainable influenza surveillance. **Methods:** IEDCR as the national influenza centre (NIC) initiated the national influenza surveillance Bangladesh (NISB) in May 2010 to identify and monitor circulating strains of influenza virus in Bangladesh. We conducted policy level advocacy to prioritize influenza surveillance in Bangladesh. The government provided funding to procure logistics and develop human resources to start the surveillance. We selected 14 government district hospitals distributed across the country according to their geographic locations and avoided overlap with other influenza surveillance sites. At each site, hospital physicians collect demographic and clinical information from influenza like illness (ILI) and severe acute respiratory illness (SARI) cases on two consecutive days per month. Laboratory technicians collect throat and nasal swabs in viral transport media (VTM) and samples are transported in cold boxes to IEDCR. Samples processed for influenza typing and subtyping by rRT-PCR at IEDCR's NIC laboratory. **Results:** From each site we trained two laboratory technicians on sample collection, storage and transportation. We also trained three physicians and district health manager on the surveillance methodology. During May 2010 to June 2011, we enrolled 1547 cases. We tested 865 samples and 134 (15%) were positive for influenza where 46 (34%) were influenza A and 88 (66%) were influenza B. All influenza A positive cases were subtyped as pandemic (H1N1) 2009. Due to lack of manpower, surveillance activities at three hospitals are not yet fully implemented. **Conclusions:** Sustainable influenza surveillance can be conducted by governments in limited resource countries if there is sufficient commitment, strong monitoring and supervision.

**Board 27. Evaluation of Active and Passive Surveillance Systems for Community-Associated *Clostridium difficile* Infections, Connecticut**

**T. Styles**[1], J. Brockmeyer[2], C. Lyons[3], J. Meek[3], J. Hadler[3], M. Cartter[2];
[1]CDC/OSELS/SEPDPO/EFAB/CT, Milford, CT, USA, [2]Connecticut Department of Public Health, Hartford, CT, USA, [3]Yale, Emerging Infections Program, New Haven, CT, USA.

**Background:** *Clostridium difficile* infections (CDI) are typically considered healthcare-associated. In recent years, community-associated (CA-) CDI in persons traditionally at low-risk (e.g., children, or peripartum women) have increased in occurrence in the US and Canada. During 2006, the Connecticut Department of Public Health (CDPH) made CA-CDI provider reportable, but not laboratory reportable. During 2009, the Connecticut Emerging Infections Program (EIP) began active population-based surveillance in 2 metropolitan areas; cases were identified through laboratory reports. We conducted an evaluation to determine impact and efficiency of having 2 systems and suggest improvements. **Methods:** Connecticut's passive (state-based) and active (EIP-based) surveillance systems were evaluated according to Centers for Disease Control and Prevention (CDC) Updated Guidelines for Evaluating Public Health Surveillance Systems. Direct comparisons were limited to CA-CDI cases from August 2009-December 2010 and the geographic area served by both systems. **Results:** Because chart reviews are needed for case classification and risk factor assessment, both systems are labor-intensive; the active system requires 2 full-time equivalent employees (FTEs) compared with 0.34 FTE for the passive system. The 2 systems use different case definitions, making comparisons difficult. Sensitivity of the passive system was ~23% for hospitalized patients with CA-CDI when compared with the active system. Demographic and risk factor data were similar between systems. Only the active (EIP) system collects risk factor information on illnesses among outpatients, inpatients, and long-term-care residents. Further, the active system obtains stool specimens for isolation and characterization, providing both data and stool specimens to the CDC as part of national EIP CDI surveillance. **Conclusions:** The statewide

system allows CDPH to track statewide trends, but consideration should be given to using the active system's case definition. To conserve resources, discontinuing chart review for risk factors in the statewide system should be considered. Continuation of the EIP active surveillance system is recommended because it adds to the national understanding of the population burden of CDI, and allows for analysis of isolates.

**Board 28. Temporal-spatial Analysis and Genetic Mapping: Applying Two Alternative Methods for Performing Hemagglutinin (HA) Drift Surveillance at the U.S. Air Force School of Aerospace Medicine's (USAFSAM) Department of Defense (DoD) Global Laboratory-Based Influenza Surveillance Program**

**C.A. Schlorman**[1,2], B.C. Connors[1,2], M.C. Anguiano[1,2], V.H. MacIntosh[1], K. Tastad[1,2];
[1]United States Air Force School of Aerospace Medicine, Patterson Air Force Base, OH, USA, [2]Henry M. Jackson Foundation for the Advancement of Military Medicine, Inc., Bethesda, MD, USA.

**Background:** The DoD Global Lab-based Influenza Surveillance Program at the USAFSAM performs typing and subtyping of influenza isolates from DoD populations, selected specimens undergo molecular characterization. Viral RNA of the HA1 region is sequenced, and a phylogenetic comparison is made between the resulting sequence and current vaccine. Findings are presented at the annual Vaccines and Related Biological Products Advisory Committee of the Food and Drug Administration meeting where components for the next season's flu vaccine are chosen. **Methods:** Sequencing and epidemiological data for the period of Oct 2004 to Sep 2011 obtained from DoD populations were used. The dataset consisted of 1,601 influenza A (H3N2) viruses with the HA1 segments sequenced and analyzed. We hypothesize that the spatiotemporal genetic distances of HA1 drift between DoD populations are correlated with geographic distances, as the result of the unique situation of military environments and populations. Spatial statistical methods were combined with genetic analytic techniques to explore genetic evolution of influenza A (H3N2) viruses at the HA1 gene location. Additionally, a genetic mapping model [Smith et al., Science 305, 371-376], was used that enables a reliable quantitative interpretation of genetic drift data and eases the visualization and interpretation of genetic data. **Results:** Both modeling techniques provided unique representations of the HA1 nucleotide sequence data that the DoD program has not utilized in the past. The results matched well with the phylogenetic comparison. Our results document the potential effect of space and time on genetic evolution of the H3 influenza virus. The initial analysis of 1,601 influenza A (H3N2) viruses with HA1 segments from DoD population suggest that both modeling techniques increase the value of surveillance data. **Conclusions:** This exploratory study may give insight into processes underlying viral evolution and may suggest that genetic differentiation is associated with geographic distance in military populations. It should help facilitate the selection of vaccine strains for seasonal influenza and better recognition of potential divergence from current seasonal influenza vaccine. These were novel methods for the USAFSAM surveillance team.

**Board 29. Comparison of *Salmonella* Data Submitted to the National Notifiable Diseases Surveillance System, National *Salmonella* Surveillance System, PulseNet, and FoodNet for the Years 2007, 2008, and 2009**

**D. Sheehan**[1], T. Stiles[2], D. Bopp[3];
[1]APHL, Barrington, RI, USA, [2]Hinton State Laboratory Institute, Jamaica Plain, MA, USA, [3]Wadsworth Center, NYDOH, Albany, NY, USA.

**Background:** *Salmonella* infections are nationally notifiable. With one exception, state laws mandate that cases be reported to state and local health departments and to the Centers for Disease Control and Prevention (CDC). *Salmonella* surveillance information is submitted to CDC though different systems. Demographic information from all cases of *Salmonella*, independent of culture confirmation, is reported to the National Notifiable Diseases Surveillance System (NNDSS). All cases that are culture-confirmed by

state public health labs are reported to the CDC National Salmonella Surveillance System (NSSS). Isolates further subtyped by PFGE are submitted to the PulseNet database. In addition, 10 states participate in FoodNet, an active surveillance system pursuing additional information on all Salmonella cases. These 10 sites represent approximately 15.2% of the US population. FoodNet data is considered to be the most accurate reflection of the incidence of foodborne illness because it is active foodborne disease surveillance. **Methods:** This study compared *Salmonella* cases reported to each system for 2007 through 2009. NNDSS data were not broken down by serotype and were not analyzed further. Data from the remaining systems were analyzed for prevalence of serotype, changes over time, and reporting gaps. Both NSSS and PulseNet had a large number of isolates with unknown or unconfirmed serotypes. **Results:** When PulseNet data were compared to NSSS data for years 2008 and 2009, it was noted that a number equal to approximately half of all isolates submitted to NSSS as “unknown serotype” were grouped into a specific serotype based on PFGE pattern. In 2008, 5,639 isolates were sent to NSSS with no serotype data, but 2,675 isolates without serotype data in PulseNet were assigned a serotype specific PFGE pattern. **Conclusions:** Some variations among serotypes were reflected in all three systems, whereas others were not. For most serotypes, the total submitted to NSSS were approximately the same as those submitted to PulseNet. Overall, several discrepancies and several areas for improvement were observed between the systems analyzed.

**Board 30. US Department of Navy Comprehensive Influenza Report for the 2011-2012 Influenza Season**

**T. Luse**, A. McCabe, G. Nowak;
Navy and Marine Corps Public Health Center, Portsmouth, VA, USA.

**Background:** Since the 2007-2008 influenza season, the EpiData Center (EDC) at the Navy and Marine Corps Public Health Center has monitored influenza laboratory testing and results at US military laboratories, as well as pharmacy antiviral drug transactions, for Department of Navy (DON) beneficiaries around the globe. A weekly report was created for dissemination among all DON stakeholders to inform them of any changes among laboratory or pharmacy trends. While feedback for the report was positive, the report was not a comprehensive overview of influenza impact or burden on DON beneficiaries; stakeholders had to utilize other sources or reports, including some generated outside the DON, to make an assessment of mission and clinical impact. **Methods**: To better centralize influenza surveillance, as well as provide a comprehensive overview of disease impact, the EDC has developed a new dynamic, weekly report for the 2011-2012 season that will provide an all-inclusive overview of the influenza season. In addition to the laboratory and pharmacy data previously utilized, EDC added multiple sources to enable a more complete evaluation of the disease impact. **Results**: New information available for the report included outpatient encounter data for Influenza-like-illness tracking, inpatient admission records to assess incidence of potentially severe cases, immunization coverage for US military active duty and reserve service members, monitoring of influenza medical event reports in accordance with DOD instructions, trends in the type of laboratory testing ordered by providers (e.g. rapid diagnostics, cultures), disease comorbidities among inpatient influenza laboratory positive cases, bacterial coinfections with influenza within two weeks, monitoring of radiology data to identify pneumonia among laboratory positive cases, and influenza news items of interest. **Conclusions:** The new report will allow for a ‘one-stop shop’ in which stakeholders can get up to date information during the influenza season.

## Healthcare-Associated Infections

Monday, March 12
12:30 PM – 1:30 PM
Grand Hall

**Board 31. Incidence of and Risk Factors for Nosocomial Diarrhea in Tertiary Care Hospitals in Bangladesh, 2007-2010**

**M.U. Bhuiyan**[1], S.P. Luby[1,2], R.U. Zaman[1], M.W. Rahman[1], M.J. Hossain[1], M. Rahman[3], K.S. Ramirez[1,2], E.A. Baumgartner[1,2], E.S. Gurley[1];
[1]International Centre for Diarrheal Disease Research, Bangladesh, Dhaka, Bangladesh, [2]Centers for Disease Control and Prevention, Atlanta, GA, USA, [3]Institute of Epidemiology Disease Control and Research, Dhaka, Bangladesh.

**Background:** Prolonged use of antibiotics often causes nosocomial diarrhea among hospitalized patients worldwide. In low-income hospital settings where infection control is poor, patients may have an additional risk for developing nosocomial diarrhea by acquiring gastrointestinal pathogens. This study aimed to determine incidence and risk factors for nosocomial diarrhea in tertiary care hospitals in Bangladesh. **Methods:** We defined nosocomial diarrhea as passage of ≥ 3 liquid stools per day after 72 hours of hospitalization. During April 2007-April 2010, surveillance physicians at three tertiary care hospitals identified patients in adult medicine and pediatric wards who developed nosocomial diarrhea. We compared these patients to randomly selected patients from the same wards who stayed >72 hours but did not develop diarrhea. We calculated the incidence of nosocomial diarrhea per 1000 patient days at risk and counted clusters of nosocomial diarrhea, defined as occurrence of ≥ 3 diarrhea cases within a week in a single ward. **Results:** A total of 23,004 patients were hospitalized for >72 hours in these study wards representing 80,398 patient days at risk and 1% (N=257) of these patients developed nosocomial diarrhea. The incidence of nosocomial diarrhea was higher in pediatric than adult wards (3.9 vs 2.7 per 1000 patient days at risk, p<0.001) and higher among infants than older children (OR=4.4, 95% CI=2.6-7.4). Children with diarrhea were less likely to have received antibiotics within 72 hours of admission than children without diarrhea (OR=0.4, 95% CI=0.2-0.6). Among adults, we did not find association between nosocomial diarrhea and exposure to antibiotics, age of patient or length of hospital stay. We identified 19 clusters of nosocomial diarrhea; 13 (68%) clusters occurred in pediatric wards. Three (4%) of 78 infants with nosocomial diarrhea died. **Conclusions:** Infants were at highest risk for nosocomial diarrhea and associated death. The reduced risk associated with antibiotic use and frequent clustering of cases suggests that exogenous introduction of gastrointestinal pathogens could have caused illness in children. Further studies to explore the possible ways of exposure to gastrointestinal pathogens could help to develop preventive measures for nosocomial diarrhea in these settings.

**Board 32. Risk of Infection from the Physical Environment in Bangladeshi Hospitals: Putting Infection Control into Context**

**N.A. Rimi**[1], R. Sultana[1], M.S. Islam[1], M. Uddin[1], M. Sharker[1], N. Nahar[1], S.P. Luby[1,2], E.S. Gurley[1];
[1]International Center for Diarrheal Disease Research, Bangladesh (icddr,b), Dhaka, Bangladesh, [2]Centers for Disease Control and Prevention (CDC), Atlanta, GA, USA.

**Background:** Hospital environment is associated with risk of transmission of infectious diseases through airborne particles, respiratory droplets or contact with patient fluids. International infection control guidelines exist, but assume a level of basic infrastructure that may not be present in low income settings. This study describes the physical environment of hospitals in Bangladesh to understand the risks and opportunities to control infection. **Methods:** From March to September 2007, 5 social scientists conducted 51 hours of observation in 22 sessions in 1 pediatric and 1 adult male medicine

ward in 3 tertiary hospitals to record environmental contamination with patient secretions and medical waste and observe handwashing and use of personal protective equipment. We also recorded the number of patients, family caregivers, visitors and staff at the beginning and end of each session, measured the ward area and informally observed waste disposal outside the wards. **Results:** We recorded a mean of 5 (95% CI: 4, 7) uncovered coughs or sneezes per 100 square feet per hour and observed only one person using a mask. The floors were soiled with saliva, mucous, vomitus and blood a mean of 3 times per hour. Among 29 potential handwashing stations, only 7 had running water and soap, which were exclusively for staff use. Soap was used to wash hands only 0.6 times per hour. Use of nebulizer occurred 0.2 times per hour and no disinfection was observed before or after use. We observed a median of 7 (95% CI: 5, 9) people per 100 square feet in the wards. Used medical supplies were often discarded in open containers under the beds. At two hospitals, waste was later disposed of on the grounds outside the hospitals where people collected them to resell. Mosquitoes and feral cats were commonly observed in the wards. **Conclusions:** Overcrowded hospital environments pose a threat of infection for all patients, family caregivers and hospital staff, particularly through contact with contaminated hands or objects. Interventions focused solely on education or training without improving infrastructure are unlikely to improve infection control in these hospitals. Improving access to handwashing stations and promoting proper disposal of medical waste in combination with behavior change communication could reduce the risk of disease transmission.

**Board 33. Detection and Control of a Pertussis Outbreak in a Medical School and University Hospital**

**K. Tokuda**;
Tohoku University Graduate School of Medicine, Sendai, Japan.

**Background:** Health care workers (HCWs) would be under the risk of contracting pertussis in both their daily activities and communities. Booster immunization for adolescents and adults had not been introduced in Japan. In July 2007, we investigated a pertussis outbreak in a medical school and university hospital in Kochi prefecture in western part of Japan. No local epidemic had been reported from sentinel pediatricians. **Methods:** We conducted a questionnaire survey which targeted the period from 1April to 8 August 2007, active surveillance from 9 August to 8 September 2007, and active case finding for all students and staff members. A case was defined as a person who developed pertussis-like illness (PLI), such as paroxysms of cough and inspiratory whoop with a positive result of either culture or PCR test by a retrospective questionnaire survey and active case finding for all students and staffs. A retrospective cohort study was conducted to evaluate risk factors for infection. **Results:** A total of 189 cases were detected (Incidence Rate [IR]=9.3%). Index case could not be specified. The interview with cases in early stage of the outbreak revealed that most cases had contact with persons having PLI in communities. The IR in surgery-related departments was significantly higher than that in internal medicine-related departments ($p<0.05$). In general, the number of nurse-cases was strongly related to the number of student-cases (Correlation Coefficient [$R^2$]=0.925). A cohort study showed that the risk in practical training in surgery was significantly higher in student-cases than in other students (RR=4.3, 95%CI=2.7-6.9). Cases rapidly decreased after introducing chemoprophylaxis, school closure and restricting work of symptomatic staff members. There were no inpatient-cases due to secondary transmission.
**Conclusions:** Chemoprophylaxis, school closure and restricting work of symptomatic staff members were effective measures to prevent the further spread. This outbreak was probably reflected to local epidemics, which was not identified by sentinel surveillance system only by limited pediatricians. Case-based surveillance for pertussis including adolescents and adults should be introduced nationwide in Japan. Booster pertussis immunization for adolescents and adults should be considered, too.

**Board 34. Knowledge, Attitudes and Practices of Influenza Vaccination among Health-Care Providers in Kashmir (India)**

**N.K. Bali**[1], M. Ashraf[1], F. Ahmad[1], U.H. Khan[1], M.-A. Widdowson[1], R.B. Lal[2], P.A. Koul[1];
[1]SheriKashmir Institute of Medical Sciences, Srinagar, India, [2]Centers for Disease Control and Prevention, Atlanta, GA, USA.

**Background**: The acceptance for influenza vaccination among health-care providers (HCPs) is low. The objective of this study was to explore knowledge, attitudes, and practices associated with influenza vaccination in HCPs in Kashmir, a temperate climate region in the north Indian state of Jammu & Kashmir. **Materials and methods**: From April to June 2010, a self-administered questionnaire was distributed to 1750 HCPs (1465 medical professionals; 285 support staff members) in 3 major hospitals of Kashmir, and information sought on history of previous influenza vaccination as well as motivations, perceptions, and preferences. **Results:** Of 1750 distributed questionnaires, 1421 (81%) were completed. Ninety-five percent (n=1348) of respondents considered influenza capable of adverse consequences for themselves, their family or patients; 1204 (85%) considered it a potentially severe disease. While 1144 (81%) participants were aware of a vaccine against influenza, and 830 (58%) of its local availability; only 62 (4.4%) had actually ever received the vaccine in past 5 years. The reasons cited for not getting routinely vaccinated (n=1359) included ignorance of vaccine availability (460; 34%), skepticism about its effectiveness (261; 19%), inability to find time for vaccination (169; 12%), fear of side effects (71; 5.25%) and a perception of low personal risk for influenza (86; 6%). Although 1330 (94%) participants believed that vaccine programs are generally beneficial, 865 (61%) believed them to be motivated by profit. Also 684 (48%) believed that the vaccine could cause unknown illness, 444 (31%) believed vaccine adverse effects were underreported, and 83 (6%) considered vaccination unsafe. A majority (88%) believed that vaccination should be mandatory for HCPs dealing with high risk patients and must be provided conveniently as part of an employee health program. More than 75% (n=1178) wanted to get vaccinated in the current year. **Conclusions:** Influenza vaccination coverage levels are very low among HCPs in the northern Indian region of Kashmir; poor knowledge of vaccine availability, effectiveness and safety being important barriers. Multifaceted and adaptable measures need to be invoked urgently for a uniform and equitable influenza vaccination among these HCPs.

**Board 35. Outbreak of Rapidly Growing Mycobacteria Infection Post Video-assisted Gastroenterology Surgery–Manaus City, Brazil, 2010**

**G.B. VILLAR**[1], F.S. Bordalo[1], T.C. Ramos[2], V. Pereira[2], M. Cordeiro[2], F.T. Freitas[1], W.N. Araujo[1], V.M. Souza[1];
[1]Brazilian Field Epidemiology Training Program (Episus), Secretariat Surveillance in Health, Ministry of Health, Brasilia, Brazil, [2]Foundation of Health Surveillance, Manaus-AM, Brazil, Manaus, Brazil.

**Background**: Since 2004 Brazil has been faced outbreaks of rapidly growing mycobacteria infection (RGM) that occurred in different states presenting a single mycobacteria clone. In June 2010, the epidemiologic surveillance center in Amazonas state reported to the Brazilian Ministry of Health three suspected cases of RGM after video-assisted surgery. In August, Brazilian FETP team joined an ongoing investigation intending to identify extent, risk factors, and agent involved. **Methods**: We conducted a descriptive and retrospective cohort study. Cases were defined as completed procedures of video-assisted gastroenterology surgery with team-1 in a hospital from July 2009 to August 2010 with clinical findings suggestive of RGM infection. Relative risk (RR) was used as measure of association, with $\alpha$=5% and 95% confidence interval (CI). We conducted bivariate and logistic regression analysis (adjusted by sex and age group), to those variables with p<0,20. **Results**: The attack rate was 27% (60/222). Among infected patients the median age was 40 (range: 20-82) years and 72% (159) was female, 11 (18%) patients had mycobacterial culture positive, with *M. massiliense* identified in 27%, and 49 (82%) presented clinical symptoms (95% secretion; 86% local pain; 71% hyperemia) or suggestive histology.

Median incubation period was 30 (range: 7-150) days. Risk factors independently associated were: surgery longer than 60 minutes (RR=2.4; 95%CI=1.2-4.5), does not be the first surgery in the day (RR=2.7, 95%CI=1.4-4.3). No new cases were reported after May 2010, when new reprocessing instruments measures were adopted. **Conclusions**: Hospital infection outbreak occurred, caused mainly by *M. massiliense*. Longer surgeries and inadequate reprocessing conditions after each surgery were identified as risk factors. We recommend efficient mechanical cleaning of instruments and sterilization in autoclave.

## Molecular Epidemiology

Monday, March 12
12:30 PM – 1:30 PM
Grand Hall

**Board 36. Evidence of Multiple Zoonotic Transmission Events Among Group A Rotaviruses Suggests Continuous Generation of Novel Recombinant Strains in Eastern India**

**A. Mukherjee**, S. Mullick, M. Chawla-Sarkar;
National Institute of Cholera and Enteric Diseases, Kolkata, India.

**Background:** Due to close proximity of human and cattle in rural areas of developing countries like India, interspecies transmission is a major source of rapid generation of recombinants and genetic or antigenic variants. In course of study to gather more information on zoonotic transmission and genomic diversity of rotavirus, complete surveillance study as well as full genomic analysis of few human group A rotavirus strains from Eastern India, Manipur and Kolkata, were done after exhibiting the evidence of animal-like rota-viral infections. **Methods:** Fecal specimens were collected from children with acute diarrhea aged <60 months from BC ROY children's hospital, Kolkata and RIMS, Manipur. Rotavirus antigen was confirmed by ELISA and RNA-PAGE. Then, RT-PCR followed by multiplex semi-nested PCR was done. After sequencing, the nucleotides and deduced amino acids were analyzed for characterization. **Results:** During the surveillance for viral diarrhea in children at Dr. B.C. Roy Memorial Hospital for Children, Kolkata, West Bengal, increasing number of G9 strains were found (10.1%) since 2005. In 2007, one human group A rotavirus G9P[6] strain mcs/13-07, detected from a three year old child, revealed a VP8* segment closely related to porcine P[6] strains of sublineage ID in phylogenetic analysis. To further characterize the evolutionary diversity of strain mcs/13-07, all gene segments were analyzed. Gene segments VP6 and NSP4 exhibited genetic relatedness to Wa-like human subgroup II strains while VP1-3, NSP1-3 and NSP5-6 were closely related to porcine strains. During study on infantile rotavirus at RIMS, Manipur, almost 50% of children admitted with severe diarrhea, group A rotavirus was the predominant etiology. In Manipur, G1P[8] and G2P[6] were the most predominant (36% and 22%) followed by G12P[6] (8%) and G9P[6] (3%). Other than G9P[19], few unusual genotypes G4P[4], G10P[6] and G4P[6] were also identified at low frequency (<0.5%) with a porcine or bovine origin. Zoonotic transmission was confirmed since NSP4 gene of G4P[4] or [6] and G9P[19] clustered with genotype B of porcine strains, whereas G10 strain revealed NSP4 of genotype A with bovine characteristics. **Conclusions:** Thus, detection of these reassortant strains provided further evidence for frequent complex interspecies transmission events in developing countries, a fact taken into consideration during designing of next generation rotavirus vaccines.

**Board 37. Molecular Evolution of GII-4 Norovirus Strains**

**D.J. Allen**, K. Zakikhany, D. Brown, M. Iturriza-Gomara;
Health Protection Agency, London, UK.

**Background:** Human noroviruses are a genetically diverse group of RNA viruses that cause sporadic cases and outbreaks of gastroenteritis among all ages. Genogroup II-genotype 4 (GII-4) norovirus strains dominate worldwide, and are associated with epidemics and seasonal outbreaks. We have previously shown through analysis of the hypervariable P2 region of the capsid protein that a reservoir of random genetic diversity is maintained in the circulating norovirus population. We predicted that antigenically novel GII-4 strains emerge from this reservoir through a selective process mediated by host immunological pressure. Our data suggests that selection is focused on two surface-exposed epitopes (Site A/ Site B) in the P2 domain. **Methods:** We aimed to further characterise the diversity at these epitope sites among circulating GII-4 norovirus strains. We developed a sequencing-by-synthesis method specifically targeting the Site A/Site B epitopes, removing the need to sequence long regions of the capsid gene, allowing rapid and economical high throughput testing of large numbers of strains. We then screened 918 GII-4 norovirus isolates from clinical specimens collected 2006-2011. **Results:** Sequence analysis revealed 65 different Site A/Site B amino acid motif combinations. Motif TQE-STT was most frequently detected in 2006. A strain replacement event occurred 2006-2007, after which motif SRN-STT became most frequently detected. We found that Site A motifs (*n*=20) could be grouped into 3 antigenic clusters (1, 2, and 3) based on surface area profiles, and the viruses within each predicted to be antigenically similar. Transition between these antigenic clusters was observed: cluster 2 became more frequently detected; cluster 1 became less frequently detected; and between 2008-2010 cluster 3 was lost from the circulating virus population. **Conclusions:** The change in frequencies of detection of clusters 1 and 2 was concomitant with an increased number of outbreaks of GII-4 norovirus-associated gastroenteritis in 2009, suggesting that the increase in frequency of cluster 2 viruses may represent a population of antibody escape mutant viruses, and may have contributed significantly to the increase in case numbers in 2009.

**Board 38. Single Endemic Genotype of Measles Virus Continuously Circulating in China for at Least 16 Years**

**Y. Zhang**[1], S. Xu[1], H. Wang[1], Z. Zhu[1], Y. Ji[1], P. Rota[2], D. Featherstone[3], Y. Jee[4], W. Bellini[2], W. Xu[1];
[1]National Institute for Viral Disease Control and Prevention, China CDC, Beijing, China, [2]Centers for Disease Control and Prevention,, Atlanta, GA, USA, [3]Immunization, Vaccines and Biologicals, World Health Organization, Geneva, Switzerland, [4]Expanded Programme on Immunization, Western Pacific Regional Office, World Health Organization, Manila, Philippines.

**Background:** The WHO Regional Committee of the Western Pacific Region (WPR) formally declared a measles elimination goal in 2005 and established a target date of 2012 for regional measles elimination. In China, Measles virus (MeV) surveillance has been established since 1993. Molecular surveillance is most beneficial when there is continuous high quality surveillance and it is possible to observe the change in viral genotypes over time in a particular geographical region. **Method:** The incidence of measles in China from 1991 to 2008 was reviewed, and the nucleotide sequences from 1503 measles viruses during 1993 to 2008 were phylogenetically analyzed. **Results:** The results showed measles epidemics peaked approximately every 3 to 5 years with the range of measles cases detected between 56,850 and 140,048 per year. The Chinese measles virus (MeV) strains detected represented three genotypes; H1, H2 and A. Genotype H1 was the predominant genotype throughout China continuously circulating for at least 16 years. Genotype H1 sequences could be divided into two distinct clusters, H1a and H1b. Within the standard 450 nucleotide sequencing window in the N gene, a 4.2% average nucleotide divergence was found between the H1a and H1b clusters, and the nucleotide sequence and predicted amino acid homologies of H1a viruses were 92.3%-100% and 84.7%-100%, H1b were 97.1%-100% and 95.3%-100%, respectively. Viruses from both clusters were distributed throughout China with no apparent geographic restriction and multiple co-circulating lineages were present. Cluster H1a and H1b viruses were co-circulating during 1993 to 2005, while no H1b viruses were detected after 2005 and

the transmission of that cluster has presumable been interrupted. Analysis of the nucleotide and predicted amino acid changes in the N proteins of H1a and H1b viruses showed no evidence of selective pressure. **Conclusions:** This study investigated the genotype and cluster distribution of MeV in China over a 16-year period to establish a genetic baseline before MeV elimination in Western Pacific Regional Office (WPRO). Continuous and extensive MeV surveillance and the ability to quickly identify imported measles cases will become more critical as measles elimination goals are achieved in China in the near future.

**Board 39. Emerging Rickettsioses in Thailand, Molecular Evidences**

**J. Gaywee**, T. Ruang-areerate, P. Sakpaisal, K. Youngpakool, W. Rodkvamtook, N. Sangjun, N. Kuttasingkee, K. Somsri, P. Kaewsatien, L. Kumtim;
Armed Forces Research Institute of Medical Sciences, Bangkok, Thailand.

**Background:** Rickettsioses are a major infectious disease problem throughout the world, including Thailand. To gain essential information regarding incidence, causative species, and the transmission cycle of these diseases, we have launched a rickettsioses surveillance program since 2003. **Methods and Results:** To identify which rickettsial species circulating in Thailand, during 2003-2007, 718 febrile patients from the upper regions of Thailand were tested for rickettsia infection utilizing in-house developed molecular tool. 9% were infected with *Rickettsia spp.* and 13% with *Orientia tsutsugamushi,* respectively. DNA sequence analysis revealed the causative species were *Rickettsia typhi*, a flea-borne Typhus Group, *R. honei, R. felis, R. rickettsii* and *R. japonica*, a tick-borne Spotted Fever and *O. tsutsugamushi* 10 clustered strains. We clearly demonstrated that flea-borne, tick-borne and mite-borne rickettsiae are distributed in Thailand. To understand how these pathogens are transmitted to humans, reservoirs and vectors were investigated. Focusing areas along Thai borders, wild rodents were captured and blood suckling arthropods were collected. From April 2008 to September 2010, 211 clones of arthropods including twelve species of fleas, lice, and ticks from different hosts and locations were evaluated. Rickettsial genes were detected in 65.7% (92/140) and 71.8% (51/71) of arthropods from Thai-Myanmar and Thai-Cambodia borders, respectively. *R. japonica*, *R. rickettsii* and *R. massiliae* were detected in *Dermacentor, Rhipicephalus* and *Haemaphysalis* ticks collected from humans and dogs. Spotted fever group *Rickettsia* Cf 1, Cf 5 and SE 313 were also detected in fleas and lice collected from dogs, cats, cattle and chickens. These findings indicate the presence of rickettsial vectors in border areas, thus the endemic foci for rickettsioses. **Conclusions:** This information is useful for implementing an effective disease prevention and control program. Furthermore, the availability of new local isolates may lead to the development of more sensitive and specific diagnostic tools.

**Board 40. Application of MLVA to an Outbreak of *E. coli* O157:H7 Associated with Walnuts in Canada, 2011**

**C. Goodman**[1], A. Hexemer[2], L. Tschetter[1], A. Kearney[1], A. Maki[3], S. Bekal[4], J. Reid[5], C. Nadon[1];
[1]Public Health Agency of Canada, Winnipeg, MB, Canada, [2]Public Health Agency of Canada, Guelph, ON, Canada, [3]Public Health Ontario, Toronto, ON, Canada, [4]Institut national de santé publique du Québec, Sainte-Anne-de-Bellevue, QC, Canada, [5]New Brunswick Region 2 Reference Centre, Saint John, NB, Canada.

**Background:** Pulsed-field gel electrophoresis (PFGE) is the primary tool for characterizing *E. coli* O157:H7 in Canada; all human clinical isolates are subtyped by PFGE in real time as part of the PulseNet Canada surveillance network. While this method demonstrates good discriminatory power and epidemiological concordance, additional discriminatory power is often helpful for public health investigations. To address this, multi-loci variable number tandem repeat analysis (MLVA) has been developed and extensively applied in the United States and Europe. An outbreak of *E. coli* O157:H7 during 2011 marks the first time MLVA was applied to an outbreak investigation in Canada. **Methods:** PFGE was performed

on all isolates by provincial public health laboratories and analyzed against the central PulseNet Canada national database. Isolates with a PFGE pattern matching or highly similar to the case definition were selected for MLVA analyses following the standardized PulseNet USA method. **Results:** A total of 19 cases had matching or similar PFGE patterns during the timeframe of the investigation. Of those, 14 cases from 3 provinces matched by PFGE and met the laboratory case definition. Thirteen shared the same MLVA profile. One case with matching PFGE had a MLVA profile differing from the main outbreak profile by one repeat at one locus. The cases with similar PFGE patterns had MLVA profiles differing by multiple repeats at up to 6 loci. This outbreak was epidemiologically linked to the consumption of shelled walnuts. Several months after the outbreak, cases with outbreak PFGE pattern appeared again; however, the MLVA profile of these cases differed significantly from the walnut-associated outbreak. **Conclusions:** MLVA confirmed the PFGE results in this outbreak and added strength to the laboratory component of the investigation, and was also useful in differentiating this outbreak from a temporally similar cluster. This investigation clearly demonstrates the powerful potential of this tool to not only support PFGE findings but to help differentiate distinct outbreaks that can occur simultaneously with similar or matching PFGE profiles. However, it also highlights the need for Canada to develop interpretation guidelines based on empirical evidence on strains of *E. coli* O157:H7 occurring in Canada.

## Antimicrobial Resistance

Monday, March 12
12:30 PM – 1:30 PM
Grand Hall

**Board 41. Antimicrobial Susceptibility Monitoring of Respiratory Tract Pathogens Isolated from Diseased Cattle and Swine across Europe between 2004 and 2006**

**A. de Jong**[1], S. Simjee[1], V. Thomas[1], K. Maher[2], I. Morrissey[2], P. Butty[1], H. Moyaert[1], U. Klein[1], H. Marion[1], D. Rigaut[1], B. Schiessl[1], M. Vallé[1];
[1]VetPath Study Group, Brussels, Belgium, [2]Quotient Bioresearch, Fordham, UK.

**Background:** VetPath is an ongoing pan-European resistance monitoring program, in place since 1998, for veterinary pathogens isolated from diseased antimicrobial-naive cattle, pigs and poultry. Susceptibilities of pathogens isolated from cattle and swine respiratory tract infections are presented here. **Methods:** Lung samples or either nasopharyngeal or nasal swabs were collected from animals with acute clinical signs, not recently treated with antimicrobials, in 11 EU countries. *Pasteurella multocida* (*Pm*) and *Mannheimia haemolytica* (*Mh*) from cattle and *Pm*, *Actinobacillus pleuropneumoniae* (*Ap*) and *Streptococcus suis* (*Ss*) from swine were isolated (one isolate/farm/outbreak). Antimicrobial susceptibility was studied for 17 antibiotics in a central laboratory by broth microdilution as per CLSI recommendations. Results were interpreted using CLSI resistance breakpoints (M31-A3, 2008) where available. **Results:** In all, 561 isolates were recovered. In cattle, 82 *Mh* and 105 *Pm* were isolated, the majority of these were susceptible to antibiotics for which a CLSI resistance breakpoint is available. Resistance of *Pm* and *Mh* to ceftiofur, enrofloxacin, amoxicillin/clavulanic acid and co-trimoxazole was absent. Florfenicol (*Pm*) and tilmicosin (*Mh*) resistance was only 1 and 2%. Spectinomycin and tetracycline resistance varied from 4 to 15%. For amoxicillin, only one *Mh* isolate showed MIC ≥64 mg/L while the highest MIC observed was 4 mg/L for cephalexin and 0.03 mg/L for cefquinome. MIC distributions of marbofloxacin and enrofloxacin were similar. For danofloxacin, 94% *Mh* and 99% *Pm* were susceptible. Tylosin and lincomycin showed similar MIC patterns with $MIC_{90}$ of 32 mg/L and ≥32 mg/L. A total of 135 *Pm*, 129 *Ap* and 110 *Ss* were recovered from swine. Resistance to ceftiofur, amoxicillin/clavulanic acid, tilmicosin and tiamulin was absent; resistance to enrofloxacin and florfenicol

was <1%. Whilst co-trimoxazole resistance varied from 3 to 7%, tetracycline resistance varied from 15% (*Ap*) to 82% (*Ss*). For antibiotics not having a breakpoint, similar MIC ranges as those obtained for cattle isolates were observed. **Conclusions:** Generally, the results show an absence or baseline antimicrobial resistance among the major respiratory tract pathogens isolated from diseased but non-treated cattle and swine across the EU.

**Board 42. Encouraging Trends in Invasive Infection with Methicillin-Resistant *Staphylococcus aureus* (MRSA) in Connecticut, 2001-2010**

**S. Petit**[1], H. Altier[1], C. Marquez[1], M. Cartter[1], J. Hadler[2];
[1]Connecticut Department of Public Health, Hartford, CT, USA, [2]Yale University, Emerging Infections Program, New Haven, CT, USA.

**Background:** Antibiotic resistant staphylococci have long been of public health concern. In 2001, Connecticut began statewide laboratory reporting of invasive MRSA. In 2007, national attention was called to it in a publication by the Emerging Infections Program on the magnitude of the invasive MRSA problem and passing of legislation in many states mandating reporting of hospital infections. We examined 10 year trends in MRSA incidence with emphasis on trends since 2007. **Methods:** Cases were defined as MRSA isolates from normally sterile body sites, then classified after medical record review as hospital onset (HO, isolate >2 days after admission), community onset, healthcare associated (HA, hospitalization, surgery, dialysis or long term care facility stay in the past year, or central line at diagnosis); or community associated (CA). Annual rates per 100,000 population were calculated. HO rates were also calculated by 100,000 patient bed-days in 3 hospital groupings by patient volume. Incidence trends were examined by chi square for trend overall (2001-10) and in 3 time periods, 2001-03, 2004-06 and 2007-10. **Results:** In 2001-10, 8758 cases of invasive MRSA were reported; 58% HA, 31% HO and 11% CA. From 2001-10, incidence decreased for overall, HA, and HO (26.0 per 100,000 population to 22.1, 14.4 to 13.8, and 10.0 to 5.2 respectively, all $p<0.01$). Incidence of CA increased (1.4 to 3.1, $p<0.01$). When broken into the 3 time periods, the only trends were decreases in overall, HO and HA from 2007-10 (all $p\leq0.04$) and an increase in CA from 2004-06 ($p<0.01$). From 2007-10, overall incidence dropped by 18.8%, HO by 33.2%, and HA by 12.8%. After the earlier increase, CA dropped 12.7% from 2007-10. There was a consistent downward trend in HO incidence by patient volume overall and for high and medium volume hospitals from 2001-10 ($p<0.01$ for each). Although low volume hospitals had an increase from 2001-06 ($p=0.04$), they had a decrease from 2007-10 ($p=0.01$). Over time, rates by patient volume converged, with a 3.9-fold higher incidence in high compared to low volume hospitals in 2001 reduced to 1.5-fold in 2010. **Conclusions:** Since 2007, HA, HO and CA MRSA have been decreasing, coincident with increased public, public health and hospital attention. Continued monitoring is needed to assess the sustainability of the apparent prevention gains.

**Board 43. Dissemination and Characterization of Class I and II Integrons in *Salmonella enterica* Isolated from Human Patients**

**M. Ghafari**[1], B. Bakhshi[2], M. Pourshafie[3], N. Amirmozafari[1], M. Salimi[3], B. Zarbakhsh[3], M. Rahbar[4], M. Hajia[4], M. Mohamadzadeh[5], F. Eghbalpour[3];
[1]Islamic Azad University, Science and Research Branch, Tehran, Iran, Islamic Republic of, [2]Tarbiat Modares University, The Faculty of Medical Sciences, Tehran, Iran, Islamic Republic of, [3]Pasteur Institute of Iran, Tehran, Iran, Islamic Republic of, [4]Center for Disease Control, Ministry of Health and Medical Education, Tehran, Iran, Islamic Republic of, [5]Department of Microbiology, Milad Hospital, Tehran, Iran, Islamic Republic of.

**Background:** *Salmonella enteric*a (S. enterica) is the major cause of 21 million annual cases of typhoid fever and 216,000 deaths in all over the world. The initial aim of this study were perusing dissemination of the genes responsible for antimicrobial resistance that has been largely attributed to mobile genetic

elements, including Class I and II integrons in *S. enterica* strains. We tried to specify the prevalence of class I and II integrons in *S. enterica* serogroups isolated from patients by PCR alongside determination of antibiotic susceptibility profile and attempted to explore the relationship between the presence of class I and II integrons genes and antibiotic susceptibility pattern. **Methods:** Eighty *S. enterica* strains isolated from patients with diarrhea who were admitted to the hospital. According to performed biochemical tests, bacteria isolates were classified in five following serogroups; A (2.5%), B (10.1%), C(25.3%), D(32.5%) and E(29.1%). Class I integron was detected by amplification of class-specific integrase gene using specific primers, and also their resistance genes content was identified by PCR-sequencing of their variable resistance region. According to our results, performing PCR to investigate the presence of class I and II integrons, Along with determination of the antibiotic susceptibility profile test, provides useful information needed for the ongoing surveillance of multiply resistant *S. enterica* and other bacterial pathogens involved in outbreaks. **Results:** 26.25% of the isolates (21 out of 80) were found to contain the Class I integron's conserved region and 95.2% of these isolates (20 out of 21) had the class I integron's gene variable region. None of the isolates contained class II integron gene. The data were compared with information obtained from the determination of antibiotic susceptibility profile test and amplified fragments sequencing. **Conclusions:** Performing PCR to reach a reliable serogrouping of salmonella isolates is an important approach in serodiagnosis of salmonellosis. Class I integron was extensively found among serogroup C, which emphasizes on its transferability among this group of *salmonella enterica* isolates. Class II integron was not detected among the isolates indicative of Salmonella strains incompatibility to this class of integrons.

**Board 44. Point-Prevalence Survey of Antibacterial Use at Select Hospitals in Egypt-2011**

**M.M. Talaat**[1], A. Kandeel[2], A. El-Kholy[3], T. Saeid[1], R. Galal[2], H. Bazara'a[3], S. Hafez[4], M. Yehia[5], G. Ismail[6], M. Abdel-Fattah[7], A. Osman[8], M. Abdel-Fattah[2], D. Calfee[9];
[1]US Naval Medical Research Unit, No. 3, Cairo, Egypt, [2]Ministry of Health and Population, Cairo, Egypt, [3]Faculty of Medicine, Cairo University, Cairo, Egypt, [4]Faculty of Medicine, Alexandria University, Alexandria, Egypt, [5]Faculty of Medicine, Azhar University, Cairo, Egypt, [6]Faculty of Medicine, Ain Shams University, Cairo, Egypt, [7]Minya University Hospitals, Minya, Egypt, [8]Faculty of Medicine, Minya University, Minya, Egypt, [9]Weill Cornell Medical College, New York, NY, USA.

**Background:** Inappropriate antibiotic (ABT) use leads to an unnecessary increase in the risk of antimicrobial resistance and other adverse outcomes. The prevalence of and practices related to antibiotic use in Egyptian hospitals have not been described. The objectives of this study were to determine the prevalence and characteristics of ABT use in Egypt to identify opportunities for improvement. **Methods:** A point prevalence survey was conducted in 18 hospitals between 14-27 March 2011. All patients on a hospital unit at 8AM on the designated day of survey were evaluated. Infection control teams collected data from patient's medical charts and interviews of treating physicians, if required. **Results:** During the survey, 3,408 patients (54% female) were on the 265 hospital wards. Fifty-nine percent of patients were receiving at least 1 antibiotic and 28% were receiving 2 or more. The most commonly prescribed antibiotics were 3$^{rd}$ generation cephalosporins (29%), beta-lactam/beta-lactamase inhibitors (20%), and nitroimidazoles (15%). A greater proportion of males than females was receiving antibiotics (62% vs 57%, p=0.001). Antibiotic use was more common among patients <12 years of age than those ≥12 (76% vs 56%, p<0.001) and among ICU patients (76%) than those on surgical and medical wards (62% and 51%, respectively, p<0.001). Reasons for antibiotic use included treatment of community- (27%) and healthcare-associated infections (11%) and surgical (39%) and medical (23%) prophylaxis (PPX). Common body sites for which antibiotics prescribed were: respiratory (19%), gastrointestinal (16%), gynecological tracts (16%), and skin, bone and joint (15%). Among 704 recipients of surgical PPX for which timing of the first dose was documented, 28% received the dose 0-120 minutes prior to incision. The remainder received the first dose >2 hours before (21%) or after incision (51%).

Among 326 patients with known duration of surgical PPX, 75% received >24 hours of PPX. **Conclusions:** The prevalence of antibiotic use in Egyptian hospitals was high (59%). Although additional assessment of ABT prescription practices in individual hospitals is needed, obvious targets for initial antimicrobial stewardship activities include provision of ABT prescription guidelines and optimization of the timing and duration of surgical PPX.

**Board 45. Antimicrobial Resistance in *Shigella* Isolates Along the US-Mexico Border: Interim Results**
**J.A. Smith**[1], A.E. Phippard[2], A.C. Kimura[3], P. Kriner[4], K. Lopez[4], G. Washabaugh[5];
[1]California Department of Public Health, San Diego, CA, USA, [2]Centers for Disease Control and Prevention, San Diego, CA, USA, [3]California Department of Public Health, Los Angeles, CA, USA, [4]Imperial County Public Health Department, El Centro, CA, USA, [5]San Diego County Public Health Laboratory, San Diego, CA, USA.
**Background:** In the US, the highest incidence rates for shigellosis occur in regions bordering Mexico, regions which some studies show may also have higher levels of antimicrobial resistance (AMR). A national survey found that AMR *Shigella* was predicted by travel to Mexico. Selective pressures unique to the US-Mexico border region may contribute to the development and spread of AMR *Shigella*. **Methods:** All non-outbreak associated *Shigella* cases with onset on or after October 1, 2008 in San Diego County (SDC) and Imperial County (IC) were asked to participate in a survey. Available *Shigella* isolates were submitted to the SDC Public Health Laboratory for antibiotic susceptibility testing (AST). **Results:** As of March 31, 2011, 382 sporadic *Shigella* cases were reported to SDC and IC. The median age was 25 years; 66% were Hispanic and 15% were born outside of the United States, mainly in Mexico. Of subjects with available information, 40% had recent international travel, predominantly to Mexico. Seventy-eight percent of SDC and IC *Shigella* isolates were resistant to at least one antibiotic; 73% to Trimethoprim-sulfamethoxazole (SXT), 21% to Ampicillin (AMP), 0.6% to Ceftriaxone and 0.6% to Ciprofloxacin. The large majority of resistance to AMP was seen in SDC, with the exception of 1 isolate in a case-patient from IC. A higher proportion of foreign-born case-patients had resistance to at least one antibiotic (92%) compared to US-born case patients (76%). International travelers do not appear to have a higher proportion of antibiotic resistance compared to non-travelers in our study thus far. **Conclusions:** Interim data suggest that patterns of resistance occurring along the California-Baja California border may vary by county of residence and country of birth; resistance patterns in Imperial County may be more similar to those reported in Mexico than to those in the US. In addition, our data suggests that being born outside of the United States may play a larger role in predicting antibiotic resistance than international travel. Additional studies and further analysis is required to fully understand the interplay between country of birth, county of residence, international travel and antibiotic resistance in the California/Baja California border region.

## Tropical Infections and Parasitic Diseases

Monday, March 12
12:30 PM – 1:30 PM
Grand Hall

**Board 46. Impact of Insecticide-Treated Bednets on Malaria Transmission Indices on the South Coast of Kenya**
**C.H. King**[1], F. Mutuku[2], P.L. Mungai[1], C. Mbogo[3], J. Mwangangi[3], E.M. Muchiri[4], E.D. Walker[5], U. Kitron[2];
[1]Case Western Reserve University, Cleveland, OH, USA, [2]Emory University, Atlanta, GA, USA, [3]Kenya

Medical Research Institute, Kilifi, Kenya, [4]Ministry of Public Health and Sanitation, Nairobi, Kenya, [5]Michigan State University, East Lansing, MI, USA.
**Background:** Besides reducing malaria vector densities, prolonged bednet usage is linked i) to behavioral shifts to exophagy (outdoor biting) by two important malaria vectors in Africa, *An. gambiae* s.l. and *An. funestus,* ii) to a decline of *An. gambiae* s.s. relative to *An. arabiensis,* and iii) to changes in host feeding preference. In southern coastal Kenya, bednet use was negligible in 1997-1998 when *Anopheles funestus* and *An. gambiae* s.s. were the primary malaria vectors, with *An. arabiensis* and *An. merus* playing a secondary role. Since 2001, bednet use has increased progressively, reaching substantially high levels with corresponding declines in malaria transmission. **Methods:** To evaluate the interval impact of increased household bednet use within this area on vector composition and human-vector contact, we collected indoor resting mosquitoes during 2009-10, using pyrethrum spray catches. Current bednet coverage and usage were determined in the same houses. Species distribution was determined, and number of mosquitoes per house, human-biting rates (HBR), and entomological inoculation rate (EIR) were compared to those reported for the same area during 1997-98 when bednet coverage was minimal. **Results:** In 2009-2010, the predominant malaria vectors in the study area were *An. funestus* and *An. gambiae* s.l., both of which are highly anthropophilic. However, as compared to 1997-1998, we noted a significant decline in the relative proportion of *An. gambiae* s.s. among collected mosquitoes coupled with a proportionate increase of *An. arabiensis*. Following > 5 years of 60-86% coverage, the density, human biting rate and EIR of indoor resting mosquitoes were reduced by more than 92% for *An. funestus* and by 75% for *An. gambiae* s.l. In addition, the host feeding choice shifted more toward non-human vertebrates. HBR was higher in houses without nets compared to houses with nets. **Conclusions:** These data indicate a much reduced human biting rate and a diminishing role of *An. gambiae* s.s. in malaria transmission following higher bednet coverage. While increasing bednet coverage beyond the current levels may not significantly reduce the transmission potential of *An. arabiensis*, it is anticipated that increasing or at least sustaining high bed net coverage will result in a diminished role for *An. funestus* in malaria transmission.

### Board 47. An Outbreak of Dengue Fever in Houston, Texas, with Evidence of Autochthonous Transmission between 2003 and 2005

**K.O. Murray**[1], L. Rodriguez[1], E. Herrington[1], V. Kharat[1], N. Vasilakis[2], C. Walker[1], C. Turner[3], S. Khuwaja[3], R. Arafat[3], S. Weaver[2], D. Martinez[4], C. Kilborn[4], R. Bueno[4], M. Reyna[4], T. Sharp[5], J. Munoz[5];
[1]The University of Texas Health Science Center at Houston, Houston, TX, USA, [2]The University of Texas Medical Branch, Galveston, TX, USA, [3]City of Houston Department of Health and Human Services, Houston, TX, USA, [4]Harris County Public Health and Environmental Services, Houston, TX, USA, [5]Centers for Disease Control and Prevention, San Juan, PR, USA.
**Background**: Houston, Texas maintains an appropriate climate and mosquito populations to support the circulation of dengue virus (DENV). **Methods:** We tested 3,768 cerebral spinal fluid (CSF) and serum specimens from Houston patients hospitalized between 2003 and 2005 with suspected arboviral disease for the presence of anti-DENV IgM antibodies. PCR and plaque reduction neutralization test was used to further confirm dengue infection. **Results:** We identified 47 specimens with detectable anti-DENV IgM antibody; two also had DENV-2 detected by RT-PCR. The epidemic curve supports the presence of an outbreak in 2003. Chart abstractions were completed for 42 cases; 57% were diagnosed with meningitis and/or encephalitis and 43% met the case definition for dengue fever. Two cases were fatal, including one with illness compatible with dengue shock syndrome. Most cases (84%) did not have a history of travel to a dengue endemic area. **Conclusions/Significance:** Our results support local transmission of DENV during the study period. These findings heighten the need for dengue surveillance in the southern Unites States.

**Board 48. Microsatellite DNA Loci from a Genome-wide Database Show Significant Variance in Genetic Diversity among Populations of *Schistosoma haematobium***

**T.C. Glenn**[1], S.L. Lance[2], A.M. McKee[1], B.L. Webster[3], A.M. Emery[3], A. Zerlotini[4], G. Oliveira[4], D. Rollinson[3], B.C. Faircloth[5];
[1]UGA, Athens, GA, USA, [2]UGA, SREL, Aiken, SC, USA, [3]Natural History Museum, London, UK, [4]Oswaldo Cruz Foundation, Barro Preto, Brazil, [5]UCLA, Los Angeles, CA, USA.

**Background:** Urinary schistosomiasis caused by *Schistosoma haematobium* is widely distributed across Africa and is increasingly being targeted for control. Genome sequences and population genetic parameters can give insight into the potential for population- or species-level drug resistance. Genome sequences and genetic diversity estimates are available from *Schistosoma mansoni* and *S. japonicum*, the two other species of most significant human health relevance, but such genetic data are lacking for *S. haematobium*. **Methods:** We conducted 454 titanium sequencing of random DNA fragments from cercariae collected from a snail infected with a single *S. haematobium* miracidium. We assembled the resulting 1,058,114 genomic DNA sequences into contigs and aligned the contigs to the genome sequences of *S. mansoni* and *S. japonicum*. We identified microsatellite DNA loci across all three species and designed a total of 13,790 primer pairs to amplify unique microsatellite loci in *S. haematobium* (available at http://www.cebio.org/projetos/schistosoma-haematobium-genome). To validate the primers we designed, we screened 48 randomly selected primer pairs and identified 15 that amplified consistently and were easily scored. We genotyped these 15 loci in individual worms from six widely separated locations across Africa. **Results:** The *S. haematobium* genome contained about the same overall proportion of microsatellite DNA loci as *S. mansoni* and *S. japonicum*, but the frequency and length distributions of specific motifs differed among species. For the loci genotyped, a population of *S. haematobium* from Zanzibar had the highest levels of diversity, four other populations were nearly as diverse, but the population of *S. haematobium* from South Africa had much lower levels of genetic diversity. **Conclusions:** About 30% of the primers in the database of *S. haematobium* microsatellite DNA loci should yield amplifiable and easily scored polymorphic markers. Sequence conservation among the three species investigated here is relatively high. Thus, by comparing alignments of all three species, it should be possible to generate microsatellite markers that will work across most species of *Schistosoma*. Full genome-sequencing of several individuals of these species would be desirable for further work in diagnostics and drug resistance.

**Board 49. A Tale of Anthropogenic Disturbance: Forest Degradation and Interspecies Proximity in Relation to Parasite Communities in Howler Monkeys (Alouatta palliata) and Humans**

**W.D. Helenbrook**, W.M. Shields, C.M. Whipps;
State University of New York College of Environmental Science and Forestry, Syracuse, NY, USA.

**Background:** Nearly forty percent of pathogens responsible for tropical infectious diseases in humans originate in primates. The mechanism by which complex interactions between the environment, hosts, and parasites lead to disease emergence is still not fully understood despite a growing body of research. Indicators of these three variables were evaluated in mantled howler monkeys (*Alouatta palliata*) and people living near a forest reserve in northwestern Ecuador, in order to assess their potential role in disease emergence. This study aimed to: 1) chronicle primate parasitism, 2) investigate associations of environmental degradation and parasitism, and 3) assess human attributes and actions associated with parasitism and potential transmission between human and howler monkey populations. **Methods:** Human and monkey endoparasite communities were characterized using fecal flotations and sedimentations, as well as PCR based analysis. Humans from surrounding communities were administered demographic surveys to evaluate risk factors associated with parasitism. **Results:** Of one hundred and one howler monkey fecal samples collected, 8 species of protozoa, 8 nematodes, 8 platyhelminth, and 1 apicomplexan were detected. Notable genera include *Ascaris, Trichuris,*

*Schistosoma, Taenia, Diphyllobothrium, Toxoplasma,* and *Entamoeba*. In 50 human fecal samples, *Ascaris, Strongyloides, Trichuris, Cystiosospora, Schistostoma,* and *Entamoeba* were observed. In older growth forests, parasite diversity in primates appears to be greater than in logged areas. Considering human proximity, greater parasite diversity was observed in howler monkeys further away from people. Key human demographic data potentially influencing parasitism were an individual's proximity to monkeys and to forested areas, age, and use of treated water. **Conclusions:** Results indicate that forest degradation and human proximity to primate populations have an influence on parasite prevalence, abundance, and species diversity in howler monkeys. Decrease in howler monkey parasite diversity in disturbed forests is indicative of broken ecological links. Nonetheless, some of the same parasites observed in howler monkeys were also found in people. Ongoing data analyses examine the association of human parasitism and demographic factors.

## H1N1 Influenza

Monday, March 12
12:30 PM – 1:30 PM
Grand Hall

**Board 50. Preventive Effects of Ganyou Oral Spray and Traditional Chinese Medicine Daguo Decotion on Influenza in a Middle School in Chengdu,Sichuan, China 2009**

**F. Dou**[1], X. liang[1], Z. Yin[1], C. Du[1], C. Wang[1], Z. Liu[1], X. Deng[1], J. He[1], H. Zha[2], L. Ma[1];
[1]Chengdu Center for Disease Control and Prevention, Chengdu, China, [2]Chenghua Center for Disease Control and Prevention, Chengdu, China.

**Objective:** To evaluate the effects of influenza prevention drugs Ganyou Oral Spray and traditional Chinese medicine Daguo Decotion on influenza-like cases, influenza-like symptoms and upper respiratory tract infection(URTI) or common cold, and to explore the risk factors of these infections in order to provide scientific preventive measures for influenza. **Methods:** All students in a middle school in Chengdu City from September 2009 to January 15th, 2010 were enrolled in this study. According to the occurrence of fever and upper respiratory symptoms or not during this period, they were divided into influenza-like case group($n$=238), influenza-like symptoms group ($n$=310) and common cold or URTI group ($n$=500) while those without fever and upper respiratory tract symptoms were selected as the control group ($n$=276). The utilization of Ganyou Oral Spray, traditional Chinese medicine Daguo Decotion were investigated by questionnaire. Meanwhile, some of their health behaviors were surveyed by asking whether they wash hands before eating, often touch lips with hands and pick nostrils. **Results:** Multifactorial logistic analysis showed that using Ganyou Oral Spray before fever was a protective factor for influenza-like cases, cases of influenza-like symptoms and cases of common cold or URTI (Odds Ratio, OR =0.518,0.564 and 0.731, respectively) while traditional Chinese medicine Daguo Decotion was a protective factor only for the former two kinds of cases (OR=0.450 and 0.396, respectively). Age (OR = 0.835) were protective factors for the cases of influenza-like symptoms. However, often touching lips with hands was a risk factor for the three kinds of cases(OR=2.429,2.305 and 1.955, respectively).The influenza prevention rate of Ganyou Oral Spray in the three kinds of cases was 48.2%, 43.6% and 26.9%, respectively. That of traditional Chinese medicine Daguo Decotion in influenza-like cases and cases of influenza-like symptoms was 55.0% and 60.4%, respectively. **Conclusions:** Ganyou Oral Spray has certain preventive effect on influenza-like case, influenza-like symptoms and common cold or URTI. Taking traditional Chinese medicine Daguo Decotion can also prevent influenza-like cases, influenza-like symptoms to some extent. Often touching lips with hands is a risk factor for influenza infection.

**Board 51. Epidemiology of Influenza in Zambia post Influenza A, H1N1 Pandemic, 2009**

**M.L. Liwewe**[1], A. Theo[2], P. Simusika[2], I. Ndumba[1], E. Chentulo[2], H. Nkamba[2], M. Kapina[3], C. Malama[4], P. Songolo[5], M. Monze[2];
[1]World Health Organistion /Ministry of Health, Lusaka, Zambia, [2]University Teaching Hospital - Virology Laboratory, Lusaka, Zambia, [3]Ministry of Health, Lusaka, Zambia, [4]Centres for Diseases Surveillance & Prevention - Zambia, Lusaka, Zambia, [5]World Health Organistion, Lusaka, Zambia.

**Background:** On 10 August 2010, WHO Director-General Dr Margaret Chan announced that the H1N1 influenza virus has moved into the post-pandemic period, citing that localized outbreaks of various magnitudes are likely to continue. In Zambia as is seen globally, the levels and patterns of H1N1 transmission in the declared post pandemic period differ significantly from what was observed during the pandemic. Out-of-season outbreaks are no longer being reported in Zambia. In fact, since the declaration of a post pandemic period was announced, Zambia has recorded only 2 cases of Influenza A, H1N1 pandemic 2009. **Methodology:** Zambian public health authorities has in place a surveillance system for detecting suspected Influenza cases including Influenza A,H1N1 Pandemic 2009 and seasonal flu. Samples collected from suspected cases are analyzed primarily by Real time Polymerase Chain Reaction (RT-PCR). **Results:** 464 suspected flu cases were investigated under the Influenza Sentinel surveillance program from August 2010 to September 2011. Out of the 87 positive cases, 2 (2.3%) were Influenza A, H1N1 pandemic, 2 (2.3%) A, H3N2, 8 (9.2%) Influenza A not Sub typed and 69 (79.3%) Influenza B. In 2009, out of the 112 laboratory confirmed Influenza cases the following were the subtype frequencies: 86 cases (68%) AH1N1pandemic; 22 cases (20%) AH1N1 seasonal; 1 (2%) AH3N2; 9 (7%) A Not Sub typed and 4 (3%) FluB. In 2010, out of the 64 laboratory confirmed Influenza cases the following were the subtype frequencies: 10 cases (16%) AH1N1pandemic; 1 case (1%) AH1N1 seasonal; 12 (19%) AH3N2; 9 (14%) A Not Sub typed and 32 (50%) FluB. In 2011 as at 30th September, out of the 64 laboratory confirmed Influenza cases the following were the subtype frequencies: 2 cases (2.7%) AH1N1pandemic; 0 cases (0%) AH1N1 seasonal; 2 (2.7%) AH3N2; 8 (10.9%) A Not Sub typed and 61 (83.6%) FluB. **Discussion and Conclusions:** Zambia in the post pandemic period has seen a shift in the pattern of influenza infections presenting from mostly Influenza type A in 2009 to Influenza type B in 2010 and 2011. As anticipated by the teams in WHO reviewing the status of Influenza A, H1N1 pandemic worldwide, the pandemic is of the past in Zambia with only 2 cases having been picked through the surveillance system.

**Board 52. Post Pandemic Testing for Seasonal and Pandemic Influenza Viruses at a Tertiary Care Hospital in Vellore, South India**

**A.M. Abraham**[1], M. Moorthy[1], S.K. Reddy[1], S. Kumar[1], S. Vijayakumar[1], D. Sekhar[1], R. Lal[2], A.C. Mishra[3];
[1]Christian Medical College, Vellore, Vellore, India, [2]CDC India Influenza Program, New Delhi, India, [3]National Institute of Virology, Pune, India.

**Background:** The end of the H1N1 2009 pandemic was declared on August 10, 2010. We determined the trends in the detection of influenza viruses among patients with influenza like illness (ILI) and severe acute respiratory illness (SARI) during the post-pandemic period. **Methods:** Patients were enrolled from pediatric, adult medicine and emergency outpatient clinics and all inpatient wards of Christian Medical College from June 1, 2010 to August 31, 2011. ILI was defined as presence of fever (>100°F), cough, sore throat and rhinorrhea in the absence of any other diagnosis, and SARI was defined as presence of ILI symptoms and breathlessness requiring hospitalization and who were moderately to severely ill on a clinical scale developed in the hospital. Throat and nasal swabs were collected and tested for influenza A including pandemic H1N1 and influenza B viruses by real time RT-PCR following the CDC protocol. **Results:** Of 1,541 ILI cases, 426 (28%) were positive for influenza A and 107 (7 %) for influenza B. Of the influenza A positive, 78 (18%) were seasonal (H3N2) influenza viruses and 348 (82%) were pandemic H1N1 2009 (pH1N1). Of 1345 SARI cases, 165 (12%) were positive for influenza A and 38 (3%) for

influenza B. Of the influenza A positive, 26 (16%) were seasonal (H3N2) influenza viruses and 139 (84%) were pH1N1. Among both ILI and SARI cases, pH1N1 virus was predominantly detected during July-October, 2010 while seasonal H3N2 viruses were detected during July-December, 2010 and reappeared in July, 2011. Influenza B viruses were detected during July, 2010-April, 2011 at low levels. All 3 viruses circulated during July-October, 2010; 2 co-infections (pH1N1 and influenza B) each were detected among ILI and SARI cases. **Conclusions:** Pandemic H1N1 virus infection was predominantly detected from our hospital in Vellore during the post-pandemic period until October 2010. Seasonal H3N2 that co-circulated with the pandemic strain in 2010 re-emerged in July 2011. Both seasonal influenza A and pH1N1 viruses were associated with ILI and SARI during the post-pandemic period. *(Co-funded by the Indian Council for Medical Research and Centre for Disease Control, Atlanta, USA Co-operative agreement 5U51IP 000333).*

***Board* 53. The Influenza A Virus Nucleocapsid Protein Sequesters Tumour Suppressor LIMD1 to Enhance Viral Gene Expression.**

**S.K. Lal**[1], A.K. Mayank[1], S. Sharma[1], J.M. Katz[2], N.J. Cox[2], R.B. Lal[2], S. Sambhara[2];
[1]International Centre for Genetic Engg. & Biotechnology, New Delhi, India, [2]Centers for Disease Control and Prevention, Atlanta, GA, USA.

**Background:** The influenza virus nucleoprotein (NP) is an indispensable player engaged in multiple stages of the virus life-cycle, most critical among which include replication, transcription, assembly of genome and budding. **Methods:** In order to discover host proteins that interact with NP, the most conserved viral protein, we screened a human lung cDNA library using the yeast two-hybrid system with NP as bait. LIMD1 was identified as one of the host cell protein interacting with viral NP. The full-length human cDNA LIMD1 tested positive both for His - prototrophy and β-galactosidase activity. The interaction was further validated by co-immunoprecipitation in vitro and in transiently-transfected mammalian cells. **Results:** LIM-domain containing proteins participate in intracellular signaling, transcriptional regulation and cellular differentiation during development. Interestingly, LIMD1 has been reported to be a tumor suppressor gene, regulating cell-cycle (transition from G1 to S phase) through its interaction with retinoblastoma (Rb) tumor suppressor. Furthermore, it is known to be predominantly localized in the cytoplasm. Intriguingly, our immunofluorescence studies indicate that NP co-localizes with LIMD1 specifically in the nucleus, suggesting that NP causes translocation of LIMD1 from cytoplasm to nucleus. Additionally, RNAi-mediated knockdown of LIMD1 expression leads to increased levels of viral NP mRNA as well as viral titers in virus-infected A549 cells. Furthermore, LIMD1 silencing in virus infected cells led to elevated of levels of phosphorylated Rb, suggesting a pro-proliferative effect post-infection. **Conclusions:** Therefore we hypothesize that NP-LIMD1 interaction plays a vital role during viral replication and transcription.

**Board 54. Incidence Rate of Symptomatic Pandemic 2009A/H1N1 and Seasonal Influenza Infection in a Rural Indian community**

**K. Fowler**[1], S. Broor[2], W. Sullender[1], V. Gupta[3], M.-A. Widdowson[4], R.B. Lal[4], A. Krishnan[2];
[1]University of Alabama, Birmingham,, Birmingham, AL, USA, [2]All India Institute of Medical Sciences, New Delhi, India, [3]The InCLEN Trust, New Delhi, India, [4]Centers for Disease Control and Prevention, Atlanta, GA, USA.

**Background:** Since November 2009, three villages in Ballabgarh (located north India) have been under prospective weekly active surveillance for febrile acute respiratory illness (FARI) as part of a trial of seasonal inactivated trivalent influenza vaccine (TIV). This provided a unique opportunity to study the spread of 2009A/H1N1 virus in this rural community. **Methods:** Possible influenza cases in the population under surveillance(n=18220) were identified during weekly household visits by field workers using a FARI case definition. Cases were clinically assessed by trained nurses and had throat and/or nasal

swabs collected for influenza testing by real-time (RT)-PCR. We calculated the incidence of the 2009A/H1N1 and seasonal influenza viruses from Nov 2009 to October 2010. **Results:** Of the 711,731 person-weeks under surveillance from Nov 2009-Oct 2010, 9,395 FARI were reported (incidence rate 686/1000 p-yrs). Of the 7571 FARI cases tested, 1410 (18.6%) were positive for influenza. Of these, 749 (53%) were with pandemic 2009A/H1N1, and 661 (8.7%) were with seasonal influenza (643 influenza B and 18 with H3N2). The population incidence rate for 2009A/H1N1 infection was almost 7 times higher (179/1000 p-yr) than that for seasonal influenza virus (26/1000 p-yr) during the pandemic year. The peak 2009A/H1N1 incidence observed was 713/1000 p-yr in week 50 in 2009, whereas seasonal influenza was only 39/1000 p-yr in the same week. The second peak of influenza positivity was observed in August to October 2010, with incident rates of 156 and 122/1000 p-yr, respectively for pandemic 2009A/H1N1 and seasonal influenza. The incidence rate of 2009A/H1N1 virus decreased to <3/1000 p-yr during Feb-July 2010, whereas the incidence rate of seasonal influenza viruses remained at 28-40/1000 p-yr during these months. The highest rates of pandemic 2009A/H1N1 were observed in children between the ages of 0-18 yrs. **Conclusions:** The incidence of 2009A/H1N1 virus infections was noted to be up to 20 times higher than incidence of seasonal influenza in certain weeks and thus had the potential to severely burden the health care delivery system. These studies were supported in part by cooperative agreements U01 IP000177 from the Centers for Disease Control and Prevention, Atlanta, USA

## Risk Assessment

Monday, March 12
12:30 PM – 1:30 PM
Grand Hall

**Board 55. Rapid Risk Assessment of A Novel *Bunyavirus* Human Infection in China**

**W. Xiong**[1], Z. Feng[1], A.R. Foxwell[2];
[1]Chinese Center for Disease Control and Prevention, Beijing, China, [2]World Health Organization Regional Office for the Western Pacific, Manila, Philippines.

**Background:** Severe fever with thrombocytopenia syndrome (SFTS) is a tick-borne hemorrhagic fever-like disease first reported in rural area of Central China between late spring and early summer in 2009. At that time, an unusually high case fatality rate (CFR) of 30% was reported, which aroused widely social concerns. In June 2009, a novel phlebovirus in bunyavirus family was isolated from a patient's blood sample, which was named SFTS virus (SFTSV). According to the published data, active surveillance in 6 provinces of Central and Northeast China found that among 241 hospitalized patients who met the definition for SFTS, SFTSV was found in 171 of them. Because SFTS is a newly discovered disease caused by a novel virus with high initial CFR, some concerns about potential spread and more death if there's no effective intervention are predictable and that is the reason of this assessment. **Methods:** 1. Literature review and data analysis. Using Google Scholar, literatures about hazard, exposure, and vulnerability factors of SFTSV infection are collected and reviewed. Key words (both in English and in Chinese) used include severe fever with thrombocytopenia syndrome, tick-borne disease, SFTS, SFTSV, phlebovirus, bunyavirus, tick, *H. longicornis,* healthcare seeking, social panic, etc. Demographic data of population in 6 surveillance provinces are gathered from China statistical yearbook 2010. Chi-square test is used to compare the age and gender differences between patient and original population. 2. Create risk assessment matrix. Based on the results of literature review and data analysis, a risk assessment matrix is created to assess the likelihood of spread and the magnitude of public health impact. The matrix consists of 3 parts: threat of the agent (hazard), exposure to agent, and vulnerability. In each part,

related information are sorted into 3 groups: negative factor (means can accelerate spread or magnify public health impact), positive factor (means can hold spread or reduce public health impact), and important information gap. **Results:** 8 published documents are included for analysis, which include scientific papers and governmental guidelines. For the hazard, negative factors include: SFTSV is a new recognized member of phlebovirus genus in bunyavirus family, which included many public health important virus like Rift valley fever virus, Toscana virus and phleboviruses; human infection may lead to multiorgan dysfunction in severe cases; and a high CFR in hospitalized cases was observed in the early stage. Positive factor is no evidence of human to human transmission was found. For the exposure, negative factors include: the vector is widely spread in mainland China; lots of common animals may act as reservoir; and some sub-population like farmer, female and elder seem to be at high risk. Positive factor is the virus was only isolated from a specific tick species. For the vulnerability, negative factors include: human are generally susceptive; resident lived in rural area usually have passive healthcare seeking behavior; and mass media report may lead to panic in public and overloading in healthcare system. Positive factors include: most of the local hospitals have the ability of clinical diagnosis; most of the provincial CDCs have the ability of lab confirmation; and public can get education information from multisource. Important information gaps exist, such as transmission mode, communicability, spectrum of infection, seroprevelance and human immunity. **Conclusions:** The public health risk related to exposure to SFTSV in China is low to moderate: the likelihood of spread is likely, the magnitude of impact on public health is minor. There is a potential increase in the number of cases reported from other provinces when the surveillance is expanded and a greater need for diagnosis and healthcare service during the peak season. Giving above mentioned key information gaps, the level of confidence of this risk statement is moderate.

**Board 56. A Systematic Review of Acute Encephalitis Syndrome in India**

**S.S. Hossain**[1], K.C. Earhart[1], L.S. Chauhan[2];
[1]CDC, New Delhi, India, [2]National CDC, India, New Delhi, India.

**Background:** Acute Encephalitis Syndrome (AES) has been reported in India since at least 1952. Fifteen states have reported AES outbreaks in recent years. Uttar Pradesh, the largest Indian Sstate, has been most affected with 34 districts reporting AES cases in recent years. The etiology of a recent upsurge of cases has not been well defined. Reporting of cases has only recently begun through separate surveillance systems, impeding calculation of burden of disease. The national health program responsible for disease control collates information on cases and deaths and posts it on its website. Similarly the Integrated Disease Control Project (IDSP) of the Central Government reports data on cases and outbreaks of AES. Research on AES is conducted by various local, regional and national institutions in India and the findings are published in scientific journals. **Methods:** We reviewed existing literature and available data on AES in India. A key word search for observational studies of clinical and epidemiological patterns, outbreak, diagnostic and etiologic studies, and mosquito bionomics and prevention strategies identified 188 articles, mostly published in peer-reviewed journals in India. **Results:** In the publications reviewed, Japanese Encephalitis virus, Coxsackie B virus, Enterovirus 76 and 75, Chandipura virus, Measles and West Nile Virus were most frequently implicated, in different regions of the country. Data compiled from the weekly reports of IDSP include 10,297 cases of AES throughout India in 2010. For 2011, the national program reported 4,458 cases of AES with 584 deaths as of September 13th. In addition, weekly outbreak reports document 22 AES outbreaks during the preceding 29 months. **Conclusions:** While AES causes a heavy toll in India, data on the etiology, burden, distribution and risk factors are known in piecemeal fashion only. Existing case-counts likely may represent a substantial underestimate. Clear understanding of the etiologic-specific burden of disease and associated risk factors are is required for prioritizing, defining, implementing and evaluating appropriate control measures. Given the complexity of AES, developing effective laboratory-based

surveillance and outbreak response capacity may best be addressed with a pilot or "proof-of-concept" approach.

**Board 57. Statistical Estimation of Association between Reported Exposure and Sporadic *Salmonella* serotype Enteritidis Illnesses Based on Logistic and Lasso Model**

**W. Gu**, R.M. Hoekstra, A.R. Vieira, D.J. Cole;
Centers for Disease Control and Prevention, Atlanta, GA, USA.

**Background:** Most *Salmonella* serotype Enteritidis (SE) infections in the US are sporadic (not associated with recognized outbreaks). Ascertaining exposure sources of sporadic SE infections provides important information for evaluation of food intervention policies. Case-control studies are conducted to investigate associations between sporadic illness and a broad range of exposures. In these situations, statistical analysis by logistic regression might be hampered by correlated variables in large data sets. Evaluating new ways to analyze such studies may help to advance causal inference, especially in studies of foodborne illnesses that have several transmission pathways. **Methods:** We re-analyzed data on exposures in persons >12 years old collected by a case-control study of SE conducted by the Foodborne Diseases Active Surveillance Network (FoodNet) in 2002. We compare two model-building strategies: conventional two-step logistic regression and lasso modeling. For logistic regression, bivariate analysis was used to select a candidate set of variables for subsequent stepwise selection. We compared two scenarios of model building by varying the threshold p-value (0.05 vs. 0.1) for variable selection. For lasso modeling, cross validation was applied to determine the cut-value of λ which minimized the deviance of model prediction on the validate parts. Population attributable fractions (PAF) were calculated for all models. **Results:** Four variables significantly associated with increased risk of SE were identified by both logistic and lasso models: international travel, consumption of chicken cooked outside the home, consumption of uncooked ground beef, and consumption of steak. An additional 14 variables were significant in either logistic or lasso models, but not both. Estimated PAF of logistic models tended to be larger than corresponding lasso estimation (e.g. PAF of chicken consumption=38.7% and 12.8% from logistic and lasso, respectively). **Conclusions:** Our study suggests that determination of significant risk factors of illness from case-control studies is highly sensitive to the analytic tools and models used. Combined use of logistic regression and lasso models might be complementary for identifying and estimating risks using case-control study data.

**Board 58. Assessment of Risk Perception and Behaviours towards Avian Influenza (AI) in Ibagwa-aka: a Rural Community in Enugu State, Nigeria, 2010**

**O.P. Ossai**[1], P. Nguku[1], C.O. Ekwueme[2], D. Nwagbo[2], M. Otiji[3], C.P. Igweagu[3], N.E. Ossai[3], C.C. Nwodo[4], H.O. Aneke[3], C.H. Onah[3], J.I. Ajogwu[3], T. Dahiru[5];
[1]Nigerian FELTP, Abuja., Nigeria, [2]University of Nigeria Teaching Hospital., Enugu., Nigeria, [3]Ministry of Health., Enugu., Nigeria, [4]ESUT Teaching Hospital., Enugu., Nigeria, [5]Ahmadu Bello University Hospital., Zaria., Nigeria.

**Background:** In 2006, Nigeria was aroused by an outbreak of avian influenza in poultry that affected 26 out of its 36 states. A human case was confirmed in 2007 and subsequently, several suspected but unconfirmed cases were reported. The government of Nigeria put in place some control measures including public enlightenment and risk communication which were effective. However, soon after the outbreaks subsided, these measures were relaxed ( last outbreak was in July 2008). The recent warning by WHO that the world is still at the brink of avian influenza outbreaks makes it imperative that countries should take proactive measures to avert another major pandemic. In rural communities in Nigeria, culturally determined behaviours such as sharing bed space with birds, eating sick/ dead birds and raw eggs, using bird droppings as manure which can increase the risk of transmission of AI are known to be common practices. We assessed the current behaviours and risk perception towards AI

among rural inhabitants. **Methods:** This is cross-sectional descriptive study in which 400 respondents were selected through a multistage sampling method. A pretested standardized questionnaire was used to collect data on awareness, risky behaviours and risk perception. Data was analyzed using Microsoft excel 3.0. **Results:** About 345 persons(86.3%) responded to the interview. Majority of the respondents were females(60.3%).Awareness regarding AI was high (86.4%). Mass media provided the greatest source of awareness(78.3%). About 78% still use bird droppings as manure, 38.3% eat dead/ sick birds, 36.2% share bed space with birds and 50.7% eat raw eggs. Only about 22.6% of those who indulge in these practices feel they are at the risk of AI. **Conclusions:** The low risk perception and continued indulgence in risky behaviours necessitates the need for sustained public health messages on risk/behavioural change communication in our rural communities.

**Board 59. Microbial Risk Assessment by Extrapolating Dose-response Curves: Lessons from Anthrax**

**T.H. Whalen**;
Frontline Healthcare Workers Safety Foundation, Atlanta, GA, USA.

Much microbial risk assessment extrapolates mathematical dose-response curves fit to high dose animal studies to estimate risk to humans from very low doses; for example 2% calculated infection rate from exposure to nine anthrax spores. Historical accounts of actual human exposures do not support predicting disease from such low doses. The 5 cases of inhalation anthrax in the 1957 Manchester outbreak occurred during processing of an unusually dirty and dusty batch of goat hair. Air monitoring after the outbreak had ended found hundreds of spores in the air at a time when the raw materials were more normal and no new cases occurred. Brachman (1966) exposed macaques to anthrax-contaminated air from a working mill for 47 days. Calculations based on these data strongly suggest that over 600 spore-containing particles were inhaled by humans working in the mill on 17 (36%) of the 47 days. This implies approximately 120 high-dose days per worker per year for hundreds of workers over the 60 years prior to widespread vaccination of mill workers, yet fewer than ten cases of inhalational anthrax were recorded. Millworker family members likely received more than nine secondhand spores per day; by Meselson's calculations 2% per day should have contracted anthrax, but no inhalation cases were reported among mill workers families in the US despite millions of person-days of exposure. The primary path of infection for inhalation anthrax can vary with the dose, including opportunistic infection at low doses via existing lung lesions or other predisposing, phagocytic cell-mediated infection at higher doses, and rapid fulminant septicemia at extremely high doses. In any disease with different infection paths at different doses, the low dose risk to humans may be much higher or lower than implied by a probit or other mathematical curve fit to high doses. New tools are needed to integrate laboratory and field data in a multi-model approach especially for emerging disease.

## Laboratory Support

Monday, March 12
12:30 PM – 1:30 PM
Grand Hall

**Board 60. Arboviral Infections in Hospitalized Acute Febrile Illness Patients in Ghana, 2009-2010**

**M.E. Morales-Betoulle**[1], K. Kronmann[2], W.K. Ampofo[3], M.A. ElBadry[1], N. Puplampu[2], M. Clemens[2], J. Valle[4], A. Jones[4], K. Koram[3], E. Nyarko[5], G. Amofah[6], E. Dueger[4];
[1]NAMRU-3, Cairo, Egypt, [2]NAMRU-3, Ghana detachment, Accra, Ghana, [3]Noguchi Memorial Institute for Medical Research, Accra, Ghana, [4]CDC, NAMRU-3, Cairo, Egypt, [5]37 Military Hospital, Accra, Ghana, [6]Ghana Health Service, Accra, Ghana.

**Background:** Vector-borne infections such as malaria, dengue (DENV), chikungunya (CHIKV), and Q-fever, account for a large proportion of the infectious disease burden worldwide. In Ghana, acute febrile illness (AFI) is a significant concern with an estimated 2-3 million suspected malaria cases between 1991-2001. Nevertheless, the etiologies of non-malarial AFI are relatively unknown. The non-specific clinical presentation of most febrile illnesses makes diagnosis difficult, thus increasing the need for laboratory-based confirmation. The aim of this study was to identify arboviral infections associated with AFI in Ghana. **Methods:** AFI surveillance began in 2009 at 3 major referral hospitals (Accra, Tema and Tamale) and a military hospital in Ghana. The study used a case definition similar to the WHO dengue fever case definition: hospitalized patient presenting with history/current fever >38°C and no known source (including respiratory) of infection. Subjects were enrolled following protocols previously approved by the institutional review boards. Blood samples were acquired at time of enrollment (acute); convalescent samples were taken when possible. Real-time RT-PCR assays specific for each of the four DENV or CHIKV were used to detect viral RNA. ELISA was used to test for antibodies (IgM and IgG) reactive to different arboviruses including DENV, West Nile virus (WNV), yellow fever virus (YFV) and CHIKV. Virus isolation was attempted when applicable. **Results:** A total of 161 samples from patients hospitalized for AFI were tested. The mean age of cases was 29.1 years; 42.9% were from the military hospital; 29.8% from Accra and Tema respectively (southern coast) and 24.2% from Tamale (northern region). IgG reactive antibodies to DENV, WNV and YFV were detected in 59%, 54% and 5% of the samples respectively. IgG to CHIKV were detected in 35% of the samples. IgM to DENV or WNV were detected in 6% and 2% of the samples respectively. Four samples were positive for DENV-3 RNA and one sample resulted in the isolation of CHIKV. **Conclusions:** Our results show high levels of previous exposure to flaviviruses in Ghana. Confirmation of DENV-3 infections in four patients correlates with recent findings of DENV-3 circulating in other countries of West Africa. In addition, CHIKV was isolated corroborating the possibility of co-circulation of DENV and CHIKV in Ghana.

**Board 61. Isolation of Human Adenovirus C Containing an HAdV-6-like Fiber Gene and HAdV-2-like HVR-7 Region from Patients with Influenza-like Illness in Egypt**

**P. Demian**[1], L. Dickson[2], A. Naguib[3], A. El-Gohary[3], R. Siam[4], A. Kajon[2], C. Cornelius[1];
[1]Naval Medical Research Unit No. 3, Cairo, Egypt, [2]Lovelace Respiratory Research Institute, Albuquerque, NM, USA, Albuquerque, NM, USA, [3]Ministry of Health, Egypt, Cairo, Egypt, [4]American University in Cairo, Cairo, Egypt.

**Background:** Human Adenoviruses (HAdV) can cause a wide range of infections in humans including respiratory tract infections which are mainly caused by HAdV-B and HAdV-C. HAdV is also known for its ability to undergo intraspecies genomic recombination resulting in novel variants and re-shaping phylogenetic relationships. The aim of this study was to examine 59 HAdV-C isolates from Egyptian patients seeking care for influenza like illness (ILI) between 2003 and 2010 for the presence of intertypic recombinants. **Methods:** To this end we sequenced two regions of the genome for each isolate: the 1.8kb fiber gene and the 600bp portion of the hexon gene comprising the HVR-7 (Hypervariable Region-7). Sequences were analyzed and edited using BioEdit, and MEGA4.0 was used to create Maximum likelihood phylogenetic trees for each of the regions. **Results:** Using these methods, as well as BLAST, we identified 10 isolates containing a Human Adenovirus 6 (HAdV-6)-like fiber gene and a Human Adenovirus 2 (HAdV-2)-like HVR-7. These 10 isolates had 99.3-100% identity to one another in the fiber gene which exhibited high similarity to that reported in the Russian HAdV-C strains isolated in the years from 2001 to 2005. The HVR-7 region of the 10 isolates had slightly more variation, 91.4-99.5% identity, as expected. **Conclusions**: Recombination events are common due to the high frequency of Adenoviral co-infections in humans. Additional sequencing of the remainder of the genome, or informational segments of the genome, is required to understand the contribution of each of the parental viruses (HAdV-6 and HAdV-2) to the recombinant strain.

**Board 62. Invasive GAS cases in the New York State Emerging Infections Program, 2000-2009: *emm* Types as Indicators for Virulence**

**G.L. Smith**[1], D.S. Abraham[2], J.B. Karr[3], N.L. Spina[2], K.E. Burzlaff[3], D.J. Kohlerschmidt[4], S.M. Zansky[2];
[1]NYSDOH, Geneva, NY, USA, [2]NYSDOH, Albany, NY, USA, [3]NYSDOH, Rochester, NY, USA, [4]NYS Wadsworth Center Laboratory, Albany, NY, USA.

**Background:** *Streptococcus pyogenes* (GAS) is a pathogen responsible for human illness ranging from minor to serious, life-threatening infections. In NYS, invasive GAS (iGAS) is a reportable disease with several hundred cases investigated annually. We examined iGAS cases and their corresponding isolate *emm* types to identify associations between specific strains and hospitalization (H), extended hospitalization (EH), Streptococcal Toxic Shock Syndrome (STSS), Necrotizing Fasciitis (NF) and death. **Methods:** The NYS Emerging Infections Program (EIP) participates in CDC's Active Bacterial Core surveillance (ABCs) program and conducts surveillance in 15 counties for laboratory-confirmed cases of iGAS, defined as isolation from a sterile site or from a wound specimen obtained from a patient with NF or STSS. All cases are investigated with a standard form. All available isolates are submitted to NYS Wadsworth Center Laboratory for confirmation and subsequently sent to CDC for *emm* typing. EH was defined as greater than 7 days. Death is associated with illness if a hospitalized case died prior to discharge. Differences in distributions of *emm* types by gender, age and race were analyzed. Logistic regression was used to examine the risk of death, H, EH, NF, and STSS by *emm* types. SAS v9.2 was used for analysis. **Results:** NYS EIP investigated 712 iGAS cases from 2000- 2009. Over 40 *emm* types were identified, and the most common *emm* types were 1 (23.6%), 28 (11.9%), 12 (10.2%), 89 (8.7%) and 3 (7.6%), accounting for over 62% of all isolates. There were no significant differences by age, gender or race among the *emm* types. Age was a significant predictor for both H and EH, but *emm* type, race and gender were not. Individuals with *emm* type 1 had significantly greater odds of NF and STSS than those with all other *emm* types. **Conclusions:** We noted numerous iGAS *emm* types, but the majority were not associated with increased virulence. However, *emm* type 1, the most common type in NYS EIP, was associated with more severe presentations such as STSS and NF, a finding similar to national data. As reported by others, a vaccine targeting the most common *emm* types would be beneficial in preventing morbidity and mortality. It is advantageous to continue to monitor iGAS epidemiologic characteristics, including *emm* types to recognize significant trends and changes.

**Board 63. Increasing the Capacity of a PulseNet Network by the Development of a Web-Based Training Program**

**M. Majcher**, K. Karlowsky, D. Sheedy, S. Silcox, B. Szklarczuk, M. Cameron, C. Nadon;
Public Health Agency of Canada, Winnipeg, MB, Canada.

**Background:** PulseNet (PN) is an electronic network for rapidly sharing molecular subtyping data between geographically dispersed members and facilitates early detection and epidemiologic investigation of foodborne disease outbreaks. The PulseNet Canada (PNC) network consists of provincial and federal laboratories which generate and share pulsed-field gel electrophoresis (PFGE) data. The continued success and relevance of PNC's role in mitigating food and waterborne disease outbreak burdens requires consistent and accessible training materials for PulseNet participants. We have developed a comprehensive web-based training (WBT) program to better utilize training resources and enhance PNC network capacity and operations. **Methods:** Online training methods were reviewed and used to guide course design. Curriculum was designed with a modular structure with learning objectives for the skills and expertise required to fulfill PN participation. Standardized, internationally recognized PN methods were the basis for core content development. A web format was built using an approach to best ensure increased accessibility (multiple languages), usability (module based) and operability (laptop, tablet) using a future-proof web-based standard, the Web Experience Toolkit. **Results:** Modules were arranged into 4 learning streams tailored to specific roles: PFGE, Analysis, PFGE and Analysis, and

Public Health Applications. An interactive website was constructed with intuitive module navigation through text and video tutorials that included images, animations, self-guided exercises, and tests. Downloadable and printable content was designed for complete protocols, quick reference sheets, and troubleshooting guides. Materials were also included to assist in PFGE laboratory start-up. The entire course was produced in both English and French. **Conclusions:** An intuitive, streamlined WBT program offering accessible, decentralized training to all participants within a national PulseNet community can potentially increase participation and ultimately strengthen foodborne outbreak detection and response. The development process that generated this WBT program may serve as a model for designing and implementing future WBT tools for other laboratory-based surveillance and analysis.

## Zoonotic and Animal Diseases

Monday, March 12
12:30 PM – 1:30 PM
Grand Hall

**Board 64. Human Leptospirosis in São Paulo, Brazil, and Its Association with Global Climate Change**

**E.C. Romero**, R.M. Blanco;
Adolfo Lutz Institute, Sao Paulo, Brazil.

**Background**: Leptospirosis, a zoonotic disease with great public health importance, is a neglected tropical disease and has now been identified as an emerging infectious disease. Because of global warming, flood is increasing causing outbreak of leptospirosis. A retrospective study was undertaken to describe seasonal patterns of human leptospirosis cases in São Paulo, Brazil between January 1998 and July 2011, to analyse if there was an association between the occurrence of cases and global climate change. **Methods**: A total of 21,271 sera from patients with suspected leptospirosis were analyzed by the microagglutination test. A confirmed case was defined by positive blood culture or serum samples with titer of 1:800 or seroconversion between two samples. Serogroup was determined by the highest titer and cross-agglutination was considered when the highest titer were the same with two or more serogroups. Also, 83 *Leptospira* spp. isolates were identified by MLST, PFGE and serotyping. Data from all patients including age and gender was also collected. **Results**: A total of 2,047 cases were recorded. Although there are a great number of cases no significant variation in the number of cases was observed. The seasonal summer distribution was evident in all years with a peak in March and April; however, 1998-2000 was characterized by a high number of cases. Heavy rainfall was followed by an increase in cases of leptospirosis. Although leptospirosis occurred in persons of all ages, middle-aged adults from 31 to 40 years were most frequently infected (24.2%), mostly males (83.7%). By serology, Icterohaemorrhagiae was the predominant serogroup (56.3%), followed by Autumnalis (8.0%) and Cynopteri (2.4%). Cross-agglutination occurred in 332 cases. A significant finding of this study was that both culture and serology showed Icterohaemorrhagiae as the predominant serogroup in all years. Results of culture also showed an increase of the Canicola serogroup (4.8%). **Conclusions:** Our data suggest that the observed cases of leptospirosis may be linked to climate and its impact on floods and the rodent population. An understanding of the relationship between leptospirosis incidence and heavy rains is indispensable for implementing appropriate preventive measures.

**Board 65. Backyard Poultry Rearing Practices in Bangladesh: Implications for Risk of Avian Influenza**

**I.S. Shanta**[1], M.A. Hasnat[1], A. Mikolon[1,2], M. Salah Uddin Khan[1], N. Haider[1], A.A. Bhuyan[1], M.A. Hossain[3], E.S. Gurley[1], E.A. Baumgartner[2], S.P. Luby[1,2];
[1]ICDDR,B (International Centre for Diarrheal Disease Research, Bangladesh), Dhaka, Bangladesh,

[2]Centers for Disease Control and Prevention, Atlanta, GA, USA, Atlanta, GA, USA, [3]Department of livestock service, Ministry of Fisheries and Livestock, Bangladesh, Dhaka, Bangladesh.
**Background:** Since 2007, highly pathogenic avian influenza A/H5N1 has been confirmed in poultry in 51 of 64 districts of Bangladesh and 58 outbreaks occurred in backyard poultry. Fifty percent of poultry in Bangladesh is raised in backyards where people have close contact with poultry. We assessed poultry raisers' knowledge about avian influenza and compared current poultry raising practices to the practices recommended by the Government of Bangladesh. **Methods:** During May 2009 to July 2011, we enrolled a nationally representative sample of 1753 backyard poultry raisers with flocks of one to 55 poultry from 85 rural village clusters selected by probability proportional to population size. Researchers visited households and collected information about poultry raising practices from owners using a structured questionnaire. **Results:** Fifty-three percent of poultry raisers had never heard of avian influenza or "bird flu". The majority of poultry raisers (95%) reported free range poultry roaming into their homes. Against recommendations, 84% of poultry raisers typically handled sick or dead birds, 52% slaughtered sick birds, and 55% kept sick birds inside the house. In 37% of poultry raisers' households, children touched or played with poultry, and in 16% of households, slaughtered poultry. Forty-three percent of poultry raisers reported never washing their hands with soap after handling poultry, while 0.5% covered their nose and mouth with a cloth when handling poultry. Most (87%) reported fully cooking poultry products before consumption. In spite of recommendations, 46% used poultry manure as fertilizer. Only 1% reported poultry illness and deaths to local authorities. When poultry raisers experienced a flu-like illness, 15% reported consulting a medical doctor and reporting was two times higher in the raisers who heeded avian influenza. **Conclusions:** Four years after the introduction of H5N1 in Bangladesh, the majority of poultry raisers have not heard of avian influenza or "bird flu", and government recommendations are infrequently being followed by backyard poultry producers. Prevention messages should be reevaluated to assess optimal communication channels used for dissemination, the acceptability, feasibility and effectiveness of the proposed behaviors to reduce risk in the target communities.

**Board 66. Exposure to a Rabid Zebra among Tourists and Staff at a Safari Lodge in Kenya, August 2011**

**M. Obonyo**[1], W. Arvelo[2], S. Kadivane[1], M. Orundu[3], E. Lankau[4], P. Munyua[2], D. Mutonga[3], N. Marano[4], J. Githinji[5], C. Rupprecht[4], J. Omolo[1], J. Montgomery[2];
[1]Kenya Field Epidemiology and Laboratory Training Program, Ministry of Public Health and Sanitation, Nairobi, Kenya, [2]Centers for Disease Control and Prevention, Nairobi, Kenya, [3]Ministry of Public Health and Sanitation, Nairobi, Kenya, [4]Centers for Disease Control and Prevention, Atlanta, GA, USA, [5]Central Veterinary Investigation Laboratory, Ministry of Livestock, Nairobi, Kenya.
**Background:** Rabies is a fatal viral infection, resulting in >55,000 deaths globally each year. In August 2011, a young orphaned zebra at a Kenyan safari lodge acquired rabies and potentially exposed >150 tourists and staff. An investigation was initiated to determine exposures among the staff, and to describe animal bite surveillance and preparedness in the affected district. **Methods:** We conducted unstructured interviews among the lodge staff on circumstances surrounding the zebra's illness. Rabies exposure was clinically assessed by a physician. We reviewed animal bite report forms from the outpatient department at the district hospital. Level of district preparedness was assessed using the WHO Expert Consultation on Rabies Prevention and Control checklist. **Results:** The zebra was reported bitten by a dog on July 31, 2011. The zebra became ill on August 23, and died three days later. There were 22 employees working at the lodge during that time. Six (27%) were determined to have high risk (bitten or contact with saliva by bottle feeding or veterinary care) and required four doses of rabies vaccine and one of immune globulin, and 16 (73%) had low risk due to casual contact and received only four doses of rabies vaccine, by WHO recommendations. From January 2010 to September 2011, 118 cases of animal bites were reported in the district; 67 (57%) occurred among males, 65 (55%) in children

<15 years old, and 61 (52%) were inflicted in a lower extremity. Dogs accounted for 98% of reported bites; 96 (81%) were domestic and 23 (19%) were stray. Sixteen (14%) patients received a full course of post exposure prophylaxis (PEP). In terms of district preparedness, communications regarding confirmed rabies in wildlife animal species was limited, and surveillance reporting was linked to PEP provision. **Conclusions:** Dog bites are a main source of rabies exposure in the district, but exposure can also arise from interactions between wildlife and domestic animals, highlighting the importance of a one health approach with strong communication between wildlife, veterinary, and human health sectors in Kenya. Efforts to increase completion of PEP, and integration of animal bite and rabies surveillance should be implemented in the district to improve rabies prevention and control.

**Board 67. Epidemiological Monitoring of Avian Virus Circulation in Nature, an Estimation of Biological Danger of the Pestholes in the Kyrgyz Republic.**

**K.T. Kasymbekova**[1,2], Z. Nurmatov[1], A.Ostashenko[2], D. Raimbekov[3], M. Moldokmatova[1], G. Saparova[1]; [1]DSES MoH Kyrgyz Republic, Bishkek, Kyrgyzstan, [2]2- National Academy of Science, Bishkek, Kyrgyz Republic;, Bishkek, Kyrgyzstan.

**Background:** The role of wild migrant birds (predominantly tied to water and water-marsh lands) in the circulation of influenza virus on the territory of Kyrgyz Republic is determined; danger of infection of poultry by avian flu virus from wild birds is evaluated. **Methods:** During 2 years of the project ISTC KR - 1429 (June 2009 - May 2011) implementation, the surveys of reservoirs of avian flu on the territory of Kyrgyz Republic have been conducted. Expeditionary trips have been organized for this purpose. Swaps from trachea and cloacas from 2424 birds belonging to 74 species have been collected from alive birds. Laboratory diagnostics of avian flu have been executed by molecular genetic and virological methods. Isolation of influenza virus was conducted on MDCK cell cultures lines. We used commercial test systems for RNA-extraction (“QIAmp Viral RNA mini kit”) as well as Real-time PCR tests to detect avian influenza virus. Subtyping of received positive samples by PCR method in Real Time PCR with primers to influenza virus H1, H2, H5 detected. **Results:** To localize natural centers of the virus the survey of wild birds nesting places in summer period is carried out. The main emphasis were made on study of species of nesting colonially birds, as in conditions of colonial nesting a probability of the virus circulation among the population is the most probable. For these purposes nestlings and adult birds have been fowled. In summer colonies of waterfowls in Issyk-Kul lake t were examined. At this time, chicks form colonies of Black-headed gulls, Common Terns, Yellow-legged gulls, Cormorants and Herons have been caught. In Issyk-Kul lake, places of mass nesting of Black-headed gulls, Yellow-legged Gulls, Common Tern, Grey Heron have been identified. RNA influenza virus A has been detected in 38 samples (1,6% out of researched samples). Influenza virus A (H5) detected in one case, the rest 37 samples are not typed. Analyses of the frequency of avian influenza virus detection from species determined that more often avian influenza virus was detected among Mallard - 16 positive findings (42.1%) and black-headed gull-4 positive findings (10.5%). More often influenza A viruses was detected in summer, when it was detected 17 out of 38 positive findings what made 44.7% from positive findings. In autumn the frequency of positive findings was 11 and made 28.9%. In spring the frequency of positive findings was 8 and made 21.1%. The minimal frequency of virus detection was in winter period 5.3% from the number of positive findings. 6 isolates influenza A (from materials from Night Heron, 2 Mallards and Common Teal) with cytopathogenic effect on a cell culture have been detected. **Conclusions:** 1)During researches of 2424 birds belonging to 74 species in 38 samples RNA influenza virus A has been detected (1,6% out of researched samples). 2) More often avian influenza virus was detected among Mallard - 16 positive findings (42.1%) and black-headed gull-4 positive findings (10.5%). 3) More often influenza A viruses was detected in summer.

**Board 68. Persistence of Methicillin-Resistant *Staphylococcus aureus* (MRSA) in Connecticut Pig Farms**

**L.U. Odofin**[1], B. Hanson[2], T.C. Smith[2], R. Heimer[1];
[1]Yale University School of Public Health, New Haven, CT, USA, [2]University of Iowa College of Public Health, Iowa City, IA, USA.

**Background:** There have been many reports on MRSA in livestock, albeit few in the United States (US). Even fewer have investigated persistence of MRSA in the farm settings, thus the premise of this study. This study compares both MRSA-positive and negative farms as identified by a 2009 baseline study conducted a year ago looking at *S. aureus* prevalence on Connecticut farms. **Methods**: From Dec 2010-July 2011 nasal samples were collected from 51 pigs and a human on 5 MRSA-positive pig farms. These were farms that either had MRSA-positive swine or humans at baseline. At the same time, 83 pigs and 2 humans were sampled from 13 MRSA-negative farms. Selection of negative farms was mainly based on proximity to positive farms. Environmental swabs from animal housing (pen rails, feeders, water trough, nursery nipples) were also collected at all 12 farms. Samples were then analyzed using established microbiology methods. *Spa* typing was used to type *S. aureus* strains while Panton-Valentine Leukocidin (PVL) gene detection was done by PCR. A farm assessment form and questionnaire were used to elicit information about husbandry practices and human exposure risks. **Results**: Preliminary results reveal that two out of the five (40%) MRSA positive farms still had MRSA colonized swine (5 pigs); one of the remaining three had a farmer colonized with MSSA. Over a third (38%) of the negative farms had MRSA colonized swine (16/? pigs). According to preliminary typing, one of these farms was colonized with PVL-positive t008 strains (USA 300). Three of the 8 (38%) MRSA-positive farms reported routine use of tylosin, which belongs to same antimicrobial class as erythromycin. Remarkably, 94% (15/16) of these farms` isolates were resistant to erythromycin with over 80% being cross-resistant to clindamycin. However, only three MRSA isolates (3/21; 14%) were resistant to tetracycline in contrast to 100% (8/8) found at baseline. **Conclusions**: USA300 continues to circulate on US farms, suggesting that pigs are competent reservoir for this strain. This finding has possible health implications for farmers and even neighboring communities.

**Board 69. *Cryptosporidium parvum* Zoonotic Genotype Diagnosed in Dairy Calves from Rio de Janeiro State Farms, Brazil**

**T.C. Bomfim, IV**, M.C. do Couto, IV, A.P. Sudré, IV, M.d. Lima, IV;
Universidade Federal Rural do Rio de Janeiro, Rio de Janeiro, Brazil.

**Background:** The dairy farming is one of the most important activities in Brazil. The southeastern region is responsible for great part of the dairy products available in the consumer markets. The presence of symptoms of bovine cryptosporidiosis will depend on the age of the infected animal and the species of *Cryptosporidium* involved. Pre-weaned calves (≤ 2 months of age) may develop diarrhea, weight loss, morbidity and even mortality. On the other hand, usually no clinical symptoms are observed in post-weaned calves (≥ 2 months of age). Four species are commonly found infecting calves, *C. parvum*, *C. andersoni*, *C. bovis* and *C. ryanae,* among those, *C. parvum* is the most important because of its great zoonotic potential. **Methods:** The aim of the present study was to diagnose the presence of *Cryptosporidium* sp. in calves up to one year of age from dairy farms located in the State of Rio de Janeiro, Brazil using optical microscopy and molecular techniques. During one year, 143 fecal samples were collected from calves up to 12 months of age raised in three dairy farms in the State of Rio de Janeiro. The samples were processed using the centrifugal flotation in saturated sugar solution technique and observed under optical microscope with phase contrast. Microscopy positive samples were submitted to molecular techniques (Nested-PCR and sequencing). **Results:** After microscopic examination of all collected samples, 19.6% of the animals were positive for at least one species of *Cryptosporidium*. Twenty-five samples were considered positive for *Cryptosporidium* sp. and were confirmed by Nested-PCR. All material obtained by the Nested-PCR was purified and sent for

sequencing. **Conclusions:** The results obtained by sequencing confirmed the presence of *C. parvum* in pre-weaned calves, demonstrating the relationship between the age of infected calves and the species/genotype of *Cryptosporidium*, highlighting the importance of infection in young animals which are usually infected by zoonotic species.

**Board 70. The Importance of Enzootic Stability in Explaining the Spatio-temporal Pattern of Hemorrhagic Disease in White-tailed Deer**

**K. Magori**[1], D.E. Stallknecht[2], B.A. White[1], A.W. Park[1];
[1]Odum School of Ecology, University of Georgia, Athens, GA, USA, [2]Southeastern Cooperative Wildlife Disease Study, University of Georgia, Athens, GA, USA.

**Background:** Hemorrhagic disease (HD) is the most important infectious disease affecting white-tailed deer (WTD) in North America, causing a range of clinical symptoms from morbidity to outbreaks with massive mortality. HD is caused by several serotypes of related orbiviruses, such as the Epizootic Hemorrhagic Disease Virus (EHDV) and Blue-tongue Virus (BTV), both transmitted by Culicoides biting midges. A particular characteristic of HD is enzootic stability, defined as the absence of clinical disease despite high levels of infection in the host population. Enzootic stability in HD occurs in the Southern US (particularly in TX, OK and KS), where HD is not reported despite virus isolation and close to 100% seroprevalence detected for EHDV and BTV in WTD. **Methods:** Capitalizing on a 30-year surveillance dataset by the Southeastern Cooperative Wildlife Disease Study group (SCWDS), we characterize the complex spatio-temporal pattern of HD. We elucidate the mechanisms responsible for enzootic stability for HD in WTD using a conceptual model. We use statistical models to explain the spatio-temporal pattern of HD across the United States. **Results:** Our conceptual model reproduces the pattern of enzootic stability for HD in WTD through the mechanisms of maternal protection of fawns by antibodies secreted in the milk of does, and partial cross-immunity between serotypes of EHDV and BTV. Statistical models incorporating enzootic stability in the Southern US can explain the annual epizootics of HD viruses and their persistence at the continental scale. The statistical framework incorporating enzootic stability provides reliable probability estimates for the presence of infection, morbidity and mortality, and reporting in a particular county for WTD, which correlate with local risk of infection to livestock. **Conclusions:** Enzootic stability for HD, operating through maternal protection and partial cross-immunity, is a critical phenomenon to incorporate in order to understand the spatio-temporal dynamics of HD and to predict the presence of HD in WTD as well as the risk of infection to livestock.

**Board 71. Mathematical Modeling of H3N8 Canine Influenza Virus Transmission in Shelter Facilities**

**T.C. Anderson**, P.C. Crawford, E.P. Gibbs, J.J. Pohedra, C.C. Lord;
University of Florida, Gainesville, FL, USA.

**Background:** Canine influenza virus subtype H3N8 (H3N8 CIV) is a highly contagious respiratory infection. Dogs housed in communal settings are at greatest risk of infection. The objective of this study was to develop a mathematical model of H3N8 CIV transmission in shelters in order to assess the importance of multiple factors in the likelihood of an epidemic following virus introduction. **Methods:** A deterministic mathematical model was developed for fixed capacity, high turnover shelters. Model classes included susceptible, latent, infectious clinical, infectious subclinical, and recovered dogs, and fomites. Differential equations, representing the change over time for each class during a simulation, were programmed in MATLAB. Model parameters accounted for virus transmission factors, and immigration and emigration due to typical dog movements. Parameter ranges, most probable values, and distributions were established using published literature, experimental data, and expert opinion. Sensitivity analyses were performed to refine these ranges. Latin hypercube sampling was used to generate 500 parameter sets for simulations. Model outputs included: epidemic occurrence, establishment of endemic infection, epidemic duration, numbers of infected dogs, and the relative

contribution of direct and indirect transmission to epidemic dynamics. Stepwise linear regression was conducted to assess the effect of model parameters on select outputs. **Results:** H3N8 CIV epidemics occurred in 498/500 (99.6%) simulations. Endemic infection was established in 100%. The average epidemic lasted 38 days, and large numbers of dogs were affected. Model parameters associated with indirect virus transmission and shelter size most significantly influenced these outcomes. Of 498 epidemics, 21% were primarily due to direct virus transmission, 63% were primarily due to indirect virus transmission, and 16% were due to both. **Conclusions:** Given the model structure and parameter ranges used, and in the face of no interventions, a canine influenza epidemic almost always occurred when an infected dog was introduced into a shelter. Prevention and control strategies that reduce transmission between dogs and fomites and manage the number of incoming susceptible dogs will likely reduce the impact of H3N8 CIV epidemics in shelters.

## Viral Hepatitis

Monday, March 12
12:30 PM – 1:30 PM
Grand Hall

**Board 72. Underestimated Burden of Hepatitis E: Contribution of Small Clusters Identified in Bangladesh**

**H.M. Sazzad**[1], E.S. Gurley[1], J. Hossain[1], A.B. Labrique[2], S.K. Saha[3,4], S.K. Chowdhury[1], S. Islam[1], Y. Khudyakov[5], C.-G. Teo[5], M. Rahman[6], S.P. Luby[1,5];
[1]ICDDR,B, Dhaka, Bangladesh, [2]Johns Hopkins Bloomberg School of Public Health, Baltimore, MD, USA, [3]Popular Diagnostic Centre Ltd., Dhaka, Bangladesh, [4]Department of Microbiology, Dhaka Shishu Hospital,, Dhaka, Bangladesh, [5]Centers for Disease Control and Prevention (CDC), Atlanta, GA, USA, [6]Institute of Epidemiology Disease Control and Research (IEDCR), Dhaka, Bangladesh.

**Background:** Hepatitis E is usually reported in the literature as large outbreaks with thousands of cases associated with contaminated drinking water systems. Small clusters of hepatitis E may occur in endemic areas, but may be less frequently reported to public health authorities. The objective of this analysis was to report on the frequency of small clusters of hepatitis E identified through event based outbreak surveillance and a research project in Bangladesh. **Methods:** We defined a small cluster as at least 2 cases who were <100 anti-HEV-IgM positive with clinical jaundice and developed illness within 2 months of each other and were residing in the same home, neighborhoods or workplaces. We identified these clusters through ongoing outbreak surveillance by IEDCR and ICDDR,B and from a research study in an urban private laboratory in Dhaka. We used an ELISA produced by Diagnostic Systems to identify the cases. **Results:** During November 2008 thru July 2011, we identified 30 small clusters of hepatitis E in Bangladesh. The mean number of clusters was 1 (range: 1 - 4) per month. The median number of cases in each cluster was 3 (range: 2 - 74). The only cluster reported through outbreak surveillance consisted of 74 cases and occurred in a rural area. The remaining 29 clusters were identified from the diagnostic laboratory in urban Dhaka. Seventeen (60%) of urban clusters occurred within families or neighborhoods. Two clusters originated in residential hostels for medical professionals, including the rural one. The remaining eleven were associated with the workplace, including 3 physicians from a pediatric hospital and 10 co-workers at a bank. **Conclusions:** The multiple small clusters of hepatitis E in Bangladesh that we identified through limited surveillance and diagnostics reflect the underestimated burden of hepatitis E in Bangladesh. Many similar small clusters almost certainly occur but remain unrecognized. Burden estimates that only consider the large reported outbreaks may underestimate true burden by missing a substantial number of such cases. Subsequent investigations should focus on

describing transmission routes associated with small clusters. Improved surveillance for and early intervention with small clusters could prevent future large hepatitis E outbreaks that may start as a small clusters.

**Board 73. Risk Factors for Sporadic Hepatitis E in Dhaka, Bangladesh**

**H.M. Sazzad**[1], E.S. Gurley[1], A.B. Labrique[2], S.K. Saha[3,4], Y. Khudyakov[5], C.-G. Teo[5], S.P. Luby[1,5];
[1]ICDDR,B, Dhaka, Bangladesh, [2]Department of International Health and Department of Epidemiology, Johns Hopkins Bloomberg School of Public Health,, Baltimore, MD, USA, [3]Popular Diagnostic Centre Ltd.,, Dhaka, Bangladesh, [4]Department of Microbiology, Dhaka Shishu Hospital, Dhaka, Bangladesh, [5]Centers for Disease Control and Prevention, Atlanta, GA, USA.

**Background:** Fecal contamination of drinking water is associated with large hepatitis E virus (HEV) outbreaks in endemic areas. Sporadic HEV infections in high-income countries have been associated with exposure to blood and animal contact. We hypothesized that sporadic HEV in Dhaka, Bangladesh, could be due to contaminated drinking water, contact with other people's infected blood, contact with animals, meat consumption, or having oral sex. **Methods:** We defined sporadic cases as anti HEV IgM-positive persons with jaundice identified between November 2008 and October 2009 with no known occurrence of jaundice in their home, neighborhood or workplace within the preceding 2 months. We selected eligible cases from a diagnostic centre in Dhaka, and then selected age-matched controls from both sick and healthy anicteric clients presenting at the same facility with a normal blood count. We conducted face-to-face interviews with cases and controls; cases and sick controls were asked about their exposures in the 2 months prior to illness onset. Healthy controls were asked about exposures in the 2 months prior to their visit to the diagnostic center. We asked about: contact with another person's blood; contact with sharp instruments, including therapeutic injections prior to the visit to the diagnostic center, and a barber's razor; contact with animals; consuming meat; performing oral sex; and drinking water likely to be unclean. We built a multivariate conditional logistic regression model with the pooled exposures that represent common transmission pathways and were associated ($p<0.2$) with HEV in univariate analysis. **Results:** Of the109 cases and 109 controls, more cases than controls were male (65% vs. 43%, p=0.001). In univariate analysis risks identified were: being male; contact with another person's blood or sharing sharp instruments; animal contact in the home; and stagnant water near home. In multivariate analysis independent risks were: being male (adjusted mOR 2.2, 95% CI: 1.2 - 3.9; $p$=0.01) and contact with another person's blood or sharing sharp instruments (adjusted mOR 2.1, 95% CI: 1.1 - 4.1; $p$=0.03). **Discussion:** HEV can be blood-borne. Efforts to prevent blood borne infections, such as promotion of safe injections and using disposable blades at the barber's may reduce the risk of sporadic HEV infection in Dhaka.

**Board 74. Characterization of Foreign-born Chronic Hepatitis B cases in 7 Population-based Surveillance Sites, United States, 2001-2010**

**S. Liu**[1], K. Bornschlegel[2], S. Shallow[3], K. Iqbal[4], E. Rizzo[5], K. Gerard[6], G. Van Ness[7], M. Klevens[4];
[1]CDC/ORISE, Atlanta, GA, USA, [2]New York City Department of Health and Mental Hygiene, New York, NY, USA, [3]Communicable Disease Control and Prevention San Francisco Department of Public Health, San Francisco, CA, USA, [4]CDC, Atlanta, GA, USA, [5]New York State Department of Health, Albany, NY, USA, [6]Connecticut Department of Public Health, Hartford, CT, USA, [7]Oregon Public Health Division, Portland, OR, USA.

**Background:** Although national surveys indicate that the prevalence of chronic hepatitis B among foreign-born persons in the United States has decreased since 1999, persons born outside the US nonetheless have a 5.6 times higher infection prevalence than US-born persons. Understanding infection among foreign-born persons is critical for future prevention activities. **Methods:** CDC funded chronic hepatitis B surveillance activities in health departments in Colorado, San Francisco, Minnesota,

34 counties in New York State, New York City, Oregon, and Connecticut. Staff at these sites collected demographic data on all cases that met the CDC case definition and investigated all or a sample of cases to collect supplemental epidemiologic information. Using 2001-2010 data from those sites, we classified the reported birth countries by WHO region, selected cases with known country of birth, and tested differences between foreign- and US-born cases. We restricted trend analyses to 2005-2010 due to more complete data reporting for those years. **Results:** A total of 79,221 chronic hepatitis B cases were reported from the 7 sites during 2001-2010. Of these, 23,181 (29.2%) reported place of birth; 20,776 (26.2%) outside the US and 2,405 (3.0%) US born. Of the foreign-born cases, 8,161 (39.3%) specified a country of birth. Foreign-born cases were more likely than US-born to be female (44.1% vs 36.6%, $p<0.0001$) and to be aged 20-49 years (65.7% vs 56.0%, $p<0.0001$). The largest percentage of foreign-born cases (n=3,471; 40.8%) were from the Western Pacific region, predominantly China and Vietnam; followed by Southeast Asia region (n=1,799; 21.1%), predominantly Burma; and Eastern Mediterranean countries (n=1,055; 12.4%), predominantly Somalia. During 2005-2010, hepatitis B cases among foreign-born persons increased 109% from 2,021 to 4,232 and were much more likely to be reported than those among US-born persons. **Conclusions:** We found a high and increasing number of foreign born persons with chronic hepatitis B, especially women and young adults. These surveillance data are useful for targeting prevention activities, such as screening and educational efforts for immigrants from high prevalence countries.

**Board 75. Increasing Rates of *Hepatitis C* Past or Present Infection Reports Among Adolescents and Young Adults in Pennsylvania.**

**S.W. Boktor**, K.O. Waller, S.M. Ostroff;
Pennsylvania Department of Health, Harrisburg, PA, USA.

**Background:** Chronic hepatitis C infection is the leading cause of advanced liver cirrhosis and liver cancer in the United States. The infection is most prevalent among persons 45 to 60 years of age, but limited data from a few states suggest an emerging trend of chronic infection in adolescents and young adults. We assessed surveillance data in Pennsylvania to identify whether a similar trend was occurring in younger age groups. **Method:** We reviewed Pennsylvania's hepatitis C surveillance data for 2003 (the first full year of data after hepatitis C became reportable in Pennsylvania) to 2010. Most reports of hepatitis C are received from laboratories via electronic laboratory reporting. Age-specific rates of reported cases were calculated, and then compared over time. Spatial and demographic characteristics were also assessed. **Results:** For the age group 15-34 years, the number of reports of newly recognized cases of chronic hepatitis C increased from 1,384 in 2003 to 2,393 in 2010. This resulted in a near doubling of rates from 43 to 72 cases per 100,000 population. In contrast, overall rates of newly reported cases for all age groups combined declined from 85 to 72 cases per 100,000 from 2003 to 2010. A similar decreasing trend was seen in the 45-64 year old age group (185 to 142 cases per 100,000 from 2003 to 2010). The proportion of cases in males in the 15-34 year old age group rose from 50% in 2003 to 63% in 2010. The change in rates between 2003 and 2010 appears largest in some rural areas of Pennsylvania rather than in the two large urban centers. **Conclusions:** It appears that a new wave of hepatitis C infections has developed in adolescents and young adults in Pennsylvania. This has profound consequences for the control of this disease and has implications for long-term treatment of infected individuals. Studies are urgently needed to better characterize risk factors for hepatitis C infection in this age group, so that effective interventions strategies can be designed.

## E1. Zoonotic and Animal Diseases

Monday, March 12
3:15 PM – 4:45 PM
Centennial I

**Novel Polyomaviruses from Bats in Kenya and Guatemala: A Potential for Emerging Zoonotic PyV Infection**

**Y. Tao**[1], C. Conrardy[1], M. Shi[1], I.V. Kuzmin[1], S. Recuenco[1], B. Agwanda[2], D.A. Alvarez Castillo[3], J.A. Ellison[1], A.S. Turmelle[1], M. Niezgoda[1], D. Moran[3], R.F. Breiman[4], K.A. Lindblade[5], C.E. Rupprecht[1], L.J. Anderson[1], S. Tong[1];
[1]Centers for Disease Control and Prevention, Atlanta, GA, USA, [2]National Museum, Kenya Wildlife Service, Nairobi, Kenya, [3]Center for Health Studies, Universidad del Valle de Guatemala, Guatemala City, Guatemala, [4]Global Disease Detection Division, Centers for Disease Control and Prevention-Kenya, Nairobi, Kenya, [5]CDC Regional Office for Central America and Panama, Guatemala City, Guatemala.

**Background:** Polyomaviruses (PyV) are small nonenveloped dsDNA viruses. PyV infections are usually mild or asymptomatic, with symptomatic disease occurring primarily in immunosuppressed patients. They have also demonstrated oncogenic potential. To date, 9 human PyVs have been identified and the PyV family has greatly expanded to more than 30 species and 3 genera. There are likely to be many more as of yet unidentified PyVs infecting humans and other animals. **Methods:** A total of 402 bats (221 bats of more than 20 species from Kenya during 2006 and 181 bats of 15 species from Guatemala during 2009) were sampled. Fecal and oral swabs (n=391) were screened for PyV by a pan PyV PCR. Each positive PCR amplicon (402 bp) was sequenced and a Bayesian tree was constructed for the sequences obtained. Full genome sequencing was performed for representatives of each phylogenetic group to characterize genomic organization and to attempt to better define evolutionary relationships. **Results:** Twenty five Kenyan fecal swabs and 1 Guatemalan oral swab were PyV positive. Among the 26 PyVs, 10 were grouped with Merkel Cell PyV in 4 different lineages; 9 were grouped with chimpanzee PyVs and segregated in 2 lineages; 2 were distant relatives of *Myotis* bat PyV; 1 was grouped with sea lion PyV; the other 4 in 3 different lineages formed a long branch with uncertain phylogenetic position. These 12 lineages were harbored by 8 bat species and may represent 12 new putative PyV species based on their genetic divergence (65-77% nt identity). The 7 representative full genomes from 12 different lineages had typical PyV genome organizations with a size range of 4899-5372 bp in length. In addition, we observed phylogenetic incongruence for PyV G203, whose VP1-VP2 region was grouped with the murine-bovine-sea lion cluster while the LT region was grouped with JC-BK-SA12-SV40 cluster, suggesting a potential recombination in this virus. **Conclusions:** We detected 12 distinct, potentially novel genetic lineages of PyVs in bats from Kenya and Guatemala, greatly expanding current PyV diversity. The diversity of PyVs found in bats suggests bats are likely reservoirs of PyV and represent a potential source of zoonotic PyV infection. We also for the first time show genomic recombination occurs during PyV evolution.

**Understanding the Ecology of Nipah Virus in Pteropodid Bats in Bangladesh**

**J.H. Epstein**[1], A. Islam[1], S. Ali Khan[2], M. Sanchez[3], K.J. Olival[1], S. Khan[4], A.M. Kilpatrick[5], M.J. Hossein[4], S.J. Anthony[3], G. Crameri[6], E.S. Gurley[4], T. Briese[3], L. Wang[6], W.I. Lipkin[3], S.P. Luby[4], P. Daszak[1];
[1]EcoHealth Alliance, New York, NY, USA, [2]Chittagong Veterinary and Animal Sciences University, Chittagong, Bangladesh, [3]Center for Infection and Immunity, Mailman School of Public Health, Columbia University, New York, NY, USA, [4]International Centre for Diarrheal Disease Research, Bangladesh, Dhaka, Bangladesh, [5]University of California at Santa Cruz, Santa Cruz, CA, USA, [6]Australian Animal Health Laboratory, Geelong, Australia.

**Background**: Nipah virus (NiV) is an emerging zoonotic virus that has caused near-annual outbreaks of encephalitis in Bangladesh between 2001 and 2011 with greater than 75% mortality. Infections are seasonal (Dec-Apr) and almost exclusively detected within a western region of the country termed the "Nipah Belt." *Pteropus* bat species are a reservoir for henipaviruses in other parts of their range, though little is understood about NiV in bats in Bangladesh. We are studying NiV dynamics in fruit bats to determine how they relate to human infection patterns. Here we provide preliminary data for NiV exposure and infection in several bat species and movement data for *Pteropus giganteus*. **Methods**: From 2006-2011 we collected urine, blood, and saliva from 100 *Pteropus giganteus* in 4 districts within and 4 outside the Nipah Belt. 100 *P. giganteus* were sampled quarterly from a single population within the Belt. 140 *Rousettus leschenaultii* and 75 *Cynopterus spp.* were also sampled within the Belt. Samples were stored in lysis buffer at -80C. A subset of urine and saliva samples has been screened for NiV RNA by nested PCR. We screened *P. giganteus* sera for anti-NiV IgG antibodies using an indirect ELISA and other bat sera by a recombinant expressed NiV-N antigen ELISA and Luminex assay. 16 *P. giganteus* were fitted with satellite transmitters to observe their movements. **Results**: NiV RNA-positive *P. giganteus* were only identified within the Nipah Belt between November and June. IgG-positive bats were found across Bangladesh (seroprevalence: 20% - 56%). Other bat species tested negative for anti-NiV antibodies and RNA. *P. giganteus* primarily remained within 10km of their roost, however, four bats traveled to new roosts between 90 and 280 km away. **Conclusions**: We found evidence confirming *P. giganteus* as a reservoir for Nipah virus in Bangladesh. NiV RNA was only detected in bats inside the Nipah Belt, but during a larger period (Nov-Jun) than in humans. *Pteropus giganteus* appears to carry NiV throughout Bangladesh. Negative results from other bat species suggest that they may have a less important role than *P. giganteus* in NiV transmission. Human activities that occur between December and April that increase contact with bat excreta within the Nipah Belt may be particularly important for explaining observed human infection patterns.

**Isolation of *Salmonella* Oranienburg from Horses and Wild Turkeys on a Ranch in Northern California, and Contamination of the Family's Edible Home Garden Following Raw Manure Application**

**M. Jay-Russell**, J.E. Madigan, Y. Liu, A. Fisher, S. Madigan, B.A. Byrne;
University of California, Davis, Davis, CA, USA.

**Background:** In July-August 2010, an outbreak of *Salmonella enterica* serotype Oranienburg occurred among 6 horses at a private ranch in Mendocino County, California. The index case was hospitalized for colic surgery. We conducted a farm investigation to identify potential sources of *Salmonella*. The owners reported larger than usual populations of wild turkeys on their farm compared to previous years, with groups of over 30 turkeys often congregating in the horse pens, feeding areas, and water troughs. The owners also reported using untreated raw horse manure as a fertilizer in their edible home garden, and had experienced an undiagnosed diarrheal illness at the time of the horse salmonellosis outbreak. **Methods:** We visited the ranch twice in August 2010 to collect environmental samples. To monitor for persistence of viable *Salmonella*, we re-sampled the garden soil every 1-2 months through March 2011. Clinical and environmental samples were cultured for *Salmonella* using enrichment and selective plating. Confirmed isolates were serotyped at the USDA National Veterinary Services Laboratory, and subtyped by PFGE analysis using the PulseNet protocol. **Results***:* *Salmonella* was isolated from 6 (75%) of 8 horses and an asymptomatic pet dog. Positive samples from the barn area included 2 (29%) of 7 water troughs and 4 (50%) of 8 composite samples from the manure storage pile. Sixteen (22%) of 71 wild turkey fecal samples collected around the barn and pastures were positive, in addition to 9 (18%) of 51 garden soil samples. Well water, garden vegetables, and other animal feces (pet cat, wild rabbit) were negative. *Salmonella* Oranienburg with a PFGE pattern indistinguishable from the horse outbreak strain was found in wild turkey feces, stored manure, water trough, and garden soil samples. Viable *Salmonella* persisted in garden soil for 7 months. **Conclusions:** The outbreak illustrates the potential for widespread

dissemination of a rare *Salmonella* serotype in a ranch environment. Because wild birds are common reservoirs of *Salmonella*, we speculate that wild turkey feces were the source of the outbreak. However, we cannot rule-out the possibility that turkeys were exposed on the ranch. The results also underscore the need to educate the public about food safety hazards associated with using raw manure on edible home gardens.

**Is That a Rodent in Your Luggage? A Look into Bushmeat Importation into the United States - September 2005-December 2010**

**T.R. Bell**[1,2], H. Bair-Brake[1], A.L. Higgins[1], S. Shapiro[1], N. Marano[1], N.J. Cohen[1], G. Galland[1];
[1]Centers for Disease Control and Prevention, Atlanta, GA, USA, [2]Council of State and Territorial Epidemiologists, Atlanta, GA, USA.

**Background:** Bushmeat, defined as meat derived from wild animals, is considered a potential source of human infection. The HIV epidemic has been associated with the hunting and processing of bushmeat, and recent studies have found evidence of simian foamy viruses in bushmeat samples confiscated at United States ports of entry. Existing US regulations prohibit importation of bushmeat from specific animals; however, an unknown amount of illegal importation still occurs. This project on illegal US importation of bushmeat includes a descriptive analysis of surveillance data and a qualitative analysis of focus group responses among African expatriates. **Methods:** A keyword search was performed in the Centers for Disease Control and Prevention's Quarantine Activity Reporting System (QARS) to capture all bushmeat-related reports from September 2005 through December 2010; reports were analyzed to identify geographic and seasonal trends. Three focus groups of African expatriates were held in Atlanta, Georgia in June 2009. **Results:** In total, 543 confiscated bushmeat items were recorded in QARS. Half of the confiscated bushmeat was identified as rodent. Africa was the most frequent continent of origin, with 68% of all confiscated bushmeat originating from Ghana and Nigeria. Seasonality was evident, with bushmeat confiscations peaking in late spring to early summer after adjustment for travel volume. Four times more bushmeat was confiscated during an enhanced surveillance program in June 2010 than during the same period in previous years. The 25 total focus group participants revealed that bushmeat importation is a multifaceted issue. Participants had a wide range of responses regarding knowledge attitudes and practices about bushmeat supply and demand, its health benefits or consequences, and importation restrictions. Nearly all who responded to a question specifically about legality of importation thought that bushmeat was legal, in at least some situations. **Conclusions:** Even with regulations in place, bushmeat is entering the United States. Longstanding cultural practices make it difficult for persons to acknowledge potential health risks. Enforcing penalties associated with bushmeat confiscations, along with health education aimed at high-risk groups, may be useful to deter import attempts.

**Tracking Pathogen Transmission at the Human-Wildlife Interface: Banded Mongoose (*Mungos mungo*) and *Escherichia coli* as a Model System in Chobe, Botswana**

**R.R. Pesapane**, M. Ponder, K. Alexander;
Virginia Tech, Blacksburg, VA, USA.

**Background:** A primary challenge to managing emerging infectious disease (EID) is identifying coupling points where pathogen transmission occurs at the human-wildlife-environmental interface. Anthropogenic changes function as drivers of EID and human waste is the most ubiquitous disturbance. While there is growing interest in understanding and managing the public health threat of EID emanating from multihost pathogens, little is known regarding transmission pathways that link humans and animals. Are microorganisms being exchanged between humans and wildlife and what transmission pathways link these populations? **Methods:** We use *Escherichia coli*, a model organism, and banded mongoose (*Mungos mungo)* for evaluating exchange of waste-borne microorganisms at the human-

wildlife interface. Fecal *E. coli* isolates were collected from five mongoose troops that live closely with humans as well as one troop with limited human contact. Human sources of fecal waste in the environment were collected from bush latrines and sewage. Only antibiotic-resistant mongoose isolates were used in the analysis as resistance has been demonstrated as a sensitive marker for determining isolates of human origin. Phylogenetic relationships were determined through rep-PCR fingerprinting and MLST-PCR sequence analysis. **Results:** Antibiotic resistance was found in 56.5% of all mongoose sampled from study troops with high levels of multi-drug resistance detected among isolates (27.9%). Mongoose and human isolates consistently cluster together in rep- and MLST-PCR phylogenetic analysis demonstrating that transmission events are ongoing. AMOVA and $F_{ST}$ values ($F_{ST}$=0.002) show no significant differences between *E. coli* from human and mongoose populations (p=0.18) therefore confirming that these populations are readily exchanging resistant strains of *E. coli* and that human fecal waste is an important link for microorganism transmission. **Conclusions:** Microorganism transmission does occur at the human-wildlife interface through human fecal waste, identifying an important mechanism for the transmission of pathogens of concern to both public and animal health. In this study, human fecal waste is also identified as an important mechanism for the spread of antibiotic resistance in the environment, an emerging global health threat.

**Surveillance of Coronaviruses in Kenyan Bats from 2006-2010**

**C. Conrardy**[1], M. Shi[1,2], I.V. Kuzmin[1], J. Zhang[1,3], B. Agwanda[4], M. Niezgoda[1], R.F. Breiman[5], C.E. Rupprecht[1], L.J. Anderson[6], S. Tong[1];
[1]Centers for Disease Control and Prevention, Atlanta, GA, USA, [2]Center for Infectious Disease Dynamics, Pennsylvania State, University Park, PA, USA, [3]Atlanta Research and Education Foundation, Decatur, GA, USA, [4]National Museum, Kenya Wildlife Service, Nairobi, Kenya, [5]Global Disease Detection Division, Centers for Disease Control-Kenya, Nairobi, Kenya, [6]Division of Infectious Disease, Emory University School of Medicine, Atlanta, GA, USA.

**Background:** The 2003 outbreak of SARS generated renewed interest in coronaviruses (CoVs). CoV surveillance studies in bats have been undertaken in Asia, Europe, the Americas and Africa. As a result, a diverse number of *alpha* and *beta* CoVs , including SARS-like CoVs have been detected in bats. The purpose of this study is to define the prevalence and diversity of CoVs in Kenya bats. **Methods:** Over 2,200 bats from 17 genera were collected during 2006-2010 from 43 different locations in southern Kenya. Fecal and oral swabs were screened for the presence of CoV RNA using semi-nested RT-PCR with consensus degenerate primers targeting CoV Orf1b. The CoV RNA positive samples were sequenced to obtain 350bp fragments, followed by alignment with CoV reference sequences. A phylogenetic tree was generated using maximum likelihood method. In addition, we assessed spatial dynamics and host structure of CoVs by examining the correlation between virus genetic distance matrices and corresponding host genetic and geographic distance matrices. **Results:** The CoV RNA positive rate ranged from10-25% between 2006-2010, depending on the year, location, and bat species sampled. Diverse CoVs detected in Kenya bats formed 13 clusters within *alpha-* and *betacoronavirus*, which significantly extends the currently described CoV diversity. Among these, 6 clusters merited a potential assignment of novel CoV species based on inter-cluster genetic distances. In addition, we found CoVs that shared >80% sequence identity with human CoV 229E and NL63, which may suggest a zoonotic origin of human 229E and NL63. The comparisons of CoVs and bats phylogenetic trees indicated that CoVs are unlikely to co-evolve with bats. On the contrary, the diversity of CoV was shaped by dynamic processes which included frequent host switches and efficient long distance dispersal over large geographic space. **Conclusions:** This study demonstrates the diversity of CoVs in bats from Kenya. These data support previous proposals that bats are likely the primary host reservoir of *alpha* and *beta* CoVs and may carry the progenitors of common human respiratory CoVs such as NL63 and 229E. Continued

surveillance of bats as reservoirs of CoVs could elucidate the evolution of CoVs and their potential impact on human health.

## E2. Vaccine Issues

Monday, March 12
3:15 PM – 4:45 PM
Centennial II

**Parasitism in Children Aged 3 Years and Under: Effects on Growth and Vaccine Response in Rural Coastal Kenya**

**A. LaBeaud**[1], M. McKibben[2], P. Mungai[3], E. McKibben[2], G. Gildengorin[1], L.J. Sutherland[2], C.L. King[2], I. Malhotra[2];
[1]Children's Hospital Oakland Research Institute, Oakland, CA, USA, [2]Case Western Reserve University, Cleveland, OH, USA, [3]Ministry of Health, Msambweni, Kenya.

**Background:** Children are at high risk for helminth and protozoal infections, although estimates of parasitic burden and effects on growth are lacking in those under age 5 years. Recent evidence shows that parasitic infections may also alter vaccine response. Our objective was to document the prevalence of parasites and their effects on growth and response to childhood vaccines in young children in coastal Kenya. **Methods:** Stool, urine, and blood samples were collected from children at 6 month intervals until age 3 years and tested for soil-transmitted helminths (STH: *Ascaris, Trichuris*, hookworm, *Strongyloides*), protozoa (malaria, *Giardia*), and schistosomiasis. Height, weight, and head circumference (HC) were measured at each visit. Prenatal maternal helminth and protozoal infections were documented. Response to tetanus, diphtheria, hepatitis B virus, *Haemophilus influenzae* type B, and poliovirus vaccinations were measured by standard ELISA. **Results:** Of 545 children, 32% had parasitism and 8% had polyparasitism. Hookworm was most prevalent STH (11%), followed by *Trichuris* (10%), *Ascaris* (4%) and *Strongyloides* (2%). *Giardia* was the most prevalent protozoan (13%) followed by malaria (12%). 4% had schistosomiasis by IgG4 testing. Early childhood infection with STH, hookworm, and malaria were associated with maternal infection. Polyparasitized children were more likely to have polyparasitized mothers ($p=0.01$) and have poor HC growth rate (0.002). Children with hookworm ($p=0.01$), any STH ($p=0.049$) or any parasitic infection ($p=0.039$) had slower weight gain. Children with hookworm, *Trichuris*, or *Giardia* had statistically lower tetanus titers than uninfected children; those with malaria or any parasitic infection had statistically lower diphtheria titers. **Conclusions:** Our results document the under recognized burden of parasitism in children aged 0-36 months in rural Kenya. Parasitic infections in this young age group have detrimental effects on weight, height, and HC growth rates and may have significant implications on child health. Certain parasitic infections in childhood, such as STH and malaria, may also be linked to decreased response to standard childhood vaccinations.

**Early Success of First Conjugate Serogroup A Vaccination Campaign in Burkina Faso**

**R.T. Novak**[1], L. Kambou[2], F. Diomandé[1,3], F.T. Tarbangdo[2], R. Ouedraogo[4], L. Sangaré[5], C. Lingani[3], S. Martin[1], L. Mayer[1], M. LaForce[6], F. Avokey[3], M. Djingarey[3], T.A. Clark[1], S. Tiendregeogo[2], N. Messonnier[1];
[1]Centers for Disease Control and Prevention, Atlanta, GA, USA, [2]Direction de la Lutte contre la Maladie, Ministère de la Santé, Ouagadougou, Burkina Faso, [3]WHO Intercountry Support Team for West Africa, Ouagadougou, Burkina Faso, [4]Centre Hospitalier Universitaire Pédiatrique Charles de Gaulle, Ouagadougou, Burkina Faso, [5]Centre Hospitalier Universitaire Yalgado, Ouagadougou, Burkina Faso, [6]Meningitis Vaccine Project at the Program for Appropriate Technology in Health, Washington, DC, USA.

**Background:** Serogroup A (SGA) meningococcus causes major epidemics of meningitis in sub-Saharan Africa. An affordable, highly immunogenic SGA meningococcal conjugate vaccine (MenAfriVac) was licensed in late 2009 and prequalified by the World Health Organization. In late 2010, Burkina Faso became the first country to implement a national preventive campaign, vaccinating 11 million aged 1-29 years (estimated >90% coverage). **Methods:** We examined national population-based and enhanced laboratory-based surveillance data from Jan 1997 through June 2011 and from 2005 through 2011, respectively, to evaluate changes in the burden of meningitis syndrome and laboratory-confirmed SGA meningococcal meningitis. We assessed changes in incidence of suspected meningitis, bacterial meningitis, and serogroup-specific meningococcal disease and examined trends at district level. To assess impact of vaccination, weekly cumulative incidence was modeled using a piecewise exponential survival model. **Results:** During the 14-year period, 152,103 cases and 18,481 deaths of suspected meningitis were reported. Compared to the pre-MenAfriVac period from 1997 through 2010, after vaccine introduction in 2011, the national mean suspected meningitis incidence rate decreased by 80% from 82.0 per 100,000 population to 16.6 per 100,000. There was a 71% decline in risk of meningitis (hazard ratio [HR] = 0.29, 95% CI 0.28-0.30) and a 64% decline in risk of fatal meningitis (HR = 0.36, 95% CI 0.33-0.40). In 2011, none of the 63 districts experienced epidemics (incidence ≥100 cases per 100,000) whereas at least one district experienced an epidemic in each pre-MenAfriVac year. We observed a statistically significant decrease in incidence of bacterial meningitis across all age groups targeted for vaccination and among persons ≥30 year old who were vaccine ineligible. The incidence of laboratory-confirmed SGA *Neisseria meningitidis* dropped significantly to 0.01 per 100,000 following vaccination. **Conclusions:** Early evidence suggests the SGA conjugate vaccine greatly reduced the rate of meningitis in persons in the target age-group, and in the general population due to high coverage and herd immunity. These early data suggest that fully implementing MenAfriVac in at-risk countries could end epidemic meningitis in sub-Saharan Africa.

**An Update on Hospitalized Pneumococcal Bacteremia in Rural Thailand**

**J.C. Rhodes**[1], S. Dejsirilert[2], P. Jorakate[1], A. Kaewpan[1], P. Salika[1], T. Akarachotpong[1], P. Prapasiri[1], S. Naorat[1], P. Areerat[3], A. Ruayajin[4], P. Sawanpanyalert[2], S. Maloney[1], L. Peruski Jr[1], H. Baggett[1];
[1]Thailand MOPH-US Centers for Disease Control Collaboration, Nonthaburi, Thailand, [2]National Institute of Health, MOPH, Nonthaburi, Thailand, [3]Nakhon Phanom Provincial Health Office, Nakhon Phanom, Thailand, [4]Sa Kaeo Provincial Health Office, Sa Kaeo, Thailand.

**Background:** *Streptococcus pneumoniae* causes an estimated 185,000 deaths in Southeast Asia annually. Pneumococcal conjugate vaccine (PCV) is highly effective, but costs prohibitive use in Thailand and other middle income countries not eligible for GAVI funding. Market forces may reduce PCV costs in the near future, making updated burden estimates critical for policy discussions. **Methods:** We implemented automated blood culture systems in two rural Thai provinces as part of population-based surveillance for bacteremia. Blood cultures were collected from hospitalized patients as clinically indicated. A case of pneumococcal bacteremia was defined as blood culture isolation of *S. pneumoniae*. **Results:** From May 2005 - March 2010, 200 cases of pneumococcal bacteremia were confirmed in hospitalized patients. Of these, 84% had clinical pneumonia, 64% received oxygen therapy, 29% required mechanical ventilation, and 23% (n=46) died. Serum antimicrobial testing confirmed pre-culture antibiotic treatment in 25% of patients overall vs. only 3.5% of cases. Annual incidence of hospitalized pneumococcal bacteremia was 3.6 per 100,000 person-years overall; higher in children aged <5 years at 11.7 and adults ≥65 years at 14.2, and highest among infants <1 year at 33.8. Mean case count per month was higher during December - March compared to the rest of the year: 6.6 vs. 2.1 (p<0.001). The most common serotypes were 14 (13%) and 23F (14%); 49% (65% in patients <5 years) were serotypes in the 7-valent PCV (PCV7) and 71% (83% in <5 years) in PCV13. Antibiotic non-susceptibility was high for co-trimoxazole (57%), erythromycin (30%), and clindamycin (20%), and higher in PCV 7 serotypes. One isolate from 2007 was

penicillin non-susceptible. **Conclusions:** We demonstrate a high pneumococcal bacteremia burden, yet we surely underestimate incidence by detecting hospitalized cases only, by depending on underutilized and low sensitivity blood cultures, and because of high rates of pre-culture antibiotics. Our findings corroborate those of other Thai researchers that PCV can be expected to have high serotype coverage and may reduce antibiotic resistance through prevention of infections with non-susceptible organisms. These findings will complement ongoing cost effectiveness analyses to support vaccine policy evaluation in Thailand.

**Missed Opportunities for PCV13 Administration, United States, 2010–2011**

**C.M. Cox**[1], R. Link-Gelles[1], M.M. Farley[2], W. Schaffner[3], A. Thomas[4], A. Reingold[5], L.H. Harrison[6], C. Lexau[7], S. Zansky[8], S. Petit[9], K. Gershman[10], M. Nichols[11], R. Guevara[12], J.W. Anderson[13], J. Rosen[14], B.A. Juni[7], L. McGee[1], B.W. Beall[1], M.R. Moore[1];
[1]Centers for Disease Control and Prevention, Atlanta, GA, USA, [2]Emory University and VAMC, Atlanta, GA, USA, [3]Vanderbilt University, Nashville, TN, USA, [4]Oregon Public Health Division, Portland, OR, USA, [5]University of California, Berkeley, Berkeley, CA, USA, [6]Johns Hopkins Bloomberg School of Public Health, Baltimore, MD, USA, [7]Minnesota Department of Health, St. Paul, MN, USA, [8]New York State Department of Health, Albany, NY, USA, [9]Connecticut Department of Public Health, Hartford, CT, USA, [10]Colorado Department of Health and Environment, Denver, DE, USA, [11]New Mexico Department of Health, Santa Fe, NM, USA, [12]Los Angeles County Department of Public Health, Los Angeles, CA, USA, [13]Utah Department of Health, Salt Lake City, UT, USA, [14]New York City Department of Health and Mental Hygiene, New York City, NY, USA.

**Background:** On March 12, 2010, the Advisory Committee on Immunization Practices (ACIP) published recommendations for use of a 13-valent Pneumococcal Conjugate Vaccine (PCV13) which includes 6 serotypes not in the previously available 7-valent vaccine (PCV7). A single supplemental dose of PCV13 was recommended for all children aged 14-59 months who had previously received an age-appropriate series of PCV7. We sought to evaluate implementation of this recommendation. **Methods:** We analyzed data from CDC's ongoing PCV13 vaccine effectiveness evaluation to identify cases of IPD caused by pneumococcal serotypes unique to PCV13 following vaccine introduction. Twelve areas across the United States participate in the evaluation, representing a population of 3.2 million children younger than 5 years of age. Children who were not eligible to receive PCV13 at least two weeks before IPD diagnosis, those who received one or more doses of PCV13, and those without a complete vaccination history were excluded from the analysis. **Results:** During May 1, 2010 through April 30, 2011, we identified 135 total cases of IPD secondary to pneumococcal serotypes unique to PCV13, of which 81 (58%) had a complete vaccination history. Of these, 66 had not previously received a dose of PCV13. Three of the 66 cases were ineligible to receive PCV13 given dates of PCV7 receipt and timing of disease onset. Of the 63 remaining cases, 43 (68%) were in children 2-4 years of age. The majority of children (94%) had no underlying medical conditions. Forty-eight (76%) were hospitalized with no reported deaths. Thirty-nine cases (62%) were in children who had received a full PCV7 series but had not received a supplemental PCV13 dose; an additional 11 (18%) had received 3 PCV7 doses but not a fourth dose, which should have been PCV13. **Conclusions:** In the year following PCV13 introduction, IPD cases due to pneumococcal serotypes unique to PCV13 continued to occur among children who had not received recommended a supplemental dose of PCV13. To improve prevention of invasive pneumococcal disease, healthcare providers should provide a single supplemental dose of PCV13 to all children 14 through 59 months of age who have received an age-appropriate series of PCV7.

**Varicella Disease in Beijing, China, 2007–2010**

**C. Wang**;
Centers for Disease Control and Prevention, Atlanta, GA, USA.

**Background:** In China, varicella vaccine is licensed for use on a private basis in children ≥12 months with a single dose recommended. Varicella became a notifiable disease in Beijing in Dec 2006 and thereafter, Beijing local health departments have provided free outbreak control vaccination to susceptible students in school settings. We used surveillance data to describe the epidemiology of varicella and uptake of varicella vaccine in Beijing during 2007-2010. **Methods:** Clinical practitioners report varicella cases of any age and doses of varicella vaccine administered to children. Standard demographic and limited clinical information is reported on every case; vaccination status is not collected. Varicella outbreaks in school settings are investigated by local health departments. Doses of varicella vaccine administered for outbreak control are recorded. Varicella vaccine coverage was estimated for each year for birth cohorts from 2004 to 2008 using the number of children in the immunization registry by birth year as the denominator without adjustment for history of varicella. **Results:** During 2007-2010, 15,544 - 18,256 varicella cases were reported annually with stable total incidence (range 0.98 - 1.05 per 1,000 persons). Twenty-two percent of the cases were < 5 years, 42% were 5-14 years and 36% were ≥ 15 years. The proportion of student cases decreased significantly from 73% in 2007 to 66% in 2010 and the proportion of adult cases increased significantly from 17% in 2007 to 23% in 2010. Varicella had a seasonal pattern with two peaks and troughs in incidence each year. Vaccine coverage at 2 years of age increased from 62% in 2006 to 74% in 2010, and was 80% in children aged 3-6 years in 2010. A total of 303 outbreaks with 3,845 related cases were reported from 2007 to 2010. Approximately 68% of outbreaks occurred in elementary schools. Cases in kindergartens and elementary schools were more likely to have occurred in vaccinated students than were cases in middle/high schools (66% vs. 27%, P<0.001). **Conclusions:** Moderately high one-dose vaccine coverage in young children has been achieved in Beijing with private vaccine purchase. More than a third of cases occur among older adolescents and adults. These data may help inform vaccine policy and other potential measures for varicella prevention and control in Beijing.

**Characteristics of Severe Pertussis Infections among Infants ≤90 Days of Age Admitted to Pediatric Intensive Care Units, Southern California, September 2009–June 2011**

**E.L. Murray**[1], D. Nieves[2], J.S. Bradley[3], W.H. Mason[4], D. Lehman[5], R. Harrison[6], K. Harriman[1], J.D. Cherry[6]; [1]Immunization Branch, California Department of Public Health, Richmond, CA, USA, [2]Children's Hospital of Orange County, Orange, CA, USA, [3]Rady Childen's Hospital, San Diego, CA, USA, [4]Children's Hospital of Los Angeles, Los Angeles, CA, USA, [5]Cedars-Sinai Medical Center, Los Angeles, CA, USA, [6]Mattel Children's Hospital, University of California, Los Angeles, Los Angeles, CA, USA.

**Background:** Pertussis, an acute respiratory disease caused by the bacteria *Bordetella pertussis,* can cause severe illness and death among infants. During the 2010 pertussis epidemic in California, 10 infants died due to pertussis. Data on the most effective treatment of critically ill infants is needed. **Methods:** We collected demographic and clinical information on infants ≤90 days of age who were admitted or transferred to five participating Southern California pediatric intensive care units (PICU) with *B. pertussis* infections from September 1, 2009 - June 30, 2011. Infants who were diagnosed with pulmonary hypertension or died were considered to have more severe pertussis infections. Student's t-tests and Fischer's exact tests were used to compare more severe with less severe infections. **Results:** Thirty-one infants were admitted to a participating PICU during the study period. The mean age at illness onset was 39 days, 55% were female and 87% were Hispanic; 8 had more severe infections, of whom 7 had pulmonary hypertension and 4 died. Infants with more severe infections were demographically similar to infants with less severe infections, and no significant differences in the time from illness onset to first presentation for medical care were identified. Compared to infants with less severe infections, infants with more severe infections had higher peak white blood cell counts (WBC), 72.8 vs. 26.3 x $10^3/mm^3$ (p <0.01) and their WBC exceeded 30.0 more rapidly after illness onset, 5.1 vs. 14.6 days (p <0.01). Additionally, infants with more severe infections were more likely than infants with less severe

infections to have a 50% increase in WBC in ≤24 hours, (50%; 0%, $p = 0.01$), receive an exchange transfusion (75%; 0%; $p < 0.01$), be diagnosed with pneumonia (100%; 43%; $p = 0.01$) and be intubated (75%; 9%; $p < 0.01$). **Conclusions:** Infants with more severe pertussis infections were more likely to have higher WBC and more rapid increases in their WBC than infants with less severe infections. Identifying these infants early during the course of their illnesses may allow for more rapid implementation of interventions like exchange transfusion that could potentially reduce the severity of disease and prevent death. Assessing WBC early in the course of illness may identify infants at greatest risk of severe disease.

## E3. H1N1 Influenza

Monday, March 12
3:15 PM – 4:45 PM
Centennial III

**Oseltamivir Inhibiting Viral Release but Enhancing Apoptosis of Influenza A H1N1-Infected Cells**

**R. Gao**, N. Du, Z. Li, J. Zhou, Y. Zhu, Y. Shu;
National Institute for Viral Diseases Control and Prevention, China CDC, Beijing, China.

**Background:** Influenza virus type A (and type B) causes recurrent epidemics every year, leading to significant human morbidity and mortality. Prompt antiviral drug treatment and chemoprophylaxis are recommended for pandemic or to-be pandemic influenza virus infection when specific vaccine is unavailable. Current options of therapy with oseltamivir for influenza still have arguments though antiviral effectiveness of oseltamivir has been approved in previous studies. **Methods:** Influenza A viruses (seasonal H1N1, 2009 pH1N1 and H5N1) were inoculated to infected THP-1, A549 or primary NK cells. Apoptosis, cytokines reponse or viral load was tested on both oseltamivir-treated and non-treated cells. Additionally, NKp46 and CD107a expression in priamary NK cells were tested. The oseltamivir-resistant H1N1 virus strains were used for a control in this study. **Results:** Our results showed that oseltamivir enhance apoptosis of infected A-549, THP-1 or primary NK cells by 2009 pH1N1 as well as seasonal H1N1 and highly pathogenic avian influenza H5N1 virus though cytokines or chemokines level decreased in the infected cells with oseltamivir treatment. Quantitative PCR suggested that viral gene copies were much higher in oseltamivir-treated cells than non-treated cells at 6h or 24h post of infection (POI) though viral gene copies is lower in oseltamivir-treated cells than in non-treated cells at 1h of POI. Furthermore, NKp46 in $CD56^{dim}$ NK cells increased in infected NK cells, and is lower in oseltamivir-treated NK cell than non-treated cells. Additionally, CD107a, a membrane protein which is a marker of NK cell activity, decreased in seasonal H1N1 infected NK cells with oseltamivir-treatment, but increased in 2009 pH1N1 infected NK cells with oseltamivir-treatment. **Conclusions:** The present study suggested that oseltamivir can induce apoptosis of infected-cells though it is effective on H1N1 infection by inhibiting viral entrance of viral release. Oseltamivir induce polarization of NK cells towards cytotoxicity after 2009 pH1N1 infection. Thus, oseltamivir application could result into a minus effect on the influenza patient. The clinical appropriateness of antiviral drugs should, therefore, be validated for different patient or in different illness stage.

**Decreased Serologic Responses among Vaccinated Military Recruits during 2011 Correspond to Genetic Drift in Concurrent Circulating Pandemic A/H1N1 Viruses**

**P.J. Blair**[1], D.J. Faix[1], A.W. Hawksworth[1], C.A. Myers[1], C.J. Hansen[1], R. Ortiguerra[1], L. Pacha[2], S.M. Garcia[3], A.A. Eick[4], S. Khurana[5], H. Golding[5];
[1]Naval Health Research Center, San Diego, CA, USA, [2]US Coast Guard Headquarters, Washington, DC, USA, [3]Marine Corps Recruit Depot, Parris Island, SC, USA, [4]Armed Forces Health Surveillance Center,

Silver Spring, MD, USA, [5]Food and Drug Administration, Department of Health and Human Services, Bethesda, MD, USA.
**Background:** Population-based Febrile Respiratory Illness (FRI) surveillance is conducted by the Department of Defense (DoD) at military recruit sites. Information gathered from influenza-vaccinated recruits contributes to an estimate of vaccine effectiveness. Between January and March 2011, 64 cases of laboratory confirmed 2009 A/H1N1 (pH1N1) infections, including one fatality, occurred in a representatively sampled group of immunized recruits at Fort Jackson, South Carolina implying poor efficacy for the 2009 pH1N1 component of the Live Attenuated Influenza Vaccine (LAIV). **Methods:** To test for serologic protection, serum samples were drawn from vaccinated recruits at Fort Jackson (LAIV), Paris Island (LAIV and Trivalent Inactivated Vaccine (TIV)) and Cape May, New Jersey (TIV). Responses in sera drawn 4-5 weeks post-vaccination were measured against pre-vaccination sera. A subset of 78 LAIV and 64 TIV sera pairs from recruits who reported no prior influenza vaccination or fever during recruit training were tested by microneutralization (MN) and hemagglutination-inhibition (HAI) assays. **Results:** Results demonstrated that MN responses to LAIV were significantly lower than those to TIV (p=). Additionally, the fold change in MN titer associated with TIV vaccination was significantly different between 2011 and 2009 pH1N1 viruses (ANOVA p-value = 0.0006). HAI analyses revealed similar trends. In naïve individuals, affinity for TIV-generated *vs.* LAIV-generated antibodies was greater in the quantity and quality for the high affinity anti-HA binding responses. **Conclusions:** These data demonstrated that among the recruits in this study in 2011, both TIV and LAIV vaccine type and pH1N1 virus year (2009 *vs.* 2011) influenced seroprotection. Finally, sequence analysis of the HA1 gene in concurrent circulating 2011 pH1N1 isolates from Fort Jackson exhibited modest (6-8 amino acid differences) divergence from the vaccine strain. Viruses from Fort Jackson in 2011 branched as sub-variants in clade IV. We hypothesize that antigen drift in pH1N1 viruses contributed to reduced vaccine effectiveness at Fort Jackson in 2011. Our findings have wider implications regarding vaccine protection from circulating pH1N1 viruses in the 2011-2012 influenza seasons.

**Hospitalized Patients with Influenza Viruses in Thailand during the 2009 Pandemic**

**S.J. Olsen**[1,2], S. Thamthitiwat[1], M. Chittaganpitch[3], P. Prapasiri[1], S. Naorat[1], W. Sanasuttipun[4], S. Chantra[5], S.A. Maloney[1,2], H.C. Baggett[1,2];
[1]Thailand MOPH - U.S. Centers for Disease Control and Prevention Collaboration, Nonthaburi, Thailand, [2]Centers for Disease Control and Prevention, Atlanta, GA, USA, [3]Ministry of Public Health, Nonthaburi, Thailand, [4]Provincial Hospital, Nakhon Phanom, Thailand, [5]Provincial Hospital, Sa Kaeo, Thailand.
**Background:** The 2009 pandemic influenza A (H1N1) virus (pH1N1) was first identified in Thailand in May 2009. We assessed the contribution of pH1N1 to respiratory hospitalizations and compared characteristics of influenza test positive and test negative patients hospitalized with respiratory illness during the pandemic. **Methods:** In two rural provinces conducting ongoing surveillance for hospitalized respiratory tract illness, nurses identified cases by a) the surveillance case definition (fever/abnormal WBC and a respiratory sign/symptom) or b) clinical suspicion of influenza. They used a standardized questionnaire to assess clinical illness, underlying disease, treatment and hospital course. Patients were tested for influenza viruses by rapid antigen test on a nasal swab and/or real-time RT-PCR on a nasopharyngeal swab. **Results:** Between Oct 2009-Aug 2010, 1,178 patients were identified at 20 hospitals. Of those, 537 were tested for influenza viruses and 93 (17%) were positive [75 had influenza A (36 pH1N1, 19 H3N2, 8 H1N1, 11 not subtyped, and 1 unsubtypeable) and 18 were influenza B]. The median age in persons with A (H3N2) was 57 years, with A (pH1N1) was 26 and with influenza B was 20 ($p<0.01$). Overall, 26 (28%) had $\geq$1 underlying medical condition, including one pregnant patient with influenza B. Severe outcomes were uncommon; one patient required mechanical ventilation and only one patient with influenza A died in hospital. Compared to 444 influenza test negative patients, influenza-infected persons were similar in percent male (51% vs. 46%), median age (24 vs. 23 years),

requirement for mechanical ventilation (1% vs. 1%), mortality (1% vs. 1%), reported vaccination (3% vs. 7%) and receipt of antivirals within 2 days of onset (42% vs. 49%), but they were significantly less likely to have underlying diseases [26 (28%) vs. 188 (42%), p=0.01] and the difference was present at all ages. **Conclusions:** Influenza viruses were found in 17% of patients hospitalized with respiratory illness but all subtypes were found. Influenza patients were difficult to distinguish from other respiratory patients. Recognized underlying disease was less common in influenza positive than negative patients. Influenza vaccination use was low but rapid use of antivirals was common and may have contributed to good outcomes.

**Using the Timely 122 Cities Mortality Reporting System Data to Detect Shifts in Mortality toward Younger Age Groups during 2009 H1N1 Pandemics**

**P. Cheng**, H. Zhou, L. Brammer, D. Shay;
Centers for Disease Control and Prevention, Atlanta, GA, USA.

**Background:** During influenza pandemics, mortality may be greatest among younger populations, while during seasonal epidemics, older populations are at greatest risk of influenza-associated mortality. These results were obtained using final mortality vital records data. We analyzed 122 Cities Mortality Reporting System (122 CMRS) data stratified by age group from 1962 through 2010 to see if shifts in mortality patterns could be detected with this more timely data system. We compared the proportions of influenza-coded deaths occurring among three age groups (0-24 years, 25-64 years, and 65+ years) during the study period. We also compared influenza-associated pneumonia and influenza (P&I) deaths estimated by using modeling techniques for the same time period. **Methods:** Weekly numbers of P&I deaths by age group from the 122 CMRS data for the years 1962-2010 were extracted for data analysis. The 2009 H1N1 pandemic, two recent severe H3N2 epidemics (1999/2000 and 2003/2004 seasons), and the 1968 pandemic seasons were specifically selected for comparison. We applied negative binomial models to P&I deaths for estimating influenza-associated deaths with influenza-coded deaths as an indicator of influenza activity. The proportions of influenza-coded deaths and the modeled influenza-associated deaths among three age groups were compared. **Results:** Sixty-four percent of influenza-coded deaths during the 2009 pandemic occurred among those aged 25-64 years. In contrast, only 12% of influenza-coded deaths during severe H3N2 epidemics and 29% of influenza-coded deaths during the 1968 pandemic occurred in this age group. When comparing influenza-associated deaths estimated by using negative binomial regression models, 48% occurred among those aged 25-64 years during the 2009 pandemic. In contrast, only 10% of influenza-associated deaths during severe H3N2 epidemics and 31% of influenza-associated deaths during the 1968 pandemic occurred in this age group. **Conclusions:** The proportions of influenza-coded deaths and the modeled influenza-associated deaths by age group for 2009 H1N1 pandemic and the 1968 pandemic were unusual. 122 CMRS data can detect shifts in influenza mortality patterns during a pandemic, and are available with a 1-week delay, compared with a 2- to 3-year lag in availability of final mortality data.

**Effectiveness of Repeated Annual Influenza Vaccination**

**A.C. Bateman**[1], B.A. Kieke[1], S.A. Irving[1], J.K. Meece[2], D.K. Shay[3], E.A. Belongia[1];
[1]Epidemiology Research Center, Marshfield Clinic Research Foundation, Marshfield, WI, USA, [2]Core Laboratory, Marshfield Clinic Research Foundation, Marshfield, WI, USA, [3]Centers for Disease Control and Prevention, Atlanta, GA, USA.

**Background:** The objective of this study was to assess the independent and combined effects of monovalent 2009 A(H1N1) vaccine and 2010-11 trivalent inactivated vaccine for the prevention of influenza infection during the 2010-11 season. **Methods:** Patients in a population cohort with medically attended acute respiratory illness <8 days in duration were prospectively recruited over 10 weeks (January-March 2011) and tested for influenza by real-time reverse transcription PCR (rRT-PCR).

Eligibility criteria included feverishness or cough. Case-control analyses were performed using data from patients with rRT-PCR confirmed influenza (cases) and ill patients without influenza (test-negative controls). A validated immunization registry was used to assess receipt of seasonal and pandemic vaccines in 2009-10 and 2010-11. Logistic regression analysis was performed to estimate vaccine effectiveness (VE) in 2010-11 for different combinations of monovalent 2009 A(H1N1) vaccine and 2010-11 trivalent inactivated vaccine (TIV). VE was calculated as 100 x (1- adjusted odds ratio). Models were adjusted for gender, age, week of enrollment, presence of a high-risk medical condition, and receipt of seasonal 2009-10 vaccine. **Results:** The effectiveness of 2010-11 TIV alone was 65% (95% CI, 45%-78%). Receipt of either inactivated or live-attenuated 2009 A(H1N1) vaccine alone did not protect against medically attended influenza in the 2010-11 season (adjusted VE of -10% and 1%, respectively). The combination of inactivated 2009 A(H1N1) and 2010-11 TIV showed similar protection to 2010-11 TIV alone (VE=57%; 95% CI, 24%-75%). The effectiveness of vaccination with both live-attenuated 2009 A(H1N1) and 2010-11 TIV was 85% (95% CI, 38%-97%). Receipt of both vaccines did not yield significantly greater effectiveness than receipt of TIV alone (p=0.29). **Conclusions**: Influenza vaccine protection against pandemic 2009 A(H1N1) waned over one season. The VE point estimate for vaccination with both live-attenuated 2009 A(H1N1) and 2010-11 TIV was higher than vaccination with other vaccines alone or in combination, but study power was limited and the difference was not significant.

**Estimation of the Mortality Burden of the 2009 Influenza A/H1N1 Pandemic in the USA, Based on State Inpatient Deaths: An Alternative to Vital Statistics**

**V. Charu**[1], L. Simonsen[1,2], R. Lustig[3], R. Jordan[4], C. Steiner[4], C. Viboud[1];
[1]National Institutes of Health, Bethesda, MD, USA, [2]George Washington University and Sage Analytica, Washington, DC, USA, [3]Sage Analytica, Bethesda, MD, USA, [4]Agency for Healthcare Research and Quality, Rockville, MD, USA.

**Background:** The mortality impact of the 2009 influenza A/H1N1 pandemic remains controversial; only preliminary estimates are available for the US due to reporting delays in vital statistics. Here we propose an approach to estimate influenza-related mortality rates using inpatient deaths from administrative hospitalization databases, which are currently available ~two years prior to release of vital statistics. **Methods:** We compiled weekly pneumonia and influenza (P&I) inpatient deaths from 9 State Inpatient Databases maintained by the Healthcare Cost and Utilization Project, representing ~30% of the US population, 1994-2009. We applied Serfling regression models to estimate influenza-related seasonal excess P&I deaths by age (< and >=65 years). Calibration of these estimates against traditionnal US national vital statistics, 1994-2007, allowed for extrapolation of the pandemic mortality burden during Apr-Dec 2009. **Results:** Age-specific seasonal excess P&I mortality estimates derived from the inpatient mortality databases were highly correlated with those derived from vital statistics data ($R^2$ range 0.7-0.9). We estimate that the 2009 A/H1N1 pandemic was associated with 4,600 excess P&I deaths and 44,000 excess all-cause deaths in the US, a death toll similar to the average seasonal influenza experience. However, 53% of pandemic-related deaths occurred in people under 65 years of age, compared to only 16% during seasonal epidemics (P<0.01). **Conclusions:** Our estimates suggest that the pandemic was associated with a high mortality burden in individuals under 65 yrs, relative to their experience with seasonal influenza. Influenza-related excess mortality estimates in individuals over 65 yrs may lack precision and sensitivity analyses including data from more states are planned. Our new inpatient mortality approach provides more timely estimates of influenza-related mortality rates, well in advance of the release of US vital statistics data. This approach may be applicable to monitoring mortality trends in other diseases, with the State Inpatient Databases now covering 44 US states.

## E4. Novel Surveillance Systems

Monday, March 12
3:15 PM – 4:45 PM
Centennial IV

### A Novel Approach to Measure Contribution of Viral Hepatitis in Chronic Liver Disease Using Ambulatory Care Data

**O. Utuama**, H. Roberts, M. Klevens, E. Teshale, E. Hughes, R. Jiles;
Centers for Disease Control and Prevention, Atlanta, GA, USA.

**Background:** Chronic liver diseases (CLD) are a major cause of morbidity and mortality in the United States. Several studies confined to small geographic areas have suggested that hepatitis B and C infections contribute to the development of cirrhosis and primary liver cancer in as many as 50% of cases. Of key interest therefore are the distribution of hepatitis B, hepatitis C and other etiologies of CLD in the larger population. **Methods:** To identify cases of CLD, we applied an algorithm developed by Bell to 2007 National Ambulatory Medical Care and National Hospitals Ambulatory Medical Care data. These surveys use a complex multi-stage probability sampling technique to generate an encounter-based, representative sample of U.S. ambulatory care utilization. In 2007, this sample consisted of 102,741 visits to physician offices, emergency or outpatient departments. A weighted analysis of frequencies and percentages of CLD visits was conducted to estimate the burden of CLD attributable to viral hepatitis. **Results:** An unweighted total of 788 potential CLD visits and 22 CLD etiologies were identified, representing an estimated 6,738,463 (95% CI=5,264,357; 8,212,569) CLD-related visits in 2007. Whites, females and patients aged 55 years and older comprised 73.0%, 52.6% and 44.8% of such visits, respectively. Diagnoses of hepatitis B, hepatitis C and hepatobiliary cirrhosis accounted for 6.8%, 3.6% and 10.2%, respectively of CLD visits. Of visits by patients with hepatobiliary cirrhosis or primary hepatocellular carcinoma, 7.0% were associated with viral hepatitis. **Conclusions:** In our study, hepatitis B and C accounted for 10.4% of CLD visits in 2007, representing an estimated 700,188 visits. Data from this geographically broader population support findings from small-scale studies that viral hepatitis is an important contributor to cirrhosis and primary hepatocellular carcinoma. The use of ambulatory care visit data is likely to improve the understanding of the role of viral hepatitis in CLD in the U.S.

### Application of an Automated Scoring System for Integrating Multiple Surveillance Systems, United States, 2009

**L. Richardson**[1,2], A. Krueger[1], J. Reynolds[1,2], M. Patrick[1], K. Swanson[1,2], K. Heiman[1,2], D. Cole[1];
[1]Centers for Disease Control and Prevention, Atlanta, GA, USA, [2]Atlanta Research and Education Foundation, Atlanta, GA, USA.

**Background:** National foodborne disease surveillance consists of multiple data collection systems. Isolate-based systems collect data on individual isolates, such as pulsed-field gel electrophoresis patterns, antimicrobial resistance phenotypes, and patient demographics. Outbreak surveillance collects aggregate data on foods, etiologies, and outbreak settings. Food safety policy-makers and stakeholders need integrated information for decision making. Historically, linking databases relied solely on a state public health laboratory identification number (SlabsID). Records with missing or incorrectly-formatted IDs could not be matched. We introduce an automated process for linking multiple surveillance systems using additional epidemiological variables. **Methods:** *Salmonella* serotype Newport records from four surveillance systems were matched using SlabsID, date of birth, specimen collection date, serotype, age, gender, county, and specimen source. Variables were ranked and assigned points based on the likelihood of a match: SlabsID received the highest rank and point value and non-specific variables (e.g. age, sex) received lower points. A linking score was calculated for "candidate" matches by dividing the

points received for each variable by the total points possible. The candidate with the highest linking score was considered the most likely match and was confirmed or rejected by inspection. The proportion of matched records was calculated by dividing the number of matched records by the number of records. Tests for statistical significance were performed using exact methods. **Results:** The proportion of matched records was significantly higher using the automated scoring system compared to using only SlabsID (59% versus 42%; p<.0001). Inconsistent formatting of the SlabsID was the primary reason for "missed" matches. Identical values among other epidemiological variables created additional links between the databases. **Conclusions:** The use of an automated scoring system significantly increases the number of records contained in an integrated database and should be considered when linking data from multiple sources. Linked datasets are a powerful tool for public health, as they provide comprehensive data for in-depth analysis of preventable diseases.

**Are Smart Phones Better than Paper-Based Questionnaires for Surveillance Data Collection? A Comparative Evaluation Using Influenza Sentinel Surveillance Sites in Kenya, 2011**

**H.N. Njuguna**[1], C. Deborah[1], G. Emukule[1], D. Kinyanjui[2], C. Kinkade[3], J.A. Mott[1], M. Katz[1];
[1]Centers for Disease Control, Nairobi, Kenya, [2]Kenya Medical Research Institute/Centers for Disease Control, Nairobi, Kenya, [3]Centers for Disease Control and Prevention, Atlanta, GA, USA.

**Background:** Manual data collection and data entry using paper-based questionnaires can be time consuming and prone to errors. We introduced smartphones in 4 hospital-based influenza sentinel surveillance sites in Kenya. We compared smartphone-collected data to paper-based-collected data previously collected by the same surveillance officer. **Methods:** Since 2006, the Kenya Ministry of Health and the Kenya Medical Research Institute/Centers for Disease Control have conducted sentinel influenza surveillance at 11 hospitals in Kenya. At each site, surveillance officers identify patients with respiratory illness and administer a brief (18 question) questionnaire that includes demographic and clinical information. From May - June 2011, we pilot-tested an electronic data collection system using Field Adapted Survey Toolkit (FAST) on HTC Touch Pro2 smartphones at four sentinel sites. For each site, we compared questionnaires collected using smartphones to an equal number of paper-based questionnaires collected by the same surveillance officer. **Results:** A total of 1,019 paper-based questionnaires were collected at the four sites from Dec 14, 2010- June 6, 2011 and 1,019 smartphone questionnaires were collected at the same four sites from May 3, 2011-Aug 26, 2011. In all, 5% of paper-based questionnaires were determined to be incomplete compared to 3% of smartphone questionnaires. Seven paper-based questionnaires had duplicated patient identification numbers, while no duplication was seen in smartphone data. Smartphone data were uploaded into the database within 8 hours of collection, and paper-based data took an average of 24 days to be uploaded. For one year, paper-based data collection costs approximately 16,800 USD compared to approximately 14,500 USD for smartphone data collection. All surveillance officers reported that smartphones were much easier, faster, and more convenient to use as data collection tools. **Conclusions:** At four influenza surveillance sites, electronically collected data were subject to fewer errors and were quickly available for data analysis. Operating costs were less for smartphones when compared to paper-based data collection. Electronic data collection using smartphones has the potential to improve data integrity and reduce costs.

**Evaluation of a Collaborative Program with Traditional Healers to Expand Surveillance for Plague in Northern Uganda**

**E.C. Zielinski-Gutierrez**[1], T. Apangu[2], M. Hayden[3], A. Monaghan[3], C. Black[4], K. Cavanaugh[5], P. Mead[1], K. Griffith[1];
[1]Centers for Disease Control and Prevention, Fort Collins, CO, USA, [2]Uganda Virus Research Institute,

Arua, Uganda, [3]National Center for Atmospheric Research, Boulder, CO, USA, [4]University of Colorado-Denver, Denver, CO, USA, [5]Colorado College, Colorado Springs, CO, USA.
**Background:** Plague, caused by the bacterium *Yersinia pestis*¸ is an important cause of morbidity and mortality in the West Nile region of Uganda. Plague needs to be treated with appropriate antibiotics to reduce mortality. Estimates for Uganda suggest that up to 60% of the population use traditional medicine (WHO 2002). In this overwhelmingly rural, upland area with limited resources, traditional healers are an important component of local health care. This may contribute to inadequate treatment, underreporting of plague cases, and occupational risk for healers. **Methods:** Working with staff from the Uganda Virus Research Institute and Uganda Ministry of Health, CDC undertook a qualitative assessment with a sample of traditional healers in 2009. Eleven healers from two districts in the West Nile region were interviewed; their practices varied and included herbal treatment, healing using spirits and witchcraft. A pilot referral network was put into place in 2010 with 10 traditional healers, and an evaluation of the program was undertaken in 2011, one year after the inception of the program. **Results:** The assessment found that healers were aware of plague in their area and most indicated that they see patients with symptoms that could fit the description of plague. While most reported referring suspected plague patients to the local clinic, many also described administering "first aid" for hours to days before referral. There was strong willingness to participate in training about plague and to engage in referral. After the initial assessment, 10 healers were trained through individual visits and introduced to local clinic staff who conducted monthly follow-up visits. Traditional healers were provided with key tools for referral: a bicycle, referral cards and a cell phone programmed with airtime and clinic contacts. There was no confirmed plague activity in the region in 2010, however monthly tracking and an evaluation showed that the traditional healers did refer >150 patients to health centers, many with other serious illnesses. The majority of referral tools were still in place. Most healers requested additional training and expansion of the program to other local healers. **Conclusions:** Recommendations for the future of this program, and implications for non-traditional rural surveillance efforts, will be presented.

**Enhanced Surveillance for Fatal Dengue-like Acute Febrile Illness – Puerto Rico, 2010**

**K.M. Tomashek**[1], A. Rivera[1], I. Rivera[2], D. Blau[3], J. Munoz-Jordan[1], E. Hunsperger[1], J. Bhatnagar[3], C. Drew[3], C. Paddock[3], W.-J. Shieh[3], L. Alvarez[2], C. Chavez[2], F. Cortes[2], F. Davila[2], E. Rodriguez[2], R. Rodriguez[2], D. Sanabria[2], J. Serrano[2], J. Torres[2], R. Galloway[3], S.R. Zaki[3], H. Margolis[1];
[1]Centers for Disease Control and Prevention, San Juan, PR, USA, [2]Institute of Forensics Sciences, San Juan, PR, USA, [3]Centers for Disease Control and Prevention, Atlanta, GA, USA.
**Background:** Dengue is a major public health problem, with increasing global incidence and severity. Recent fatal case reviews from Puerto Rico suggest that dengue mortality is under recognized and underreported. We conducted enhanced surveillance for deaths following acute febrile illness (AFI) to identify dengue deaths. **Methods:** Identification of fatal AFI cases began in 2010, with specimens collected by hospital and forensic pathologists. Serum was tested for dengue virus (DENV) RNA by reverse transcriptase polymerase chain reaction (RT-PCR), and anti-DENV IgM and IgG by enzyme-linked immunosorbent assay (ELISA); tissue was tested for DENV RNA and antigens by RT-PCR and immunohistochemistry (IHC). A laboratory-positive dengue case was serum or tissue RT-PCR positive or anti-DENV IgM ELISA positive. A laboratory-negative case was IgM ELISA negative in serum collected >5 days after fever onset, tissue RT-PCR negative, or RT-PCR negative in serum taken ≤5 days after illness onset. A laboratory-indeterminate case was DENV RNA or anti-DENV IgM negative in serum collected ≤5 days after illness onset and no convalescent serum specimen and tissue available for testing. Medical records from cases are being abstracted using a standard form. **Results:** Among 122 fatal AFI cases detected, 39 (32%) were dengue laboratory-positive, 61 (50%) laboratory-negative, 21 (17%) indeterminate and one was not processed. The majority (77, 63%) had an autopsy performed and 18

(15%) had paired serum samples. Half (19, 49%) of laboratory-positive cases had positive tissue RT-PCR, 17 (44%) had positive serum RT-PCR, and 3 (8%) were anti-DENV IgM positive. Twelve of the 19 positive tissue RT-PCR cases were IHC positive. Of 36 RT-PCR-positive specimens, DENV-1 (58%), DENV-4 (28%) and DENV-2 (14%) were detected. The median age of laboratory-positive cases was 45 years (range 0.4 months to 88 years); 35 (90%) were ≥20 years old and 16 (46%) were male. A disproportion number of laboratory positive fatal cases in 2010 were residents of the less populated health regions including Aquadilla and Arecibo. **Conclusions:** Autopsy rates doubled under the new system and the proportion of indeterminate diagnoses was cut in half. Enhanced surveillance detected more laboratory confirmed dengue deaths than in the 1998 and 2007 epidemics combined.

**A Surveillance Sector Review: A Novel Method Used to Review All of the Infectious Disease Surveillance Systems Operating in a Single Country**

**M.G. Baker**, S. Easther, N. Wilson;
University of Otago, Wellington, New Zealand.

**Background:** The new International Health Regulations (IHR) require World Health Organization (WHO) member states to assess their core capacity for surveillance. Such reviews also have the potential to identify important surveillance gaps, improve the organisation of disparate surveillance systems and to focus attention on upstream hazards, determinants, and interventions. **Methods:** We developed a surveillance sector review method for evaluating all of the surveillance systems and related activities across a sector, in this case those concerned with infectious diseases in New Zealand. The first stage was a systematic description of these surveillance systems using a newly developed framework and classification system. Key informant interviews were conducted to validate the available information on the systems identified. **Results:** We identified 91 surveillance systems and related activities in the 12 coherent categories of infectious diseases examined. The majority (n = 40 or 44%) of these were disease surveillance systems. They covered all categories, particularly for more severe outcomes including those resulting in death or hospitalizations. Except for some notifiable diseases and influenza, surveillance of less severe, but important infectious diseases occurring in the community was largely absent. There were 31 systems (34%) for surveillance of upstream infectious disease hazards, including risk and protective factors. This area tended to have many potential gaps and lack integration, partly because such systems were operated by a range of different agencies, often outside the health sector. There were fewer surveillance systems for determinants, including population size and characteristics (n = 9), and interventions (n = 11). **Conclusions:** It was possible to create and populate a workable framework for describing all the infectious diseases surveillance systems and related activities in a single developed country and to identify potential surveillance sector gaps. This was the first stage in a review process that will lead to identification of priorities for surveillance sector development.

## E5. Antimicrobial Resistance

Monday, March 12
3:15 PM – 4:45 PM
Regency VI

**Pulsetypes and Antimicrobial Resistance Genes Associated with Multidrug-Resistant *Salmonella enterica* serovar Typhimurium from Humans in Guangdong, China**

**Z. Liang**[1], S. Wu[2], B. Ke[3], L. Ran[4], D. He[3], B. Li[3], X. Deng[3], C. Ke[3], M. Mikoleit[2], J.D. Klena[2];
[1]Sun Yat-Sen University, Guangzhou, China, [2]Centers for Disease Control and Prevention, Atlanta, GA,

USA, [3]Guangdong Provincial Center for Disease Control and Prevention, Guangzhou City, Guangdong Province, China, [4]Chinese Center for Disease Control and Prevention, Beijing, China.
**Background:** *Salmonella* is a significantly important foodborne pathogen globally. *Salmonella* Typhimurium (ST) and Enteritidis are the most common serovars in human infections worldwide. Multidrug-resistant (MDR) ST is of big public concern as it can increase clinical treatment failure. Salmonella Genomic Island 1 (SGI1) is a 43-kb chromosomal region encoding resistance to ACSSuT (A: ampicillin; C: chloramphenicol; S: streptomycin; Su: sulfamethoxazole; T: tetracycline). Due to the location of SGI1, antimicrobial resistance associated with this genetic element is stably maintained. This study was conducted to determine the prevalence of SGI1 in ST strains isolated in Guangdong Province in China. **Methods:** A total of 232 ST strains were collected from outpatients with diarrhea from 22 hospitals in Guangdong Province from September 2009 to July 2011. For antimicrobial susceptibility testing (AST), we selected relevant β-lactams, quinolone, and aminoglycosides as well as chloramphenicol, tetracycline and sulfanilamide to determine whether these isolates were MDR. DNA was extracted from isolates with the ACSSuT pattern and PCR detection of SGI1 performed. Pulsetypes, based on pulsed field gel electrophoresis patterns generated after XbaI-restriction enzyme digestion, was performed on the ACSSuT-resistant strains to determine relatedness. **Results:** According to AST, 217 isolates were resistant to 3 or more classes of antimicrobials, considered to be MDR. Among these isolates, 64.5% (140/217) had the ACSSuT resistant pattern. Nine strains (9/140; 6.43%) were positive for SGI1. Seven of the nine SGI1-positive isolates were from infants. PFGE analysis indicated there were two clusters in the 9 SGI1-positive STs; two of the isolates from infants had a pulsetype indistinguishable to that of a ST strain commonly associated with infant diarrhea in Guangdong province. Conclusions: SGI1 is emerging as a resistance mechanism in isolates of ST from Guangdong Province, potentially creating a serious public health challenge. Two of nine SGI1-positive strains shared a pulsetype that is common among ST in Guangdong province. Increased surveillance for MDR ST is necessary for Guangdong and other areas of China to track dissemination of these problematic strains.

**Fluconazole-Resistant *Candida parapsilosis*: An Emerging Agent of Fungemia in South Africa**

**N.P. Govender**[1,2], J. Patel[1], R. Kularatne[3,2], Y. Coovadia[4,5], S. Seetharam[3,2], N. Bosman[3,2], A. Whitelaw[6,7], A. Hoosen[8,9], A. Ahlquist[10], I. Zietsman[11], TRAC-South Africa Group;
[1]National Institute for Communicable Diseases, a Division of the National Health Laboratory Service, Johannesburg, South Africa, [2]University of the Witwatersrand, Johannesburg, South Africa, [3]National Health Laboratory Service, Johannesburg, South Africa, [4]National Health Laboratory Service, Durban, South Africa, [5]University of KwaZulu-Natal, Durban, South Africa, [6]National Health Laboratory Service, Cape Town, South Africa, [7]University of Cape Town, Cape Town, South Africa, [8]National Health Laboratory Service, Pretoria, South Africa, [9]University of Pretoria, Pretoria, South Africa, [10]Centers for Disease Control and Prevention, Atlanta, GA, USA, [11]Ampath National Laboratory Service, Johannesburg, South Africa.
**Background**: Although candidemia is a common and deadly hospital infection, few data are available from sub-Saharan Africa. **Methods**: From February 2009 through August 2010, sentinel, laboratory-based surveillance for candidemia was undertaken in South Africa at 11 public-sector hospitals and at private-sector hospitals served by 4 private laboratory groups. An incident episode was defined as the first blood culture isolation of *Candida* species from a patient admitted to a sentinel hospital. Incidence was calculated for 9 public-sector hospitals in 2009 using hospital admission denominators. Isolates and demographic data were submitted for each case. Isolates were identified to species-level and broth dilution susceptibility testing was performed for 9 antifungal drugs; proposed new CLSI breakpoints were used to interpret results. **Results**: Overall, 2,172 incident cases were detected, 773 (36%) by audit. The incidence of candidemia ranged from 8.1 to 21.2 cases per 10,000 admissions. Of 1,399 reported cases, 1,360 (97%) had a viable isolate. *Candida parapsilosis* (CP), the most common pathogen overall,

caused 572 (42%) cases. Over half of CP isolates (307/572; 54%) were fluconazole-resistant. The incidence of fluconazole-resistant CP disease ranged from 0.2 to 8.1 cases per 10,000 admissions. Only 29% (90/307) and 24% (74/307) of fluconazole-resistant isolates were susceptible to itraconazole and voriconazole. Compared with other cases of CP fungemia, cases of fluconazole-resistant CP were significantly more likely to be detected in Gauteng vs. any other province (271/422, 64% vs. 35/145, 24%; p<0.001) and in the private- vs. public-sector (148/220, 67% vs. 159/348, 46%; p<0.001). Neonates were not more likely to have fluconazole-resistant CP fungemia than any other age group (76/143, 53% vs. 211/390, 54%; p=0.84). **Conclusions**: The incidence of candidemia at South African hospitals is higher than reported incidence from developed countries. CP was the most common pathogen and remarkably, over half of these isolates were fluconazole-resistant, most with cross-resistance to other triazoles, limiting treatment options. Most of these cases were detected from hospitals in the most populous province and occurred across all age groups. Smouldering, undetected nosocomial outbreaks may have occurred.

**Antibiotic Prescription Practices for Acute Respiratory Infections in Minya District, Upper Egypt**
**M.M. Talaat**[1], T. Saeid[1], L. Hicks[2], W. El-Shoubary[1], M. Abdel Fattah[3], K. Dooling[2], M. Ginidy[3], O. Ragab[3], A. Kandeel[3];
[1]US Naval Medical Research Unit, No. 3, Cairo, Egypt, [2]Centers for Disease Control and Prevention, Atlanta, GA, USA, [3]Ministry of Health and Population, Cairo, Egypt.
**Background:** Excessive use of antibiotics has contributed to the emergence and spread of antibiotic-resistant bacteria. This is the first study in Egypt to describe the antibiotic prescription practices of physicians and pharmacists for acute respiratory infections (ARIs) in Minya district, Upper Egypt. **Methods:** All physicians treating ARI patients (internists, pediatricians, family doctors) in ambulatory clinics (n=254) as well as all pharmacists (n=567) in Minya district were invited to participate in a survey evaluating their antibiotic prescribing practices for ARIs (common cold, sinusitis, bronchitis, pneumonia). **Results:** Of professionals contacted, 236 physicians (93%) and 483 pharmacists (85%) practicing in public and private settings responded. Physicians saw a median of 55 patients per day (1-150) in the government clinics compared to 5 patients per day (1-50) in private clinics. Pharmacists saw a median of 50 patients per day in both government and private settings. The proportion of physicians who reported prescribing antibiotics most of the time or sometimes was 42.8% for common cold, 87.2% for sinusitis, 94.1% for bronchitis, and 91.6% for pneumonia. Beta-lactams were most frequently cited as the preferred antibiotic for all ARIs followed by macrolides (for colds and sinusitis) and quinolones (for bronchitis and pneumonia). In the month before the survey, 82.2% of physicians reported being asked by patients for an antibiotic when they felt one was not indicated. Among pharmacists (83.6%) reported prescribing antibiotics for patients requesting medical consultation, where 52.5% of pharmacists reported prescribing antibiotics most of the time or sometimes for common cold. Most pharmacists (59%) reported at least 3 patients per day requesting antibiotics without a prescription. Antibiotic selection was similar to physicians, however, pharmacists reported prescribing a shorter duration of treatment for colds (1-2d) (17.6% of pharmacists vs. 2.7% physicians). **Conclusions:** Antibiotic prescribing for ARIs by physicians and pharmacists in Minya district is excessive. Innovative intervention strategies are under development to reduce unnecessary antibiotic prescription for ARIs.

**Mupirocin Resistant HA-MRSA with Inducible Clindamycin Resistance Causing Skin Infections in a Tertiary Care Centre from Chennai, South India**
**N. Abimanyu**[1], S. M[1], B.S. Dass D[1], P. Krishnan[1], B. Sinha[2], S. G[3];
[1]Dr. ALM PG Institute of Basic Medical Sciences, University of Madras, Chennai, India, [2]Institute of Hygiene and Microbiology, University of Wuerzburg, Wuerzburg, Germany, [3]Madras Medical College and Govt. General Hospital, Chennai, India.

**Background:** Mupirocin is a topical antibiotic used for treating skin infections and eradicating nasal colonization by MRSA. Understanding the epidemiology of mupirocin resistance among *Staphylococcus aureus* is very important for proper use. Mupirocin resistant MRSA have been reported from various parts of the world. Clindamycin is another important antibiotic used for the treatment of skin and soft tissue infections caused by MRSA. Resistance to clindamycin has also been reported, there is increasing occurrence of inducible clindamycin resistance. Treatment failures of infections with inducible clindamycin resistance have been documented. Here, we report emergence of a novel mupirocin resistant HAMRSA clone with inducible clindamycin resistance causing skin infections in a tertiary care center in Chennai, South India. **Methods:** 94 *S. aureus* isolates acquired from clinical specimens from various skin infections were included in this study. All the isolates were screened for methicillin resistance by cefoxitin disc diffusion method. AST was done for various antibiotics by disc diffusion method. Mupirocin resistance was screened by mupirocin (5µg) disc diffusion test and detected by *ileS*2 gene PCR. Multiplex PCRs were done for detection of PVLMRSA and SCC*mec* typing. *spa* typing was done by the method of Keiswerth. The study was approved by the IEC. **Results:** 63/94(67%) isolates were found to be MRSA. All MRSA isolates were SCC*mec* typable, with type V (49.2%), type III (38.1%), type I (11.1%) and type IV (1.5%). 16/63 (25.4%) MRSA isolates were found to be mupirocin resistant. All mupirocin resistant MRSA isolates belong to the SCC*mec* type III (HAMRSA), *spa* type t037 and were positive for inducible clindamycin resistance. All mupirocin resistant isolates were MDR with sensitive only to vancomycin, fusidic acid and linezolid. Other major spa types obtained in this study were t064 (n=22), t657 (n=9) and t425 (n=5). 15/94(15.9%) *S. aureus* isolates were found to be positive for *pvl* by PCR of which 9 were found to be PVL-MRSA and had SCC*mec* type V and *spa* type t657. **Conclusions:** We report emergence of a novel multidrug resistant HAMRSA clone with mupirocin and inducible clindamycin resistance. High prevalence of MRSA (67%) in this study is already a big concern, the emergence of this kind of MDR-MRSA clone has to be addressed to prevent the increase in mortality and morbidity.

***Staphylococcus aureus* (SA) Colonization among Children with and without Skin and Soft Tissue Infections (SSTI)**

**K. Como-Sabetti**[1,2], W. Pomputius[2], B. Juni[1], G. Salo[1], G. Short[1], J. Mahon[1], R. Lynfield[1];
[1]Minnesota Department of Health, St. Paul, MN, USA, [2]Children's Hospitals and Clinics of Minnesota, Minneapolis, MN, USA.

**Background:** MRSA infections have increased over the past 15 years. The prevalence, sites and types of SA strains colonizing infected and non-infected children are not well defined. **Methods:** Children 6 mo-18 yrs treated at Minneapolis - St. Paul Children's Hospitals with a SSTI SA infection and controls (ER patients with injuries) had nasal, throat, and perirectal or groin swabs cultured. Cases culture positive for SA were defined as culture-confirmed (CC) or, if no positive culture, SSTI by clinical diagnosis. SA identification and PFGE were done at MN Dept. Health. **Results:** 69 cases and 228 controls were enrolled 11/09-8/11; 80% lacked healthcare risk factors for MRSA; median age was 6 yrs. 22 (63%) of CC cases had MRSA infection. Overall, 60% were colonized with SA; (69% CC, 53% SSTI, 60% controls); 26% of CC, 21% of SSTI cases and 4% of controls had MRSA colonization. MRSA colonization was more likely among cases (OR=6.58, $p<0.01$), SA colonization did not differ between cases and controls. Throat was the most likely site for SA colonization ($p<0.01$; 44% CC, 45% SSTI, 58% controls); 11% of cases had MRSA throat colonization. SA or MRSA throat colonization did not differ between cases and controls. Cases were more likely to have MRSA nasal colonization than controls (OR=14.82, $p<0.01$) and to have perirectal/groin SA and MRSA colonization (OR=2.64, $p<0.01$ and OR=20.24, $p<0.01$). 18 cases had a buttock/groin SSTI and perirectal/groin culture; they were more likely to have perirectal/groin colonization compared to other cases (67% vs. 24%; OR=6.25; $p<0.01$). 34% children had 1 positive site; 29% ≥ 2 sites (colonization or infection). Of these, 23/85 (27%) were colonized with different USA

groups. Four children had MRSA and MSSA. **Conclusions:** Colonization was frequent in this population of generally healthy children and most often with MSSA. MRSA colonization was more likely in cases than controls. The perirectal/groin sites were useful to detect colonization, especially in cases with buttock SSTI. About 25% of children with multiple sites of SA were colonized or infected with more than one SA strain, including a few who were dually colonized with MRSA and MSSA. Further insight on the role and impact of colonization is needed.

**Prevalence and Molecular Epidemiology of *Staphylococcus aureus* Colonization in a Cohort of Rural Iowans**

**T.C. Smith**, M. Quick, B. Hanson, B. Forshey, R. Nair, A. Kates, S. Farina, A. Opese, J. Wu, S. O'Malley, S. Wardyn;
University of Iowa, Iowa City, IA, USA.

**Background**: Livestock farmers are an emerging group at risk of colonization with methicillin-resistant *Staphylococcus aureus* (MRSA). In a previous study conducted by our group, almost half of swine workers and pigs sampled were found to be colonized with MRSA on farms in Iowa and Illinois. Livestock-associated MRSA has been documented in an increasing number of countries, although most studies have examined a relatively small number of farm workers in cross-sectional studies and have frequently been conducted on the farm site. A large-scale, longitudinal study of swine workers examining carriage of and infection with antibiotic-resistant *S. aureus* has not been conducted in the United States, but is of vital importance, as the epidemiology of MRSA is rapidly changing. **Methods**: We have launched a prospective study of rural Iowans, examining 1) individuals enrolled in the Agricultural Health Study, a long-term cohort study of pesticide applicators, focusing on those who raise swine and 2) a matched population-based group lacking exposure to livestock. In both of these groups, we will examine both colonization and infection with *S. aureus*. **Results**: To date, we have enrolled 1287 adults and 52 children in 32 counties throughout Iowa. *S. aureus* was isolated from 26.50% (341/1287) of adults and 26.92% (14/52) of minors. MRSA was found in 3.50% (45/1287) of adults. Three hundred eighteen of 410 total isolates have been tested for antibiotic susceptibility; 149 isolates showed no resistance to the panel of antibiotics tested, 105 showed resistance to two or more antibiotics, and 64 showed resistance to a single antibiotic. *spa* typing has been completed for 267/410 isolates. The most common type currently is *spa* type t002 (sequence type 5), a type which is common both in humans as well as swine and poultry. This was found in 34/267 (12.7%) of isolates tested. Sequence type 398-associated *spa* types (“livestock-associated *S. aureus*) were found in 36/267 (13.5%) of isolates. The most common of these was t034 (25/267, 9.4%), followed by t571 (8/267, 3.0%) and t011 (3/267, 1.1%). **Conclusions**: Livestock-associated types of *S. aureus* are common colonizers of Iowans in our cohort, including individuals lacking contact with livestock or poultry. This suggests the potential for human-to-human transmission or other transmission mechanisms.

## E6. Late-Breakers I

Monday, March 12
3:15 PM – 4:45 PM
Regency VII

**Wild Polio Virus Outbreaks in the Central African Republic and Other Polio-Free Countries at Risk of Importations, January 2010 to January 2012**

**I.U. Ogbuanu**[1], C. Lamoureux[2], S. Lowther[1], C. Burns[1], C. Wolff[2], P. Chenoweth[1], R. Tangermann[2], F. Mahoney[1], S. Wassilak[1];

[1]Centers for Disease Control and Prevention, Atlanta, GA, USA, [2]World Health Organization (WHO), Geneva, Switzerland.
**Background:** Despite an estimated 99% decrease in wild poliovirus (WPV) cases since 1988, sustained WPV transmission in endemic countries continues to lead to outbreaks in previously polio-free countries and jeopardizes the Global Polio Eradication Initiative (GPEI) goal. **Methods:** Using WHO surveillance data, we provide an overview of outbreaks reported during 2010-2011 and describe details of the most recent 2011 outbreak in the Central African Republic (CAR), a country at high risk for WPV importations because of proximity to polio-affected countries. **Results:** During 2010, 20 WPV outbreaks with 1056 total cases were reported in 13 countries (including 441 in Congo and 458 in Tajikistan). During 2011, 13 WPV outbreaks with 76 total cases were reported in 9 countries, including 48 cases in West Africa (Côte d'Ivoire, Guinea, Mali and Niger), 1 case in the Horn of Africa (Kenya) and 21 in China. Outbreak size was smaller in 2011 (median=1 case; range=1-36 cases) compared to 2010 (median=4 cases; range=1-458 cases). The most recent outbreak in 2011 was reported in CAR, with 4 cases to date near the northern border with Chad from 19 September to 8 December. After successful control of a focal outbreak of 14 cases in 2009, the following GPEI surveillance and vaccination indicators worsened each quarter from January 2010 up to the 2011 outbreak: the percent of 6-35 month-old children with non-polio acute flaccid paralysis (NP-AFP) with ≥4 doses declined from 27% to 20%; the percent of missed children in selected monitoring of vaccination campaigns increased from 10% to 18%; the proportion of prefectures meeting the NP-AFP standard of 2 reported cases per 100,000 among children aged <15 years declined from 100% to 83%; and the percent of adequate stool specimens collected from persons with AFP cases declined from 91% to 85%. **Conclusions:** The outbreak in CAR illustrates how ongoing gaps in GPEI surveillance and vaccination indicators characterize a country in which an outbreak was possible following a cross-border importation. Polio-free countries at risk of WPV importations, must maintain high population immunity through routine immunization and supplemental immunization activities, and maintain sensitive AFP surveillance systems promptly testing specimens in accredited laboratories to prevent and limit WPV outbreaks.

**Dog Vaccination and Campaign Management for Effective Rabies Control: The Bali Experience**

**P.P. Suseno**[1], L. Schoonman[2], P. Pudjiatmoko[1], I.P. Sumantra[3], I.W. Sukanadi[3], I.W. Mardiana[3], S.A. Crafter[2], E. Sawitri[2], A. Budiantono[1], A.S. Lubis[1], A.P. Dewi[2], E. Brum[2,4];
[1]Directorate of Animal Health, Directorate-General of Livestock and Animal Health Services, Ministry of Agriculture, Jakarta, Indonesia, [2]Food and Agriculture Organization of the United Nations, Jakarta, Indonesia, [3]Livestock and Animal Health Services, Province of Bali, Denpasar, Indonesia, [4]Tufts University Cummings School of Veterinary Medicine, North Grafton, MA, USA.
**Background:** Rabies was confirmed in southern Bali in late 2008. Initial dog vaccination and culling of unconfined dogs was not successful to control the disease and by June 2010 rabies has spread to all nine districts in Bali with 133 human deaths recorded to date. **Methods:** In 2010, the first island-wide dog vaccination campaign was implemented, followed by a second island-wide campaign in 2011. The strategy in both vaccination campaigns focused on achieving at least 70% vaccination coverage in each sub-village in Bali by using vaccination teams with specially-trained dog catchers. These teams enabled vaccination of the roaming dog population. The second vaccination campaign was planned and managed from the provincial level. A well-managed campaign implementation system was put in place with the following key components: 1) SMS reporting alongside a paper reporting system, 2) daily, weekly, and monthly coordination meetings, and 3) development of campaign-specific SOPs and in-service training of field staff. The number of dogs vaccinated and post-vaccination survey results were sent daily by the 93 vaccination teams via SMS to the provincial campaign management unit, which enabled this unit to monitor the daily vaccination coverage at sub-village level and to advise on re-vaccination if coverage was insufficient. The campaign management consisted of divisions for vaccination, rapid response and

surveillance, logistics, and communication both at provincial and district level. Key people in the campaign management and field staff were trained based on their responsibilities in the SOP such as dog catching and handling, vaccination, cold chain and logistic management, rapid response, communication, surveillance, and integrated human bite case management. **Results:** Using this well-managed and coordinated control strategy more than 234,000 dogs were vaccinated (estimated dog population coverage 71-84%) in 4373 sub-villages (100%) within a three month period. This simple strategy successfully reduced the human cases by 72% and animal cases by 79% in 2011 compared to 2010. **Conclusions:** This experience in Bali shows that a simple control strategy based on dog vaccination implemented with daily monitoring and management of vaccination activities can effectively reduce rabies in both animals and humans.

**Cost-Effectiveness of Intervention Strategies for Pandemic Influenza: The Role of Pandemic Severity**

**G. Milne**[1], N. Halder[1], M. Postma[2], J. Kelso[1];
[1]University of Western Australia, Crawley, Australia, [2]University of Groningen, Groningen, Netherlands.

**Background:** Obviously, the severity of an influenza pandemic directly influences its overall cost as high healthcare costs and productivity losses due to death arise from severe pandemics. Yet, economic analyses taking severity explicitly into account are scarce. For different levels of severity, we applied an individual-based simulation model of a community of 30k persons in Western Australia and combined this with an economic analysis to determine the cost of a range of mitigating interventions. **Methods:** Using recently obtained data from the 2009 H1N1 pandemic, and a 1 to 5 severity scale relating hospitalization and ICU rates to case fatality ratios (CFR), we determined which interventions are most cost-effective for a particular severity category. Social distancing and antiviral drug interventions were simulated under 3 transmission settings and 5 severity categories. **Results:** For low severity pandemics, as interventions increase in effectiveness (reducing the illness attack rate AR by combining interventions together or extending their durations) costs also increase. In contrast, for high severity pandemics, increasing intervention effectiveness decreases total costs. For a pandemic with a reproduction number of 1.8 (and no single intervention reduces AR by > 50%) a highly effective combined strategy of antiviral treatment, household prophylaxis and extended school closure reduces the AR from 32% to 9%. For a low severity pandemic (CFR 0.1%) this intervention costs $34,873 per Life Year Saved (LYS); for a high severity pandemic (CFR 0.75%) it costs $10,972 per LYS; for an extremely severe pandemic (CFR 2.5%) costs are $8,318 per LYS. Total pandemic costs of this strategy are $474, $1,089 and $2,734 per person, respectively. For low severity pandemics the overall costs are dominated by productivity losses due to illness and social distancing interventions; for high severity pandemics, costs are dominated by healthcare costs and costs arising from productivity losses due to death. **Conclusions:** Results indicate that interventions which include rigorous social distancing policies, considered unacceptable for low severity pandemics due to societal disruption, are necessary for high severity pandemics to substantially reduce the attack rate and are also the most cost-effective strategies available.

**Rapid Investigation of a Multistate Outbreak of Listeriosis Associated with Farm A Cantaloupes Using the *Listeria* Initiative—United States, August–October 2011**

**J. McCollum**[1,2], B. Silk[1], K. Jackson[1], K. O'Connor[1], M. Imanishi[1], A. Cronquist[2], S. Cosgrove[2], R. Vogt[3], T. Ghosh[3], N. Jain[1,3], M. Ibraheem[1,4], A. Gelfius[5], T. DuVernoy[5], C. Spires[5], S. Merriweather[5], P. Teitell[6], M. Freeman[1], L. Joseph[1], E. Trees[1], K. Neil[1], C. Tarr[1], B. Mahon[1], G. Wright[1], R. Tauxe[1];
[1]Centers for Disease Control and Prevention, Atlanta, GA, USA, [2]Colorado Department of Public Health and Environment, Denver, CO, USA, [3]Tri-County Health Department, Greenwood Village, CO, USA, [4]New Mexico Department of Health, Santa Fe, NM, USA, [5]Food and Drug Administration, College Park, MD, USA, [6]Food and Drug Administration, Denver, CO, USA.

**Background:** Listeriosis has a long median incubation period and affects specific higher-risk populations, making outbreak investigations challenging. The *Listeria* Initiative (LI), an enhanced surveillance system that links routinely-collected epidemiologic data on food histories with molecular subtyping data, was started in 2004 to address these issues. On September 2, 2011, the Colorado Department of Public Health and Environment notified CDC of an apparent outbreak. **Methods:** We identified outbreak strains of *L. monocytogenes* and defined cases as illnesses with these strains isolated August 1-October 31. As case food histories were reported, we compared outbreak-associated cases to sporadic cases in patients ≥60 years old with specimens collected August, 2004-2010. Traceback and environmental investigations were initiated simultaneously. **Results:** On September 9, a case-case comparison of 11 outbreak-associated cases to 85 sporadic cases reported to the LI showed an association with cantaloupe (odds ratio=8.5; 95% confidence interval [CI]: 1.3, ∞). By September 14, this association strengthened (n=19 cases; OR=14.9; 95% CI: 2.4, ∞), and Farm A issued a voluntary recall of whole cantaloupes. Four outbreak strains of *L. monocytogenes* were identified. These strains were isolated from cantaloupe samples in patients' homes, grocery stores, and environmental samples from Farm A's processing facility. In total, 146 cases and 31 deaths in 28 states were reported. Most patients were ≥60 years old (86%) and 94% of patients reported eating cantaloupe. **Conclusions:** Rapid interviewing and subtyping in the LI enabled a case-case analysis early in the investigation, which rapidly confirmed an association between the outbreak-associated cases and whole cantaloupe. This epidemiological evidence supported a voluntary recall of Farm A cantaloupes within 10 days of reporting to CDC, preventing additional cases and deaths.

## A Dengue Outbreak in a Small Pacific Island Chain—Republic of the Marshall Islands, 2011–2012

**T.M. Sharp**[1], E.J. Nilles[2], J. Perez-Padilla[1], K.S. Tikomaidraubuta4[3], E. Azures[3], P. Lalita[3], J.L. Munoz-Jordan[1], E. Hunsperger[1], J. Langidrik[3], T.-H. Chen[4], K.M. Tomashek[1], H.S. Margolis[1];
[1]Centers for Disease Control and Prevention, San Juan, PR, USA, [2]World Health Organization, Division of Pacific Technical Support, Suva, Fiji, [3]Majuro Hospital, Majuro, Marshall Islands, [4]Centers for Disease Control and Prevention, Honolulu, HI, USA.

**Background:** Dengue, a potentially fatal febrile illness caused by four mosquito-transmitted dengue viruses (DENV-1-4), is endemic in many regions of the Pacific; however, little is known about dengue in the Republic of the Marshall Islands (RMI). In October 2011, a dengue outbreak was identified by hospital staff in Majuro, RMI. To better understand the epidemiology of dengue in this population, surveillance data were used to identify and describe reported cases. **Methods:** Suspected cases were individuals in RMI that met the 2009 World Health Organization dengue clinical case definition and had a serum specimen submitted for diagnostic testing. Lab-positive cases had the DENV protein NS1 and/or anti-DENV IgM antibody detected with the Standard Diagnostics Dengue Duo rapid diagnostic test. All available serum specimens were also tested by serotype-specific RT-PCR. **Results:** As of January 18, 2012, 1,464 suspected dengue cases had been identified, of which 677 (46.2%) were lab-positive. Of 335 specimens tested by RT-PCR, DENV-4 was detected in 222 (66.3%); no other DENV serotypes were detected. Cases were detected on the atolls of Arno, Enewetak, Kwajalein, Majuro and Utrik, where 0.3%, 1.4%, 0.6%, 2.2% and 1.4% of residents, respectively, tested lab-positive. Individuals 0-9 and 10-29 years of age tested lab-positive at a rate of 6.0 and 41.3 per 1,000 residents, respectively. Atypical clinical cases included two cases of vertical DENV transmission, one case of dengue encephalitis, two individuals co-infected with DENV/*Salmonella* Typhi, and one individual co-infected with DENV/*Mycobacterium leprae*. There were no dengue-related deaths. **Conclusions:** This is the first reported outbreak of dengue in RMI. However, the age groups of affected individuals and known propensity for DENV-4 to cause clinically apparent illness predominantly on secondary DENV infection suggest that: 1) there may have been a dengue outbreak in RMI ~10 years ago that was unrecognized due to lack of surveillance; and 2) the actual number of individuals infected during this outbreak may

have been substantially higher than the number of reported cases. Routine clinical and epidemiologic monitoring is recommended to determine if DENV is endemic in RMI and identify future outbreaks.

**Morbidity Due To Schistosomiasis Among School Children—Kenya, 2011**

**A. Samuels**[1], P. Mwinzi[2], M. Elizabeth[2], G. Muchiri[2], M. Hyde[1], S. Montgomery[1], D. Karanja[2], E. Secor[1];
[1]Centers for Disease Control and Prevention, Atlanta, GA, USA, [2]Kenya Medical Research Institute, Kisumu, Kenya.

**Background:** Schistosomiasis affects >350 million persons worldwide, and is associated with liver abnormalities, stunting, wasting, anemia, exercise intolerance, and decreased quality-of-life. We present baseline data from a multi-year study on the impact of control strategies for *Schistosoma mansoni*-associated morbidity. **Methods:** We randomly selected 822 Kenyans aged 7-8 years from communities with *S. mansoni* infection prevalence ≥25%. Stools were tested for *S. mansoni* and soil-transmitted helminth (STH) infection; blood was tested for malaria and anemia. Abdominal ultrasound, anthropometric, quality-of-life, and exercise tolerance examinations were performed to assess morbidity. Data were analyzed using chi-squared tests and Poisson regression, accounting for clustering with generalized estimating equations. **Results:** A total of 597 children contributed ≥2 stools and complete morbidity assessments. *S. mansoni,* STH, and *Plasmodium falciparum* infection prevalence were 69%, 25%, and 8%, respectively. Anemia and hepatomegaly prevalence were 36% and 67%, respectively. In univariate analysis, anemia was more common in children with schistosomiasis (40%) than without (28%; P=0.01). Hepatomegaly was more common in children with heavy intensity of schistosomiasis, defined by >399 eggs per gram of stool, (79%) compared to uninfected (62%; P=0.01). In multivariate analyses, heavy intensity schistosomiasis (adjusted prevalence ratio [aPR] 1.78; confidence interval [CI]: 1.17-2.69) and malaria (aPR 1.37; CI: 1.06-1.78) were predictors for anemia. Liver abnormalities were more common in children with *S. mansoni* (aPR 0.78; CI: 0.60-0.999) and STH infections (aPR 0.63; CI: 0.42-0.97). Schistosomiasis was not associated with lower quality-of-life, poor exercise tolerance, or abnormal anthropometry. **Conclusions:** Schistosomiasis-associated morbidity in Kenyan school-children included anemia and liver abnormalities. However, measuring impact of schistosomiasis control might be challenging, and new tools are necessary to better inform programmatic decision-making.

## Antimicrobial Resistance

Monday, March 12
5:00 PM – 6:00 PM
Grand Hall

**Board 76. Alarming Trend of Antibiotic Resistance in *Pseudomonas aeruginosa* Isolates**

**M.E. El Zowalaty**;
Zagazig University, Zagazig, Egypt.

**Background:** *P. aeruginosa* is a concerning opportunistic human pathogen frequently causing nosocomial and life threatening infections. Various clinical problems caused by *P. aeruginosa* are partly because the organism is inherently resistant to antibiotics and is able to acquire resistance to most effective antimicrobial drugs. **Methods:** The present study was thus conducted to determine the occurence of resistance to commonly prescriped antibiotics in *P. aeruginosa* isolated from patients admitted to university-affiliated hospitals in Zagazig, Egypt. A total of 250 specimens were examined. The MICs were determined according to CLSI guidelines. The mechanisms of resistance by β-lactamases and efflux mediated resisatance were invetigated as described previously. **Results:** From 250 specimens

collected, 86 isolates of *P. aeruginosa* (34.4%) were isolated. It was found that piperacillin, meropenem, amikacin, and polymyxin B were the most effective antibiotics and all tested isolates were susceptible (100%), followed by imipenem (99%), ticarcillin (80%), ciprofloxacin (59%), ceftazidime (58%), cefipime (40%), gentamicin (9%) and norfloxacin (8%). *P. aeruginosa* isolates were highly resistant to aztreonam, azithromycin, ceftriaxone, and amoxicillin/clavulanate. It was found that β-lactamase production and multiple drug resistance efflux pumps were predominant in resistant isolates. The present results showed that 42 (48.8%) of *P. aeruginosa* isolates were β-lactamase producers. In addition, efflux pump was identified in 34 (39.5%) of *P. aeruginosa* that effectively utilized an efflux-mediated mechanism of resistance against cefotaxime, ticarcillin, azetreonam, meropenem, and norfloxacin but not to streptomycin. The MICs of selected antibiotics were determined in presence and absence of efflux inhibitors dinitrophenol (DNP) and dicyclohexylcarbodiimide (DCCD). The addition of DNP and DCCD enhanced the activities of selected antibiotics as observed by reduction of MIC. **Conclusions:** The importance of the study is indicating that escalating rates of MDR among *P. aeruginosa* isolates remain a concerning clinical problem facing patients and health officials. This requires an urgent need for control policy of prescription and use of antibiotics in hospitals for prevention of steady increase in resistance.

**Board 77. The Use of *Escherichia coli* as a Sentinel for Antimicrobial Resistance in *Salmonella***

**N.A. Womack**;
US Food and Drug Administration, Laurel, MD, USA.

**Background:** National monitoring programs for antimicrobial-resistant enteric pathogens often include *Escherichia coli* as a sentinel for the consequences of selection pressure in samples where *Salmonella* is not recovered. The purpose of this study is to determine the predictive power of *E. coli* for resistance in *Salmonella* from retail meats in the National Antimicrobial Resistance Monitoring System (NARMS). **Methods:** Following NARMS standard methods, one colony for each bacterium was isolated for susceptibility testing. Antimicrobial minimum inhibitory concentrations (MIC) were determined by broth microdilution following CLSI standards. The predictive power of *E. coli* in determining resistance in *Salmonella* was assessed using both the Positive Predictive Value (PPV) and Generalized Linear Mixed Model (GLMM) analysis. *E. coli* and *Salmonella* were recovered from 15,208 retail meat samples, including chicken breast (n = 3,808), ground turkey (n = 3,762), ground beef (n=3,818), and pork chops (n=3,820) purchased from grocery stores in Georgia, Maryland, Oregon, and Tennessee. We analyzed 9 years (2002-2010) of susceptibility monitoring data for 15 antimicrobial agents. **Results:** There were 3,078 retail samples culture-positive for *Salmonella* and 10,479 retail samples positive for *E. coli*. Of these samples, there were a total of 855 that were simultaneously positive for both *E. coli* and *Salmonella*. When observing the prevalence and PPV of antimicrobial resistance among the 855 paired isolates, we found that the PPV for *E. coli* predicting resistance for *Salmonella* varied by drug and serotype, and was higher when the frequency of resistance was high. In the GLMM analysis, the predicted probability was presented only for those drugs (amoxicillin/clavulanic acid, ampicillin, ceftiofur, cefoxitin, and ceftriaxone) where *E. coli* resistance had a statistically significant effect on *Salmonella* resistance at the 95% probability level. For these drugs, the probability of resistance in *Salmonella* when resistance was present in *E. coli* ranged from 26-43%. **Conclusions:** Results indicate that the predictive power of *E. coli* as a sentinel organism for resistance in *Salmonella* varies by antimicrobial agent and should be considered with caution.

**Board 78. Epidemiology of the Newly Reportable Carbapenem-Resistant Enterobacteriaceae (CRE) in Tennessee**

**A.E. Edwards**, M.B. Crist, M.A. Kainer;
Tennessee Department of Health, Nashville, TN, USA.

**Background:** Antimicrobial resistance is a challenge for medical and public health professionals. Limitations in the availability of new drugs and misuse of antibiotics have resulted in organisms that are more difficult to treat. One group of emerging drug-resistant organisms is carbapenem-resistant *Enterobacteriaceae* (CRE). CRE pose a significant health threat because they exhibit plasmid-mediated resistance that can spread between genera and confer resistance to nearly all classes of antibiotics. In order to proactively assess the presence of these organisms in Tennessee (TN), the TN Department of Health (TDH) recently added CRE to its reportable conditions. The aim was to provide insight into the epidemiology of CRE in TN, and to prevent transmission. **Methods:** All healthcare facilities and laboratories in TN are required to report CRE to TDH by telephone the next day and via written report within one week. Once received, information on species, antibiotic resistance, patient demographics, and specimen source are entered into two databases. One system, National Electronic Disease Surveillance System, is for state and national reporting and the other, WHONET, is for analysis of resistance patterns. To ensure accuracy of the data, reports are validated to ensure they meet the new Clinical Laboratory Standards Institute breakpoints. **Results:** From 1/2/2011-9/30/2011, 101 cases of CRE were reported to the state. Most cases occurred in adults with an average age of 61 (median=66); 7 pediatric cases were also reported. Of the reported cases, 39% occurred among men and 61% among women. *Enterobacter cloacae* (43%) is the most common isolate, followed by *Escherichia coli* (32%), *Klebsiella pneumoniae* (19%), *Serratia marcescens* (3%), *Citrobacter freundii* (2%), *Morganella morganii* (2%), and *Providencia stuartii* (1%). The level of resistance was also determined for doripenem (High (H)=57%, Low (L)= 43%; N=7), ertapenem (H=52%, L= 47%; N=92), imipenem (H=84%, L=16%;N=19); and meropenem (H=75%, L=25%; N=28). **Conclusions**: TDH will use these data to inform policies regarding CRE infection control in healthcare settings, including nursing homes and physician offices. At present, we are expanding our state laboratory's capacity to include testing for the plasmids responsible for conferring carbapenem resistance.

**Board 79. Antimicrobial Susceptibility of Commensal *Escherichia coli* from Cattle, Pigs, and Chickens (2002-2006) Recovered from 8 EU Countries (EASSA Program)**

**A. de Jong**, S. Simjee, M. Vallé, H. Moyaert, V. Thomas, P. Butty, U. Klein, H. Marion, T. Shryock;
EASSA Study Group, Brussels, Belgium.

**Background:** The European Antimicrobial Susceptibility Surveillance in Animals (EASSA) is the first ongoing program to study the susceptibility to human-use antibiotics of zoonotic and commensal bacteria from healthy food animals across the EU. Here, antimicrobial susceptibility is reported for commensal *E. coli (Ec)* recovered from 2002 to 2006. **Methods:** Colon or caecal content was randomly collected at 4 abattoirs per country (5 countries per host). Each herd/flock was sampled once. *Ec* were isolated using standard methods. Susceptibility testing against 9 antibiotics was done by agar dilution (CLSI, M31-A3) in a central laboratory. Clinical resistance (CLSI, M100-S21, except colistin: EUCAST) was assessed per antimicrobial and country; decreased susceptibility (DS), applicable for 4 antibiotics, was based on epidemiological cut-off (ECV) values as defined by EFSA (2008) and comprises the number of isolates with MIC values exceeding the wild-type MIC distribution (>ECV) but not deemed to be clinically resistant (<clinical breakpoint). **Results:** In total 4501 *Ec* were recovered (cattle (Ca) n=1396, pigs (P) n=1543, chickens (Ch) n=1562). Mean resistance (%) for *Ec* for each respective animal host was: ampicillin (A) 6.2, 32.2, 53.1; cefepime 0, 0, 0; cefotaxime 0, 0.2, 4.0; ciprofloxacin 1.0, 0.4, 5.6; chloramphenicol 3.2, 16.5, 14.4; colistin 0.1, 0.4, 0.2; gentamicin (G) 1.4, 1.6, 3.5; tetracycline 12.3, 67.1, 67.2; and trimethoprim/sulfamethoxazole 5.6, 40.3, 48.2. DS was particularly apparent for ciprofloxacin (2-4 % for Ca and P; 32% for Ch), whereas the corresponding figures for cefotaxime were 0.4 and 1.9 %. In case of A and G, DS was negligible (<0.7%). Overall, France and Italy (Ca), France and Spain (P, Ch) and Germany and Netherlands (Ch) showed the highest number of resistant isolates; Denmark (P) showed the lowest. **Conclusions**: This pan-EU survey, with standardized methods, shows variable antimicrobial

resistance among enteric *E. coli* isolates at slaughter. Prevalence of resistance varied strongly for older drugs and between countries but clinical resistance to newer critically important compounds was absent to low. For the latter, decreased susceptibility varied from very low to high. Epidemiological cut-off values are important tools to assess epidemiological resistance to the new classes.

**Board 80. AFHSC-GEIS DoD Antimicrobial Resistance Surveillance Efforts and Vision Forward**
**R.M. Chandrasekera**, D. Blazes;
Armed Forces Health Surveillance Center, Silver Spring, MD, USA.
**Background:** The Armed Forces Health Surveillance Center (AFHSC) has become more invested in surveillance regarding antimicrobial resistant organisms (ARO). In FY11, AFHSC-GEIS supported 12 ARO activities in six countries. Wound infections have been a common complication of injury for US military personnel serving in combat zones. Outbreaks of *Acinetobacter* infections have been common, requiring continuous surveillance with genetic characterization to direct infection control measures. **Methods:** AFHSC has supported development of a centralized microbiology lab within the military to characterize multi-drug resistant pathogens and assist in DoD outbreak investigations. AFHSC also supports several Methicillin-resistant *Staphylococcus aureus* studies in recruit and active duty populations in constrained environments (e.g. shipboard populations) where risk for infection is high. Outside the Continental United States, AFHSC has supported several healthcare-associated infection surveillance efforts. **Results:** In FY11, surveillance was initiated at several hospitals in Peru to determine patterns of antimicrobial drug resistance among nosocomial infections. Preliminary findings showed nearly 50% of isolated *Acinetobacter* were multidrug resistant. Several activities abroad also include antimicrobial susceptibility testing of isolates collected through previous studies and development of advanced characterization techniques. For example, techniques have been developed by the Armed Forces Research Institute of Medical Sciences to identify and characterize extended spectrum beta-lactamase genes in *Enterotoxigenic E. coli* (ETEC) after a study was conducted to look at resistance mechanisms in ETEC. **Conclusions:** Moving forward, AFHSC-GEIS aims to develop a robust antimicrobial surveillance network. This will be accomplished by leveraging existing laboratory infrastructure, equipment and personnel, further defining priorities regarding resistant pathogens, and through the development of data standardization tools that allow comparison of data across regions and longitudinally. The ultimate goal is to generate data that will inform policies and interventions to better treat and prevent infectious disease threats to both military and civilian populations.

**Board 81. Establishment of In Vitro Susceptibility Profiles for *C.gattii* Molecular Types**
**N.J. Iqbal**[1], C. Bolden[1], E. DeBess[2], N. Marsden-Haug[3], R. Wohrle[3], J.R. Harris[1], S.R. Lockhart[1];
[1]Centers for Disease Control and Prevention, Atlanta, GA, USA, [2]Oregon Department of Human Services, Portland, OR, USA, [3]Washington State Department of Health, Tumwater, WA, USA.
**Background:** *Cryptococcus gattii* causes invasive disease in both humans and animals. *C. gattii* can be divided into four molecular types that may define cryptic species. There is evidence that molecular types of *C. gattii* are restricted geographically and susceptibility to antifungal drugs may differ among the types. We performed susceptibility testing on *C. gattii* isolates representing each of the four molecular types to determine if antifungal susceptibility differences exist. **Methods:** A total of 298 *C. gattii* isolates stored at Centers for Disease Control and Prevention and obtained from human, animal, and environmental sources from Bostwana, South Africa, Australia, India, US, and Canada were used in the study. Multilocus sequence typing (MLST) analysis of the *URA5* and *IGS* genes was used to group the strains into previously-defined molecular types. Susceptibility data for fluconazole (FLU), voriconazole (VOR), itraconazole (ITR), and posaconazole (POS) were generated for all isolates using broth microdilution as described in CLSI document M27 A-3. **Results:** VGII isolates had the highest geometric mean (GM) MIC values for FLU, VOR, and ITR and VGI isolates had the lowest GM MIC for all drugs

tested. These differences were statistically significant ($p<0.001$). With the exception of posaconazole, GM MIC values for each drug tested had the pattern of VGI < VGIII < VGIV < VGII. For posaconazole alone VGII isolates had the same GM MIC as VGIII and VGIV isolates. The VGIIc subtype from the current emergence in the Pacific Northwest US had the highest GM MIC values for FLU (21.7) and VOR (0.23) and may represent clonal expansion of an azole resistance phenotype. **Conclusions:** Isolates of molecular type VGII were less susceptible than other molecular types *in vitro* to FLU, VOR, and ITR. Differences in *in vitro* susceptibility imply that molecular types of *C. gattii* could respond differently to antifungal treatment. Because the molecular types are somewhat associated with geography, geographic origin of an isolate may help determine its *in vitro* susceptibility to antifungals.

## Influenza Preparedness: Lessons Learned

Monday, March 12
5:00 PM – 6:00 PM
Grand Hall

**Board 82. Comparison of Clinical Features between Seasonal and Pandemic Influenza Cases in Pakistan**

**N. Badar**, U. Bashir Aamir, R. Mehmood, N. Nisar, S.Z. Zaidi, B.M. Kazi;
National Institute of Health, Pakistan, Islamabad, Pakistan.

**Background:** In April 2009, a novel pandemic influenza A H1N1 emerged that spread globally causing significant morbidity worldwide. Various studies have shown the clinical features of pandemic H1N1 cases were prolonged while the disease severity was comparable between seasonal and pandemic H1 cases. This study analyzed the statistical significance of clinical features and co-morbid conditions among seasonal influenza A (H1N1 and H3N2), Pandemic influenza A H1N1 2009 (Pdm H1) and influenza B. **Methods:** Respiratory samples (Nasopharyngeal or throat swabs) and epidemiological data from suspected ILI/SARI patients (based on WHO case definitions) collected from January 2009 to April 2010 were included in the study. Samples were analyzed by real time PCR according to CDC protocols. The epidemiological data including clinical features (fever, cough, sore throat and shortness of breath) and predisposing risk factors (allergies, respiratory, liver, metabolic, heart diseases and smoking) from these three positive influenza groups was analyzed based on results for influenza subtype. **Results:** A total of 2275 patients were sampled out of which 614(27%) were positive for influenza of which seasonal Infl A (18 with A/H1, 72 with A/ H3), 279 were influenza pdm/H1 and 245 Inf-B. Mean ages in the groups were 10 +/-13.2 years, 28 +/-17.4 years and 24 +/-19.9 years. Age was observed as a significant contributing factor among influenza groups by analysis of variance ($P<.001$). Multivariate analysis of clinical features among the groups showed statistically significant differences for fever ($p < .001$), cough ($p < 0.001$), sore throat ($p = 0.013$) and shortness of breath ($p = 0.002$). The hospitalization trend was significantly higher for Pdm H1 and Influenza B cases ($p < 0.001$). The impact of predisposing risk factors was not statistically significant among the three groups. **Conclusions:** Our study showed a significantly high incidence of fever and cough in seasonal H1 and H3 infections as compared to Pdm H1 and seasonal Inf B. On the other hand, symptoms such as sore throat and shortness of breath were more frequent in influenza B and Pdm A H1N1 infections. In conclusion, the epidemiology of PdmH1 in Pakistan was significantly different for some clinical features from seasonal influenza subtypes circulating before and during the pandemic.

**Board 83. Epidemiology and Burden of Influenza among Children Less Than 5 Years Old in Suzhou, China**

**T. Zhang**[1], Y. Ding[2], J. Zhang[3], D. Wang[1], Q. Zhu[1], J. Wu[1], Y. Jiang[1], Z. Feng[4], J. McFarland[5], M. Steinhoff[6], S. Black[6], G. Zhao[1];
[1]School of Public Health, Fudan University, Shanghai, China, [2]Suzhou Children Hospital, Suzhou, China, [3]Suzhou CDC, Suzhou, China, [4]China CDC, Beijing, China, [5]US CDC, Beijing, China, [6]Cincinnati Children's Hospital, Cincinnati, OH, USA.

**Background:** Data on the epidemiology and disease burden of children with laboratory-confirmed influenza in China are limited. Prospective data are needed to develop vaccine policy and improve pandemic preparedness. **Methods:** A retrospective study was conducted on children with documented influenza infection hospitalized at Suzhou Children Hospital during 2005 to 2009. Since March 2011, we conducted a prospective study to identify influenza-like illness (ILI) cases with influenza confirmed by reverse transcriptase-polymerase chain reaction or immunofluorescent assay in outpatient clinics and inpatient wards from Suzhou Children Hospital in China. The socio-economic information, medical expenditure, absenteeism and other indirect costs incurred by ILI cases of outpatient and inpatient have been collected. At the same time, the medical resources were also investigated in Suzhou City. Age-specific excess rate of hospitalization was calculated and extrapolated to estimate Suzhou annual influenza pneumonia hospital admissions. The preliminary data of retrospective study and prospective study from March to August were analysis. **Results:** A total of 480 hospitalized children positive by immunofluorescent assay for influenza had been identified during 2005 to 2009. The hospitalizations for influenza occurred year round, most commonly in the winter with a second late summer peak (August-September). The excess rate of hospitalization associated with influenza among children less than 5 years old in different years and age group was estimated to between 15.4 - 378.3/10,000. From March 2011 to August 2011, influenza was identified in 20 (7.5%) ILI outpatients and in 4 (2.2%) ILI inpatients. There were 48.2% ILI outpatients had medicine before doctor visit and about 42.6% outpatients went to the hospital two or more times. Thus the mean medical expenditure of ILI outpatients was 1029.1 RMB. The absenteeism was 7.0 days (IQR: 3.4-8.5 days). The hospitalization costs of the ILI inpatients were 4488.9 RMB (IQR: 3565.5 - 5692.0). **Conclusions:** Influenza hospitalization in young children occurs throughout the year in Suzhou. The burden of influenza among children less than 5 years old in Suzhou area may be underestimated, indicating that immunization of influenza vaccine could be beneficial.

**Board 84. Evaluation of Definitions Used To Identify Influenza among Hospitalized Patients – Evidence from a Rural Population in Western India**

**S. Hirve**[1,2], M. Chadha[3], K. Lafond[4], F. Dawood[4], P. Lele[1], S. Sambhudas[1], A. Deoshatwar[1,3], S. Juvekar[1], R. Lal[5], A. Mishra[3];
[1]KEM Hospital Research Center, Pune, India, [2]Department of Public Health and Clinical Medicine, Umea University, Umea, Sweden, [3]National Institute of Virology, Pune, India, [4]Centers for Disease Control and Prevention, Atlanta, GA, USA, [5]Centers for Disease Control and Prevention, New Delhi, India.

**Background:** Syndromic case definitions for influenza surveillance are still a matter of considerable debate due in part to the wide clinical spectrum of influenza, which can present differently depending on patient age and virus sub-type. As a result, case definitions are not standardized across surveillance programs, making global comparison of disease burden estimates difficult. The Influenza Disease Burden, India study used very broad clinical criteria, enrolling in-patients with any acute medical illness in the 22 villages covered by the Vadu Demographic Surveillance Area, Pune. **Methods:** From May 2009 to April 2011 we enrolled hospital admissions with any acute medical illness, including acute exacerbations of pre-existing chronic illness, collecting detailed information on clinical presentation. Nasal and throat swab specimens were tested by RT-PCR for influenza. Sensitivity and specificity analysis was carried out for individual and grouped clinical symptoms, as well as for commonly-used definitions

of influenza-like illness (ILI), acute respiratory infection (ARI) and severe acute respiratory infection (SARI). **Results:** Among 3,291 enrolled inpatients suspected for influenza, 667 (20%) tested positive for influenza. Fever was the most common symptom in both influenza positive (96%) and negative (93%) patients. Cough (93% and 79%) and sore throat (58% and 37%) were more common in influenza positive than influenza negative patients. Difficulty breathing was seen in less than 5% of influenza positive patients. The WHO ILI definition had a sensitivity of 76%, specificity 39%, and PPV 28%. Broadening the WHO ILI definition to include reported fever gave a higher sensitivity of 93%, with specificity 20%, and PPV 26%. The SARI case definition, which included difficulty breathing, had the lowest sensitivity of 5%, high specificity 94%, and PPV 24%.The broader ARI definition had the highest sensitivity of 99%, with lowest specificity 1%, and PPV 20%. **Conclusions:** Our findings suggest that replacing measured fever by reported fever increases the sensitivity but decreases the specificity of the ILI definition for finding laboratory-confirmed influenza. The SARI case definition (which includes difficulty breathing) results in missing influenza cases and underestimating the true disease burden.

## Zoonotic and Animal Diseases

Monday, March 12
5:00 PM – 6:00 PM
Grand Hall

**Board 85. Reports of Ill or Dead Dogs Entering the United States, 2005 – 2010**

**J.R. Sinclair**;
Centers for Disease Control and Prevention, Philadelphia, PA, USA.

**Background:** An estimated 287,000 dogs entered the United States during 2006; dogs are of public health interest because they can transmit multiple zoonotic diseases, including rabies, leptospirosis, leishmaniasis, and roundworms. US federal regulations require that dogs be healthy upon arrival into the United States and be fully immunized against rabies. Accordingly, carriers are required to notify the US quarantine station upon arrival of any ill or dead dogs. Importers of unvaccinated dogs are required to confine the dog upon arrival until it is fully immunized against rabies. **Methods:** Free-text reports entered into the CDC's Quarantine Activity Reporting System (QARS) during 2005 - 2010 were systematically reviewed. The terms "pup" and "dog" were used to identify all dog import reports in QARS. These reports then were searched for key terms related to illness or death (e.g., dead, deceased, sick, diarrhea, vomit, necropsy, and lethargic). All QARS reports of ill or dead dogs with no known prior history of illness were entered into a Microsoft Access database for further analyses. **Results:** A total of 144 dogs (range 1- 17 dogs per QARS report) were described as ill or dead upon arrival into the United States, with hypoglycemia or dehydration as the most common presumptive diagnoses. Where age was reported, the majority of ill or dead dogs [70%, (70/101)] were 4 months of age or younger, with brachycephalic and toy breeds most commonly listed. Ill or dead dogs arrived from at least 28 countries, 21 of which are considered rabies-endemic by the World Health Organization. Fewer than half of the reports [41% (39/94)] indicated that the dogs, whether reportedly originating from rabies-free or -endemic countries, were fully vaccinated against rabies. **Conclusions:** Among ill or dead dogs reported in QARS, puppies are more likely to arrive ill or dead, and thereby require additional efforts to rule out an infectious disease as the cause. In addition, unvaccinated dogs represent a health threat for the reintroduction of canine rabies to the United States. Revisions to federal regulations should aim to minimize possible transmission of zoonotic diseases through animal vaccination, deworming, and identification requirements for US entry.

**Board 86. Risk Factors for Avian Influenza A/H5N1 in Backyard Poultry Flocks in Bangladesh**

**N. Haider**[1], M. Goni Osmani[2], E. Azziz-Baumgartner[3], M. Salah Uddin Khan[1], S.K. Paul[1], M.A. Hasnat[1], M.Z. Rahman[1], M. Rahman[1], K. Hossain[1], D. Southern[1], E.S. Gurley[1], A.B. Mikolon[1,3], S.P. Luby[1,3];
[1]International Centre for Diarrheal Diseases Research, Bangladesh (ICDDR,B), Dhaka, Bangladesh, [2]Department of Livestock Services, Ministry of Fisheries and Livestock, Bangladesh, Dhaka, Bangladesh, [3]Centers for Disease Control and Prevention, Atlanta, GA, USA.

**Background:** Backyard poultry are raised by 69% of Bangladesh households. Since 2007, 51 of country's 64 districts have reported influenza A/H5N1 outbreaks in poultry. The three human influenza A/H5N1 cases that have been identified in Bangladesh all had an exposure history to poultry. Controlling outbreaks in poultry can reduce the risk of transmission to humans. We identified risk factors for avian influenza A/H5N1outbreaks in backyard poultry flocks. **Methods:** During March 2009 to July 2011, we enrolled 25 backyard poultry flocks having at least one poultry tested positive for influenza A/H5 by real time RT-PCR, identified through surveillance. We enrolled 119 geographically matched control flocks within a 10 km radius of these cases, in which there was no reported poultry mortality within the previous two weeks, and where poultry and environmental samples tested negative for influenza A/H5. We considered the onset of the outbreak to be when the first chicken died in the case flocks. We collected information from case and control households on poultry flock-size, presence of dead birds, and live bird market visits by household members. **Results:** Case flocks had more chickens than controls [25 vs. 10 birds, p=0.008] and were located closer to the local government livestock office (4.0 vs. 5.4 km, p=0.01). Compared with control flocks, case flock owners were more likely to visit a live bird market within two weeks preceding the poultry outbreak (68% vs. 25%, p<0.001) and more likely to see dead birds within a 500 meter radius of their households (56% vs. 19%, p<0.001). In a multivariate conditional logistic regression model, household members visiting a live bird market [OR = 7.4, CI=1.8-31], seeing dead birds within a 500 meter radius of their households during the two weeks preceding an outbreak (OR=5.8, CI=1.8-18.7) and flock size (OR=1.04, CI: 1.005-1.07) were associated with avian influenza A/H5N1 infection in case flocks. **Conclusions:** In Bangladesh, live bird markets apparently played a role in spreading influenza A/H5N1 to backyard poultry and larger flocks were at increased risk. Prevention efforts should target live bird markets and the poultry marketing chain to reduce the spread of influenza A/H5N1 in backyard poultry. Increased sighting of dead birds may signal a high risk that could encourage preventive actions.

**Board 87. Pandemic Influenza H1N1 in Pigs Raised in Small Holder Farms in Kenya, 2010**

**P.M. Munyua**[1], M. Junghae[1], J. Halliday[2], E. Ogola[3], L. Mwasi[3], R.F. Breiman[1], J. Mott[1,4], M.A. Katz[1,4], C. Cardona[5], M.K. Njenga[1];
[1]Centers for Disease Control and Prevention-Kenya, Nairobi, Kenya, [2]College of Medical, Veterinary and Life Sciences, University of Glasgow, Glasgow, UK, [3]Kenya Medical Research Institute/Centers for Disease Control and Prevention-Kenya, Nairobi, Kenya, [4]Centers for Disease Control and Prevention, Atlanta, GA, USA, [5]College of Veterinary Medicine, University of Minnesota, St. Paul, MN, USA.

**Background:** Since the emergence of the 2009 pandemic influenza A H1N1 (pH1N1) virus in 2009, and its subsequent global spread in humans, the virus has been detected in multiple animal species. Our objective was to define the prevalence of influenza A viruses in pigs taken for slaughter in a pig slaughterhouse located in Nairobi, and determine if the pH1N1 virus that was introduced into the human population in Kenya in July of 2009 had spread to pigs by May 2010. **Methods:** The study was conducted among pigs brought to the slaughterhouse during May 4-17, 2010. Pigs arrived in groups and a maximum of 2 pigs from each group were randomly selected and blood, nasal, and rectal swabs collected immediately post-mortem. A standardized questionnaire was administered to the trader to collect data on pig numbers at the farms from which the sampled pigs came. Swabs were tested for influenza A by rt RT-PCR, and sera tested for anti-influenza A nucleoprotein antibodies by ELISA. All

ELISA-positive sera were tested by hemagglutination inhibition (HI) for antibodies to human influenza A strains pH1N1, seasonal H1N1 and H3N2 using the 2009/2010 WHO influenza diagnostic kit following standard protocols. **Results:** Data were collected from 203 pigs from 178 groups. The median number of pigs in each group was 1 (range 1-33). Only 38/178 groups had data on the number of pigs at the farms they came from; of these, the median number of pigs at the farm was 90 (range 2-1000). Thirty (14.9%) of the sera were positive for influenza A antibodies. The seropositive pigs were drawn from 29 (16.3%) groups. On HI assay, 25 (83.3%) of the sera were reactive to the pH1N1 antigens and one (3.3%) was reactive to both pH1N1 and seasonal H1N1 antigens. None of the sera were reactive to the H3N2 antigens. In total, 3(1.5%) nasal and 3(1.5%) rectal swabs from six pigs were positive for influenza A. Virus culture and subtyping of the six influenza A positive swabs is pending. **Conclusions:** We report possible circulation of pH1N1 among pigs raised in small holder farms across Kenya, though we cannot rule out serologic cross reaction with antibodies to other SIV strains. Pigs play an important role in the evolution of influenza strains and continued surveillance in pigs, and in humans, may be of value for identifying the emergence of novel influenza strains.

**Board 88. Clinical and Epidemiologic Description of Fatal Leptospirosis Cases—Puerto Rico, 2010**

**T.M. Sharp**[1], J. Pérez-Padilla[1], B. Rivera[2], R. Galloway[3], M. Swaminathan[3], W.-J. Shieh[3], J. Bhatnagar[3], D.M. Blau[3], G. Han[1], A. Rivera[1], R. Rodriguez-Acosta[1], S.R. Zaki[3], W. Bower[3], K.M. Tomashek[1];
[1]Centers for Disease Control and Prevention, San Juan, PR, USA, [2]Puerto Rico Department of Health, San Juan, PR, USA, [3]Centers for Disease Control and Prevention, Atlanta, GA, USA.

**Background:** Leptospirosis, a potentially fatal infection with a mortality rate of ~10%, is endemic in Puerto Rico where up to 100 cases are reported each year to the Puerto Rico Department of Health. In January, 2010, CDC's Dengue Branch initiated enhanced surveillance for deaths following an acute febrile illness (AFI), which included testing of specimens taken at autopsy and review of death certificates at the Vital Registry of Puerto Rico. In the present study, we sought to describe the fatal leptospirosis cases identified. **Methods**: Lab positive leptospirosis cases were defined as having: (i) *Leptospira* antigen detected by immunohistochemistry (IHC) in a tissue specimen; (ii) *Leptospira* genome detected by PCR in DNA extracted from a blood specimen; or (iii) a record of anti-*Leptospira* IgM antibody detected by a private laboratory while the case was hospitalized. Suspect cases had "leptospirosis" listed on the death certificate with no or negative *Leptospira* diagnostic test results. Testing to rule out dengue virus (DENV) infection was performed for all cases with specimen available. Review of medical charts from all health care visits was performed for all cases. **Results:** We identified 19 lab positive and 5 suspect leptospirosis deaths in 2010. Of the lab positive cases, 14 had *Leptospira* antigen detected by IHC, 7 had detectable anti-*Leptospira* IgM antibody, and 3 had *Leptospira* genome detected by PCR in a blood specimen; in one case, multi-locus sequence typing identified the infecting serovar as Icterohaemorrhagiae/Copenhageni. DENV infection was ruled out in 75% of all cases; one *Leptospira*/DENV co-infection was identified. The median age of all cases was 51 years (range: 19-84), and 19 (79%) were male; most cases were either unemployed or agricultural workers. The peak number of deaths occurred in October, which is typically at the end of the rainy season in Puerto Rico. Frequent clinical findings included thrombocytopenia (100%), metabolic acidosis (92%), acute kidney failure (88%), jaundice (75%), altered mental status (63%), edema (58%), hematuria (58%), and hemoptysis (42%). **Conclusions:** We identified a case series of 24 fatal cases of leptospirosis in 2010. Deaths occurred at a rate of 0.64 per 100,000 residents of Puerto Rico, suggesting a burden of disease larger than is being reported.

**Board 89. Contribution to Statewide Rabies Prevention Efforts of Public Animal Rabies Vaccination Clinics, Maryland, 2006-2008**

**K.C. Mitchell**[1], S.B. Dolan[1,2], K.A. Feldman[1];
[1]Department of Health and Mental Hygiene, Baltimore, MD, USA, [2]Emory University, Atlanta, GA, USA.
**Background:** Rabies vaccination of dogs, cats, and ferrets is mandated in Maryland, and the 24 local health departments (LHDs) are required to offer low cost animal rabies clinics. The scope and effectiveness of these clinics has not previously been described. **Methods:** LHDs provided the number of vaccinations administered to dogs, cats, and ferrets at public clinics for 2006 - 2008; descriptive analyses were conducted. The proportions of owned dogs and cats vaccinated at a public clinic were estimated using American Veterinary Medical Association formulae for estimating pet populations. Qualitative data on clinic characteristics were summarized. Clients at two clinics completed questionnaires to assess the value of public clinics and identify barriers to rabies vaccination. **Results:** Twenty-three counties conducted 6 clinics (range 2-12), on average, each year. In 2006, 23 counties reported vaccinating 22,638 animals at public clinics; in 21 counties that reported species, 13,850 dogs, 6,715 cats, and 182 ferrets were vaccinated. In 2007, 21,604 animals were vaccinated in all counties. In 2008, 22 counties reported vaccinating 23,502 animals. The proportion of owned dogs and cats vaccinated at public clinics in 2008 was 0.77%; this likely underestimates the proportion of owned pets actually due for vaccination because no data exist to estimate the denominator. For owned dogs, the proportion vaccinated at public clinics in 22 counties was 1.1%; for owned cats, the proportion was 0.4. Dogs were 2.72 times more likely to be vaccinated at a public clinic than cats (95% CI 2.65-2.80). Seven counties reported the annual cost to conduct clinics was \$1,355-\$9,000. Of 132 clients surveyed, 82% attended a public clinic for affordability; only 4% would not vaccinate their pet if public clinics were not available. **Conclusions:** Thousands of Maryland animals are vaccinated each year at public clinics, contributing to statewide rabies prevention efforts; yet these account for a small proportion of owned dogs and cats in the state. Dogs are more likely to be vaccinated at public clinics than cats, but this likely reflects overall rabies vaccination practices, regardless of setting. Although most clients indicated they would rabies vaccinate their pets if public clinics were not available, some suggested that cost would prohibit vaccination.

**Board 90. Evidence of Rickettsial Infections in White-tailed Deer (*Odocoileus virginianus*) in Caroline County, Virginia**

**K.E. Mullins**[1,2], J. Jiang[1], M.K. Miller[3], M.L. Desena[3], J.W. McBride[4], A.L. Richards[1,2];
[1]Naval Medical Research Center, Silver Spring, MD, USA, [2]Uniformed Services University of the Health Sciences, Bethesda, MD, USA, [3]Public Health Command Region-North, Ft. Meade, MD, USA, [4]University of Texas Medical Branch, Galveston, TX, USA.
**Background:** Rickettsial infections can be caused by a variety of genera including *Rickettsia*, *Anaplasma*, and *Ehrlichia*, all of which are obligate intracellular, vectorborne, Gram-negative bacteria. Several species are endemic in the United States, including *E. chaffeensis*, *A. phagocytophilum*, and spotted fever group (SFG) *Rickettsia* (*R. parkeri* and *R. rickettsii*), and are transmitted by ticks. Due to the large white-tailed deer (*Odocoileus virginianus*) population in the United States, which are in constant contact with ticks, prevalence of rickettsial infections in deer, current or past, should be a good indicator of the (re) emergence of tick-borne rickettsial agents in the United States. **Methods:** In this study blood samples were collected from 50 deer (with ticks present) in Caroline county, Virginia. Serum was screened for antibodies to *E. chaffeensis* and *SFG Rickettsia* using an *E. chaffeensis* IgG ELISA assay (120 kDa protein antigen) and a SFG IgG ELISA assay (*R. conorrii* antigen). DNA was extracted from the samples for use in qPCR to identify deer infected with *Anaplasma*, *E. chaffeensis*, and SFG *Rickettsia* (primers and probes: specific for *msp2*, 16s rRNA, and *ompB*, respectively). **Results**: The SFG ELISA found 33 deer positive for antibodies by screening at a 1:100 serum dilution and 27 were confirmed by titer format ELISA. No deer were positive for *E. chaffeensis* upon screen ELISA. Additionally, the results from

the qPCR found 10 deer positive for *Anaplasma*, 3 deer positive for SFG *Rickettsia*, and no deer positive for *E. chaffeensis*. **Conclusions:** The lack of positive samples for *E. chaffeensis* was surprising considering *E. chaffeensis* is endemic in the United States and deer have been identified as a reservoir for *E. chaffeensis*. The qPCR results for *Anaplasma* help confirm reports that deer can be infected with *Anaplasma*. Antibodies to SFG *Rickettsia* are also evidence that SFG *Rickettsia* have the ability to infect deer. Overall, the results seem consistent with the (re) emergence of tick-borne rickettsial agents, excluding *E. chaffeensis*, and confirm the presence of tick-borne agents infection of deer in the southeastern United States.

**Board 91. Virulent Genes Profile, Serotypes, and Antimicrobial Resistance of Shiga Toxin-Producing *Escherichia coli* (STEC) in Weaned Dairy Calves—Minnesota, 2009**

**G.S. Ejigu**[1], S. Godden[2], S. Sreevatsan[2], J. Bender[2];
[1]University of Minnesota, Minneapolis, MN, USA, [2]University of Minnesota, St. Paul, MN, USA.

**Background:** Cattle are an important reservoir for *Shiga*-toxin producing E. coli. **Methods:** From April to November 2009, pooled fresh fecal samples from weaned dairy calf pens were sampled from fifty dairy farms during spring, summer and fall visits (5 samples per farm per visit). Standard microbiological analyses were applied to identify STEC serotypes, associated virulent genes, and antibiotic susceptibility. **Results:** STEC was isolated from 47 of 50 herds. Most STEC isolates carried stx1 gene (26%). Fifteen, 11, 7 and 6% carried hlyA, eaeA, stx2 and both stx1 and stx2 respectively. The predominant isolates were non-O157 serotypes. The most frequent were O84:H2, OM:H2, O26:H11, O161:H25, O103:H2, O157:H7. All isolates tested were resistant to penicillin, clindamycin and tilmicosin. One hundred one (86.8%), 94 (62%), 91 (60%), 58 (38%), 41 (27%), 33 (21.9%) and 2 (1.3%) of isolates were resistance to sulphadimethoxime, florfenicol, tetracyclines/oxytetracycline, neomycine, ampicillin, ceftiofur, and enrofloxacin, respectively. Seventy-eight percent of isolates tested were multidrug-resistant. There was no association between herd size and shedding of STEC (P = 0.89) but sampling season was associated with farm level prevalence (P = 0.005). Our study indicated weaned dairy calves shed a range of STEC serotypes carrying various virulent genes and an increased number of isolates were multidrug-resistant.

**Board 93. Equine Influenza A Epizootic in Mongolia, 2011: Isolation and Characterization of a H3N8 Virus**

**G.L. Heil**[1], M. Yondon[2], B. Jamiyan[2], B. Zayat[2], G.C. Gray[1];
[1]Department of Environmental and Global Health, College of Public Health and Health Professions, and Emerging Pathogens Institute, University of Florida, Gainesville, FL, USA, [2]Institute of Veterinary Medicine and Department of Veterinary and Animal Breeding, Ulaanbaatar, Mongolia.

**Background:** In the early spring of 2011, a limited outbreak of influenza-like-illness occurred among horses near the Mongolian capital Ulaanbataar. Within a few weeks of the onset of the outbreak, horse enthusiasts converged upon Ulaanbaatar from every corner of the country to take part in horse races as part of the annual national celebration of Nadaam. As owners and their horses returned to their homes the viruses traveled with them to each of the 21 aimags (provinces) in Mongolia, thus far causing an 74,608 illnesses and 40 deaths among Mongolia's 1.92 million horses. In the work presented here, we sought to isolate and sequence the genome of the virus associated with this epizootic to determine the nature of its origins. **Methods**: Early in the outbreak swabs from the nares affected animals were obtained for laboratory analysis. Real-time-RT-PCR was used to confirm the presence of influenza A in a subset of the collected specimens. Influenza A positive specimens were cultured in eggs resulting in the amplification of the virus. Viral RNA was isolated and influenza genome segments were amplified by gene segment specific RT-PCR. Gene segments were sequenced by the method of Sanger. The resulting sequence data was compared to data in the NCBI sequence database to identify likely viral source of each gene segment. **Results:** Three viral isolates were obtained from the outbreak. BLAST analysis of

sequence data revealed that the HA, NA, and M genes segments were most similar to those of viruses last seen circulating in neighboring Kazakhstan in 2007 (A/Equine/Otar/764/2007(H3N8)) with approximately 96-98% identity for the three gene segments. **Conclusions:** While further genomic analyses are in progress, the viruses associated with the 2011 Mongolian equine influenza epizootic appear to be similar to a H3N8 equine influenza virus that widely circulated in Central Asia during 2007.

## Improving Preparedness for Infectious Diseases

Monday, March 12
5:00 PM – 6:00 PM
Grand Hall

**Board 93. Identification of Diarrheagenic *Escherichia coli* in Southern China**

**Y. Jin**, Q. Liu, Y. Chen, H. Yang;
Longgang Center for Disease Control and Prevention of Shenzhen, Shenzhen,Guangdong, P.R.China, China.

**Background:** Enteric infections remain an important public health problem in Southern China. In this region, diarrheagenic *Escherichia coli* (DEC) is one of the most common pathogens of gastrointestinal diseases, but rarely few studies have been done to detect and classify DEC pathogroups. DEC can be classified into six groups based on distinct molecular, clinical and pathological features. This study aimed at investigating DEC in Longgang District, Shenzhen to provide epidemiology data of different DEC categories. **Methods:** A total of 193 DEC strains were isolated from stool samples collected in 2010 from patients with acute/persistent diarrhea at Longang People Hospital and Center Hospital in Shenzhen. Isolates were identified by traditional biochemical and serological tests. PCR method was used to detect the presence of six different virulence genes of enteropathogenic *E. coli* (EPEC), enterotoxigenic *E. coli* (ETEC), enteroinvasive *E. coli* (EIEC), and enterohemorghagic *E. coli* (EHEC). **Results:** ETEC were the most prevalent, accounting for 50.8% of all DEC isolates. The most common serotype was O25 (23.5% of ETEC isolates). Thirty six isolates (36.7% of ETEC isolates) were *lt* or/and *st* positive. Seventy-four isolates (38.3%) were identified as EPEC, among which 27% carried the *bfp* gene and the most common serotype was O1 (52.7%). There were a total of 19 isolates (9.8%) detected as EIEC, and three of them carried *ipaH* gene. Two O157:non-H7 EHEC were not detected carrying *stx*1 or *stx*2 genes in this study group. **Conclusions:** Our results show that the strains of DEC are important etiological agents in Shenzhen, P. R. China. ETEC and EPEC were the most common subgroups. This study also makes a contribution to the epidemiologic survey of important DEC pathogens and provides scientific basis for further controlling and preventing the diarrhea caused by DEC.

**Board 94. A Survey of Knowledge, Attitudes, and Practices Related to Reporting of Avian Influenza and other Notifiable Infectious Diseases by Public Sector Physicians in Nigeria, 2008**

**I.T. Dalhatu**[1], K.E. Lafond[2], D. Gross[2], E.E. Ekanem[1], S.D. Ahmed[3], P. Peebles[2], V. Shinde[4], A. Mounts[4], A. Nasidi[5];
[1]US Centers for Disease Control and Prevention, Abuja, Nigeria, [2]Centers for Disease Control and Prevention, Atlanta, GA, USA, [3]Federal Ministry of Health, Abuja, Nigeria, [4]World Health Organization, Geneva, Switzerland, [5]Nigeria Centre for Disease Control, Abuja, Nigeria.

**Background**: The first human case of avian influenza (AI) infection in the African region was identified in Lagos, Nigeria in January 2007. Case finding for human infection with AI and other notifiable diseases relies on the ability of physicians to identify suspected cases, collect specimens for testing, and report to the Ministry of Health. The purpose of this study was to determine physician knowledge, attitudes, and

practices (KAPs) pertaining to diagnosis and reporting of AI and other notifiable and epidemic prone infectious diseases in Nigeria. **Methods**: Between November and December 2008, we surveyed 245 public sector physicians working in 6 Nigerian cities (Ibadan, Kano, Benin City, Maiduguri, Aba, and Abuja). Survey components included basic respondent demographics, reporting practices for AI and other diseases, obstacles to disease reporting and information sources used by the physicians. **Results**: All 245 respondents reported that they had heard of AI and that humans could become infected with AI. A majority correctly identified common symptoms (91%) and modes of transmission (89%). Nearly all participating physicians reported having a cell phone that they currently use (99%), with more than 99% sending text messages (SMS) at least weekly. Similarly, 94% reported having a current email address and 84% checked email at least weekly. Internet websites were reported as the single most useful source for learning about AI (26%). Most (67%) participating physicians had previously reported a notifiable disease; the most common disease reported was polio (42%).The most common perceived obstacles to reporting notifiable diseases identified were: lack of infrastructure/logistics or reporting system (31%); doctors not knowing how or to whom to report (26%); and doctors not knowing that they should report (24%). **Conclusions:** Despite widespread awareness of avian influenza, including common symptoms and modes of transmission, there was no consensus among physicians to whom they should report notifiable diseases, and physicians noted many obstacles to reporting. Since physicians reported widespread use of internet, email and cell phones, future improvements to the reporting system could benefit from increased utilization of the internet, as well as SMS and email-based communication.

**Board 95. On-Site Stool Culture Capacity Offers First Glimpse at Bacterial Pathogens Causing Diarrheal Disease in Remote Refugee Camp in Kenya**

**A.K. Tepo**[1], F.O. Oyier[1], S.A. Mowlid[1], E. Auko[1], I. Ndege[1], A.A. Hussein[2], W. Kabugi[2], M. Abdul Kader[2], W.J. Burton[3], J. Oundo[4], R. Nyoka[4], J.A. Ahmed[4], R.F. Breiman[4], R.B. Eidex[4];
[1]Kenya Medical Research Institute, Nairobi, Kenya, [2]International Rescue Committee, Nairobi, Kenya, [3]United Nations High Commissioner for Refugees, Nairobi, Kenya, [4]Centers for Disease Control and Prevention, Nairobi, Kenya.

**Background:** Diarrheal illnesses are a common cause of morbidity and mortality in children and adults in refugee camps. Frequent outbreaks of acute watery diarrhea, including at least two outbreaks of cholera, associated with inadequate clean water, poor sanitary conditions and overcrowding have been reported in Dadaab refugee camps. Stool culture is rarely available in refugee camp settings and sending specimens to a referral lab is costly and time-consuming, as a result the etiology goes undiagnosed. However, due to the risk of rapidly spreading and highly fatal epidemics of cholera or bacillary dysentery, the United Nations High Commissioner for Refugees, International Rescue Committee (IRC), Kenya Medical Research Institute and US Centers for Disease Control and Prevention established laboratory capacity for stool culture in Hagadera Camp, Dadaab. We review data from stool cultures conducted between May and September 2011. **Methods:** Stool specimens were collected from patients of any age presenting to health facilities in Hagadera, Dadaab with a history of diarrhea, defined as having ≥3 loose stools within 24 hours. Whole stool specimens were collected and culture for bacterial pathogens was performed on-site. **Results:** Between May and September 2011, stool specimens from 292 patients were cultured. The most common pathogens identified were *Shigella* spp (48; 16.4%), *Campylobacter* spp (20; 6.8%), and *Salmonella* spp (14; 4.8%). Eleven (22.9%) *Shigella* isolates were *S. dysenteriae*; one was *S. dysenteriae* type 1, the agent of bacillary dysentery. *Aeromonas hydrophyla*, *Escherichia coli*, and *Morganella morganii* were isolated from 11 (3.8%), 9 (3.1%), and 4 (1.4%) specimens respectively. *Vibrio* spp. was identified in 5 specimens; of which 3 were *V. cholerae* serogroup O1, serotype Inaba, the agent of cholera. **Conclusions:** It is useful to perform stool culture in some refugee settings. Highly pathogenic bacteria including *S. dysenteriae* type 1 and *Vibrio cholerae* O1 which are associated with large and sometimes explosive outbreaks were identified as causes of diarrheal

disease in a refugee population with no detectable ongoing epidemic. Early detection of these organisms can help strengthen prevention and response planning.

**Board 96. Progress in Use of the Web by State and Territorial Health Departments To Enhance Infectious Disease Surveillance—United States, 2003-2011**

**N.M. M'ikanatha**[1,2], T. McAdams[3], D.D. Rohn[4], R.M. Rivera[5], D.P. Welliver[6];
[1]Pennsylvania Department of Health, Harrisburg, PA, USA, [2]University of Pennsylvania Perelman School of Medicine, Philadelphia, PA, USA, [3]Minnesota Department of Health, St. Paul, MN, USA, [4]Maryland Department of Health and Mental Hygiene, Baltimore, MD, USA, [5]Franklin and Marshall College, Lancaster, PA, USA, [6]Clarific Services, Columbus, OH, USA.

**Background:** The emergence of the Web as a dominant source of information for healthcare providers warrants its consideration for use in infectious disease surveillance. Previous studies indicate some clinicians are unaware of why, what, where, how and when to report diseases. Our aim was to document progress and suggest areas for improvement in use of the Web to enhance surveillance for endemic and emerging diseases. **Methods:** During 2003, 2009, and 2011 we collected Web-based surveillance information in each of the 57 jurisdictions that submit data on nationally notifiable diseases to the Centers for Disease Control and Prevention (CDC). On each jurisdiction's Web site, we searched for practical information including mandate (rationale), a reportable diseases list (RDL), link to RDL on home page, case reporting form, and link to online reporting. For 2003 and 2009, we searched for presence of CDC BT Category A agents and telephone number for reporting. For 2009, we added items on novel pH1N1 influenza. **Results:** We found rationale for reporting in 74% of the Web sites in 2003 and 77% in 2011 while most sites had RDL (83% in 2003; 88% in 2011). Thirty-five percent of the sites had reporting forms in 2003 compared with 54% in 2011 while <10 had links to online reporting in both time periods. In 2003 and 2009, virtually all sites listed anthrax but <50% included viral hemorrhagic fevers (VHFs), and <90% (75-84%) included a telephone number for urgent reporting. We found that 90% of the jurisdictions' Web sites had links to pH1N1 on the home page, 74% displayed current data on influenza reported in the jurisdiction, and 67% included guidelines on submission of pH1N1 specimens. **Conclusions:** State and territorial health departments have taken advantage of the Web to provide information to improve reporting and recognition of evolving public health threats. The limited presence (<50%) of some important requirements (e.g., VHF) and lack of telephone numbers in <90% of the sites suggests need for improvements. An approach that includes assessment of Web content, healthcare providers' perspective and increased communication among subject matter experts and Web content designers could enhance the value of the Web as source of practical guidance to enhance surveillance including recognition of emerging threats.

## Laboratory Support

Monday, March 12
5:00 PM – 6:00 PM
Grand Hall

**Board 97. Untapped Potential: Negative and Non-Influenza Specimens in the DoD Global, Lab-based, Influenza Surveillance Program at US Air Force School of Aerospace Medicine (USAFSAM)**

**K. Tastad**[1,2], V.H. MacIntosh[1], L.V. Lloyd[1,3], J. Abshire[1,2], S. Zorich[1], L. Canas[1];
[1]United States Air Force School of Aerospace Medicine, Wright-Patterson AFB, OH, USA, [2]Henry M. Jackson Foundation for the Advancement of Military Medicine, Inc., Bethesda, MD, USA, [3]Oak Ridge Institute for Science and Technology, Oak Ridge, TN, USA.

**Background:** More than 70 DoD sites in over 40 countries have submitted respiratory specimens from patients with influenza-like illness (ILI) for testing by DoD Global, Lab-based, Influenza Surveillance Program at USAFSAM. This DoD surveillance system has existed since 1998. Typically over half of tests are negative for a respiratory virus. Positive, non-influenza viral results and negatives are valuable in tracking viral pathogens, evaluating surveillance methods and, potentially, in pathogen discovery. Further testing of negatives is being considered. **Methods**: Specimens are cultured for influenza A and B, adenovirus, enterovirus, HSV, parainfluenza (1-3) and RSV and tested with molecular methods for influenza. Original aliquots and isolates are stored frozen at -80$^0$C. Trends were evaluated using surveillance data back to 1998. Results for the 2010-2011 influenza season were analyzed according to three groups: influenza positive (IP), other virus positive (OVP) and no virus detected (NVD). Accompanying patient questionnaires with data on symptoms/severity were available for 43%. **Results**: Of 2,467 specimens (2010-11 season), 819 (33%) were IP; 309 (13%) OVP; and 1277 (52%) NVD. IP were most likely to meet the ILI case definition (71%) compared with 61% OVP and 49% with NVD. The NVD group was youngest (median age 6 years) compared to 20 and 21 years for IP and OVP. Active duty personnel with NVD were put on restricted duty nearly as often (63%) as those with influenza (70%) and more often than those with OVP (58%). Adenovirus comprised the greatest proportion of OVP (n=152, 49%), followed by RSV (n=80, 26%) and parainfluenza (n=70, 23%). There are 11,201 archived negative specimens available for future analysis. **Conclusions**: Archived specimens include over 11,000 high quality negative specimens that were submitted for ILI with known submission date, location, demographics, symptoms, severity and other relevant data. No virus was isolated in over half of specimens tested, leaving the diagnostic question unanswered. While the influenza system has been particularly useful for influenza and adenovirus surveillance, there is untapped potential for identifying pathogens responsible for significant illness in the military population. It is essential to monitor other pathogens by looking critically at negatives.

**Board 98. Molecular Characterization of *Vibrio parahaemolyticus* from Human Infection in Shenzhen City, China**

**Q. Hu**[1,2], Y. Wang[1], Y. Li[1], X. Shi[1], Y. Li[1], Y. Qiu[1], J. Mou[1], Y. Liao[1], H. Ma[1], J. Cheng[1];
[1]Shenzhen Centre for Disease Control and Prevention, Shenzhen, China, [2]School of Life Science, Shenzhen University, Shenzhen, China.

**Background:** To determine the occurrence and distribution of specific clones of pathogenic *V. parahaemolyticus* (VP) in Shenzhen City, Guangdong Province, China. Further, to assess the relationship of serotype O3:K6 to a globally distributed pandemic clone. **Methods:** A total of 1720 isolates recovered from diarrhea patients samples from 2007-2010 and foodborne outbreaks cases associated with VP from 2002-2010 were used in the study. All 1720 isolates were serotyped and real-time PCR was used to detect the virulence genes *tlh*, *toxR*, *tdh, trh* and Orf8 was detected in 68 different serotypes strains. The main serotypes were typed by pulsed field gel electrophoresis (PFGE). The strains with dominant serotypes and PFGE patterns were assayed by GS-PCR and toxRS sequencing to test whether they are pandemic clone. Multilocus sequence typing (MLST) analysis was reserved for exemplary 36 O3:K6 and 5 O4:K8 isolates. **Results:** Seventy-nine serotypes were observed among the 1720 isolates, including O3:K6 (59%), O4: K8 (10%), O1: KUT (4%), O1: K25 (3%), O3: K29 (3%). Comparing data after and before 2008, the percentage of O3:K29 increased from 0.03% to 6.71%, while O3:K6 and O4: K8 remained to be the top two serotypes. Most of strains (99.36%) were PCR positive for *tlh, toxR,* and *tdh* and eleven strains were *tdh* negative. Except for O4:K8, O4:K68, O1:K56, O9:k44, other 64 serotypes strains were positive for orf8. PFGE identified 55 patterns for a total of 519 isolates. For 331 O3:K6 strains, 34 PFGE pattern were observed, with which two patterns (27.5%) were being dominant and 15 clusters of isolates were found. Only 34 of 80 isolates were GS-PCR positive. MLST showed that all 36 O3:K6 isolates were ST3 and all 5 O4:K8 strains were ST189, matching the description of the pandemic VP clone. **Conclusions:**

Shenzhen has a recognizable burden of diarrheal illness caused by VP. Serotyping indicated that although there was a large variety of diversity, the dominant serotype was O3:K6. The VP isolates in Shenzhen were mainly *tdh* positive and *trh* negative, which is consistent with the current pandemic O3:K6 clone. The pandemic O3:K6 clone did appear to co-exist with other clones of O3:K6, as well as O4:K8, O1:K25. The potential outbreak of VP can be monitored in the laboratory-based surveillance. So prevention and control of VP should be priority in Shenzhen.

**Board 99. Contribution of the BacT/ALERT MB Mycobacteria Blood Bottle to Bloodstream Infection Surveillance in Thailand: Added Yield for *Burkholderia pseudomallei***

**P. Jorakate**[1], M. Higdon[1], A. Kaewpan[1], S. Makprasert[1], A. Lapamnouysup[1], S.A. Maloney[1], L.F. Peruski[2], H.C. Bagget[1];
[1]International Emerging Infections Program, Thailand Ministry of Public Health- US Centers for Disease Control and Prevention Collaboration, Nonthaburi, Thailand, [2]Global Disease Detection and Response Center - Central America US Center for Disease Control and Prevention, Guatemala City, Guatemala.

**Background:** We conducted active bloodstream infection surveillance in 2 rural Thailand provinces. Blood specimens were divided into 2 culture bottles: 1 optimized for aerobic growth (BacT/ALERT FAN® or F bottle) and a second for enhanced growth of mycobacteria and other fastidious organisms (MB bottle). Routine use of MB culture bottles is expensive, and incubation for up to 42 days strained incubator capacity. Cost containment issues and the unknown clinical contribution of MB bottles prompted this analysis. **Methods:** Blood cultures were collected as clinically indicated among hospitalized patients. Cultures were processed using the BactT/ALERT 3D automated culture system. We assessed the added yield of MB bottles by comparing the proportion of pathogens detected by MB vs. F bottles from 2005 -2010. **Results:** Of 5970 positive cultures for 58 clinically relevant pathogens, most common were *Escherichia coli* (31%), *Burkholderia pseudomallei* (12%), *Klebsiella pneumonia* (9%) and *Staphylococcus aureus* (7%). For all pathogens, 60% were identified in both F and MB bottles, 21% only in F, and 20% only in MB. Mycobacterial infections were detected only by MB bottles. Among patients with a pathogen detected, isolation of the pathogen was significantly more common from the MB bottle than from the F bottle for *Histoplasma capsulatum* (100% vs. 7.7%), *Penicillium* species (100% vs. 66%), and *Burkholderia pseudomallei* (91% vs. 74%) ($p<0.01$ for all). The association between bottle type and *B. pseudomallei* isolation was limited to patients pre-treated with antibiotics: among all patients with prior antibiotics, MB bottles were nearly twice as likely as F bottles to yield *B. pseudomallei* (1.5% vs. 0.83%, $p<0.001$). For most other pathogens, the proportion isolated from MB and F bottles was similar. **Conclusions:** For most pathogens, the proportion detected by MB bottles was similar to that detected by F bottles, suggesting the observed added yield of using 2 bottles is due to additional volume of blood cultured and not different growth media. However, for certain pathogens, MB bottles substantially improved detection. Targeted use of MB bottles is warranted for patients at risk for mycobacterial and fungal infections, as well as *B. pseudomallei*, a common cause of septicemia in Thailand.

**Board 100. Assessing Current Practices within the PulseNet USA Network: Data From APHL's 2010 PulseNet Survey**

**K.A. Kubota**, D.H. Kim, K. Watarida;
Association of Public Health Laboratories, Silver Spring, MD, USA.

**Background:** PulseNet, the national molecular subtyping network for foodborne disease surveillance was established in 1996 by the CDC, USDA, four state public health laboratories and APHL. PulseNet utilizes Pulsed-field Gel Electrophoresis (PFGE) as the method to subtype bacterial pathogens. In 2011, APHL fielded a capabilities and capacity survey to determine practices at the bench level and identify gaps within the current surveillance system. **Methods:** In May 2011, APHL conducted a survey related to PulseNet activities from January 1-December 31, 2010. The survey was sent to 68 public health and

agricultural laboratories through MR Interview survey tool. 59 laboratories responded for an overall response rate of 87%. In 2009, APHL launched a PulseNet survey to assess 2008 year activities. We included data comparisons from 2008 since some questions were similar. **Results:** Of the 59 laboratories, 50,266 STEC, *Salmonella, Listeria monocytogenes*, *Shigella* and *Campylobacter* isolates from clinical specimens were PFGE typed in 2010. This increased from 47,433 PFGE patterns submitted in 2008. PFGE typing increased for non-O157 STEC (49%), *L. monocytogenes* (21%), and *Salmonella* (12%) pathogens. A decrease in PFGE typing of *Shigella* isolates was observed (-42%). Compared to the number of clinical cases reported, most isolates from STEC cases were PFGE typed and uploaded to CDC. This is in contrast to the other pathogens: 95% for *Salmonella*, 88% for *L. monocytogenes*, and 37% for *Shigella,* respectively. Median turn-around-times (TATs) were calculated from receipt into the PFGE laboratory to pattern upload. Since 2008, laboratories maintained relatively level TATs in 2010 for STEC (3.5 days), *L. monocytogenes* (3 days), *Shigella* (5 days), and *Campylobacter* (4.5 days). A decrease was seen for *Salmonella* from 5 to 4 days. Since 2008, staff assigned to PulseNet activities remained relatively unchanged with an average of 1.7 FTEs. **Conclusions:** Overall, data show that laboratories within the PulseNet network increased their workloads using the same amount of personnel from 2008. PFGE typing continues to increase for many pathogens. TATs at state/local levels have remained the same for most pathogens although improvement was seen in *Salmonella* testing. Analysis of the data will continue to assess practices at the bench level.

## Early Warning Systems

Monday, March 12
5:00 PM – 6:00 PM
Grand Hall

**Board 101. Ecoclimatic Conditions Associated with the 2008–2011 Rift Valley Fever Activity in Southern Africa**

**A. Anyamba**[1], J. Small[1], K.J. Linthicum[2], R. Swanepoel[3], E.W. Pak[1], G. de Klerk[4], C.J. Tucker[1], J. Paweska[5], L. Blumberg[5], F.G. Benson[6], S.C. Britch[2], S. de La Rocque[7], P.B. Formenty,[8];
[1]NASA Goddard Space Flight Center, Greenbelt, MD, USA, [2]USDA-ARS Center for Medical, Agricultural and Veterinary Entomolgy, Gainesville, FL, USA, [3]University of Pretoria, Pretoria, South Africa, [4]Department of Agriculture, Forestry, and Fisheries (DAFF), Pretoria, South Africa, [5]NICD, Pretoria, South Africa, [6]Department of Health, Pretoria, South Africa, [7]Food and Agriculture Organisation of the United Nations, Rome, Italy, [8]World Health Organization, Geneva, Switzerland.

**Background:** The time period 2008-2011 has been marked by a series of Rift Valley fever (RVF) outbreaks in the Southern Africa region. Multi-year episodes of RVF have not occurred in the region since the mid-1970s. We examine climatic and ecological conditions associated with the outbreaks and present results of our prediction and risk assessments prior to each outbreak episode. **Methods:** We examined temporal and cumulative anomaly patterns in both rainfall and satellite derived normalized difference vegetation index (NDVI) for the entire growing season (September - May) of each year. These parameters have been previously demonstrated to be closely associated with RVF outbreaks in other regions of Africa. The anomalies in NDVI are used to drive a persistence model to map areas at potential risk of RVF activity. **Results:** Analysis results show that each outbreak was preceded by ~ 3 months of above normal and widespread rainfall and vegetation green up. Prediction and risk assessment results show an average warning of 1 to 3 months before the first case of RVF was identified. Outbreak locations are coincident with areas mapped to be at risk except for some areas in eastern South Africa and Madagascar that are outside the potential epizootic zone. The outbreaks represent 4 clusters of

outbreaks in spatially distinct areas especially over South Africa with NE-SW orientation defined by the progression of rainfall over the 4-year study period. **Conclusions:** Our results indicate that the multi-year RVF outbreak in Southern Africa was driven by teleconnections to the persistent cold *El Niño* Southern Oscillation (ENSO) / *La Niña* conditions that resulted in above normal rainfall conditions during the southern hemisphere summer months. However, the multi-focal patterns of outbreaks may be associated with the spatial variation of the immune status of the livestock-amplifying hosts, a factor that is not fully understood and possibly the concomitant vaccination campaign.

**Board 102. Clusters of Severe Viral Respiratory Infections among Hospitalized Patients Identified through National Influenza Surveillance, Bangladesh, 2009-2010**

**A.A. Mamun**[1], E. Azziz-Baumgartner[2], K. Sturm-Ramirez[1,2], R.U. Zaman[1], T. Azim[1], M. Rahman[1], A.S. M Alamgir[3], M.Z. Rahman[1], S. Nasreen[1], M.U. Bhuiyan[1], N. Homaira[1], M. Rahman[1], S.P. Luby[1,2];
[1]International Centre for Diarrhoeal Diseases Research, Bangladesh, Dhaka, Bangladesh, [2]Centers for Disease Control and Prevention, Atlanta, GA, USA, [3]Institute of Epidemiology, Disease Control and Research, Bangladesh, Dhaka, Bangladesh.

**Background:** During 2009-2010, we identified clusters of severe respiratory disease through a national influenza surveillance system in Bangladesh to identify respiratory pathogens with pandemic potential. Here we describe the epidemiology of clusters and assess the effectiveness of this cluster surveillance. **Methods:** Staff in each of the 12 surveillance hospitals identified potential clusters daily, defined as 2 or more in-patients with severe acute respiratory illness (SARI, aged ≥5 years with history of fever within 21 days and cough or sore throat) and/or severe pneumonia aged <5 years, (defined by WHO), who live within a 30 minute walk or within 3 kilometer radius and who develop symptoms within 7 days of each other. Teams administered a questionnaire and obtained nasal and throat swabs for rRT-PCR testing for influenza, respiratory syncytial virus (RSV), human metapneumovirus (hMPV), adenovirus and parainfluenza. **Results:** From May 2009-September 2010, we identified 125 clusters of 271 case-patients in 11 of 12 hospitals. Among these clusters, 69 (55%) had only severe pneumonia case-patients <5 years old, 31 (25%) had only SARI ≥5 years old, and 25 (20%) had both SARI and severe pneumonia case-patients. Most clusters, 108 of 125 (86%) comprised 2 case-patients. The largest identified cluster comprised 5 case-patients and two clusters comprised 4 case-patients. The mean number of clusters per month was 7 and. For 25 out of 125 clusters (20%), each of the case-patients tested positive for RSV alone. Of all case-patients 163 (60%) were positive for one of the tested respiratory viruses: 126 (77%) severe pneumonia and 37 (23%) SARI. The median age of case-patients infected with either RSV, hMPV, parainfluenza or adenovirus was 5 months as compared to 22 years for influenza ( $p<0.001$). The median time lag between cluster identification and availability of etiology was seven days. **Conclusions:** This cluster surveillance effectively identified clusters of severe viral respiratory disease but most were caused by common childhood pathogens. Based on these findings, we have refined the cluster surveillance system to focus on clusters that are more likely to represent emerging infections by implementing age-group specific cluster definitions and rapidly screening out common childhood pathogens in the laboratory.

**Board 103. Integrated Epidemiological and Remote Sensing Applications as an Early Warning System to Predict West Nile Virus Infection in the Northern Great Plains**

**T.-W. Chuang**, A. Lamsal, Y. Liu, M.C. Wimberly;
South Dakota State University, Brookings, SD, USA.

**Background:** The Northern Great Plains (NGP) has been a hotspot of West Nile virus (WNV) incidence since 2002. Mosquito ecology and the transmission of vector-borne disease and are influenced by multiple environmental factors, and climatic variability is one of the most important drivers. An early warning system (EASTWeb) was established to integrate epidemiological and satellite remote sensing

data forecast climatic risk factors for WNV epidemics in the NGP. This system automatically acquires and processes satellite remote sensing imagery from a variety of sources and generates output in customized formats for further analysis and mapping. **Methods:** The system produces a variety of environmental metrics including land surface temperature (LST) and the normalized difference vegetation index (NDVI) derived from Moderate-Resolution Imaging Spectroradiometer (MODIS) products. We also used these inputs to model actual evaportranspiration (ETa) with the simplified surface energy balance method. These environmental metrics are sensitive to seasonal and interannual fluctuations in temperature and precipitation, and are hypothesized to influence mosquito population dynamics and WNV amplification. Non-linear generalized additive models (GAMs) were used to evaluate the influences of cumulative LST, NDVI, and ETa anomalies on interannual variations of WNV incidence from 2004-2010. **Results:** The best-fitting model incorporated the early onset of spring (indicated by NDVI) and late spring/summer of accumulated moisture and temperature (ETa and LST), highlighting the influences of climatic variability on WNV amplification and transmission to humans. The risk forecasting map indicated a low WNV risk across the NGP in 2011, which is concordant with the low case reports in this year. **Conclusions:** Our system provides tools and products to facilitate the acquisition and analysis of remote sensing data for epidemiological research and enhance the efficiency of disease surveillance and prevention.

## H1N1 Influenza

Monday, March 12
5:00 PM – 6:00 PM
Grand Hall

**Board 104. Isolation, Antigenic Analysis, and Some Biological Properties of Human Influenza Viruses Circulated in Russia in the Epidemic Seasons 2009–2011**

**M. Eropkin**, D. Baibus, N. Konovalova;
Research Institute of Influenza, Saint-Petersburg, Russian Federation.

**Background:** Influenza etiological situation in Russia in the 2 last epidemic seasons. **Methods:** Virus isolation, antigenic and genetic analysis of isolates. **Results:** During the first wave of pandemic influenza in Russia (from the 46$^{th}$ week of 2009 to the 2$^{nd}$ week of 2010) we isolated 417 strains of pandemic influenza A(H1N1)pdm09 what composed 92 % of all influenza virus strains isolated at our laboratory in the epidemic season 2009-2010. All Russian pandemic strains were antigenically homogeneous and reacted with WHO diagnostic antiserum as well as with antisera obtained at our laboratory to the reference strains A/California/07/09 and A/St.Petersburg/56/09 up to 1-1/4 of homologic titer. HAI also showed a high degree of homology with the old H1N1 strains of swine origin - A/New Jersey/8/76, A/SW/1976/31, A/Iowa/15/30. The 2010-2011 influenza epidemic in Russia was characterized by the high level of the circulation of pandemic virus A(H1N1)pdm09, comparable to the level of previous season (57.7 % of isolates) with the simultaneous participation in the epidemic process of influenza B of Victorian lineage (41.3 %) and very small proportion (about 2 %) - of the sub-type A(H3N2). The strains A(H3N2) and B, isolated in Russia, underwent a significant antigenic drift as compared to the previous epidemic season and formed the evolutionary advanced groups. Opposite to that, pandemic influenza strains did not undergo any essential antigenic changes. Besides that, we did not find on the phenotype level any particularities in the pandemic strains isolated from post-mortem materials. According to its biological properties the pandemic virus A(H1N1)pdm09 can be attributed to the moderately pathogenic strains. All studied strains contained the S31N substitution in M2 protein, which determines resistance to the adamantane antivirals and had no H275Y substitution in neuraminidase, which determines

resistance to oseltamivir. The substitution of amino acid residue Asp to Gly at position 222 of HA was found in eight from eleven isolates (73%) from postmortem lung and trachea samples, and two from twenty isolates (10%) from patients' nasopharyngeal swabs. **Conclusions:** We did not observe any significant antigenic drift as well as a growth of pathogenicity in the pandemic H1N1 pdm09 virus during two last epidemic seasons, while new circulating variants of H3N2 and B viruses were significantly different from previously circulated strains.

**Board 105. Serial Cross-Sectional Serological Surveys Conducted in Medical Institutions to Understand the Trend in the Seroprevalence of Pandemic Influenza H1N1 (2009) Virus Infection in China**

**C. Xu**;
Chinese National Influenza Center, Beijing, China.

**Background:** We conducted four serological surveys in the same hospitals from all 31 provinces to monitor age-specific seroprevalence of 2009 pandemic influenza A(H1N1) (2009 H1N1) in different time points during the pandemic wave. **Methods:** The first serological survey was performed in early December 2009, and other 3 surveys were performed monthly during January to March 2010. A total of 43,172 serum samples were obtained from individuals who reported not receiving the 2009 H1N1 vaccine. Antibody response to 2009 H1N1 was measured using haemagglutination inhibition assay. $X^2$ test($P<0.05$) and the multiple comparison($P<0.0011$) was used to compare the difference in seroprevalence of 2009 H1N1 by age group in the four surveys and a published baseline serological survey. **Results:** There were statistically significant increases in overall seroprevalence between baseline(1.2%) and early December(7.6%), between early December and January(18.6%), and between January and February(20.5%) respectively. For individuals younger than 60 years, the statistically significant increases in seroprevalence were observed from baseline to early December, and from early December to January. For individual aged ≥60 years, a statistically significant increase in seroprevalence occurred from early-December to January ($P < 0.001$). In all the four surveys, the seroprevalence was the highest in 6-15 and 16-24 years. **Conclusions:** These serological surveys indicated the overall seroprevalence sharply increased from early December 2009 to February 2010, and plateaued thereafter. The seroprevalence increased quicker in individuals younger than 60 years compared with individuals older than 60 years. School-aged children and young adults had an important role in transmission of 2009 H1N1.

**Board 106. Influenza A Virus Nucleocapsid Protein Interacts with a Cytoskeleton Scaffolding Protein b (beta) Actinin-4 to Regulate Viral Replication and Transcription**

**S.K. Lal**[1], S. Sharma[1], A.K. Mayank[1], S. Tripathi[1], J.R. Patel[2], P. Gaur[1], J.M. Katz[2], N.J. Cox[2], R.B. Lal[2], S. Sambhara[2];
[1]International Centre for Genetic Engg. and Biotechnology, New Delhi, India, [2]Centers for Disease Control and Prevention, Atlanta, GA, USA.

**Background:** The human host cellular machinery is exploited by the influenza virus for its survival and replication inside the host cell. To discover host cellular proteins that interact with viral proteins, we screened a human lung cDNA library using the yeast two-hybrid system with NP as bait and found that α-Actinin-4 as one of the cellular proteins interacting with NP. **Methods:** In vitro coimmunoprecipitation of NP and Actinin-4 coexpressed in HEK 293 cells confirmed a direct interaction between these proteins. The interaction was further validated in influenza A virus infected lung epithelial A549 cells. **Results:** Interestingly, the NP-Actinin-4 interaction was observed to be conserved across several subtypes of influenza A virus, including the 2009 H1N1 pandemic virus. Small interfering RNA knockdown of Actinin-4 expression resulted in a significant decrease of influenza A/PR8/34 virus NP and NS1 protein expression as well as a significant reduction in viral titers, 24 hrs post-infection suggesting that Actinin-4 was required for viral replication. Moreover, immunofluorescence studies revealed that both NP and α-

Actinin-4 co-localized in the perinuclear region of IAV infected A549 cells as well as in HEK293 cells transiently expressing NP. The PI3K/Akt signaling cascade is activated upon influenza A virus infection leading to the phosphorylation of IRF-3, NF-κB, and AP-1 which subsequently activate specific target genes and promotes viral survival and replication. Silencing Actinin-4 followed by influenza virus infection resulted in the downregulation of PI3K and Akt signaling molecules along with increased cell death. **Conclusions:** Taken together, our findings indicate that α-Actinin-4 is an interacting partner of influenza virus NP and this interaction is essential for viral replication.

**Board 107. Viral Etiology of Acute Lower Respiratory Infections in Hospitalized Children below 1 Year of Age in Southwest India**

**G. Arunkumar**, C. Uma, S. Sampada, A. Sanyal, J. George;
Manipal Centre for Virus Research (ICMR Virology Lab-Grade-I & Regional Reference lab for Influenza Virus), Manipal University, Manipal, India.

**Background**: Acute lower respiratory infection (ALRI) is the leading cause of morbidity and mortality among young children worldwide. The data on viral etiology of ALRI from India are sparse. This study attempts to identify the viral etiology of ALRI in children less than one year old in southwest India. **Methods**: Children less than 1 year of age hospitalized in various districts of Kerala and Karnataka States along the southwestern coast of India during June - September, 2011 (monsoon season) formed the study group. These cases were consecutively selected from the samples received as part of the passive surveillance for the pandemic Influenza A/ H1N1/2009. The nasopharyngeal and or throat swabs were tested for viruses using a multiplex real time PCR assay (FTD Respiratory 21 plus, Fast track diagnostics, Luxembourg). The viruses screened include influenza A/B, pandemic influenza A/H1N1, respiratory syncytial virus(RSV), rhinovirus(RhV), adenovirus (AV), parainfluenza viruses 1-4, enterovirus (EV), parechovirus (PV), human metapneumovirus (Hmpv), human bocavirus (Hbov), and human coronaviruses (Hcov- 229E, Hcov-OC43, Hcov -NL63, Hcov-HKU1). **Results**: Of the 89 children with ALRI, 60 (67.4%) had evidence of viral infection. RSV was the most frequent virus, detected in 37 cases (41.6%), followed by Hbov (14.6%), rhinovirus and Hmpv (both 9%), adenovirus (4.5%), enterovirus (3.4%), parainfluenza virus-1 (2.3%), and influenza A, influenza A/H1N1, Hcov-OC43, Hcov-HKU1, parainfluenza-2, and parainfluenza-3 (all 1.1%). While 44 cases (73.3%) yielded single virus, 18.3% had dual viruses and 8.3% triple viruses. Most of the Hbov(69.2%) and Hmpv (66.7%) were associated with dual or triple virus infection. Nineteen children (21.6%) had no identifiable virus in their samples. **Conclusions:** More than two-thirds of the children in their first year of life with ALRI had viral etiology; RSV was the most common, followed by Hbov and Hmpv. Influenza virus was associated with only 2 cases in spite of the influenza season in southwest India. This is the first comprehensive report on virological work up on ALRI in young children from this part of the world.

## Policy Implications and Infectious Diseases

Monday, March 12
5:00 PM – 6:00 PM
Grand Hall

**Board 108. Economic Burden of Bacteremic Melioidosis in Eastern Thailand**

**S. Bhengsri**[1,2], J. Lertiendumrong[3], H.C. Baggett[1], S. Thamthitiwat[1], S. Naorat[1], P. Jorakate[1], S. Chantra[4], K. Tisayaticom[3], S.A. Maloney[1];
[1]International Emerging Infections Program, Thailand MOPH-US Centers for Disease Control and Prevention Collaboration, Nonthaburi, Thailand, [2]Department of Tropical Hygiene, Faculty of Tropical

Medicine, Mahidol University, Bangkok, Thailand, [3]International Health Policy Program, Ministry of Public Health, Nonthaburi, Thailand, [4]Sa Kaeo Crown Price Hospital, Sa Kaeo, Thailand.
**Background:** Melioidosis, a severe disease caused by *Burkholderia pseudomallei*, is among the most common causes of septicemia in Thailand and results in substantial costs. Data on economic impact are lacking, especially from regions outside of the highly endemic Northeast. We describe the economic impact of bacteremic melioidosis in Eastern Thailand. **Methods:** Bacteremic melioidosis cases during 2006-2008 were identified through bloodstream infection surveillance in all hospitals in Sa Kaeo (SK). Using hospital charge data from the National Health Insurance database, we estimated direct medical costs related to hospitalization by applying a cost-to-charge ratio of 0.88. Transportation costs were calculated from National Health and Welfare data. Indirect costs included productivity loss for caregivers and patients during hospitalization, and for fatal cases, future productivity loss due to premature death, calculated by multiplying time lost by the average monthly household income. Each patient was assumed to have 1 caregiver during hospitalization. The consumer price index was used to adjust costs to present value. Costs are presented in US dollars (1$=30.49 Thai Baht) and as means ± SD. **Results:** Seventy-three bacteremic melioidosis patients were identified in SK (average annual incidence = 4.6/100,000 persons, 95% CI=3.9-5.5); 39 (53%) patients were discharged in improved condition, 27 (37%) died, and 7 (10%) were transferred with unknown outcome. Fifty-two of 73 (71%) patients had cost data available. The average total cost/fatal case was $16,305 (direct medical: $1,963 ± 2,790, transportation: $15, productivity loss from morbidity [$115 ± 181] and mortality [$14,212 ± 9,535]: $14,327), which far exceeded that of non-fatal cases ($1,852; direct medical: $1,577±1596, transportation: $15, productivity loss from morbidity: $260±137). The average total cost of bacteremic melioidosis in SK was $145,102/year (direct medical costs: 20% [$29,916], transportation: 0.2% [$260] and productivity loss: 79% [$114,926]). **Conclusions:** In addition to the high disease burden, melioidosis has substantial economic impact, even in an area not previously known to be endemic. Early detection and treatment is required to prevent premature mortality, which accounts for the greatest economic losses.

**Board 109. Examination of Risk Factors for Recurrence of *Clostridium difficile* Infection in Selected New Haven County, Connecticut Towns**

**B. Paccha**, J. Hadler, C. Lyons, J. Meek, R. Heimer;
Connecticut Emerging Infections Program, Yale School of Public Health, Yale University, New Haven, CT, USA.
**Background:** Symptomatic *Clostridium difficile* infection (CDI) is commonly preceded by antibiotic use and is frequent in health care settings. In the last decade, with the emergence of more toxigenic strains, both hospital- and community-onset CDI have been a growing concern. Since late 2009, the Connecticut Emerging Infectious Program has been conducting active, laboratory-based surveillance for CDI in the metropolitan areas of two cities in New Haven County. This study aims to identify risk factors for CDI recurrence among cases initially diagnosed in the surveillance area from January - December 2010. **Methods:** Cases were surveillance area residents ≥1 year of age with an initial positive CDI lab test. They were classified as health care facility onset (HCFO, onset >3 days after hospital or long term care facility (LTCF) admission), community onset-HCF-associated (CO-HCFA, onset within 3 months of hospital discharge, outpatient surgery or LTCF residency) or community associated (CA). Recurrent (R) cases were those with ≥1 positive CDI lab test 2-8 weeks after the initial test. Chart review was done for all incident CO-HCFA and CA cases and for 1:10 HCFO cases. Incident case characteristics of R cases were compared to non-R cases. **Results:** In 2010, 826 unique persons had an initial incident episode of whom 130 (16%) had ≥1 recurrence. R cases were significantly older (75.1 vs 69.9 years), more likely to reside in a LTCF (53 vs 40%) and to use H2 blockers (10 vs 3%) than non-R cases. Differences between R and non-R cases for the healthcare associated group (HCFO+CO-HCFA) were similar to those for all cases.

Among the CA group, the R cases, in addition to being significantly older (68.9 vs 56.6 years), were more likely to have used immunosuppressants (36 vs 13%), but less likely to have used antimicrobials (73 vs. 43%). **Conclusions:** Recurrent CDI is more common among the elderly and those coming from LTCF. Only use of H2 blockers appears to distinguish recurrent from non-recurrent cases. CA cases arising without antecedent antibiotic use are also at higher risk of recurrence. Further investigation is needed in this group to determine if they harbor more toxigenic strains. Important limitations to our study include the use of a non-clinical definition of recurrence, and no treatment data for the initial CDI diagnosis.

**Board 110. Analysis of Sentinel Surveillance of Severe Acute Respiratory Infections (SARI) in Uralsk, Republic of Kazakhstan, in 2008–2011 Epidemic Seasons**

**G.K. Naurzalina**;
Uralsk State Sanitary and Epidemiological Surveillance Authority, Uralsk, Kazakhstan.

**Background:** Determining in whom influenza occurs and the viral types causing influenza assists in determining immunization need, devising immunization strategies, and contributes to global influenza knowledge. Sentinel surveillance of severe acute respiratory infections (SARI) began in 3 sentinel hospitals of the major city of western Kazakhstan in 2008 and we report epidemiologic and viral detection results for all three years. **Methods:** Nasal and throat swabs were collected from each patient > 1 year of age who met the WHO SARI standard case definition and with symptoms $\leq$ 72 hours. Influenza virus was detected by real-time PCR in the oblast virology laboratory. **Results:** Two hundred eighty patients were recorded in the 2008-2009 epidemic season. Children 1-4 years-of-age accounted for 75% (N=210), and children 5-14 years, 16% (N=45) of cases. Influenza A virus began to actively circulate in week 52 of 2008, and influenza B virus in week 8 of 2009. Influenza virus was detected in 116 (41%) of 280 tested patients, with influenza A detected in 56 patients (48%) and influenza B in 60 patients (52%). 50 patients had seasonal influenza A/H1N1, two patients had H3N2; and influenza A was not subtyped in 4 patients. Seventy patients were tested in the 2009-2010 season. Children 1-4 years-of-age accounted for 73% (N=51). In 22 (31%) of 70 tested patients, only influenza A virus was detected (subtyping was not performed). One hundred fifteen patients were recorded in the 2010-2011 epidemic season. The percentage of children of 1-4 years of age was 80% (N=92). Influenza A virus actively circulated in week 46 of 2010, influenza B virus in week 7 of 2011. 26 of 115 (23%) patients had influenza virus detected, with influenza B detected in 8 patients and influenza A in 18; 14 of them had influenza H3N2, 3 had influenza A/H1N1-09 and one patient had untypeable influenza A. **Conclusions:** Children 1-4 years-of-age are highly affected by influenza, making up as much as 80% of those with SARI over the last three seasons. The beginning of the epidemic season is characterized by circulation of influenza A virus, while influenza B virus appeared at the end of the season.

**Board 111. Implications of Screening Definitions for Estimation of Influenza Disease Burden**

**A. Krishnan**[1], V. Gupta[1,2], S. Rai[1], S. Dhakad[3], V. Raghuram S[3], M.-A. Widdowson[4], R. Lal[4], S. Broor[1];
[1]All India Institute of Medical Sciences, New Delhi, India, [2]The Inclen Trust, New Delhi, India, [3]National Institute of Virology, Pune, India, [4]Centers for Disease Control and Prevention, Atlanta, GA, USA.

**Background:** Surveillance data can be used to estimate influenza disease burden. However the choice of clinical definition used, often based on efficiency considerations, has implications for estimation of disease burden. Using data from health facility-based surveillance of all acute medical admissions from rural Ballabgarh in north India, we compared the influenza prevalence estimated using different currently-used surveillance definitions. **Methods**: Inpatients with acute medical illnesses from the selected 33 health facilities were enrolled and tested for influenza. Physicians assessed all subjects for influenza-like illness (ILI- fever plus cough/sore throat), acute respiratory infection (ARI- any symptom among cough/ earache/ nasal discharge/difficulty breathing / sore throat in >2 years), febrile acute respiratory illness (FARI- fever plus ARI) and severe acute respiratory infection (SARI- ILI plus fast

breathing in ≥5 years; IMCI-defined pneumonia or severe pneumonia in <5 years). The prevalence for each definition was estimated as the number who would have been found positive if only those meeting the definition were tested divided by the total eligible population. Efficiency was measured by numbers needed to test (NNT) calculated separately for each definition as the ratio of samples tested to the number detected positive. **Results:** Of the 1,043 subjects tested from August 2009-July 2011, 74 (7.1%) tested positive for influenza. Out of 1,043, 906 (86.9%) satisfied the case definition of ARI, 449 (43.0%) for ILI, 503 (48.2%) for SARI, and 479 (45.9%) for FARI. The influenza prevalence was 6.7% for ARI, 5.8% for FARI, 5.6% for ILI, 5.1% for SARI. The NNT were 7.7 for ILI, 7.9 for FARI, 12.9 for ARI, and 9.5 for SARI. The ARI prevalence (6.7%) was closest to the true prevalence (7.1%) but was least efficient (NNT 12.9). ILI was most efficient (NNT 7.7) but gave a lower prevalence estimate (5.6%). **Conclusions:** The choice of screening definition used for influenza surveillance among inpatients greatly influences the efficiency of surveillance and also impacts burden estimates. Researchers using surveillance data for estimation of burden need to be aware of these differences. *This study was supported in part by cooperative agreement U01 IP000206 from the Centers for Disease Control and Prevention, Atlanta, USA.*

## Modeling

Monday, March 12
5:00 PM – 6:00 PM
Grand Hall

**Board 112. Development of Multi-Criteria Decision Analysis Tools to Assess the Risk of Emergence or Re-emergence of Infectious Diseases Associated with Climate Change in Canada**

**R. Cox**, C. Revie, J. Sanchez;
University of Prince Edward Island, Charlottetown, PE, Canada.

**Background:** Policy decision makers require rational methods to prioritise emerging infectious diseases and to identify those that warrant detailed investigation. **Method:** We used expert opinion to determine the criteria that could be used to prioritise diseases according to the likelihood of emergence in Canada in response to climate change. Multi Criteria Decision Analysis (MCDA) was used to develop two different tools that could be used to rank diseases based on the collected expert opinion. These were an Excel spreadsheet tool and a model developed using the MCDA software 'MACBETH'. The models were tested on a few example diseases. **Results:** We identified a total of 40 criteria that might be used for prioritisation. A weight was applied to the criteria based on the opinion of 64 experts. Some of the criteria that were considered most influential in disease prioritisation were: potential economic impact, severity of disease in the general human population, human case fatality rate and the type of climate that the pathogen can tolerate. Some of the climate criteria that were most likely to influence disease emergence were: an annual increase in temperature, an increase in summer temperature and an increase in summer precipitation. These climate variables were considered to be most influential on vector borne diseases. In the Excel spreadsheet the diseases that tended to rank the highest were West Nile virus and Giardia. In the MACBETH model *Giardia* ranked the highest and Coccidioidomycosis ranked the lowest. **Conclusions:**The spreadsheet tool used a simple weighting average to rank disease criteria and proved a user friendly approach to prioritisation. The MACBETH model is a practical graphical tool that allows the user to make qualitative judgements about criteria to rank diseases. Although our research applied specifically to Canada, the methods can be adapted to alternative situations.

**Board 113. Predicting Transmission of Avian Influenza Virus in Wild Birds from Incomplete Epidemic Data**

**V. Henaux**[1], M.D. Samuel[2], J. Parmley[3], C. Soos[4];
[1]Department of Forest and Wildlife Ecology, University of Wisconsin, Madison, WI, USA, [2]U. S. Geological Survey, Wisconsin Cooperative Wildlife Research Unit, Madison, WI, USA, [3]Canadian Cooperative Wildlife Health Centre, University of Guelph, Guelph, ON, Canada, [4]Environment Canada, Saskatoon, SK, Canada.

**Background:** Wild aquatic birds play an important role in the maintenance and spread of all low pathogenic avian influenza viruses (LPAIv), which provide the precursor for the development of highly pathogenic (HP) AIv affecting domestic animals and humans. However, there is currently little scientific information about the epizootiology of AIv in wild birds. Based on published laboratory studies, we previously developed epidemic models ("Susceptible-Exposed-Infectious-Recovered") which described the course of LP and HPAIv infection in individual hosts. However, predicting AI dynamics in free-ranging populations remains challenging because LPAIv data from field studies are usually incomplete and HPAIv outbreaks are rare, typically discovered after epizootiological dynamics have occurred and/or affected birds have migrated. **Methods:** We used a maximum likelihood approach to estimate the rate of AIv infection in wild waterfowl populations by fitting the AI epidemic models to disease surveillance data. **Results:** Using weekly prevalence data in hatch-year birds at post breeding and migration stopover sites in North America, we predicted probabilities of LPAIv infection ranging from 0.07 to 0.27 day$^{-1}$. We found lower daily probabilities of infection in the Pacific flyway (0.05-0.07), high probabilities in prairie Canada (0.21-0.27), and variable probabilities in eastern Canada (0.02-0.18). Using the mean LPAIv infection rate to predict HPAI, we estimated a shorter and lower epidemic curve for HPAI (5 weeks with peak prevalence of 29%) than LPAI (7 weeks with peak prevalence of 59%). This finding indicates it is harder to detect HPAIv than LPAIv with swabs from live birds, which are commonly used during surveillance programs. **Conclusions:** Our study highlights the potential of integrating incomplete surveillance data with epizootic models to estimate rates of disease transmission and evaluate epizootic dynamics. Such information is critical for predicting the risk of spread to domestic birds and informing surveillance and disease control programs.

**Board 114**. **Predicting the Distribution of *Culex tritaeniorhynchus*, the Primary Vector of Japanese Encephalitis Virus, Using Ecological Niche Modeling**

R. Miller[1], **P. Masuoka**[1], T.A. Klein[2], H.-C. Kim[3], J.-Y. Yeh[4], J. Grieco[1];
[1]Uniformed Services University, Bethesda, MD, USA, [2]65th Medical Brigade,US Army MEDDAC, Seoul, Republic of Korea, [3]5th Medical Detachment, US Army, Seoul, Republic of Korea, [4]National Veterinary Research and Quarantine Service, Anyang, Republic of Korea.

**Background:** The principal vector of Japanese encephalitis virus (JEV) is *Culex tritaeniorhynchus*. The primary breeding habitats of this mosquito include low-lying grassy areas that are prone to flooding and flooded rice fields. The virus is maintained in large wading birds, while swine (domestic and wild) serve as amplifying hosts. While vaccination programs have greatly reduced the incidence of JE in some countries (e.g., Republic of Korea and Japan), complacency, fear of vaccines and inadequate vaccination programs in other countries have left many populations susceptible to infection. Ecological niche models of JEV vector(s) can aid in disease risk assessment and elucidate factors that affect mosquito distribution. **Methods:** The purpose of this study was to create an ecological niche model to predict the distribution of *Cx. tritaeniorhynchus* in Asia. The model was built using mosquito location data from the literature and from Mosquito Map (mosquitomap.org). Environmental data (precipitation, temperature, elevation) were obtained from WorldClim (worldclim.org). Maxent software (http://www.cs.princeton.edu/~schapire/maxent/) was used to build the model and create probability maps of the distribution of *Cx. tritaeniorhynchus*. Maps to compare to the model were obtained for

swine and rice fields. JE case data were obtained from ProMED reports (www.promedmail.org) from 1994 to 2010 and reports from the Korean Centers for Control and Prevention (K-CDC). **Results:** Statistical analysis revealed that the *Cx. tritaeniorhynchus* model predicted vector presence significantly better than a random distribution. The environmental variable that had the most impact on the model was the mean temperature of the wettest quarter. Comparisons with rice and swine distribution maps showed similarities to the predicted distribution of *Cx. tritaeniorhynchus*. Seventy two percent of JE case locations had a greater than 0.25 predicted probability for the presence of *Cx. tritaeniorhynchus*. Analysis of the distribution map found that seven countries have greater than 50% of their land area with a high probability of *Cx. tritaeniorhynchus* presence. **Conclusions:** The current ecological niche model could assist countries in planning vaccination programs and other preventive measures that reduce the risk of JEV infections.

**Board 115. Using Host Population Structure to Forecast the Spread of Rabies in Northern Ohio**
**S. Wisely**[1], A. Berensten[2], A. Gregory[1], M. Dunbar[2];
[1]Kansas State University, Manhattan, KS, USA, [2]USDA APHIS Wildlife Services, Ft. Collins, CO, USA.
**Background:** In the United States, unique rabies strains are maintained in distinct wildlife species, including the raccoon (*Procyon lotor*). Raccoon variant rabies (RVR) has spread throughout the eastern US and is moving west. To combat the westward spread of the disease, oral rabies vaccines (ORV) are distributed along natural barriers that may restrict raccoon movement, such as mountain ranges and rivers. The spread of rabies into Ohio is prevented by an ORV program in the eastern portion of the state and utilizes the mountains of western Pennsylvania; however, in 2004 a rabid raccoon breached the barrier. **Methods:** To better understand and control the spread of rabies into northeast Ohio from Pennsylvania, we used multilocus genetic tools and landscape resistance modeling to investigate dispersal dynamics and land use patterns within the urban footprint of northeastern Ohio. These results can be used to forecast the potential spread of RVR and design control and ORV programs. **Results:** We collected genetic samples from 317 raccoons in 8 counties in and around Cleveland, Ohio. We found high levels of diversity and low levels of genetic structure (global $F_{ST} = 0.01$) in the population suggesting that this population is large, highly admixed, and mobile. We did not find evidence of sex-biased dispersal, but found spatial structuring of kin groups. Two populations (metropolitan Cleveland and rural outskirts) separated by 100 km exchanged 5 migrants over 1 to 2 generations. **Conclusions:** Isolation by resistance modeling suggested greenbelts within the urban footprint may provide enhanced dispersal opportunities that maintain genetic diversity, but could be effective targets where ORV distribution could be concentrated. More detailed analysis of population density is warranted to determine the quantity of ORV baits needed to vaccinate a sufficient proportion of the host population.

**Board 116. The Impact of the Definition of the Symptom-free Interval on Illness Duration and Incidence in Longitudinal, Household Morbidity Surveillance in Rural Western Kenya**
**A.O. Audi**[1,2], D.R. Feikin[1,2], G.M. Bigogo[1,2], B.O. Nyawanda[1,2], J. Williamson[2], R.F. Breiman[1,3], D.C. Burton[1,2];
[1]International Emerging Infections Program-Kenya, Division of Global Disease Detection and Emergency Response, Centers for Disease Control and Prevention (CDC)-Kenya, Kisumu and Nairobi, Kenya, Kisumu, Kenya, [2]Kenya Medical Research Institute/CDC Research and Public Health Collaboration, Kisumu and Nairobi, Kenya, Kisumu, Kenya, [3]Kenya Medical Research Institute/CDC Research and Public Health Collaboration, Kisumu and Nairobi, Kenya, Nairobi, Kenya.
**Background:** In sub-Saharan Africa, longitudinal household-based morbidity surveillance can be used to characterize the epidemiology of infectious diseases. However, with continuous symptom monitoring, there is a need to define a symptom-free interval to distinguish separate illness episodes. The selection of the symptom-free interval may influence estimates of disease burden and mean duration. The longer

the required symptom-free interval, the more likely that 2 discrete illness episodes would be classified as 1 episode. **Methods:** From July 1, 2006 to December 31, 2010, we collected symptom data weekly or biweekly from approximately 25,000 individuals participating in population-based surveillance in rural western Kenya. Standard questionnaires were administered at homes to gather data on the onset and duration of respiratory (cough), febrile (reported fever), and diarrheal (≥3 loose stools in a 24-hour period) illnesses. We used Poisson regression to evaluate the impact on incidence of varying the required symptom-free interval from 1 day to 7 days. **Results:** When the required symptom-free interval increased from 1 day to 7 days, the mean duration of diarrhea episodes increased from 3.2 to 5.5 days in children < 5 years, and from 2.7 to 4.6 days in persons ≥ 5 years. Mean duration of respiratory and febrile symptoms likewise increased from 4.9 to 9.4 days and from 2.9 to 4.5 days, respectively, for children < 5 years, and increased from 5.2 to 11.0 days and from 3.1 to 5.0 days, respectively, in persons ≥ 5 years. For each illness syndrome, incidence was higher when using a shorter symptom-free interval. When using a 1-day rather than a 7-day symptom-free interval, the incidence of febrile, diarrheal, and respiratory illness increased 43% (95% CI 42-44%), 54% (95% CI 52-56%), and 75% (95% CI 73-76%), respectively, among children < 5 years. For persons ≥ 5 years, the incidences increased 47% (95% CI 46-48%), 51% (95% CI 49-52%), and 94% (95% CI 92-96%), respectively. **Conclusions:** The number of symptom-free days used to define a new illness episode has substantial impact on estimates of mean illness duration and incidence from longitudinal infectious disease surveillance. Standardization of symptom-free days in case definitions is important for comparability of data from studies defining infectious disease burden.

**Board 117. Surrogate Epidemic-Evolution: Models for Real-time Outbreak Characterization**
**J. Ray**[1], C. Safta[1], S. Lefantzi[1], K. Cheng[2], D. Crary[1];
[1]Sandia National Laboratories, Livermore, CA, USA, [2]Applied Research Associates, Inc., Arlington, VA, USA.
**Background:** Characterization of epidemics, e.g., estimation of a time-dependent spread rate, conditioned on syndromic surveillance data available in the early epoch of an outbreak, can be useful for crisis management and resource planning. Probabilistic estimation can address the problem of sparse data, but the computational expense, especially when using accurate epidemic-evolution models, make the approach infeasible for real-time use. **Methods:** We cast the problem of characterization of an outbreak as a Bayesian inverse problem, predicated on time-series data. The detailed epidemic model is constructed by convolving distributions for the incubation period and the visit delay. The resulting model has 5 unknown parameters, characterizing a time-dependent disease spread rate, the final epidemic size and start time of the outbreak. We then create its (parametrized) surrogate using polynomial chaos expansions. Such expansions (basis functions) are novel in medicine but commonly used in some engineering disciplines. We use an adaptive Markov chain Monte Carlo method to fit the model to the data, and obtain parameter estimates as distributions. **Results**: We tested our characterization technique on data from the 1918 influenza pandemic from Camp Custer, MI. We find that the surrogate model reduces the computational time by a factor of 50, to around 10 minutes per characterization. Further, 7-14 days of data are found sufficient to obtain estimates with sufficient specificity for crisis management and response. The error in the characterization resulting from the use of the surrogate, vis-a-vis the detailed model is about 5-10%. **Conclusions:** Surrogate models are a feasible means of accelerating outbreak characterization from syndromic surveillance data. Real-time characterizations may thus be possible. *This work was funded by DTRA under contract HDTRA1-09-C-0034. Nancy Nurthen is the PM.*

## Molecular Epidemiology

Monday, March 12
5:00 PM – 6:00 PM
Grand Hall

**Board 118. Genetic Diversity among 1,4,[5],12:i:- and 1,4,[5],12:-:1,2 Monophasic and in 1,4,[5],12:-:- Non-Motile Variants of *Salmonella enterica* Serotype Typhimurium**

**A. Brisabois**, M. Bugarel, S.A. Granier, S. Roussel, P. Fach;
Anses, Maisons-Alfort, France.

**Background:** Most *Salmonella enterica* subspecies *enterica* serovars possess 2 flagellar phases encoded by *fliC* (phase 1) and *fljB* (phase 2) genes respectively, but some strains may lack either the first or the second phase H or both phases. Numerous zoonotic *Salmonella* monitoring programs pointed out the increase of *Salmonella*-Typhimurium-like isolates worldwide, in particular those with the antigenic formulae 1,4,[5],12:i:-. Then, the public health risk associated to the emergence of such monophasic isolates is posed. This study aims to characterize the emerging monophasic strains lacking either the second or the first phase H-antigen or the both phases (non-motile variant) through the detection of different molecular markers of interest. **Methods**: The genetic diversity of a collection of 58 monophasic and non-motile isolates of from swine, bovine and poultry sources was investigated using a novel multiplex real-time PCR able to simultaneously target ten different classes of genes in regards to antimicrobial resistance phenotypes and pulsed field gel electrophoresis (PFGE) types. **Results:** The majority of the S. 1,4,[5],12:i:- strains were genotypically confirmed to be variant of serovar Typhimurium. Five *fljB* positive S. 1,4,[5],12:i:- variants could be considered as inconsistent variants suggesting that the phase 2 expression is blocked by mutation or deletion. Based on the combination of detection of the following markers, *Salmonella* Pathogenic islands, virulence plasmid antimicrobial resistance markers ($bla_{TEM}$ and *sul1*), *Salmonella* Genomic island (SGI-1 left junction), integrase of class 1 integron and DT104 16S-23S spacer region, thirteen different genotypes were identified among the 58 isolates. All isolates showed virulence potential determined by the presence of four SPI markers exhibited in a same genotype mainly encountered for S. 1,4,[5],12:-:1,2. In contrast, a high-number of markers was detected for S. 1,4,[5],12:i:- gathering all the investigated markers except SGI-1. **Conclusions:** This study on the French collection could suggest presence of the previously described Spanish clone in France as well as, to a lesser extent, some inconsistent variants exhibiting a resistance-type to 4 antimicrobials described in numerous other countries.

**Board 119. Molecular Characterization of Enterotoxigenic *Escherichia coli* Isolates from Diarrhea Patients in Shenzhen City, China**

**Q. Hu**[1,1], Y. Li[1], D. Lv[2], Y. Lin[1], Y. Qiu[1], X. Shi[1], J. Mou[1], H. Ma[1], J. Cheng[1];
[1]Shenzhen Centre for Disease Control and Prevention, Shenzhen, China, [2]School of Life Science, Shenzhen University, Shenzhen, China.

**Background:** To determine the epidemiological and etiological relationship of enterotoxigenic *Escherichia coli* (ETEC) clinical isolates in diarrhea patients in Shenzhen city, Guangdong province, China. **Methods:** Stool samples were collected from 15884 diarrheal patients of all ages in 2007-2010. *E. coli*-like colonies were identified by detecting the e*stt*A2-4 (STh), *est*A1(STp) or *elt*B(LT) gene using PCR. ETEC isolates were serotyped and antimicrobial resistance to 23 antibiotics was determined using the Kirby-Bauer disc diffusion method. PCR was used to determine the presence of the major structural subunit genes of 18 colonization factor antigens. Finally, pulsed-field gel electrophoresis (PFGE) using the restriction enzymes Xbaland AvrII was performed. **Results:** Forty-eight ETEC isolates (16.7%) of 287 *E. coli*-like colonies were identified by enterotoxin PCR. Among 48 isolates, 24 were STh positive, 18 were

STp positive and 9 were LT positive (three were positive for both STh and LT encoding genes). The age of infected individuals ranged from 1 to 68 years old, however 88% of patients were older than 10 years old; only 6 patients were children. Fifty-six percent of patients were female. Eleven patients had fever and 12 vomited. The most common O-antigen serotype was O159 (41.6%), followed by O27 and O6 (16.7%), O169 (12.5%), O148 (8.3%) and O25 (4.2%). Only 27 isolates had identifiable H-antigen results and the three most identified serotypes were O27:H7, O159:H34 and O159:H7. CFAs were detected in 30 ETEC (62.5%) and the most common CFA based on PCR was CS6 (n=26), distantly followed by CS21 (n=5), CS13 (n=4), CS3 (n=3), CS1 (n=2), and CS2 (n=1); and no CFA genes were detected in 18 strains. Seven isolates had multiple CFAs (CS6 and CS13, n=4; CS1, CS3 and CS21, n=1; CS2, CS3 and CS21, n=1; and CS6 and CS21 (n=1). Three clusters of indistinguishable isolates were identified. The isolates were sensitive to the most antibiotics. **Conclusions:** ETEC isolated from diarrheal cases in Shenzhen appear to have unique epidemiologic features as compared to studies in developing countries. The potential outbreaks could be monitored. ETEC potentially represents a significant but treatable cause of diarrhea in Shenzhen and surveillance for ETEC outbreaks along with prevention and control strategies could mitigate the risk of this disease.

**Board 120. Etiologic Role of Respiratory and Enteric Pathogens in Cases of Severe Acute Respiratory Viral Infections of Children during the First 4 Years of Life**

**K.T. Kasymbekova**[1,2], Z. Nurmatov[1], G. Muratbaeva[2] , M. Moldokmatova[1],E. Nogoibaeva[1],D. Otorbaeva[1];
[1]Department of State Sanitary Epidemiological Surveillance Ministry of Health, Bishkek, Kyrgyzstan,
[2]Centers for Disease Control and Prevention/Central Asia Region, Almaty, Kazakhstan.

**Background:** According to a study conducted in the sentinel surveillance in Kyrgyzstan, severe acute respiratory infections (SARI) are prevalent in children during their first 4 years of life. These children were diagnosed with respiratory onset and clinic diarrheal syndrome. **Methods:** In the season 2010-2011 surveys have been conducted by real-time PCR for respiratory viruses and intestinal infections of 100 children younger than 4 with SARI. **Results**: In the etiological structure of respiratory viruses influenza A (H3) and B made 5% and 3% respectively. Specific weight of parainfluenza viruses made 30%, less likely to identify rhinoviruses, respiratory syncytial viruses, metapnevmoviruses, amounting to 20%, 17%, and 14% respectively. In the etiological structure of identified enteric pathogens prevailed viruses (noraviruses, rotaviruses, adenoviruses, 41%, 15.4% and 7% respectively). Astroviruses were detected only in 2.6%. The role of bacterial pathogens was significantly lower. Among the bacteria most often detected are *Campylobacter* and *Shigella*, much less detected are *Salmonella*: 17.9%, 12.8%, and 2.6% respectively. Non-influenza respiratory viruses and enteric pathogens were detected more frequently in children under 2. It is determined that 8% of the children had mixed infection and acute intestinal infections of acute intestinal infection and acute respiratory infection , including 3% of cases it was a combination of parainfluenza with norovirus or rotavirus infection. Nine percent of children had mixed infections of different respiratory viral infections, including 7% of cases identified 2 viral agents, in 2% - 3 respiratory viruses. **Conclusions:** 1) Respiratory viruses and enteric pathogens were detected more frequently in children with TORI under 2 years old; 2)i n the etiological structure of respiratory viruses predominated parainfluenza viruses, rhinoviruses , respiratory syncytial viruses, and metapneumovirus; 4) in the etiological structure of enteric pathogens, viruses prevailed; and 4) 8% of the children had mixed respiratory infection and acute intestinal infections.

**Board 121. Etiological Surveillance for Severe Fever with Thrombocytopenia Syndrome in Hubei, China, 2010-2011**

**J. Xu**, J. Zhan, F. Zhan, Y. Jiang, X. Huo, X. Guan, X. Li, J. Cheng;
Hubei Province Center for Disease Control and Prevention, Wuhan, China.

**Background:** Severe fever with thrombocytopenia syndrome (SFTS) is an emerging infectious disease discovered in 2010, which is caused by a novel bunyavirus, SFTS virus (SFTSV). SFTS occurs in north-eastern and central China, such as Hubei, Henan, Shandong, etc. SFTS causes high fatality rate of 12% and as high as 30% in some areas. Since 2010, there have been 420 reported cases of SFTS in Hubei province in central China, with a case fatality rate of 12%. **Methods:** Acute-phase blood samples were collected from patients with the case definition of SFTS in Hubei province. All acute sera were used to detect viral RNA of SFTSV by real-time RT-PCR, and then RT-PCR positive samples were used to isolate viruses by inoculation of cell culture. The cultured isolates were confirmed by indirect immunofluorescence and characterized nucleic acid sequencing. Further phylogenetic analysis was conducted by including strains with known geographical origin from other provinces in China. **Results:** From 2010 to 2011, a total of 355 blood samples were collected and tested by real-time RT-PCR, 135 samples in 2010 and 220 samples in 2011, respectively. Out of 135 samples in 2010, 90 (66.7%) were positive for SFTSV; while out of 220 samples in 2011, 85 (38.6%) were positive for SFTSV. The result indicates that is high positive rate by real-time RT-PCR test. Compared to 66.7% in 2010, the real-time RT-PCR positive rate of 38.6% in 2011 decreased dramatically. The results were consistent with those from other provinces in China. In addition, from these positive samples, we isolated 8 strains of the bunyavirus and sequenced their genomes. Phylogenetic analysis conducted by the neighbor-joining method (MEGA package, version 4) by 1,000 bootstrap resamplings indicated that they were genetically similar to previously isolated strains by other provinces including Henan, Shandong, Anhui, Jiangsu, and Liaoning. **Conclusions:** High viral RNA positive rate indicates that real-time RT-PCR test is a valuable tool in the early diagnosis of SFTS in annual epidemic season. Different geographical distributions of SFTSV were genetically similar to each other. Importantly, dramatic decrease in positive rate of real-time RT-PCR in 2011 indicates whether SFTS is also caused by unknown pathogens except SFTSV.

**Board 122. Long Term Endurance of Dengue Virus Serotype 2 and Transient Emergence of DENV-3 in Puerto Rico: 1986-2010**

**G.A. Santiago**, J.L. Munoz-Jordan;
Centers for Disease Control and Prevention, San Juan, PR, USA.

**Background:** Dengue virus (DENV) exists as 4 serotypes. Migration has contributed to the divergence of distinct lineages within each serotype. Using data from 25 years of dengue surveillance in Puerto Rico, we found that the genetically distinct Southeast Asian DENV-2 strain continuously circulated on the island; whereas the Indian subcontinent DENV-3 strain emerged in 1998 and disappeared in 2007. **Results:** We sequenced the complete genomes of 160 DENV-2 and 92 DENV-3 low passage isolates obtained from Puerto Rican patients with laboratory confirmed dengue and characterized the genetic diversity and phylogeography of these two serotypes for 21 and 10 years, respectively. Genetic lineages were analyzed with respect to temporal, geographical and serotype-specific incidence data. While DENV-2 evolved as one lineage with a unique clade replacement, DENV-3 emerged and expanded through five parallel lineages. The expansion of DENV-3 was concomitant with a transient 4 year decline of DENV-2. Bayesian skyline analysis showed that the fast dissemination of DENV-3 on the island was characterized by a large genetic population size as compared by a low population size of DENV-2 during that time period. In addition, our phylogeography analysis combined with serotype-specific incidence data showed that the presence of a DENV serotype in a given location and time is usually correlated with the absence of the other serotype. **Conclusions:** Co-transmission of DENV-2 and DENV-3 lineages occurs through complex dissemination patterns and serotypes seem to mutually exclude one another geographically and temporally. We found evidence of sustained microevolution within lineages, transient refuge and fast migration across the island. One limitation of our study is the unavailability of viruses involved in silent transmission. Research to further define the biological and epidemiological

determinants of DENV emergence and decline may help us prevent the spread and re-emergence of dengue.

**Board 123. Spatial Pattern of Pathogens Associated with Fever of Unknown Origin in Kenya**

**J.N. Waitumbi**;
Walter Reed Project/KEMRI, Kisumu, Kenya.

**Background:** Fevers of unknown origin (FUO) are a common presentation in patients visiting healthcare facilities in Kenya. The causes of these illnesses are vast and have determinants such as ecology, rainfall patterns, human-animal contacts, and population movements among many others. This study reports on pathogens identified by molecular methods in patients presenting with FUO at various clinics in Kenya. **Methods:** 1716 blood samples were collected between March 2008 and July 2011 under an "acute febrile illnesses" human use protocol (KEMRI SSC 1282; WRAIR 1402) from participating clinics located in the lowlands, highlands and arid areas. Entry criterion to the study was documented fever (temperature > 38 °C) without a readily diagnosable cause. Nucleic acids were extracted from blood and used for RT-qPCR diagnosis of *Plasmodia*, *Salmonella*, *Rickettsia*, *Brucella* and dengue fever virus. **Results:** Of the 1,716 specimens analyzed, 461 (25%) had malaria, 95 (5.9%) *Salmonella*, 23 (1.7%) *Rickettsia*, 6 (0.34%) *Brucella* and 10 (0.6%) dengue fever virus, either alone or in combination. Interestingly, malaria accounted for the greater percent of etiologies associated with FUO in all areas but was highest in the arid areas (52.3%) compared to 26.3% in the lowlands or 12.7% in the highlands. In arid regions, 60% of the participants had diagnosable cause of FUO (malaria 52.3%, *Salmonella* 2.8%, *Rickettsia* 2.8%, *Brucella* 1.5% and dengue 0.9%) compared to 35.2% from the lowlands (26.3% malaria, 7.7% *Salmonella*, 1.2% *Rickettsia*) or 19.5% from the highland (12.7% malaria, 5.7% *Salmonella*, 0.9% *Rickettsia* and 0.2% dengue). **Conclusions:** Molecular data from this study indicates that malaria and *Salmonella* are a significant burden of FUO in Kenya. Unexpectedly, malaria was identified as a greater cause of FOU in the arid regions than in the traditional malaria endemic lowlands of Kenya. The unusually high proportion of undiagnosed FUO are worrying and calls for newer methods with greater capability for multiplexing such as MassTag PCR, Lumixex, and TAC.

## Novel Surveillance Systems

Monday, March 12
5:00 PM – 6:00 PM
Grand Hall

**Board 124. The Ratio of Emergency Department Visits for ILI and Self-reported Illness on a Survey to Seroprevalence of 2009 H1N1 Infection, Florida, 2009**

**R.S. Hopkins**, K. Goodin, M. Weise, A. Kite-Powell, J.J. Hamilton;
Florida Department of Health, Tallahassee, FL, USA.

**Background:** In an evolving influenza epidemic, existing real-time surveillance systems do not provide needed information about cumulative incidence of infection in the population. A seroprevalence survey carried out in Tampa Bay, Florida in late 2009 yielded such an estimate for the 2009 H1N1 influenza pandemic. We calculated the ratio of influenza-like illnesses detected in 2 existing surveillance systems to actual infections. **Methods:** Total emergency department (ED) visits for influenza-like illness (ILI) during the epidemic period were estimated using the ESSENCE-FL syndromic surveillance system, by age group, for Florida and for Tampa Bay. Cumulative statewide reports of ILI using 3 different definitions were derived from the Behavioral Risk Factor Surveillance System (BRFSS), for interviews conducted Oct. 1—Dec. 27, 2009, using the BRFSS weights to make statewide estimates for persons aged 18 and older.

**Results:** For all ages combined, for the whole state there were an estimated 27.5 infections for every ILI ED visit. Age-specific ratios rose with age, from 8.5 to 1 for children aged under 5 years to 66 to 1 for persons aged 65 and over. Corresponding ratios for just Tampa Bay were 37 to 1, 11.3 to 1, and 98 to 1. For persons aged 18 and over, there were 3.0 infections for every 1 person reporting ILI (fever and cough and/or sore throat) on BRFSS interview. Ratios ranged from 1.4 to 1 for persons aged 50 to 64 years, to 8.4 to 1 for persons 65 and over. For more stringent BRFSS ILI definitions, ratios are much higher. **Conclusions:** For jurisdictions using syndromic surveillance systems or ongoing BRFSS-like interviews, these ratios provide a way to estimate weekly and cumulative incidence, which can be used together with estimates of the reproductive number ($R_0$) for planning and forecasting. Syndromic surveillance data allow more detailed age and geographic breakdowns and a full age range, and are more timely than BRFSS data.

**Board 125. Enhanced Passive Surveillance System for Animal Health – Texas Pilot Project**

**J. Akkina**, D. Cox, C. Johnson, M. Remmenga, G. Ross, A. Scott, W. Weber;
Veterinary Services, Animal and Plant Health Inspection Service, United States Department of Agriculture, Fort Collins, CO, USA.

**Background:** The Veterinary Services (VS) strategic vision includes increased focus on disease detection, preparedness, response, and expansion of the veterinary health mission to include public health and wildlife. Traditional animal surveillance programs have focused on disease- and agent-specific detection with confirmed laboratory diagnosis, limiting their disease scope and usefulness to detect emerging diseases. The goal of the Enhanced Passive Surveillance (EPS) project is to provide non-disease-specific surveillance (i.e. syndromic) and early detection of endemic, zoonotic, and emerging animal diseases through the integration of multiple surveillance streams in near real time. **Methods:** A pilot project focusing on cattle primarily in Texas will explore the usefulness of the EPS system. This project is a collaborative effort including: VS, Texas Animal Health Commission, Texas Veterinary Medical Diagnostic Laboratory, National Center for Foreign Animal and Zoonotic Disease Defense, and New Mexico Agriculture Livestock Incident Response Team. Surveillance streams to be integrated include data from private practitioners, livestock markets, livestock harvesting facilities (LHF), veterinary diagnostic laboratories, and wildlife information sources. Currently the LHF data stream is operational and other streams are in development. Counts of animals condemned by various categories (e.g. pneumonia) obtained from the Food Safety Inspection Service are analyzed weekly using a CuSum statistical method to identify counts significantly above expected levels. Similar methods will be used to analyze the other data streams. **Results:** Over a 12-month period, analysis of the LHF stream indicated two weeks with counts of beef cow condemnations for pneumonia in Texas slaughter plants processing beef cows significantly above the expected level. **Conclusions:** Timely surveillance focused on early detection of disease and changes in animal health status will allow animal health officials, public health officials, and private practitioners to respond more quickly and to make decisions regarding disease control measures based on more information. The integration and analysis of multiple surveillance streams simultaneously will increase the potential to identify significant animal health events.

## Tropical Infections and Parasitic Diseases

Monday, March 12
5:00 PM – 6:00 PM
Grand Hall

**Board 126. Investigation of Human Visceral Leishmaniasis Outbreak in an Unusual Ecosystem: Saltwater Marsh**

**P.B. Magalhes**, E.D. Moreira Jr;
Oswaldo Cruz Foundation, Salvador, Brazil.

**Background:** Human visceral leishmaniasis (HVL) is recognized as an important reemerging infectious disease in the world. Recently, the occurrence of HVL in large urban centers outskirts has caused concern to public health. The mangrove is a coastal ecosystem of transition between marine and terrestrial environments, subject to tidal regime with plants and animals adapted to high salinity and periodic flooding. Describe a HVL outbreak in a previously unaffected area and the epidemiological characteristics of cases notified. **Methods:** The outbreak took place in the municipality of Salinas, in 2004, a 15 thousand city located in northeastern Brazil. It has a tropical climate, with an annual average temperature of 25.4°C and rainfall of 1920 mm yearly. The natural predominant vegetation consists of some small deciduous trees, shrubs, cordgrass, and pickleweed, the latter two by far the best adapted plants for regular tidal inundation. **Results:** Overall, 26 cases were reported. Fifty-three percent were male and 30.8% adults (≥ 20 years). All patients had been diagnosed by either parasitological demonstration of the *Leishmania* or by clinical presentation (fever, hepatoespenomegaly and pancytopenia) plus response to antimonial therapy. The lethality was 3.85% (1/26). In 34.6% of households with cases, all other occupants of the house were with evidence of infection prior to LVH, despite the failure to develop symptoms. About 60% of households had dogs and half were captured sandflies (*Lutzomyia longipalpis*) in a single attempt. **Conclusions:** To our knowledge, this report documents for the first time the occurrence of HVL in a highly unexpected environment. It also highlights the ability of the sandfly (*Lutzomia longipalpis*) to adapt to a saltwater marsh ecosystem. The implications of these findings should be further studied.

**Board 127. Local Dengue in Florida**

**D. Stanek**[1], L. Stark[2], L. Anil[1], C. Blackmore[1];
[1]Florida Department of Health, Tallahassee, FL, USA, [2]Florida Department of Health Bureau of Laboratories, Tampa, FL, USA.

**Background:** From July 2009 through September 30, 2011 there were 7 known dengue virus introductions into Florida. **Methods:** Following are important epidemiologic, ecologic, and laboratory findings from these outbreaks. **Findings:** Introductions occurred in 5 of Florida's 67 counties: Broward (1), Hillsborough (1), Martin (1), Miami-Dade (3), and Monroe (1). One of the 7 outbreaks persisted for 1.5 years (Monroe) with 93 cases identified. In six instances only a single locally transmitted case was found. In two instances the source case was known (Martin and Hillsborough) and involved secondary infection following international travel by another household member. While 4 introductions occurred in counties with the highest number of imported dengue cases in the state (Miami-Dade and Broward), 2 were in counties with a relatively low number of imported cases detected (Martin and Monroe) including the outbreak with longest sustained transmission. In 2 instances the outbreak area included popular domestic and international tourist destinations (Monroe and Miami). Six of the 7 outbreaks were in counties that housed international airports and/or cruise ship ports. Likely exposure site for index cases were at the case home (5), tourist staying in a bed and breakfast (1), and occupational outside work (1). Five of the exposure sites were in neighborhoods with yards and ample vegetation, 1

involved an apartment complex in an urban area, and one site was not identified. *Aedes aegypti* was the implicated vector in 6 of the events, while *Ae. albopictus* appeared to be the vector in Martin County. During the first outbreak, laboratory capacity was significantly enhanced by the addition of molecular diagnostic techniques. This was particularly important when assessing suspect cases with previous dengue virus exposure, due to the transient nature of dengue IgM in many of these cases. Virus type was identified in 4 instances: DEN-1 in 2 cases, DEN-2 in 1 case and DEN-3 in 1 case. Virus strain was also available for 3 outbreaks, providing valuable epidemiologic information. Additional virus typing is pending. **Conclusions:** Dengue introduction ecology in Florida is variable. However, most introductions occurred in suburban settings, in counties with large numbers of imported cases, and involved *Ae. aegypti*.

**Board 128. *Trichuris trichiura*: A Problematic Parasite for Deworming Programs?**

**J. Gabrie**[1], A. Sanchez[1], M.M. Rueda[2], M. Canales[2], T.W. Gyorkos[3];
[1]Department of Community Health Sciences, Brock University, St. Catharines, ON, Canada, [2]School of Microbiology. National Autonomous University of Honduras, Tegucigalpa, M.D.C., Honduras, [3]Division of Clinical Epidemiology, McGill University Health Centre and Department of Epidemiology, Biostatistics and Occupational Health, McGill University, Montreal, QC, Canada.

**Background:** Mass school-based preventive chemotherapy is recommended by PAHO/WHO as the main intervention strategy for reducing the occurrence of soil-transmitted helminth infections (STH) in children living in highly endemic areas. In Honduras, deworming campaigns have been in place for several years. A cross-sectional survey aiming to study gender-specific determinants of STH in school-age children provided data from which we sought to obtain an indication of deworming effectiveness by comparing the occurrence of STH in children who had previously been dewormed with children who had not been previously dewormed. **Methods:** The survey was conducted between Feb. and Mar. 2011 in 8 rural primary schools in Honduras with the objective of enrolling 377 children. Stool specimens were examined using the Kato-Katz technique and epidemiological data, using a standardized questionnaire. Prevalence odds ratios (POR) were calculated for each STH infection and compared between groups. **Results:** 320 (84.9%) children (aged 7-14; mean: 10) from 8 schools completed the study, among which 275 (85.9%) children from 6 schools had previously received at least one deworming treatment. The overall STH prevalence was 67%, with 62%, 27% and 6.5% for *Trichuris, Ascaris* and hookworm infections, respectively. Bivariate analyses showed that previously dewormed children were 24% more likely to be *Trichuris*-positive than children not previously dewormed (POR= 1.24, 95%CL: 0.62,2.46). Further multivariate analyses will adjust for possible confounding. **Conclusions:** Despite previous deworming treatment, the prevalence of STH remained high in the study population, and *Trichuris* infections, in particular, seemed to be problematic. In addition to the monitoring of coverage, deworming programs would benefit from also monitoring parasite-specific infections. In this way, infections not responding adequately to treatment can be identified and alternative interventions or frequency of interventions considered.

**Board 129. Malaria in Greece, 2011**

**K. Danis**[1], A. Baka[1], I. Terzaki[1], G. Dougas[1], M. Detsis[1], E. Papanikolaou[1], S. Gewehr[2], C. Kefaloudi[1], A. Balaska[1], A. Economopoulou[1], S. Tsiodras[1], N. Vakalis[3], S. Bonovas[1], J. Kremastinou[1];
[1]Hellenic Centre Disease Control and Prevention, Athens, Greece, [2]Ecodevelopment, SA, Thessaloniki, Greece, [3]National School Public Health, Athens, Greece.

**Background:** Greece was declared malaria free by the World Health Organisation in 1974. In 2009 a cluster of 8 cases of *P. vivax* was reported from Evrotas, Lakonia in southern Greece. In late May 2011, a new case occurred in the same area. An investigation was initiated in order to identify new cases, to determine the potential source of infection and determine the affected area(s). **Methods:** Malaria is a

notifiable disease in Greece. Malaria cases were interviewed using a questionnaire, gathering information on potential modes of transmission and clinical and detailed travel history. Diagnosis was done by microscopy and all positive samples were confirmed by PCR. Entomological surveys were conducted in identified affected areas. **Results:** By 23/09/2011, 35 malaria cases of *P. vivax* infection were identified in Greece; 19 in Greek citizens. Sixteen cases were in migrant seasonal agricultural workers; 13 from malaria endemic countries. Disease onset ranged from 23/05/2011 to 14/09/2011 with a peak (13 cases) during the first two weeks of September. Twenty-nine cases resided in the small ($25km^2$) agricultural area of the Evrotas delta, Lakonia. The remaining six cases were distributed in agricultural areas in four other districts. A high number of migrant workers from malaria endemic countries reside in all affected areas. The most likely implicated mosquito vector was *Anopheles sacharovi*. **Conclusions:** Preliminary findings indicate that local transmission of *P. vivax* may have occurred in 2011 in mainly one de-limited geographical area in Greece. Control measures included increasing awareness among physicians, active case-finding and treatment and intensified vector control. Research on the implicated mosquito vector is needed to better understand the transmission dynamics in Greece and allow targeted control measures.

**Board 130. Dengue Outbreak among African Union Peacekeepers—Mogadishu, Somalia, 2011**

**D.F. Arguello**[1], H. Kyobe Bosaa[2], J.M. Montgomery[3], B. Fields[4], L. Waiboci[4], S. Gikundi[4], V. Ombala[4], R. Downing[5], J. Lutwama[6], J.T. Kayiwa[6], S. Balinandi[7], J. Borchert[8], T. Shoemaker[7], J. Muñoz-Jordan[1], E. Hunsperger[1], E. Hunsperger[1], R. Lanciotti[9], I. Kimuli Kasozi[2], R. Nasci[9], H. Margolis[1];
[1]Dengue Branch, Centers for Disease Control and Prevention, San Juan, PR, USA, [2]Ugandan Peoples Defense Force, Kampala, Uganda, [3]International Emerging Infectious Disease Program, Centers for Disease Control and Prevention, Nairobi, Kenya, [4]Global Disease Detection Program, Centers for Disease Control and Prevention, Nairobi, Kenya, [5]Global AIDS Program, Centers for Disease Control and Prevention, Entebbe, Uganda, [6]Uganda Virus Research Institute, Entebbe, Uganda, [7]Viral Special Pathogens Branch, Centers for Disease Control and Prevention, Entebbe, Uganda, [8]Bacterial Diseases Branch, Centers for Disease Control and Prevention, Entebbe, Uganda, [9] Arbovirus Diseases Branch, Centers for Disease Control and Prevention, Fort Collins, CO, USA.

**Background:** In May 2011, an outbreak of hemorrhagic fever cases, including three deaths, was observed among African Union Mission in Somalia (AMISOM) soldiers in Mogadishu, Somalia. **Methods:** To identify the etiology and describe the scope of this outbreak, surveillance and testing was established at the three AMISOM hospitals in Mogadishu. A case was defined as a febrile patient with a negative malaria blood smear and ≥ 2 of the following: anorexia, headache, vomiting, hemorrhage, or arthralgias. Serum specimens were tested for Ebola virus (EBOV), Marburg virus (MARV), yellow fever virus (YFV) and dengue virus (DENV) using antigen-capture enzyme linked immunosorbent assays (ELISA), real-time reverse transcription polymerase chain-reaction (rRT-PCR) and IgM capture (MAC) ELISA. Specimens were also tested for zika virus (ZIKA) and chikungunya virus (CHIKV) using MAC ELISA and rRT-PCR. **Results:** Between May and August 2011, 161 cases were reported and 134 were tested. CHIKV, EBOV, MARV, YFV, and ZIKV testing were all negative. Dengue was confirmed in 107 (80%) cases, corresponding to a 1% attack rate among AMISOM peacekeepers in Mogadishu and 4% among those hospitalized with fever. Dengue cases came from 13 outposts in Mogadishu. Of 82 cases with a positive rRT-PCR, 37%, 26%, and 7% had DENV-1, -3, or -2, respectively; 31% were tested with an assay that did not allow for virus typing. Of 105 dengue cases with complete data, 103 (98%) were male; 76 (72%) were Ugandan and 29 (28%) were Burundian; 13 (12%) had at least one hemorrhagic manifestation; 54 (50%) were hospitalized (median duration = 4 days [range1--8]); none died. **Conclusions:** This dengue outbreak in African peacekeeping forces highlights that DENV transmission occurs in African countries and populations are at risk of disease, including severe dengue. The outbreak also illustrates the importance of dengue diagnostic testing in the evaluation of patients with acute febrile illness. Further

studies are needed to determine the epidemiology of dengue in Mogadishu and Somalia, and to determine the risk DENV transmission poses to civilians and international relief agency personnel.

## Strengthening Public Health Systems

Monday, March 12
5:00 PM – 6:00 PM
Grand Hall

**Board 131. Usefulness of Syndromic Surveillance for Decision-Making during the 2009 H1N1 Pandemic in Ontario, Canada**

**R. Savage**[1], A. Chu[1,2], N. Crowcroft[1,2], L. Rosella[1,2], D. Willison[1,3], I. Johnson[1,2];
[1]Public Health Ontario, Toronto, ON, Canada, [2]University of Toronto, Toronto, ON, Canada, [3]McMaster University, Hamilton, ON, Canada.

**Background:** Despite the growing popularity of syndromic surveillance, little is known about how these systems are used to support public health actions. This study seeks to describe the use and value of syndromic surveillance systems in Ontario, Canada both routinely and during the 2009 H1N1 pandemic within the context of other existing surveillance systems. **Methods:** All of Ontario's 36 public health units, the provincial ministry of health, and federal public health agency completed a web survey in 2010 to identify surveillance systems used routinely and during the pandemic, and to rate their accuracy, timeliness and reliability. Follow-up semi-structured interviews were conducted with syndromic surveillance users and non-users to evaluate the role of syndromic data in supporting decision-making during the pandemic. **Results:** Among the 38 organizations, 22 (58%) use syndromic surveillance systems. Traditional surveillance using laboratory and reportable disease data, and syndromic surveillance using school absenteeism data were the most frequently used data sources routinely and during the pandemic. Laboratory data were considered more reliable, timely and accurate than syndromic data (92%, 51%, 89% of organizations rated it as 'very acceptable'). Hospital/clinic screening data were rated the most reliable and timely syndromic data source (50% and 43%) and emergency department visit data the most accurate (48%). Despite this, syndromic data were reported to be useful for supporting influenza assessment centre operations and communications with the public. Interviews revealed inconsistency in methods used to generate alerts of aberrant events and frequent uncertainty in how to respond to alerts, with only 13/25 (52%) of systems having response protocols (12% written). **Conclusions:** Although traditional surveillance systems were considered more reliable, timely, and accurate than syndromic data, syndromic surveillance did support some public health actions taken during the pandemic. Further development and validation of standardized alerting algorithms and response protocols could enhance the effectiveness of syndromic data for public health surveillance.

**Board 132. Etiology of Diarrheal Disease among Hospitalized Patients Enrolled in Integrated Infectious Disease Surveillance in Northern and Greater Accra Regions of Ghana**

**G. Armah**[1], R. Akuffo[2], M. Clemens[2], K. Kronmann[2], A. Jones[3], P. Agbenohevi[4], K. Sagoe[5], N. Puplampu[2], T. Nzussouo[6], E. Dueger[7];
[1]Noguchi Memorial Institute for Medical Research, Accra, Ghana, [2]U.S Naval Medical Research Unit No.3, Ghana Detachment, Accra, Ghana, [3]U.S Naval Medical Research Unit No.3, Global Disease Detection and Response Program, Cairo, Egypt, [4]37 Military Hospital, Ministry of Defence, Accra, Ghana, [5]Tamale Teaching Hospital, Ministry of Health, Tamale, Ghana, [6]U.S Centers for Disease Control and Prevention, Atlanta, GA/U.S Naval Medical Research Unit No.3, Ghana Detachment, Accra, Ghana, [7]U.S Centers for Disease Control and Prevention, Atlanta, GA/U.S Naval Medical Research Unit, Cairo, Egypt.

**Background:** Despite advances in health technology and increased use of oral rehydration therapy (ORT), diarrheal diseases continue to remain among the five highest causes of mortality among children under five years of age. **Methods:** Surveillance for hospitalized acute diarrheal infections (ADI) was initiated in November 2010 as part of ongoing integrated infectious disease surveillance for acute respiratory infections (ARI) and acute febrile illnesses (AFI) in a referral hospital in the greater Accra region, and a teaching hospital in the northern region of Ghana. Patients who met a standardized case definition provided demographic and epidemiologic data. Stool samples were cultured and subjected to a panel of viral and parasite enzyme immunoassays (EIA). **Results:** In the first 10 months of surveillance, 268 patients were enrolled with ADI. Of these 29 (10.4%) also met case definition criteria for ARI and 94 (32.2%) for AFI. 109 (41%) ADI patients were under 5 years, and 146 (54%) were females. Outcome data available for 178 (66%) ADI patients showed that 9 patients died, 167 were discharged and 1 was transferred to another hospital. Of 59 samples tested to date by viral EIA, 17 (28.8%) were positive for rotaviruses, 4 (6.8%), for adenoviruses, 2 (3.4%), for noroviruses and 3(5.1%) for astroviruses, while 3(5.1%) had mixed infections. Fifty-seven samples were tested for parasitic infections; one (1.8%) each was positive for *Giardia* and *Cryptosporidium* respectively while another sample (1.8%) had a mixed infection. Pathogens isolated from stool cultures include one *Shigella* sp., one *Salmonella enterica*, one *Routella* sp., and one *Vibrio cholerae*. An NP/OP swab from a 10 year old male meeting both the ARI and ADI case definitions tested positive for influenza B. **Conclusions:** This preliminary data provides previously unknown information on viral distribution of ADI in Ghana. Continued integrated hospital-based surveillance that includes ADI will elucidate etiologies of hospitalized diarrheal diseases in Ghana and detect associations between diarrheal diseases and other disease syndromes.

**Board 133. Use of Standardized Case Definitions in Surveillance for Especially Dangerous Pathogens in Kazakhstan, 2009–2010**

**Y. Bumburidi**[1], A. Zholshorinov[2], V. Zeman[1], I. Ivasiv[1], C. Pitenko[1];
[1]Centers for Disease Control and Prevention (CDC)/Central Asia Region (CAR) Cooperative Biological Engagement Program (CBEP), Almaty, Kazakhstan, [2]Ministry of Health of Kazakhstan, Almaty, Kazakhstan.

**Background:** The use of standardized case definitions (SCDs) is vital to surveillance. While training in the use of SCDs is common, evaluation of the effectiveness of this training in clinical practice is not. SCDs were introduced into surveillance for Especially Dangerous Pathogens (EDPs) in Kazakstan in 2006. Each physician encountering a suspect EDP case must complete an initial emergency case notification form (ECNF) followed by a final form with ultimate diagnosis. To introduce SCD into surveillance, CDC/Central Asia Region has been training clinicians, epidemiologists, and laboratory specialists. **Methods:** Training coverage differed by oblast ("state"). In some oblasts, only clinicians were trained ("defined as lesser-trained group"; LTG), while in other oblasts- or rayon- ("county") clinicians as well as epidemiologists and/or laboratory specialists were trained (defined "highly-trained group"; HTG). Four oblasts were selected with different training coverage, two in the LTG and two in the HTG. Data on the use of SCDs in initial ECNFs and their accuracy as judged by final confirmation of the initial diagnosis on final ECNFs were collected in all districts of the four oblasts. Infectious disease registration logs (form 60 u) from 2009-2010 were used as the source of information. We calculated percentage of initial ECNFs for brucellosis and anthrax using SCDs and percentage of final correct diagnoses. We compared the results we obtained between the HTG and LTG groups, using chi-square or Fisher's exact tests for statistical testing. **Results:** A brucellosis case classification was used three times less often in the LTG (296/1037; 28%) than in the HTG (1055/1178; 89%), $p<0.01$. An unsubstantiated initial diagnosis of brucellosis in LTG (508; 49%) was 5 times higher than in the HTG (107; 9%), $p<0.01$. An anthrax case classification was used in neither of 2 (0%) registered cases in the LTG vs. 8 (61%) of 13 cases in the HTG, $p=0.2$; an initial unsubstantiated diagnosis of anthrax (2/2; 100%) in the LTG was 12 times higher than in the HTG (1/13;

8%), p=0.03. **Conclusions:** Intensive training led to improved use of SCDs and, hence, improved surveillance. In addition to training all groups important in surveillance, we recommend evaluating on-the-job performance to document the effectiveness of training.

**Board 134. Exposure Ascertainment among Sporadic Campylobacteriosis Cases—Assessing the Utility of a Data Collection Tool, Foodborne Diseases Active Surveillance Network**

**K.C. Swanson**[1,2], D.J. Cole[2], K.E. Fullerton[2], O. Henao[2], P. Clogher[3], S.J. Chai[2];
[1]Atlanta Research and Education Foundation, Atlanta, GA, USA, [2]Centers for Disease Control and Prevention, Atlanta, GA, USA, [3]Connecticut Emerging Infections Program, New Haven, CT, USA.

**Background:** Determining the proportion of foodborne infections due to specific foods and settings is essential for food source attribution, but even if exposure data for sporadic cases is routinely collected it is not reported to the Foodborne Diseases Active Surveillance Network (FoodNet). FoodNet plans to routinely collect this information. The proposed data collection tool classifies food exposures into commodities, and nonfood exposures into environmental, waterborne, or person-to-person transmission pathways. We report the results of piloting the proposed tool using existing FoodNet data. **Methods:** We mapped exposures from the most recent *Campylobacter* case-control study and 1998-99 population-based survey using the proposed data tool. We compared mapped exposures of 1316 cases in the case-control study with 1316 controls from the same study (comparison 1) and 12,755 controls from the survey (comparison 2). We used bivariable logistic regression to examine associations and compared these results with the published multivariable results. We used Cohen's kappa coefficient to assess agreement between the results of comparisons 1 and 2. **Results:** Animal contact, untreated water, seafood, and fruits-nuts were significantly associated with infection in both comparisons and the original multivariable analysis. Poultry, undercooked meat, and raw dairy were not significantly associated with illness in comparison 1 or 2. In the published multivariable results, setting determined whether poultry consumption was risky versus protective; and undercooked meat and raw dairy both included risky and protective food items. Agreement in significance for exposures between comparisons 1 and 2 was moderate (K = 0.57). All significant exposures agreed in direction. **Conclusions:** The proposed tool can identify some common risk factors for campylobacteriosis, but misses others. It does not capture setting, which may be important for food items such as poultry. Product type could be important for undercooked meat and raw dairy exposures. Modifications that address these limitations may improve the tool. Further work is needed to develop a tool that collects routine exposure information from sporadic cases and that provides data for attributing foodborne illnesses to specific commodities and settings.

## Immigrant and Refugee Health

Monday, March 12
5:00 PM – 6:00 PM
Grand Hall

**Board 135. Epidemiologic Profile of Refugees Arriving in Washington State in Fiscal Year 2010**

**L. Vonnnahme**[1], P. Houck[1], E. Yanni[2], H. Burke[2];
[1]Centers for Disease Control and Prevention, Seattle, WA, USA, [2]Centers for Disease Control and Prevention, Atlanta, GA, USA.

**Background:** Washington is among the top 10 states for refugee arrivals. In fiscal year (FY) 2010, 3003 refugees settled there, an 18% increase from FY 2009. We profiled the health of Washington State refugees to guide improvement of health services. **Methods:** We reviewed results of health screening

completed shortly after refugees settled in Washington in FY 2010, including demographics; test results for tuberculosis (TB), hepatitis B virus (HBV), parasites, and sexually transmitted infections; vaccinations received abroad and domestically; and chronic conditions (e.g., hypertension and diabetes). We calculated demographically-stratified frequencies of health conditions. **Results:** Analysis is complete for 5 of 6 regions in the state, representing 1018 of 3003 refugees. The 3 largest populations were Burmese (29%), Iraqis (20%), and Bhutanese (15%). At initial assessment, 57% of refugees were tested for HBV; 4% of those had evidence of acute or chronic infection. An additional 13% had immunity to HBV due to vaccination or prior infection. The majority (97%) of refugees were tested for TB; 19% had a positive interferon-gamma release assay or tuberculin skin test, of which only 33% had a chest radiograph (3 positive). All refugees aged $\geq$ 13 years (n=757) were said to be tested for HIV, but only 37% had results (2 positive). Syphilis testing is performed in 2 regions in refugees aged $\geq$ 13 years (n=151); 90% were tested (one positive). Stool testing for ova and parasites was completed on 79% of refugees; 25% of those were positive. Among refugees aged $\geq$ 18 years, 12% had hypertension and 3% were diagnosed with diabetes. **Conclusions:** Health screening and reporting for refugees were not uniform among local health jurisdictions (LHJs) in Washington State. Of concern was the low frequency of testing for HBV and HIV. Given the high presence of ova and parasites, universal screening in the state is recommended. Although positivity was high for initial TB testing, follow-up results were generally unavailable, as most were referred for further evaluation. Chronic conditions are currently not a priority, but should be considered as they are a long-term expense for LHJs and society. Standard guidelines for domestic screening of refugees should be developed and implemented in Washington State.

**Board 136. Measles among US-Bound Refugees from Malaysia—August–September 2011**

**E.M. Taylor**[1], S. Shetty[1], J. Berliet[1], S. Tsering[2], J. Zipprich[3], D. Lee[1], P. Kutty[1], K. Marienau[1], K. Ross[1], C. Schembri[1], G. Wallace[1], A. Barskey[1], H. Burke[1], K. Harriman[3], C. Edwards[4], D. Blythe[5], D. Shah[5], M. Said[5], J. Morillo[6], S. Smith[6], D. Hopfensperger[2], L. Ortega[1], M. Weinberg[1], N. Marano[1], W. Zhou[1];
[1]Centers for Disease Control and Prevention, Atlanta, GA, USA, [2]Wisconsin Department of Health Services, Madison, WI, USA, [3]California Department of Public Health, Richmond, CA, USA, [4]Montgomery County Department of Health and Human Services, Rockville, MD, USA, [5]Maryland Department of Health and Mental Hygiene, Rockville, MD, USA, [6]North Carolina Division of Public Health, Raleigh, NC, USA.

**Background:** Despite the availability of a safe and effective vaccine, measles remains endemic in many countries. Since the elimination of measles in the United States in 2000, imported measles cases are a major public health concern due to the potential for outbreaks among unvaccinated populations. As of August 26, 2011, 198 measles cases and 15 outbreaks had been reported in the United States, the highest number since 1996. **Methods:** On August 26, 2011, CDC was notified of a suspected measles case in an unvaccinated 15-year-old refugee from Burma (index case), who had lived in urban Kuala Lumpur, Malaysia; the diagnosis was confirmed by measles immunoglobulin M. CDC along with local and state health departments initiated contact investigations and heightened surveillance activities to identify secondary cases. CDC also implemented control measures to prevent further measles transmission and importation. **Results:** Thirty-one refugees who traveled from Malaysia on the same flight with the index case arrived in 7 states (MD, NC, NH, OK, TX, WA, and WI). Six secondary cases were identified: all in unvaccinated persons, 3 in refugees, (median age 1.8 years; range 7 months to 25 years). On September 7, CDC was notified of another laboratory-confirmed case in an unvaccinated 23-month-old refugee from Burma, who also traveled from Malaysia on August 24, but on a different flight than the index case. One secondary measles case in an unvaccinated 11-month-old non-refugee child was linked to this case. Refugee travel from Malaysia to the United States was temporarily suspended. Additional measures included vaccinating US-bound refugees in Malaysia without evidence of measles immunity with measles, mumps, and rubella vaccine; postponing their travel for 21 days after vaccination; administering post-arrival health examinations in the US; creating awareness among the

medical community; isolating suspected measles patients; obtaining specimens for measles confirmation and virus genotyping; and promptly reporting cases to local health departments. **Conclusions:** This outbreak highlights the importance of vaccinating US-bound refugees and US residents according to the Advisory Committee for Immunization Practice recommendations, as well as the need to better understand the health and vaccination status of urban refugees.

**Board 137. Cholera Outbreak in Mae La Refugee Camp, Thailand, 2010**

**C. Deglise**;
Premiere Urgence-Aide Medicale Internationale, Bangkok, Thailand.

**Background:** Refugee camps are vulnerable to cholera because of resources constraints, limited sanitation infrastructure, and overcrowding. Established along the Thai-Burmese Border since more than 20 years, Mae La refugee camp provides temporary shelter for approximately 45,000 mostly Karen refugees in a four-square-km area. Cholera is endemic in Mae La camp and cases have been detected in four of the last seven years. Additional cases among Burmese migrant living in the area have also been detected. We describe the latest outbreak occurring in 2010. **Methods:** A suspected cholera case was defined as any patient presenting with acute watery diarrhea and severe dehydration. Confirmatory testing (isolation of *Vibrio cholerae* O1 or O139) was performed for all suspected cases. Suspected cases were identified through self-report and active case finding. Contact tracing and outbreak response were conducted at the household level for all cases. **Results:** The outbreak of cholera in Mae La camp included 578 suspected cases and lasted from the 27th of May to the 14th of December 2010. The peak was reached within the first 6 weeks and sporadic cases occurred in the last 3 months. Among all 578 suspected cases, 363 (63%) were laboratory-positive for *V. cholerae* O1 Ogawa (including 6 cases in non-residents), 1 was laboratory-positive for *V. cholerae* O1 Inaba, and 213 (37%) were laboratory-negative. Among camp residents, the overall incidence of confirmed symptomatic cholera was 8.2 cases per 1000 refugees; incidence was highest in persons aged 5-15 years (range: 3 months to 80 years) and in males. Less than 40% of the confirmed cases were classified as severe and no case fatality was reported. In addition to the confirmed cases, 150 asymptomatic carriers were identified during active case finding. **Conclusions:** The risk of cholera outbreaks becoming recurrent is high in a protracted refugee setting, with a highly vulnerable and mobile population situated in an endemic border area. A coordinated approach between partners in and out of the camp and strong support from the community is needed to ensure preparedness, surveillance, and early response. Although improved water, sanitation, and hygiene remain the definitive solution, oral cholera vaccination could be considered as a complementary interim strategy in such situations.

## Foodborne and Waterborne Infections

Monday, March 12
5:00 PM – 5:00 PM
Grand Hall

**Board 138. Laboratory-based Surveillance of *Salmonella* Infections in China in 2010**

**L. Ran**, Z. Wang, Y. Gao, Y. Feng, Z. Feng;
Chinese Center for Disease Control and Prevention, Beijing, China.

**Background:** Salmonellosis is a significant public health problem in China. To monitor the trends of *Salmonella* infection in China, a laboratory-based *Salmonella* sentinel surveillance program was launched in China since 2006. We described here the epidemiology, serotypes, antimicrobial susceptibility of *Salmonella* isolates found in our program in 2010. **Methods:** Six provinces (Chongqing,

Fujian, Guangdong, Hennan, Shanghai, Sichuan) in China are included in this program. *Salmonella* isolates from patients with diarrhea were sent from sentinel hospitals to local public health laboratories for confirmation, serotyping and antimicrobial susceptibility testing using standardized methods. **Results:** A total of 107 hospitals in 37 cities and prefectures from the 6 provinces provided isolates and epidemiologic data for analysis. Of all stool samples submitted to laboratories, 1,209 (3%) grew *Salmonella enterica*. Positive isolates were most commonly detected between May and October. The median age of infected patients was 5 years (ranging from 8 days to 80 years); 45% of the infections occurred in patients < 2 years old. Of the 1209 isolates, we found 89 serotypes, of which serotype Typhimurium (33%) and serotype Enteritidis (23%) were the most common. The overall prevalence of resistance was extremely high for conventional antimicrobial agents, including sulfamethoxazolum (64%), nalidixic acid (59%), ampicillin (49%), tetracycline (48%), streptomycin (46%), trimethoprimum (35%), chloramphenicols (31%) and gentamicin (27%), but relatively low for new antibiotics, including cefotaxime (7%), ceftazidime (6%) and ciprofloxacin (4%). The resistant rates for specific antimicrobial agents in Typhimurium strains were dramatically higher than those in all tested strains as well as those in Enteritidis strains. Roughly 55% of isolates, including 78% of all Typhimurium strains, 56% of all Enteritidis strains, were resistant to three or more antimicrobial agents. **Conclusions:** In this national surveillance report of laboratory-confirmed *Salmonella* infections in China in 2010, we found that Typhimurium and Enteritidis are the most common serotypes, and that efforts to reduce antimicrobial resistance among *Salmonella*, especially *Salmonella* enterica Typhimurium in China are needed.

**Board 139. An Outbreak of *E. coli* O157:H7 Infections Linked to Commercial Strawberries Contaminated by Deer**

**M. Laidler**, W. Keene;
Oregon Public Health Divison, Portland, OR, USA.

**Background:** In August of 2011, routine follow-up to a pulsed-field gel electrophoresis (PFGE) cluster of *E. coli* O157:H7 ("O157") cases led to the identification of an outbreak among residents of northwestern Oregon. We describe the epidemiological, environmental, and laboratory investigation of this event. **Methods:** Confirmed cases were defined as persons with a positive O157 stool culture and PFGE pattern 1108OREXH-1; household members with compatible illness were considered presumptive cases. Cases were interviewed using standard hypothesis-generating questionnaires. A case-control study using age-, sex-, and neighborhood-matched controls was conducted. Sales tracebacks identified suppliers. Environmental samples (water, soil, vegetation, deer feces) were collected and screened for O157 by PCR and culture. **Results:** We identified 15 cases (14 confirmed) with onsets from July 10-29, 2011, in 5 Oregon counties. The median age was 68 (range, 4-85); 11 cases were female. Four persons developed hemolytic uremic syndrome (HUS); 2 died. All but one case reported consumption of "local" strawberries (i.e., those purchased from roadside stands, on-farm stands, or farmers's markets). Consumption of local strawberries was associated with increased risk of illness (matched odds ratio 19.61; 95% CI 2.93, Infinity; $p < 0.001$). Tracebacks implicated a specific Oregon farm. Strawberries from Farm A were sold at ≥56 outlets in Oregon and Washington. O157 was cultured from 21 (5%) of 464 environmental samples collected at Farm A; 6 of 10 typed by PFGE were indistinguishable from cases. Fields from farm A were contaminated with deer feces (range, 5-588 visible deer fecal deposits/hectare; median, 274). Only samples containing visible fecal material were positive. **Conclusions:** The outbreak was caused by consumption of strawberries contaminated by deer, a previously implicated source of O157. Control of produce contamination by wild cervids may require aggressive and potentially costly measures.

**Board 140. Occurrence of Shiga Toxin-producing *Escherichia coli* and *Salmonella enterica* in Domestic and Wild Canid Populations in a U.S.-Mexico Desert Southwest Produce Production Region**

**A.M. Fisher**[1], Y. Liu[1], A. Thiptara[1,2], T. Nguyen[1], M. Jay-Russell[1];
[1]Western Institute for Food Safety and Security, University of California, Davis, Davis, CA, USA, [2]Epidemiology Section, Veterinary Research and Development Center (Southern Region), Nakhonsithammarat, Thailand.

**Background:** There is limited information on the importance of animal reservoirs in the transfer of zoonotic enteric pathogens to fresh produce in the desert southwest, a major production region recently linked to a lettuce-related outbreak of *Escherichia coli* O145 infections. Due to frequent stray dog and coyote sightings near produce fields along the U.S.-Mexico border, we conducted a survey to determine the prevalence of shiga toxin-producing *E. coli* (STEC) and *Salmonella enterica* in these populations. **Methods:** From November 2010 to May 2011, fecal samples from 358 impounded dogs at three county animal shelters in the U.S. and Mexico, and 103 fresh coyote scat samples from lands near produce fields, were collected. STEC and *Salmonella* were cultured by extended enrichment and serogroup specific IMS followed by serotyping. **Results:** *E. coli* O157 was isolated from 2 (1.9%) coyote and no shelter dog samples. Isolates belonging to serogroups O26, O103, O113, and O145 were recovered from 60 (16.8%) dog and 13 (12.6%) coyote samples. All *E. coli* strains were *stx*1 and *stx*2 negative; 8 strains were positive for other virulence determinants (*eae*, *hyl*A). *Salmonella* was cultured from 33 (9.2%) dog and 33 (32%) coyote samples. Overall, 29 *Salmonella* serotypes were found, with ~60% from dogs belonging to serotypes Senftenberg or Typhimurium. After adjusting for age, the odds of a dog from the Mexican shelter being *Salmonella* positive was 4.93 (P=0.005, 95% CI: 1.63, 14.92) times higher than one of the two U.S. shelters. No statistically significant difference was observed between the number of *Salmonella* positive dogs and age, gender, body weight or reason impounded. **Conclusions:** Our findings suggest that stray dogs and coyotes in the desert southwest are not significant reservoirs of STEC, although the public health significance of shiga toxin-negative *E. coli* belonging to serogroups O157, O145, O103, O113, and O26 is unclear. In contrast, *Salmonella* was prevalent in these shelter dog and coyote populations. We speculate that the high rates of *Salmonella* recovery from stray dogs and coyotes may be due to their hunting and scavenging behavior. These results underscore the importance of good agriculture practices relating to mitigation of animal intrusions into fresh produce fields.

**Board 141. Epidemiology of Foodborne Outbreaks Due to *Clostridium perfringens*, United States, 1998-2008**

**J.E. Grass**[1,2], B.E. Mahon[1], L.H. Gould[1];
[1]Centers for Disease Control and Prevention, Atlanta, GA, USA, [2]Atlanta Research and Education Foundation, Decatur, GA, USA.

**Background:** *Clostridium perfringens* is estimated to be the third most common cause of foodborne illness in the United States, causing one million illnesses each year. **Methods:** Local, state, and territorial health departments voluntarily report foodborne *C. perfringens* outbreaks to CDC through the Foodborne Disease Outbreak Surveillance System. Outbreaks during 1998-2008 confirmed by laboratory evidence were included in this analysis. A food item was implicated if *C. perfringens* was isolated from food or based on epidemiologic or laboratory evidence. Implicated foods were classified into one of 17 standard food commodities when all ingredients belonged to the same commodity. **Results:** From 1998-2008, 253 confirmed outbreaks of *C. perfringens* illness were reported with 13,182 illnesses, 74 hospitalizations, and 5 deaths. The number of outbreaks reported each year ranged from 16 to 31 with no apparent trend. The annual number of outbreak-related illnesses ranged from 359 to 2,173. The median outbreak size was 24 illnesses (range: 2-950). The number of *C. perfringens* outbreaks varied by season, with most occurring during the fall (71 outbreaks, 28%), followed by spring (66 outbreaks, 26%);

winter (61 outbreaks, 24%); and summer (55 outbreaks, 22%). Restaurants (44%) were the most common setting of food preparation. Other preparation settings included catering facility (19%), private home (13%), prison or jail (11%), and school (4%). Among the 128 (51%) outbreaks attributed to a single food commodity, beef was the most common commodity (59 outbreaks, 46%), followed by poultry (38 outbreaks, 30%), and pork (20 outbreaks, 16%). Combined, meat-poultry commodities accounted for 91% of outbreaks with an identified single food commodity. **Conclusions:** Outbreaks caused by *C. perfringens* occur regularly, are often large, and cause substantial morbidity. The vast majority are linked to the consumption of contaminated meat and poultry products, suggesting enhanced efforts at slaughter are warranted to minimize contamination of these foods. Proper cooking, cooling, and hot holding can decrease the growth of *C. perfringens* and the formation of toxin in food. Adherence to these recommendations, especially in restaurants and catering facilities, could decrease the number of outbreaks and illnesses.

**Board 142. Increasing Number of Listeriosis Outbreaks Associated with Mexican-style Cheese Made from Pasteurized Milk**

**K.A. Jackson**, S.D. Johnson, L.H. Gould, B.J. Silk;
Centers for Disease Control and Prevention, Atlanta, GA, USA.

**Background:** Listeriosis is caused by *Listeria monocytogenes,* a gram-positive bacillus transmitted primarily through food. Mexican-style cheese made from unpasteurized milk is a well established cause of listeriosis outbreaks. Pasteurization kills *Listeria*; however contamination can occur after pasteurization. **Methods:** We queried CDC's Foodborne Disease Outbreak Surveillance System (FDOSS), an electronic surveillance system to which states voluntarily submit reports of foodborne outbreaks, for all listeriosis outbreaks associated with pasteurized dairy products from 1985 to 2010. FDOSS captures information on etiology, food vehicle, aggregate age group and sex data, and number of illnesses, hospitalizations, and deaths. Additional data were reviewed from published reports and unpublished CDC data. Cases in pregnant women or infants ≤28 days old were considered pregnancy-associated. **Results:** From 1985 to 2010, 9 outbreaks of listeriosis with 247 illnesses associated with pasteurized dairy products were reported. Numbers of ill patients ranged from 2 to 142 (median 6). All 7 outbreaks with available data were associated with domestically-produced commercial products. Five outbreaks (168 cases) were associated with commercial Mexican-style cheese; 4 occurred after 2007. Among the 4 outbreaks associated with Mexican-style cheese with available data, the percentage of pregnancy-associated cases ranged from 66% to 88% (median 86%). Ethnicity data were available for 17 cases; all were Hispanic. Fifteen (58%) patients were hospitalized; there were 33 fetal or neonatal deaths. Other vehicles included milk (2 outbreaks) and unspecified types of cheese (2). Processing practices reported as possibly contributing were inadequate pasteurization, poor sanitation, and insufficient separation between raw milk processing and packaging of finished product. **Conclusions:** The number of reported listeriosis outbreaks associated with Mexican-style cheese made from pasteurized milk has increased in recent years; reasons for this increase are not well understood and may be due to many factors. These outbreaks disproportionately affect pregnant, Hispanic women. Adherence to pasteurization protocols and prevention of contamination after pasteurization are essential to prevent future outbreaks.

**Board 143. Aichivirus, Parechovirus, and Bocavirus in Children < 5 years Hospitalized for Acute Gastroenteritis in the United States: A Case Control Study**

**P. Chhabra**[1], D. Payne[1], M.A. Staat[2], K.M. Edwards[3], P.G. Szilagyi[4], A. Nix[1], X. Lu[1], U.D. Parashar[1], J. Vinjé[1];
[1]Centers for Disease Control and Prevention, Atlanta, GA, USA, [2]Cincinnati Children's Hospital Medical Center, Cincinnati, OH, USA, [3]VanderBilt University Medical Center, Nashville, TN, USA, [4]University of Rochester School of Medicine and Dentistry, Rochester, NY, USA.

**Background:** Norovirus (NoV), rotavirus, sapovirus, astrovirus, and adenovirus are established viruses associated with acute gastroenteritis (AGE) in children worldwide. Other enteric viruses such as aichivirus (AiV), human parechovirus (HPeV), and human bocavirus (BoV) have been frequently detected in fecal specimens. However, their association with the disease is not yet established as most studies focus on symptomatic individuals and lack data of age-matched asymptomatic controls. The New Vaccine Surveillance Network (NVSN) conducted year-round active population-based surveillance for AGE in hospital and emergency department settings (and for healthy controls) in 2008-2009 among children < 5 years of age. **Methods:** A total of 991 fecal specimens collected from 500 children (<5 years of age) with AGE appearing in clinics, emergency departments, or inpatient settings of hospitals in 3 counties (Rochester NY, Nashville TN, and Cincinnati, OH) and 491 asymptomatic children with no history of diarrhea or vomiting within 10 days. All the specimens were tested for the presence of AiV, HPeV and BoV by real-time (RT) PCR. Genotyping of each virus was carried out by conventional nested (RT) PCR assays. **Results:** At least 1 viral agent was detected in 57 (5.7%) of 991 fecal specimens. These included 23 (4.6%) and 34 (6.9%) specimens from AGE patients and asymptomatic controls, respectively. HPeV (4.4%) and BoV (2.4%) were found more prevalent in asymptomatic controls as compare to symptomatic patients (3.4% and 0.6%, respectively). AiV was detected only in symptomatic patients and in a very low (0.6%) prevalence. All AiV and 76.4% of the HPeV positive specimens from AGE patients showed mixed infections with other viral AGE pathogens. The majority (86.6%) of the children with HPeV and BoV infections experienced mild to moderate AGE symptoms. HPeV infections were detected from April to December while BoV infections were detected from December to June. Four HPeV (HPeV1, HPeV3, HPeV4, HPeV5) and 2 BoV (BoV1, BoV3) genotypes were found circulating in the study population. **Conclusions:** Overall, the study does not support a causative role of AiV, HPeV, and BoV in AGE in children < 5 years of age in the US.

## Healthcare-Associated Infections

Monday, March 12
5:00 PM – 6:00 PM
Grand Hall

### Board 144. Etiology and Incidence of Nosocomial Viral Respiratory Illnesses among Pediatric Patients in Tertiary Care Hospitals in Bangladesh, 2008–2011

**M.U. Bhuiyan**[1], S.P. Luby[1,2], R.U. Zaman[1], M.W. Rahman[1], M.J. Hossain[1], M. Rahman[1], T. Azim[1], K.S. Ramirez[1,2], M. Rahman[3], E.A. Baumgartner[1,2], E.S. Gurley[1];
[1]International Centre for Diarrheal Disease Research, Bangladesh, Dhaka, Bangladesh, [2]Centers for Disease Control and Prevention, Atlanta, GA, USA, [3]Institute of Epidemiology Disease Control and Research, Dhaka, Bangladesh.

**Background:** Children admitted to resource-poor health facilities are at risk for acquiring nosocomial respiratory illnesses because of limited personal hygiene, patient overcrowding and poor infection control. This study aimed to determine the etiology and incidence of nosocomial viral respiratory illnesses among pediatric patients in Bangladesh. **Methods:** During May 2008 - April 2011, surveillance physicians at 3 tertiary hospitals in Bangladesh identified patients who were admitted in pediatric wards and developed nosocomial respiratory illness. We defined nosocomial respiratory illness as new onset of fever, cough, runny nose, or difficult breathing after 72 hours of hospitalization. Nasopharyngeal and throat swabs were collected from those patients and tested for a panel of common respiratory viruses including influenza virus, respiratory syncytial virus (RSV), parainfluenza virus type 1, 2, and 3 (HPIV) and human metapneumovirus (HMPV) using real-time RT-PCR in the ICDDR,B laboratory. We calculated the

incidence of nosocomial respiratory viral illness per 1000 patient days at risk. **Results:** A total of 8,196 children were hospitalized for >72 hours accounting for 32,944 patient days at risk; 313 children (4%) had nosocomial respiratory illnesses with an incidence of 9 per 1000 patient days. We collected specimens from 294 children; 86 (29%) had evidence of viral pathogens that we tested for: 24 (28%) had influenza virus, 21 (24%) HMPV, 18 (21%) HPIV type 3, and 15 (17%) had RSV. The incidence of nosocomial respiratory illnesses associated with a respiratory virus was 3 per 1000 patient days. There were 4 (1%) deaths among cases; 1 was infected with influenza A/H1, 1 with HMPV, and 2 tested negative for respiratory viruses. **Conclusions:** One third of nosocomial respiratory illnesses among children in tertiary hospitals in Bangladesh were associated with a respiratory viral pathogen. Nevertheless, we did not find evidence of the viral pathogens in 71% of children, suggesting that many of these illnesses could be caused by bacteria or other viruses that we did not test for. Further studies to better characterize the etiology of nosocomial respiratory illnesses could help to develop infection control interventions and prevent spread of respiratory pathogens in this setting.

**Board 145. Incidence of Hospital-Acquired Acute Respiratory Illness on Pediatric and Adult Medical Wards in Egypt and Jordan**

**M. Curless**[1], T. Saied[1], A. El Kholy[2], M. Abdallat[3], S. Hafez[4], M.G. El Anany[2], M.A. Younan[2], H.E. El Hagar[2], N. Jarour[3], B.L. House[1], M. Talaat[1];
[1]Naval Medical Research Unit No. 3 (NAMRU-3), Cairo, Egypt, [2]Cairo University Hospitals, Cairo, Egypt, [3]Ministry of Health, Amman, Jordan, [4]Alexandria University Hospitals, Alexandria, Egypt.

**Background:** Hospital-acquired respiratory infections cause considerable illness and mortality especially in resource-limited countries where infection control programs are evolving. We describe the epidemiologic characteristics and etiologic spectrum of hospital-acquired viral respiratory infections in Egypt and Jordan. **Methods:** Prospective active surveillance was conducted at 4 university hospitals in Egypt and one general hospital in Jordan between 11 March 2010 and 31 July 2011. Patients admitted for 3 or more calendar days on 15 adult and pediatric medical wards were screened daily by hospital infection control teams for influenza like illness (ILI) symptoms (fever ≥38°C, and cough or sore throat). Basic epidemiologic and outcome data was collected on standardized forms; viral etiology was determined through RT-PCR on nasopharyngeal (NP) and oropharyngeal (OP) swabs. **Results:** A total of 8826 patients (6039 Egypt, 2787 Jordan) were screened; 259 (2.9%) episodes of hospital-acquired ILI developed in 251 patients. Among infected patients the median age was 1.5 years (range 2 days - 77 years) and 124 (49%) were females. The incidence of hospital-acquired ILI was 4.9 and 4.6 / 1000 patient days in Egypt and Jordan respectively. Incidence was significantly higher in pediatric wards (7.3/1000 patient days) than in adult wards (1.7 /1000 patient days) (P <0.01). The median time to develop hospital-acquired ILI was 4 days after hospital admission (range 4 - 29 days). No significant difference was observed between mortality of patients with ILI (1.9%) and without (1.6%). Mean hospital stay was significantly longer in patients with hospital-acquired ILI (10.9 days ± 6.8) compared to those without infection (9.7 days ± 6) (CI 0.96-1.51; P < .01). Of the 259 episodes of ILI, 85 (34%) provided NP/OP swabs. Among these, 29 (34%) tested positive for at least one viral pathogen: 10 parainfluenza virus 3, 8 RSV, 5 influenza A (1 pH1N1 2009, 4 seasonal H3N2), 3 adenovirus, 2 influenza B and 1 human metapneumovirus. **Conclusions:** High transmission of hospital-acquired viral respiratory infections was observed on pediatric wards in Egypt and Jordan. Enforcing respiratory infection control measures to prevent the spread of hospital-acquired viral infections should be encouraged with special emphasis on pediatric wards.

**Board 146. Prevalence of Healthcare-Associated Infections in a Tertiary Hospital in Southwest China**

**Z. Zong**, F. Qiao, W. Yin, S. Xu, D. Pu;
West China Hospital of Sichuan University, Chengdu, China.

**Background:** Healthcare-associated infections (HAI) are an under-recognized problem in China. Healthcare utilization is high with over 80% occupancy rate for inpatient beds and an average length of stay of 11 days. Infection control is particularly challenging, in part due to a dearth of information regarding HAIs in China. We conducted a point prevalence survey at West China Hospital, a tertiary hospital with 4,400 beds serving as the referral medical center in southwest China, to understand the prevalence and evaluate the burden of HAIs. **Methods:** All patients hospitalized or discharged on June 25, 2011 were included in the survey. Patients were examined at bedside for signs and symptoms of infection. Study physicians abstracted medical records using standard medical record abstraction forms which included questions on underlying illnesses, demographics, and antibiotic use. An HAI was defined by diagnostic criteria set by the Chinese Ministry of Health. All data were double inputted into Epidata and analyzed using the SPSS software. **Results:** A total of 4,552 out of 4,625 eligible patients (98.4%) were surveyed. The prevalence of all HAI was 4.68%. Lower respiratory tract infection including pneumonia was the most common type and accounted for 49.3% of all HAI, followed by surgical site infection (14.42%), urinary tract infection (11.63%) and upper respiratory tract infection (8.84%). The prevalence of HAI at 10% or more was seen in neurological ICU (45.83%), medical ICU (32.43%), surgical ICU (21.74%), Chinese medicine (21.31%), geriatrics (11.86%), orthopedics (10.64%), neurology (10.31%), nephrology (10.29%) and chest ICU (10%). **Conclusions:** The survey showed that HAIs remain a serious challenge locally, particularly in ICUs. Lower respiratory tract infection was the most common type of infection, accounting for an half of all HAIs. Interventional measures against HAIs, particularly those in ICU and lower respiratory tract infection, should therefore be implemented.

**Board 147. Diarrheal Illness among US Residents Providing Medical Services in Haiti during the Cholera Epidemic, 2010-2011**

**K. Schilling**[1], E. Cartwright[1,2], J. Stamper[1], M. Locke[1], D. Esposito[1], V. Balaban[1], E. Mintz[1];
[1]Centers for Disease Control and Prevention, Atlanta, GA, USA, [2]Emory University School of Medicine, Division of Infectious Diseases, Atlanta, GA, USA.

**Background:** On October 21, 2010, for the first time in over a century, epidemic cholera was confirmed in Haiti. In response, cholera treatment centers (CTCs) were established throughout the country. CTCs were primarily run by international non-governmental organizations (NGOs), and staffed by foreign healthcare workers (HCWs). Although cholera transmission to HCWs is rare, CDC was informed of 2 HCWs who became ill with cholera after providing medical services in Haiti. **Methods:** To assess the incidence of diarrheal illness (defined as: $\geq$ 3 loose stools/24 hours) among HCWs that occurred in Haiti or within 5 days of returning, we conducted a cross-sectional, web-based survey. US residents who provided medical services in Haiti between October 21, 2010 and May 31, 2011 were eligible to participate. **Results:** We emailed 896 participants from 50 US-based, health-focused NGOs; 381 (44%) completed the survey. The median age of respondents is 47 years (range 19 -81 yrs); 65% were female. Most respondents traveled to Haiti once (n=322, 85%); 59 (15%) went ≥2 times. The median trip duration was 8 days. Among respondents, 153 (40%) were trained as nurses, 117 (31%) as medical doctors, 51 (13%) as allied health professionals (AHP); 60 (16%) had no formal medical training. Overall, 311 (82%) reported always washing their hands after patient care activities. Among those who did not always wash, lack of clean water or hand sanitizer was the most common reason (n=30, 43%) cited. Diarrhea was reported by 31 (8%) respondents (9% MDs, 8% nurses and AHP, and 7% with no medical training). Of these, 17 (55%) reported taking oral antibiotics and 4 (13%) sought medical care, including one medical evacuation. Use of a chronic, acid-suppressing medication was not significantly different amongst those who developed diarrhea (n= 5, 16%) when compared with those who didn't (n=59, 17%). **Conclusions:** US HCWs traveled to Haiti to respond to the cholera epidemic. While US HCWs temporarily increased healthcare capacity, the relatively short trip duration implies frequent staff turnover. Lack of handwashing supplies may have led to diarrheal illnesses experienced by some HCWs and could have

contributed to other nosocomial infections. To reduce the incidence of diarrheal illness in HCWs working in Haiti, NGOs should ensure ample access to handwashing supplies.

**Board 148. When One Case of Hepatitis C Becomes an Outbreak**
**E. Bancroft**, S. Hathaway, A. Itano;
Los Angeles County Department of Public Health, Los Angeles, CA, USA.
**Background:** Poor injection practices have been associated with the transmission of hepatitis B (HBV) and hepatitis C (HCV). In August 2010, we investigated a case of acute HCV in a patient whose only risk factor was receiving multiple injections at a pain clinic. **Methods:** We performed a search of our hepatitis registry to identify other pain clinic patients that have been reported with HBV or HCV. We observed injection procedures at the pain clinic. We obtained blood from the index patient and a possible source patient with chronic HCV. The blood was sent to CDC for genetic analysis. We notified other patients of their potential risk to bloodborne pathogens and encouraged them to be tested by their healthcare providers. **Results:** We identified a patient with chronic HCV who had received an injection at the pain clinic immediately preceding the index patient. We also identified a patient in the registry with acute HBV who had received injections at the pain clinic during his incubation period and had no other risk factors for HBV. We observed a nurse inject a medication into a heplock of a patient and then use the same needle and syringe to access a multidose vial of saline that was to be used for other patients. Multiple other infection control breeches were also observed. Both HCV patients had genotype 2b and several hepatitis C virus variants with identical sections of the viral genome; this was consistent with both patients being infected with the same strain of HCV. We notified 2,300 patients that had received injections from this nurse during the 4 years she had worked at the clinic. In the hepatitis registry, a total of 59 (2.6%) were reported with a positive test for either HBV or HCV before notification. Within 2 months after the notification, 18 patients from the pain clinic were newly reported with HCV and 1 was reported with HBV. **Conclusions:** There was at least one case of patient to patient transmission of HCV at the pain clinic, most likely due to poor injection practices. Given the baseline prevalence of HBV and HCV in this patient population, the 19 newly identified HBV and HCV cases represent a minimum of 730 patients tested. However, because of intermittent exposure, lack of other risk exposure histories, and inability to genotype, we cannot definitively link the newly reported cases to transmission at the pain clinic.

## Foodborne and Waterborne Infections

Tuesday, March 13
12:30 PM – 1:30 PM
Grand Hall

**Board 149. Antibiotics Sensitivity Profile of Microorganisms Encountered in the Riverine Areas of Ondo State, Nigeria**
**O.A. Ajayi**;
Adekunle Ajasin University,, Akungba-Akoko, Ondo State, Nigeria.
**Background:** This study shows the antibiotics susceptibility pattern and complexity of micro-organisms encountered in ecological zones of the oil producing riverine areas of Ondo state. Nigeria. **Methods:** The water and soil samples obtained from these riverine areas were serially diluted and appropriate diluents was inoculated in sterile petri-dish plates and cultured at $37^0$c for 24 hours using a pour plate technique. The *in vitro* antibiotic susceptibility testing of bacterial isolates was performed using the standardized disc agar diffusion method to determine the sensitivity pattern of the test organisms. **Results:** The

species of organisms encountered are *Micrococcus spp.* 9 (34.6%) which was the predominant bacteria isolated from the soil and water samples. Similarly, *Pseudomonas spp*. 5, (19.2%) was from soil and water samples. *Bacillus spp.* 5 (14.3%), *Bacillus circulans,* 2, (5.7%), *Bacillus cereus* and *Bacillus subtilis* having 1 (11.1%) isolate each, was also among the major isolates from the soil samples. *Micrococcus luteus,* 1, (11.1%) was isolated from fish and crab samples. Some other groups of bacteria generally isolated were, *Proteus spp, Klebsiella spp, Streptococcus spp, Veillonela spp, E. coli* with 1 (11.1%) isolate respectively and *Proteus vulgaris* 2 constitute 22.2%. The isolates showed wide range of antibiotic sensitivity towards some of the antibiotics used including Gentamicin (10-27mm), Cotrimoxazole (up to 27mm) and Perfloxacin (up to 30mm), while Ofloxacin and Ciprofloxacin also have values up to 30mm inhibition zone. Many isolates were susceptible in good range to antibiotics during the study as recorded in the antibiogram (167 out of 171). This include *Micrococcus spp* (22.2%), *Pseudomonas spp* (18.1%) and *Bacillus spp* (16.2%) having the highest degree of sensitivity. Others are *Pseudomonas aeruginosa* and *Proteus vulgaris* (5.8%), *E. coli* (4.7%), *Bacillus subtilis* (1.8%). **Conclusions:** Diversified organisms with multiple antibiotic resistance were encountered during the study. About 50% of the antibiotics used were effective against most of the tested isolates. The results signify the need for environmental monitoring and susceptibility tests before administration of antibiotics in health management systems in this area.

**Board 150. The Increasing Problem of *Salmonella enterica* serotype Newport in Infants and in the South, United States**

**S.J. Chai**[1], S. Crim[1], A. Nisler[2], J. Reynolds[2], K.C. Swanson[2], L.H. Gould[1], B. Karp[1];
[1]Centers for Disease Control and Prevention, Atlanta, GA, USA, [2]Atlanta Research and Education Foundation, Atlanta, GA, USA.

**Background:** In the 10 sites of the Foodborne Diseases Active Surveillance Network (FoodNet), incidence of laboratory-confirmed human infections from *Salmonella enterica* serotype Newport (Newport) has been increasing. In 2010, Newport was the second most commonly reported *Salmonella* serotype, surpassing Typhimurium for the first time since surveillance began in 1996. **Methods:** We compared data on Newport during 2004-2010 from FoodNet and 3 national human surveillance systems: Laboratory-based Enteric Disease Surveillance (LEDS), the Foodborne Diseases Outbreak Surveillance System (FDOSS), and the National Antimicrobial Resistance Monitoring System (NARMS). NARMS tests every 20$^{th}$ human non-typhoidal *Salmonella* isolate received by public health laboratories for antimicrobial sensitivity. We examined data by demographic characteristics, geographic location, and antimicrobial resistance phenotype. Changes between 2004 and 2010 were calculated from a least-means squared line through the plotted 2004-2010 data. **Results:** During 2004-2010, Newport incidence in FoodNet and LEDS was consistently higher in infants than in other persons and higher in the South than in other regions. Percentage increases in incidence were also highest among infants (92% FoodNet; 128% LEDS) and persons in the South (108% FoodNet; 45% LEDS). In NARMS, percentage increases in submission rates of pansusceptible isolates per 100,000 population were also higher among infants (622%) than other persons (56%) and higher in the South (87%) than in other regions (11%). No meaningful differences or changes over time were observed when data were examined by sex, race, and ethnicity, or in numbers of reported Newport outbreaks or associated illnesses. **Conclusions:** The incidence of Newport infections is highest and has increased most in infants and persons in the South. Because resistant Newport infections have been linked to food animal sources, one hypothesis for the increasing submission rates of pansusceptible Newport is that exposures to sources other than food animals (e.g., the environment) are increasing, especially in infants and in the South. Studies of exposures to which infants may be particularly susceptible and exposures among residents of and visitors to the South may help to elucidate the sources and focus control efforts.

**Board 151. Hospitalization Rates of Major Foodborne Diseases in Japan**

**H. Toyofuku**;
National Institute of Public Health, Wako, Japan.

**Background:** Previously there were no hospitalization data of foodborne diseases in Japan because these data are not mandatory to report. To this estimate the leading cause of hospitalization and hospitalization rates of major foodborne pathogens, the detailed food borne outbreak investigation reports (DFBOVR) which are required for certain significant pathogens and large outbreaks, and may contain the number of cases who were hospitalized were analyzed. **Methods:** DFBOVR submitted to the Minister of Health, Labor and Welfare by governors of prefectures and mayors of big cities in 2006 were collected, and reports which contained hospitalization data were analyzed by pathogens. **Results:** Total 54 DFBOVRs contained the number of cases who were hospitalized. The leading causes of hospitalization were nontyphoidal *Salmonella* spp. (36%), *Campylobacter* spp. (28%), norovirus (22%), and STEC O157 (12%). The hospitalization rates of these pathogens were 36.2%, 12.8%, 1.26% and 14.8%, respectively. **Conclusions:** This is the first attempt to analyze the hospitalization rates of major foodborne pathogens in Japan. The order of the leading causes of hospitalization is very similar to that of published in the United State. Even though data limitation due to the use of outbreak data needs to be considered, still this dataset is reasonable estimations of the hospitalization rates, and could be used to evaluate severities of these foodborne diseases and to prioritize risk management activities to reduce public health risk associated with foodborne diseases.

**Board 152. The National Outbreak Reporting System: Preliminary Results of the First Year of Surveillance for Multiple Modes of Transmission—United States, 2009**

**K.L. Manikonda**[1,2], M.E. Wikswo[1], V.A. Roberts[1], L. Richardson[1,2], L.H. Gould[1], J.S. Yoder[1], A.J. Hall[1];
[1]Centers for Disease Control and Prevention, Atlanta, GA, USA, [2]Atlanta Research and Education Foundation, Decatur, GA, USA.

**Background:** The Centers for Disease Control and Prevention (CDC) has conducted national surveillance for foodborne and waterborne disease outbreaks reported by local and state health departments since the early 1970s, initially using paper-based reporting systems. The National Outbreak Reporting System (NORS) launched in 2009 as a web-based reporting system for all waterborne disease outbreaks and enteric disease outbreaks involving foodborne, person-to-person, animal contact, environmental contamination, and unknown modes of transmission. **Methods:** We analyzed all reported outbreaks that occurred in 2009 by primary mode of transmission, etiology, and setting. We restricted the analysis to single-etiology outbreaks for which the etiology was confirmed or suspected. **Results:** During 2009, 1,219 single-etiology outbreaks were reported by 51 states and U.S. territories (range 1-122 outbreaks), resulting in 34,160 outbreak-associated illnesses, 999 hospitalizations, and 48 deaths. The primary mode of transmission was person-to-person in 618 (51%) outbreaks, foodborne in 458 (38%), animal contact in 24 (2%), waterborne[†] in 17 (1%), and environmental contamination in 3 (0.2%). The remaining 99 (8%) outbreaks were reportedly of unknown mode of transmission. The most common pathogen reported among all single-etiology outbreaks was norovirus (280 outbreaks, 23%), followed by *Shigella* (72, 6%) and *Salmonella* (57, 5%). The most frequently reported settings of outbreaks among all modes of transmission were nursing homes (250 outbreaks, 21%), restaurants (208, 17%), and private homes (124, 10%). **Conclusions:** NORS provides the first U.S. comprehensive national surveillance system for waterborne and enteric disease outbreaks and highlights the tremendous public health burden these outbreaks represent. By collecting data on outbreaks with different modes of transmission into one system, NORS provides valuable insights into pathogens that have multiple routes of transmission and can help identify priority pathways and settings for public health interventions.

[*]Data are preliminary since 45% of states have not completed reporting.

[†]Waterborne counts are expected to be low due to a later data-close out schedule.

**Board 153. Changes in Foodborne Disease Outbreak Pathogens and Implicated Foods—United States, 1998-2008**

**K.A. Walsh**[1], K.M. Herman[2], A.R. Vieira[3], L. Simmons[3], A.J. Hall[2], D. Cole[2], L.H. Gould[2]
[1]Atlanta Research Education Foundation, Atlanta, GA, USA, [2]Centers for Disease Control and Prevention, Atlanta, GA, USA, [3]Oak Ridge Institute for Science and Education, Atlanta, GA, USA.

**Background:** Foodborne disease outbreaks are an important public health burden. Outbreak surveillance and trends analysis provides insights into the pathogen-food pairs for which effective control strategies have been implemented or might be of benefit. **Methods:** We reviewed data on foodborne outbreaks (≥ 2 illnesses resulting from ingestion of a common food) reported to CDC's Foodborne Disease Outbreak Surveillance during 1998-2008. For food vehicles that could be classified in a single commodity, the mean proportion of outbreaks attributed to commodities and changes over time were evaluated. **Results:** There were 13,405 outbreaks reported, causing 273,120 illnesses, 9,109 hospitalizations, and 200 deaths. Among the 7,998 (60%) outbreaks and 204,048 (75%) associated illnesses with an etiology reported, most outbreaks were caused by viral (45%) and bacterial pathogens (45%). Norovirus infections caused 95% of viral outbreaks. The proportion of outbreaks with a reported etiology that were attributed to norovirus infections increased from 28% in 1998-2000 to 50% in 2007-2008. The most common bacterial pathogen was *Salmonella,* causing 1,449 outbreaks (40%). The proportion of outbreaks of *Salmonella* infections was similar over time (22% in 1998-2000 and 19% in 2007-2008). However, outbreaks caused by serotype Enteritidis infections decreased from 44% of outbreaks of *Salmonella* infections in 1998-2000 to 24% in 2007-2008. Outbreaks of *Salmonella* infections were most often attributed to poultry (30%) and eggs (24%). Outbreaks of norovirus infections were most often attributed to leafy vegetables (32%); a food worker was implicated in almost half of these outbreaks. The proportion of outbreaks of *Salmonella* infections attributed to eggs decreased from 35% in 1998-2000 to 17% in 2007-2008. The proportion of outbreaks of norovirus infections attributed to leafy vegetables was 30% in 1998-2000 and 42% in 2007-2008. **Conclusions:** Over these 11 years, the decrease in outbreaks of *Salmonella* infection attributed to eggs parallels the decrease in the outbreaks of serotype Enteriditis infections, suggesting food safety progress in this pathogen-food pair. Outbreaks of norovirus infections attributed to leafy vegetables may be on the rise and focus should be placed on prevention of infections from ill food workers.

**Board 154. Trichinellosis Hospitalizations in the United States, 2004-2008**

**J.L. Jones**;
Centers for Disease Control and Prevention, Atlanta, GA, USA.

**Background:** There is concern that field-raised "organic" livestock production and undercooking of meat could lead to an increase in trichinellosis. **Methods:** Using the National Inpatient Sample (NIS), a component of the Healthcare Cost and Utilization Project, Agency for Healthcare Research and Quality, we examined trichinellosis-associated hospitalizations for the 5-year period 2004-2008. The NIS is designed to represent a 20% sample of U.S. community hospitals and includes information on up to 8 million hospital discharges per year from approximately 1,000 hospitals. The NIS is weighted to produce national estimates. To identify trichinellosis-associated hospitalizations, we used ICD 9 CM code 124 in any hospital discharge summary diagnostic position. **Results:** The estimated hospitalizations and 95% confidence limits (CL) were as follows: 2004, 69 (33, 105); 2005, 59 (26, 91); 2006, 45 (15, 75); 2007, 48 (17, 78); 2008, 70 (34, 105). Over the 5-year period, 74% (95% CL 63%, 85%) of trichinellosis-associated hospitalizations were among women, 42% (95% CL 29%, 54%) among black persons, 25% (95% CL 14%, 36%) among white persons, 8% (95% CL 1%, 15%) among Hispanic persons, 5% (95% CL 0%, 10%) among Asian/Pacific Islanders, 2% (95% CL 0%, 5%) among Native Americans, and 18% among other races (including missing racial category). By age, a majority (56%; 95% CL 44%, 69%) of these hospitalizations occurred among persons 20-49 years old. **Conclusions:** There was not an overall increase in this 5-year

period; however, trichinellosis-associated hospitalizations continue to occur in the U.S. Most cases were in women and a large proportion in black persons. Although cases cannot be validated by independent medical record review, the NIS provides an opportunity for population-based monitoring of trichinellosis in addition to case reporting, which is often incomplete, and will help CDC to track the illness as meat production practices evolve.

## New or Rapid Diagnostics

Tuesday, March 13
12:30 PM – 1:30 PM
Grand Hall

**Board 155. Detection of *stx1* and *stx2* Genes of Enterohemorrhagic *Escherichia coli* using Real-time PCR**

**Q. Liu**, Y. Jin, Y. Chen;
Longgang Center for Disease Control and Prevention of Shenzhen, Shenzhen, China.

**Background:** Enterohemorrhagic *Escherichia coli* (EHEC) is an important human pathogen responsible for diarrhea, abdominal pain, and more serious complications such as hemorrhagic colitis and hemolytic-uremic syndrome (HUS). Traditional culture-based detection methods are time-consuming and detect only the O157:H7 *E. coli* serotype and neglect other EHEC serotypes. The objective of this study was to develop a real-time PCR assay for rapid detection of all serotypes of EHEC strains. **Methods:** Based on multiple-sequence alignments, the primers and Taqman probes were designed to specifically amplify *stx1* and *stx2* virulence genes in the same reaction. Report dyes of two probes were FAM and HEX, respectively, and quencher dye was BHQ1. These primer pairs and probes were combined for use in a duplex real-time PCR assay. We performed assays with genomic DNA from six EHEC strains and 46 non-EHEC strains. **Results:** We developed a duplex real-time fluorescence PCR assay for detecting *stx1* and *stx2* genes of EHEC strains. The results showed that the detection limit was about 100 genome copies per real-time PCR assay. No amplification was observed for all non-EHEC strains. The total time between preparation of DNA from EHEC colonies on agar plates and completion of real time PCR assay was less than 100 minutes. **Conclusions:** Our study demonstrated that developed duplex real-time PCR assay can be used as a simple, rapid, sensitive, and effective method for EHEC strain detection.

**Board 156. Whole Genome Sequencing and Digital DNA-DNA Hybridization for Determination of Novel Bacterial Species**

**B. Humrighouse**, A. Whitney, L. Helsel, A. Nicholson, D. Hollis, J. McQuiston;
Centers for Disease Control and Prevention, Atlanta, GA, USA.

**Background**: Identification of novel and rare bacterial species has been an important function of the Special Bacterial Reference Laboratory (SBRL) at CDC for more than six decades. The classic reference standard of new species identification has been DNA-DNA hybridization (DDH) using radioisotopic melting curve analysis. The traditional method is labor-intensive, so *in silico* DNA-DNA hybridization, using whole genome sequences, is an attractive replacement. Eight bacterial genera including three isolates previously classified as *Aggregatibacter* sp. were chosen for analysis in order to evaluate this methodology. Aggregatibacter, a genus comprised of Gram-negative rod-shaped bacterium, includes species *A. actinomycetemcomitans* and *A. aphrophilus*, which have been associated with periodontitus and infective endocarditis, respectively. *A. aphrophilus* has also been reported to be associated with brain abscess, as well as bone and joint infections. **Methods**: To evaluate digital DDH (DDDH) for new species classification, traditional DDH data for eight bacterial genera were compared to DDDH results.

Whole genome DNA extracted from isolates that had been phenotypically characterized as *Aggregatibacter* sp. was sequenced on Illumina and 454 next-generation sequencing platforms. Resulting concatenated sequences were analyzed using a web-based genome distance calculator (http://ggdc.gbdp.org) and compared to traditional DDH results. **Results**: Biochemical and 16S rRNA gene sequence results placed the unidentified *Aggregatibacter* species near *A. aphrophilus*. DDDH results correlated well with traditional results when NCBI-BLAST sequence similarity search algorithm and a distance formula for incomplete genomes were used. By DDDH, the unidentified *Aggregatibacter* isolates were delineated as a separate, novel species. **Conclusions**: Currently, classification of novel bacterial species requires the use of multiple assays including biochemical tests, 16S rRNA sequencing, and DDH. Our preliminary analysis suggests that DDDH using whole genome sequences will offer a more efficient method for new species identification than the traditional labor-intensive method.

**Board 157. Rapid Differentiation of *Bartonella* spp. Using a Real-time PCR and Pyrosequencing Based Algorithm**

S.N. Buss, **L.L. Gebhardt**, K.A. Musser;
New York State Department of Health, Wadsworth Center, Albany, NY, USA.

**Background:** The number of *Bartonella* species associated with human disease and the range of symptoms associated with *Bartonella* infections has risen dramatically since the 1990s. While serology is most commonly used to diagnose *Bartonella*, it does not reliably discriminate between *Bartonella* spp. Fast and accurate species differentiation could provide epidemiological insight and inform more effective treatment interventions. Here we describe a novel algorithm that combines real-time PCR and pyrosequencing for differentiation of medically relevant *Bartonella* spp. **Methods:** PCR negative whole human blood and CSF was spiked with *Bartonella henselae* (ATCC 49882) and ten-fold serial dilutions were prepared. DNA was then extracted from 130 µl of the spiked blood. DNA was also extracted from samples spiked with *B. quintana* (90-268)*, B. elizabethae* (F9251)*, B. clarridgeiae* (ATCC 51734)*, B. vinsonii* subsp. *arupensis* (OK 94-513), *B. vinsonii* subsp. *berkhofii* (ATCC 51672) and 20 different tick-borne pathogens or common PCR contaminants. The Masterpure™ DNA purification kit was used for all extractions. Samples were screened in a real-time PCR assay targeting a unique 72 bp region of the *Bartonella gltA* gene. A new pyrosequencing assay that examines a 272 bp segment of the *rpoB* gene was then used to differentiate among the *Bartonella* spp. **Results:** The limit of detection of the real-time PCR was 5 colony forming units and all 6 *Bartonella* spp. tested positive in the assay. None of the 20 non-*Bartonella* organisms in our specificity panel tested positive in the real-time assay. The pyrosequencing assay successfully discriminated between the 6 *Bartonella* spp. and did not cross-react with unintentional targets. At least 4 additional *Bartonella* spp. could be detected and differentiated using this algorithm, based on *in silico* analysis. In addition to blood and CSF, lymph nodes from two highly suspect patients were tested and found to be positive for B. henselae proving tissue to also be a valuable option for testing. **Conclusions:** Used together, the real-time PCR screening assay and the pyrosequencing assay described herein represent the first sensitive, specific and cost effective method for rapid differentiation of medically relevant *Bartonella* spp. from blood, CSF, and tissues.

**Board 158. Evaluation of Commercially Available Dengue Diagnostic Tests: NS1 and IgM Rapid Tests and NS1 ELISAs**

**E.A. Hunsperger**[1], S. Yoksan[2], P. Buchy[3], V. Nguyen[4], S. Sekaran[5], D. Enria[6], S. Vasquez[7], E. Cartozian[1], J. Pelegrino[7], H. Artsob[8], M. Guzman[7], M. Guillerm[9], J. Zwang[9], S. Kliks[10], R. Peeling[9], H. Margolis[1];
[1]Centers for Disease Control and Prevention, San Juan, PR, USA, [2]Mahidol University, Bangkok, Thailand, [3]Institute Pasteur Cambodia, Phnom Penh, Cambodia, [4]Hospital of Tropical Diseases, Ho Chi Minh City, Viet Nam, [5]University of Malaya, Kuala Lumpur, Malaysia, [6]INEVH, Pergamino, Argentina, [7]IPK, Habana,

Cuba, [8]Health Canada, Winnepeg, MB, Canada, [9]WHO/TDR, Geneva, Switzerland, [10]PDVI, Berkeley, CA, USA.
**Background:** The World Health Organization (WHO) with the Pediatric Dengue Vaccine Initiative (PDVI) established a network of 7 worldwide laboratories with dengue diagnostic expertise to provide an independent evaluation of currently available commercial kits for dengue diagnostics. Each laboratory contributed serum samples to develop a well characterized panel for testing dengue non-structural protein 1 (NS-1) antigen and dengue virus IgM antibodies (IgM anti-DENV). **Methods:** Asia and America region panels of similar composition were developed to evaluate NS1 and IgM anti-DENV test kits. The NS1 combined panel consisted of 192 sera from 147 patients defined as dengue positive and 142 negatives by culture or PCR. The IgM anti-DENV panel had 228 positive and 155 negative sera as defined by reference MAC ELISAs at Mahidol University and CDC. Seven companies submitted 3 NS1 microplate ELISAs, 1 IgM microplate ELISA, 4 NS1 rapid diagnostic tests (RDT) and 4 IgM anti-DENV RDTs for evaluation. All kits were evaluated at the network sites using region specific panels. The panel was coded so that the technicians performing the evaluation were blinded to the reference assay results. Evaluation results were analyzed to determine sensitivity, specificity, inter-laboratory agreement, inter-reader agreement, lot-to-lot variation and ease-of-use. **Results**: Results showed that 3 NS1 microplate ELISAs had sensitivity ranging from 46-52% and specificity of 71-80%. In comparison, the 4 NS1 RDTs had sensitivities ranging from 28-59% and specificity of 76-80%. Of the 4 IgM RDTs, sensitivities ranged from 52-95% with specificity from 86-92%. Sensitivity of IgM ELISA was 96% and specificity of 84%. **Conclusions:** The range of acceptable sensitivity and specificity from the combined Asia/America is being considered by a panel of experts convened by WHO/PDVI. The threshold for acceptable performance may vary by the purpose of testing and by local epidemiology and final recommendations will be distributed as a report upon completion.

## Improving Global Health Equity for Infectious Diseases

Tuesday, March 13
12:30 PM – 1:30 PM
Grand Hall

### Board 159. An Outbreak of Influenza B in an Isolated Nomadic Community in the Himalayan Mountains of Jammu and Kashmir, India

**P.A. Koul**[1], U.H. Khan[1], M.A. Mir[2], F. Ahmad[1], N.K. Bali[1], R.B. Lal[3], S. Broor[2];
[1]SheriKashmir Institute of Medical Sciences, Srinagar, India, [2]All India Institute of Medical Sciences, New Delhi, India, [3]Centers for Disease Control and Prevention, Atlanta, GA, USA.
**Background:** Community outbreaks of disease among nomadic populations with minimal access to healthcare generally remain undocumented. In May 2011, a spurt of acute respiratory tract infections (ARI) including four deaths was reported in the isolated nomadic population of Sangerwani in the Shopian district of Jammu and Kashmir, north India. We investigated this outbreak. **Methods:** Sangerwini (altitude 7500 ft) in the Himalayan Mountains consists of 13 villages with a total population of 17,573 nomads (31% aged 5-14 years). Each family has 4-12 members living in single room (*kothas*) of wood and stone/mud with no ventilation which is frequently shared with cattle housed towards a side separated on occasion by walls extending halfway to the ceiling. Dwellings are quite smoky due to wood burning for cooking or heating. A medical team attended the area from 14-23 May 2011. A total of 526 persons with ARI were screened and throat/pharyngeal swabs were obtained from 84 consecutive patients with influenza-like illness (ILI) (defined by fever >38 and a cough or sore throat) for influenza testing. The distribution of influenza virus types was compared with that among 108 patients with ILI at

a tertiary care centre in Srinagar (SKIMS, 70 km away) at the same time. **Results:** Of the 84 sampled (75% being <10yrs), 12 (14.3%) tested positive for influenza B but none for Influenza A. In contrast, only 1 of 108 (1%) ILI cases from SKIMS was positive for Influenza B, suggesting a localized outbreak in this isolated community of nomads living in the terrain with maximum temperatures ranging between 15 - 16$^0$C compared to 22$^0$C in the city. Symptoms included fever, chills, cough, shortness of breath, sore throat, nasal discharge, headache, body aches, fatigue, vomiting and diarrhea in variable frequency and all presented within 7 days of their illness. Chills were more frequent among influenza B positive patients but other clinical features were similarly dispersed. No chronic medical conditions were identified among the influenza B positive patients. Nearly 40% of patients with ILI and 25% of influenza B positives had evidence of familial clustering. **Conclusions:** This first report of confirmed influenza B in a nomadic population argues for adoption of surveillance strategies in tandem with infection control practices and vaccination.

**Board 160. Knowledge and Practices on Water Treatment, Sanitation, and Prevention in Resource-limited Areas of Port-au-Prince during a Cholera Outbreak, Haiti 2010**

**V.E. Beau De Rochars**[1], J. Tipret[2], M. Patrick[3], L. Jacobson[3], K. Barbour[3], D. Berendes[3], D. Bensyl[3], C. Frazier[4], G. Tabbal[5], J. Domercant[6], R. Archer[3], T. Roels[3], J. Tappero[3], T. Handzel[3];
[1]Centers for Disease Control and Prevention, Tallahassee, FL, USA, [2]GRET, Port-au-Prince, Haiti, [3]Centers for Disease Control and Prevention, Atlanta, GA, USA, [4]US Public Health Service, Tucson Area Indian Health Service, AZ, USA, [5]UNICEF, Port-au-Prince, Haiti, [6]Centers for Disease Control and Prevention, Port-au-Prince, Haiti.

**Background:** Cholera, an acute diarrheal disease, quickly spread throughout Haiti after the first case was reported in October 2010. By September 25, 2011, 401,527 cases and 6,002 fatalities occurred. Poorer neighborhoods in Port-au-Prince, known as resource-limited, were considered at higher risk due to high population density and lower water and sanitation services. In November, cholera prevention education campaigns and distribution of water purification tablets were implemented. To assess their impact, a household survey was conducted in resource-limited areas of Port-au-Prince in December 2010.
**Methods:** We used a multi-stage cluster survey design to select individual households for inclusion. In the first-stage sampling, a total of 27 clusters were selected from the sampling frame using population proportional to size sampling (PPS); 15 households were selected randomly per cluster. A standardized survey instrument was used to assess cholera knowledge, key prevention measures, access to safe water and sanitation, and receipt and use of household water treatment products. Frequencies and chi-square were used to describe practices and assess the differences. Household drinking water was tested for chlorine residual to assess use of purification tablets for household chlorination water during the cholera outbreak. **Results:** A total of 405 households were included in the survey. Cholera symptoms knowledge was high (diarrhea 89%, vomiting 85%) as was knowledge of mode of transmission (drinking contaminated water 72%). Although only 41.5% received water purification tablets, household-reported water treatment increased from 30% pre-outbreak to 73.9% post-outbreak (p-value<0.01). Overall, 59.5% of household drinking water samples were positive for residual chlorine indicating use of distributed or purchased (70.2%) self-reported water purification tablets. A great majority (99.4%) was willing to use the water purification tablets if they were available for them. **Conclusions:** Survey results indicate that cholera knowledge post-implementation was high and hygiene interventions increased access to potable water in Port-au-Prince resource-limited areas. Similar intervention strategies should be implemented in other high-risk populations of Haiti as part of the cholera response.

**Board 161. Preventing Complications of Hepatitis C: The Threat of Population Overweight**

**M. Klevens**, M. Denniston, D. Daniels, R. Jiles;
Centers for Disease Control and Prevention, Atlanta, GA, USA.

**Background:** In the United States, the obesity epidemic could exacerbate complications and costs associated with hepatitis C virus (HCV) infection. Infection guidelines recommend counseling obese persons to attempt to lose weight because overweight contributes to liver fibrosis and reduces treatment response. **Methods:** We analyzed data from the 2005-2008 National Health and Nutrition Examination Survey (NHANES), a complex, stratified, multistage probability sample of the non-institutionalized, civilian population of the United States. NHANES collects information using standardized household interviews, physical examinations, and testing of biologic samples. We calculated prevalence and odds ratios of selected health factors and characteristics. The main outcome was self-reported weight loss attempts in the past year. Body mass index (BMI) was categorized into 3-levels: normal (< 25), overweight (25-30), and obese ( >30). We defined a case of chronic HCV infection as a person who tested positive for both antibody to HCV and HCV-RNA. **Results:** Of the 21,750 persons aged ≥ 6 years sampled in NHANES, 16,847 (77.5%) were interviewed and 16,218 (96.3% of those interviewed) were examined. Serum samples were available for anti-HCV testing for 14,803 (91.3%) of the persons examined; 142 were anti-HCV positive and RNA positive, 54 were anti-HCV positive but RNA negative, and 14,558 were anti-HCV negative. Compared to HCV-uninfected adults (33.7 %; 95% CI=32.6-34.8), those with HCV infection (43.4%; 95% CI=34.0-53.2) were more likely to be overweight, but less likely to be obese (34.1% vs. 18.8%; p<0.01). Infected persons also were less likely than those who were not infected to report attempting weight loss (crude odds ratio=0.4; 95% CI 0.3-0.7), although HCV infection was not significantly associated with weight loss in our multivariable analysis, controlling for BMI, sociodemographics and other factors. **Conclusions:** Although being overweight can exacerbate complications for persons with HCV infection, we found that infected persons were not more likely than uninfected persons to attempt weight loss. Improved efforts to support weight control among adults with HCV infection are warranted.

**Board 162. Malnutrition, Socioeconomic Position, and Mortality among Children under 5 Years Seeking Care for Infectious Disease Syndromes at a Rural Hospital in Western Kenya, 2007–2011**

**D.C. Burton**[1,2], G.M. Bigogo[1,2], A. Audi[1,2], J. Williamson[1], R.K. Wadhera[3], A. Nyaguara[1,2], B. Aura[1,2], D.R. Feikin[1,2], R.F. Breiman[1,2];

[1]Centers for Disease Control and Prevention-Kenya, Nairobi and Kisumu, Kenya, [2]Kenya Medical Research Institute/CDC Research and Public Health Collaboration, Kisumu and Nairobi, Kenya, [3]Brigham and Women's Hospital, Harvard Medical School, Boston, MA, USA.

**Background**: Relationships between socioeconomic position (SEP), nutritional status and infectious disease mortality among children in rural Kenya are not fully described. **Methods**: Children under 5 years enrolled in a longitudinal study of infectious disease morbidity from May 2007 - April 2011 were provided free care for all infectious disease syndromes at a private hospital in rural western Kenya. Height and weight were measured at each sick visit and compared to a standard reference population. Stunting (height-for-age z-score < -2), underweight (weight-for-age z-score < -2), and wasting (weight-for-height z-score < -2) were each defined as moderate if the respective z-score was ≥ -3, and severe if the z-score was < -3. SEP was derived from responses to a local demographic survey as a weighted average of assets using multiple correspondence analysis, which divided respondent households into wealth index quintiles. Log-binomial regression and Cox proportional hazards models were used to examine predictors of nutritional status and mortality, respectively, accounting for household clustering. **Results**: Of 21,536 sick visits, moderate and severe stunting were observed in 18.9% and 13.8%, respectively; moderate and severe underweight in 11.5% and 5.7%, respectively; and moderate and severe wasting in 5.6% and 3.8%, respectively. Controlling for age and gender, children in the lowest SEP quintile had increased risk of stunting (adjusted risk ratio [ARR] 1.45, 95% confidence interval [CI] 1.26-1.66) and underweight (ARR 1.72, 95% CI 1.41-2.09) (p-values <0.001), but not of wasting, compared to children in the highest quintile. Children in the lowest SEP quintile also had higher mortality than those

in the highest quintile (smallest hazard ratio [HR] 2.47, 95% CI 1.36-4.48; p < 0.01), controlling for age, gender and nutritional status. Stunting, underweight and wasting were associated with approximately 2- to 8-fold increased mortality (all p-values <0.001), controlling for age, gender and SEP. **Conclusions**: In rural Kenya, data on SEP may be used to target nutrition interventions to high-risk children, and anthropometric data may be used at health facilities to identify children at increased risk of death who may warrant more intensive therapy and follow-up.

## Vulnerable Populations

Tuesday, March 13
12:30 PM – 1:30 PM
Grand Hall

**Board 163. Rift Valley Fever Virus Seropositivity in Pediatric Patients: A Study of Behavioral and Environmental Risks**

**A. LaBeaud**[1], A. Singh[1], S. Muiruri[2], L.J. Sutherland[3], E.M. Muchiri[2], G. Gildengorin[1], C.H. King[3];
[1]Children's Hospital Oakland Research Institute, Oakland, CA, USA, [2]Ministry of Health, Nairobi, Kenya, [3]Case Western Reserve University, Cleveland, OH, USA.

**Background:** In recent years there have been two major Rift Valley fever virus (RVFV) outbreaks in Northeastern Kenya. Children as well as adults are at risk for RVFV infection during and in between outbreaks. Little data has focused on the risk factors for children and the differences in these factors between adults and children in endemic areas. The objective of this study is to compare these risk factors, which include non-animal and animal exposures, from data derived from two prior RVFV village surveys in Northeastern Kenya. **Methods:** Surveys were conducted before (2006) and after (2009) the last major RVF outbreak in this region in 2006-2007. Participants underwent questionnaire administration, physical exam, vision testing, and blood collection for RVFV testing.
Results: In 2006, 247 participants (117 children) were enrolled and in 2009, 191 participants (44 children) were enrolled. Forty children in 2009 were repeats from the 2006 survey, none of which seroconverted. Childhood RVF seroprevalence rates were not statistically different between the two years (2006: 3.4% vs. 2009: 6.8%; p =0.39). Children were less likely to be seropositive than adults (p= <0.001). In general, children had less reported mosquito and animal exposures than adults. Children were less likely than adults to report: cooking raw meat (p<0.001), skinning animals (p<0.001), disposing of an aborted animal fetus (p=0.017) and consuming raw milk (p=0.022). However, children were more likely to report milking animals (p=0.034) and sheltering livestock (p=0.002). Comparisons between seropositive and sero-negative children revealed that those who were seropositive were more likely to have had camel contact (p=0.006) and consume raw milk (p= 0.022). Children from households with RVFV exposed adults were not more likely to be RVFV seropositive compared to those from households without exposed adults. **Conclusions:** Our results show that children have different animal exposures than their adult counterparts, which may put them at varied risk for RVFV infection. Raw milk consumption may be an important risk factor for RVFV exposure in pediatric populations.

**Board 164. Invasive Mold Disease (IMD) in Immunocompromissed (IC) Non-HIV Patients: Preliminary Data from the First Argentinean Registry for Invasive Mycosis (REMIIN)**

**M.C. Dignani**[1], T. Chiller[2], G. Davel[3], S. Rossi[4], A. Valledor[5], G. Guerrini[6], M.L. Pereyra[7], I. Roccia Rossi[8], R. Jordán[9], F. Herrera[10], A. Laborde[11], A. Vila[12], H. Peretti[13], A. Zárate[14], A. Bajalee[2], A. Hevia[3], N. Refojo[3], REMIIN (Registro Micosis Invasoras);
[1]REMIIN (Registro de Micosis Invasoras), Buenos Aires, Argentina, [2]Centers for Disease Control and

Prevention, Atlanta, GA, USA, [3]ANLIS, Buenos Aires, Argentina, [4]CDR, Buenos Aires, Argentina, [5]Htal. Italiano, Buenos Aires, Argentina, [6]Htal. Rossi, La Plata, Argentina, [7]Htal. Austral, Pilar, Argentina, [8]Htal. San Martín, La Plata, Argentina, [9]Htal. Británico, Buenos Aires, Argentina, [10]CEMIC, Buenos Aires, Argentina, [11]Fundaleu, Buenos Aires, Argentina, [12]Htal. Italiano, Mendoza, Argentina, [13]Sanatorio Británico, Rosario, Argentina, [14]Htal. Privado, Cordoba, Argentina.
**Background:** The epidemiology of IMD in IC patients varies in different institutions and countries, mainly due to: 1) the patient history of antifungal treatments (AFT), 2) geography, and, 3) institutional reservoirs. A multicenter national registry for IMD (REMIIN) was created to understand the Argentinean epidemiology of IMD. Preliminary results are presented. **Methods:** The criteria to be enrolled in the REMIIN were: 1) non-HIV immunesuppression, 2) probable or proven IMD (EORTC/MSG), 3) signed informed consent, and 4) recent IMD (less than 3 months after the end of the AFT). **Results:** From 3/2010 to 5/2011, 27 patients from 10 centers were enrolled. Median age was 51 (6-70) years old and 63% were male. Underlying diseases included hematological malignancies (23), renal transplantation (2), steroid treatment (1) and chronic granulomatous disease (1). Most IMDs were probable (15) and localized (22). The organisms causing IMDs were *Aspergillus* spp. (18), *Fusarium solani* (5), Zygomycetes (3) and Hyphae (1), *Paecilomyces lilacinus* (1), *Phialophora* spp. (1), *Phialemonium curvatum*, and *Trichoderma* spp. (1). Aspergillosis was diagnosed by culture (13) or by GM (5). The species distribution of Aspergillus included: *A. fumigatus* (6), *A. flavus* (2), *A. terreus* (2), *A. sydowii* (2), and *A. niger* (1). The Zygomycetes were identified by culture (2) or by pathology (1). They included: *Rhizopus oryzae* (1), and *Syncephalastrum racemosus* (1). Mixed IMDs were found in 3 patients. One included 3 molds (*Phialemonium curvatum* + *Syncephalastrum racemosus* + *A sydowi*), one included 2 molds (*F. solani* + *A. sydowii*) and one included 1 mold (*F. solani*) + positive GM. Initial AFTs were Voriconazole (10), Liposomal amphotericin B (9), deoxicolate Amphotericin B (5), Posaconazole (1), Itraconazole (1) and Liposomal Amphotericin B + Voriconazole (1). Survival was 85% on day 30 of AFT and 63% at a median follow up of 92 days (12 - 914). **Conclusions:** Preliminary data from the first multicenter registry of IMD in IC patients in Argentina showed that the most common etiological agents were *Aspergillus* spp. followed by *Fusarium* spp. and Zygomycetes. These results are comparable to those seen in the northern hemisphere.

**Board 165. Retrospective Analysis of Demographics and Clinical Factors Associated with Febrile Respiratory Illness among US Military Basic Trainees**

**D.S. Padin**, S. Putnam, A. Hawksworth, D.J. Faix, P.J. Blair;
Naval Health Research Center, San Diego, CA, USA.
**Background:** Basic trainees in the U.S. military have historically been vulnerable to respiratory infection. Crowded living conditions and stressful work environments make these populations prone to outbreaks of febrile respiratory illness (FRI). Adenovirus and influenza are the most common etiological agents responsible for FRI among trainees and present with similar clinical signs and symptoms. Identifying clinical factors to differentiate between adenovirus and influenza will help improve diagnosis, allow for appropriate use of therapeutics and improve troop readiness. **Methods**: Specimens were systematically collected from trainees meeting FRI case definition (fever ≥ 100.5 F with either cough or sore throat; or provider diagnosed pneumonia) at eight basic training centers. PCR and / or cell culture testing for respiratory pathogens were performed on specimens. Interviewer-administered questionnaires collected information on patient demographics and clinical factors. Polychotomous logistic regression was employed to assess the association between these factors and FRI outcome categories - laboratory confirmed adenovirus, influenza, or other FRI. Sensitivity (SN), specificity (SP), positive and negative predictive values (PPV, NPV) were calculated for individual predictors and clinical combinations of predictors. **Results**: Among 18,439 FRI cases sampled between 2004 and 2009, 62.8% were laboratory confirmed adenovirus cases and 7.65% were laboratory confirmed influenza cases. Subjects were

predominantly young males (86.8% males; mean years 20.8 ± 3.8) from Fort Jackson (18.8%), Great Lakes (17.5%), Fort Leonard Wood (15.6%) and MCRD San Diego (15.1%) and Fort Benning (13.5%). The best multivariate predictors of adenovirus were sore throat (3.03 [2.71-3.40]), cough (2.32 [2.07-2.59]), fever (2.22 [1.99-2.47]) and training weeks 3+ (2.17 [1.96-2.42]) with a positive predictive value (PPV) of 78% ($p \leq .05$). A combination of cough, training weeks 0-2, fever, and acute onset were most predictive of influenza (PPV = 40%; $p \leq .05$). **Conclusions**: Specific demographic and clinical factors were associated with laboratory confirmed adenovirus and influenza among trainees. Findings from this study can guide clinicians in the diagnosis and treatment of military trainees presenting with FRI.

**Board 166. Vaccination among Adults Hospitalized with Influenza in New Haven County, Connecticut during the 2005-06 thru 2009-10 Influenza Seasons**

**K. Tam**, K. Yousey-Hindes, J. Hadler;
Connecticut Emerging Infections Program, New Haven, CT, USA.

**Background**: Influenza causes substantial morbidity and mortality among high-risk adults. To reduce the incidence of influenza and its complications, recommendations for annual vaccination have expanded to include all adults. Assuming that relative vaccination rates by medical risk factors for complications among hospitalized adults are similar to those in the general population, we used data from the Connecticut Emerging Infections Program's (EIP) population-based surveillance system for influenza-related hospitalizations to examine influenza vaccination rates among adults hospitalized for influenza-related illnesses in New Haven County (NHC), Connecticut during the 2005-06 thru 2009-10 influenza seasons. **Methods**: Hospitalized laboratory-confirmed influenza cases residing in NHC were identified through the EIP surveillance system. Demographic data, medical conditions, and vaccination history were obtained through medical chart reviews and interviews with physicians and patients. Bivariate analyses were performed to compare vaccination rates among cases with medical conditions to those without; p-values <0.05 were considered statistically significant. **Results:** A total of 1,136 adults were hospitalized with influenza in NHC during the five influenza seasons, of which 1,026 (90.3%) had underlying medical conditions. Cases with medical conditions were significantly more likely to be vaccinated than those without medical conditions (56.5% vs. 29.7%). The highest vaccination rates were among those with chronic lung disease (66.7%), cardiovascular disease (63.2%), chronic metabolic disease (59.8%), and asthma (49.1%), all of which were significantly higher than among those without any medical condition. The lowest vaccination rate was among pregnant women (25.9%), not statistically different than in women with no medical conditions (30.8%). **Conclusions:** Our analysis shows that the majority of high-risk adults, when compared to those not at high-risk, are being vaccinated for influenza. However, additional efforts are needed to improve vaccination in pregnant women. Implementation of universal adult vaccinations should help to reduce the risk of exposure of those at high risk and the overall burden of influenza on the health care system.

## Novel Agents of Public Health Importance

Tuesday, March 13
12:30 PM – 1:30 PM
Grand Hall

**Board 167. Control of Avian Influenza A (H5N1) Virus in Live Bird Markets in Low Resource Settings**

**G. Samaan**[1,2], F. Hendrawati[3], T. Taylor[4], K. Lokuge[2];
[1]Centres for Disease Control and Prevention, Jakarta, Indonesia, [2]Australian National University,

Canberra, Australia, [3]Disease Investigation Center, Maros, Indonesia, [4]Australian Animal Health Laboratory, Geelong, Australia.
**Background:** Live bird markets are known to amplify, circulate and propagate avian influenza viruses, including the H5N1-virus which poses pandemic threat. This study assesses the feasibility and impact of implementing the WHO guideline for control of avian influenza (AI) H5N1 virus in markets in a low resource setting. **Methods:** The WHO guideline was applied in two live bird markets in Indonesia and was operationalized using a participatory approach. Feasibility was assessed through observation and stakeholder surveys. Impact was directly assessed by comparing AI H5N1 virus contamination pre- and post-intervention. Impact was also indirectly assessed through comparison of contamination levels with the hygiene indicator pathogen *Escherichia coli* (*E. coli*). **Results:** Both infrastructure and behavior change interventions were introduced in the two markets. Most market stakeholders were satisfied with the interventions. Since there was no AI H5N1 virus circulating during most of the study period, it was not possible to directly assess impact. However, the indirect measure of impact showed a significant reduction in *E. Coli* levels. This improvement in hygiene, coupled with the introduction of measures known to reduce AI H5N1 contamination such as zoning, daily waste removal and cleaning, strongly suggest that the risk of H5N1-contamination was reduced in the two markets. **Conclusions:** This is the first study to report the feasibility and impact of the WHO guideline. The study supports the view that contamination and transmission of AI H5N1 virus in markets can be controlled through the infrastructure and behavior change interventions recommended in the WHO guideline.

**Board 168. Serological Evidence of Human Infection with Porcine Reproductive and Respiratory Syndrome Virus**

**K.R. Atanasova**[1], R.A. Hesse[2], G.C. Gray[1];
[1]University of Florida, Gainesville, FL, USA, [2]Kansas State University, Manhattan, KS, USA.
**Background:** Porcine reproductive and respiratory syndrome virus (PRRSV) is economically one of the most important pathogens for the swine industry and one of the major components of the multi-factorial porcine respiratory disease complex. It is an enveloped positive-sense single-strand RNA virus classified in the order *Nidovirales*, family *Arteriviridae.* PRRSV, enzootic in the United States, can cause a wide range of symptoms in swine of different ages (e.g. abortions and reproductive failure in sows, respiratory symptoms in piglets), as well as have an asymptomatic course or be masked by concurrent infections with other pathogens. There have been only a few attempts to investigate the ability of humans to serve as mechanical vectors of PRRSV, and to our knowledge there are no published studies investigating the ability of PRRSV to cause an immune response in humans. The goal of this study is to investigate whether swine workers and people exposed to swine may develop an antibody response to PRRSV. **Methods:** We examined sera collected in the period 2004-2006 from asymptomatic healthy swine workers from Iowa, USA. Currently 201 of the sera, collected in 2006, were tested in a swine immunoperoxidase monolayer assay (IPMA) that was adapted for screening of human sera. Sera from PRRSV hyper-immunized and PRRSV sero-negative pigs were used as positive and negative control respectively. **Results:** Two of the 201 tested human sera demonstrated strong reactivity in the assay (antibody titers ≥ 1:512). The staining pattern resembled closely that seen in the positive control serum. The test was repeated three times giving similar results. **Conclusions:** As far as the authors know this is the first time that human serum has been shown to have elevated antibodies against an *Arterivirus*. These data suggest that PRRSV can infect humans, although the virus has not been associated with human disease. While it is possible that the sero-reactivity in this study may be evidence of cross-reactivity from other viral infections, these data suggest that further studies of possible human infection with PRRSV are indicated. Assays of additional human sera will be presented.

**Board 169. Public Health Investigation of a Novel *Ehrlichia ewingii* Case Acquired from Leukoreduced Platelet Transfusion**

**J. Matthias**[1], D. Stanek[1], M. Bertholf[2], J. Regan[3], J.P. Whittle[4];
[1]Florida Department of Health, Tallahassee, FL, USA, [2]The Blood Alliance, Inc., Jacksonville, FL, USA, [3]Centers for Disease Control and Prevention, Atlanta, GA, USA, [4]Mercer University School of Medicine, Memorial University Medical Center, Savannah, GA, USA.

**Background:** A patient with acute lymphoblastic leukemia presented to a Georgia hospital with acute fever and malaise (7/19) and eventually neutropenia (7/29) following 3 transfusions during treatment for his cancer. The hospital lab identified suspect *Ehrlichia* morulae on 7/30 using microscopy; reverse transcription polymerase chain reaction (RT-PCR) performed on 8/2 was positive for *Ehrlichia* sp. Additional PCR testing at CDC confirmed the pathogen to be *Ehrlichia ewingii.* The patient had no known tick exposure. **Methods:** The Florida blood bank that provided the transfusion products performed a trace-back on the 3 donors and obtained whole blood and serum from each. Tracebacks of other recipients receiving blood products collected from the positive donor between 5/12 and 7/27 were performed to assess if any additional recipients had symptoms relating to *Ehrlichia ewingii.* **Results:** Transfusion products received included leukoreduced single donor platelets on 6/22, irradiated and leukoreduced red blood cells on 6/22 and leukoreduced single donor platelets on 6/27. Follow-up of *Ehrlichia* serology was positive for one donor, who had a titer of 1:512. This donor had provided leukoreduced apheresed platelets for the patient, and additionally regularly donated platelets and plasma 1 or 2 times per month. Over the course of the investigation the donor was found to have known tick exposures at his home in Florida and at a wooded property in South Carolina. He reported no febrile illnesses in the 2 months prior and following the suspect donation. Routine complete blood counts (CBC) performed by the blood bank at the time of each donation were within normal limits. Five recipients received leukoreduced platelets and 3 recipients received plasma from the donor. Three of the recipients died within 1-2 days of transfusion due to unrelated causes. The remaining 5 recipients reported no symptoms of illness associated with *E. ewingii*. *Ehrlichia* serology tests are being conducted on serum samples from these recipients. **Conclusions:** This is the first known transmission of any *Ehrlichia* species by means of blood transfusion. Leukoreduction of platelets is designed to reduce the risk of transmission of infections transmitted by intracellular white blood cell pathogens, but this case demonstrates risk is not eliminated.

## Influenza Preparedness: Lessons Learned

Tuesday, March 13
12:30 PM – 1:30 PM
Grand Hall

**Board 170. Pandemic H1N1 Influenza in Bhutan**

**R.V. Gibbons**[1], B. Thapa[2], S. Zangmo[2], R.G. Jarman[1], P. Bhoomiboonchoo[1], S. Wangchuk[2];
[1]Armed Forces Research Institute of Medical Sciences, Bangkok, Thailand, [2]Public Health Laboratory, Ministry of Health, Thimphu, Bhutan.

**Background:** The Himalayan Kingdom of Bhutan remains relatively isolated; there are no trains, only three major roads cross its national borders (from India), and there are only one to two flights a day at the lone airport. Over the past 50 years the Department of Virology at the Armed Forces Research Institute of Medical Sciences in Bangkok has assisted with determining the etiologies of many outbreaks of febrile and respiratory illnesses in Asia. **Methods:** ILI cases were defined as a person with fever (≥ 38C) and cough or sore throat. **Results:** The first cases of pandemic 2009 influenza virus (pH1N1)

infection in Bhutan were reported in July 2009, 3 months after the virus was first reported in Mexico in April 2009. The sampling of the cases actually occurred in June. The month delay was due to the lack of testing capability in Bhutan and the need to ship samples to Bangkok. During the official WHO pandemic period (11June 2009 to 8 August 2010), a total of 2264 samples were collected and tested by RT-PCR of which 20.7% (467) were confirmed pH1N1. H3N2, H1N1, and B were positive in 1.9%, 1.2%, and 6.9% of samples, respectively. The virus was first identified in Thimpu (the capital located 60km from the airport) and Paro (the city with the airport) and later in Gelephu (a border city with a major road into India). The highest rate (29.0%) of pH1N1 cases was detected in the 6-20 year-old age group; the lowest rate (0.7%) was in those over 50 years old. The mean age of those with pH1N1 was 19.5 years, trending to be younger than those with H1N1 (23.1 years), significantly older than those with H3H2 (16.9 years) and B (15.4 years). Importantly, Bhutan accelerated its timetable for opening influenza surveillance sites, increasing from three sentinel sites in April 2009 to eleven a year later. In April 2010 the National Public Health Laboratory established PCR capability for influenza. **Conclusions:** Despite relative isolation, Bhutan was part of the pandemic within 2 months of pH1N1 reports. The capability established at the sentinel sites and the laboratory will be used to continue influenza surveillance with plans to add acute encephalitis and fever surveillance. The pH1N1 pandemic has made Bhutan more prepared for epidemics in the future.

**Board 171. Pandemic Influenza A (H1N1)—2009 (India): Action Taken, Experiences, and Lessons Learnt**

**A. Choudhry**, S. Khare, L. Chauhan;
National Centre for Disease Control, New Delhi, India.

**Background:** Currently, all 6 regions of WHO are showing moderate to low level of activity regarding influenza A (H1N1). Very low activity of influenza is being reported from 31 states and UTs of the country. A total of 48337 lab confirmed cases 2795 deaths have been reported from 31 states and UTs of India since the emergence of pandemic H1N1 influenza in the country. National Centre for Disease Control (NCDC) was identified as the nodal agency for collection, transportation, testing and reporting of samples received from various parts of the country. A total of 8027 lab confirmed cases have been reported from May, 2009 to September 2011. **Methods:** Samples of all suspected cases from various identified institutions and hospitals were tested at NCDC in the Microbiology Division through RT-PCR technique. **Results:** Since the start of the pandemic in 2009 the number of lab confirmed cases has shown a downward trend with 25912 cases and 956 deaths (CFR 3.6%) in 2009, 20219 cases and 1772 (CFR 8.7%) in 2010 and 2206 cases and 67 deaths (CFR 3.03%) in 2011. Average % positivity of 11 functional network labs in 2009 of 0-29%,2010 0-32% and in 2011 0-9%. At NCDC for H1N1 positivity in 2009 was 25.3% in 2009, in 2010 16.4% and in 2011 8.6 % showing a decreasing trend .Positivity for other types of influenza viruses shows 11.4% positive for seasonal influenza A in 2009 5.8% in 2010 and 8.4% in 2011. influenza B showed no activity in 2009, in 2010, 0.4 % positive and in 2011 3.55% positive. Influenza A H3 0.09% positive in 2009, 8.6% in 2010 and 0.20% positive in 2011. The major age group affected was group 20-39 in all 3 years . Males were affected more than females. Month wise data to know the trend of H1N1 positive cases showed single peak was observed in September2009, peak was observed in the month of august and September2010 and two peaks one in the month of March and second in the month of May 2011 . Influenza A(Seasonal flu) showed single peak in August 2009 ,in two peaks in August, September 2010 ,two peaks in 2011 March and May . No peak For Influenza B in 2009, some activity observed during the month of January 2010 and 3 peaks were observed in 2011 in February, May and August 2011. **Conclusions:** The 2009 pandemic H1N1 has given a great opportunity to initiate country-wide influenza surveillance as no exact data regarding burden and trend of influenza for India is not known.

**Board 172. Enhancing Pandemic Preparedness and Response Capacity in the Republic of Moldova**
**R. Cojocaru**, A. Ursu, I. Bahnarel, S. Gheorghita, C. Spinu, V. Dubalari, I. Spinu;
National Centre for Public Health, Chisinau, Republic of Moldova.
**Background:** The Avian Influenza Control and Human Pandemic Preparedness and Response (AIHP) project financed by World Bank started in the Republic of Moldova in 2006. The project was a part of the Global Program for Avian Influenza Control and Human Pandemic Preparedness and Response. Within the project the following activities were implemented: laboratory staff was trained in influenza surveillance methods, infection control, clinical management of patients, pandemic response, sample collection, biosafety measures, and virus isolation; an intensive care unit was built and equipped; the National Viral Laboratory (NVL) for seasonal influenza was renovated for BSL-2 and BSL-2+ capabilities. The laboratory was equipped for influenza testing. **Methods:** The cooperative agreement Enhancing Pandemic Preparedness and Response Capacity in the Republic of Moldova started in August 2009. FY 2011 was the third year of funding through the agreement. **Results:** Cooperative agreement activities were implemented by the national counterparts from the Ministry of Health (MoH) in close collaboration with WHO Regional and Country Offices, and other international partners.
The Republic of Moldova implemented regular hospital-based surveillance in nine sentinel sites, where data was collected on ARI, ILI, and SARI. The National counterparts are in the process of updating the documents for implementing the integration of laboratory-based surveillance in accordance with CDC recommendations provided within the monitoring visit that took place in May, 2010. The MoH started laboratory-based surveillance in five sites within the CDC cooperative agreement in 2011. The NVL publishes regular surveillance reports regarding the epidemiological and laboratory data and reports weekly information through WHO EuroFlu network. The WHO and CDC's experts assessed the laboratory capacity of the NVL. They provided some recommendations and assistance to the national counterparts in their efforts to become a National Influenza Centre (NIC) recognized by WHO. The NVL uses PCR and virological assay diagnostic techniques. The new influenza pandemic revealed challenges and gaps in the National Pandemic Preparedness Plan (NPPP) in 2009. The intersectoral interaction, coordination and communication proved to be weaker than expected, and public and private sector organizations and essential services providers were not sufficiently engaged in pandemic preparedness and response. A NPPP working group updated the plan to ensure a high-level political commitment and involvement by the whole of society. The NVL participated in the WHO EQAP for the detection of influenza virus subtypes A and B. Clinical samples were confirmed by the WHO CC London. The WHO Shipment Funds Project covered the transportation cost. The MoH National Focal Point of International Health Regulations (2005) (IHR) has provided the notification of new influenza cases and deaths to WHO Focal Point. **Conclusions:** As a result of the collaborative activities between the Moldovan MoH and U.S. CDC the human and infrastructure capacities for pandemic preparedness, surveillance, monitoring, early response, communication, and infection control are in continuous improvement.

**Board 173. Sentinel Surveillance of Influenza and Acute Respiratory Infections in Taldykorgan, Almaty Oblast in the Last Three Epidemic Seasons**
**S.B. Kapsalamova**;
Taldykorgan State Sanitary and Epidemiological Surveillance Authority, Taldykorgan, Kazakhstan.
**Background:** Surveillance of influenza-like illnesses (ILI) and the influenza virus types that cause it are important to identify morbidity due to influenza and identify groups who should be targeted for vaccination. Sentinel surveillance of influenza was introduced in Taldykorgan, a city of about 166000, in 2008, with the principal goal of identifying circulating influenza virus types. **Methods:** Three sentinel sites were chosen. Samples were selected from patients >1 year of age who met a standard case definition. Samples were studied in the city virology laboratory by real-time PCR. **Results:** Eighty-four patients (66 of whom were children < 14 years of age) in the 2008-2009 epidemic season, 68 patients

(54 of whom were children < 14 years of age) in the 2009-2010 epidemic season, and 49 patients (36 of whom were children < 14 years of age) in 2010-2011 epidemic seasons were tested.
*2008-2009* **-** 41 (49%) of 84 patients had positive results. Influenza A was identified in 35 (85%) and influenza B in 6 (15%). Seasonal influenza A/H1 was the most common subtype (N=19; 55%), followed by A/H3 (N=11;31%) and influenza A unsubtyped (N=5;14%). Influenza A appeared at week 50 and remained throughout the season, while influenza B appeared in week 14. *2009-2010* **-** 15 (22%) of 68 patients had positive results and all were positive for influenza A (N=15;100%). Subtyping was not performed. *2010-2011* **-** 12 (24%) of 49 patients had positive results. Influenza A was found in 4 (33%) and influenza B (67%), with subtypes A: A/H3 - 3 (75%), influenza A/H1N1-09 -1 (25%). Influenza B circulated early in the season with influenza A following. **Conclusions:** We were successful in beginning influenza surveillance to determine circulating influenza virus. The 2008-2009 season began with circulating seasonal influenza A/H1N1 with influenza B co-circulating in the end of the season, while only influenza A circulated in 2009-2010. Conversely, the 2010-2011 epidemic season started with active circulation of influenza B with subsequent co-circulation of influenza A/H3 and A/H1N1-09. Why identification rates were twice as high in the initial season compared to the other two is uncertain. To determine magnitude of influenza as well as need for and success of vaccination, surveillance with determination of infecting virus is mandatory.

## Molecular Epidemiology

Tuesday, March 13
12:30 PM – 1:30 PM
Grand Hall

**Board 174. Whole Genome Sequencing Identifies Epidemiologically Informative Single Nucleotide Polymorphism Traits for the Subtyping of *Salmonella enterica* serovar Enteritidis**

**A. Kearney**[1], M. Taylor[2], E. Galanis[2], L. Hoang[3,4], M. Leslie[5], L. Chui[6,7], V. Allen[8], M. Gilmour[1,9];
[1]National Microbiology Laboratory, Public Health Agency of Canada, Winnipeg, MB, Canada, [2]British Columbia Centre for Disease Control, Vancouver, BC, Canada, [3]British Columbia Centre for Disease Control Public Health Reference Microbiology Laboratory, Provincial Health Services Authority, Vancouver, BC, Canada, [4]University of British Columbia, Department of Pathology and Laboratory Medicine, Vancouver, BC, Canada, [5]Animal Health Centre, British Columbia Ministry of Agriculture, Abbotsford, BC, Canada, [6]Alberta Provincial Laboratory for Public Health, Edmonton, AB, Canada, [7]Department of Laboratory Medicine and Pathology, University of Alberta, Edmonton, AB, Canada, [8]Public Health Laboratories, Public Health Ontario, Toronto, ON, Canada, [9]Department of Medical Microbiology and Infectious Diseases, University of Manitoba, Winnipeg, MB, Canada.
**Background:** In Canada, *Salmonella enterica* serovar Enteritidis accounts for approximately 40% of all clinical *Salmonella* isolates. *S*. Enteritidis is a highly clonal foodborne pathogen, and current laboratory methods including phage typing and Pulsed-field gel electrophoresis (PFGE) offer limited discrimination between isolates, making it difficult to differentiate outbreak events from concurrent sporadic isolates. **Methods:** Six human *S*. Enteritidis isolates, from British Columbia, representing the most common PFGE and Phage Type pattern combinations seen in Canada were selected for whole genome sequencing using the Roche GS FLX and Illumina platforms. Macrodiversity amongst isolates was identified using the Artemis Comparison tool. Single nucleotide polymorphisms (SNPs) were identified using MUMmer 3.0 and confirmed by PCR. A SNP typing method based on the 28 most informative SNP sites was run on 211 isolates (10 animal, 1 food and 62 human from British Columbia; 90 human from Ontario; and 20 animal, 1 food and 27 human from Alberta). Available epidemiologic information was used to assess the results

of the SNP typing assay. **Results:** The 6 chromosomes were collinear, and macrodiversity was due to the differential carriage of prophage and plasmids. The differences in prophage and plasmid content also accounted for PFGE pattern differences. A total of 803 SNP sites were identified amongst the sequenced isolates as compared to the reference strain P12509. The SNP typing method was able to distinguish between isolates with the same PFGE pattern and PT. Notably, isolates with epidemiologic linkages clustered together based on SNP typing. **Conclusions:** SNP typing substantiated the genetic lineages previously observed amongst *S*. Enteritidis based on PFGE and PT data and provided additional discrimination between isolates that are indistinguishable based on PFGE and PT data alone. The *S*. Enteritidis SNP assay has the potential to become a powerful tool to investigate the genetic relationship amongst *S*. Enteritidis isolate and to accurately identify distinct public health events.

**Board 175. Investigation of Clonal Structure of *Salmonella* ser. Enteritidis Isolates From Outbreaks by Pulsed-Field Gel Electrophoresis in the Russian Federation During 2010-2011**

**K.V. Kuleshov**, O.A. Fadina, S.S. Rozhnova, A.T. Podkolzin, G.A. Shipulin;
Federal Budget Institute of Science Central Research Institute for Epidemiology, Moscow, Russian Federation.

**Background:** *Salmonella* ser. Enteritidis is the most common cause of outbreaks of salmonellosis. The aim is to study the molecular genotype of *Salmonella* by pulsed-field gel electrophoresis (PFGE) in the Russian Federation from 2010 to 2011. **Methods:** We used PFGE of macro-restricted DNA using both XbaI and BlnI restriction enzymes to determine the molecular subtype of 181 *Salmonella* ser. Enteritidis isolates. Isolates were collected from 17 outbreaks which occurred in thirteen different regions (22 cities) of the Russian Federation during 2010-2011. The average number of isolates per outbreak was 10,4. **Results:** The isolates within each outbreak had the same XbaI and BlnI PFGE-patterns. Six patterns were seen among the 181 isolates using the primary restriction enzyme XbaI and 7 patterns by the secondary enzyme BlnI. Six clusters according to XbaI/BlnI pattern combinations were seen. The cluster JEGX01.0001/JEGA26.0001 which includes 142 isolates from 12 outbreaks (11 regions) was found to be predominating among 6 different PFGE clusters. Each of the other 5 outbreaks had the individual combination pattern. **Conclusions:** Our results confirmed the clonal nature of *S.* Enteritidis strains in the Russia Federation and the possible existence of major world-wide clone of *Salmonella* ser. Enteritidis. However, this requires further research in collaboration with other laboratories around the world.

**Board 176. Influenza Sub-typing Analysis of a Patient Population at Johns Hopkins Hospital during the 2010-2011 Influenza Season**

**L.A. Murillo**[1], K. Jeng[1], J. Hardick[1], A. Valsamakis[1], R. Sampath[2], L. Blyn[2], C.A. Gaydos[1];
[1]Johns Hopkins University, Baltimore, MD, USA, [2]IBIS/Abbott Molecular, Carlsbad, CA, USA.

**Background:** The 2009 H1N1 pandemic highlighted the need for accurate, rapid tests to subtype influenza isolates. A broad range assay shown to be adaptable to novel influenza specimens is the RT-PCR/electron spray ionization mass spectrometry (ESI-MS) Influenza Typing Assay (Abbott Molecular). We present the performance characteristics of RT-PCR/ESI-MS and analyze the genetic variation of influenza strains detected by spectrometry data in patients presenting to the Johns Hopkins Hospital, January-May, 2011. **Methods:** Nasopharyngeal (NP) samples were obtained from patients presenting to the Hospital with a suspected respiratory infection. A portion of each virology positive and selected virology negative samples was sent to the Maryland State Laboratory for confirmatory CDC RT-PCR. Before testing, a separate 300 ul aliquot of sample was provided for nucleic acid extraction and was analysed by the ESI-MS protocol using the PLEX-ID instrument. The assay included primer sets targeting 8 genes (PB1, NP, M1, PA, NS1, NS2, H and N). Positive controls were included (Zeptometrix, Inc). **Results:** A total of 77 NP samples were processed. According to reference testing (CDC RT-PCR ), 41 samples were identified as swine H1N1 (not designated as swine or seasonal), 24 as H3N2, 10 were

negative and 2 were of insufficient quantity to be tested. The RT-PCR/ESI-MS assay identified 32 samples as pandemic swine H1N1, 2 mixed pandemic H1N1 phenotypes, 5 seasonal H1N1, 24 H3N2, and 14 negative samples. The most prevalent pandemic H1N1 strain was Influenza A/New York/15/2009 pandemic-H1N1 (16) followed by Influenza A/California/05/2009 pandemic-H1N1 (7). Seven SNPS were detected in various previously identified strains. The most prevalent H3N2 strain was Influenza A/Thailand/CU-B1697/2009 (9) followed by Influenza A/ Kentucky/UR07-0148/2008 (7); eight were less prevalent strains. **Conclusions:** The RT-PCR/ESI-MS protocol is a useful tool for surveying influenza and discovering new genotypes. The advantage of RT-PCR/ESI-MS over the current gold standard RT-PCR protocol is that it provides rapid genomic characterization, which is important for monitoring the variability of viral strains in real time for rapid surveillance purposes.

**Board 177. High Risk HPV in Cervical Smears in the UAE**

**W.U. Tariq**;
Tawam Hospital in affiliation with Johns Hopkins Medicine, Al Ain,, United Arab Emirates.

**Background:** High risk human papillomavirus (HR HPV) genotpes (16, 18 and others) are important in the aetiology of human cervical carcinoma. Their screening, as an adjunct to cytology may help in follow up, early detection, intervention and post therapeutic considerations of affected patients. The prevalence of such genotypes varies in different social settings. In an attempt to delineate local scenario in the ladies of UAE, this preliminary data was analysed. **Methods:** The cytological findings were recordedfrom the reoutine Pap smears. The cases of AUSCUS and LSIL were further processed to extract the DNA and the real time PCR test was applied for High Risk HPV. **Results:** Altogether, 380 smears qualified for the test and suffiecient material for further test was available in case of 371 samples (AUSCUS 230 and LSIL 141). The high risk HPV was found in 112 (30%). In case of AUSCUS 40/230 smears (17%) and in case of LSIL 79 smears (56%) showed the presence of viral DNA of HR HPV). The viral geotypes were as follows: HPV-16:22/112 (19.6%),HPV-18:6/112(5.4%), Other HR HPV:68/112(60.7%), Other HR HPV and HPV-16:12/112 (10.7%), Other HR HPV and HPV-18:2/112 (1.8%) and Other HR HPV with HPV-16 and HPV-18:2/112 (1.8%). In other words altogether HPV-16 was seen in 36/112 (32%), HPV-18 in 10/112 (9%) and other HR HPV types in 84/112 (75%) cases. Therefore other high risk types were dominate in our population. **Conclusions:** To the best of our knowledge, this is the first clinically based study from this region which shows the presence of HR HPV, thus necessitating the need for prophylaxis, prevention and surveillance.

**Board 178. Molecular Typing of Isolates of *Bartonella bacilliformis* by Analysis of Intergenic**

**M.L. Tang**, J.J. Son, G.A. Dasch;
Centers for Disease Control and Prevention, Atlanta, GA, USA.

**Background:** *Bartonella bacilliformis* (Bb) is the causative agent of Carrion's disease. It manifests in 2 distinct clinical symptoms, either as an acute onset fever which progresses to a severe haemolytic anaemia (Oroyo fever), or as a chronic wart-like skin eruption (verruga peruana). The disease is common in the Andean regions of Peru but also occurs in Bolivia, Ecuador and Colombia. Hambuch et. al. 2004 found significant genetic variation among isolates by amplified fragment polymorphism analysis and Chaloner et. al. 2011 confirmed this through multilocus sequence typing (MLST). We have shown that Bb isolates vary in protein, antigen and pulsed-field gel electrophoresis patterns. The present work demonstrates that intergenic regions (IGR) contain both insertion/deletions (INDEL) and single nucleotide polymorphisms (SNP) that are useful genetic markers for Bb isolates. **Methods:** Primers in adjacent open reading frames (ORFs) were chosen to permit PCR amplification and sequencing of 128 of the 153 IGRs of 300-500 bp found in isolate KC583. Amplicons were visualized on 1.5% ethium bromide agarose gel. Amplified products showed obvious size differences in electrophoretic mobility were sequenced to confirm and characterize the differences observed. **Results:** 78 of the 128 primers

designed amplified KC583 DNA 300-500 bp IGR satisfactorily. They were then tested with 13 DNAs from isolates previously found to differ in IGR regions greater than 1000 bp but these are more laborious to sequence. Amplicons which differed in IGR size among the 13 DNAs were confirmed to have INDELs of the expected size and in some cases, additional SNPs. 11 of the 78 primer pairs did not amplify all of the IGRs, some due to SNPs at the primer sites and others possibly due to loss of ORFs. Primers for the more variable IGR were used to analyze DNAs from 60 Bb isolates. **Conclusions:** Analysis of small IGRs is a useful low technology method for evaluating the genetic profiles of different isolates of *B. bacilliformis* as it does not require accurate sequencing like MLST. While the small IGRs of Bb were less variable than the large IGRs examined previously, it was possible to identify the most divergent isolates of Bb which will be analyzed by whole genome sequencing to better understand its genetic diversity and the clinical diversity of Carrion's disease.

**Board 179. Multiple Strains of *Yersinia pestis* Associated with an Outbreak of Plague in Uganda**

**L.B. Respicio**, B.M. Yockey, M.E. Schriefer, J.M. Petersen;
Centers for Disease Control and Prevention, Fort Collins, CO, USA.

**Background:** *Yersinia pestis* is the etiological agent of plague, a highly pathogenic zoonotic disease most commonly transmitted to humans through exposure to fleas and their mammalian hosts. Plague is distributed worldwide, with the majority of human cases in recent decades being reported from Africa. Cases of human plague are most often sporadic, with outbreaks rare. In 2008-2009, an outbreak of plague occurred in the Arua and Nebbi districts in northwestern Uganda (an area of 10 km by 20 km), with >40 cases culture confirmed in a 4 month period. **Methods:** To analyze strain diversity in the context of this localized outbreak and compare with strain diversity observed in previous years, molecular typing was performed on isolates from the outbreak as well as isolates collected from the region in the 5 year period prior to the outbreak. Pulsed-field gel electrophoresis (PFGE) typing was performed with *Asc*I using the PulseNet protocol for *Y. pestis* and patterns analyzed using BioNumerics v. 5 (Applied Maths, Inc). **Results and conclusions:** Ten different PFGE patterns were observed among the 47 isolates from the outbreak with clustering of the 10 patterns into 6 PFGE groups. No temporal association was observed for strains falling with the same or different PFGE groups over the course of the outbreak. When cases were plotted based on PFGE group and GPS coordinates corresponding to the patient's residence, geographic separation was observed for several PFGE groups. Geographic overlap was also observed among the PFGE groups where strains with differing PFGE patterns were found within the same parish (4 km x 4 km). One of the PFGE patterns associated with the outbreak was observed in a strain previously isolated from the same region. Our results indicate the outbreak was associated with several strains, suggesting that multiple *Y. pestis* strains were likely present and circulating in the local animal and vector populations at the time of the outbreak.

## Zoonotic and Animal Diseases

Tuesday, March 13
12:30 PM – 1:30 PM
Grand Hall

**Board 180. Risk Factors for Cluster Outbreaks of Avian Influenza A H5N1 Infection, Indonesia**

**G. Samaan**[1,2], Directorate-General for Disease Control and Environmental Health, Ministry of Health, Indonesia, P.M. Kelly[3];
[1]Centers for Disease Control and Prevention, Jakarta, Indonesia, [2]Australian National University, Canberra, Australia, [3]ACT Health, Canberra, Australia.

**Background:** By July 30, 2009, Indonesia had reported 139 outbreaks of avian influenza (AI) H5N1 infection in humans. Risk factors for case clustering remain largely unknown. This study assesses risk factors for cluster outbreaks and for secondary case infection. **Methods:** The 113 sporadic and 26 cluster outbreaks were compared on household and individual level variables. Variables assessed include those never reported previously including household size and genealogical relationships between cases and their contacts. **Results:** Cluster outbreaks had larger households and more blood-related contacts especially first degree relatives compared to sporadic case outbreaks. Risk factors for cluster outbreaks were the number of first degree blood-relatives to the index case [Adjusted Odds Ratio (aOR) 1.50, 95% Confidence Interval (CI) 1.20-1.86] and index cases having direct exposure to sources of AI H5N1 virus (aOR 3.20, 95% CI 1.15-8.90). Risk factors for secondary case infection were being aged between five and 17 (aOR 8.32, 95% CI 1.72-40.25), or 18 and 30 (aOR 6.04, 95% CI 1.21-30.08), having direct exposure to sources of AI H5N1 virus (aOR 3.48, 95% CI 1.28-9.46), and being a first degree relative to an index case (aOR 11.0, 95% CI 1.43-84.66). Siblings to index cases were five times more likely to become secondary cases (OR 4.72, 95% CI 1.67-13.35). **Conclusions:** The type of exposure and the genealogical relationship between index cases and their contacts impacts the risk of clustering. The study adds evidence that AI H5N1 infection is influenced by, and may even depend upon, host genetic susceptibility.

**Board 181. Exploratory Research on Perceptions of Health Risk at the Human-Animal-Ecosystem Interface**

**A. Laing**, C. Prue, C. Rubin;
Centers for Disease Control and Prevention, Atlanta, GA, USA.

**Background:** Over 60% of existing human pathogens are zoonotic and approximately 75% of emerging pathogens can be traced back to animal origin. In the United States the co-existence of humans and non-human animals is demonstrated through everyday activities (e.g., prevalence of companion animals, popularity of outdoor activities). Therefore, it is important to understand the public's perception of health risks related to exposure to animals and the environment as well as the actions people take to protect themselves when conducting activities they may perceive as risky. The Centers for Disease Control and Prevention (CDC) embraces a "One Health" approach to addressing disease emergence at the interface of animal, humans, and the environment. This approach looks beyond humans at this ecologic interface by dramatically expanding the opportunities for disease detection, control, and prevention. It also leads to not only better human health but also animal health and environmental stewardship. Because this approach is new, CDC was interested in exploring public perceptions of these ideas. **Methods:** CDC is analyzing items which explored the public's understanding of disease transmission at the human-animal-ecosystem interface, perceptions of risk and risk acceptance, and motivations for protective behaviors from the Porter Novelli's *Healthstyles* survey. **Results:** The main objective is to better understand what people perceive as risky and the choices they make in order to protect themselves when faced with a perceived health risk at this interface. Data for this research is based on the results of a series of consumer mail panel surveys resulting in a sample of 2375 respondents. Data analysis is currently underway. **Conclusions:** Recognizing that everyday life activities lie on a continuum of perceived "risk avoidance" to perceived "risk acceptance" for each individual, understanding where certain activities fall on that continuum and what motivates people to protect themselves can lead to more informed public health messaging and interventions. Understanding health risk perceptions and protective behavior can improve public health messaging and interventions addressing infectious diseases that are likely to emerge from interactions between humans, animals, and the environment.

**Board 182. Pet Ownership and Animal Interaction in Canadian Households: Zoonotic Disease Implications for Public Health**

**J.W. Stull**, J.S. Weese;
Ontario Veterinary College, University of Guelph, Guelph, ON, Canada.

**Background:** Many human infections are transmitted through contact with animals (zoonoses). Data suggest pet ownership is increasing, with over 50% of homes in most countries having a pet. Despite the importance of household pets as a source of human infections, little is known of the public's pet ownership and contact practices. The objective of this study was to describe household knowledge and practices of pet ownership and animal contact for the general public in Ontario, Canada. **Methods:** A 10-minute questionnaire was developed. Individual and household-level data were gathered including demographics, knowledge of zoonotic diseases, disease information provided by medical staff and other sources, and pet ownership and animal contact practices. The questionnaire was self-administered to adults in the waiting areas of 2 multi-physician general practice offices. **Results:** 641 adults from different households completed the survey (75% response rate). Over 60% of participants had one or more household pets and 37% of non-pet owning households had frequent contact with animals. Pet ownership was high (45%) for households with one or more higher risk individuals (i.e., < 5 yrs, ≥ 65 yrs, diagnosed with an immunocompromising condition). High risk behaviours were frequently identified. Thirty-two percent of dog-owning households fed raw meat or egg food items. Most (>60%) dog and cat-owning households with children < 5 yrs reported the children never or only sometimes washed their hands after touching the animal. Approximately 30% of pet and non-pet owning respondents were not comfortable with their knowledge of diseases transmitted through pet contact or ways to reduce such diseases. Most respondents were unable to correctly identify zoonotic diseases from a list of common diseases. Most respondents (64%) had never received zoonotic disease information; when they had received this information, it was often self-acquired (e.g., internet). **Conclusions:** Pet ownership and animal contact were frequently observed. High risk practices were regularly reported and level of zoonotic disease knowledge was often low, indicating a need for improved education for both pet and non-owning households. Human and public health personnel should address these areas to ensure pet and non-pet owning households are targeted.

**Board 183. Sero-prevalence of H5N1 Antibodies among Poultry Workers in Bangladesh, 2008–2009**

**S. Nasreen**[1], S.U. Khan[1], E. Azziz-Baumgartner[2], K. Hancock[2], J.M. Katz[2], V. Veguilla[2], D. Wang[2], M. Rahman[3], A. Alamgir[3], E.S. Gurley[1], K. Sturm-Ramirez[1,2], T.M. Uyeki[2], S.P. Luby[1,2];
[1]International Centre for Diarrhoeal Disease Research, Bangladesh (ICDDR,B), Dhaka, Bangladesh, [2]Centers for Disease Control and Prevention, Atlanta, GA, USA, [3]Institute of Epidemiology, Disease Control and Research (IEDCR), Government of Bangladesh, Dhaka, Bangladesh.

**Background:** Bangladesh has experienced widespread poultry outbreaks of highly pathogenic avian influenza A (H5N1) virus since 2007. The risk of avian-to-human H5N1 virus transmission among poultry workers in Bangladesh is unknown. We conducted a cross-sectional study to determine the seroprevalence of H5N1 antibodies and risk factors for H5N1 virus infection among poultry workers during 2008-09. **Methods:** We identified 89 commercial poultry farms with confirmed H5N1 outbreaks from December 2007 to June 2009 and 3 live bird markets in Dhaka with poultry mortality exceeding 5-10% for 2 successive days during 2008. From January to July 2009, we enrolled workers at these sites and collected sera, demographic information, and history of poultry exposures. Microneutralization assay was performed to detect H5N1 neutralizing antibodies in sera using A/Bangladesh/207095/2008 (H5N1). A seropositive result was defined as an H5N1 neutralizing antibody titer ≥1:40, confirmed by horse red blood cell hemagglutination inhibition assay or H5-specific Western blot. **Results:** Among participants, 87% of farm workers (n = 212) and all market workers (n = 210) were male, with a median age of 30 years, range 18-59. Farm workers reported poultry exposure through feeding (97%), giving

water (83%), collecting eggs (83%), cleaning water trays (80%), cleaning feeding trays (76%), cleaning poultry stall/feces (61%), slaughtering (38%), defeathering (22%), evisceration (22%), poultry culling (48%), handling sick poultry during culling (94%) and contact with poultry that died from illness (91%). Market workers reported poultry contact through feeding (53%), cleaning water trays (51%), cleaning feeding trays (48%), slaughtering (39%), cleaning poultry cages/feces (38%), or evisceration (31%) and 85% reported contact with poultry that died from disease. No poultry workers were seropositive for H5N1 antibodies. **Conclusions:** Despite extensive poultry exposures, no serological evidence of H5N1 virus infection was found among farm or market poultry workers during 2008-09. However, the recent cases of human H5N1 infection detected in Bangladesh and the continued circulation of H5N1 among poultry warrants continued surveillance among a larger cohort of poultry workers to monitor the risk of avian-to-human H5N1 virus transmission.

**Board 184. Impact of Emergent Spotted Fever Group Rickettsiae in the Gulf Coast Tick, *Amblyomma maculatum***

**F.A. Girao**[1], J. Goddard[1], C. Paddock[2], A. Varela-Stokes[1];
[1]Mississippi State University, Mississippi State, MS, USA, [2]Centers for Disease Control and Prevention, Atlanta, GA, USA.

**Background:** *Amblyomma maculatum*, the Gulf Coast tick, transmits the emerging human pathogen, *Rickettsia parkeri,* causing a disease similar to, but milder than Rocky Mountain spotted fever. To date, over 20 cases of human infection have been reported with four of those reported occurring in Mississippi, where Rocky Mountain spotted fever also occurs. In addition, "*Candidatus* R. andeanae", a possible endosymbiont in *A. maculatum,* was recently reported occurring simpatrically with *R. parkeri*. Methods: We collected 707 adult Gulf Coast ticks in ten sites in Mississippi during summers of 2008 to 2010. Fragments of the tick mitochondrial 16S rRNA, and rickettsial outer-membrane protein A (*rompA*) gene, and species-specific *rompA* regions for *R. parkeri* and "*Candidatus* R. andeanae" were PCR amplified. **Results:** We amplified tick mtDNA in 98.7% of ticks collected. Of these, 128 ticks (18.4%) were positive for spotted fever group Rickettsiae and 105 ticks (15%) were positive for *R. parkeri rompA* singly. The endosymbiont was detected in 23 ticks (3.3%) of which 13 (1.9%) were co-infected with *R. parkeri.* **Conclusions:** This is the first report of co-infection with these two species. We are also evaluating population heterogeneity of *A. maculatum* and *R. parkeri* using Single Strand Conformation Polymorphism (SSCP). Thus far, we have identified 5 unique tick haplotypes, and confirmed by sequencing. We are currently processing SSCP for rickettsial DNA and anticipate correlating tick and rickettsial haplotypes to specific sites in Mississippi. Through our findings, we hope to increase awareness *R. parkeri* disease and better understand the ecology and epidemiology of *A. maculatum* and *R. parkeri* as well as *Candidatus* R. andeanae.

**Board 185. Detection and Characterization of Potential Zoonotic Pathogens in South African Bat Species**

**W. Markotter**[1], M. Geldenhuys[1], M. Tjale[1], S. McCulloch[1], J. Weyer[2], T. Kearney[3], L.H. Nel[1];
[1]University of Pretoria, Pretoria, South Africa, [2]National Institute for Communicable Diseases of the National Health Laboratory Service, Johannesburg, South Africa, [3]Ditsong National Museum of Natural History, Pretoria, South Africa.

**Background**: Over one hundred bat species, representing both the Pteropodiformes and the Vespertilioniformes orders occur in Southern Africa. In recent years a number of zoonotic emerging infectious diseases have been linked with bat species and it appears likely that more are to be discovered. **Methods:** In 2004 we initiated surveillance in bats with the initial objective to identify rabies-related lyssaviruses but this has since been expanded to include other pathogens. Sampling included submission of bats from conservation groups, museums or the general public as well as active

collection. Sensitive methods were developed to detect nucleic acids of lyssaviruses, coronaviruses, *Bartonella*, and *Rickettsia*. All positive results were further characterized by DNA sequencing and phylogenetic analysis. For rabies-related lyssavirus surveillance, serum samples were also collected and analyzed for the presence of neutralizing antibodies. **Results:** A nested RT-PCR targeting the coronavirus RNA dependent RNA polymerase gene indicated a ~4% prevalence (n=76) and phylogenetic analysis identified both alpha and beta coronaviruses. Real-time and conventional PCR targeting the gltA and 16SrDNA gene of *Rickettsia* indicated a ~3 % prevalence (n=322) and a high similarity to *Rickettsia conorri*. A conventional PCR targeting the gltA gene of *Bartonella* also indicated ~3% prevalence (n=322). Real-time RT-PCR targeting the lyssavirus nucleoprotein gene (n=700) identified Lagos bat virus RNA in only three bats but no other lyssaviruses. Antibodies that could neutralize Lagos bat virus were detected in *Epomophorus wahlbergi* (n=80) and for Duvenhage virus in *Miniopterus natalensis* (n=40). **Conclusions:** This is the first report of nucleic acid detection of coronaviruses, *Bartonella*, and *Rickettsia* in South African bats. Since *Epomophorus wahlbergi* is a known host for Lagos bat virus, corresponding neutralizing antibodies were expected. However the specific species involved in Duvenhage virus transmission is unknown and this was the first detection of corresponding neutralizing antibodies in *Miniopterus natalensis*. Globally, the detection, surveillance and epidemiological study of pathogens and potential pathogens remain paramount in progressive and responsible application of One Health principles.

**Board 186. Household Characteristics Associated with Rodent Presence and *Leptospira* Carriage in Three Community Settings in the Los Ríos Region of Chile**

**M.R. Mason**[1], M. Gonzalez[2], G. Acosta[2], C. Encina[2], C. Munoz-Zanzi[1,2];
[1]University of Minnesota, Minneapolis, MN, USA, [2]Institute of Preventive Veterinary Medicine, Austral University of Chile, Valdivia, Chile.

**Background:** Leptospirosis is a bacterial zoonotic disease of worldwide distribution caused by pathogenic *Leptospira*. Most mammalian species are natural carriers. Humans become infected through direct contact with fluids from infected animals or, more commonly, indirectly through contact with a *Leptospira*-contaminated environment. Global disease burden due to leptospirosis is unknown, but considered to be substantial. As major players in the transmission of *Leptospira*, studies have shown risk of human infection increases with proximity to rodents. Consequently, the purpose of this study was to examine household and community-level factors that may be associated with rodent presence, as well as with *Leptospira* carriage in rodents. **Methods:** A survey of 212 households in 6 communities was conducted in Southern Chile representing 3 types of communities: urban slums, rural villages, and farm areas. Traps were placed in the home and peri-domestic area and a questionnaire was administered at the household level. Rodents were assessed for *Leptospira* carriage using PCR. Mixed modeling, with random effects at household and community levels, determined factors associated with number of rodents trapped and their *Leptospira* carriage. **Results:** Households in slums were significantly younger and poorer than in rural villages and farm areas (p<.01). A greater proportion of households in rural villages (95%) and slums (99%) had potable tap water and solid waste removal (89% and 96%, respectively) than farm areas (32% potable water, 66% solid waste removal). Rodents were trapped in 106 households. More rodents were trapped per household (P<.01) in farm areas (3.4) than in rural villages (2.3) and slums (1.5). *Rattus rattus* was the dominant species in farm areas and rural villages while *Mus musculus* rodents were more frequently trapped in slums. Rural villages had the highest proportion of households with ≥1 PCR+ rodent (43.3%) and of all PCR+ rodents (21.4%). Six percent of rodents from farm areas were PCR+ and only 17.4% of households had ≥1 PCR+ rodent. Rodents from slums were negative. **Conclusions:** Identifying household and community-level factors associated with rodent presence and rodent *Leptospira* carriage allows for targeted interventions that intercept transmission and reduce human infection.

**Board 187. Prevalence of Brucellosis in Different Animal Species and Man in Sennar State**

**A.A. Taha**, E.O. Mansour;
Veterinary Research Institute, Khartoum, Sudan.

**Background:** The problem of brucellosis in Sennar State was considered recently, although its history was a long one. The objective of this work was to study prevalence of brucellosis in different animals species and man in Sennar State. **Methods:** Sample size of animal species was estimated presampling. A total of 2428 blood for serum samples was collected; 1547 from cattle, 585 from sheep, 169 from goats, 100 from equines and 28 from man; and a total of 24 vaginal swabs and 3 hygroma aspirates were collected from cattle sheep and goats. Serological and bacteriological examinations were carried out for examination of the samples collected. The tests used were Rose Bengal Plate test, modified Rose Bengal Plate test and Competitive ELISA were used for the examinations. Serum Agglutination test was used to measure antibody levels in serum samples of the positive animals. Suspected materials for Brucella infection used for culture, were hygroma aspirates and vaginal swabs. They were cultured on serum dextrose agar medium and incubated at 37°C under 10% carbon dioxide and examined daily for 10 days. Colonies resembling those of *Brucella* were used to prepare slide smears which were stained with modified Ziehl-Neelsen stain, and positives were further identified to genus, species and biovar levels using the standard methods. Serological testes showed the prevalence of brucellosis to be 23.7% in cattle, 4.10% in sheep, 4.73% in goats and 3.57% in man. The differences between the results of the tests used were marginal because the disease is endemic in the area. The highest prevalence of the disease was found in dairy cattle. Two strains of *B. abortus* biovar 6 and one strain of *B. abortus* biovar 1 were isolated from cattle. Different animal species in the state share the same pasture lands, and water resources and those of households share the same premises at night. Thus spread of brucellosis is most likely to occur among different animals, and from them to man, through direct and indirect contact with infected animals and animal products. **Conclusions:** The study recommends continuous surveys of brucellosis in the state to determine the magnitude of the problem and spread of the disease in cattle, sheep, goats, equines, camles and people in contact with infected animals and animal products. Immediate control measures of the disease through extension, to raise awareness of animal owners about its consequences and public health and economic impacts and to encourage them to participate in its control ,through vaccination to bring down the prevalence of the disease then followed by an eradication programme of the disease through test and slaughter.

## Vaccine-Preventable Diseases

Tuesday, March 13
12:30 PM – 1:30 PM
Grand Hall

**Board 188. Evaluation of School-Related Exposures as Potential Risk Factors for Pertussis Infection in Children Aged 7 to 12 Years in Minnesota, 2010**

**S.K. Kemble**[1,2], C. Kenyon[1], K. White[1], C. Miller[1], E. Zell[2], T. Skoff[2], K. Ehresmann[1], R. Lynfield[1];
[1]Minnesota Department of Health, St. Paul, MN, USA, [2]Centers for Disease Control and Prevention, Atlanta, GA, USA.

**Background:** During 2005-2010, pertussis incidence rose 30% among Minnesota preadolescents and reported outbreaks in elementary schools increased. Although studies are under way to evaluate the role of waning vaccine immunity, other risk factors for pertussis among this group have not been examined. **Methods:** We conducted a case-control study among children aged 7-12 years in Minnesota. By using statewide surveillance data, we recruited case patients meeting the Council of State and

Territorial Epidemiologists definition for probable or confirmed pertussis, with exposure periods during the 2010 school terms. Control subjects were identified through birth records autopopulated in the Minnesota immunization information system. We enrolled 3 control subjects/case patient with frequency matched according to age. Parents were interviewed regarding school-related and extracurricular activities and immunizations. Exposure odds ratios and 95% confidence intervals for case patients and control subjects were estimated using unconditional logistic regression. **Results:** We enrolled 214 case patients and 828 control subjects. Sixty-eight (32%) case patients and 207 (25%) control subjects (odds ratio [OR], 1.40; 95% confidence interval [CI], 1.01-1.94) participated in choir. When asked about vaccination practices, parents of 46 (21%) case patients and 125 (15%) control subjects (OR, 1.54; CI, 1.05-2.24) reported declining or postponing a vaccination for a child under their care; parents of 39 (18%) case patients and 75 (9%) control subjects (OR, 2.24; CI, 1.47-3.41) reported having children under their care insufficiently vaccinated according to routine schedules. No significant differences were found in classroom dynamics, school size, and total number of extracurricular activities. **Conclusions:** This investigation suggests choir participation is associated with contracting pertussis. When pertussis is diagnosed in children who participate in choir, targeted prophylaxis of contacts in this setting should be considered. Our results also highlight the continued need to counsel parents on the importance of vaccinations.

**Board 189. Influence of Economic Development and Accessibility to Immunization Services on Varicella Vaccine Uptake in Shandong Province, China**

**C. Wang**;
Centers for Disease Control and Prevention, Atlanta, GA, USA.

**Background:** Varicella vaccine has been available in the private sector in China for a decade, with one dose recommended for children. Varicella vaccination coverage has not been assessed previously. **Methods:** A cross-sectional study was conducted in Shandong province in May 2011 to examine varicella vaccination coverage in children aged 16-40 months. One county was randomly selected in each prefecture from the counties with above average 2008-2010 measles incidence. Five cities and three villages/communities per city were randomly selected in each county. A total of 10 children (half born in 2008 and half in 2009) were randomly enrolled from each village/community. Data on vaccination status came from immunization cards or records of the designated immunization clinics; prior varicella disease history was collected from parental recall. Each county was categorized as economically developed, sub-developed, or developing according to the gross domestic product per capita of the prefecture. The distance to the designated immunization clinic (<5 kilometers vs. ≥5 kilometers) and rural vs. non-rural residency were used as measures of access to immunization services. **Results:** The overall one-dose varicella vaccination coverage was 62% (1571/2532) with substantial variation by county (range: 16.7% - 94.7%). Children from more developed regions were more likely to be vaccinated (vaccination coverage= 77.8%, 61.0%, and 47.1% in developed, sub-developed, and developing regions, respectively, P<0.001).Rural residents were less likely to have received varicella vaccine (59.6% vs. 72.1%, P<0.001). Children living within five kilometers of an immunization clinic were more likely to have received varicella vaccine (62.6% vs. 59.3%, P=0.02). Children born in 2008 had higher varicella vaccination coverage than those born in 2009 (64.7% vs. 59.4%, P=0.005). **Conclusions:** One-dose varicella vaccination coverage is relatively high in China considering that it is currently only available in the private sector. Coverage levels will need to be increased further in order to ensure the elimination of severe varicella and varicella deaths. Further work is needed to understand the extent to which additional increases in coverage will require removal of financial barriers to varicella vaccination.

**Board 190. Assess the Effect of PMTCT HBV in Chaoyang District, Beijing, 2009–2010**

**J.-X. Ma, Sr.**[1], Q. Li, Jr.[1], W. Zhang, Sr.[2], M. Liu, Jr.[3], L. Pang, Jr.[3], X.-H. Pang, Jr.[2];
[1]Chaoyang Center for Disease Control and Prevention, Beijing, China, [2]Beijing Center for Disease Control and Prevention, Beijing, China, [3]Beijing Ditan Hospital, Beijing, China.

**Background:** Most perinatal HBV transmission from mother to child can be prevented by administration of HB vaccine and hepatitis B immunglobulin to the infant shortly after birth, although breakthrough infections occur. Assess the effect of the intervention on Prevention of Mother-to-Child Transmission would may lead to development of strategies to minimize transmission. **Methods:** All infants born to mothers identified by screening to be HBsAg+ in Chaoyang District, Beijing, China are referred to ditan hospital for their birth. Specimens from the mother and infant are obtained at the time of birth and frozen, and the children are administered HB vaccine (10ug Dalian Hansenula hepatitis B vaccine) and HBIG(200IU) during the first 6 h of life. The children are enrolled into a perinatal HBV prevention program and administered subsequent vaccine doses at 1 months and 6 months of age and tested for infection to HBV and development of seroprotection at 7-15 months of age (using Abbott reagents). All data are entered into a database. **Results:** We aim to analyze these data and conduct additional testing for HBeAg and HBV DNA to compare children who developed breakthrough infection to those who did not. Final 1014 infants born between January 2009 to December 2010 are followed, the carrier rate of these children are 2.56% at 7-15 months of age, positive rates of anti HBs are 96.4% and GMT are 400.40IU, respectively. Among 151 infants who received three dose vacines only, the carrier rate was 0.67%; compared with 2.89% among 889 infants who received HB vaccine and HBIG at birth. Further reduce of carrier rate of children are not observed among children who are administered HB vaccine in conjunction with HBIG, independent of mother's HbeAg and HBVDNA maternal status. **Conclusions:** Further reduction of carrier rate of children is not observed among children who are administered HB vaccine in conjunction with HBIG, independent of mother's HbeAg and HBVDNA maternal status.

**Board 191. Varicella Vaccine Effectiveness under Conditions of Intense Exposure in China**

**C. Wang**;
Centers for Disease Control and Prevention, Atlanta, GA, USA.

**Background:** Varicella vaccine has been available in China for purchase on a private basis since 1998, with a single dose recommended for children ≥12 months of age. However, there are few data on varicella vaccine effectiveness (VE) in China. **Methods:** A matched case control study was conducted in kindergartens and elementary schools of Tai'an prefecture, Shandong province, China, from 2010 to 2011. A varicella case was defined as an acute generalized maculopapulovesicular rash without other apparent cause in a student. Breakthrough varicella was defined as varicella > 42 days after vaccination. Two matched controls were randomly selected from among the classmates of the case. Vaccination status was collected from immunization records or parental recall if immunization records were not available. Information on prior disease history and clinical presentations came from a survey of students' parents. Varicella vaccination coverage in each kindergarten or elementary school was also collected during the investigation. **Results:** There were no differences in sex, age, and chronic disease between the 182 cases and the 360 matched controls with two cases were 1:1 matched due to insufficient controls in the classroom. Cases were much less likely to be vaccinated (10.4% vs. 36.7%, P<0.001). The single-dose varicella VE was 86.4% (95% confidence interval: 73.9%-93.0%, P<0.001), and the VE for prevention moderate-severe disease (≥50 lesions) was 92.5% (95% confidence interval: 76.6% to 97.6%, P<0.001). The 150 vaccine recipients (19 cases and 131 controls) received vaccines from five manufacturers. There was no difference in vaccine effectiveness by manufacturer for the two vaccine types for which there were an adequate number of recipients (Changsheng and Baike, 71% vs. 89%, P=0.13). Average vaccination coverage in the 44 schools was 38.8%, with substantial variation (0 to 94%) by school. Coverage was lower in elementary schools than in kindergartens (29.9% vs. 48.4%, P=0.03)

**Conclusions:** Single dose varicella vaccine is highly effective in preventing varicella, particularly moderate-severe disease, even under high intensity of exposure. Further study of the factors associated with vaccine uptake in China is needed in order to increase coverage and maximize the protective effect of varicella vaccination.

## Outbreak Investigation: Lab and Epi Response

Tuesday, March 13
12:30 PM – 1:30 PM
Grand Hall

### Board 192. Diagnostic Misstep Using a Biological Analyzer for Detection of Staphylococcal Enterotoxin B

**S.M. Tallent**, J.M. Hait, R.W. Bennett;
US FDA/CFSAN/ORS, College Park, MD, USA.

**Background:** School children and faculty members experienced symptoms of vomiting, nausea, abdominal cramps, and diarrhea after eating food prepared by the caterer contracted by Guam's Department of Education to service five public schools. The first illness was reported within an hour after serving breakfast affecting a total a 295 students and two faculty members. The illnesses were consistent with staphylococcal food poisoning (SFP) often implicated following ingestion of food that has been contaminated with Staphylococcus aureus capable of producing enterotoxins. **Methods:** The items offered for breakfast included egg salad sandwiches, milk, cantaloupe, and peaches. Samples of milk, cantaloupe and egg salad sandwiches were tested using an electrochemiluminescence (ECL) analyzer approved for use in environmental and food testing. Samples were also submitted to the state health department for standard microbiological and immunological analysis. All food samples were analyzed for pre-formed enterotoxins and bacterial growth using approved standard methods. Additionally, outbreak samples and isolates were tested with a lateral flow device (LFD) often used by support teams to test for SEB. **Results:** The ECL detected pre-formed Staphylococcal enterotoxin B (SEB) from the egg salad sandwiches; however, Staphylococcal enterotoxin D (SED) was detected by ELISA testing. Additionally, Staphylococcus aureus was isolated from the egg salad sandwiches in which all isolates tested positive for SED production. The LFD also gave false positive reactions using purified SED $\geq$10μg/mL, food samples and cell-free culture supernatants. **Conclusions:** While researchers have demonstrated that the ECL and LFD can detect low levels of SEB, no evidence was found that excluded cross-reactivity with other staphylococcal enterotoxins. The ECL and LFD are valuable tools used by public health officials, but caution must be exercised when reporting results on samples that are of public health concern. More studies are required with various food matrices and enterotoxins before such methods can be recommended for general use.

### Board 193. Optimal Number of Specimens to Test during Institutional Respiratory Infection Outbreaks, Ontario

**A. Marchand-Austin**[1], A. Peci[2], A.-L. Winter[3], J. Gheewala[2], N. Lombardi[2], J. Gubbay[2];
[1]Public Health Agency of Canada, Toronto, ON, Canada, [2]Public Health Ontario Laboratories, Toronto, ON, Canada, [3]Public Health Ontario, Toronto, ON, Canada.

**Background**: Laboratory testing is crucial in the identification of institutional respiratory infection outbreaks. Determining the number of specimens to be tested during a suspected outbreak is challenging as outbreak management needs and testing costs must be balanced. The objective of this study was to determine the optimal number of respiratory specimens to be tested by multiplex

respiratory viral PCR (MRVP) during an outbreak. **Methods**: We reviewed respiratory specimens from outbreak investigations submitted to Public Health Ontario Laboratories (PHOLs) from September 1st, 2009 to August 31st, 2011. MRVP was used to test for respiratory viruses. Outbreaks were compared based on the chronological specimen that yielded the first detected organism. **Results**: A total of 5,760 specimens from 1,707 respiratory outbreaks were tested for viruses (average 3.38, range 1to12 specimens per outbreak). Of all outbreaks, 1,454 (85.2%) had at least one virus detected. Of those, 1,142 (78.6 %) had a virus detected on the 1st specimen, 229 (15.7 %) on the 2nd specimen, 48 (3.3 %) on the 3rd specimen, and 23 (1.6 %) on the 4th specimen. Only 12 (0.8%) of the remaining outbreaks had the 1st virus detected on the 5th or subsequent specimen. Eighty (5.5%) of positive outbreaks had a new 2nd, 3rd or 4th virus identified on the 5th or subsequent specimen and in 8 (10%) of these the new virus was influenza. **Conclusions**: In 1,442 (99%) of MRVP-positive respiratory outbreaks a virus was identified by testing 4 or less specimens, and in only 0.8% of outbreaks was the first positive specimen obtained by testing more specimens. A 2nd, 3rd or 4th respiratory virus was detected after the 4th chronological specimen in 5.5% of positive outbreaks. This study suggests that initial testing of 4 specimens is sufficient to detect viral causes of respiratory outbreaks. Further viral testing should be reserved for a small minority of outbreaks where a pathogen is not found and the outbreak continues to evolve.

**Board 194. Influenza B Virus Outbreak at a Boys' Residential School in Northern Bangladesh, 2011**

**F. Haque**[1,2], M.J. Hossain[1], A.D. Khan[1], R. Rahima[2], S.M. Hasan[1], E.S. Gurley[1], K. Sturm-Ramirez[1,3], A. Mikolon[1,3], M. Rahman[1], M. Rahman[2], S.P. Luby[1,3];
[1]International Centre for Diarrhoeal Diseases Research, Bangladesh (ICDDR,B), Dhaka, Bangladesh, [2]Institute of Epidemiology, Disease Control and Research (IEDCR), Dhaka, Bangladesh, [3]Centers for Disease Control and Prevention, Atlanta, GA, USA.

**Background:** In August 2011, national newspapers and TV channels reported an outbreak of an unknown febrile illness in northern Bangladesh, which affected many residential students of a boys' school. A multidisciplinary team investigated this outbreak to determine the etiology of illness and possible exposures. **Methods:** We defined a case of influenza-like-illness (ILI) as any person from the school with documented fever (>100 $^{o}$F) and cough or sore throat. We visited the hospital to identify admitted ILI cases and also visited the school to identify additional ILI cases and explore the environmental context. We listed the ILI cases between 1 and 13 August. Using a structured questionnaire, we noted their clinical profiles and exposure histories. We collected nasal and throat swabs from 22 hospitalized cases. Using real time RT-PCR, we tested the specimens for influenza A and B viruses, respiratory syncytial virus, human metapneumovirus, human parainfluenza virus and adenovirus. **Results:** The index case was a 16 year old residential student who developed mild ILI on 4 August. We found a total of 28 ILI cases including the index case from the dormitory; 27 of these were hospitalized (including 26 students and 1 teacher). The dormitory, a two room structure of only 44 square meters, housed 55 students and 4 teachers; the attack rate within the school's dormitory was 47% (28/59). Among the hospitalized cases, 2 developed illness on 7 August and 25 on 9 August and their average age was 10 years (range: 6-21 years). In addition to fever, symptoms among hospitalized cases included cough (82%), headache (63%), runny nose (52%), sore throat (33%) and respiratory distress (11%). All 22 swab specimens tested for various pathogens were positive for influenza B virus only. Hospitalized cases received supportive care and 23 out of 27 patients received antibiotics. All patients were discharged after 5 days. **Conclusions:** Overcrowded living conditions in the school's dormitory could have facilitated the person-to-person transmission of influenza B, leading to this explosive outbreak. Crowded schools represent an important site for rapid transmission of respiratory pathogens. Research on strategies to identify such high-risk settings and to reduce transmission within them could slow the transmission of a high mortality new pandemic virus.

**Board 195. Large Norovirus Outbreak Associated with Person-to-Person Transmission, U.S. Air Force Academy, July 2011**

**A.S. Chapman**[1], C.T. Witkop[2], J.D. Escobar[1], C.A. Schlorman[1], L.S. DeMarcus[1], L.M. Marmer[1], M.E. Crum[1];
[1] United States Air Force School of Aerospace Medicine, Wright-Patterson AFB, OH, USA, [2]10th Medical Group, United States Air Force Academy, Colorado Springs, CO, USA.

**Background:** Acute gastrointestinal illnesses are a major burden on the U.S. population, causing approximately 179 million episodes of symptomatic infection, 473,000 hospitalizations, and 5,000 deaths per year. At least 50% of gastrointestinal outbreaks are associated with the genus *Norovirus*, of the family *Caliciviridae*. The U.S. Air Force Academy previously experienced a large point source *Norovirus* outbreak in July 1988 that was linked to a contaminated food ingredient used to prepare a specific meal in the dining facility. **Methods:** In July 2011, an outbreak of *Norovirus* occurred among first year cadets engaged in an annual 2-week field training exercise. A case control study was conducted to assess potential exposures resulting in illness. **Results:** The outbreak resulted in 290 confirmed and suspected cases among cadets, medical staff, and food service personnel working at the field training site. The investigation suggested the virus was likely introduced to the relatively confined cadet population by one or more food service workers, although an asymptomatic cadet could not be ruled out. An initial spike in vomiting and diarrhea cases was epidemiologically linked to contact with contaminated common use utensils in the dining facility rather than a foodborne vehicle. Close proximity to someone vomiting inside the sleeping tent (odds ratio (OR) 1.7; p=0.03) and cleaning a bathroom at the training site (OR 1.7; p=0.04) were reported more commonly among cases compared to controls. Additionally, consuming or drinking water from bathroom sinks against medical recommendation was much more frequently reported among cases (OR 2.7; p=0.0001). **Conclusions:** The majority of reported cases (70%) during this outbreak resulted from person-to-person transmission that was facilitated by episodes of public vomiting, excessive soiling of bathrooms which the first-year cadets were required to clean, and poor personal hygiene practices in the field environment.

**Board 196. Decline in Foodborne Disease Outbreaks Associated with Pork, United States, 1998–2008**

**A. Fothergill**[1], L.H. Gould[2];
[1]Rollins School of Public Health, Emory University, Atlanta, GA, USA, [2]Centers for Disease Control and Prevention, Atlanta, GA, USA.

**Background:** The average American eats nearly 49 pounds of pork in a year. We reviewed foodborne disease outbreaks associated with this commodity. **Methods:** We obtained data from the CDC's Foodborne Disease Outbreak Surveillance System for outbreaks during 1998-2008 in which pork was implicated as the single food commodity. We analyzed outbreak frequency and size, number of hospitalizations and deaths, etiologies, and settings of food preparation. Changes in reporting trends over time were evaluated by linear regression. **Results:** Pork was reported as the implicated food commodity in 259 outbreaks, resulting in 5,113 illnesses, 294 hospitalizations, and 2 deaths. There was an average of 24 outbreaks a year (range, 19-30) and an average of 20 ill persons per outbreak (range, 2-198). A single pathogen was identified for 180 (69%) outbreaks. Among these, the most common pathogens were *Staphylococcus* enterotoxin (56 outbreaks), *Salmonella* (43), *Clostridium* (38) and norovirus (26). Outbreaks were reported from 37 states and Puerto Rico; more than half (61%) were reported from only 9 states. The most commonly reported locations where food was prepared were in a restaurant or deli (46%) and private home (19%). Outbreaks were distributed fairly evenly across seasons: fall (29%), spring (25%), summer (24%), and winter (22%). The number of outbreaks declined significantly from 27 in 1998 to 19 in 2008 (p=0.004), which was largely driven by a 40% decrease in *Staphylococcus* enterotoxin outbreaks. . The percentage of outbreaks where food was prepared in a restaurant or deli decreased from 55% in 1998 to 42% in 2008. **Conclusions:** During these 11 years, the number of outbreaks due to pork decreased significantly and was driven by a decline in the number of

*Staphylococcus* outbreaks. Further understanding factors that might have influenced this decline, including contamination of pork products, changes in laboratory capacity, and changes in food handling practices will help to improve the safety of pork products and identify control measures.

**Board 197. *Bordetella holmesii* Epidemiology During a Large Outbreak of Pertussis-Like Illness in Ohio; 2010 through 2011**

**L. Rodgers**[1,2], A. Cohn[1], S. Martin[1], J. Budd[2], A. Terranella[1], S. Mandal[1], L. McGlone[1], A. Emanuel[3], L. Tondella[1], L. Pawloski[1], K. Tatti[1], M. Marcon[4], K. Spicer[4], A. Leber[4], D. Salamon[4], R. Iyer[5], N. Tucker[5], C. Hicks[5], M. DiOrio[2], E. Koch[3], M. LeMaile-Williams[3], T. Clark[1];
[1]Centers for Disease Control and Prevention, Atlanta, GA, USA, [2]Ohio Department of Health, Columbus, OH, USA, [3]Columbus Public Health, Columbus, OH, USA, [4]Nationwide Childrens Hospital, Columbus, OH, USA, [5]Franklin County Board of Health, Columbus, OH, USA.

**Background:** During 2010, reported pertussis incidence in the United States was 8.9 cases/100,000 persons, a rate equal to the 2004 resurgence. During May 2010-May 2011, a suspected pertussis outbreak was reported in Ohio; a majority of cases were classified on the basis of a highly sensitive single target polymerase chain reaction (PCR) (IS*481*) susceptible to false-positivity. Difficulties with pertussis diagnosis and uncharacteristic age distribution prompted an investigation. **Methods:** We evaluated demographics, clinical signs and symptoms, vaccination status, and laboratory diagnostic methods. We conducted serologic testing of suspect cases and retrospective analyses of IS*481*-positive specimens by using a multitarget PCR that differentiates between *Bordetella pertussis, Bordetella parapertussis,* and *Bordetella holmesii*. **Results:** A total of 843 suspected, probable, and confirmed cases were reported (incidence 73/100,000). Discordance between strongly positive PCR results and negative serologic findings highlighted a possibility of other *Bordetella* species. Of 276 specimens tested for *Bordetella* sp. by PCR, *B. pertussis* and *B. holmesii* were confirmed in 128 (46%) and 51 (18%), respectively and 5 (2%) had both. Among 128 *B. pertussis* patients, 85% had paroxysmal coughing; 37% had posttussis emesis; and 19% had whoop; median cough duration was 22 (range: 2-82) days. Presentation was similar for *B. holmesii* patients (paroxysms 89%, emesis 43%, and whoop 24%); median cough duration was 18 (range: 6-119) days. Among 51 patients with *B. holmesii* 61% were aged 11-18 years, compared with 35%/128 patients with *B. pertussis* ($P = .002$). Among 31 adolescents aged 11-18 years with *B. holmesii*, 18 (58%) had received a tetanus-diphtheria-acellular pertussis vaccine before illness onset, compared with 20/45 (44%) patients with *B. pertussis* ($P = .24$). **Conclusions:** This is the first documented mixed outbreak of *B. pertussis* and *B. holmesii*. Although *B. pertussis* was confirmed as primary etiologic agent, *B. holmesii* contributed substantially, particularly among adolescents. These data highlight importance of multitarget PCR testing to differentiate *Bordetella* species. Further study of *B. holmesii* and pertussis-like illness is needed.

## H1N1 Influenza

Tuesday, March 13
12:30 PM – 1:30 PM
Grand Hall

**Board 198. Use of Two Pre-existing Influenza Surveillance Systems in Vietnam's Response to Pandemic Influenza A/H1N1/2009, May 2009 to July 2010**

**B.K. Kapella**[1], N.V. Binh[2], T.T. Duong[2], P.T. Lan[2], N.T. Hien[3], T.N. Duong[4], P. Hung[2], P.V. Kien[2], T.X. Tung[3], J.C. Kile[1], V.N. Long[2];
[1]U.S. Centers for Disease Control and Prevention, Hanoi, Viet Nam, [2]Department of Preventive Medicine,

Ministry of Health, Hanoi, Viet Nam, [3]National Institute of Hygiene and Epidemiology, Hanoi, Viet Nam, [4]Pasteur Institute, HCMC, Viet Nam.
**Background:** In April 2009, a new influenza virus was reported in North America. Within weeks, influenza A/H1N1 2009 was being detected around the world. Vietnam quickly gained the capacity to test for the virus, and prepared a containment strategy. Vietnam's surveillance systems, designed to track seasonal influenza, were poised to provide data to the Ministry of Health (MOH). **Methods:** Since 1998, influenza-like illness (ILI) has been a reportable clinical syndrome in Vietnam. Since 2006, Vietnam has also had active laboratory enhanced sentinel surveillance for influenza. Data were analyzed from both systems to assist the MOH in pandemic policy determination. **Results:** The clinical surveillance system identified the first case of influenza A/H1N1 2009 in Vietnam on 31 May 2009. From 31 May to 15 July 2009, 300 of the 337 (89%) laboratory-confirmed cases had a history of travel from countries with known H1N1 2009 within the previous 14 days. On 16 July 2009, the clinical surveillance system identified the first community outbreak of H1N1 2009 in Vietnam in a secondary school in Ho Chi Minh City. Results of the sentinel surveillance system showed that H1N1 2009 activity peaked during 10-16 September 2009, with 99% of positive specimens confirmed in the influenza sentinel surveillance as H1N1 2009. By mid-October, the case count for laboratory-confirmed cases in Vietnam surpassed 10,000, and the MOH stopped reporting individual cases and switched from a strategy of containment to mitigation. Consistent data from the sentinel surveillance showed a decline in cases from September 2009 until none were reported in May 2010, and only one in June 2010. Based on this data, the MOH reported the pandemic nationally controlled on 14 July 2010. Conclusions: The clinical and sentinel influenza surveillance systems in Vietnam allowed health authorities to detect and track H1N1 2009, and to issue informed data-driven policies in an effort to reduce the spread of H1N1 2009 influenza into and within the country during the critical first months of the pandemic. It also allowed the MOH to determine the peak of activity and to direct resources appropriately after the first wave was over. Such evidence-based decision making during the pandemic would not have been possible without these pre-existing surveillance systems for seasonal influenza.

**Board 199. The 2009 Influenza Pandemic: A Brief Review of the Strengths and Weaknesses of the New Zealand Response**

**N. Wilson**, J.A. Summers, M.G. Baker;
University of Otago, Wellington, New Zealand.
**Background:** We aimed to review the health sector response to the 2009 influenza pandemic in New Zealand (one of the first countries to report cases). **Methods:** The journal literature was found to contain over 40 Medline-indexed publications relating to this country and we also identified online reports from government agencies. **Results:** From this literature it appears that in 2009, 27% of the population had evidence of infection from the pandemic strain, 1122 people were hospitalized (with pandemic influenza as the primary diagnosis), 119 of those hospitalized were treated in intensive care units, and there were 49 pandemic-attributed deaths. The health burden was relatively worse for Maori (indigenous New Zealanders) and Pacific peoples. **Conclusions:** The available evidence suggests that the overall response in the public health, primary care and hospital sectors was appropriate and well organized. Nevertheless, a number of opportunities seem to have been missed, including a lack of the following: (i) a detailed review of the epidemiology and health sector response; (ii) sophisticated analytic studies to identify risk factors (e.g., using case-control studies); (iii) studies on pandemic vaccine uptake and public acceptability; and (iv) evaluation of the health protection messages that were used in campaigns and in media releases from health authorities. There is still time to rectify some of these deficits in the New Zealand context. Similarly, it could be useful if all countries completed reviews of the response to this pandemic so as to inform future pandemic planning and disaster management at both the national and international levels.

**Board 200. Dynamic Trends of Circulating Influenza Viruses in a Rural Indian Community under Active Surveillance during Pandemic and Post-Pandemic Periods**

**S. Broor**[1], K. Fowler[2], V. Gupta[1], B.B. Singh[1], R. Kumar[1], A. Krishnan[1], D.R. Purakayastha[1], S. Dalal[1], M.-A. Widdowson[3], R.B. Lal[3], W.M. Sullender[2];
[1]All India Institute of Medical Sciences, New Delhi, India, [2]University of Alabama at Birmingham, Birmingham, AL, USA, [3]Centres for Disease Control and Prevention, Atlanta, GA, USA.

**Background:** Three villages in Ballabgarh located in Haryana state of north India are under prospective weekly active surveillance for febrile acute respiratory illness (FARI) under the Comprehensive Rural Health Services Project (CRHSP), of All India Institute of Medical Sciences (AIIMS) as part of trial of seasonal inactivated trivalent influenza vaccine (TIV) in children. This provided a unique opportunity to study the prevalence of influenza viruses in this community during the pandemic and post pandemic periods. **Methods:** Weekly house-to-house surveillance for FARI by trained field workers is being carried out in this dynamic cohort of >18000 individuals. Nasal and oropharangeal swabs are collected from reported FARI cases and transported to the virology laboratory at AIIMS within 24 h. Influenza viruses are detected by real time RT-PCR and influenza A positive samples are subtyped using primers and probes for the HA genes of 2009A/H1N1, seasonal H1 and H3. **Results:** From November 2009 to July 2011, 1729 (12%) of 13,960 samples from FARI cases were influenza-positive. Among these, 768 (44%) were identified as 2009A/H1N1, 282 (16%) as seasonal influenza A/H3, and 679 (39%) as influenza B viruses. The prevalence of influenza types and subtypes varied significantly over the study period. First, a distinct peak of 2009A/H1N1 was observed in November (15% of all FARI samples tested for the month) and December 2009 (33%) with a second wave during the post-pandemic phase in August (15%) to September 2010 (19%). Influenza B viruses co-circulated at almost similar levels (range 2-16%) during the same period. Low levels (<1% of FARI samples positive) of influenza activity were observed during October 2010-June 2011, followed by the distinct appearance of seasonal influenza A/H3 viruses in July 2011 (18% positivity). **Conclusions:** These data show a complex pattern of circulating influenza viruses in a large surveillance setting in this rural Indian community during pandemic and post pandemic phases. The circulating viral strains are comparable to what is being observed in other parts of India, as well as globally. *These studies were supported in part by cooperative agreements U01 IP000177 from the Centers for Disease Control and Prevention, Atlanta, USA.*

**Board 201. Study of Influenza A Virus Nucleoprotein Interaction with Cellular Heat Shock Protein 40 And its Role in Nuclear Import of Influenza Ribonucleoprotein Complex**

**S. Tripathi**, K. Shukla, H. Nailwal, S.K. Lal;
International Center for Genetic Engineering And Biotechnology, New Delhi, India.

**Background:** Influenza A viruses (IAVs) are major cause of worldwide human morbidity, and cause recurrent pandemics. They quickly acquire mutations and become resistant to the drugs targeting viral proteins. To circumvent this problem, virus-host protein interactions, which help in virus replication, can be targeted. Thus, it becomes imperative to identify novel virus-host interactions and their role in viral life cycle. Earlier we reported that IAV nucleoprotein interacts with host chaperone heat shock protein (Hsp40) (Sharma K. *et al,* 2011). It is known that Hsp40 is upregulated under stress conditions and localizes to nucleus. In current study we investigated whether the IAV NP exploits this attribute of Hsp40 to promote nuclear import of IAV ribonucleoprotein complex. **Methods and Results:**Interaction between IAV NP and Hsp40 was characterized by co-immunoprecipitation studies using purified proteins. Domain mapping studies indicated that Hsp40 binds to IAV NP through its C-terminal peptide binding domain and its N terminal J domain is dispensable for the interaction. A time course study of cellular localization showed that Hsp40 and IAV NP colocalize in nucleus during early stages of virus replication, indicating role of Hsp40 in nuclear import of IAV NP. To check this we studied the effect of Hsp40 overexpression on nuclear localization of ectopically expressed IAV NP. It was observed that

increased Hsp40 expression enhanced the amount of IAV NP present in nuclear fraction. Further, it was observed in IAV infected cells, Hsp40 overexpression enhanced virus replication and siRNA mediated inhibition of Hsp40 expression reduced IAV replication. Treatment of IAV infected cells with Hsp40 inhibitor drug KNK437 led to reduction in the virus titers significantly. Further characterization of IAV NP-Hsp40 interaction and its role in nuclear import of IAV RNP complex is being studied currently. **Conclusions:** Our study shows that IAV NP can subvert Hsp40 function in multiple ways, and exploit this interaction to promote virus replication as well as inhibit anti-viral host response. Availability of interaction domain and structural information of both NP and Hsp40, makes this interaction an ideal target to develop novel anti-influenza drug candidates.

**Board 202. Influenza Virus Seroprevalence among Pregnant Women in Kerala State, India**

**G. Ashok**, C. UMA, S. SREERAJ, S. SAMPADA, S. ANIRBAN, G. JIJI, G. ARUNKUMAR;
Manipal Centre for Virus Research (ICMR Virology Lab-Grade-I & Regional Reference Lab for Influenza virus), Manipal University, Manipal, India.

**Background**: During the 2010 peak of the pandemic Influenza A/H1N1/2009 virus activity, the South Indian State, Kerala reported a steep rise in maternal mortality associated with Influenza. Hence to estimate the seroprevalence of Influenza virus infection among antenatal women in North Kerala we conducted an unlinked anonymous sentinel sero-survey in May 2011, just before the arrival of Monsoon. **Methods:** An unlinked anonymous sentinel cross sectional sero-survey was conducted at the antenatal clinics of Taluk Head Quarters Hospital (THQH), Thirurangady, Malappuram, District Hospital, Calicut and District Hospital, Kannur, Kerala State, India from 16$^{th}$ to 31$^{st}$ May 2011. Anti-Influenza virus antibodies were detected by Haemagglutination Inhibition Assay (HAI) using WHO/CDC Influenza HAI kit for 2010/2011. Human O group RBCs were used for HAI. An HAI titre of ≥40 was considered positive. **Results**: Of the 232 subjects 96 (41.4%) were from THQH, Thirurangady, 65 (28%) from DH, Calicut and 71(30.6%) from DH, Kannur. The mean and median age of the study population was 26.84 ±4.74 and 27 years (n=94) respectively. None of the study subjects received influenza vaccination any time during their life. The overall seroprevalence of Influenza A/H1N1/2009 virus infection was 23.3% whereas that of Seasonal Influenza A/H1N1 was 44.4%, Influenza A/H3N2 was55.8%, Influenza B (Yamagata) was 79.7% and Influenza B (Victoria) was 69.8%. Further, 23.7% of study participants had hemagglutination inhibition antibodies against all circulating types of influenza viruses while 20.3% participants were naïve to any influenza A/B infection. **Conclusions:** Nearly three-fourths of the pregnant women attending the antenatal clinics in north Kerala remain susceptible to pandemic Influenza A /H1N1/2009 virus and one fifth of them are naïve to any circulating influenza viruses in the pre-monsoon period in 2011. As none of the participants received influenza vaccination during their life time the prevalence obtained in this study was the true representation of influenza activity. Targeted intervention in terms of more awareness among antenatal women and obstetricians coupled with early identification of cases and treatment and vaccination of pregnant women may significantly reduce the mortality of influenza virus infection in pregnant women.

## Laboratory Support

Tuesday, March 13
12:30 PM – 1:30 PM
Grand Hall

**Board 203. Preliminary Results from *N. gonorrhoeae* Surveillance and Drug Resistance in Djibouti**

**K. Chisholm**[1], E. Dueger[1,2], H.Y. Darar[3], A.A. Ahmed[4], S.F. Ahmed[5], M. Wasfy[1], P. Sebeny[5];
[1]Global Disease Detection and Response Program, U.S. Naval Medical Research Unit No. 3, Cairo, Egypt, [2]Global Disease Detection and Emergency Response Program, U.S. Centers for Disease Control and Prevention, Atlanta, GA, USA, [3]Centre Yonis Toussaint, Ministry of Health, Djibouti, Djibouti, [4]National Institute of Public Heatlh, Ministry of Health, Djibouti, Djibouti, [5]Bacterial and Parasitic Disease Research Program, U.S. Naval Medical Research Unit No. 3, Cairo, Egypt.

**Background:** *Neisseria gonorrhoeae* is a common sexually transmitted infection (STI) worldwide, yet prevalence and drug resistance patterns in the Horn of Africa are generally unknown. **Methods:** Laboratory diagnostic capacity and surveillance for *N. gonorrhoeae* were established in September 2010 at Centre Yonis Toussaint (CYT) in Djibouti. Patients presenting with signs and symptoms of STI are enrolled and provide epidemiologic data, urethral or cervical swabs and urine samples for analysis. Bacterial culture and antibiotic sensitivity testing (AST) are performed at CYT, after which bacterial isolates and urine samples are sent to U.S. Naval Medical Research Unit No. 3 for confirmatory testing, further AST and nucleic acid amplification testing (NAAT). **Results:** After 1 year of surveillance, 182 patients (55.4% men and 83.9% Djiboutian) had enrolled in surveillance activities. Fifteen (16.9%) patients reported having already taken medication for their current infection, and 12 (13.2%) reported having previously had a similar infection. The most common signs and symptoms were discharge (98.0%), painful urination (92.0%) and burning (78.0%) for men and discharge (90.2%), abdominal pain (78.1%) and itching (61.0%) for women. Of the 104 patients for whom culture results from CYT are available, growth was noted in 38 (36.6%) samples, 22 (21.2%) of which were identified as *N. gonorrhoeae*. Due to storage and transportation issues, only 6 of 35 isolates were confirmed *N. gonorrhoeae*-positive by culture at NAMRU-3. These 6 samples showed 100% resistance to penicillin and tetracycline, 16.7% resistance to levofloxacin and ciprofloxacin, and no resistance to azithromycin, cefepime, ceftazidime or ceftriaxone. NAAT results from 135 urine samples indicated *N. gonorrhoeae* infection in an additional 49 samples (including 88.9% of samples identified as *N. gonorrhoeae* at CYT) for an overall prevalence of 40.7% among surveillance participants. Enrolled patients with confirmed *N. gonorrhoeae* were statistically more likely to be male, to be unmarried, to be employed, to use *khat*, or to have had a new partner in the previous three months. **Conclusions:** Preliminary results suggest that *N. gonorrhoeae* is a common cause of STI in Djibouti and that continued surveillance is needed to determine the extent of antibiotic resistance.

**Board 204. Meeting Obligations of the International Health Regulations (IHR) (2005): Mapping Public Health Laboratories and Assessing Their Performance for Infectious Disease Detection in Thailand**

**A.H. Peruski**[1], M. Birmingham[2], C. Tantinimitkul[2], L. Chungsamanukool[3], P. Chungsamanukool[3], R. Guntapong[3], C. Pulsrikarn[3], L. Saengklai[3], K. Supawat[3], A. Thattiyaphong[3], D. Wongsommart[3], W. Wootta[3], A. Nikiema[4], A. Pierson[4], C. Fridlund[5], L. Peruski[5], S. Maloney[5], X. Liu[5];
[1]WHO, currently at Boston University School of Public Health, Boston, MA, USA, [2]WHO, Bangkok, Thailand, [3]Department of Medical Sciences,Thailand Ministry of Public Health, Bangkok, Thailand, [4]Integrated Quality Laboratory Services, Lyon, France, [5]Centers for Disease Control and Prevention, Atlanta, GA, USA.

**Background:** To meet obligations under the IHR (2005), the World Health Organization (WHO), US-Centers for Disease Control and Prevention (CDC), CDC Global Disease Detection Thailand Regional Center and Thailand Ministry of Public Health (MOPH) worked collaboratively to map and assess the national public health laboratory (PHL) system: 1) In terms of surveillance, networking, quality assurance, and data management, laboratories were quantitatively and qualitatively reviewed using the WHO laboratory assessment tool (LAT); 2) a database of findings, including GIS mapping of PHLs and their catchment areas was produced; and 3) a program to strengthen the national PHL system based on the findings is being developed. **Methods:** The WHO LAT was adapted to Thailand through a participatory process of WHO, CDC, and MOPH. The adapted version employed a specific scoring matrix for the assessment and comprised 15 subsections listed below. Each subsection had a quantitative output. Two trained teams composed of WHO and MOPH personnel jointly performed the onsite assessments in December 2010 over a 2-week period in a total of 17 PHLs around the country. **Results:** Based on the average scores of the 17 PHLs assessed, among the 15 sections: Budget & finances, Quality, Data management, and Communications were strong areas (>90%), Supplies, Buildings & facilities, Biosafety, Sampling, Equipment, and public health functions scored very good (80-90%), and Information technologies, Staffing level, Training and supervision, and Tests performed needed improvement (60-70%). The findings highlighted both strengths and weaknesses of the PHL system as a whole and of the individual laboratories though the scores are not valid for comparisons with other countries due to a country specific scoring matrix. **Conclusions:** The LAT was successfully adapted to the Thai context through a participatory process and used by both national and external laboratory personnel. The findings of this assessment highlighted strengths, and should also help stimulate additional resources and efforts to address some of the gaps in order to meet core capacity obligations of the IHR (2005). Finally, this is the first time that this process has been used to assess laboratories in Asia and could serve as a paradigm for building laboratory capacity to meet IHR obligations.

**Board 205. Recovery of Live Virus from ViveST™ After Storage at Ambient Temperature**

**K.L. Barr**[1], G.L. Heil[1], K. Reese[2], G.C. Gray[1];
[1]University of Florida, Gainesville, FL, USA, [2]ViveBio, Norcross, GA, USA.

**Background:** A major impediment to performing virological field studies in developing nations is the lack of ultralow freezers as well as the expense and difficulty of shipping frozen samples. Traditionally, biological samples could be shipped dried on filter paper or FTA cards; however, viruses often lost viability. A recently developed product, ViveST™, provides the ability to store up to 1mL of biological specimen on a synthetic matrix at ambient temperature for use in specimen storage and transportation. The matrix is housed on the screw-cap of a tube such that the sample is self-contained during storage and shipping. This product has been approved for the recovery of viral nucleic acids from plasma and cell culture supernatants but its applications as a viral recovery device have not been evaluated. **Methods:** The capacity of the ViveST™ device to preserve live virus at ambient temperature was evaluated via attempts to recover live virus from serum and cell culture supernatants that had been stored at ambient temperature for 1 to 4 weeks. Several enveloped and non-enveloped viruses were stored using the ViveST™ including: human adenovirus 14p, influenza virus H1N1 (Brisbane), dengue virus 2 (NGC), echovirus 3, rhinovirus 15, Coxsackie virus B5 (Faulkner) and herpes simplex virus 1. The viability of virus was verified via titration using plaque assays or focus-forming unit assays. **Results:** Preliminary viral titer studies indicated that ViveST™ preserved several types of live virus for periods ranging from 1 to 4 weeks at ambient temperature. **Conclusions:** These data suggest that ViveST™ might be useful in field specimen collection scenarios where ultra-cold storage is not available.

**Board 206. The Centers for Disease Control and Prevention Real Time RT-PCR Assay for Diagnosis of Dengue: Analytical Validation and Clinical Application**

**J.L. Munoz-Jordan**, G.A. Santiago, E. Vergne;
Centers for Disease Control and Prevention, San Juan, PR, USA.

**Background:** Dengue is an illness caused by infection with any one of four related dengue virus (DENV) serotypes (DENV-1,-2,-3 and-4) which are transmitted by *Aedes* sp. mosquitoes, and an estimated 50 million people in approximately 100 countries present with dengue annually. Laboratory diagnosis of dengue is best made during the acute phase of the illness when DENV circulates in the blood and can be detected by assays for the viral RNA genome or antigens to DENV. IgM antibody to DENV is also produced during this time, but only becomes detectable at days 3-5 after onset of fever. **Results:** We have validated primer and probe sets to detect DENV-1, -2, -3, and -4 RNA in serum and plasma. These primers and probes have been designed with consideration to currently circulating DENV strains from around the world. The assay contains a Human Specimen Control (HSC), a noninfectious cultured human cell component that provides a positive signal and demonstrates successful recovery of RNA as well as the integrity of the RNA extraction reagent. Several RNA extraction and RT-PCR protocols have been validated for this assay. The assay can be run in singleplex (each DENV serotype detected in a separate reaction) or in multiplex (the four DENV serotypes run in the same reaction). These two formats provide equal sensitivity of DENV detection. This assay has also been validated in retrospectively and prospectively collected serum samples from patients over an illness range of 0-5 days. The assay is 80% sensitive and 99% specific in patients confirmed as having dengue using seroconversion to IgM anti-DENV in paired specimens. **Conclusions:** This assay can be used to make the diagnosis of dengue in a patient early in the course of a dengue-like illness. Detection of DENV during this time period is considered diagnostic for dengue, dengue hemorrhagic fever, or dengue shock syndrome. The qualitative detection and identification of DENV-1, -2, -3 and -4 from serum or plasma can be used to provide epidemiologic information for surveillance of DENV serotypes.

## Effective and Sustainable Surveillance Platforms

Tuesday, March 13
12:30 PM – 1:30 PM
Grand Hall

**Board 207. Evaluation of Immunoblot Test Results Compared with the Council of State and Territorial Epidemiologists Case Definition for *Bordetella pertussis*—Maricopa County, Arizona, February–July 2011**

**S. Yasmin**[1], R. Klein[2], K.M. Bisgard[1], S.W. Martin[1], R.H. Sunenshine[1];
[1]Centers for Disease Control and Prevention, Atlanta, GA, USA, [2]Maricopa County Department of Public Health, Phoenix, AZ, USA.

**Background:** Pertussis is endemic in the United States; 27,550 U.S. cases were reported in 2010. Difficulties associated with diagnosis make public health surveillance challenging. Currently, except for Massachusetts, the Council of State and Territorial Epidemiologists (CSTE) case definition does not include serologic confirmation; however, multiple serologic tests with unknown clinical accuracy are commercially available. We assessed the increased use of serologic tests in a defined geographic area to better understand its potential impact on pertussis surveillance. **Methods:** We included suspect cases reported to Maricopa County from February 1 to July 31, 2011. Laboratory method and demographic data were obtained; interviews were conducted to determine if illness met the clinical case definition [cough for ≥14 days with either paroxysms of cough, inspiratory whoop, or posttussive vomiting].

Patients diagnosed at one laboratory where any positive ELISA result (for IgA, IgM, or IgG to antigens filamentous hemagglutinin (FHA) or pertussis toxin (PT) triggers an immunoblot test of the corresponding positive antibody were further described. Since IgG-PT has been shown to have better accuracy, we included patients with IgG-PT/FHA results. **Results:** A total of 1026 suspected pertussis cases were reported (median age 46, age range 0-97 years, 64.3% female); 29 (2.82%) were culture- or PCR-confirmed (median age <1 year, age range 0-29 years, 58.6% female); 921 (89.8%) had serologic testing (median age 48, age range 0-95 years, 65% female). Of these, 439 (42.8%) were interviewed; 199 (19.4%) were diagnosed using ELISA and immunoblot at one laboratory. The mean age among 199 patients was 46.5 (range: 1-87, median 49) years; 71% were female. Among 142 clinical cases, 57 (40%) were IgG-positive. Among the 74 IgG-positive patients, 57 (77%) were clinical cases. Compared with those not meeting the case definition, patients with a case were younger (mean age 46 vs 52 years; $P=0.05$). **Conclusions:** The high use of serology demonstrates the need for further investigations into its validity and its potential impact on pertussis surveillance. No single diagnostic test is useful during all stages of pertussis; serology might offer improved late diagnosis, but healthcare provider education is needed to ensure appropriate use.

**Board 208. Cluster Surveillance for Detecting Encephalitis Outbreaks in Bangladesh**

**A.M. Naser**[1], M.J. Hossain[1], H.M. Sazzad[1], N. Homaira[1], E. Gurley[1], G. Podder[1], S. Afroz[1], P. Rollin[2], P. Daszak[3], B.-N. Ahmed[4], M. Rahman[5], S. Luby[1,2];
[1]ICDDR,B, Dhaka, Bangladesh, [2]Centers for Disease Control and Prevention, Atlanta, GA, USA, [3]EcoHealth Alliance, New York, NY, USA, [4]Disease Control, Directorate General of Health Services, Ministry of Health and Family Welfare, Dhaka, Bangladesh, [5]Institute of Epidemiology Disease Control and Research, (IEDCR), Ministry of Health and Family Welfare, Dhaka, Bangladesh.

**Background:** Emerging and reemerging viruses can cause encephalitis outbreaks. Etiological testing of all encephalitis cases to identify emerging pathogens is prohibitively expensive for surveillance in low income countries. In 2006, Institute of Epidemiology, Disease Control & Research and International Centre for Diarrhoeal Disease Research, Bangladesh established surveillance in 10 government hospitals in Bangladesh focusing on detecting clusters of encephalitis. We evaluated the efficiency of this surveillance to detect potential pathogens of public health importance, using Nipah virus cluster detection as a model. **Methods:** Surveillance physicians listed patients admitted to medicine or pediatric units with acute onset of fever and altered mental status, seizure or other neurological deficits. Physicians reviewed line-listed data and asked patients or caregivers about other sick persons or recent deaths with similar symptoms in their communities to assess if a case was part of a cluster. We defined a cluster as at least 2 cases living within 30 minutes walking distance of each other who developed similar symptoms within 3 weeks of each other. An investigation team visited clustered cases in the hospitals or in communities to collect illness and exposure histories. The team collected blood from living cases. We tested serum specimens by Nipah-specific IgM ELISA. **Results:** Between 2006 and 2011, physicians listed 5406 patients in surveillance hospitals. We identified 60 clusters consisting of 172 cases; 46 (77%) clusters had 2 cases per cluster, 6 (10%) clusters had 3 cases per cluster and 8 (13%) clusters had more than 3 (range: 4 - 20) cases per cluster. We confirmed Nipah virus infection in 10 (17 %) clusters; 3 were two-case clusters and 7 clusters included more than 3 cases. The etiology of one cluster of 5 cases, which included 2 deaths, remains unknown. Of the total 172 cluster-cases, 55 (32 %) had Nipah infection. **Conclusions:** Cluster surveillance narrowed the focus from thousands of clinical cases to a smaller number that could be investigated in greater detail. This approach efficiently identified Nipah virus outbreaks, and could be used to direct resources to identify transmission of other emerging pathogens of public health importance by expanding diagnostic tests for clusters of unknown etiology.

**Board 209. The Multidrug-Resistant Organism Repository and Surveillance Network (MRSN)**

P. Waterman, Y. Kwak, R. Clifford, M. Riley, J. Hung, C. Black, P. McGann, **E. Lesho**;
Walter Reed Army Institute of Research, Silver Spring, MD, USA.

**Background:** The mission of the MRSN is enterprise-wide collection and characterization of multidrug-resistant organisms (MDRO) to inform infection control, antibiotic usage, and healthcare policy. **Methods:** Increasing numbers of hospitals submit MDRO when isolated from infections or surveillance activities. Canine MDRO are collected along the breeding and training centers for military working dogs. Identity and susceptibility is confirmed in triplicate on all the major automated systems (bioMérieux, Siemens, Beckon Dickinson) and on validated broth microdilution panels (Trek Diagnostics). Pulsed-field gel electrophoresis, polymerase chain reaction for drug resistance genes, optical genome mapping (OGM) and sequencing are performed. Regular (i.e. monthly antibiograms) and event-driven (outbreak) reports are distributed. **Results:** To date, 13 hospitals (5 in war zones) submit an average of 550 isolates/month. Outbreak investigation assistance was requested 10 times. $Bla_{NDM-1}$ was detected in *Providencia stuartii* from Afghanistan. In June 2011, *qacA* was detected in 8 methicillin-resistant *S. aureus*. In July 2011, a large potential reservoir of *qacA/B*- containing coagulase-negative *Staphylococcus* sp, and a cluster of highly colistin resistant Gram negatives was detected. OGM and sequence analysis revealed clinically relevant resistance determinants in *P. falciparum*, evidence for highly mobile elements as vehicles of resistance transfer between Enterobacteriaceae, and rapid genome fluctuations in serial *A. baumannii* isolates from the same patient. Carriage of canine MDRO increased as dogs were transported along supply and training centers. **Conclusions:** The MRSN provided valuable infection control support, especially to unstable areas such as Afghanistan, Iraq, and post-earthquake Haiti. It determined sources of outbreaks, facilitated interruption of nosocomial transmissions, influenced antibiotic stewardship and surveillance policy. It was the first to detect $Bla_{NDM-1}$ in Afghanistan and *qacA/B* in the U.S., and a $Bla_{NDM-1}$ carrying plasmid in a new species.

**Board 210. Armed Forces Health Surveillance Center's Capacity Building and Biosurveillance Systems Program**

**J. Cockrill**, C. Perdue, D. Blazes;
Armed Forces Health Surveillance Center, Silver Spring, MD, USA.

**Background:** Originally established by Presidential Directive NSTC-7 in 1997, the Department of Defense (DoD) Global Emerging Infections Surveillance and Responsive System (GEIS) was expanded in 2006 and subsequently incorporated as a Division of the Armed Forces Health Surveillance Center (AFHSC) in 2008. AFHSC-GEIS divides its surveillance efforts into five categories of infectious diseases: respiratory, gastrointestinal, and febrile and vectorborne infections, antimicrobial resistance, and sexually transmitted infections. Elements of the capacity building and biosurveillance systems program (CBBSP) cross-cuts each of the infectious disease categories. **Methods:** Surveillance and response systems supporting International Health Regulations (IHR (2005)) compliance necessarily incorporate a robust public health workforce, laboratory diagnostic capability and appropriate host country support. The CBBSP seeks to assist partner countries in developing the knowledge, skills, and capabilities to create new—or enhance and support existing—public health programs and biosurveillance initiatives within their respective countries. This is accomplished through funding AFHSC-GEIS partner proposals that seek to increase 1) human resources capacity 2) electronic biosurveillance capabilities and 3) laboratory diagnostic capabilities. **Results:** More than 5,000 laboratorians and public health professionals have been involved in AFHSC-GEIS sponsored capacity building training programs since 2009. CBBSP has executed initiatives in more than 40 countries, the majority of which were low-income nations. Recent accomplishments include the development and deployment of the Suite for Automated Global Electronic bioSurveillance (SAGES) in Peru, the Philippines, and Cambodia, and the training of citizen-scientists critical laboratory techniques. **Conclusions:** The AFHSC CBBSP strategy reflects the White

House guidance for US government funding of IHR 2005 core capacity development. Moving forward, the CBBSP aims to develop and expand relationships with global health partners with respect to building core public health capacities.

**Board 211. Establishing an International Circumpolar Collaborative Tuberculosis Surveillance Working Group**

**T. Zulz**[1], V. Gallant[2], D. Scholten[2];
[1]Centers for Disease Control, Anchorage, AK, USA, [2]Public Health Agency of Canada, Ottawa, ON, Canada.

**Background:** The International Circumpolar Surveillance (ICS) project was established in 1998 to create an infectious disease surveillance network throughout Arctic regions. ICS allows for the collection, comparison and sharing of uniform laboratory and epidemiological data on infectious diseases and assists in developing prevention and control strategies. Collaborative surveillance initially focused on invasive pneumococcal disease. In 2006, due to concerns about high rates of tuberculosis (TB) in the Arctic, the ICS Steering Committee approved creation of a TB Working Group. **Methods:** An organizational meeting in 2006 with representatives from interested circumpolar regions was held in Yellowknife, NWT, Canada. An evaluation was conducted in 2007 to determine group priorities and identify critical gaps to be addressed. In person working group meetings and regular conference calls have been held. Process related issues have been addressed. Working group products have been identified. **Results:** Greenland, northern Canada, several Russian Federation regions and the U.S. Arctic (Alaska) are represented on the working group. Three in-person working group meetings were held and two meetings were also held in the Russian Federation to facilitate their involvement. The evaluation identified five goals for the group (1) improve surveillance of TB, (2) identify trends in TB epidemiology, (3) assess the incidence of TB, (4) increase awareness of TB, and (5) collaborate on TB research. The group has developed Terms of Reference, core data elements, case definitions and a draft data sharing agreement which will address data ownership and privacy issues. Five years of retrospective TB surveillance data will be collected in 2012 with the goal of producing a descriptive epidemiologic report on TB in circumpolar regions. Prospective collection of surveillance data is planned following completion of the report. **Conclusions:** The ICS TB Working Group has great potential to influence public health in circumpolar countries. Continued collaboration will provide shared data that may identify common issues and assist in developing strategies for TB detection and control.

## New Vaccines

Tuesday, March 13
12:30 PM – 1:30 PM
Grand Hall

**Board 212. Genetic Characterization of Enterovirus71 Complete Genome Isolated in Beijing**

**Z. Wei**;
Beijing Center for Disease Control and Prevention, School of Public Health and Family Medicine of Capital Medical University, Beijing, China.

**Objective:** To investigate the characterization of the complete genome of EV71 in Beijing, and to provide basis for selecting appropriate virus strain to develop vaccine. **Methods:** Twelve throat swab samples were collected from children with hand.foot.mouth disease(HFMD).One sample named 08YM-3 was cultured and isolated in vero cells.Viral RNA was extracted and carded out by RT-PCR and 5’,3’ rapid amplification of cDNA ends(RACE) to obtain the sequence from 08YM-3.PCR products were cloned and

analyzed. Nucleotide identity between sequences was calculated and sequence alignments were made to generate phylogenetic trees using MegAlign in DNAStar. **Results:** Three clones were constructed that covered EV7l complete genome.Data from sequences analysis showed tllat this viral strain named BJ08 shared 95.6%-96.7%, 88.3%-96.1%,78.1%-94.0%, 90.8%-94.6%,85.9%-94.1%and 90.9%-93.9%in 5'UTR,Pl,P2,P3, 3'UTR region and complete genome with C4 subtype respectively.BJ08 showed low nucleotides identity(<90%)with other subtypes.Phylogenetic trees established from alignment of the complete genome and VP l region indicated that BJ08 belonged to C4 subtype.BJ08 and C4 subtype strains shared the same amino acids in 6 sites in VP l region.which were associated with EV7 l subtype.There was no mutation in VP 1 antigen epitope(92-1 07aa). **Conclusions:** This BJ08 strain belonged to C4 subtype.Further study on EV7l complete genome would have great significance for vaccine research.

**Board 213. Forecasting Invasive Pneumococcal Disease Trends after the Introduction of 13-Valent Pneumococcal Conjugate Vaccine in the United States, 2010–2020**

**R. Link-Gelles**, T.H. Taylor, Jr., M.R. Moore;
Centers for Disease Control and Prevention, Atlanta, GA, USA.

**Background**: Following introduction of 7-valent pneumococcal conjugate vaccine (PCV7) for children in 2000, rates of invasive pneumococcal disease (IPD) caused by PCV7 serotypes declined dramatically among children and adults (herd effects). At the same time, rates of IPD caused by non-PCV7 serotypes increased, a phenomenon referred to as "serotype replacement". By 2010, when 13-valent pneumococcal conjugate vaccine (PCV13) was introduced in the United States, PCV13 serotypes caused 44-64% of IPD, depending on age. We constructed a model to forecast IPD trends through 2020 under differing scenarios of serotype replacement and herd effects. **Methods**: IPD cases (isolation of pneumococcus from sterile sites) were identified through 10 Active Bacterial Core surveillance (ABCs) sites from 1998-2009. Vaccine coverage rates were obtained from the National Immunization Survey. We developed and validated a deterministic Poisson model based on rates of IPD during 1998-2009 and used this model to forecast future rates, assuming the serotypes unique to PCV13 will decrease as PCV7 serotypes decreased between 2000 and 2009. The base case model estimated rates with serotype replacement and herd effects similar to those seen with PCV7. In sensitivity analyses, we decreased levels of serotype replacement and herd effects. **Results**: During 1998-2009, ABCs identified 43,507 cases of IPD. In the base case model, overall rates (cases per 100,000) of IPD in children <5 years old decreased from 21.7 in 2009 to 10.2 in 2020. This decrease was driven mainly by a decrease in IPD caused by serotype 19A, which was forecast to decrease from 9.3 in 2009 to 0.28 cases in 2020. Under a scenario with no serotype replacement, overall rates of IPD in children <5 years old would decrease to 8.9 cases per 100,000 by 2020. In the base case scenario, rates of IPD among adults ≥65 years old would decline by 25% from 38.3 to 28.7 by 2020. **Conclusions**: Regardless of potential serotype replacement, our model forecasts a significant decrease in overall IPD in the decade following PCV13 introduction, due mainly to decreases in serotype 19A. These estimates can be used to establish disease reduction goals and to monitor progress toward them.

## Vector-borne Diseases and Climate Change

Tuesday, March 13
12:30 PM – 1:30 PM
Grand Hall

**Board 214. Early Diagnosis of Lyme Carditis Is Important To Prevent Severe Complications**

**Q. Shi**[1], J. Weisenreider[2], J. Chintanaboina[1], C. Christian[1], H. Wu[1], J. Jia[1], L. Thomas-Hemak[1];
[1]The Wright Center for Graduate Medical Education, Scranton, PA, USA, [2]Pike County Family Health Center, Honesdale, PA, Scranton, PA, USA.

**Background:** The incidence of Lyme disease is rapidly increasing with a current incidence of over 400,000 cases per year in USA. Additionally, carditis is becoming an increasingly frequent complication of Lyme disease. Early diagnosis and treatment of Lyme carditis is important to prevent complications and shorten the course of illness and recovery. We report an unusual case of Lyme carditis with literature review. Case report: A 60-year-old African-American male with a history of hypertension and type-2 diabetes mellitus presented with an episode of chest discomfort and light-headedness. Patient admitted that he might have been bitten by ticks recently but denied any history of rash. Home medications included amlodipine, benazepril, glimepiride, and metformin. Physical examination was completely normal except for a heart rate of 48/minute. Electrocardiogram (ECG) showed third-degree AV block with narrow junctional escape rhythm. Lyme Immunoglobulin-M antibody titres were positive. Serum electrolytes were normal. Patient did not receive atropine or pacemaker and was just treated with intravenous ceftriaxone 2 gm daily for 21 days and symptoms resolved completely.
**Discussion:** Lyme disease is caused by Borrelia burgdorferi transmitted by the Ixodes tick. About 4-10% of patients with untreated Lyme disease develop carditis. Cardiac complications include fluctuating degrees of atrioventricular (AV) block, cardiomegaly, or congestive heart failure. The cardinal manifestation of Lyme carditis is conduction system disease, commonly at the AV node. Studies show that the lack of response of most patients to atropine is suggestive of a direct effect of Lyme disease on the AV node, rather than an indirect vagotonic effect. Resolution of AV block has been proposed to be due to regression of inflammation in the context of early initiation of antibiotic therapy. Temporary pacing may be necessary in about 30% of patients. However, prophylactic transvenous pacing is not recommended. **Conclusions:** We suggest that patients presenting with suspected cardiac symptoms in endemic areas should have a screening EKG along with Lyme titers. They should be closely monitored for any changes on the EKG and worsening hemodynamic parameters during the initial phase of the disease.

**Board 215. Post-epidemic Seroprevalence in the Epicentre of a West Nile Virus Outbreak in Central Macedonia, Greece, 2010**

**G.A. Ladbury**[1,2], M. Gavana[3], K. Danis[4], T. Stardeli[3], A. Panos[3], M. Dragasaki[3], S. Giannakopoulos[3], F. Goma[5], M. Keramarou[6,2], M. Moirasgenti[3], Y. Theocharopoulos[4], E. Tsantaki[3], A. Vakfari[3], E. Vouzounerakis[3], D. Papamichail[7], A. Lenglet[8], S. Gewehr[9], A. Benos[3], A. Papa[3], T. Panagiotopoulos[7];
[1]Dutch National Institute for Public Health and the Environment (RIVM), Bilthoven, Netherlands, [2]European Programme for Intervention Epidemiology Training (EPIET), European Centre for Disease Prevention and Control (ECDC), Stockholm, Sweden, [3]Aristotle University of Thessaloniki, Thessaloniki, Greece, [4]Hellenic National Centre for Disease Prevention and Control (KEELPNO), Athens, Greece, [5]Hellenic Centre for Disease Prevention and Control (KEELPNO), Thessaloniki, Greece, [6]Public Health Wales, Cardiff, UK, [7]National School of Public Health (ESDY), Athens, Greece, [8]European Centre for Disease Prevention and Control (ECDC), Stockholm, Sweden, [9]Ecodevelopment, Thessaloniki, Greece.

**Background:** During the summer of 2010, 262 cases including 35 deaths from West Nile virus infection were reported from Central Macedonia, Greece. We conducted a seroprevalence study in the outbreak epicentre to determine post-epidemic seroprevalence and identify risk factors for infection. **Methods:** Sixty clusters of 6 households were randomly selected from the Pella and Imathia districts in Central Macedonia. All household members aged ≥18 years were eligible to participate. Information on individual- and household-level exposures was gathered using standard questionnaires. Sera were tested for WNV-specific IgM and IgG using ELISA. The relationships between exposures and IgG seropositivity were explored using crude prevalence ratios and binomial regression, weighting exposures and outcome by age and town size and adjusting for cluster design. **Results:** Overall, 723 (69.3%) of 1043 individuals participated, of whom 644 provided sera. Fifteen samples were IgM positive (weighted seroprevalence 1.9% [95% CI: 1.0-3.5]); these were all additionally IgG positive. In total, 41 samples were IgG positive (weighted seroprevalence 5.8% [95% CI: 3.8-8.6]), all of which had high titres indicating recent infection. Factors associated with IgG positivity included living in a rural area [adjusted prevalence ratio (aPR)=8.18, [95% CI: 1.14-58.72]; being a housewife [aPR=4.30, 95% CI: 2.18-8.46]; being retired [aPR=2.60, 95% CI: 1.38-4.90]; having water repositories on one's property [aPR=2.67-1.42-5.00]; and agricultural labour [aPR=2.93, CI: 1.34-6.40]. **Conclusions:** This survey demonstrates that WNV transmission in the outbreak epicentre was widespread, particularly in rural areas. The risk groups identified may reflect increased exposure to mosquitoes. General interventions, such as health education and mosquito control, with particular attention to these groups may contribute to the prevention of human WNV infections during future periods of WNV activity in this area.

**Board 216. Impact of Drought on the Spatial Pattern of Transmission of *Schistosoma haematobium* in Coastal Kenya**

**C.H. King**[1], F. Mutuku[2], A.L. Bustinduy[1], P.L. Mungai[1], E.M. Muchiri[3], U. Kitron[2];
[1]Case Western Reserve University, Cleveland, OH, USA, [2]Emory University, Atlanta, GA, USA, [3]Ministry of Public Health and Sanitation, Nairobi, Kenya.

**Background:** Urinary schistosomiasis remains a significant public health problem in sub-Saharan Africa. In Kenya, the coastal strip is hyperendemic for urinary schistosomiasis but with substantial spatial and temporal heterogeneities. Local water use and proximity of snail intermediate host breeding sites typically drive the focal distribution of schistosomiasis. *Schistosoma haematobium* has been studied in the Msambweni area since 1984. Prevalence has remained high (>50%) over the years, despite introduction of alternative water sources (boreholes) and chemotherapy programs targeting school children. Because a major drought between 2001 and 2009 resulted in drying of ponds that were known sources of infection and we detected very few or no snails in ponds that had been infested in the past, we analyzed interval temporal changes in spatial patterns of active *Schistosoma haematobium* infection in different age groups of local residents. **Methods:** After GPS/GIS and parasitological updates of the Mililani Village community, household-level spatial patterns of infection for children of various age groups in 2009 was contrasted with historical data from 2000. Both global (Ripley's weighted K function) and local/focal (Getis G statistic) clustering of infection density were considered. **Results:** The significant local clustering of high- and low-infection levels among school-aged children that occurred in 2000 was absent in 2009. **Conclusions:** We attribute the disappearance of significant clustering around transmission hot spots to a decade-long drought in our study area. This shift in spatial infection pattern means that, in this setting, there is a need to consider the value of community-wide interventions rather than rely on focalized efforts aimed at only a few hot spots. Our findings point to the potential impact of climatic variability on transmission patterns of *S. haematobium*. Such changes need to be considered when implementing urinary schistosomiasis surveillance and control.

**Board 217. Reported *Ehrlichia chaffeensis*, a Cause of Human Ehrlichiosis, in Minnesota, 2011: Emerging Pathogen or Misleading Diagnostic Result?**

**H. Friedlander**, M. Kemperman, D. Neitzel, M. Korinek, K. Smith;
Minnesota Department of Health, St. Paul, MN, USA.

**Background:** Human monocytic ehrlichiosis (HME; *Ehrlichia chaffeensis*) is a tick-borne disease with clinical manifestations similar to human anaplasmosis (HA; *Anaplasma phagocytophilum* [*A. phago.*]). HME is endemic in states south of Minnesota (MN); HA is highly endemic in MN. *Ehrlichia muris*-like (EML) agent was recently documented as a cause of human illness in MN and Wisconsin (WI). Both *A. phago.* and the EML agent might produce cross-reactive antibodies to *E. chaffeensis*. The MN Dept. of Health (MDH) has received increased numbers of *E. chaffeensis* reports in patients with no recent travel. Similar clinical aspects, serologic cross-reactivity, and lack of travel raise suspicion as to the presence of HME in MN. **Methods:** MDH collected laboratory details (assays performed; *A. phago.* titers) on all *E. chaffeensis* antibody reports in 2011. Reports with only IgM findings were not cases. IgG IFA titers for *E. chaffeensis* were compared to those of *A. phago.*; those less than *A. phago.* were potential HA cases; those <4-fold higher or with no or unknown *A. phago*. testing were potential HA/ehrlichiosis-undetermined cases; and those 4-fold higher than *A. phago.* were potential HME cases. We collected clinical details and classified cases per the CSTE surveillance case definition. **Results:** From Jan-Sept 2011, 121 *E. chaffeensis* antibody findings were reported. Twenty-two were IgM positive only with negative or unknown *A. phago*. results (not cases). Of the 99 IgG positive *E. chaffeensis* reports, *A. phago.* tests were performed in 93 (94%) reports, of which 61 (62%) were potential HA cases. Thirty-one (33%) reports were potential HA/ehrlichiosis-undetermined cases due to *A. phago.* titers that were not performed, missing, or <4-fold smaller than *E. chaffeensis*. The remaining 7 (7%) reports were potential HME, of which 5 (5%) were clinically compatible, probable HME cases. **Conclusions:** Over half of MN's *E. chaffeensis* antibody reports in 2011 were potential HA cases due to likely cross-reactivity with *A. phago*. The one-third of reports that were potentially HA/ehrlichiosis-undetermined could be HME, HA, or EML. Identification of only 5 probable HME cases suggests that while endemicity is possible in MN, serologic cross-reactivity with the EML agent cannot be discounted, warranting further investigation into HME and EML in MN.

## Travelers' Health

Tuesday, March 13
12:30 PM – 1:30 PM
Grand Hall

**Board 218. Pre-Travel Rabies Vaccine Use among U.S. Travelers Seen at Global TravEpiNet Clinics**

**S.B. Dolan**[1], E.S. Jentes[1], M.J. Sotir[1], P. Han[1], J. Blanton[1], S.R. Rao[2], R.C. LaRocque[3], E.T. Ryan[3], Global TravEpiNet Consortium;
[1]Centers for Disease Control and Prevention, Atlanta, GA, USA, [2]Department of Biostatistics, University of Massachusetts, Worcester, MA, USA, [3]Massachusetts General Hospital, Boston, MA, USA.

**Background:** Rabies exposure poses a risk to travelers, especially those visiting areas with high rabies endemicity. We described characteristics of travelers, especially those traveling for ≥28 days to rabies high-risk areas, who received rabies vaccine during consultations at Global TravEpiNet Consortium (GTEN) clinics. **Methods:** GTEN clinics use an online tool to document pre-travel consultations of U.S. international travelers. The tool links to CDC's country-specific rabies vaccine recommendations, which are categorized as no recommendation, weak, moderate, and strong. We analyzed data collected from 18 GTEN clinics from January 2009 through January 2011, including traveler characteristics and rabies

vaccination status. **Results:** Of 13,235 GTEN travelers, 406 (3%) received a vaccination at their pre-travel consultation, 226 (2%) reported having been previously immunized against rabies, and 12,603 (95%) who were not previously immunized were not vaccinated during the visit. Those vaccinated during their visit were younger than those not vaccinated (median 28 vs. 36 years, Wilcoxon rank sum p<.01). The most common purposes of travel for vaccinated travelers were leisure (26%), research/education (17%), and nonmedical service work (14%). Of the 13,009 vaccinated travelers without previous immunity, 8,070 (62%) visited a single country with a strong recommendation. Of these, 1675 (21%) traveled for ≥28 days; 832 had itineraries that clinicians determined to not indicate vaccination, vaccination was recommended for 498 who declined it, 145 received vaccination at the visit, and 200 did not receive it for other reasons. For those who declined vaccination, the most common purpose of travel was visiting family or relatives (18%). Of those traveling ≥ 28 days or longer, 63% were visiting rural areas. **Conclusions:** Few GTEN travelers are vaccinated for rabies during their pre-travel consultations, even though almost two-thirds visited a single country with strong recommendation and 21% of these travelers were there for more than one month. Administration of rabies vaccine should be considered for travelers visiting countries with strong rabies vaccine recommendations, especially those with extended travel durations, and those who are visiting friends and relatives or traveling to rural areas.

**Board 219. AFHSC-GEIS DoD Enteric Disease Surveillance Efforts and Vision Forward**

**R.M. Chandrasekera**[1], D. Blazes[1], M. Riddle[2];
[1]Armed Forces Health Surveillance Center, Silver Spring, MD, USA, [2]Naval Medical Research Center, Silver Spring, MD, USA.

**Background:** Enteric disease surveillance is a priority within the US military due to the high risk of gastrointestinal infections within military personnel during deployments as well as the impact on health security among the civilian population. **Methods:** Through a global network of Department of Defense (DoD) laboratories supported by the Armed Forces Health Surveillance Center's Division of Global Emerging Infections Systems (AFHSC-GEIS), enteric infection surveillance is conducted in US military populations, adult travelers, and military and civilian populations of partner nations to better understand circulating pathogens, infection risk, disease burden, and identify potential infection treatment and control measures. **Results:** In FY11, 17 enteric activities were conducted in eight countries around the world. These efforts have led to a better understanding of local pathogens, e.g. identification of the first cases of enterohemorrhagic *E. coli* from civilian sites in Kenya. Surveillance projects have also led to an improved awareness of antibiotic resistance patterns, e.g. preliminary data show a high proportion of *Shigella* isolates resistant to nalidixic acid (83%) and TMP-SXT (100%) in Bhutan and high proportion of *Shigella* isolates demonstrating resistance to sulfa antibiotics (85.3%) in Peru. In the continental US, efforts have concentrated on bolstering norovirus surveillance in US military shipboard and recruit populations to provide enhanced epidemiological and laboratory support during suspected norovirus outbreak investigations. **Conclusions:** Moving forward, the AFHSC partner network aims to incorporate surveillance to better understand the burden of post-infectious sequelae and improve identification and characterization of pathogens. In addition, a data standardization plan will be developed for utilization by partners to make data comparable across regions and ultimately, more meaningful for global public health. The enteric pathogen landscape is constantly changing as the recent 2011 *E.coli* outbreak in Germany demonstrated, and dictates the need for the AFHSC-GEIS partner network to continue conducting timely and relevant global disease surveillance.

**Board 220. Travel-Associated Cases of Dengue Reported to the Centers for Disease Control and Prevention, 2005 through 2010**

**D.F. Arguello**[1], L. Santiago[1], A. Rivera[1], J. Lehman[2], E. Hunsperger[1], J. Muñoz-Jordan[1], K.M. Tomashek[1], H. Margolis[1];

[1]Centers for Disease Control and Prevention, San Juan, PR, USA, [2]Centers for Disease Control and Prevention, Fort Collins, CO, USA.
**Background:** Dengue is one of the leading causes of febrile illness in travelers returning from the tropics. We describe travel-related dengue cases from residents of the 50 states and the District of Columbia (DC) with illness onset between 2005--2010 and use 2010 dengue diagnostic testing to estimate disease burden. **Methods:** Travel-related dengue case data reported to the Centers for Disease Control and Prevention's (CDC) national arbovirus surveillance system (ArboNET) and case data from patients with specimens submitted to the CDC Dengue Branch (DB) but not reported to ArboNET were analyzed. A laboratory-positive case was defined by the presence of anti-dengue virus (DENV) or DENV RNA in a submitted specimen. We used dengue diagnostic testing data for specimens submitted to the 6 of 7 state public health, 2 CDC, and 2 commercial reference laboratories that provide testing to estimate the number of dengue cases in 2010. **Results:** Between 2005--2010, 1,697 laboratory-positive travel-related cases were reported (annual average = 285); mean age was 40 years and 51% were male. Of those with clinical information, 93% (1,267), 4% (62), and 3% (37) were classified as dengue fever (DF), DF with hemorrhage, or dengue hemorrhagic fever or shock syndrome, respectively. The average annual number of hospitalizations and deaths was 113 and 1, respectively. Half of the cases traveled to Dominican Republic, Puerto Rico, India, Mexico or Haiti. Of the 14,899 US specimens tested for dengue in 2010, 91% were tested at commercial laboratories and 9% were tested at state or CDC laboratories. Of these specimens, 23% (3,027) were laboratory-positive (97% with anti-DENV IgM). When the estimated number of dengue cases derived from the laboratory testing data was compared to laboratory-positive dengue cases reported to ArboNet-DB there was a 4-fold degree of underreporting. **Conclusions:** Travel to the tropics continues to place US travelers at risk for dengue and disease burden in this population is underestimated by national surveillance. Persons traveling to the tropics should seek pre-travel consultation, minimize mosquito exposure while traveling, and seek medical attention if fever develops during travel or after return. Improvement in national surveillance reporting is needed to determine risk and impact of dengue among US travelers.

**Board 221. Measles Transmission during Air Travel, United States, December 1, 2008–May 31, 2011**

**K. Nelson**;
Centers for Disease Control and Prevention, St. Paul, MN, USA.
**Background**: The Centers for Disease Control and Prevention's (CDC) Division of Global Migration and Quarantine (DGMQ) and US health departments conduct contact investigations for travelers who were infectious with measles during a flight to identify passengers at risk of exposure and implement public health interventions to mitigate disease spread. We sought to determine if the current CDC measles protocol effectively identifies passengers at most risk of in-flight exposure and infection. **Methods**: We analyzed data from contact investigations initiated by DGMQ from December 2008 through May 2011. Per protocol, passenger-contacts were defined as passengers seated within 2 rows of the index case, and children ≤2 years old seated in the lap of an adult anywhere on the aircraft. Names and contact information of passenger-contacts were obtained from airline flight manifests and distributed to US health departments or foreign public health authorities. Passengers without contact information were considered lost to follow-up. US health departments were asked to report outcomes of their contact investigations to DGMQ. We cross-referenced the National Notifiable Diseases Surveillance System (NNDSS) and CDC's National Center for Immunization and Respiratory Diseases (NCIRD) measles databases with passenger-contact demographic data to identify cases in passenger-contacts that had not been reported to DGMQ, and cross-referenced travel dates of cases in those databases with flight dates of index cases to identify any cases in passengers not identified as flight contacts. **Results**: Our evaluation included 65 index cases on 94 flights. Median flight duration was 7 hours (range: 0.6 − 17 hrs). Of 3078 passenger-contacts, information for 667 (22%) was provided to foreign public health

authorities or was insufficient. Information for 2411 (78%) was provided to US health departments; 7/2411 (0.3%) developed measles within one incubation period of their flights. The median age of secondary cases was 2 years (range: 0.4 - 36 yrs). No additional secondary flight-related cases were identified from NNDSS or NCIRD databases. **Conclusions**: Our evaluation provided evidence of measles transmission aboard aircraft. CDC's protocol is effective for identifying passengers at most risk of in-flight measles exposure and infection.

**Board 222. Epidemiology of International Travel-Associated *Campylobacter* Cases in Maryland, 2004–2009**

**A.M. Palmer**, C. Jung, B.C. Hogan, D. Blythe;
Maryland Department of Health and Mental Hygiene, Baltimore, MD, USA.

**Background:** *Campylobacter* infections cause acute diarrheal illness that can lead to hospitalization or death. It is estimated that *Campylobacter* infections account for nearly 850,000 foodborne illnesses in the United States annually, many of them attributed to domestic poultry. Understanding the impact of campylobacteriosis cases acquired internationally is important to such burden and attribution estimates. In addition, in 2005, the US Food and Drug Administration implemented a ban on fluorquinolone (FQ) use in poultry in an effort to reduce the number of human infections with FQ-resistant strains of *Campylobacter*; international travelers may still be exposed because these antibiotics are still used in many other countries. **Methods:** Travel history was collected for all lab-confirmed cases of *Campylobacter* in Maryland, which participates in the Foodborne Diseases Active Surveillance Network (FoodNet). Every viable *Campylobacter* isolate received at the state laboratory was forwarded to the National Antimicrobial Resistance Monitoring System (NARMS) to determine antimicrobial susceptibility. Data from the FoodNet and NARMS systems were linked for analysis. **Results:** During the 6 year study period, 2396 *Campylobacter* cases were reported to FoodNet by Maryland. Travel history was collected for 2036 (85%) cases of which 460 (23%) of the cases traveled internationally. Domestically-acquired *Campylobacter* cases were more often hospitalized (21.8% vs 6.1%). While age distribution and sex were similar between groups, 19.5% of White, 8.2% of African American, and 37.1% of Asian *Campylobacter* cases with known history traveled internationally. Of the 61 NARMS-linked cases, international travelers had a greater proportion of antibiotic resistant *Campylobacter* infections than did non-travelers (54.1% vs. 16.2% for nalidixic acid, and 55.7% vs. 15.5% for ciprofloxacin). **Conclusions:** International travel plays a major role in campylobacteriosis in Maryland and accounts for a high proportion of cases. Such cases differ from domestic cases many regards, notably resistance to antibiotics, including FQs. Recognizing these differences is key to both medical treatment decisions and understanding burden and attribution estimates of US campylobacteriosis.

## J1. Vectorborne Diseases and Climate Change

Tuesday, March 13
3:15 PM – 4:45 PM
Centennial I

**West Nile Virus in Europe During 2010: Is the Epidemiology of the Disease Changing?**

**A. Lenglet**[1], K. Danis[2], A. Papa[3], A. Sirbu[4], K. Kristalovics[5], M. Sierra[6], E. Depoortere[1], W. Van Bortel[1], H. Zeller[1], D. Coulombier[1];
[1]European Centre for Disease Prevention and Control (ECDC), Solna, Sweden, [2]Hellenic Center for Disease Control and Prevention (KEELPNO), Athens, Greece, [3]National Reference Centre for Arboviruses, Aristotle University of Thessaloniki, Thessaloniki, Greece, [4]Romanian National Institute of Public Health,

Bucharest, Romania, [5]National Centre for Epidemiology, Budapest, Hungary, [6]Ministry of Health, Madrid, Spain.
**Background:** Outbreaks of West Nile virus (WNV) infection in humans in European Union (EU) countries are rare, despite its circulation in this region since the 1950s and a large urban outbreak in humans in 1996 in Romania. In August 2010, Greece and Romania reported unusually high numbers of human cases of WNV infection. We reviewed the available epidemiological and virological evidence in the EU during the 2010 transmission season in order to investigate if the epidemiology of WNV is changing in this region and to strengthen preparedness accordingly. **Methods:** EU countries at-risk-for or with known WNV circulation provided epidemiological data on probable and confirmed human cases of WNV infection reported during 2010 according to the EU case definition. In addition, information was provided on the circulating viruses and on indicators from veterinary and entomological surveillance systems. **Results:** In total, 344 probable and confirmed autochthonous human cases of WNV infection were reported from Greece (262), Hungary (17), Romania (57), Italy (6) and Spain (2) between July-December 2010. Lineage 1 was identified in Italy (humans) and in Spain (horses) and lineage 2 was identified in Greece (humans and mosquitoes) and in Romania (humans). Person >50 years of age were the most affected age group in all countries. Infections in horses provided early warning for viral circulation only in Spain. Indicators from bird and mosquito surveillance systems provided information regarding potential mosquito vectors and wild bird habitats, facilitating the detection and sequencing of circulating WNV strains. **Conclusions:** The WNV season in 2010 in the EU was different compared to previous years with: increased areas reporting WNV circulation in humans, Greece experiencing an outbreak in humans for the first time and lineage 2 virus emerging as able to infect humans. These results suggest that the epidemiology of the disease is changing. Investigations are needed to understand the potential impact of these findings in the EU, particularly on blood and tissue safety and the clinical presentation of lineage 2 viral infections. Preliminary 2011 data indicate that WNV activity is spreading and transmission is established in the EU and might continue to occur in the future.

**Powassan Virus, an Under-Recognized Cause of Severe Tickborne Disease: Heightened Surveillance and Recognition in Minnesota, 2008–2011**

**M. Kemperman**, D. Neitzel, J. Palm, L. Gongping, K. Kathryn, D. Boxrud, A. DeVries, C. Lees, R. Lynfield, K. Smith;
Minnesota Department of Health, St. Paul, MN, USA.
**Background:** Powassan virus (POWV) is a flavivirus transmitted by *Ixodes scapularis* (lineage II POWV) and other *Ixodes* ticks (lineage I). POWV is related to West Nile virus (WNV) and can cause neuroinvasive disease. From 1999-2009, 25 US POW cases were reported. The Minnesota (MN) Dept. of Health (MDH) identified MN's first 3 cases in 2008-09 and lineage II POWV in northern MN *I. scapularis* in 2009. **Methods:** In 2010-11, MDH enhanced POW surveillance. Spinal fluid and serum were tested by pan-Flavivirus PCR and by IgM EIA for POWV and WNV, with PRNT by CDC. MDH tested *I. scapularis* for POWV by PCR. We used UPGMA global cluster analysis to compare NS5 gene sequences of 2008-11 clinical and northern tick specimens to published sequences. **Results:** MDH confirmed 12 MN POW cases in 2010-11. Our 15 cases since 2008 were identified via the Unexplained Critical Illness program (4), provider requests for POWV or WNV tests (9), active lab surveillance (1), and re-testing WNV-positive specimens from commercial labs (1). Lineage II POWV RNA was identified in 3 (20%) cases. All cases were POWV IgM and PRNT positive except 1 PCR-positive immunocompromised case; 3 (20%) also had suggestive WNV IgM results. Thirteen (87%) cases were neuroinvasive; 2 (13%) were only febrile. Median age was 52 years (range, 3 months—70 years); 12 (80%) were male. Onsets occurred May-Oct, peaking in June-July (10 [67%] cases). Cases lived in 12 counties; all were exposed in northern MN where *I. scapularis* are endemic, 10 (67%) near home. Of 112 *I. scapularis* from SE MN, 4 (4%) were POWV PCR-

positive. NS5 gene sequences from the 3 PCR-positive cases clustered closely together in the lineage II clade; the next closest lineage II branch included WI and CO strains. Northern MN tick sequences clustered most closely to WI lineage II strains. **Conclusions:** With enhanced surveillance, MN POW case numbers increased markedly in 2010-11. Molecular findings and spatial distribution of cases and ticks indicate risk for lineage II POWV from *I. scapularis* in northern and SE MN. Only 3 US labs offer POWV testing on human clinical specimens, and provider awareness is low. Some cases might be attributed to WNV because of serologic cross-reactivity. Public health officials should communicate POWV risk to the public and explore ways to increase provider awareness and POWV testing access.

### Sequential Dengue Virus Infections—Puerto Rico, 2005–2010

**T.M. Sharp**, J.L. Munoz-Jordan, E. Hunsperger, O. Padro, H.S. Margolis, K.M. Tomashek;
Centers for Disease Control and Prevention, San Juan, PR, USA.

**Background:** Dengue, a potentially fatal febrile illness caused by four mosquito-transmitted dengue viruses (DENV-1-4), is endemic in Puerto Rico. Early studies by Sabin demonstrated that inter-serotype, or heterotypic, immunity can protect against symptomatic illness for ~2 months, and serotype-specific, or homotypic, immunity was thought to be lifelong. To confirm the duration of protective homo- and heterotypic immunity following a previous episode of dengue, we utilized patient and laboratory data gathered through a passive dengue surveillance system from 2005-2010. **Methods:** Acute dengue was defined as DENV detected by serotype-specific reverse transcriptase polymerase chain reaction (RT-PCR) in a serum specimen collected ≤ 5 days after symptom onset, and recent DENV infection was defined as anti-DENV IgM antibody present in a serum specimen collected ≥ 5 days after symptom onset. **Results:** We identified 53,633 individuals reported as a suspected dengue case from 2005-2010, of whom 949 (3.7%) were reported on more than one occasion. Of these individuals, 28 (3.0%) had acute dengue or recent DENV infection on two distinct occasions. Median time between infections was 452 days (range: 37 - 1,935), and median age at first disease episode was 13 years (range: 0.5 - 73); 23 (82%) individuals were male. Eleven of 28 individuals tested positive by RT-PCR on both occasions, 9 had anti-DENV IgM followed by a positive RT-PCR result, 5 were RT-PCR positive followed by anti-DENV IgM, and 3 were anti-DENV IgM positive on both occasions. Two DENV-1/4 co-infections were identified, as well as one individual who was twice infected with DENV-2 16 months apart. Efforts are currently underway to characterize these 28 individuals' antibody repertoire following first identified DENV infection and the contribution of cell-mediated immunity to subsequent infection. **Conclusions:** Overall, these observations suggest that heterotypic DENV immunity may be as short lived as 37 days, and that homotypic immunity may not be lifelong.

### Deep Sequencing Characterization of Whole Microbial Communities: A Critical New Tool for Vector-borne Disease Ecology

**A.J. Williams**[1,2], A. Frace[1], T. Mixson-Hayden[1], G.A. Dasch[1];
[1]Centers for Disease Control and Prevention, Atlanta, GA, USA, [2]Emory University, Atlanta, GA, USA.

**Background:** Second generation sequencing platforms now provide a previously unimaginable depth of coverage of complex microbial communities at a fraction of the cost and effort necessary for the largest first generation studies. As third generation platforms approach maturity, cost continues to decline and throughput to increase. For the first time methodologies are being developed that allow the study of pathogenic microbes in the context in which they evolve: a complex community of microbes interacting with each other and their eukaryotic host(s). This is particularly important for vector-borne diseases because an understanding of microbial community dynamics and their influence on pathogen distribution and abundance can provide powerful tools for risk prediction and control. **Methods:** The bacterial communities associated with the lone star tick were assessed. *Amblyomma americanum* is the most abundant and aggressive human-biting tick in the southeastern United States and a vector of

multiple known and suspected vertebrate pathogens. DNA was extracted from individual nymphal and adult ticks and amplified with barcoded primers targeting variable regions 3-5 of the bacterial 16S rRNA gene and sequenced on the Roche 454 Titanium platform. Sequence quality control processing and community analysis was performed using the software mothur. **Results:** An average of approximately 2000 high quality sequence reads were obtained from 200 individual tick DNAs on one plate. Community composition was highly variable, but nymphs and adult male *A. americanum* bacterial communities were more similar and generally dominated by one or two genera of bacteria, while adult females contained communities that were more evenly distributed across four or more genera. Both expected and novel taxa were detected. **Conclusions:** Defining the composition of a vector's microbial community and the range of natural variation between life stages is the first step toward a comprehensive understanding of the community's dynamics. The influence of environment, blood meal source, and life stage on the prevalence and diversity of the bacterial community of *Amblyomma americanum* are being investigated.

### *Bartonella* Species in Bats from Thailand

**M.Y. Kosoy**[1], Y. Bai[1], S. Boonmar[2], P. Sawatwong[2], P. Jorakate[2], L. Peruski[2], H. Baggett[2], B. Lumlertdacha[3], R. Franka[4], C. Rupprecht[4], S. Maloney[2];
[1]Centers for Disease Control and Prevention, Fort Collins, CO, USA, [2]International Emerging Infections Program, Thailand MOPH-US Centers for Disease Control Collaboration, Nonthaburi, Thailand, [3]Queen Saovabha Memorial Institute Thai Red Cross Society, Bangkok, Thailand, [4]Centers for Disease Control and Prevention, Atlanta, GA, USA.
**Background:** Bats are known to be associated with a variety of emerging viruses; however, bacterial species have been rarely reported in bats. Recently, new bacteria belonging to the genus *Bartonella* were described in bats from Guatemala, Kenya, and the UK. The objectives of this study were to: 1) estimate prevalence of *Bartonella* infections among diverse chiropteran species in Thailand, 2) isolate and characterize novel *Bartonella* spp., and 3) evaluate host-specific relations between *Bartonella* spp. and bats in Southeast Asia. **Methods:** Samples were collected from bats in 4 Thai provinces: Chiang Rai (north), Kamphaeng Phet (west), Khon Kaen (north-east), and Sa Kaeo (east). Whole blood from 93 bats of 7 species was cultured by inoculating blood-enriched agar. *Bartonella* isolates were verified by PCR amplification and sequencing of a specific region in the citrate synthase gene (*glt*A). **Results:** *Bartonella* bacteria were cultured from blood of 34 (36.6%) of 93 bats distributed among all four provinces and representing five bat species: 17 *Tadarida plicata,* 12 *Hipposideros larvatus,* 3 *H. armiger,* 1 *H. fulvus*, and 1 *Taphozous melanopogon.* Phylogenetically, *Bartonella* isolates clustered by host bat species. All isolates from the *Tadarida* bats clustered in a separate monophyletic group, while the *Hipposideros* bats harbored strains clustered around 3 groups of *Bartonella*. All 33 *Bartonella gltA* sequences obtained from *Tadarida* and *Hipposideros* bats were very distinct from all sequences previously described in bats and other animals (<94% *glt*A identity), but 1 isolate obtained from *T. melanopogon* was relatively close to a *Bartonella* strain identified in *Coleura afra* bats from Kenya (96% *gltA* identity). **Conclusions:** The high prevalence of *Bartonella* infection in Thai bats suggests that bats may be natural reservoirs in maintaining circulation of *Bartonella* species in nature. Our findings highlight the need to study whether the bat-originated *Bartonella* species are responsible for illnesses in humans and domestic animals in Thailand and Southeast Asia.

### Lyme and Other Tickborne Disease Prevention Study

**A. Kay**[1], A. Hinckley[1], J. Meek[2], J. Ray[2], S. Niesobecki[2], K. Feldman[3], E. Jones[3], B. Backenson[4], W. Miranda[4], P. Mead[1];
[1]Centers for Disease Control and Prevention, Fort Collins, CO, USA, [2]Connecticut Emerging Infections Program (Yale University and Connecticut Department of Health), New Haven, CT, USA, [3]Maryland

Department of Health and Mental Hygiene, Baltimore, MD, USA, [4]New York State Department of Health, Albany, NY, USA.
**Background:** In the northeastern U.S., tickborne diseases (TBDs) are a major public health concern and identifying effective prevention measures has been challenging. Exposure to infected ticks is highest in the peridomestic environment. Residential use of pesticides to control ticks is widespread in certain areas. However, the efficacy of residential pesticides to prevent human TBDs is unknown. Through a TickNET collaboration with CDC and Emerging Infections Programs in Connecticut (CT), Maryland (MD), and New York (NY) we sought to evaluate the efficacy of a single, targeted, residential acaricide (tick pesticide) application to prevent TBDs. **Methods:** We conducted a randomized, blinded, placebo-controlled trial in select areas of CT, MD, and NY. Participants were consenting adults from freestanding houses with ≥2 inhabitants and a yard. An enrollment survey was administered to measure select risk factors for TBDs and demographics. Households were randomly assigned to receive a single, targeted, application of commercially available acaricide or placebo on their yard in May or June. Tick drags were conducted 2-3 weeks post-treatment on a 10% sample to verify treatment efficacy. Surveys regarding numbers of ticks attached and crawling were administered at 1, 2, 3, and 4 months post-treatment. A final survey to capture self-reported TBDs was administered at 5 months. Reports were validated by medical record review. **Results:** More than 4500 households expressed interest in the study following targeted recruitment mailings; 1534 were enrolled. Properties ranged from 1/2 to 5 acres in size and 1335 (87%) had tick habitat. Post-treatment tick drags indicated acaricide was effective at controlling ticks in CT and MD. The number of ticks reported by participants was similar between the acaricide and placebo groups at 1, 2, and 3 months post-treatment. Participant reports of TBDs are pending completion of the final survey and record review. **Conclusions:** The overwhelming, positive response by the community indicates an unmet need for effective TBD prevention. Demonstration of efficacy is critical to gaining generalized acceptance of acaricide use and can promote further research towards optimizing application methodology and exploration of natural alternatives.

## J2. Policy Implications and Infectious Diseases

Tuesday, March 13
3:15 PM – 4:45 PM
Centennial II

### Burden of Bacteremic Melioidosis in Eastern and Northeastern Thailand

**S. Bhengsri**[1,2], H.C. Baggett[1], S. Thamthitiwat[1], P. Jorakate[1], A. Kaewpan[1], S. Naorat[1], P. Prapasiri[1], K. Tanwisaid[3], S. Chantra[4], L.F. Peruski[1], S.A. Maloney[1];
[1]International Emerging Infections Program, Thailand MOPH–US Centers for Disease Control and Prevention Collaboration, Nonthaburi, Thailand, [2]Department of Tropical Hygiene, Faculty of Tropical Medicine, Mahidol University, Bangkok, Thailand, [3]Nakhon Phanom Hospital, Nakhon Phanom, Thailand, [4]Sa Kaeo Crown Price Hospital, Sa Kaeo, Thailand.
**Background:** *Burkholderia pseudomallei*, causative agent of melioidosis, is highly endemic in Northeastern Thailand. However, population-based burden estimates are limited, as are reports from other areas of Thailand. We describe the burden of bacteremic melioidosis in the Eastern province of Sa Kaeo (SK) and the Northeastern province of Nakhon Phanom (NP) during 2006-2010. **Methods:** Automated blood cultures were implemented in both provinces as part of active surveillance for bloodstream infections in all hospitals. Blood cultures were collected as clinically indicated. Clinical characteristics and in-hospital outcomes were abstracted from medical records of hospitalized patients with *B. pseudomallei* isolated from blood. Patients discharged in moribund condition were categorized

as fatal cases. **Results:** *B. pseudomallei* was identified in 14% of patients with pathogens identified (125/2,064 [6%] patients in SK and 480/2,281 [21%] patients in NP). The average annual incidence of bacteremic melioidosis in SK and NP per 100,000 persons was 4.6 (95% CI = 3.9-5.5) and 12.9 (95% CI = 11.9-14.2), respectively. Incidence peaked among those aged 50-69 years in both provinces. Among cases with known outcomes, 34% (122/364) were fatal, 41% (27/66) in SK and 95/203 (32%) in NP (p=0.2). Population mortality rates per 100,000 persons were 1.7 (95% CI = 1.1-2.5) in SK and 4.3 (95%CI = 3.5-5.2) in NP. Of 324 cases with records reviewed, the median age was 52 years (range 2-85), 57% were male and 64% were rice farmers. Fifty-four percent had diabetes, 16% had renal disease and 31% had no apparent underlying disease. Patients with fatal outcome were more likely than those with non-fatal disease to have pneumonia (46% vs. 15%, p<0.01), elevated bilirubin (57% vs. 32%, p<0.01), and elevated creatinine (82% vs.54%, p<0.01), but less likely to have diabetes (44% vs. 60%, p=0.01. Case fatality did not differ by sex or age group. **Conclusions:** Melioidosis burden is substantial, including in Eastern Thailand, where previous reports were rare. Clinicians throughout Thailand should be aware of disease frequency, predictors of severity, and treatment strategies.

**Clinical and Epidemiology Features in Patients with *Streptococcus suis* Infection in Nakhon Phanom Province, Thailand, 2005–2011**

**P. Prapasiri**[1], D. Ditsungneon[2], S.J. Olsen[1,3], H.C. Baggett[1,3], B. Pilalam[2], S. Singhasri[2], S. Chawalchitiporn[2], S. Dejsirilerd[4], K. Sornwong[2], T. Simali[2], K. Suphawat[4], P. Areerat[5];
[1]International Emerging Infections Program, Thailand (Centers for Disease Control and Prevention/IEIP), Nonthaburi, Thailand, [2]International Emerging Infections Program, Nakhon Phanom Office, Nakhon Phanom, Thailand, [3]Centers for Disease Control and Prevention, Atlanta, Georgia, Atlanta, GA, USA, [4]National Institute of Health, Department of Medical Sciences, Nonthaburi, Thailand, Nonthaburi, Thailand, [5]Nakhon Phanom Provincial Health Office, Nakhon Phanom, Thailand.

**Background:** *Streptococcus suis* is a zoonotic pathogen that infects pigs and causes septicemia and meningitis in humans. The disease in humans is severe and can lead to hearing loss and death. We describe clinical features and risk factors in patients infected with *S. suis* in Northeastern Thailand. **Methods:** A case was defined as a blood culture positive for *S. suis* in a person hospitalized in Nakhon Phanom (population 701,041) during 2006 to 2011.We investigated all cases using medical record reviews and in-depth interviews. **Results:** We identified 20 confirmed *S. suis* cases. Nine cases occurred in clusters, 5 in April 2010 and 4 in June 2011.The median age was 52 years (range 29-69); 15(75%) were male. Clinical features included fever in 18 patients (90%), myalgia in 13 (65%), headache in 11 (55%), nausea and/or vomiting in 11 (55%), difficulty breathing in 10 (50%), stiff neck in 9 (45%), altered consciousness in 9 (45%), poor balance in 8 (40%), hearing impairment in 6 (30%), and diarrhea in 4 (20%). Seven (35%) patients were diagnosed with meningitis at admission. Predisposing factors included 12 (60%) patients (all males) with a history of regular (4) or heavy (8) alcohol use; two had hypertension and one diabetes. Average hospital stay was 9 days. All patients were treated with ceftriaxone or penicillin. Irreversible deafness occurred in 3 patients (15%), all of whom were heavy alcohol consumers. No deaths occurred in hospital or within 30 days of discharge. All patients reported pig or pork exposure in the 7 days before onset; 7 (35%) slaughtered pigs, 8 (40%) consumed raw or undercooked pork, 6 (30%) consumed raw pig blood. Incubation from presumed exposure to symptom onset ranged from 1-7 days. All patients reported having no knowledge of *S. suis* infection or risk factors. **Conclusions:** Clinicians should consider *S. suis* in patients hospitalized in northeastern Thailand where high-risk pig exposures are common. Patients admitted with septicemia or meningitis should be asked about exposure to pigs and undercooked pork, and blood cultures positive for *Streptococci* should be evaluated for *S. suis*. Efforts to increase awareness of *S. suis* may help improve adherence to national recommendation to avoid consumption of raw or uncooked pork products.

**Changing Trends in Six Decades of Infectious Disease Mortality in Thailand**

**S. Aungkulanon**[1], M. McCarron[2], J. Lertiendumrong[1], S.J. Olsen[3,2], K. Bundhamcharoen[1];
[1]International Health Policy Program, Nonthaburi, Thailand, [2]Centers for Disease Control and Prevention, Atlanta, GA, USA, [3]Thailand Ministry of Public Health - United States Centers for Disease Control and Prevention Collaboration, Nonthaburi, Thailand.

**Introduction**: Thailand is a home to many tropical diseases and has a high burden of two infectious diseases of great global health importance, HIV/AIDS and tuberculosis. It has also had a high burden of respiratory disease. We reviewed trends in mortality due to infectious diseases to better define current diseases of concern. **Method**: We analyzed morality trends between 1958 and 2009 using data from the series of the Report of Public Health Statistic published by the Ministry of Public Health of Thailand. To standardize the coding, we mapped all ICD codes to version 9. Census and estimated mid-year population data were used to calculate rates. **Result:** From 1958 to the mid 1990s, the infectious disease mortality rate in Thailand declined 5-fold from 163.4 per 100,000 in 1958 to 29.5 per 100,000 in 1997; on average, the rate decreased by 3.21 per 100,000 (95% CI: 2.78-3.65) per year largely due to declines in malaria, tuberculosis, pneumonia and gastrointestinal coded deaths. From 1998 to 2003, the mortality rate rebounded to a peak of 70 per 100,000 in 2003; this increase coincided with increases in mortality from AIDS (2.23/100,000 in 1994 to 25.83 in 2003), the re-emergence of tuberculosis (5.91/100,000 in 1994 to 10.97 in 2003), and increases in pneumonia deaths (9.02/100,000 in 1998 to 23.68 in 2004). From 2004-2009, the rate declined to 41 per 100,000, a period when AIDS deaths decreased from 18.35/100,000 to 6.38. **Conclusions:** The emergence of AIDS and the increase in tuberculosis and pneumonia mortality in the late 20$^{th}$ century dramatically interrupted the epidemiological transition in Thailand. Although an intensive national prevention campaign during the mid-1990s resulted in a decrease in HIV/AIDS mortality after 2004, this decline remains unstable. The recent increase of infectious mortality emphasizes the threat posed by behavioral changes and microbes and demands investment in preparedness.

**Leptospirosis Risk Reduction in Western India: Making an Impact through Intersectoral Collaboration**

**N. Rustagi**, U.V. Rana, A. Kumar;
National Centre of Disease Control, Delhi, India.

**Background:** Leptospirosis is endemic in coastal areas of India. In 2011, a surge in cases of leptospirosis were reported in Southern Gujarat and Maharashtra following heavy rains in August. A team from the National Centre for Disease Control investigated the situation in order to understand the prevailing situation responsible for this surge in affected districts, assess eco-epidemiological factors causing increased cases and identify stakeholder's and their roles in containing and reducing disease burden. **Methods:** The team visited local healthcare facilities at all levels where discussions were held with health administrators, clinicians and laboratory staff. We also visited offices of the District administration, Agriculture and Animal Husbandry Departments and community leaders. State historical surveillance records were reviewed to compare annual cases, deaths and CFR. Houses of cases and deceased were visited to assess community based surveillance, point of care and control measures. **Results:** As of 4 Oct 2011 both Gujarat (Cases - 868; Deaths- 162; CFR- 18.7%) and Maharashtra (Cases - 531 ; Deaths- 22 ; CFR- 4.1%) reported a large number of cases of leptospirosis. When compared with historical records in Gujarat these numbers demonstrated a marked increase in cases, deaths and number of affected villages in 2011. In Maharashtra there was a rise in cases in 2011, but no change in deaths or number of affected villages. Investigation revealed initiation of chemoprophylaxis, and implementation of a rapid response team by Dept of Health as well as anti rodent measures by the Dept of Agriculture and animal hygiene guidelines by Dept of Animal Husbandry in Maharashtra State. **Conclusions:** Surveillance data demonstrates that districts in Western India are endemic for leptospirosis. These districts share similar eco- epidemiological characteristics and are at high risk of

disease outbreaks during monsoon season. During 2011, heavy rainfall correlated well with the surge in cases of leptospirosis in both states. In Maharashtra, though an increase in cases was noted, there was no correlating increase in geographic spread or mortality. This suggests that multisectorial policy decisions and prevention and control strategies were effective in reducing impact of leptospirosis.

**A Randomized, Community Trial on Effect of Handwashing**

**J. Shen**;
Chinese FETP, Beijing, China.

**Background:** In observational studies, better handwashing practices have been associated with reduced risk of communicable diseases transmitted through personal contact. However, whether this association is due to incomplete control of confounding by socioeconomic status is still debated. We conducted a randomized, controlled community trial to evaluate whether handwashing can reduce the incidences of Hand, Foot and Mouth Disease (HFMD), and upper respiratory and gastrointestinal syndromes. **Methods:** We selected 64 villages with mid-level incidence rates of HFMD during 2009-2010 in Handan Prefecture in northeastern China's Hebei Province. We randomized the villages into intervention and control groups, and implemented a comprehensive handwashing intervention program in families with children aged 6-35 months in the 32 intervention villages, but not in the 32 control villages. Trained village doctors conducted semi-monthly household visits to record incidences of HFMD, influenza like illnesses (ILI), and diarrheal illnesses. At the mid-point and end of the trial, we randomly selected 200 children in each group to collect specimens of hand swabs and tested for total coliforms. **Results:** 3757 children in intervention villages and 2719 in control villages participated in this trial. We observed significantly lower incidence rates among children in intervention villages than in control villages for ILI (3.7% vs. 13%; RR=0.28, 95% CI: 0.23-0.34), diarrheal syndrome (1.3% vs. 7.2%; RR=0.17, 95% CI: 0.13-0.24); and HFMD (0.30% vs. 0.80%; RR=0.36, 95% CI: 0.18-0.74). We also observed significantly lower positivity rates of total coliforms among children in intervention villages than in control villages at the mid-point (1.9% vs. 9.2%; RR=0.21, 95% CI: 0.070-0.60) and end of the trial (3.4% vs. 9.0%; RR=0.38, 95% CI: 0.16-0.94). **Conclusions:** Community-based handwashing interventions can improve hand hygiene and reduce the risks for HFMD, ILI and diarrhea symptom. We recommend that handwashing be adopted as a comprehensive strategy for preventing communicable diseases transmitted through personal contact.

**A Public Health Regulatory Agency's Approach to the Primary and Secondary Prevention of Foodborne Illness, Food Safety and Inspection Service, United States, 1996–2011**

**K.G. Holt**[1], V.T. Chen[2];
[1]USDA, FSIS, Atlanta, GA, USA, [2]USDA, FSIS, Washington, DC, USA.

**Background:** The Food Safety and Inspection Service (FSIS) is the public health regulatory agency that ensures that the U.S. commercial supply of meat, poultry, and egg products is safe, wholesome, and correctly labeled and packaged. In 1996, FSIS shifted to the primary prevention of foodborne illness with its publication of the Pathogen Reduction/Hazard Analysis and Critical Control Point (HACCP) Systems Final Rule. FSIS strengthened secondary prevention through enhancements to food safety outreach to consumers and foodborne illness investigations. **Methods:** We compared FSIS microbiological testing data for *Salmonella* spp. on raw products and *Listeria monocytogenes* (Lm) in ready-to-eat (RTE) foods between the pre- and post-HACCP implementation periods. We analyzed FSIS primary prevention efforts to address the higher prevalence of Lm for in-store packaged food and fit them to the Institute of Medicine's model public health agency cycle. Key enhancements to consumer outreach and foodborne illness investigations and challenges to address emerging infectious disease issues were identified. **Results:** FSIS estimated a 20% prevalence of *Salmonella* spp. on chickens from a 1994 - 1995 study in contrast to 7.5% from a similar 2007 - 2008 study. FSIS testing for *Lm* in RTE products revealed 4.60% of

samples were positive for Lm in 1990 in contrast to 0.28% in 2010. Key enhancements to consumer outreach included the use of social media and multilingual releases, such as American Sign Language videocasts. Illness investigations shifted from the pre-HACCP use of a few dedicated staff to the current, multi-disciplinary, across agency approach. FSIS recent efforts to prevent non-O157 Shiga toxin-producing *Escherichia coli* (STEC) illnesses associated with beef and current assessment of multidrug resistant pathogens revealed the challenges FSIS faces in primary prevention efforts where less robust data exist. **Conclusions:** This study illustrates that FSIS approaches to protect public health can be described as primary and secondary prevention of foodborne illness. FSIS faces challenges in primary prevention efforts where less robust data for risk analysis exist. FSIS efforts to address non-O157 STEC illustrate that primary prevention efforts based on scientific knowledge can be taken to avert a large foodborne outbreak.

## J3. Influenza Preparedness: Lessons Learned

Tuesday, March 13
3:15 PM – 4:45 PM
Centennial III

**Lessons Learned from Delivery and Deployment of Pandemic A (H1N1) 2009 Vaccine in the World Health Organization European Region**

**P. Jorgensen**;
World Health Organization, Copenhagen, Denmark.

**Background:** In the event of an influenza pandemic, rapid development and distribution of effective and safe vaccines against a new virus is critical to reduce severe disease and death, and the pressure on health care systems. The 2009 pandemic was the first influenza pandemic where vaccines were potentially available in the entire WHO European Region, covering 53 European and Central Asian countries. We conducted a survey to assess timing of vaccine delivery and vaccination policies and coverage. **Methods:** In 2010, a standard online questionnaire was distributed to 53 member states. Data collected included information on population groups recommended for vaccination, date of vaccine delivery, vaccine doses received and distributed by population group, and challenges for vaccine deployment. Timing of vaccine receipt was compared with the peak of A(H1N1) transmission in each country, defined as the week with the highest outpatient consultation rates for respiratory disease obtained from the WHO influenza surveillance network (EuroFlu). **Results:** Of 51 countries responding, 42 (82%) implemented A(H1N1) vaccination. All countries recommended vaccination for health care workers (HCW), 98% for persons with medical conditions (UMC), 90% for pregnant, and 60% for the entire population. Reported coverage for HCW ranged from 1%-95% (n=21), 3%-72% (n=9) for UMC, 0%-58% (n=15) for pregnant, and 0.4%-59% (n=28) for the entire population. Timing of vaccine receipt relative to epidemic peak ranged from 2 months before to 1 year after with 41% of countries receiving vaccine ≥2 weeks before the peak, 22% between 2 weeks before and 2 weeks after the peak, and 37% 2 weeks or longer post-peak transmission (n=41). **Conclusions:** Vaccination recommendations were similar in the Region, but coverage varied greatly and was low overall, also in primary target groups. Barriers to vaccine uptake included concern on vaccine safety, a relatively mild pandemic, and lack of campaigns directed at risk groups. However, many countries, mainly those dependent on donations, had low coverage due to insufficient vaccines. The survey highlights important weaknesses in both health communication and vaccine logistics and supply, and calls for increasing vaccine production and improving vaccine distribution and uptake for strengthening preparedness.

### Antiviral Use among Hospitalized Influenza Patients in Minnesota during the 2010–2011 Influenza Season

**A.S. DeVries**, C. Morin, S. Swanson, M. Ayers-Johnson, R. Lynfield;
Minnesota Department of Health, St. Paul, MN, USA.

**Background:** Antiviral treatment decreases influenza morbidity and mortality and is recommended for all hospitalized patients. It is also recommended for high risk groups including those aged <2 or ≥65 years, pregnant or post-partum women, persons who are American Indian/Alaska Natives (AIAN), persons who are morbidly obese, long-term care residents, persons with chronic health conditions, and persons with severe disease. Antiviral agents are often not prescribed despite data supporting their benefit. **Methods:** Active, population-based surveillance for influenza hospitalizations in the 7-county Minneapolis-St. Paul metropolitan area (MSP) was conducted from Oct.2010-April 2011 as part of FluSurv-Net. A case was defined as an MSP resident hospitalized in MSP with lab-confirmed influenza. Medical charts were reviewed for information on underlying medical conditions, antiviral treatment, and clinical outcome. **Results:** There were 446 hospitalized influenza cases identified. 291 (65%) cases received an antiviral; 288 (99%) oseltamivir. Four (1%) received treatment prior to admission and 244 (87%) received treatment on, or 1 day after, admission. Antiviral receipt varied by age group with low rates among those <1 year (46%) and <2 years (51%) of age, and highest among those ≥65 years (73%). A total of 333 (75%) cases had a known comorbidity, of which, 223 (67%) received an antiviral. There were no differences in the proportion treated with an antiviral based on obese vs. non-obese (63% vs. 66%; p=0.7), pregnant vs. non-pregnant (44% vs. 66%; p=0.1), AIAN vs non-AIAN (86% vs. 65%; p=0.4), and long-term care residents vs. non-residents (72% vs. 64%; p=0.3). Five (2%) deaths occurred among cases who received an antiviral compared to 7 deaths (5%) among those that did not receive an antiviral (p=0.1). **Conclusions:** Antivirals are indicated for any patient hospitalized with suspected influenza. Many high risk patients for whom antivirals were recommended based on underlying health conditions in addition to hospitalization were untreated. Among those treated, most received an antiviral within the first 2 days of admission. Hospital-based treatment protocols and healthcare provider education may increase antiviral treatment rates among hospitalized influenza patients, particularly among children <2 years of age.

### Influenza Disease Burden in a Rural Community of North India during and after the 2009 H1N1 Pandemic, 2009–2011

**S.K. Rai**[1], V. Gupta[2], F. Dawood[3], R. Wigh[2], A. Krishnan[1], A. Mishra[4], K. Lafond[5], J. Mott[5], R. Lal[5], S. Broor[1];
[1]All India Institute of Medical Sciences, New Delhi, India, [2]All India Institute of Medical Sciences & INCLEN, New Delhi, India, [3]Centers for Disease Control and Prevention, Atlanta, Georgia, USA, [4]National Institute of Virology, Pune, India, New Delhi, India, [5]Centers for Disease Control and Prevention, New Delhi, India.

**Background:** The burden of influenza in India remains to be defined. We estimated the burden of influenza among hospitalized patients in a rural community of north India during the 2009 H1N1 pandemic (August 2009-July 2010) and post-pandemic (August 2010-July 2011) periods. **Methods:** During August 09-July 2011, residents of Ballabhgarh Health and Demographic Surveillance System (population 87008 on 31 Dec'09) admitted for acute medical illnesses to 33 selected hospitals were enrolled. Influenza virus testing by real time-RT-PCR was done on nasal and throat swabs. A health utilization survey (HUS) was conducted during August2009-July 2010 to estimate hospitalization rates. The annual incidence of influenza-associated hospitalization adjusted for age and admission in the selected health facilities was calculated. **Results:** We enrolled 925 hospitalized patients: 367 during the pandemic and 558 during the post-pandemic period. Overall, 57 (6%) of the 925 tested positive for influenza viruses (22 were 2009A/H1N1; 19 A/H3N2 and 16 influenza B). During the pandemic, 27 (7.4%) were influenza positive vs 30 (5.4%) during the post-pandemic period with 2009A/H1N1 comprising 13

(48%) and 9 (30 %) samples respectively. There were 79423 respondents included in the HUS; 2103 hospitalizations; rate of hospitalization for acute medical illness was 86/10000/year and 61% occurred in study hospitals. The adjusted annual incidence of influenza associated hospitalization was 5 /10000 (95%CI 4-6) for the study period. There was no difference in influenza-associated hospitalization rates during the pandemic (5/10000; CI 4-7) vs. post pandemic (6/10000; CI 4-7) period. Significantly higher incidence rates of influenza-associated hospitalizations were seen in infants during the pandemic (37/10000) as compared to post pandemic period (5/10000) (p=0.03) while the rates were comparable for other age groups across the two periods. **Conclusions:** Influenza was associated with 6% of all medical admission, causing a significant burden to the health care system and families. Infants were at a higher risk of hospital admission due to influenza during the pandemic. This study was supported in part by cooperative agreements U01 IP000206 from the Centers for Disease Control and Prevention, Atlanta, USA

**Results of Sentinel Surveillance of Influenza-Like Illnesses in the Last Two Seasons, 2009–2011, in Aktau, Kazakhstan**

**K.G. Orazova**;
State Sanitary and Epidemiological Surveillance Authority, Aktau, Mangistau Oblast, Kazakhstan.

**Background:** Determining in whom influenza occurs and the viral types causing influenza assists in determining immunization need, devising immunization strategies, and contributes to global influenza knowledge. In 2009, sentinel surveillance of influenza-like illnesses (ILI) was established in 4 outpatient clinics in the city of Aktau, Mangistau Oblast. **Methods:** Sentinel data of ILI morbidity for November-April 2009-2010 and 2010-2011 were used for this abstract. Individual (sex, age), clinical, and epidemiological data of ILI cases were collected from questionnaires. Data were entered and analyzed in Epi-info 2000. Samples for laboratory tests, conducted by the virology laboratory in Aktau using PCR, were collected from all patients >1 year-of-age who met an ILI standard case definition. **Results:** Clinics tested 162 patients in the 2009-2010 epidemic season and 98 patients in the 2010-2011 season. In 2009-2010, 21 (12.9%) of 162 samples were PCR-positive; influenza A was detected in 15 (71.4 %) samples and influenza B in 6 (28.6 %) samples. In 2010-2011, 13 (13.3%) of 98 samples were positive: influenza A virus was detected in 9 (69.2 %) samples and influenza B virus in 4 (30.7 %) samples. Among influenza A viruses were all A/H3N2. Comparison of the ages of tested ILI patients in 2 epidemic seasons showed an increase in the percentage up to 38% in children 1-4 years of age in 2010-2011 and a marked decrease in the percentage of patients $\geq$65 years of age. Weekly distribution of ILI patients during the epidemic season indicates the growth of the consultation rates in weeks 51-52 of 2009 and weeks 9-10 of 2010. Further rises in consultation rates were recorded in weeks 41-42 of 2010, weeks 2-7 of 2011, when PCR-positive results of tested patients were found. In the 2009-2010 epidemic season, the peak of morbidity occured in mid-December with a steady decline starting in March 2010. No tested ILI patient had been vaccinated. **Conclusions:** Seasonal influenza A --A (H3) -- and influenza B circulated during the epidemic season in Aktau; pandemic AH1N1 was not found despite it being found elsewhere in Kazakhstan, showing that multiple sentinel sites are needed to monitor influenza activity.

**Sentinel Surveillance of Influenza-Like Illnesses (ILI) in the Kyrgyz Republic, 2009–2010 and 2010–2011**

**A.Z. Osmonov**;
National State of the Department of Sanitarium Epidemiological Surveillance, Bishkek, Kyrgyzstan.

**Background:** Determining the burden of influenza helps a country ensure there is appropriate care for ill persons, determine immunization need, detect epidemics/pandemics and contribute to global influenza knowledge. To begin these efforts, the Kyrgyz Republic introduced sentinel surveillance with laboratory confirmation of influenza-like illnesses (ILI) in 2009 and we report for its first two influenza seasons. **Methods:** Surveillance was established in a large policlinic in each of the two biggest cities-Bishkek and

Osh. In the first year, we performed only laboratory surveillance for influenza virus and, in the second year, added epidemiological surveillance which allowed us to calculate the ILI rate. For laboratory surveillance, each sentinel site collected samples from Monday to Friday from 3 patients of each age group (1-4, 5-14, 15-29, 30-64, $\geq$65 years) who met the standard WHO case definition. Real-time PCR tests of nasal or throat swabs were performed for influenza A and B virus. Influenza A- positive samples were subtyped for seasonal virus A (H1), A (H3) and pandemic virus A (H1N1)-2009. **Results:** During the 2009-2010 influenza season, 277 ILI patients were tested; 76 (27.4%) of 277 cases were positive for influenza virus. Influenza A was detected in 76 cases (96%) and influenza B in 3 (4%) due to pandemic A/H1N1-09 virus 96% (73/76). During the 2010-2011 season, 74 (31%) of 235 ILI cases were positive. This epidemic season was very unusual, starting with influenza B virus which predominated throughout the season, being found in 70 cases (95%) with one case of influenza A (H1)-2009 and three of influenza A(H3). Influenza B virus (Malaysia) was isolated in 22 cases. During the 2010-2011 season, weekly ILI consultation rates found Osh to be more affected than Bishkek, with a peak incidence of 23 per 10 000 population (31571 population of the catchment area of the policlinic) vs <2 per 10 000 (49185 population of the catchment area of the policlinic) in the peak weeks in Bishkek. **Conclusions:** Laboratory ILI surveillance determined that pandemic A/H1N1 virus was predominant in 2009-2010, and detected an unusual 2010-2011 season due to influenza B. We must broaden our epidemiologic surveillance to give a more complete picture of the burden of influenza.

**Results of Sentinel Surveillance of Severe Acute Respiratory Infections in the Kyrgyz Republic, 2008–2011**

**D.S. Otorbaeva**;
Department for State Epidemiological Surveillance, Bishkek, Kyrgyzstan.

**Background:** Determining the burden of influenza helps a country ensure there is appropriate care for ill persons, plan health care needs, determine immunization need, detect epidemics and contribute to global influenza knowledge. To begin these efforts, the Kyrgyz Republic introduced sentinel surveillance of severe acute respiratory infections (SARI) in 2008. **Methods:** In the first 2 years, we performed only laboratory surveillance for influenza virus and, in the third year, added epidemiological surveillance which allowed us to calculate the SARI rate. For laboratory surveillance, each sentinel site collected samples from Monday to Friday from 3 patients of each age group (1-4, 5-14, 15-29, 30-64, $\geq$65 years) who met the standard WHO case definition. 390 patients were tested in the 2008-2009 season, 664 patients in 2009-2010, and 398 patients in 2010-2011. Nasal and nasopharyngeal swabs were tested by real-time PCR. **Results:** Highest morbidity was recorded in the age group 1-4 years: 351 (90%) of tested 390 patients in 2008-2009, 313 (77%) of 407 tested patients in 2009-2010 and 210 (53%) of tested 398 patients in 2010-2011. The lesser number of children of 1-4 years during the last season was due to limiting testing to $\leq$ 3 patients a week from each age category. Of the 390 patients tested in 2008-2009, 128 (33%) had influenza virus detected: influenza A: H1(N=25;75%), H3 - 20% (96 cases) and influenza B (N=7;5%). Of the 664 patients tested in 2009-2010, 147 (22%) were positive. Most positive cases 84% (123) were detected in weeks 46-48 of 2009, with influenza A (H1)-09. Of the 398 patients tested in 2010-2011, influenza was confirmed in 107 (27%): influenza B was detected in 83 (78%), influenza A(H3) in 22 (21%) and influenza A/H1N1-09 in 2 cases (2%). 8 out of 398 laboratory tested SARI patients went to ICUs in 2010-2011. No deaths were recorded among them. The peak of morbidity (76 and 71 per 1000 hospitalized patients) was recorded in weeks 47 and 52 of 2010, and otherwise during the season SARI morbidity varied from 26-46 per 1000 hospitalized patients. **Conclusions:** Laboratory surveillance allowed us to detect the unusual epidemic with influenza B virus in 2010-2011and adding epidemiological surveillance to laboratory surveillance in 2010-2011 gave us for the first time the ability to better measure the burden of influenza.

## J4. Effective and Sustainable Surveillance Platforms

Tuesday, March 13
3:15 PM – 4:45 PM
Centennial IV

### Population-based Surveillance for Norovirus in Guatemala: Estimation of Disease Burden and Clinical Severity

**A.J. Hall**[1], W. Arvelo[2], A. Estevez[3], U.D. Parashar[1], K.A. Lindblade[2];
[1]Centers for Disease Control and Prevention, Atlanta, GA, USA, [2]Centers for Disease Control and Prevention Regional Office for Central America and Panama, Guatemala City, Guatemala, [3]Universidad del Valle de Guatemala, Guatemala City, Guatemala.

**Background:** Norovirus is recognized as a leading cause of diarrheal disease in developed countries. However, limited availability of diagnostics has hindered understanding of their role in developing countries, where most severe diarrheal disease and deaths occur. We therefore sought to determine the disease burden, seasonality, and clinical characteristics of norovirus disease in Guatemala through enhancement of an existing surveillance platform. **Methods:** Centralized local capacity for norovirus diagnostics was established and applied to a population-based surveillance system at multiple government health care facilities. Patients of all ages presenting with acute diarrhea to participating hospitals and ambulatory clinics in two departments, Santa Rosa (October 2007-August 2010) and Quetzaltenango (August 2009-August 2010), were recruited. Demographic and clinical data were collected along with stool specimens for norovirus detection by real-time reverse transcription-polymerase chain reaction. Clinical severity was evaluated using a modified Vesikari score for gastroenteritis on a 21-point scale. Incidence rates were calculated using the catchment area population and adjusted for healthcare utilization rates developed from household surveys. **Results:** We enrolled 2403 patients with diarrhea in the study, including 528 (22%) hospitalized and 1875 (78%) ambulatory patients; 1460 (61%) were children aged <5 years. Norovirus was detected in 114 (22%) hospitalized patients and 227 (12%) ambulatory patients, with seasonal increases during November-January. Patients infected with norovirus had a median clinical severity score of 6, slightly less severe than that of rotavirus-infected patients (median=8) but more severe than patients with bacterial or parasitic infections (median=4). Overall, we estimate norovirus was associated with 21 hospitalizations, 358 ambulatory visits, and 2261 community illnesses annually per 100,000 population. **Conclusions:** Utilizing an effective population-based surveillance system, this study demonstrates that norovirus is one of the leading causes of both moderate and severe diarrheal disease in Guatemala. These data can help guide appropriate clinical management and public health interventions for diarrheal disease.

### Strengthening Preparedness for West Nile Fever in the European Union Using Innovative Tools

**L. Marrama Rakotoarivony**[1], C. Bogaardt[1], J. Mantero[1], A. Sirbu[2], F. Popovici[3], A. Pistol[4], S. Rehmet[1], M. Janssen[5], W. Oei[5], A. Lenglet[1], K. Leitmeyer[1], F. Santos-O'Connor[1], W. Van Bortel[1], V. Estevez[1], E. Depoortere[1], H. Zeller[1], D. Coulombier[1];
[1]European Centre for Disease Prevention and Control, Stockholm, Sweden, [2]National Institute of Public Health - National Centre for Surveillance and Control Communicable Disease, Bucharest, Romania, [3]Nationale Institute of Public Health - Regional Centre of Public Health Bucuresti, Bucharest, Romania, [4]Nationale Institute of Public Health - National Centre for Surveillance and Control Communicable Disease, Bucharest, Romania, [5]University Medical Center, Utrecht, Netherlands.

**Background:** Since 2010, epidemiological and virological findings in the European Union (EU) countries indicate a geographical expansion of West Nile fever (WN) involving viruses from lineages 1 and 2. In this context, the European Centre for Disease Prevention and Control (ECDC) is developing tools to support

preparedness, risk assessment and response activities of EU Member States. **Methods:** Three complementary tools are being developed. A first tool monitors the spatial distribution of confirmed human cases at the sub-national level in the EU and neighboring countries using epidemic intelligence data from official sources. A second tool assesses the risk for WN transmission to humans in an area, using an algorithm based on multi-sectoral surveillance indicators. Finally, the European Up-Front Risk Assessment Tool (EUFRAT) estimates the risk of getting contaminated blood products collected in areas with WN transmission. It is based on a deterministic model using disease-specific parameters, data on current outbreaks, population, screening and processing techniques applied in the transfusion chain. **Results:** Through ECDC West Nile Risk Maps, information on areas reporting human cases is published weekly on the ECDC website in the form of maps and tables for the duration of the transmission season. These outputs have already been linked to by a variety of national public health agencies, travel medicine networks and public health forums on their internet sites. The risk assessment tool is currently reviewed by a panel of experts prior to be shared with EU countries. The EUFRAT is being piloted by a limited number of EU Member States before being shared with public health authorities in the EU. **Conclusions:** Due to the evolving situation of WN in the EU, strengthening preparedness is essential. The combination of these three tools represents an innovative approach for risk assessment as the outcome of one tool can be used as input to the other. Thus these tools provide public health decision makers with complementary and timely information, supporting them in their response to the risk posed by WN. Moreover, the tools contribute to harmonizing and strengthening risk assessment during WN outbreaks in and around the EU. Finally, their use might be extended to other vector-borne diseases with a potential impact on blood safety.

**Personal Digital Assistants as a Data Collection Tool for Integrated Infectious Disease Surveillance Systems in Greater Accra and Northern Regions, Ghana**

**R. Akuffo**[1], B. Doman[1], B. Olander[2], I. Brace[2], P. Agbenohevi[3], C. Sarpong[4], K. Sagoe[5], E. Dueger[6,2];
[1]US Naval Medical Research Unit-3 Ghana Detachment, Accra, Ghana, [2]US Naval Medical Research Unit-3, Cairo, Egypt, [3]37 Military Hospital, Accra, Ghana, [4]Tema General Hospital, Tema, Ghana, [5]Tamale Teaching Hospital, Tamale, Ghana, [6]Centers for Disease Control and Prevention, Cairo, Egypt.

**Background**: Infectious disease surveillance systems require timely, accurate and consistent data. In developing countries, limited resources make developing such surveillance systems challenging. However, with the development of innovative products such as PDAs, smartphones, and tablets as potential tools for data collection, the development of infectious disease surveillance systems is becoming a reality. **Methods:** In 2009, integrated syndromic surveillance for acute respiratory, febrile, and diarrheal infections began in 3 major Ghanaian referral hospitals in Accra, Tema, and Tamale. In January 2011, study sites transitioned from paper-based to PDA data collection. PDA questions were programmed using shared code software Questionnaire Mobile developed by the Centers for Disease Control's Disease Detection Program, Guatemala. **Results:** Reporting time of surveillance data decreased from an average of 30 days to 5 days. Introduction of mandatory questions on the PDAs led to a 5.4% increase in the response rate to those questions compared to paper-based forms. Hospital field staff reported greater satisfaction, efficiency and pride using PDAs compared to paper-based forms. On average, PDAs saved 30 hours of data entry, 17.5 hours of data collection, and 4 hours of material assembly per week compared to paper-based forms. The initial time and costs for set-up of data management using PDAs is relatively high. In 2009, each PDA cost $400 USD (2 per site). Programming of PDAs for the Ghana sites required approximately 200 hours of programming by highly skilled staff. An additional 100 man-hours were spent training hospital site staff in use of PDAs. **Conclusions:** In our study, the use of PDAs was found to be more efficient than that of paper-based forms and allowed more real-time reporting of data. Hospital field staff indicated that they preferred using PDAs over the paper-based forms. The burden of work was reduced significantly by the elimination of the double data entry

step, and the quality of data was increased by the addition of mandatory questions. If developing countries committed to building efficient infectious disease surveillance systems are able to secure initial start-up costs, PDAs are a potential tool for data collection.

**Comparison of Current and Proposed World Health Organization Case Definitions for Hospitalized Acute Respiratory Illness in a Population-based Surveillance Site in Egypt**

**E. Rowlinson**[1], E. Dueger[1,2], A. Mansour[1], H. Mansour[1], M. Said[1], N. Azzazy[3], M. Abukela[3], M. Nematallah[3], A. Shawky[1], N. el-Sayed[3];
[1]Global Disease Detection and Response Program, U.S. Naval Medical Research Unit No. 3, Cairo, Egypt, [2]Centers for Disease Control and Prevention, Atlanta, GA, USA, [3]Egyptian Ministry of Health, Cairo, Egypt.

**Background:** Multiple case definitions exist for surveillance of patients hospitalized with respiratory infections. We compared the sensitivity of the WHO's current and proposed case definitions for Severe Acute Respiratory Infection (SARI). **Methods:** The study population included hospitalized patients from a population based surveillance site in Damanhour, Egypt that met a broad set of ARI enrollment criteria, including WHO's current definitions for SARI , moderate and severe pneumonia , and the CDC International Emerging Infections Program definition for pneumonia (fever or hypothermia or abnormal white blood cell count AND at least one of the following: tachypnea, cough, abnormal breath sounds, sputum production, hemoptysis, chest pain, dyspnea). Patients meeting the current WHO SARI case definition were compared with those meeting the proposed WHO SARI definition (a hospitalized patient with current fever ≥38°C or history of fever plus cough). Naso- and oropharyngeal (NP/OP) swabs were tested for influenza using rt-PCR. **Results:** Between May 2009 and August 2011, 3,745 NP/OP swabs were collected from 3,850 hospitalized ARI patients; 435 (11.6%) were influenza positive. The current and new proposed WHO SARI definitions captured 896 (23.3%) and 3466 (95.6%) of these patients, respectively, and captured 72 (16.6%) and 429 (98.6%) patients positive for influenza. The current WHO SARI definition missed 14 SARI deaths, 2 of which were due to Influenza A; all 14 deaths were captured by the proposed definition. **Conclusions:** The current WHO SARI case definition failed to capture over 77% of hospitalized ARI cases and influenza events, and excluded a number of severe events, including influenza deaths. The proposed WHO SARI case definition captured nearly all hospitalized ARI and influenza cases in this population and is much easier to implement for sentinel surveillance, especially where physicians may not be familiar with case definition algorithms. However, as a larger number of cases will be captured, effective sampling schemes for pathogen testing must be considered in resource-limited settings.

**Nipah Virus Infection in Bangladesh: Updates from the 2011 Nipah Season on Where and How Transmission Occurs**

**A. Chakraborty**[1,2], M. Jahangir Hossain[1], H.M. S. Sazzad[1], M. Husain[2], S. Parveen[1], M. Islam[1], G. Podder[1], S.S. Banu[2], S. Afroj[1], P.E. Rollin[3], P. Daszak[4], M. Rahman[2], S.P. Luby[1,3], E.S. Gurley[1];
[1]International Centre for Diarrhoeal Diseases Research, Bangladesh (ICDDR,B), Dhaka, Bangladesh, [2]Institute of Epidemiology, Disease Control and Research (IEDCR), Dhaka, Bangladesh, [3]Centers for Disease Control and Prevention (CDC), Atlanta, GA, USA, [4]EcoHealth Alliance, New York, NY, USA.

**Background:** Since 2001, Nipah virus (NiV) encephalitis outbreaks have been reported in humans almost yearly during December-April in a geographically limited area of northwest and central Bangladesh, sometimes referred to as the 'Nipah belt'. Previous investigations have identified drinking raw date palm sap as a primary route of transmission from bats to people and subsequent person-to-person transmission is common. We present here the epidemiologic findings from investigations of the NiV cases identified during December 2010-March 2011. **Methods:** We identified isolated cases and clusters of NiV encephalitis through active surveillance conducted jointly by IEDCR and ICDDR,B at five sentinel

hospitals in northwest and central Bangladesh. We also detected NiV cases through passive reporting of suspect NiV encephalitis cases from physicians across the country. We used structured questionnaires, in-depth interviews, and group discussions to collect exposure histories of the cases in the 30 days before onset of illness. **Results:** We identified 42 NiV cases, 39 (93%) died. Median age of the cases was 29 years (range: 2-65 years). Median duration from onset of illness to death was 6 days (range: 1-47 days). Thirty-four cases formed four distinct clusters, the remaining 8 were isolated cases. Cases occurred in eight districts, including one district in eastern Bangladesh. Twenty-one of the 42 cases had history of drinking raw date palm sap, but the only exposure identified for 4 cases was drinking fermented date palm sap. Six cases came into contact with a NiV case prior to their illness onset, including a health care worker. **Conclusions:** In 2010-11, like previous NiV seasons in Bangladesh, NiV caused high case fatality and transmission likely occurred through both drinking raw date palm sap and person-to-person contact. Identification of drinking fermented date palm sap as a likely route of transmission to humans suggests the need for raising public awareness to strictly avoid both raw and fermented sap to prevent NiV transmission. For the second year in a row, a health care worker died from NiV infection in Bangladesh highlighting the urgent need to improve infection control in hospitals, particularly during the NiV season. The case detected in eastern Bangladesh is the first reported NiV case outside of the 'Nipah belt'.

**Department of Defense (DoD) Global Laboratory-based Influenza Surveillance Program at the US Air Force School of Aerospace Medicine (USAFSAM): A Thirteen Year Review of Influenza**

**V. MacIntosh**[1], L.C. Canas[1], J.P. Smith[1,2], B.C. Connors[1,2], C.A. Schlorman[1,2], K.J. Tastad[1,2], J.P. Abshire[1,2], L.V. Lloyd[1,3], A.B. Owens[4,5], A.C. Guerrero[6], P.A. Sjoberg[1];

[1]US Air Force School of Aerospace Medicine, Wright-Patterson Air Force Base, OH, USA, [2]Henry M. Jackson Foundation for the Advancement of Military Medicine, Inc., Bethesda, MD, USA, [3]Oak Ridge Institute for Science and Technology, Oak Ridge, TN, USA, [4]US Air Force Medical Support Agency, San Antonio, TX, USA, [5]X-Technologies, Inc., San Antonio, TX, USA, [6]US Army Medical Department Center & School, Fort Sam Houston, TX, USA.

**Background:** USAFSAM serves as the DoD Influenza Reference Center, providing a global lab-based influenza and viral respiratory surveillance system for DoD beneficiaries and foreign nationals. This system augments the Global Influenza Surveillance Network activities year-round: 1) isolating and identifying circulating viruses, 2) evaluating influenza vaccine effectiveness, 3) detecting newly emerging strains and 4) providing data and original specimens as needed to the Centers for Disease Control and Prevention (CDC). **Methods**: Over 70 global sites collect epidemiologic data and specimens from patients with influenza-like illness and send them to USAFSAM for data and lab analysis. All specimens are cultured and molecularly characterized, and many influenza viruses are further sequenced. USAFSAM maintains an archive of isolates and original specimens. Surveillance data are shared with participating sites, DoD, CDC, and the World Health Organization (WHO). Original specimens and isolates are shared with CDC for antigenic characterization and reference and vaccine seed strains. **Results**: Since 1998, 69,786 specimens from 43 countries have been processed, with 17,894 (26%) being influenza; 15,114 (84%) type A and 2,780 (16%) type B. Percent of specimens positive for influenza increased from 17% prior to the pandemic season to 33% in following seasons. Influenza type A and B percentages varied by seasonal year. Influenza type A dominated most seasons, representing up to 93% of influenza typed in 2003-04. During the 5 seasons when influenza A subtypes were routinely identified, H1 predominated in 3 and H3 in 2 seasons. In 2008-09 the earliest US detections of 2009 H1N1 occurred nearly simultaneously at the Naval Health Research Center and USAFSAM (April 14, 2009); USAFSAM processed 19,072 specimens that year. Vaccine-related contributions from this program have included at least 9 virus seed and/or reference strains. **Conclusions:** The DoD, Laboratory-based Influenza Surveillance Program managed at USAFSAM has expanded and evolved across the DoD, centralizing laboratory

capability in collaboration with the DoD, CDC and WHO. The ongoing, comprehensive system continues to make important global contributions to international influenza surveillance and prevention.

## J5. Outbreak Investigation: Lab and Epi Response I

Tuesday, March 13
3:15 PM – 4:45 PM
Regency VI

**Enhanced Surveillance Following a Case of Inhalation Anthrax: Utilizing New and Old Systems and Partnerships**

**M. Lehman**[1], R. Traxler[1], D. Blaney[1], S. Shadomy[1], T. Gomez[1], C. Rubin[1], P. Snippes Vagnone[2], C. Lees[2], C. Ballew[3], T.K. Miller[4], L. Kightlinger[5], V. Horan[5], T. Murphy[6], P. Amuso[7], A. Schmitz[8], D. Wong[9], T. Treadwell[1], R. Lynfield[2], Anthrax Investigation Team;
[1]Centers for Disease Control and Prevention, Atlanta, GA, USA, [2]Minnesota Department of Health, St. Paul, MN, USA, [3]Montana Department of Public Health and Human Services, Helena, MT, USA, [4]North Dakota Department of Health, Bismark, ND, USA, [5]South Dakota Department of Health, Pierre, SD, USA, [6]Wyoming Deaprtment of Health, Cheyenne, AB, USA, [7]Florida Deaprtment of Health, Tampa, FL, USA, [8]Florida Deaprtment of Health, Tallahassee, FL, USA, [9]National Park Service, Albuquerque, NM, USA.
**Introduction:** Inhalation anthrax was confirmed on Aug. 6, 2011 in a 61 year-old from FL. The patient had a 3 week history of travel through ND, WY, MT and SD prior to hospitalization in MN on Aug. 4. He had exposure to dust stirred up by hooved animals, and handled rocks and animal products (e.g. antlers) during travel. The specific causal exposure was unknown. Enhanced surveillance of animal and human cases was initiated because animals or animal products can be sources of exposure, and other human cases may have occurred. **Methods:** A multi-state joint animal and human investigation was conducted electronically and via phone covering June 1-Aug. 31, 2011. Anthrax cases in domestic or wild animals were sought from veterinary diagnostic and reference laboratories in the six implicated states. The National Animal Health Laboratory Network (NAHLN) reported *Bacillus* spp. isolates pending rule-out or referral for *B. anthracis*. The Laboratory Response Network (LRN) in the six states (462 human/7 vet) was queried electronically for non-hemolytic *Bacillus* spp. not already referred to LRN reference laboratories. Queries were also sent through health alerts and directly via e-mail to infection preventionists (IP), medical examiners (ME), and coroners in ND, SD, MT, MN and part of western FL for cases of unexplained death or fulminant illness potentially due to anthrax. Letters were sent by U.S. mail or fax if e-mail addresses were unknown. **Results:** No anthrax cases were reported in domestic or wild animals in 2011 prior to August. An infected cow was identified in SD on Aug. 11, but the case-isolate was a different genotype than the human case-isolate. Neither veterinary nor clinical laboratories received other *B. anthracis* isolates; there was no evidence of missed *Bacillus* spp. isolates. No potential anthrax cases were identified through IP, ME or coroner queries. **Conclusions:** Although no related anthrax cases were identified, the ability to comprehensively surveil multiple networks and systems, which included anthrax-specific diagnostics and individuals likely to make an empirical diagnosis, was critical in assessing for additional cases in animals and people. Investment in a “One Health” approach, encouraging coordination between veterinary and human health, and support of the LRN and NAHLN made this investigation possible.

**Inhalation Anthrax, 2011: Clinical Course and Investigation**

**J. Griffith**[1], D. Blaney[2], A. DeVries[1], N. Pesik[2], S. Monroe[2], D. Stanek[3], P. Ryder[4], M. Sprenkle[5], L. Delaney[2], M. Sullivan[1], S. Tostenson[1], R. Lynfield[1];

[1]Minnesota Department of Health, St. Paul, MN, USA, [2]Centers for Disease Control and Prevention, Atlanta, GA, USA, [3]Florida Department of Health, Tallahassee, FL, USA, [4]Pinellas County Department of Health, St. Petersburg, FL, USA, [5]Hennepin County Medical Center, Minneapolis, MN, USA.
**Background:** Inhalation anthrax (IA) is a rare, frequently fatal, infection. Cases have been associated with risks such as working in wool mills, exposure to animal-hide drums, or bioterrorism. On August 6, 2011, MN Public Health lab (PHL) identified a referred isolate from blood culture as *Bacillus anthracis* (BA) using Laboratory Response Network (LRN) protocols. The patient was a 61 year-old from FL who had traveled through ND, WY, MT and SD and was hospitalized in MN 2 days after developing symptoms. An investigation into his source of exposure to BA was begun. **Methods:** Interviews eliciting travel history and possible exposures during the previous 60 days were conducted with the case-patient and his wife, and medical records reviewed. Environmental sampling of locations associated with the case-patient was done and analyzed by MN and FL PHL. **Results:** ***Epidemiology.*** The case-patient recalled exposure to dust stirred up by hoofed animals while traveling in his car with open windows; he also collected rocks and handled antlers. Eight months prior to illness, he worked with rocks, antlers, hides, and metals in a home craft workshop; materials were still present at home. The FBI ruled out bioterrorism. ***Clinical.*** The case-patient had a history of chemical pneumonitis. He developed headache, cough, rigors and malaise on August 2 and was admitted with shortness of breath. A chest radiograph revealed right sided infiltrates. He was subsequently intubated and a chest tube was placed to drain a new pleural effusion. He was treated with ciprofloxacin, clindamycin, and meropenem, and received anthrax immune globulin when diagnosed with IA. He remained hemodynamically stable and was discharged to home on ciprofloxacin to complete a 60-day course. ***Environmental.*** Samples were collected from interior and exterior surfaces of the car, including air filters; from hiking boots, rocks, fishing rod, fishing lures and purchased elk antlers; and from electronic equipment, tools and other items in his workshop, garage and home. All samples were negative for BA. **Conclusions:** Although the case-patient had been in enzootic areas for anthrax, and had multiple exposures to soil and animal products, a specific causal exposure was not determined. Rapid identification of the BA isolate by the LRN facilitated prompt clinical and public health investigation.

**FoodCORE: Foodborne Diseases Centers for Outbreak Response Enhancement—Improving Foodborne Disease Outbreak Response Capacity in State and Local Health Departments**

**G.K. Biggerstaff**, J.G. Wright, J.R. Mitchell, FoodCORE;
Centers for Disease Control and Prevention, Atlanta, GA, USA.
**Background:** Each year foodborne diseases (FBD) cause illness in approximately 1 in 6 Americans, resulting in 128,000 hospitalizations and 3,000 deaths. Decreasing resources impact the ability of public health officials to identify, respond to, and control FBD outbreaks. FoodCORE was established to address gaps in FBD outbreak response by improving capacity in three core areas: laboratory, epidemiologic, and environmental health activities. FoodCORE sites build collaborative surveillance and response programs to conduct rapid, coordinated, and standardized investigations of FBD. **Methods:** FoodCORE sites were selected via competitive review process. Performance metrics, focusing on *Salmonella*, Shiga toxin-producing *Escherichia coli*, and *Listeria* (SSL), were developed based on the Council for Improving Foodborne Outbreak Response Guidelines. FoodCORE sites provide quarterly reports that are compiled by CDC FoodCORE staff. **Results:** There are currently seven FoodCORE sites: New York City, North Carolina, Ohio, South Carolina, Tennessee, Utah, and Wisconsin. From 10/1/2010 to 6/30/2011, data were reported for 33 metrics. A total of 5,267 SSL isolates were submitted to the public health laboratories. For all sites, an average of 96% of SSL isolates had PFGE data available (range: 85-100%). For *Salmonella*, the median turn-around time (TAT) for serotyping during the first two quarters (Q1/2) was 9 days (range: 6-13); this was reduced to 6 days (range: 4-10) in the third quarter (Q3). Median TAT for *Salmonella* PFGE results decreased from 12 days (range: 3-40) in Q1/2 to 8 days (range: 2-22) in Q3.

A total of 3,875 SSL cases were reported to epidemiology staff. For all sites, interviews were attempted for an average 89% (range: 74-100%) of cases. By the end of Q3, the median days from case report to interview attempt was 1 (range: 0-2). **Conclusions:** FoodCORE promotes the evaluation and application of surveillance and investigation strategies to improve the detection, investigation, and control of FBD outbreaks. FoodCORE sites are implementing work plans to address gaps in FBD outbreak response. Enhanced outbreak response efforts can shorten the time it takes to identify a source of infection and pinpoint how and why contamination occurred, in order to limit additional illnesses and help prevent future FBD outbreaks.

**Picking the Cream of the Crop: Ranking of States for Investigation of Foodborne Disease Outbreaks**
**J. Howland**, M. Pyra, J. Oesterreich, C. Conover;
Illinois Department of Public Health, Chicago, IL, USA.
**Background:** Outbreak investigation is an important component of foodborne disease control and a central responsibility of state health departments. Recent reports published by The Center for Science in the Public Interest (CSPI) and the Trust for America's Health assess states' capacity for foodborne outbreak investigation using outbreak detection rate and pathogen identification respectively. Due to their recognized excellence in foodborne outbreak investigation, Minnesota and Oregon are used as benchmarks in the CSPI paper and other publications. **Methods:** We used 2005-2008 data from CDC's Foodborne Outbreak Online Database to rank states on the following measures: outbreak identification rate per 100,000 population; percent of outbreaks with a laboratory-confirmed pathogen identified; outbreak identification rate for outbreaks with more than ten patients per 100,000 population; and percent of outbreaks with the food vehicle identified. The Spearman rank correlation was used to test the independence of the rankings. **Results:** Only one state, Hawaii, ranked in the top ten on each of the ranking lists. Despite its recognized excellence, Minnesota only appeared in the top five on two lists: outbreak identification rate and outbreak identification rate for outbreaks with more than ten patients. Oregon never appeared in the top five. State rankings for the outbreak identification rate were significantly correlated with rankings for outbreak identification rate for outbreaks with more than ten patients (r=0.93, p<.0001). No other rankings were significantly correlated. **Conclusions:** Ranking states on a single factor may inaccurately represent outbreak investigation capabilities and exclude factors relevant to outbreak control. Two ranking measures used in past publications, outbreak detection rate and rate of pathogen detection, are not significantly correlated, indicating that neither factor fully describes a state's investigative capacity. Assessment of states' outbreak investigation capabilities may benefit from inclusion of more than one measure and measures that more directly relate to outbreak control, such as percent of outbreaks with a laboratory-confirmed pathogen identified and percent of outbreaks with the food vehicle identified.

**A Cluster of Necrotizing Cutaneous Mucormycosis Following a Tornado—Joplin, Missouri, 2011**
**R.N. Fanfair**[1], S.D. Bennett[1], K. Benedict[1], J. Bos[2], Y.-C. Lo[1], T. Adebanjo[1], S.R. Lockhart[1], M. Brandt[1], J. Harris[1], G. Turabelidze[3], B.J. Park[1];
[1]Centers for Disease Control and Prevention, Atlanta, GA, USA, [2]MO Department of Health and Senior Services, St. Louis, MO, USA, [3]MO Dept of Health and Senior Services, St. Louis, MO, USA.
**Background:** Mucormycosis is a rare infection caused by environmentally-associated Mucormycetes molds. We investigated a cluster of cutaneous mucormycosis in persons who sustained trauma during the May 22, 2011 tornado in Joplin, Missouri. **Methods:** A case was defined as a necrotizing soft-tissue infection in a tornado-injured person with evidence of a Mucormycete by culture, immunohistochemistry and DNA sequencing. We conducted a case-control study to compare location, severity and mechanism of injury, and pre-disposing conditions by reviewing medical records and conducting interviews on case-patients and other hospitalized and injured controls. DNA sequencing

was performed on clinical specimens to identify species. **Results:** Of the 13 identified case-patients, median age was 48 years (range 13-76); two (15%) had diabetes and 10 (77%) were admitted to an intensive care unit (ICU). Case-patients had a mean of four wounds; foreign material was recovered from wounds in six patients (46%). Five (38%) patients died. Case-patients were all located in the zone that sustained the most severe damage during the tornado. On univariate analysis, case-patients had more wounds than controls (n=35) (mean of 4 vs. 2 wounds, p<0.001); were more likely to be admitted to ICU (77% vs. 31%, p=0.008), have penetrating trauma (39% vs. 9%, p=0.025), have rhabdomyolysis (39% vs. 0%, p=0.001), or develop sepsis during hospitalization (54% vs. 6%, p=0.001). DNA sequencing of the D1/D2 region from all 13 case-patients yielded the Mucormycete *Apophysomyces trapeziformis* (AT). **Conclusions:** Cutaneous mucormycosis has been reported after previous natural disasters, however, this is the first known cluster occurring after a tornado. Severe, penetrating injury and rhabodomyolysis were associated with infection in univariate analysis. Clinicians should suspect Mucormycetes in necrotizing soft-tissue infections following natural disasters, and consider prompt treatment for suspected infections.

**Multi-State Q Fever Outbreak in the Northwestern United States, 2011**

**A. Anderson**[1], A. Bjork[1], N. Marsden-Haug[2], R. Nett[3], G. Kersh[1], W. Nicholson[1], D. Gibson[3], T. Szymanski[4], M. Emery[5], P. Kohrs[6], D. Woodhall[1], J. McQuiston[1], Outbreak Response Group;
[1]Centers for Disease Control and Prevention, Atlanta, GA, USA, [2]Washington State Department of Health, Shoreline, WA, USA, [3]Montana DPHHS, Helena, MT, USA, [4]Montana Department of Livestock, Helena, MT, USA, [5]USDA, NVSL, Ames, IA, USA, [6]Washington State Department of Agriculture, Shoreline, WA, USA.

**Background:** We investigated an outbreak of Q fever associated with goat farms in Washington and Montana that occurred in 2011. Washington and Montana typically report 0-3 Q fever cases annually. In April, 2011, *Coxiella burnetii*, the bacterial agent of Q fever, was detected in a goat placenta collected at a farm in Grant County, WA (Farm A) where 28% (14 of 50) of pregnant goats had aborted since January. On May 25, a Q fever patient reported purchasing goats from Farm A. Subsequently, Montana farms in Cascade and Teton counties, both of which had family members and/or visitors ill with flu-like symptoms compatible with Q fever infection, reported purchasing goats from Farm A the previous winter. **Methods:** We conducted case finding and cohort studies among persons potentially exposed to goats purchased from Farm A and conducted sampling of the goats and environment to characterize the extent of the outbreak, determine risk factors for infection, and develop appropriate interventions. **Results:** Goats sold by Farm A since July 2010 were traced to 21 farms in 14 counties across 3 states (WA, MT, OR). Seventeen farms participated in the outbreak investigation. To date, 19% (20 of 108; 11 in WA, 9 in MT) of serologically tested persons, including goat owners, farm visitors, and neighbors, met the outbreak case definition of an anti-*C. burnetii* Phase II immunoglobulin G antibody titer of 1:128 or greater by immunofluorescence assay. No deaths were reported; 20% (4 of 20) of patients were hospitalized. Evidence of *C. burnetii* infection in goats was detected in 16 of the 17 herds, including confirmation of bacterial shedding in feces, vaginal mucous, or milk in 25% (153 of 605) of all goats tested and an overall seroprevalence of 21% (131 of 615). A herd management plan was implemented in WA and MT advising goat owners to, among other guidelines, disinfect birthing areas, segregate newly acquired animals, maintain a detailed animal registry, and report animal abortions and positive Q fever test results to state authorities. **Conclusions:** This outbreak illustrates that recognition and investigation of Q fever illness rely on surveillance by both animal health and public health authorities and close collaboration between local, state and federal agencies. Continued community awareness is essential for disease prevention and control.

## J6. Late-Breakers II

Tuesday, March 13
3:15 PM – 4:45 PM
Regency VII

**Nodding Syndrome, a Novel Neurologic Illness of Unknown Etiology—South Sudan, 2011**

**S. Bunga**[1], J.L. Foltz[1], L.P. Reik[2], A. Abubakar[3], J.J. Sejvar[1], C.N. Colorado[1], M.L. Opoka[4], G. Mindra[5], J.D. Ratto[1], C.J. Blanton[1], S.F. Dowell[1];
[1]Centers for Disease Control and Prevention, Atlanta, GA, USA, [2]Ministry of Health, Juba, South Sudan, [3]World Health Organization, Juba, South Sudan, [4]World Health Organization, Cairo, Egypt, [5]UNICEF, Juba, South Sudan.

**Background:** Nodding Syndrome (NS), an unexplained neurologic illness predominantly affecting children aged 5-15 years is possibly a novel seizure disorder, characterized by head-nodding. Published descriptions from Tanzania and South Sudan suggest that nodding is precipitated by exposure to food or cold and includes progressive cognitive dysfunction, neurological deterioration, and stunted growth. Postulated etiologies from past investigations include infectious, nutritional, toxicological, and genetic factors. Past investigations from Tanzania, Uganda and South Sudan remain inconclusive and the cause of NS is unknown. In May of 2011, CDC investigated a cluster of NS in South Sudan. **Methods:** To ascertain if illness in South Sudan is same as reported previously and to identify risk-factors, we conducted an age- and village-matched case-control investigation, collecting exposure information and biological specimens. We used conditional multiple logistic regression analysis to assess risk factors. **Results:** We enrolled 38 case-control pairs. The mean age-of-onset for head-nodding was 9.2 years (Range: 5.0-14.0). Cases were more likely to have had hunger episodes, sometime from birth to 2 years (matched odds ratio [mOR]: 8.2; 95% confidence interval [CI]: 1.2-∞) and more likely to have consumed food from their own-farm (mOR: 10.0; 95% CI: 1.4-434.0). Studied risk factors among cases, exposure to munitions, consumption of sorghum, crushed roots and small fish, and suspected maternal pre-natal exposures did not show any significant association. Onchocerciasis, confirmed by skin-snip, was more common among cases (mOR: 3.2; 95% CI: 1.2-8.7). **Conclusions:** The NS clinical presentation in South Sudan is consistent with NS as described elsewhere in East Africa. Our preliminary findings* highlight nutrition factors not previously identified that include, going hungry early-in-life; having consumed food from own-farm; and continue to implicate onchocerciasis. We recommend reinforcing mass ivermectin treatment for onchocerciasis, nutritional support for malnourished children, and anti-epilepsy medication for seizures. Future priorities include improving surveillance to monitor disease burden and geographic distribution, and continued work to determine etiology and guide interventions.

**Contact Investigation of International Travelers Exposed to a Rabid Zebra, Kenya—2011**

**E.W. Lankau**[1], D.M. Tack[1], M. Obonyo[2], S. Kadivane[2], J.D. Blanton[1], J.M. Montgomery[3], W. Arvelo[3], E.S. Jentes[1], N.J. Cohen[1], G.W. Brunette[1], N. Marano[1], C.E. Rupprecht[1];
[1]Centers for Disease Control and Prevention, Atlanta, GA, USA, [2]Ministry of Public Health and Sanitation, Nairobi, Kenya, [3]Centers for Disease Control and Prevention, Nairobi, Kenya.

**Background:** Although dogs present the greatest risk of rabies virus transmission in much of the world many wildlife species are susceptible to rabies infection. Travelers to canine rabies-enzootic regions might be unaware of rabies exposure risks from wildlife at their destinations. We present an investigation of international travelers exposed to a rabid zebra at a safari lodge in Eastern Kenya. Visitors from July 24-August 26 were first notified of the rabies diagnosis via an email sent by the lodge on August 30 after receiving the test results. **Methods:** In collaboration with international partners, the US CDC conducted a contact investigation of all US travelers previously notified by the lodge. CDC

estimated an infectious period from 14 days before illness onset of August 24 until the zebra's death on August 26 (or August 10-26). State public health officials called US travelers who visited the lodge from August 10-26 to perform risk assessments and to provide, if needed, post-exposure prophylaxis (PEP) recommendations. Travelers who visited from July 24 to August 9 were not considered at risk of rabies exposure but were called to follow up with rabies education. **Results:** Of 243 total travelers to the lodge, 136 (56%) were from the United States and 77 (57%) of these visited during the infectious period. A total of 28 US travelers had already initiated PEP when interviewed by US public health officials. Two of these 28 visited the lodge during the infectious period, reported skin contact with the zebra's saliva, and were uncertain about the presence of skin wounds at the time of contact. The remaining 26 US travelers who received PEP were assessed to have had low- or no-risk exposures for which PEP would not have been recommended. The World Health Organization coordinated the contact investigation for the 107 nonUS travelers from 16 other nations. **Conclusions:** US travelers sought PEP before being reached by public health officials, resulting in unnecessary PEP administration. Collaboration among public health officials and the travel industry is vital for providing accurate and timely public health responses to disease exposure events involving international travelers. International travelers should consult a physician before departure to discuss preventive care and health risks, including rabies, at their destination.

**Characteristics of Community Transmission of H275Y Oseltamivir-resistant A(H1N1)pdm09 Influenza in Australia**

**K.L. Hardie**[1], A.C. Hurt[2], D. Durrheim[1], N.J. Wilson[3], Y.M. Deng[2], M. Osbourn[1], N. Gehrig[3], A. Kelso[2]; [1]Hunter New England Population Health, Newcastle, Australia, [2]WHO Collaborating Centre for Reference and Research on Influenza, Melbourne, Australia, [3]Hunter Area Pathology Service, Newcastle, Australia.

**Background:** Oseltamivir resistance in A(H1N1)pdm09 influenza strains is rare, usually <1%, in untreated community cases. Transmission of oseltamivir-resistant A(H1N1)2009 viruses has only been detected occasionally in closed, near-contact settings. We report the first cluster of sustained community transmission. **Methods:** Virological analyses are conducted year-round on influenza specimens from the Asia-Pacific region by the World Health Organisation Collaborating Centre for Reference and Research on Influenza. Detailed information was collected on patients with a confirmed oseltamivir-resistant virus infection. **Results:** Twenty-nine (15%) of 191 A(H1N1)2009 viruses collected between May and September 2011 from a regional area near Sydney, Australia, contained the H275Y neuraminidase substitution responsible for oseltamivir resistance. Only one patient had received oseltamivir before influenza specimen collection. The resistant strains were genetically closely related, suggesting the spread of a single variant. Twenty-six (90%) of cases lived within a 50 kilometre radius, but the resistant variant was also detected up to 490 kilometres away. **Conclusions:** This cluster of cases represents the first non-institutional community transmission of H275Y oseltamivir-resistant A(H1N1)2009 influenza. These cases and preliminary data on the role of permissive mutations suggest that currently circulating A(H1N1)pdm09 viruses retain viral fitness in the presence of the H275Y mutation and that widespread emergence of oseltamivir-resistant strains may now be more likely.

**Epidemiological Methods for Investigating an Outbreak of Verocytotoxin-Producing *Escherichia coli* (VTEC) O157 PT 8 VT 1+2 with an Unusual Vehicle of Transmission**

**N. Launders**[1], M. Locking[2], M. Hanson[3], G. Willshaw[1], A. Charlett[1], R. Salmon[4], J. Cowden[2], G.K. Adak[1]; [1]Health Protection Agency, London, UK, [2]Health Protection Scotland, Glasgow, UK, [3]Scottish *E. coli* O157/VTEC Reference Laboratory, Edinburgh, UK, [4]Public Health Wales, Cardiff, UK.

**Background:** Between 1 Dec 2010 and 2 Feb 2011, 38 human VTEC O157 PT8 VT1+2 isolates were observed, around 5 times more than the same period of the previous 3 years. An outbreak control team was convened to investigate the cause. **Methods:** Cases were defined as UK residents with VTEC O157

PT8 VT1+2 infection of the outbreak profile (PFGE/MLVA) acquired in the UK after Nov 2010. A hypothesis of beef consumption was generated via a case-case analysis using HPA VTEC enhance surveillance system data. The initial case control study performed to test this provided inconclusive findings. Revised hypotheses were generated used a detailed trawling questionnaire and face-to-face interviews in cases homes. A case-control study was then performed to test the hypotheses of consumption or handling of potatoes, carrots or onions. In both case control studies controls were frequency matched on area of residence; age and sex being allowed for in the analysis. Exposures with odds ratios >1 and p-values <=0.2 in single variable analysis were included in a multivariable logistic regression model. Exposures that were not associated with illness were sequential removed. **Results:** Two hundred and fifty cases were reported from Dec 2010 to 4 Jul 2011; 75 hospitalisations, 4 cases of HUS and 1 death were reported. Cases were distributed across England, Scotland, and Wales. Most cases were female (70%) and adult (60%). The single variable analysis of the second case control study (30 cases, 62 controls) indicated exposures to several raw vegetables were associated with being a case; being in a household where either potatoes bought in sacks (OR 8.4, p=0.05), leeks bought loose (OR=33.2, p=0.009), raw turnips (OR=5.8, p=0.01), and raw swedes (OR=4.1, p=0.04). The first 3 of these remained associated in the multivariable model, together with chicken eaten away from the home. **Conclusions:** Investigation of a large national outbreak of VTEC O157 confirmed the hypotheses of exposure to raw vegetables revealing exposure to potentially soil bearing vegetables, and leeks in particular, as significantly associated with illness. Such vegetables may be stored for extended periods, and cross contamination is likely. With a common exposure such as vegetables detailed exposure information is required to identify the associations for what is an unusual source for this pathogen.

**Muscular Sarcocystosis in Travelers Returning from Tioman Island, Malaysia—2011**

**D.H. Esposito**[1], E.W. Lankau[1], F. von Sonnenburg[2], J.P. Cramer[3], M. Grobusch[4], H. Savini[5], C. Rapp[6], A. Pérignon[7], F. Gobbi[8], W. Ghesquiere[9], P. Zanger[10], A. Neumayr[11], Y. Michel[12], A.J. da Silva[1], P.P. Wilkins[1], B.M. Rosenthal[13], E. Aronica[14], D.A. Plier[15], G.W. Brunette[1], C.M. Brown[1], R. Fayer[13], M.J. Sotir[1], D.O. Freedman[15];
[1]Centers for Disease Control and Prevention, Atlanta, GA, USA, [2]Department of Infectious Disease and Tropical Medicine, University of Munich, Munich, Germany, [3]Bernhard-Nocht Clinic for Tropical Medicine, University Medical Center Hamburg-Eppendorf, Hamburg, Germany, [4]Center for Tropical and Travel Medicine, University of Amsterdam, Amsterdam, Netherlands, [5]Service de Pathologies Infectieuses et Tropicales, Hôpital d'Instruction des Armées Laveran, Marseille, France, [6]Service des Maladies Infectieuses et Tropicales, Hôpital d'Instruction des Armées Bégin, Saint-Mandé, France, [7]Service des Maladies Infectieuses et Tropicales, Hôpital Pitié-Salpêtrière, Paris, France, [8]Centro per le Malattie Tropicali, Ospedale Sacro Cuore-Don Calabria, Negrar (Verona), Italy, [9]University of British Columbia, Vancouver, BC, Canada, [10]Institut für Tropenmedizin, Tübingen, Germany, [11]Swiss Tropical and Public Health Institute, Basel, Switzerland, [12]Division of International and Humanitarian Medicine, Geneva University Hospitals, Geneva, Switzerland, [13]Animal Parasitic Diseases Laboratory, Agricultural Research Service, United States Department of Agriculture, Beltsville, MD, USA, [14]Department of (Neuro)Pathology, Academic Medical Center, University of Amsterdam, Amsterdam, Netherlands, [15]GeoSentinel Program Office, University of Alabama at Birmingham, Birmingham, AL, USA.

**Background:** *Sarcocystis* species are protozoan parasites that require a definitive predator and intermediate prey host to complete the lifecycle; humans can become accidental intermediate hosts by ingesting oocysts or sporocysts from feces of an infected definitive host. In October 2011, the GeoSentinel global surveillance system for travel-related morbidity detected an unusual cluster of myalgia and eosinophilia in travelers returning from Tioman Island, Malaysia. Sarcocysts were seen in two patients' muscle biopsies. **Methods:** Clinicians reporting patients with suspected muscular sarcocystis (MS) to GeoSentinel administered a travel and exposure survey to their patients and

completed medical chart abstractions. We defined a probable case of MS as travel to Tioman Island after March 1, 2011, eosinophilia (>5%), clinical or laboratory-supported myositis, and negative trichinellosis serology. A confirmed case met the probable case definition with histologic observation of sarcocysts in muscle tissue. **Results:** Data were received for 19 of 32 (59%) patients reported to GeoSentinel; 10 probable and 1 confirmed cases were determined. Among case patients, the median age was 46 years (range 21-59); 45% were male. All resided in Europe. All had mild to severe myalgia with onset a median of 11 days (range 2-39) after departing Tioman Island, marked eosinophilia (median highest value 14%, range 8-28%), and markedly elevated creatinine phosphokinase (median highest value 511 U/L, range 204-1435). All case-patients lodged on the northwest coast of Tioman Island, visited the beach, and swam in the ocean; none swam in fresh water. All consumed ice in beverages, 8 (73%) brushed teeth with tap water, and 7 (64%) ate fresh produce. Except for 45% reporting physical contact with cats, no other significant animal exposures were reported. **Conclusions:** GeoSentinel detected an unusual cluster of human MS in travelers returning from Tioman Island, Malaysia. We provide clinical data and possible exposure sources of this rarely documented parasitic disease in humans; infection may have occurred through local food, water, or environmental exposures while on the northwest coast of the island. Travelers to Tioman Island should ensure safe food and water consumption and practice proper hygiene to avoid exposure to animal feces.

**Meningitis Surveillance in Mali: Monitoring the Elimination of Epidemic Meningitis**

**S. Mandal**[1], S. Diarra[2], K. Touré[3], M.F. Maiga[3], K. Dao[2], N.M. Keita[3], R.T. Novak[1], F. Bougoudogo[2], T.A. Clark[1], N.E. Messonnier[1], M.N. Traoré[3];
[1]Centers for Disease Control and Prevention, Atlanta, GA, USA, [2]Institut National de Recherche en Santé Publique, Bamako, Mali, [3]Direction Nationale de la Santé, Bamako, Mali.

**Background:** Starting in 2010, countries in sub-Saharan Africa have been introducing a new serogroup A meningococcal conjugate vaccine to eliminate epidemic meningitis. Enhanced surveillance and laboratory confirmation are critical to evaluating vaccine impact and informing future vaccine programs. Mali, an early implementing country, completed vaccination of 1 to 29 year olds in three regions in December 2010 and the rest of the country in December 2011. **Methods:** We examined national population-based and reference laboratory surveillance data from January 2005 to March 2012 to estimate early vaccine impact on disease burden during the annual meningitis season (January through June). We assessed key elements of meningitis surveillance: data management, data quality, specimen collection and transport, and laboratory confirmation, to make recommendations for strengthening surveillance. **Results:** From 2007 through 2011, 3731 suspect meningitis cases were reported in Mali, and 2523 cerebro-spinal fluid specimens (68% of all notifications) were received at the reference laboratory. All 59 districts reported cases but 75% of specimens originated from early vaccinating regions. Districts did not routinely transmit case-level data or use standardized data tools. A causative pathogen was identified in 20% of specimens. Compared to six meningitis seasons prior to new vaccine introduction, population-weighted mean cumulative incidence of suspect meningitis in vaccinated regions decreased from 6.2 per 100,000 (2005 to 2010) to 3.0 per 100,000 (2011), ($p<0.05$), representing a relative risk reduction of 51% (95% CI 44%-58%). The proportion of serogroup A among meningococcal meningitis cases also declined from 95% (2007-2010) to 0% (2011), ($p<0.05$). **Conclusions:** Improvements in surveillance including laboratory confirmation have been sufficient to document vaccine impact in early implementing regions. The 2012 meningitis season is the first following national implementation of vaccination campaigns in Mali. Sustained efforts to strengthen surveillance in early implementing regions and enhanced efforts in the rest of the country are required to fully assess vaccine effectiveness and program impact.

## Strengthening Public Health Systems

Tuesday, March 13
5:00 PM – 6:00 PM
Grand Hall

**Board 223. Disease Control Programmes and Strengthening Public Health Care Services: Experiences from Pandemic Influenza Preparedness In Zambia**

**A. Theo**[1], S. wa Somwe[1], M.M. Cats[2], R. Masilani[3], M. Monze[1];
[1]University Teaching Hospital, Lusaka, Zambia, [2]US Centers For Disease Control and Prevention, South Africa, South Africa, [3]Ministry of Health, Lusaka, Zambia.

**Background:** In 2006 highly pathogenic avian influenza HPAI A H5N1 spread into Africa and human cases of H5N1 influenza appeared (Nigeria, Djibouti). National governments felt the need to increase preparedness for influenza pandemics. South-East Asia was experiencing increasing numbers of human H5N1 influenza cases with high case-fatality rates up to 60%. The experience of (re-) emerging infectious diseases, e.g. SARS (1993 SE Asia) and Ebola and novel arena virus hemorrhagic fever outbreaks in Africa, contributes to a critical need to reinforce outbreak preparedness and response capacity. **Methods:** The CDC developed a multi-discipline rapid response team (RRT) course for HPAI & pandemic influenza based on CDC/WHO/OIE/FAO principles of outbreak response. South Africa's National Institute for Communicable Diseases (NICD) Respiratory Virus Unit provided technical support and quality assurance to the national laboratory to diagnose influenza using rRT-PCR. The Surveillance for influenza-like illness (ILI) and severe acute respiratory illness (SARI) at sentinel hospitals for virological and clinical monitoring, was established coupled with development of laboratory diagnostic capacity. Training of hospital staff for Influenza Surveillance Systems (ISS) included PPE use, infection control and shipping of specimens. **Results:** A RRT training curriculum of 5 days was developed for multi-disciplinary teams. Joint RRT training for Animal and Human health (AHH) experts were established resulting in closer collaboration fitting the One Health concept. With support of CDC and the Inter-African Bureau for Animal Resources, at least 40 national and provincial outbreak response teams were trained. AI RRT training strengthened Public Health Capacity to organise response to outbreaks (measles, LUJO, H1N1 2009). NICD trained 12 scientists in rRT-PCR for influenza. There was national laboratory capacity and practices building, strengthened Lab-based health care services and research capacity, as well as south-south collaboration and Network building. ILI/SARI surveillance was established at 4 sentinel sites. **Conclusions:** The AI RRT training strengthened general outbreak response capacity and addressed a critical need to reinforce outbreak preparedness & response capacity in developing countries. Multi-disciplinary RRT boosted AHH collaboration. ISS allowed monitoring of circulating influenza viruses & related severity of illness & proved important during the H1N1 2009 pandemic. AI RRT training & ISS should be integrated into IDSR.

**Board 224. An Integrated Approach for Enhancing Capacity Building of Public Health Laboratories in Afghanistan**

**D.S. Elyan**;
US NAMRU-3, Cairo, Egypt.

**Background:** The devastated situation in Afghanistan involves all the aspects of humankind, including health sector. Recently, the U.S. government has instigated initiatives to help Afghanistan to enhance capacities in the area of public health. NAMRU-3 has utilized its unique expertise to take the leading role in this humanitarian mission. objectives: 1) Enhance CPHL and some regional lab capacities. 2) Expand lab network to provide surveillance and lab services in a range of disciplines. 3) Support disease surveillance system and basic diagnostic services. 4) Build capacity of lab staff, with the goal of

integrating all activities to help Afghanistan become an active member able to implement health regulations according to global mandates. **Methods:** With the financial support of government agencies, NAMRU-3 procured and provided lab essentials (supplies and equipment) that are required to enable lab training in serological, bacteriology and virology, vectors surveillance studies and implement safety and bio-safety standards. All required lab SOPs for QA/QC/GLP were developed, translated into local languages and addressed. A wide range of surveillance studies were developed to include vector born diseases, spectrum of AFI, diarrheal diseases, and ILI. NAMRU-3 assisted with outbreaks investigations such as Cholera, CCHF, viral hepatitis, pandemic Flu and wheat poisoning. In addition, NAMRU-3 also hosted training workshops for biomedical engineers on preventive maintenance. **Results:** In Kabul, lab capacity building at CPHL, KID, IGH, ANA, CVDRL, NMLCP labs have been provided and enhanced. Two hundred technical staff from different labs have attended multiple workshops and training sessions in Afghanistan, Egypt, Istanbul, Dubai and USA. Over 2000 clinical samples and arthropods have been collected for analyses. Pathogen discovery processes as well as measurement of respective immunological response have been accomplished. Over 500 microbial isolates were obtained and shared with reference labs for further characterization. **Conclusions:** NAMRU-3 has been able to develop core lab capacities at CPHL, KID, IGH, ANA, CVDRL and NMLCP. These efforts have helped APHI-MoPH to monitor, detect, identify, assess, contain, report, and respond to emerging public health threats.

**Board 225. Two Clusters of *Salmonella* Agona Infection Involving Multiple PFGE Patterns**

**S. Dalton**, W. Culpepper, A. Mba-Jonas, K. Neil;
Centers for Disease Control and Prevention, Atlanta, GA, USA.

**Background:** PulseNet is the molecular subtyping network composed of multiple local and state public health laboratories that identify enteric bacteria clusters through indistinguishable pulsed-field gel electrophoresis (PFGE) patterns uploaded to the national databases. Clusters are typically defined by higher-than-expected frequency of a single primary PFGE pattern uploaded in a given time frame. However, a few *Salmonella* outbreaks have been shown to include more than one PFGE pattern when other factors are included. **Methods:** Concomitant increases in multiple, unique *Salmonella* Agona PFGE patterns with slight pattern variations were noted and used to define two clusters: Cluster One in 2010 and Cluster Two in 2011. **Results:** Cluster One contained PFGE patterns: A(49 - number of infected patients), B(32), C(29), D(8), E(1), F(1) and G(1); Cluster Two contained PFGE patterns: A(80), B(16), G(15) and F(1). The source states, upload dates, and secondary enzyme PFGE results of these patterns were compared among the two clusters. Cluster One occurred June through August 2010 and had the most frequent uploads from CA(30), TX(29), and IL (22). Cluster Two occurred April through July 2011 and had the most frequent uploads from TX(27), IL (17), NY(8), CA (8), and GA(8). All isolates from both clusters yielded one indistinguishable secondary pattern. No specific food item was identified for Cluster One. Epidemiologic, traceback, and laboratory investigations linked Cluster Two to eating fresh whole papayas. Papaya PFGE matches to patterns A and G were recovered and uploaded by FDA. **Conclusions:** In this investigation, the evaluation of time frame of isolation, source states, and additional subtyping results strengthened the rationale for including multiple PFGE patterns at the initial cluster detection phase. Two of the PFGE patterns in Cluster Two were recovered from the single source of whole papaya. Therefore, surveillance for clusters of related infections using PFGE should take into consideration that multiple PFGE patterns may be associated with a single contaminant.

**Board 226. Evaluation of Publication Patterns and Attitudes Towards Evidence-based Medicine in Central Asia: Is Central Asia Benefiting from World Medical Science?**

**G. Yamshchikov**[1], G. Schmid[2];
[1]Vaccine Research Center, NIH, Bethesda, MD, USA, [2]Centers for Disease Control and Prevention, Almaty, Kazakhstan.

**Background:** In 1991, the five Republics of Central Asia (CA) (population, 60 million) inherited the Soviet health care system. While successfully providing basic health care, it was authoritarian, with little questioning of the *prikaz* (decree) that dictated medical practice. The Cold War ensured that the Soviet block had little exposure to the Western medical approach and reliance on the Russian language favored isolation. 20 years post-independence, our experience is that persisting barriers prevent full participation of CA in world medical science. To validate our experience, we evaluated literature use and attitudes towards evidence-based medicine (EBM) in medical science and healthcare in the region. **Methods:** We reviewed scientific publications using *PubMed,* searching literature 2009-June 2011 for "Asia, Central" as a MESH term or any country as a key word. To evaluate attitudes towards EBM, we interviewed 85 healthcare professionals, 12% of whom had a degree from a foreign English-speaking institution. **Results:** We found 345 publications, of which 146 (42%) had a CA first author, 104 (30%) had only foreign authors, and 95 (28%) had a foreign first author. Uzbekistan most commonly had a first author (N=77) and Turkmenistan least commonly (N=0). 260 (75%) articles were in English and 82 (24%) in Russian. Excluding papers with only foreign authors, Russian-language medical journals--*Med Parazitol* (N=21) and *Gig Sanit* (N=13)--were the most common journals, with *Biomed Central* the most common English language venue (N=9). Median impact factor of articles with a CA author was 2.40. No CA journal is indexed in *PubMed*. The Russian *Electronic Library of Science*, which catalogs Russian language publications, was incomplete and difficult to use. Of the healthcare professionals interviewed, 7% rated their English as proficient. Internet access at work was common (91%) and 33% use *PubMed.* Of interviewees, 88% thought EBM is important but only 50% thought it is used in healthcare decision-making and persons employed with international agencies or with Western educations were least likely to think so. **Conclusions:** Medical science and healthcare decision-making in CA are functioning without full benefit of EBM and the English-language medical science literature; we are reviewing ways to help overcome these impediments.

**Board 227. World Health Organization Global Foodborne Infections Network—Targets New Regions for Training on Enteric Diseases**

**H. Joshi**[1], E. Pun[1], B. Abela-Ridder[2], T. Chiller[1];
[1]CDC, Atlanta, GA, USA, [2]WHO, Geneva, Switzerland.

**Background:** The WHO Global Foodborne Infections Network (GFN) aims to build laboratory-based capacity for enteric diseases, through technical advice, reference laboratory support, training and encouraging collaborations between microbiologists and epidemiologists. Since 2000, GFN has held 80 courses that have included participants from over 150 different countries. One of the goals for GFN is to expand training in new locations and form new partnerships using platforms that have solid infrastructure to launch training courses. **Methods:** With the help of the CDC's Global Disease Detection (GDD) program two new training sites were launched in 2011: South Africa and India. The Southern Africa training took place in May 2011. This regional course was conducted at the new South Africa GDD site in Johannesburg at the National Institute for Communicable Diseases and was a collaborative effort between GFN and GDD. The training course brought together microbiologists and epidemiologists from 8 Southern Africa countries. Following the GFN course a PulseNet International training course for African countries with Pulse Field Gel Electrophoresis (PFGE) capabilities was conducted. Training for GFN in India did occur in March 2011 in Kolkata. GFN training at the GDD site in Delhi, India at the National Center for Disease Control (NCDC) is currently planned for January 2012. **Outcomes:** For the Southern Africa training course the curriculum focused on strengthening surveillance systems and writing project proposals. A total of 21 epidemiologists and microbiologists were trained. The course planned for the GDD India site will be for epidemiologists and microbiologists. This course will be a collaborative effort between GFN, GDD and NCDC in Delhi. **Conclusions:** GFN currently has 15 training sites and regional centers. Working with partners like GDD GFN aims to target new regions and

strengthen partnerships. Previously, Southern Africa and South Asia have not had GFN training sites. These new training sites will complement training sites in Africa and South East Asia and allow GFN to work towards fulfilling its vision where all countries have the capacity to prevent and control foodborne and other enteric infections.

## Bacterial/Viral Coinfections

Tuesday, March 13
5:00 PM – 6:00 PM
Grand Hall

**Board 228. Viruses Associated with Severe Acute Respiratory Infections in Children in Jingzhou City, Hubei Province in China**

**X. Huo**[1], L. Liu[1], B. Fang[1], F. Zhang[1], H. YU[2];
[1]Hubei CDC, wuhan, China, [2]Chinese Center for Disease Control and Prevention, Beijing, China.

**Background:** To determine the prevalence and identify the types of respiratory viruses causing infection in young children from population based surveillance for SARI and IPD in Jingzhou city, Hubei Province in China. **Methods:** NP/OP samples were collected from 214 children aged 0-5 years with severe acute respiratory infections enrolled as in-patients in four hospitals in Jingzhou from December 2010 to June 2011. Real-time RT-PCR was used to detect influenza virus (IFV), parainfluenza virus (PIV), adenovirus (ADV), respiratory syncytial virus (RSV ), and human metapneumovirus (hMPV). IFV and PIV were further subtyped to flu A/B and PIV-1- 2 or-3. **Results:** Out of 214 specimens, we identified 105 that contained at least one virus. We detected a virus in 42.55% (40/94) female and 54.17% (65/120) male specimens. ADV was the most dominant virus, present in 14.48% (31/214) followed closely by RSV 14.02% (30/214),PIV 13.55%(29/214). hMPV and IFV were less common with 7.01% (15/214) and 6.54% (14/214).Viral co-infections were detected in 11 specimens; ADV and hMPV were the m most common viruses involved (8 and 6 specimens respectively). RSV was the main pathogen in early winter, IFV was 32.26% (10/31) most common in early spring, and ADV was the main pathogen detected in May and June (18.92~22.22% positive). Over all, pandemic H1N1 was the main IFV detected (11 of 14 positive specimens) and PIV-3 was the main subtype of PIV infecting with young children (14 of 29 positive specimens). **Conclusions:** Our results indicate that nearly 50% specimens of children aged up to 5 years have a detectable respiratory viral pathogen; more attention should be paid to antivirual treatment for these cases. Adv, RSV and PIV were the main detectable causes of respiratory illness in these patients, but the dominant viral pathogen appeared to be seasonally dependent. Additional samples, spanning a longer time frame and more cases, will be useful to determine the true burden of respiratory virus infection on severe acute respiratory cases in Jingzhou.

**Board 229. The Burden and Epidemiology of RSV in Children Under 5 Years of Age in Rural Western Kenya**

**G. Bigogo**[1], D. Feikin[2], G. Jagero[1], M. Katz[3], J. Montgomery[3], R. Breiman[3], D. Burton[1];
[1]KEMRI/CDC, Kisumu, Kenya, [2]CDC, ATLANTA, GA, USA, [3]CDC, Nairobi, Kenya.

**Introduction:** Respiratory syncytial virus (RSV) is an important cause of lower respiratory tract illness in children worldwide. In rural Africa, the burden and epidemiology of RSV infection have not been well defined. **Methods:** As part of a longitudinal population-based infectious disease surveillance study, we collected combined nasopharyngeal/oropharyngeal (NP/OP) swabs from children <5 years presenting to a designated referral hospital in rural western Kenya with influenza-like illness (ILI), WHO-defined severe and very severe pneumonia, or any respiratory illness requiring hospitalization. Specimens were tested

for viral pathogens using real-time reverse transcriptase PCR assays. We calculated crude rates of RSV infection among children <5 years in the surveillance population and adjusted the rates accounting for health-seeking behavior and eligible children from whom samples were not collected. Among children with RSV infection, we compared hospitalization by age group using Chi-square tests. **Results:** Between January 2006 and August 2011, 7,411 sick visits with respiratory illnesses in children <5years were recorded at the study hospital. Of these, 2,653 (36%) had NP/OP swabs taken. RSV was detected in 377 (14%) of NP/OP specimens, - 109 (29%) of whom were infants and 116 (31%) from hospitalized children. The overall crude rate of RSV infection among children <5 years was 16.1 (95% confidence interval [CI] 16.0 - 16.1) infections per 1000 person-years of observation (pyo). The adjusted overall rate was 72.6 (95%CI 72.4 - 72.8) infections per 1000 pyo. By age group, the adjusted rates for children <12, 12 - 23 and 24 - 59 months were 101.9 (95% CI 101.4 - 102.6), 98.4 (95%CI 97.8 - 99.0) and 52.8 (95% CI 52.6 - 53.1) respectively. In the same age groups, 47%, 33% and 18% of RSV positive children were hospitalized respectively. Infants with RSV were three times as likely to be hospitalized compared to those aged ≥1 year (OR 3.18; 95%CI 1.77 - 5.71).Overall, 55% of RSV cases were treated with antibiotics. One child died within 30 days of RSV infection. **Conclusions:** Children under 5 years of age in rural Africa experience high rates of RSV infection with those under 2 years of age bearing the highest burden. Development and use of an RSV vaccine in sub-Saharan Africa could substantially reduce morbidity due to this disease.

**Board 230. Prevalence and Clinical Features of Influenza Virus Infections and Co-infections Involving Influenza and Other Respiratory Viruses in Kenya, 2006–2011**

**E. Lebo**[1], R. Ochola[2], N. Otieno[2], G. Emukule[1], G. Bigogo[2], R. Breiman[1], J. Mott[1], M. Katz[1];
[1]CDC-Kenya, Nairobi, Kenya, [2]KEMRI/CDC-Kenya, Nairobi, Kenya.

**Background:** Influenza and other viral respiratory pathogens contribute substantially to acute respiratory infections and although viral co-infections occur commonly, the clinical significance of these co-infections has not been defined. **Methods:** In 2006, the Kenya Medical Research Institute/ Centers for Disease Control and Prevention established surveillance for respiratory illness in two population-based surveillance sites, Kibera and Lwak; hospital-based surveillance was added in 2009 at Siaya District Hospital in Western Kenya. Nasopharyngeal (NP) and oropharyngeal (OP) swabs, along with clinical information, are collected from patients presenting with influenza-like-illness (ILI) or severe acute respiratory illness (SARI). NP/OP specimens are tested for 8 respiratory pathogens; Influenza A, Influenza B, Human Metapneumovirus, Adenovirus, Respiratory Syncytial Virus and Parainfluenza 1, 2, 3, using real time Reverse Transcriptase -Polymerase Chain Reaction. **Results:** Between October 2006 and January 2011, 8,986 respiratory specimens were collected from clinically attended SARI and ILI outpatients from Kibera and Lwak. Of these cases, 1,414 (15.9%) specimens were positive for influenza viruses; of these 459 (32.4%) were co-infected with another viral respiratory pathogen. The proportion of influenza viral co-infections was higher in children <5 years old, compared to 5-18 year olds (58.2% vs. 30.6%, p<0.01). From August 2009- January 2011, 2,716 (52.4%) specimens were collected from hospitalized SARI inpatients in Siaya. Of these cases, 205 (7.5%) specimens were positive for influenza virus; among the influenza- positive specimens 54 (26.3%) were co-infections with another respiratory virus. There was no difference in the mean duration of hospital stay (5.2 days vs. 5.0 days, P=0.6) or in frequency of presentation of severe respiratory symptoms (difficulty in breathing in all ages: nasal flaring, chest indrawing in children < 5 years) in the influenza-only inpatients compared to the co-infected patients. **Conclusions:** Viral co- infections occur commonly in patients with influenza in Kenya, especially among children <5 years old. The clinical relevance of co-infections is not clear; however, having influenza and another viral infection did not result in more severe respiratory illness.

## H1N1 Influenza

Tuesday, March 13
5:00 PM – 6:00 PM
Grand Hall

**Board 231. Influenza A Virus Neuraminidase Protein Enhances Cell Survival through Interaction with CEACAM6**

**S. Lal**[1], P. Gaur[1], J.R. Patel[2], S. Sharma[1], J.B. Bowzard[2], P. Ranjan[2], S. Gangappa[2], J.M. Katz[2], N.J. Cox[2], R.B. Lal[2], S. Sambhara[2];
[1]International Centre for Genetic Engg. & Biotechnology, New Delhi, India, [2]CDC, Atlanta, GA, USA.

**Background:** The influenza virus neuraminidase (NA) protein primarily aids in the release of progeny virions from infected cells. Here, we demonstrate a novel role for NA in enhancing host cell survival by activating the Src/Akt signaling axis via an interaction with carcinoembryonic antigen-related cell adhesion molecule 6, CEACAM6/CD66c (C6). **Results:** NA/C6 interaction lead to increased tyrosyl phosphorylation of Src, FAK, Akt, GSK3β and Bcl-2, which affects cell survival, proliferation, migration, differentiation and apoptosis. RNAi-mediated suppression of C6 resulted in a downregulation of activated Src, FAK, and Akt, increased apoptosis, and reduced expression of viral transcripts and proteins in influenza virus-infected lung epithelial cells. **Conclusions:** These findings suggest a previously unrecognized role for influenza NA in cell survival during viral replication.

**Board 232. Influenza Sentinel Surveillance in Lagos, South Western Nigeria, April 2010 – July 2011**

**O. Adeoye**[1], A. Aman-Oloniyo[2], O. Fawole[3], A. Adedokun[4], P. Nguku[1], C. Alake[5];
[1]Nigeria Field Epidemiology and Laboratory Training Programme, Abuja, Nigeria, [2]Lagos State Ministry of Health, Alausa, Ikeja., Nigeria, [3]Department of Epidemiology,Medical Statistics and Environmental Health,Faculty of Public Health, University of Ibadan, Ibadan, Nigeria, [4]Family Medicine Department, Lagos State University Teaching Hospital, Ikeja, Nigeria, [5]Lagos State University Teaching Hospital, Ikeja, Nigeria.

**Background:** Influenza is a disease that affects millions of people each year and contributes to the emerging threats of new strains of diseases worldwide. The first human cases of the pandemic Influenza A H5N1 and the novel Influenza A H1N1 first reported in Nigeria were seen in Lagos State in February 2007 and September 2009 respectively. There is paucity of information on the epidemiology of Influenza infections in Nigeria. This paper describes the characteristics of patients with ILI (Influenza like illness) and SARI(Severe acute Respiratory Illness), the strains and subtypes of Influenza virus responsible and the factors associated with these infections in Lagos State. **Method:** Lagos state is one of the four sites of the National Influenza sentinel surveillance (NISS) in the country. The first four patients who meet the case definition of ILI and all cases of SARI were recruited on the first four days of the week. Oropharyngeal and nasopharyngeal swabs are taken and sent to the National Influenza Research Laboratory for analyses. Data of cases seen between April 2010 and July 2011 were retrieved using the NISS Case Investigation Forms. Analyses was done using SPSS version 15.0 SOFTWARE. Statistical significance was set at P&lt0.005. **Results:** A total of 646 cases were enrolled. The mean age of participants was 18.63(SD 20.91).Males constituted 50.5 %( 326). ILI cases were 68.6%(443).The mean age of ILI cases was higher than SARI cases(21.66yrs{ SD21.37} vs. 11.99yrs{ SD 18.24} t= 5.583, p=0.000 ). A significantly (p=0.03) higher proportion of males (35.3%) compared with females (27.5%), had SARI .Influenza virus was responsible for 2.8 %(18) of all ILI and SARI cases consisting of pandemic flu A HINI 0.8%(5),seasonal flu A H3 0.8%(5),Influenza A Unsubtypable 0.2%(1) and Influenza B 1.1%(7). All cases of Pandemic flu A HINI were seen between March and April 2011 and only caused SARI. There was no statistical difference between the mean temperatures and ages of patients with SARI from Pandemic flu

A/HINI and those from other causes. **Conclusions:** Influenza contributed to respiratory morbidities in Nigeria including the novel H1NI1. This has implications for management of ILI and SARI by physicians especially at non sentinel sites. There is the need to design influenza vaccine in Nigeria.

**Board 233. Epidemiological and Clinical Characteristics of an Influenza A (H1N1) 2009 Outbreak in a Primary School: Vietnam Post Pandemic in 2010**

**K.C. Nguyen**[1], B.V. Nguyen[2], D.T. Tran[2], L.N. Vu[2], H.T. Nguyen[1], D.N. Tran[1];
[1]National Institute of Hygiene and Epidemiology, Hanoi, Viet Nam, [2]Department of Preventive Medicine, Ministry of Health, Hanoi, Viet Nam.

**Background:** A new pandemic influenza virus was first reported in North America in April 2009 and spread globally. Vietnam was the 54th country reporting pandemic case on May 31, 2009. Pandemic virus spread to all provinces in Vietnam, reached its peak in September 2009, and then was dominated by seasonal influenza B starting in December 2009. Monitoring epidemiological changes of this pandemic influenza virus play important role to pandemic preparedness. However, outbreaks caused by A(H1N1) 2009 continued to occur. Influenza cases were reported in a primary school in Haiphong city north of Vietnam in January 2010. This study describes the epidemiological and clinical aspects of an outbreak in a primary school. **Methods:** A prospective cohort study investigated cases by standardized data collection form. A clinical case was defined as a patient with fever and cough or sore throat. All clinical cases had throat swabs tested for influenza by RT-PCR. **Results:** The outbreak lasted from January 12_25, 2010, and included 103 clinical cases. Sixty-four (62%) of 103 cases were positive for influenza A(H1N1)2009 virus by RT-PCR. The attack rate was 25%, in girls and boys were 28% and 23%, respectively. All age groups were affected, attack rates of grade 1, 2, 3 and 4 was, 35%, 20%, 14% and 40.9%, respectively. All cases reported mild acute upper respiratory infection. Common clinical symptoms were fever (100%), sore throat (97%), and runny nose/nasal congestion (86%). Cough was reported in only 20% of cases. Other clinical signs such as fatigue and headache were rare, and no cases reported digestive disorder. None was hospitalized. **Conclusions:** Pandemic influenza A circulating at low level caused sporadic outbreaks. Attack rate of this virus remained as same as it observed during the first wave in 2009. Epidemiology and clinical symptoms of pandemic influenza were not changed while circulating in community in post pandemic in 2010.

**Board 234. Influenza Disease Burden in a Rural Setting in India, 2009–11: Integrating Epidemiologic, Virologic, and Demographic Surveillance to Estimate the Population-based Incidence of Hospitalized Influenza**

**S. Hirve**[1,2], M. Chadha[3], P. Lele[1], S. Sambhudas[1], A. Deoshatwar[1,3], S. Juvekar[1], K. Lafond[4], R. Lal[5], A. Mishra[3];
[1]KEM Hospital Research Center, Pune, India, [2]Dept. Public Health & Clinical Medicine, Umea University, Umea, Sweden, [3]National Institute of Virology, Pune, India, [4]Centers for Disease Control and Prevention, Atlanta, GA, USA, [5]Centers for Disease Control and Prevention, New Delhi, India.

**Background:** Influenza disease burden estimates are essential for health policy decisions. Influenza is a common vaccine-preventable viral disease that can cause severe or fatal disease, however its burden is less well known in tropical settings. Prospective surveillance was established to estimate the population-based incidence of hospitalized cases with influenza in a rural community in western India. **Methods:** We conducted active surveillance for eligible patients hospitalized with acute medical illnesses or an acute exacerbation of chronic medical conditions from May 2009 - April 2011. Participating sites included all 55 private and public health care facilities in Vadu Demographic Surveillance Area (DSA), Pune. Nasal and throat swabs were tested for influenza viruses using real time RT-PCR. A community-based health utilization survey estimated the proportion of acute respiratory infection patients being admitted to hospitals within and outside the Vadu DSA. Annual incidence was stratified by age and virus

sub-type. **Results:** Among 9,426 total hospitalizations, 3291 (35%) eligible patients were enrolled. Of these, 667 (20%) patients were positive for influenza virus; 340 (51%) for pandemic 2009A/H1N1 (pH1N1) and 327 (49%) for seasonal influenza viruses, including A/H3 (109; 16%), A/H1 (20; 3%) and influenza B (198; 30%). The proportion of eligible patients who were influenza-positive peaked at 39% in August 2009 and 42% in August 2010, though influenza was present to a varying extent through the study period. The annual incidence of influenza was significantly higher in May 2009 - April 2010 than during May 2010 - April 2011 (4.37 vs 3.78/1000, p=0.031). The incidence of pH1N1 did not differ in 2009-10 and 2010-11 (2.03 and 2.17, respectively). The incidence of both pH1N1 and seasonal hospitalized influenza disease was highest in the 5-29 year olds. Overall, 7.1% (667/9426) of all hospital admissions were influenza-positive (19.6% during peak influenza activity). **Conclusions:** Our study quantifies the high burden of hospitalized influenza in a rural population in India. Influenza activity peaked in August of both 2009 and 2010, although it was present year round. Integrating hospital-based and health-utilization information is an efficient approach to estimate population-based disease burden.

**Board 235. US Military Experience with Influenza in 2008–2011: Analysis of Illness Severity and Risk Factors**

**S.-E. Lee**[1], A. Eick-Cost[1], M. Johns[2], D. Blazes[1], J. Brundage[1], J. Sanchez[1];
[1]Armed Forces Health Surveillance Center, Silver Spring, MD, USA, [2]US Department of Health and Human Services, Washington, DC, USA.

**Background:** During the 2009 H1N1 influenza pandemic an estimated 35 million individuals between the ages of 18-64 in the US were infected and among these, an estimated 160,000 individuals were hospitalized. Since the pandemic, several risk factors for hospitalization have been identified in the general US population. The US military population is unique from the US general population in that service members must meet certain physical standards and medical readiness. To better understand risk factors for influenza hospitalization in the US military, we describe the clinical and epidemiological characteristics of active component service members hospitalized during any of the past three influenza seasons. **Methods:** The surveillance population included all active component service members who were hospitalized or who sought ambulatory care for influenza during any of the three influenza seasons: August 1, 2008-March 31, 2009, April 20, 2009-March 31, 2010, and August 1, 2010-March 31, 2011. An influenza event was defined as a diagnosis with an ICD-9-CM code of 487 or 488 in any of the available diagnostic positions. All data were obtained from the Defense Medical Surveillance System. **Results:** During the 2009-10 season, a total of 24,538 non-hospitalized and 395 hospitalized cases were reported. In the 2008-09 and 2010-11 seasons, a total of 47 and 79 hospitalized cases were reported, respectively. The distribution of hospitalized cases by age group was similar across all three seasons; the majority of illnesses (47-66%) occurred among 17-24 year olds. A larger proportion (42%) of 2009-10 hospitalized cases had pre-existing medical conditions compared to 2008-09 hospitalized cases (28%). Using the number of hospitalized days as a surrogate of illness severity, the median number of days hospitalized for all three seasons was the same, 3 days (IQR 2, 5). **Conclusions:** Although the 2009-10 season experienced a high burden of disease compared to the 2008-09 and 2010-11 seasons, the risk factors for hospitalization that were observed in the US general population during the H1N1 influenza pandemic were not found to be risk factors in the US military. The use of medical administrative records may introduce misclassification due to miscoding errors. Additional studies using laboratory confirmation and chart reviews may improve identification of US military risk factors.

## Novel Agents of Public Health Importance

Tuesday, March 13
5:00 PM – 6:00 PM
Grand Hall

**Board 236. The Epidemiologic Investigation of Severe Fever with Thrombocytopenia Syndrome in Hubei Province, China, 2010**

**X. Guan**, L. Liu, X.-S. Xing, X.-F. Shen, J.-Q. Xu, J.-L. Yue, X.-X. Huo, H.-X. Wu, S. Sha, F.-X. Zhan;
Hubei Center for Disease Control and Prevention, Hubei, People's Republic of China, Wuhan, China.

**Backgroud:** To identify the epidemic characteristics and risk factors of an emerging infectious disease, the Severe Fever with Thrombocytopenia Syndrome (SFTS) in Hubei province in order to provide scientific evidence of control and prevention. **Methods:** Active surveillance on SFTS isprocessed in monitoring sites that were set up in hospitals above township level (included) in Suizhou, Huanggang and Wuhan between January and December, in 2010. Specific surveillance on SFTS is launched across the Province in hospitals above county level (included). Cases that match the definition of surveillance case are identified and reported to CDCs. Cases were interviewed and their blood samples were collected and detected in the laboratory (PCR and virus isolation). We also conducted serum antibody surveys among healthy population and livestock and vector tick surveillance in high-epidemic areas. **Results:** 188 cases that match the definition of surveillance case and 21 deaths are reported by 11 cities, 32 countries and 100 towns in 2010 (the incidence rate is 0.33/106; the fatality rate is 11.2%). 68.6% (129 cases) cases' samples were collected and detected 67.4% (87 cases) positive by Real time-PCR for SFTS virus; an elevation in antibody titer by a factor of four or seroconversion was observed in 11 patients; SFTS virus were isolated from 2 patients. The total antibody positive rate was 3.8% in healthy population, and 55%, 36.7% and 80% respectively in dogs, sheep and cows. The ticks from grass, cattle and sheep were detected positive by Real time PCR. The epidemiologic analysis showed that the patients were from hilly areas at an elevation between 28-940 meters. The epidemic time is between April and December with peak from May to September. The youngest case is 11-year old, while the eldest is 81-year old. The median age is 56-year old. 95.3% patients are farmers. All Patients didn't have travel history two weeks before onset of SFTS. 93.8% patients engaged in works associated with agriculture. 52.8% patients had exposure of the ticks. 22% patients were bit by ticks. And skin injury is found in 64.2% patients. **Conclusions:** Most cases of SFTS and their livestock in Hubei are infected by SFTS virus. Ticks may be one important vector. Skin injury, contact with or bit by ticks may be risk factors.

**Board 237. Serological Assessment for the Evidence of Henipavirus Exposure in Cattle and Goats**

**S. Chowdhury**[1], A. Mikolon[1,2], G. Crameri[3], J.H. Epstein[4], M. Salah Uddin Khan[1], A. Islam[1], J. Barr[3], P. Daszak[4], L.-F. Wang[3], S.P. Luby[1,2];
[1]International Centre for Diarrheal Diseases Research, Bangladesh (ICDDR,B), Dhaka, Bangladesh, [2]Centers for Disease Control and Prevention (CDC), Atlanta, GA, USA, [3]CSIRO Australian Animal Health Laboratory (AAHL), Geelong, Australia, [4]EcoHealth Alliance, New York, NY, USA.

**Background:** Nipah virus (NiV) is an emerging zoonotic infection that has caused 152 human cases in Bangladesh from 2001 to 2011 with a case fatality of over 75%. Drinking contaminated raw date palm sap has been repeatedly identified as a vehicle of NiV transmission in humans. Date palm sap spoiled by bat feces is occasionally fed to cattle. Two outbreak investigations identified an association between close contact with sick or dead cattle and goats and human illness. However, the role of cattle or goats for NiV transmission is not clear. We conducted a cross-sectional study to look for evidence of NiV antibodies in cattle and goats. **Methods:** We sampled 400 cattle and 400 goats living within a 1500

meter radius from *Pteropus* fruit bat roosts located near 5 previous human NiV outbreak areas. We collected a blood sample from each animal. We interviewed owners on animal characteristics, husbandry, and exposure to bats. The sera were tested at the Australian Animal Health Laboratory using a Luminex-based multiplexed microsphere assay that specifically detects antibodies to henipaviruses. Positive samples were further tested by serum neutralization test (SNT) and Western blot (WB) against the NiV N (nucleocapsid) protein. **Results:** Nine (2.3%) cattle and two (0.5%) goats were positive by the Luminex assay. All of these sera were collected from animals with no apparent clinical signs of illness. The prevalence ratio (PR) for NiV positivity was higher in cattle and goat having a history of feeding on fruits partially eaten by bats or birds (PR=3.3, CI: 1.1-10, p=0.03). Cattle and goat with NiV positivity were more likely to have a history of drinking raw juice made from Asian palmyra palm fruit partially eaten by bats (PR=4.9, CI: 1.03-23.2, p=0.04). All Luminex positive sera tested negative by SNT. However, 2 Luminex positive cattle sera also tested positive by WB. **Conclusions:** The laboratory results do not confirm NiV infection, but do suggest possible infection by either novel or existing henipaviruses in cattle and goats in Bangladesh. The association of positive Luminex assays in cattle with exposures to bat contaminated foods suggests that the source of this virus is likely *Pteropus* bats. Further research to characterize this virus may identify a novel organism of public health importance.

**Board 238. The Re-emergence and Changing Epidemiology of Coccidioidomycosis, United States, 1998–2010**

**K. Benedict**, B.J. Park;
Centers for Disease Control and Prevention, Atlanta, GA, USA.

**Background**: Coccidioidomycosis is a sporadic infection caused by inhalation of airborne spores from *Coccidioides spp.*, soil-dwelling fungi endemic to the southwestern United States. Infection is costly and often debilitating, with nearly 75% of patients requiring absence from work or school and over 40% requiring hospitalization. **Methods**: To characterize long-term trends in rates of coccidioidomycosis, we analyzed data from CDC's National Notifiable Disease Surveillance System (NNDSS), which compiles information on selected infectious conditions from state and territorial surveillance systems. Crude, sex-specific, age-specific, and age-adjusted incidence rates (aIR) were calculated for Arizona (AZ), California (CA), and other endemic states (ES) combined (Nevada, New Mexico, Texas, and Utah) using U.S. Census Bureau estimates. **Results**: During 1998-2010, a total of 89,187 coccidioidomycosis cases were reported from 25 states and the District of Columbia. Nationwide, case reports increased from 2,275 in 1998 to 16,810 in 2010. In AZ, cases increased from 1,474 in 1998 (aIR 29.5/ 100,000) to 11,887 in 2010 (aIR 182.0 / 100,000), a 517% increase; CA cases increased from 719 in 1998 (aIR 2.1 / 100,000) to 4,630 in 2010 (aIR 12.3 / 100,000) (+279%). Cases in other ES combined increased from 74 (aIR 0.3 / 100,000) to 159 (aIR 0.5 / 100,000) (+68%). Cases outside endemic areas increased from 8 in 1998 to 134 in 2010. 2010 incidence in AZ was highest among persons aged ≥ 60 years (IR 290.0/ 100,000), although the largest increase was among those aged 5-19 years (1,123% increase since 1998). An increasing proportion of AZ cases were female (43% in 1999 vs. 58% in 2010). 2010 incidence in CA was highest among the 40-59 age group (IR 16.6/ 100,000), although the largest increase was among persons under 20 (+683% from 1998 to 2010). **Conclusions:** Since 1998, coccidioidomycosis incidence has increased substantially in the U.S, particularly among young persons in AZ and CA. Clinicians should be aware of the re-emergence of coccidioidomycosis, and research in methods to reduce morbidity is needed.

**Board 239. *Clostridium difficile* Infection in the Pediatric Population of Monroe County, New York**

**R.C. Smith**, S. Rhee, D. Nelson, G. Dumyati;
University of Rochester, Rochester, NY, USA.

**Background:** Since the emergence of the BI/NAP1/027 C*lostridium difficile* strain, an increase in *C. difficile* infections (CDI) in hospitalized children and severe disease acquired in the community with no

known exposure to healthcare or antibiotics has been reported. We performed surveillance in Monroe County, NY to describe the incidence, epidemiology and clinical characteristics of CDI in children. **Methods:** Active population surveillance for CDI was conducted between Oct 2009 -Sept 2011. An incident case was defined as a positive *C. difficile* stool assay >8 weeks after previous positive specimen, in a resident of the surveillance area who is ≥1 year old. Only loose stool were tested by toxin or molecular assay. A case report form was abstracted from patient records. CDI cases were classified as either community (CA) or healthcare associated depending on prior healthcare exposure (i.e. hospitalization or LTC residency in prior 12 weeks). Interviews were completed for a subset of the cases to confirm CA status. Only one incident case per patient was included in this analysis. Thirty stool samples were submitted to reference laboratories for culture and molecular characterization of isolates. **Results:** During the 2 years, 2913 CDI cases were identified; 100 cases were in children less than 18 years with a yearly incidence of 31.4 cases per 100,000 population. The median age was 5 years (SD 5.7); 25% of the cases were in the age group 12-23 months. The majority of cases (78%) were CA, 44% had an underlying medical condition, 55% received antibiotics and 13% received proton pump inhibitors in the two weeks prior to CDI. The 12-23 month age group were more likely to be CA CDI compared to the 2-17 year age group (92% vs. 72%, p=0.04). No significant differences in exposures to antibiotics, proton pump inhibitors or the presence of underlying medical conditions were noted between the two groups. Only 3 children were hospitalized due to CDI. Of the 30 stool samples cultured, 27 (90%) grew *C. difficile* and 8 (30%) were NAP1. **Conclusions:** In our population, the incidence of CDI in children is low; the disease is mild and commonly acquired in the community. A higher proportion of cases occurred in the age group 12-23 months. Further investigation of the risk of disease in this population and in children with no traditional CDI risk factors is warranted.

**Board 240. A Comparison of Viral Fitness and Virulence between Emergent Human Adenovirus 14p1 and Prototype 14p Strains**

**B. Anderson**, K. Barr, G. Heil, J. Friary, G. Gray;
University of Florida, Gainesville, FL, USA.

**Background**: The last decade has seen the emergence of a new strain of human adenovirus 14, the prevalence of which has significantly increased across the United States. Studies have suggested that the morbidity and mortality of this new strain of adenovirus, classified as HAdV-14p1, is greater than other, more prevalent, adenovirus strains. Patients have experienced more severe symptoms such as acute febrile respiratory illness, gastroenteritis and pneumonia, as well as the appearance of a greater incidence of mortality, especially in adolescent and elderly populations. Molecular analysis identified a two amino acid deletion on the third knot of the fiber gene of HAdV-14p1 as compared to prototype HAdV-14p. No studies, however, have been conducted to identify how this mutation affects the fitness and virulence of HAdV-14p1. **Methods**: In this study, we conducted in vitro and molecular assays used to evaluate growth kinetics, cellular infectivity, and cytotoxicity of HAdV-14p1, comparing it to the classical prototype strain HAdV-14p. **Results**: For growth kinetics, data show no viral replication at 30°C and minimal differences in viral replication at 37°C for both strains. Cellular infectivity data show both strains having the ability to propagate in a diverse array of cell lines, human lung and kidney cell lines having the highest potential for propagation. Cytotoxicity data indicates differences in cellular distress induced by both strains of virus in the first 8 hours, but similar cell viability levels between 8 and 48 hours. **Conclusions**: Collectively, the data from all of these assays indicate that other factors, such as individual host response, are likely contributing to an increase in virulence and additional studies are necessary to further explore these areas.

## Foodborne and Waterborne Infections

Tuesday, March 13
5:00 PM – 6:00 PM
Grand Hall

**Board 241. Effects of a Food-borne Outbreak on the Integration of a Previously Rare Serotype of Salmonellosis into a Community in New York State, 2001–2010**

**J. Karr**[1], D. Schoonmaker-Bopp[2], C. Hidalgo[3], S. Solghan[4], G. Smith[5], K. Burzlaff[1], S. Zansky[4];
[1]New York State Department of Health, Rochester, NY, USA, [2]New York State Department of Health-Wadsworth Center, Albany, NY, USA, [3]New York State Department of Health, Buffalo, NY, USA, [4]New York State Department of Health, Albany, NY, USA, [5]New York State Department of Health, Geneva, NY, USA.

**Background:** *Salmonella* is a common cause of food-borne illness, accounting for roughly 40,000 US cases per year. Of more than 2,000 serotypes, *S. anatum* had been considered rare in New York State (NYS). In September 2004 a *S. anatum* outbreak occurred in western NYS (wNYS) and was associated with the consumption of improperly handled roast beef at a Firemen's Carnival. The incidence rate of *S. anatum* was examined following the outbreak to determine if a change in serotype distribution occurred in wNYS. **Methods:** Salmonellosis is a reportable disease in NYS and all *Salmonella* isolates identified at local laboratories are forwarded to the NYS Wadsworth Center for confirmation, serotyping and pulsed-field gel electrophoresis (PFGE). All cases are interviewed with a standardized questionnaire, with data entered into an electronic database. Laboratory and interview data were analyzed for cases residing in 17 counties of wNYS from 2001-2010. **Results:** From 2001-2003 *S. anatum* rates averaged 0.04/100,000. During the outbreak year (2004), the rate peaked to 1.24/100,000. The rate remained higher in 2005 (0.46/100,000), with 92% of cases diagnosed within the first half of the year, and all exhibiting either the outbreak PFGE pattern or one other pattern. Rates declined in 2006 to 0.07/100,000. A small cluster of cases occurred in 2007, raising the rate to 0.32/100,000, but a common link was never identified. Rates declined since 2007, averaging 0.02/100,000 from 2008-2010. Specimen source varied by year; 91% of isolates were from stool in 2004, and 69% of isolates were from urine in 2005. **Conclusions:** The 2004 outbreak involving contaminated roast beef contributed to the emergence of *S. anatum* in wNYS. Person-to-person transmission, urinary tract colonization, and environmental exposures are possible reasons for the persistence of disease months after the contaminated food item was eliminated. Despite changes in PFGE patterns, when a rare organism or species is introduced into an environment where it was relatively nonexistent it can disrupt the ecological balance and have lasting effects. *S. anatum* rates returned to pre-outbreak levels, but this pattern of disease occurrence illustrates the importance of maintaining surveillance and molecular characterization of strains for monitoring trends and epidemiological changes.

**Board 242. Antimicrobial Resistance Among *Salmonella* Strains That Caused Outbreaks, United States, 2004–2008**

**J.L. Reynolds**[1,2], R. Rickert[1], J. Grass[1,2], K. Joyce[1], L.H. Gould[1], K.M. Herman[1], J. Castleman[1], F. Medalla[1];
[1]Centers for Disease Control and Prevention, Atlanta, GA, USA, [2]Atlanta Research and Education Foundation, Decatur, GA, USA.

**Background:** Investigations of foodborne outbreaks of *Salmonella* infections provide information about the major food vehicles. Testing outbreak isolates for antimicrobial susceptibility can help determine which food vehicles are commonly associated with resistant strains. We described antimicrobial resistance among *Salmonella* outbreaks. **Methods:** Local, state, and territorial health departments reported *Salmonella* outbreaks to CDC through the Foodborne Disease Outbreak Surveillance System.

CDC categorized foods implicated in outbreak reports into 17 commodities. States submitted outbreak isolates to the National Antimicrobial Resistance Monitoring System for susceptibility testing to 15 agents using broth microdilution. We linked outbreak data to isolate resistance data using a combination of variables including outbreak identification number, state, year, month, and serotype. We used the PulseNet-assigned *Xba*I pattern and cluster code to validate isolates as being part of an outbreak. **Results:** During 2004-2008, 592 non-typhoidal *Salmonella* outbreaks with known serotype information were reported and 484 outbreak isolates were received. We linked 103 (17%) outbreaks to 402 (83%) outbreak isolates. Among the 47 outbreaks attributed to a single food commodity, 8 (17%) were caused by a resistant strain. Four (50%) of these 8 were caused by strains that were resistant to ≥5 agents. Six (75%) of 8 outbreaks caused by resistant *Salmonella* and 22 (56%) of 39 outbreaks caused by pansusceptible strains were attributed to land animal commodities (e.g., beef, poultry, eggs, dairy) (p=0.44). **Conclusions:** A somewhat higher proportion of outbreaks caused by resistant than pansusceptible *Salmonella* were attributed to land animals. However, the small proportion of outbreaks that had isolates submitted for antimicrobial resistance testing indicates that those studied may not be representative of all outbreaks. Obtaining antimicrobial resistance data on all *Salmonella* outbreak isolates and collecting this information in outbreak reports will inform analyses of associations between commodity groups and resistance.

### Board 243. Emergence of Multi Drug Resistant *Vibrio cholerae* from Nepalgunj Outbreak and Different Hospitals of Nepal

**D.R. Bhatta**[1], E.D. Khanal[1], G. Shakya[2], B. Upadhyay[3];
[1]Tribhuvan university, Kathmandu, Nepal, [2]National public Health Laboratory, Kathmandu, Nepal, [3]National Public Health Laboratory, Kathmandu, Nepal.

**Background:** Cholera outbreak still remains a major health problem during the summer and monsoon season in Nepal. **Methods:** The Present laboratory based study isolated multidrug resistant *Vibrio cholerae* from the stool samples from patients with diarrheal symptoms in Nepal in the monsoon season in 2010. In all 240 stool samples 44 stool samples were collected from one of the city known as Nepalgunj during an epidemic outbreak of cholera and 196 stool samples received at National public health laboratory and processed for the presence of *Vibrio cholere.* **Results:** In all 48 *Vibrio* strains isolated 20 isolates from outbreak samples and 28 from hospital samples were identified by biochemical test, serotyping and biotyping. All isolates were *Vibrio cholerae* O1 serotype and Ogawa biotype El Tor. All strains were sensitive to Ceftriaxone, Cefotaxime, Amikacin and Gentamicin. Many strains were resistant to Ciprofloxacin, Ofloxacin, Ampicillin, Tetracycline, Chloramphenicol and Erythromycin and All strains were resistant to Nalidixic acid, Cotrimoxazole and Furazolidone. **Conclusions:** The reduced susceptibility to the tetracycline and other antibiotics was observed among the isolates from epidemic outbreak area. The MIC value for Ampicillin, Nalidixic acid, Tetracycline, Ciprofloxacin,and Chloramphenicol 15.17 mg/L, 42.67mg/L, 4.16 mg/L, 0.28mg/L and 1.42 mg/L respectively as tested by microbroth dilution test.

### Board 244. The Protective Effects of Acquired Immunity on the Population Dynamics of Human Campylobacteriosis

**A.H. Havelaar**[1,2], A.N. Swart[1], M. Tomasi[2], M. Kretzschmar[1,3], O. Diekmann[2];
[1]RIVM, Bilthoven, Netherlands, [2]Utrecht University, Utrecht, Netherlands, [3]University Medical Center, Utrecht, Netherlands.

**Background**: For many infectious diseases, the benefit of becoming infected is in subsequent immunity. When immunity is waning, it may be boosted again by re-infection, usually entailing little or no clinical symptoms. We investigate such situations when the force of infection is considered as a constant. A concrete 'realization' is found for foodborne diseases, such as campylobacteriosis and salmonellosis.

Here, the force of infection derives from bacterial growth in animal reservoirs which are transmitted to humans by a myriad of pathways, with meat and other products of animal origin as the dominant pathways. **Methods**: We assume that all individuals are born susceptible (S), and when exposed may become (asymptomatically) infected with force of infection λ, and a probability π of additionally developing symptomatic illness. An infected individual is fully protected (P) against subsequent infection. The waning of immunity is represented by transitions from P to a state of partial protection (Q) with rate α and then back to S with rate γ. When a partly protected individual is exposed, the immune status is boosted to full protection (Q -> P) without clinical illness. We define R as the number of S -> P transitions in a lifetime, with πR resulting incidents of clinical illness. **Results**: We show that R assumes a single maximum at intermediate values of λ. Parameter estimates are difficult to obtain and we explore a wide range of values. Based on volunteer experiments, we assume that α = 13 and γ = 1.1 per year. Based on exposure modelling we assume λ = 1 per year in industrialized countries. Then, R = 37 infections per lifetime. As the risk of clinical illness is approximately 1 per 200 years, we find that π ≈ 0.01, much lower than observed in volunteer experiments. This suggests that π is strongly dose-dependent and that most exposures are at low doses that do not cause disease but do boost immunity. A maximum for R is found when λ = 3.6 and R decreases at higher values of λ. In developing countries, λ is expected to be beyond the maximum for R. Here, reducing the force of infection may lead to an increase in the incidence of disease. **Conclusions**: Acquired immunity may profoundly influence the epidemiology of foodborne diseases. Not accounting for these effects may result in biased estimates in epidemiological studies as well as risk assessment studies.

**Board 245. Foodborne Disease Outbreaks in Hospitals, 1998–2008**

**A.L. Nisler**[1,2], T. MacCannell[2], B.J. Silk[2], L.H. Gould[2];
[1]Atlanta Research and Education Foundation, Atlanta, GA, USA, [2]Centers for Disease Control and Prevention, Atlanta, GA, USA.

**Background:** Foodborne outbreaks in hospitals have potentially serious consequences because of the possibility of exposing vulnerable patient populations. **Methods:** Local, tribal, and state public health departments voluntarily report foodborne disease outbreaks to the Centers for Disease Control and Prevention (CDC) Foodborne Disease Outbreak Surveillance System. We reviewed data for foodborne outbreaks during 1998-2008 in which food had been prepared or consumed in a hospital. Etiologic agents, food commodities, and locations where foods were prepared were analyzed. **Results:** A total of 63 foodborne outbreaks were reported, leading to 1,280 illnesses and three deaths. The average outbreak size was 20 illnesses (range: 2-238). The implicated food was prepared inside the hospital in 26 outbreaks (41%). In the remaining 37 outbreaks (59%), food was prepared in a restaurant or deli (20 outbreaks, 54% of 37), by a caterer (14, 35%), or another location (3, 9%). A suspected or confirmed etiology was reported for 44 (70%) outbreaks, of which norovirus was the most common etiology (59%), followed by *Clostridium perfringens* and *Salmonella* (9% each). Other etiologies were scombroid toxin, *Staphylococcus*, *Listeria monocytogenes*, *E. coli* O157:H7, and an unreported bacterial toxin. Norovirus (35%) and *Salmonella* (100%) outbreaks commonly resulted from food prepared within the hospital, while a caterer was the most common food preparation setting for *Clostridium perfringens* outbreaks (50%). All three deaths occurred during one listeriosis outbreak due to tuna salad both prepared in a hospital and consumed by hospitalized patients. Of the 17 outbreaks in which the implicated food could be assigned to a single commodity, the most frequent commodities reported were fish and leafy vegetables (24% each) and pork and poultry (18% each). **Conclusions:** Food prepared outside hospitals contributed to the most foodborne disease outbreaks. Although a food source accounts for a minority of all norovirus outbreaks in healthcare, this pathogen was the most common due to food prepared in the hospital and the most commonly reported pathogen overall. Hospitals should ensure that policies and

procedures for safe food sources and handling practices are followed especially when food is prepared outside of the hospital, which may help reduce morbidity and mortality in a vulnerable population.

**Board 246. Risk Factors for Aseptic Meningitis Due to Echovirus 19 Infection in a Town, Jiangsu Province, 2011**

**X. Li**[1], K. Xu[2], R. Zu[2], D. Wen[1], F. Tang[2], T. Shen[1];
[1]Chinese Field Epidemiology Training Program, Beijing, China, [2]Jiangsu Center for Disease Control and Prevention, Nanjing, China.

**Background:** On May 20, 2011, the health center in Town F reported that many children were diagnosed with aseptic meningitis since May 10. We investigated this outbreak to indentify the scope of the outbreak, pathogen, and risk factors of the event, and recommend control measures. **Methods:** We defined a suspected case as onset of fever (≥37.5°C), headache, plus nausea or abnormal electroencephalogram during January 1- May 31, 2011. A confirmed case was a suspected case with positive pathogen detection from patients' specimens. We searched for cases by checking medical records from 2004 to 2011 in main hospitals of Yancheng Prefecture and the health center at Town F. We conducted a case-control investigation in the primary school with highest number of cases to evaluate exposures in relation to disease onset. We collected environmental samples and, from cases, throat swab, spinal fluid, and stool specimens for enterovirus isolation and phylogenetic analysis. **Results:** Cases during January-May, 2011 exceeded the 2004-2010 background by 8 times. 88 cases (11 confirmed) were registered in May 2011 in Town F, with the peak being in mid-May. The attack rate was nearly four times as high in children aged 7-14 years (2.1%) as in children aged ≤6 years (0.57%) (RR=3.7, 95% CI=2.3-6.1), and nearly twice as high in boys (1.7%) as in girls (0.93%) (RR=1.8, 95% C =1.2-2.8). Case-patients did not cluster geographically by residence. The pathogen had a 96% homology with Echovirus19 enterovirus. 74% of case-patients did not always wash hands before meals compared to 19% of control-students (OR=10; 95% CI=3.5-29); 48% of case-patients shared hand towels, compared to 11% of control-students (OR=5.7; 95% CI=1.8-18). **Conclusions:** This aseptic meningitis outbreak was caused by Echovirus 19 and was mainly transmitted person-to-person among children with poor personal hygiene. We proposed surveillance and measures to improve personal hygiene.

## New Challenges for Old Vaccines

Tuesday, March 13
5:00 PM – 6:00 PM
Grand Hall

**Board 247. Effects of Gastroenteritis Episodes on Maintenance of Polio Vaccine Titers in Children Three Years and Under in Rural Coastal Kenya**

**A. LaBeaud**[1], P. Mungai[2], G. Gildengorin[1], E. McKibben[3], M. McKibben[3], C.L. King[3], I. Malhotra[3];
[1]Children's Hospital Oakland Research Institute, Oakland, CA, USA, [2]Ministry of Health, Msambweni, Kenya, [3]Case Western Reserve University, Cleveland, OH, USA.

**Background:** Evidence shows that infants with concurrent gastroenteritis (GE) are less likely to respond to oral polio vaccination than those without gastroenteritis. Our objective was to determine whether further episodes of gastroenteritis in the first three years of life had an effect on maintenance of polio titer. **Methods:** Children enrolled in a birth cohort in rural coastal Kenya received four trivalent polio vaccinations before 6 months of life. Sera were then drawn at 6 month intervals until age 36 months and polio titers were measured using poliovirus IgG ELISA kits. GE episodes were documented during scheduled follow-up visits and at any time of illness during the 3 year period. Student's t-test was

performed to compare those with and without GE at each time point. **Results:** Of 545 children in the study, 159 had 246 episodes of gastroenteritis in the first three years of life. GE episodes were more likely to occur between 6 and 18 months of life. The range of GE episodes per child was 0-4. Polio titers did not significantly differ between children with and without GE from 6 to 36 months of age. **Conclusions:** Although concurrent gastroenteritis may hamper immune response to oral polio vaccine, further episodes of gastroenteritis after the vaccination series do not appear to alter the maintenance of polio titers.

**Board 248. Modeling the Risk of Importation of Measles into the Americas**

**G.L. Armstrong**[1], A.E. Barskey[1], S. Redd[1], K.M. Khan[2], N.M. Gallagher[1], G. Wallace[1];
[1]Centers for Disease Control and Prevention, Atlanta, GA, USA, [2]University of Toronto, Toronto, ON, Canada.

**Background:** The Americas Region is currently reviewing data to document measles elimination in the region, and if successful, will be the first in the world to do so. While elimination implies that transmission is not sustainable, importations will continue as long as measles is present elsewhere. In 2011, the United States received more measles importations than in any year since 1996. **Methods:** Measles importation risk during a recent 5-year period (2006-10) was modeled from the reported incidence of measles in countries outside of the Americas during those years (source: WHO) and on the number of commercial airline travelers arriving from those countries during 2009 (source: IATA). A simple model was developed, which assumed a risk of measles in travelers equal to the reported incidence in their country of departure and an average measles incubation period of 10 days. US-specific estimates from the model were compared with actual importations. **Results:** In 2009, 59.9M travelers arrived by air in the Americas from outside of the region. The destination for 69% of these was the United States (USA), 12% Canada (CAN), and 19% Latin America and the Caribbean (LAC). Taking account of reported measles in the countries of departure, the model estimated 34.7 importations into the Americas per year during the 5-year period: 69% to USA; 15% to CAN; and 16% to LAC. During this time, US surveillance detected 27.6 importations per year, similar to the modeled risk of 23.8 per year. For USA during this time period, source-country-specific importation risks from the model correlated well with the actual number of importations attributed to specific countries ($p<0.0001$). The model suggests that importations into USA and CAN should be relatively more sensitive to measles trends in Asia (proportion of itineraries from WHO Western Pacific and Southeast Asian Regions: USA, 32%; CAN, 33%; LAC, 7%) whereas LAC should be more sensitive to trends in Europe (itineraries from WHO European Region: USA, 63%; CAN, 55%; LAC, 89%). **Conclusions:** Results of this simple model correlated well with the epidemiology of measles importation into the United States. The model suggests that the United States has the greatest risk of measles importation in the region, with a modeled number of importations greater than that of the rest of the region combined.

## Vectorborne Diseases and Climate Change

Tuesday, March 13
5:00 PM – 6:00 PM
Grand Hall

**Board 249. Detection of Alpha Viruses in Mosquitoes from Semi Arid Areas of Kenya**

**E. Chepkorir**[1], J. Lutomiah[2], J. Awino[1], A. Makio[3], H. Koka[3], F. Mulwa[1], L. Musila[3], R. Sang[1];
[1]International Center for Insect Physiology and Ecology (ICIPE), Nairobi, Kenya, [2]Kenya Medical Research

Institute (KEMRI), NAIROBI, Kenya, [3]United States Army Medical Research Unit-Kenya (USAMRU-K), Nairobi, Kenya.
**Background:** Alphaviruses are a diverse group principally mosquito-borne RNA viruses that cause diseases in humans worldwide. They include Chikungunya, O'nyong-nyong and Sindbis viruses (SINV) that cause febrile illnesses with encephalitis or arthritis. These viruses are of significant public health concern with Venezuelan equine encephalitis virus having the potential to be weaponised. To determine the presence and circulation of alphaviruses and the associated vector species responsible for their maintenance and transmission, a surveillance study was undertaken in two semi arid regions of Kenya. **Methodology:** Mosquitoes were trapped using $CO_2$-baited CDC light traps from December 2009 to June 2010 in 6 selected sites in Ijara and Marigat districts during the wet seasons. Mosquitoes were morphologically identified to species, pooled to 25 mosquitoes per pool and homogenized in minimum essential medium using copper beads. The homogenates were clarified by centrifugation at 12,000 rpm and the supernatants inoculated in monolayers of VERO cells in 24 well plates. The cultures were incubated at 37$^{\circ}$C and observed daily for cytopathogenic effects (CPE).Cultures showing CPE were harvested and viruses identified by RT-PCR and sequencing. **Results:** Over 92,000 mosquitoes were collected, identified into 37 species and pooled into 4,382 pools. Eleven Ndumu virus (NDUV) isolates were obtained from pools of *Ae. mcintoshi* (7), *Ae. ochraceus* (1) and *Ae. tricholabis* (2) all collected from Ijara and *An. pharoensis* (1) from Marigat. Semliki forest virus (SFV) was isolated from *Ae. ochraceus* (3) and *Ae. tricholabis* (2) from Ijara and one isolate of SINV from *Cx. antenattus* from Marigat. **Conclusions:** This study shows that SFV, SINV and NDUV are circulating among mosquito species in the two semi arid regions of Kenya and could account for some of the febrile illnesses of unknown etiology observed in these areas. SFV was detected in Ijara while SINV was found in Marigat, which have high densities of *Ae. ochraceus* and *Cx. antennatus* respectively. Therefore control strategies to prevent alphavirus transmission should be designed to target the mosquito species.

**Board 250. An Island-Wide Dengue Epidemic Demonstrates That DENV-1 Is a More Common Cause of Illness upon Primary Infection than DENV-4—Puerto Rico, 2010**

**T.M. Sharp**, A. Rivera, R. Rodriguez-Acosta, J.L. Munoz-Jordan, E. Hunsperger, L.M. Santiago, H.S. Margolis, K.M. Tomashek;
Centers for Disease Control and Prevention, San Juan, PR, USA.
**Background**: Dengue, a potentially fatal febrile illness caused by four mosquito-transmitted dengue viruses (DENV-1-4), is endemic in Puerto Rico. In January, 2010, the number of suspected dengue cases reported to the Puerto Rico Department of Health/CDC passive dengue surveillance system exceeded the epidemic threshold. To characterize this epidemic, surveillance data were used to describe all reported cases. **Methods**: Suspected cases were patients with a serum specimen submitted by a health care provider for dengue testing. Laboratory-positive cases had (i) DENV identified via reverse transcriptase polymerase chain reaction (RT-PCR) in an acute specimen taken ≤ 5 days after symptom onset; and/or (ii) anti-DENV IgM detected in a convalescent specimen taken > 5 days after symptom onset. Individuals with primary DENV infection lacked detectable anti-DENV IgG antibody in an acute specimen. **Results**: In 2010, 23,622 suspected dengue cases were reported, of which 10,956 (46.4%) were laboratory-positive. Of 7,424 RT-PCR-positive specimens, DENV-1 (69.0%) and DENV-4 (23.6%) were detected more frequently than DENV-2 (7.3%) and DENV-3 (<0.1%). Of all laboratory-positive cases, nearly half (46%) were adults ≥ 20 years of age, 4,173 (66.0%) were hospitalized, 3,773 (34.4%) had at least one hemorrhagic manifestation, and 254 (2.3%) met the 1997 WHO definition of dengue hemorrhagic fever. Enhanced fatal case surveillance detected 39 laboratory-positive dengue deaths, 89% of which were in adults. From a sample of 787 acute specimens, 21% were primary infections; 1-4 year olds were the only age group for which primary DENV infection was more common than secondary infection. Individuals infected with DENV-1 were 3.9 (95% confidence interval, 2.3-6.6) times more likely

to be experiencing a primary infection than those infected with DENV-4. **Conclusions**: These results demonstrate that DENV-1 is a more common cause of primary infection among individuals reported as a suspected dengue case than is DENV-4, and that a decrease in the frequency of primary DENV infections may be driving increases in disease severity in adults. Overall, the 2010 epidemic was long in duration, high in magnitude, and resulted in the most dengue-related deaths since surveillance for dengue began in Puerto Rico in the late 1960's.

**Board 251. West Nile Virus Infection in Greece: Two Years of Transmission (2010–2011)**

**K. Danis**[1], A. Papa[2], A. Baka[1], E. Papanikolaou[1], G. Theocharopoulos[1], M. Detsis[1], I. Terzaki[1], G. Dougas[1], C. Politis[3], S. Gewehr[4], S. Mourelatos[4], C. Dovas[5], A. Tsakris[6], N. Vakalis[7], S. Bonovas[1], G. Saroglou[1], T. Panagiotopoulos[7];
[1]Hellenic Centre Disease Control and Prevention, Athens, Greece, [2]2.Reference Laboratory for Arboviruses, First Department of Microbiology, Medical School, Aristotle University of Thessaloniki, Thessaloniki,, Greece, [3]Haemovigilance Coordinating Centre, Athens, Greece, [4]Ecodevelopment, SA, Thessaloniki, Greece, [5]Laboratory of Microbiology, Faculty of Veterinary Medicine, Aristotle, University of Thessaloniki, Thessaloniki, Greece, [6]7.Department of Microbiology, National and Kapodistrian University of Athens, Athens, Greece, [7]National School Public Health, Athens, Greece.

**Introduction:** In August 2010 the first human cases of West Nile virus (WNV) neuro-invasive disease (WNND) were reported to the Hellenic CDC, while the virus continued to circulate during 2011. WNV infection in humans had not been documented in Greece before 2010. **Methods:** Physicians in Greece were asked to notify confirmed or probable cases of WNV infection according to the EU case definition. Surveillance in equidae, birds and mosquitoes was enhanced. An action plan for protecting the blood system against WNV was implemented. **Results:** In 2010, 262 laboratory diagnosed cases of WNV infection (197 WNND and 65 WN fever) including 33 WNND-related deaths (CFR17%). Most cases resided in wetland areas in the Region of Central Macedonia. The incidence (16.3 per 100,000 population) in rural areas was 2.4 (95%CI 1.7-3.2) times higher than that in urban areas. Of the 8,261 blood donors tested with single unit NAT, four (1:2,065) were positive. Fourteen horses with neurologic signs were positive for WNV. Culex pipiens was predominant among captured mosquitoes. Lineage 2 WNV was detected from three mosquito pools, one blood donor and two magpies. This strain was highly homologous to one isolated in a goshawk in Hungary in 2004. Between 16 July and 05 October 2011, 93 cases of WNND were reported from five regions in Greece. Of these, 46 occurred in districts that had not been affected in 2010. Lineage 2 WNV sequences were again obtained from one pool of Culex pipiens mosquitoes and from seroconverted sentinel chickens in Central Macedonia. **Discussion:** The 2010 outbreak was the largest reported in the EU in the last 15 years. In 2011 the virus seems to disperse southward to newly affected areas, in a pattern similar to California, which has comparable climate to Greece. The CFR of the 2010 outbreak was higher than that reported in Lineage 1 outbreaks in North America. The amino acid substitution H249P detected in the Greek strain, which is a suspected virulence marker in lineage 1 strains, might be associated with increased virulence. The reoccurrence of human cases in two consecutive years and the spread of the virus in new areas suggest that WNV is established in Greece, and its transmission may continue to occur in the future. Changing ecological parameters and climate may influence the WNV transmission cycle.

## Novel Surveillance Systems

Tuesday, March 13
5:00 PM – 6:00 PM
Grand Hall

**Board 252. A Novel Surveillance System for Person-to-Person Enteric Disease Outbreaks, United States**

**M.E. Wikswo**, A.J. Hall;
Centers for Disease Control and Prevention, Atlanta, GA, USA.

**Background:** Approximately 179 million cases of acute gastroenteritis (AGE) occur in the United States every year, and the majority of AGE outbreaks are thought to result from direct person-to-person transmission. Although CDC has conducted surveillance for foodborne and waterborne outbreaks for over 40 years, there was no national surveillance for AGE outbreaks resulting primarily from person-to-person transmission before implementation of the National Outbreak Reporting System (NORS) in 2009. **Methods:** Local, state, and territorial public health agencies report AGE outbreaks, defined as ≥2 illnesses linked to a common exposure, to CDC via the NORS web interface. We analyzed all finalized 2009 and 2010 NORS reports of outbreaks with a primary mode of transmission of person-to-person. **Results:** A total of 2183 person-to-person AGE outbreaks occurring in 2009-10 were reported in NORS from 41 states and Washington, D.C. These outbreaks resulted in 77,829 reported illnesses with an estimated 1867 hospitalizations and 172 deaths. Of the 1393 outbreaks with a reported etiology, 1225 (88%) were either confirmed or suspected to be caused by norovirus. Other frequently reported etiologies included *Shigella* (n=91), *Salmonella* (n=14), Shiga toxin-producing *Escherichia coli* (n=13), and rotavirus (n=10). Most outbreaks due to norovirus or an unknown etiology occurred during the winter months, while outbreaks due to *Shigella* or another etiology most often occurred during the spring or summer months. Of the 1128 outbreaks with a reported setting, nursing homes and other long-term care facilities were the most frequently identified (78%), followed by daycares (6%), hospitals (4%), and schools (4%). **Conclusions:** This novel surveillance system provides the first national data on AGE outbreaks spread through person-to-person transmission, highlighting the significant burden they represent. Norovirus is the most frequently reported cause of these outbreaks and, based on epidemiologic characteristics, may be responsible for a significant portion of the reported outbreaks of unknown etiology. As enhancements to NORS are introduced, state participation in NORS may increase, allowing for improved estimation of the burden of epidemic person-to-person AGE that can guide development of targeted interventions.

**Board 253. Understanding Lyme Disease Surveillance in Maryland, 2009**

**H.J. Rutz**[1], S.B. Wee[1], M.E. Brett[2], A.B. Kay[2], A.F. Hinckley[2], K.A. Feldman[1];
[1]Maryland Department of Health and Mental Hygiene, Baltimore, MD, USA, [2]Centers for Disease Control and Prevention, Fort Collins, CO, USA.

**Background:** Maryland ranks in the top ten states for reported Lyme disease (LD) cases; it is likely that LD in Maryland is underreported as a result of many surveillance factors. Positive laboratory results are reported to the state's 24 local health departments (LHDs) who investigate and classify LD cases as Confirmed, Probable, Suspect or "Not a Case" in the state surveillance database. In 2009, 4,768 LD reports were in the state database; 2,029 (43%) were Confirmed or Probable. To better understand the burden of LD and LD surveillance and to explore alternative surveillance approaches, we conducted a survey of LHDs and sought additional billing codes and medical chart data for a sample of cases. **Methods:** In July 2011, LHDs completed an online survey about LD surveillance, including number of reports not entered in the state database, staffing availability, and investigational approach. Healthcare Providers (HCPs) were requested to supply billing codes for a 10% random sample of reported LD cases

(N=474) from 2009. The predictive value of specific codes was evaluated. A medical chart review (N=149) was conducted on all sampled Suspect and select Not a Case reports for additional clinical and diagnostic data. The additional data was used, when available, to reclassify cases as Confirmed and to calculate a more accurate estimate of LD cases for the state. **Results:** LHDs did not enter an additional 5,722 LD reports in the state database during 2009. Seven (29%) LHDs lost LD surveillance staff in the past 2 years; one lost all staff and does not currently investigate LD. In 2008, 16 (75%) LHDs investigated each LD report while 5 (21%) investigated only if sufficient laboratory evidence of infection. By 2011, 10 (42%) LHDs investigate LD reports only if sufficient evidence. LHD staff make 2 (range 1-4) attempts to contact HCPs to investigate reports. LHDs expressed concerns about burden of LD surveillance with fewer resources and the utility of LD surveillance. The billing code assessment and medical chart review are ongoing. **Conclusions:** In 2009, there were 120% more LD reports in Maryland than had been entered in the state database. Resources for LD surveillance vary by LHD, but have diminished overall. LHDs question the utility of LD surveillance and are eager for alternative approaches, such as querying billing databases.

**Board 254. Cievs Net: System of Alert and Response to Outbreaks and Epidemics in Brazil**

**P.B. Magalhães**, E.T. Masuda, S.L. Dias, G.S. Dimech, T.B. Gomes, R.D. Machado, W.A. De oliveira;
Brazilian Ministry of Health, Brasilia, Brazil.

**Background:** With the aim of increasing the capacity of surveillance and response to public health risks in Brazil, the Center for Strategic information and Response to Public Health Emergencies -CIEVS of the Brazilian Ministry of Health was launched in 2006. This Center has the purpose of developing the Focal Point activities of Brazil for the International Health Regulation (2005) of the World Health Organization. This strategy was extended to the States and cities (capitals) and today is known as “CIEVS Net” - Net of Strategic Information and Response in Health Surveillance. This study aimed describe the CIEVS and CIEVS Net as a System of Alert and Response to Risks and Emergencies in Public Health in Brazil. **Method:** A descriptive study of CIEVS Net organization in Brazil. **Results:** Internationally, the CIEVS is a Center for Emergency Operations - COE. Brazil was the third country in the Americas to organize its Center. Currently, CIEVS has 51 centers distributed among 27 states and 26 capitals destined to: detect public health emergencies; continuously assess health problems that may constitute public health emergencies; and manage, coordinate and support responses developed in emergency situations. The CIEVS in the Ministry of Health and CIEVS Net (States and cities/capitals) resulted in the detection and monitoring of 745 events, from March 2006 to December 2010. The mean and median of time between the date of the beginning of the event or the beginning of the symptom(s) of the first case(s) and the date of the notification receipt by CIEVS was 17 and 7 days, respectively, ranging from 0 to 366 days (whooping cough in indigenous people in Amapá in 2009). As to the time of opportunity of finishing the monitoring of an event by CIEVS, the mean and median was 39 and 19 days, respectively, ranging from 0-328 days (rhabdomyolysis in Amazonas in 2008). **Conclusions:** As CIEVS Net advances in the establishment of its labor processes, providing the centers at the different government spheres in Brazil with physical, structural and technological support, we note an improvement in the activities of epidemiological surveillance and especially in the context of detection, notification and appropriate monitoring of public health risks and/or emergencies.

**Board 255. Online Reporting for Public Health Surveillance Using Micro-Monetary Incentives**

**R. Chunara**, V. Chhaya, S. Bane, S.R. Mekaru, E.H. Chan, C. Freifeld, J.S. Brownstein;
Harvard Medical School, Boston, MA, USA.

**Background:** To investigate the use of novel surveillance tools, we harnessed online reporting for participatory epidemiology. Specifically, we investigated the use of a participatory system for gathering information about malaria spread directly from the public in a malaria endemic region where prevalence

information is limited. Individuals in India were incentivized to self-report their recent experience with malaria by micro-monetary payments. **Methods:** Self-reports about malaria diagnosis status and related information were solicited online via Amazon's Mechanical Turk. Responders were paid $0.02 to answer survey questions regarding their recent experience with malaria. We examined the data over time and compared to other metrics about temporal progression of the epidemic. The study was conducted in summer 2010 during a malaria outbreak in Mumbai and expanded to other cities during summer 2011. **Results:** Timing of the peak volume of weekly self-reported malaria diagnosis in 2010 was comparable to other available metrics such as the volume of media over time and information about the epidemic from media sources. Additionally, distribution of Plasmodium type diagnosis through self-report in 2010 revealed 59% for *P. vivax,* which is comparable to literature reports of the burden of *P. vivax* in India (between 50 and 69%). Self-reported *P. falciparum* diagnosis was 19% and during the 2010 outbreak and the estimated burden was between 10 and 15%. Prevalence between 2010 and 2011 via self-reports decreased significantly from 36.9% to 19.54% in Mumbai ($p = 0.001083$), and official reports also confirmed a prevalence decrease in 2011. **Conclusions:** With careful study design, micro-monetary incentives and online reporting are a rapid way to solicit public health information. This type of methodology provides a cost-effective way of executing a field study that can act as a complement to traditional public health surveillance methods, offering an opportunity to obtain information about malaria activity, timing, demographics affected or specifics such as Plasmodium diagnosis type at a finer resolution than official reports can provide. The recent adoption of technologies such as the Internet supports self-reporting mediums, and self-reporting should continue to be studied as it can foster preventative health behaviors.

**Board 256. Crimean Congo Hemorrhagic Fever Surveillance in Kazakhstan, 2009–2010**

**B. Knust**[1], Y. Bumburidi[2], Z. Medetov[3], K. Ospanov[4], S. Nichol[1];
[1]Centers for Disease Control and Prevention, Atlanta, GA, USA, [2]US Centers for Disease Control and Prevention, Almaty, Kazakhstan, [3]Ministry of Health, Shymkent, Kazakhstan, [4]Ministry of Health, Astana, Kazakhstan.

**Background:** Crimean Congo Hemorrhagic Fever (CCHF) virus is a tick-borne pathogen that causes hemorrhagic fever symptoms with high fatality in hospitalized patients. CCHF is endemic in Kazakhstan, with most cases occurring in the Southern Kazakhstan Oblast (SKO) region. Surveillance activities in this region included reporting of suspect and confirmed CCHF cases, and a tick bite registry. We summarized the available surveillance data for CCHF, and made modifications to the existing surveillance system to capture more cases and better characterize the risk of tick bite as a means of CCHF transmission. **Methods:** The national case definitions, testing protocols, and case report forms for suspect CCHF were reviewed, and recommendations were made with the goal of maximizing detection of CCHF cases. Ticks were collected from persons developing fever following a tick bite. Line lists of case-patients and weekly summaries of tick bites reported in SKO were analyzed to describe the spatial and temporal incidence of tick bites and CCHF. **Results:** Weekly total reported tick bites correlated significantly with weekly CCHF occurrence (2009 $r = 0.58$, $p = 0.002$; 2010 $r = 0.60$, $p < 0.001$). Additionally, the incidence of tick bites was significantly higher in municipalities reporting CCHF cases than in those with no CCHF cases ($p = 0.01$). **Conclusions:** In conclusion, our analysis of CCHF surveillance data in Kazakhstan found a high number of reported tick bites, with spatial and temporal association between tick bites and CCHF cases. Tick bite reporting is a surveillance technique not frequently used in CCHF epidemiology, but has great potential to provide useful information about virus transmission and disease ecology.

## Geographic Information Systems (GIS)

Tuesday, March 13
5:00 PM – 6:00 PM
Grand Hall

**Board 257. Historical Compilation and Georeferencing of Dengue and Chikungunya Outbreak Data for Disease Modeling**

**A. Anyamba**[1], K.M. Collins[1], J. Small[1], A.D. LaBeaud[2], L.J. Sutherland[3], E. Mohareb[4], K.J. Linthicum[5], C.J. Witt[6];
[1]NASA Goddard Space Flight Center, Greenbelt, MD, USA, [2]Children's Hospital & Research Center Oakland, Oakland, CA, USA, [3]Case Western Reserve University, Cleveland, OH, USA, [4]Navy Medical Research Unit-3, Cairo, Egypt, [5]USDA Center for Medical and Veterinary Entomolgy, Gainesville, FL, USA, [6]Uniformed Services University of the Health Sciences, Silver Spring, MD, USA.

**Background:** The risk of vector-borne disease spread is increasing due to significant changes and variability in the global climate and increasing global travel and trade. Understanding the relationships between climate variability and disease outbreak patterns is critical to the design and construction of predictive models. Disease vector data are spotty and disease outbreak data are often incomplete and hard to access. In an attempt to establish better outbreak data records, we present a compilation of georeferenced reports of chikungunya and dengue outbreak locations for Eastern Africa, the Middle East, South Asia and Southeastern Asia. **Methods:** Published works such as journal articles, books, and newspaper archives, as well as web-searching tools were thoroughly exploited to find village-level dengue and chikungunya case locations throughout history (1635- present). Web-based sources that provided data were: the World Health Organization, the Centers for Disease Control and Prevention, the Travel Health website of Hong Kong, the National Institutes of Health, ProMed mail, Reliefweb and Flutrackers websites. Additional data was obtained from various surveillance studies. Each case location was geo-coded using GIS mapping software by identifying the latitude and longitude location by place name. **Results:** We present the results of geo-coded case location on maps from the early 1600s to the present. In general the distribution maps indicate an increase in the number of outbreak locations over time. This may be a result of improved disease surveillance, case detection, better diagnostics and documentation. The reliability of these data is significantly higher since the 1990s. **Conclusions:** The compiled data are a strong beginning at compiling comprehensive georeferenced records for modeling purposes. However, problems associated with this methodology of data mining for case data include: 1) more frequent and accurate disease case reporting in recent history due to improved surveillance; 2) government policies discouraging accurate and timely reporting of case data; and 3) case underreporting in remote areas. Nevertheless, such a database is crucial to the study of disease-climate relationships, and ultimately to disease prevention.

**Board 258. Nontuberculous Mycobacteria: Reports of Clinical Laboratory Isolation in a Three County Area, North Carolina, 2006–2010**

**E. Hilborn**[1], G. Smith[2], M.S. Murphy[3], K. Messier[2], E. Hudgens[1], A. Ghio[1], J.-M. Maillard[4], S. Pfaller[5], J. Stout[6];
[1]US Environmental Protection Agency, Research Triangle Park, NC, USA, [2]UNC Chapel Hill, Chapel Hill, NC, USA, [3]Computer Sciences Corporation, Research Triangle Park, NC, USA, [4]North Carolina Division of Public Health, Raleigh, NC, USA, [5]US Environmental Protection Agency, Cincinnati, OH, USA, [6]Duke University Medical Center, Durham, NC, USA.

**Background:** Laboratory reports of mycobacteria isolation and identification are created during the clinical diagnostic process to differentiate *Mycobacterium tuberculosis* from nontuberculous

mycobacteria (NTM). Numbers of NTM isolations are expected to exceed true infections. **Methods:** We collaborated with North Carolina tuberculosis control personnel and diagnostic laboratories to identify and collect reports of NTM isolation among residents of three counties during 2006 - 2010. Data included: patient age, gender, race, NTM species, anatomic site of isolation, and location of patient residence when present in the report. We performed descriptive analyses and calculated cumulative incidence rates (CIR) of isolation by county, race, and age group using 2010 US Census denominator data. We mapped patient residence to evaluate environmental risk factors for infection. **Results:** We identified 559 individuals with at least one report of NTM isolation, 50% were female, 64% white; 22% African American and 14% 'other' race. Five year CIR per 100,000 were higher among white females than white males, however African American and 'other' race males had higher rates of isolation than females. Median age of isolation was 56 years (range: <1- 107 years). Isolations from anatomic sites included: 415 (74%) from respiratory specimens; 40 (7%) from sterile sites or blood culture; 30 (5%) from dermal sites; and 74 (13%) from 'other' sites. Of 505 NTM isolates identified to species or complex, 371 (74%) were slow growing NTM, and 302 (60%) were *M. avium* complex. The 5 year CIR of isolations per 100,000 persons varied among counties; county 'A': 24.4; county 'B': 11.5; county 'C': 5.4. The 5 year CIR of isolations among all ages were 8.6 / 100,000 persons, and increased with age, ranging from 2.5 for those less than 20 years, to 27.5 for those persons aged 60 and older. **Conclusions:** Gender-specific NTM isolation rates appear to be modified by race, although numbers for nonwhites were low. Slow-growing NTM isolations predominated. Isolations were more frequent among older persons. Isolation rates varied among counties, with higher rates in a county with less favorable environmental conditions for NTM occurrence suggesting a potential diagnostic bias. This abstract is of a proposed presentation and does not necessarily reflect EPA policy.

**Board 259. Analysis of Risk Area for Malaria in Military Area of Operations (AOs) along Thai–North Cambodia Border Utilizing Environmental Database and Geographic Information System**

**J. Gaywee**, N. Kuttasingkee;
Armed Forces Research Institute of Medical Sciences, Bangkok, Thailand.

**Background:** Malaria remains a major cause of disease non battle injury (DNBI) for Thai troops deployed to border areas. To decrease this DNBI, appropriate malaria prevention and control program specific to high risk areas is crucial. **Methods:** Geographic Information System (GIS) was applied to gain information of risk areas for malaria infection in provinces along Thai-North Cambodia borders. Environmental data corresponding to malaria vector such as forest area, area attitude, natural water resource for mosquito bleeding, human community as well as mosquito flying range was utilized to conduct an analysis. **Results and Conclusions:** Resulting computational simulation demonstrated that areas at the border line of Srisakate and Ubonratchathani Provinces have the highest suitable environmental factors facilitating for malaria infection, thus, the high risk areas for malaria infection. However, computational analysis also indicated that the shift and change of risk areas may occur if other factors such as migration of human host and parasite reservoirs as well as public health behavior are taking into account.

**Board 260. Neighborhood Level Socioeconomic Status and *Campylobacter* Incidence: Connecticut, 1999–2009**

**K. Bemis**, R. Marcus, J. Hadler;
Yale Emerging Infections Program, New Haven, CT, USA.

**Background:** The role of socioeconomic factors, an important determinant in many chronic and infectious diseases, has not been explored for *Campylobacter* infection. One accepted indicator of socioeconomic status at the neighborhood level is the percent of the population living below the poverty line in a given census tract. Geographic information system (GIS) technology was used to examine variation in campylobacteriosis incidence rates among wealthy and economically deprived

census tracts in Connecticut. **Methods:** Positive laboratory culture results for *Campylobacter* have been reportable in Connecticut (CT) since the early 1990s. CT census tracts were divided into four categories: 0 - 4.9%, 5 - 9.9%, 10 - 19.9%, and ≥20% of the population living below the federal poverty line as of the 2000 census. A total of 5708 of 5950 (95.9%) cases of *Campylobacter* reported from 1999-2009 were geocoded and categorized. Age-adjusted incidence rates were calculated for each category. Age-adjusted rates were also determined by race/ethnicity. **Results:** We found a dose-response relationship between higher *Campylobacter* incidence and lower census tract level economic deprivation. When neighborhood poverty level 0 - 4.9% was set as the reference group, age-adjusted incidence rate ratios were 0.88, 0.71 and 0.60 for the 5 - 9.9% level, 10 - 19.9% level, and ≥20% level, respectively (p<0.001, chi square for trend). This trend is consistent across time periods, race/ethnic groups and age groups with two exceptions. Overall, there was no neighborhood poverty gradient among blacks, and there was a reverse gradient (higher rates with higher neighborhood poverty) among 0-9 year olds. **Conclusions:** *Campylobacter* incidence generally increases as neighborhood poverty decreases. Additional study is needed to determine whether these findings are consistent with the epidemiology of known *Campylobacter* risk factors (e.g., international travel, eating out at restaurants serving chicken) or in part reflect different likelihood of *Campylobacter* diagnosis. These findings can be used to more effectively target educational interventions, especially in wealthier neighborhoods. Area-based socioeconomic measures provide an additional perspective and should continue to be used to study this pathogen and other food-borne illnesses.

**Board 261. The Spatial Relationship between Epidemics of TB and HIV**

**O. Postnov**[1], L. Averbukh[1], O. Neduzhko[1], S. Pozdnyakov[1], N. Slavina[1], Y. Farion[2];
[1]Ukrainian Anti-plague Research Institute, Odessa, Ukraine, [2]Institute of Geography of the National Academy of Sciences of Ukraine, Kiev, Ukraine.

**Background:** HIV and TB epidemics are a heavy burden for the world healthcare system. Generalization of HIV epidemic occurs in many countries. Clear identification of high risk groups and areas become one of the main ways to improve the response to HIV epidemic. TB and HIV are interrelated on the personal level (TB as an opportunistic infection) and at the population level (common social risks of injecting drug use, poverty, etc.). Spatial links between HIV and TB epidemics considered as an important topic to study. **Methods:** TB incidence among people living with HIV (PLWH) and in total population of Odessa region was studied. Spatial structure of TB and HIV prevalence as of 01.01.2010 at the county level (Rhadas) of Odessa region was analyzed using Kulldorf's spatial clustering realized in SatScan. Visualization was carried out on QuantumGIS. **Results:** The registry data on 16 951 PLWH with total observational time of 85 899 person/years was studied. Among study group 653 TB cases were found. TB incidence among PLWH was 760.2 per 100,000 person/years. The annual TB incidence in the general population ranged from 77,4 to 95,3 per 100,000 population. 12 TB spatial clusters included 288 Rhadas (58.9%) were identified. Relative risk (RR) in identified clusters ranged from 1,34 to 7,17, log likelihood ratio (LLR) ranged from 8.46 to 25.49, p<0,05 was all cases. 15 HIV spatial clusters included 298 Rhadas (60.9%) were found. RR was 1.76 to 3,14, LLR 9.15 to 201.84, p<0.05 in all clusters. Total of 327 Rhadas were included in the spatial clusters, 212 (64.8%) of Rhadas were simultaneously included in TB and HIV spatial clusters. **Conclusions:** The risk of TB among PLWH is much higher compared with a general population. Epidemics of HIV and TB have similar characteristics of spatial distribution. It is possible to use TB prevalence rates for indirect estimation of HIV prevalence and for identification of areas with high risk of HIV in a countries with strong TB surveillance system. Such estimations may be used for planning and carrying out the HIV prevention activities and increase the effectiveness of control measures.

**Board 262. Evolution of GIS Applications in Haiti Cholera Response**

**N.C. Purcell**;
Centers for Disease Control and Prevention, Port-au-Prince, Haiti.

**Background:** Since the beginning of the cholera outbreak in Haiti, the Centers for Disease Control and Prevention has used geographic information systems (GIS) as a tool for visualizing, understanding, and responding to the epidemic. **Methods:** Responding to needs articulated by the Haitian government and by CDC offices involved in the cholera response, the types of geospatial visualizations and analyses used to respond to the cholera epidemic have changed over time. Initial mapping activities focused on locating suspected cases and monitoring the spread of laboratory-confirmed cases. Extensive analyses of gaps in the placement of cholera treatment facilities in relation to communes with confirmed cases were conducted. In response to early concerns, ballast discharge areas were geographically analyzed to examine the potential for cholera to spread outside Haiti. Once cholera had become established nationwide, a dynamic web-based map was developed to enable viewers to see an integrated suite of maps, graphs, and tables displaying surveillance data by department over time. Based on the first nine months of laboratory and surveillance data, retrospective mapping of total suspected cases against laboratory-confirmed *Vibrio cholerae* specimen positivity rates was employed to highlight potential misclassification of suspected cases. **Results:** Though there are lingering difficulties in balancing the timeliness and completeness of data reporting, cholera incidence and attack rates are currently mapped nationwide at the commune level on a weekly basis and these maps are incorporated into reports disseminated by the Haitian ministry of health, Ministère de la Santé Publique et de la Population (MSPP). Additionally, GIS continues to be used to visualize the distribution of cholera treatment facilities and areas with surges in the number of new cases in order to understand the shifting landscape of resources available to combat cholera. In the future, it is hoped that leadership for mapping surveillance data can be transferred to MSPP or to the departmental level, to facilitate a swift response to future changes in the course of the epidemic. **Conclusions:** GIS has been a critical tool to guide and visualize the public health response to Haiti's cholera epidemic by adapting to changing data needs.

**Board 263. Habitat Associations of Eastern Equine Encephalitis Horse Cases in Florida from 2005–2010**

**P. Vander Kelen**[1], J. Downs[1], L. Stark[1,2], T. Unnasch[1];
[1]University of South Florida, Tampa, FL, USA, [2]Florida Department of Health, Bureau of Laboratories, Tampa, FL, USA.

**Background:** Eastern Equine Encephalitis virus (EEEV) is an alphavirus with high pathogenicity in both humans and horses. Florida continues to have the highest occurrence of human cases in the USA, with four fatalities recorded in 2010. Unlike other states, Florida displays year-round EEEV transmission, possibly due to the presence of tropical and subtropical climates that permit overwintering of the virus. This research examined spatial patterns of documented horse cases during 2005-2010 in order to understand the relationships between habitat and the intensity of EEEV transmission in Florida. **Methods:** To determine which habitats play a role in EEEV transmission to horses in Florida horse cases from 2005-2010 were analyzed. First, a cluster analysis was performed using density-based spatial clustering of applications with noise (DBSCAN). Second, habitat associations of EEEV were examined using compositional analysis and ArcGIS 9.3. In this context, the amounts of habitat types contained within 3 km buffers of horse cases were compared to the amounts available in the surrounding landscapes. **Results:** The DBSCAN algorithm identified seven distinct spatial clusters that contained 373 of the 441 horse cases, with the remaining records identified as noise. The resulting ranking matrix indicated that the top five habitats associated with horse cases were rural residential areas, pastureland, upland hardwood forests, vegetated non-forested wetlands, and tree plantations. Wetland hardwood forests, typically the habitat most associated with EEEV transmission was ranked 11 out of the 14 analyzed habitats. **Conclusions:** The results suggest that although wetland hardwood forests are

important ecological areas for vectors of EEEV, they are relatively less important in regards to EEEV transmission to horses. Instead, focusing surveillance in areas with rural residential, pastureland, upland hardwood forests, vegetated non-forested wetlands and tree plantations may lead to improved surveillance against both human and horse cases of Eastern Equine Encephalitis.

## Improving Global Health Equity for Infectious Diseases

Tuesday, March 13
5:00 PM – 6:00 PM
Grand Hall

**Board 264. Another Difference between Boys and Girls: Sex-Based Differences in Lyme disease**

**L.A. Crowder**[1], A. Rebman[1], V. Yedlin[1], M. Soloski[2], J.N. Aucott[2];
[1]Lyme Disease Research Foundation of Maryland, Lutherville, MD, USA, [2]Johns Hopkins University, School of Medicine, Baltimore, MD, USA.

**Background:** Lyme disease is the most commonly reported vector-borne infectious disease in North America. Reported cases each year has been steadily increasing since 2006, with cases numbering over 38,000 in 2009. Sex based differences have been seen in other infectious diseases, but have not been explored thoroughly in early Lyme disease. **Methods:** Seventy-six patients with clinically diagnosed early untreated Lyme disease were enrolled in a prospective cohort study. Patients were treated with doxycycline and seen seven times over a two-year period. At each visit, a physical exam, interval history, clinical symptoms, and laboratory evaluation was performed. Blood was sent for ELISA and Western Blot to a commercial laboratory, PBLs from whole blood are used for flow cytometry, and serum and plasma are frozen for future analysis. **Results:** While no significant differences were seen in the demographic variables of age, education, race, or income of our population, we noticed significant sex based differences in clinical and immune presentation in cases. We observed clinical differences by sex in symptoms recorded pre-treatment (V1): greater percentage in women of reported heart palpitations (24.32% vs. 0.0%, p=<0.001) and gastrointestinal symptoms (51.35% vs. 20.51% p=0.0079). A similar percentage of female and male patients were seropositive by 2-tier ELISA and western blot testing at V1, however 44% of females did not seroconvert by 2-tier testing when tested again 3 weeks post-treatment (V2), compared to 19% of males (p=<0.001). Significant differences by sex were seen in percentage of CD4 + CCR5 + T cells (female median (m) = 9.82 vs. male m= 5.96, p=0.0268) and in the CD4 + CCR5/CRTH2 ratio (female m=4.17, male m=2.91, p=0.0499). Monocyte values were lower in females when compared with the values in men (0.57 vs. 0.71, p=0.0391). **Conclusions:** In a cohort of patients with a confirmed diagnosis of early Lyme disease, female patients display more clinical symptoms, have a greater chance of not seroconverting post-treatment and have evidence for altered CD4 effector responses. These findings suggest that there should be further research in the field of sex based differences in the effects of early Lyme disease in otherwise healthy populations.

**Board 265. Role of Influenza among Pediatric Hospitalizations for Respiratory Disease: A Review of the Literature**

**K. Lafond**[1], P. Glew[1], H.-Y. Hsu[2], P. Wardhan[1], H. Nair[3], J. Bresee[1], E. Azziz-Baumgartner[1], M.-A. Widdowson[1];
[1]Centers for Disease Control and Prevention, Atlanta, GA, USA, [2]Emory University, Atlanta, GA, USA, [3]University of Edinburgh, Edinburgh, UK.

**Introduction**: The burden of severe influenza disease is high among children, but to date estimates have been available mostly from populations in higher income settings. We reviewed the published literature

to summarize the overall proportion of severe respiratory illness due to influenza among children globally. **Methods**: We conducted a systematic search of three biomedical databases (Pubmed, Embase, Web of Science) for published articles in any language on humans in any setting with respiratory infection (including acute respiratory infection, influenza-like illness, and pneumonia) and testing for influenza published from 1995 through 2011. We supplemented this search by reviewing citation lists and contacting experts in the field to identify additional eligible articles. Articles were included in our review if they included original human research, presented ≥12 months of continuous influenza virus testing with ≥50 samples tested, and included both number positive and number tested for influenza. We further restricted our analysis to studies of hospitalized children (<18 years), and abstracted the percent of tested inpatients positive for influenza virus. **Results**: We identified 5,533 articles through our initial search, of which 107 articles met the inclusion criteria. Of these, 52 studies from 25 countries focused on pediatric age groups. The percent of individuals that tested positive for influenza virus from the eligible studies (influenza positivity) ranged from 0 - 36%, with a median of 5% and an interquartile range of 2 to 8%. Samples tested through direct and indirect immunofluorescence had lower influenza positivity (median 2% and 3% respectively) than PCR, viral culture, or a combination of these (median 7%, 6%. and 9% respectively [p<0.01]). We found no significant difference in the percent positivity between high- (n=22) versus middle- and low- (n=27) income countries or by clinical case definition for testing (ARI versus lower respiratory infection versus pneumonia). **Conclusions:** The median proportion of pediatric hospitalizations that tested positive for influenza was 5% without systematic variation among source countries or clinical criteria used for testing. Immunofluorescence may consistently underestimate the true role of influenza in severe respiratory disease.

**Board 266. A Bacteriologic Assessment of Imported Cheese From Mexico–El Paso, Texas, 2008**

**M. Sandoval**[1], A. Corona-Luevanos[2], F. Puentes[2], L. Escobedo[2], M. Escobedo[3];
[1]CDC, Los Angeles, CA, USA, [2]Texas Department of State Health Services, El Paso, TX, USA, [3]CDC, El Paso, TX, USA.

**Background:** The high volume of travelers from Mexico entering and residing in the United States is responsible for the popularity demand for unpasteurized Mexican products. Consumption of unpasteurized cheese products has been linked to serious infectious disease outbreaks, including listeriosis, brucellosis, salmonellosis, and *Mycobacterium bovis* . To assess bacterial contamination levels of cheese imports entering through the El Paso Port of Entry, the Texas Department of State Health Services (DSHS), in collaboration with the El Paso CDC Quarantine Station, conducted an assessment of imported cheese from Mexico carried by arriving international bus passengers in El Paso, Texas. **Methods:** Cheese samples were collected from passengers arriving to El Paso from the interior of Mexico. The study population consisted of passengers 18 years of age and older traveling on commercial bus routes originating in Mexico and terminating in El Paso. Passengers who agreed to participate were asked to answering a questionnaire, which contained information on demographics, travel patterns, and symptoms. Cheese samples were collected and transported weekly to the City of El Paso Department of Public Health laboratory and were tested for *Salmonella* spp., *Listeria* spp*.,* and *Brucella spp.* Additional tests for *Mycobacterium tuberculosis* complex were conducted at the US Department of Agriculture (USDA) laboratory in Ames, Iowa. **Results:** Of 3,703 passengers who agreed to answer the questionnaire, 642 reported carrying cheese from Mexico. Of those, 247 (38.5%) provided a sample. Two tested positive for *Brucella* and one for *Salmonella*. The overall prevalence of cheese contaminated with any of the pathogens being tested (*Salmonella*, *Brucella,* and/or *Listeria)* was 1.2%. Finally, while no *M. tb* complex organisms were recovered from the cheese samples tested, *M. fortuitum* was isolated from one sample. **Conclusions:** Despite the low pathogenic contamination seen in our sample, there is still a need for additional culturally targeted interventions and surveillance of cheese imported from Mexico. Collaboration with Customs and Border Protection (CBP) to enhance surveillance, as well as with USDA

on prevention activities is necessary to reduce the risk of infectious diseases associated with imported cheese.

**Board 267. Infectious Disease Morbidity in the US Region Bordering Mexico, 1999–2009**

**C. Pezzi**[1], M. Fonseca-Ford[1], S. Waterman[1], T. Doyle[2];
[1]CDC, San Diego, CA, USA, [2]CDC, Atlanta, GA, USA.

**Background:** The United States and Mexico border extends for 3000 km and separates two countries with significant differences in health profiles and systems. The communities and living conditions of the US border region adjacent to Mexico are heavily influenced by the high volume of transborder population movement between the two countries, resulting in regional public health challenges distinct from the rest of the US. **Methods:** We reviewed infectious disease morbidity case reports from the Nationally Notifiable Disease Surveillance System to assess differences between the US-Mexico border region and the rest of the US. Incidence rates were compared by calculating rate ratios between border and non-border regions for 27 nationally notifiable infectious diseases reported between 1999 and 2009. Temporal trends in regional incidence rates were also examined over the 11-year time period. **Results:** This analysis identified excess incidence (a statistically significant rate ratio at least 1.5) of both foodborne and wound botulism, brucellosis, Hansen's disease, hepatitis A, hepatitis B, measles, shigellosis, tetanus, and varicella in the border region compared to the non-border region of the US. Though incidence of hepatitis A and B disease decreased in both border and non-border regions between 1999 and 2009, incidence remained higher in the border region relative to the rest of the US. The rate ratio for Hepatitis A incidence comparing border counties to the non border US states was 2.28 (95% CI: 2.22,2.33); the same regional comparison for Hepatitis B yielded a rate ratio of 1.54 (95% CI: 1.49,1.59). **Conclusions:** These data indicate that the incidence of several infectious diseases, a number of which are food and waterborne or vaccine preventable, remains significantly higher in the United State border region than in non-border regions despite overall decreases in incidence. These data suggest that continued efforts are needed to address infectious disease prevention and infrastructure in this complicated and dynamic border environment.

## Vaccine Preventable Diseases

Tuesday, March 13
5:00 PM – 6:00 PM
Grand Hall

**Board 268. Group A Antibody Persistence Five Years after Meningococcal Polysaccharide Vaccination in the Sudan**

**A.A. Ismail**[1], D. Granoff[2];
[1]National Public Health Laboratory, Khartoum, Sudan, [2]Center for Immunobiology and Vaccine Development, Children's Hospital Oakland Research Institute, Oakland, CA, USA.

**Background:** Large meningococcal group A epidemics occur periodically in the Sudan, a country within the "meningitis belt" of Sub-Saharan Africa. Immunization with meningococcal polysaccharide vaccine induces protective serum bactericidal titers but little information is available on the duration of protection **Methods:** Serum samples were obtained from 20 subjects, aged 11-47 years, who resided in the Sudan, and who had participated in a meningococcal polysaccharide immunogenicity study five years earlier. Persistence of serum group A bactericidal titers (measured with human complement) was compared to that of 12 immunized adults in North America with no known exposure to group A organisms **Results:** By five years the respective reciprocal geometric mean bactericidal titers had

declined in both groups (82 to 34 in Sudanese, and 69-11 in North Americans, p < or = 0.03). However, the proportion of sera with protective bactericidal titers (> or =1:4) at five years was higher in the Sudanese than North Americans (80% vs. 42%, p < or = 0.05). **Conclusions:** Recommendations for periodic meningococcal polysaccharide vaccination every 3-5 years to maintain group A immunity may be more appropriate for persons living outside of endemic areas than for persons residing in endemic regions since immunity in endemic areas can be maintained by periodic exposure to group A organisms, even during periods between epidemics.

**Board 269. Losing and Re-establishing Adenovirus Vaccine for the US Military: A 17 Year Time Line**

**J.C. Gaydos**[1], C.H. Hoke, Jr.[2], K.L. Russell[1], P.K. Russell[3];
[1]Armed Forces Health Surveillance Center, Silver Spring, MD, USA, [2]US Army Medical Materiel Development Activity, Fort Detrick, MD, USA, [3]Albert B. Sabin Vaccine Institute, Washington, DC, USA.

**Background:** Adenoviruses (ADVs) were isolated from soldiers with acute respiratory illness (ARI) in the 1950s. Up to 80% of recruits developed ADV infections and ADV Types 4 and 7 accounted for 60% of all ARI in hospitalized recruits. Researchers from the Walter Reed Army Institute of Research, the National Institutes of Health and Wyeth Laboratories, Inc., developed live, enteric-coated, oral vaccines for Types 4 and 7. Routine administration of the vaccines to recruits began in 1971. The vaccines were extremely safe. Outbreaks due to ADV 4 and 7 ceased in immunized populations. **Loss of the Vaccines:** Citing antiquated facilities, Wyeth, the sole producer, notified the Department of Defense (DoD) in 1994 that it was halting ADV vaccine production. Bulk production ceased in 1995. Tableting ended in 1996. The remaining vaccine was rationed for use only during periods of high risk for ARI. All stocks were depleted by 1999. ADV-associated ARI reappeared, generally due to Type 4 but sometimes due to Type 7 and other types. Spread of ADV outbreaks outside recruit centers was documented. ADV-associated deaths occurred. A serological survey of new, unimmunized soldiers confirmed the lack of protective antibodies to Types 4 and 7; 90% were susceptible to at least one type. The Institute of Medicine strongly recommended rapidly restoring the vaccines. **Re-Establishing the Vaccines:** The Army provided initial funding. The DoD established a requirement for the vaccines and provided long term funding. In 2001, the US Army Medical Research Acquisition Activity awarded a contract to re-establish manufacturing of Types 4 and 7 vaccine and to sponsor application to the US Food and Drug Administration (FDA) for approval to market the vaccine. Clinical trials, including a trial in about 4,000 Army and Navy volunteers, documented immunogenicity (94.5% - type 4; 93.8% - type 7), safety and efficacy (99.3% for Type 4). On March 16, 2011, the FDA approved a Biologics License Application to market Adenovirus Type 4 and Type 7 Vaccine, Live, Oral in military populations 17 through 50 years of age. **Summary:** Following notification in 1994 that ADV vaccine production would cease, awarding a contract to re-establish manufacturing and FDA approval required seven years. Licensing the new vaccine required 10 additional years.

**Board 270. Number Needed to Vaccinate to Prevent Pertussis in Infants**

**G.H. Lim**[1], S.L. Deeks[1,2], N.S. Crowcroft[1,2];
[1]Public Health Ontario, Toronto, ON, Canada, [2]University of Toronto, Toronto, ON, Canada.

**Background:** Pertussis deaths occur primarily among infants too young to have been vaccinated, however disease occurs in all ages. Canadians benefit from 6 doses of acellular pertussis vaccine in childhood/adolescence. In Ontario, Canada (13.1 million), a universal adult pertussis program was recently implemented. Others have implemented targeted vaccination of close contacts of infants (cocooning). We applied the concept of ‘number needed to treat’ to estimate the number of adults needed to be vaccinated (NNV) to prevent disease, hospitalization and death among infants had a cocoon strategy been implemented in Ontario. **Methods:** $NNV=1/(P_M \times R) + 1/(P_F \times R)$, where $P_M$, $P_F$ = proportions of infants infected by mothers, fathers and R = rates of disease, hospitalization or death

among infants. $P_M$ and $P_F$ estimates were based on several studies (14%-21% and 6%-11%, respectively). 2005-2009 incidence and hospitalization rates were derived from Ontario's reportable disease data and Discharge Abstract Database, respectively, and demographic data from Statistics Canada. Mortality rates were estimated as case fatality ratio (0.2%) X incidence rates. Sensitivity analyses examined the impact of excluding unknown sources of infection when deriving $P_M$ and $P_F$, adjusting for underreporting (with inflation factors of 9.4 for incidence and 4.7 for hospitalization), and varied estimates of $P_M$, $P_F$ and R. **Results:** Using the range of estimates for $P_M$ and $P_F$, the NNV to prevent one case, hospitalization or death from pertussis was between 1,411-2,425, 12,254-21,061 and 708,584-1,217,878, respectively. When not adjusted for underreporting, NNV increased to 13,260-22,791, 57,592-98,987 and 6,660,688-11,448,057, respectively. Rarer outcomes were associated with higher NNV. Higher estimates of R, $P_M$ and $P_F$ and adjusting for underreporting decreased NNV, as did excluding unknown sources of infection from $P_M$ and $P_F$. **Conclusions:** NNV analyses can incorporate many assumptions and assist when considering implementation of a targeted or universal program. They can also identify critical parameters that determine whether a strategy is worthwhile. For cocooning, the lower end of the range of NNV estimates is relatively low but the upper extends into unacceptably high numbers. A critical determinant is the level of pertussis underreporting.

**Board 271. Varicella Outbreak in a Highly Vaccinated Elementary School in Beijing, China**

**C. Wang**;
Centers for Disease Control and Prevention, Atlanta, GA, USA.

**Background:** Varicella vaccine is available in China for purchase on a private basis with a single dose recommended for children ≥12 months of age. Since 2006, varicella vaccine has been provided free of charge to susceptible students in school settings in Beijing for outbreak control. We investigated a varicella outbreak in an elementary school in Beijing to examine transmission patterns and risk factors for vaccine failure. **Methods:** A varicella case was defined as an acute generalized maculopapulovesicular rash without other apparent cause in a student. Breakthrough varicella was defined as varicella > 42 days after vaccination. Vaccination information was collected from immunization records. Information on prior disease history and clinical presentation came from a survey of students' parents. **Results:** Of the 951 students, 934 (98%) reported never having had varicella, of whom 916 had received 1 dose varicella vaccine and 2 had received 2 doses, for an overall 98.3% school-wide vaccination coverage prior to the outbreak. A total of 87 cases were identified from August 30 to December 28, 2010 for an overall attack rate of 9.4%. The index case was a vaccinated student exposed to a neighbor with varicella. Only one other case had any recognized varicella exposure outside of the school. Most cases were breakthrough varicella (86/87, 99%) and had <50 lesions (83/87, 95%). Among students who had received a single dose of varicella vaccine, age, sex, time since vaccination before outbreak (<5 vs. ≥5 years) and age at vaccination (<15 vs. ≥15 months) had no effect on development of breakthrough varicella, but students in grade 1 were more likely to have varicella than those in grade 2 (12.8% vs. 7.7%, P=0.01). **Conclusions:** Single dose varicella vaccine may not be sufficient to prevent varicella outbreaks. Two-dose varicella vaccination may be an option for preventing and improving control of varicella outbreaks in Beijing.

## Vulnerable Populations

Tuesday, March 13
5:00 PM – 6:00 PM
Grand Hall

**Board 272. Genetic Heterogeneity of HCV in Intrafamilial Transmission among Female Patients**

**S. Kanwal**, T. Mahmood;
Quaid e Azam University Islamabad, Islamabad, Pakistan.

**Background:** Hepatitis C is an infectious disease, caused by blood borne pathogen; the Hepatitis C Virus. Viral prevalence and incidence varies according to different geographical regions. The prevalence of HCV has increased worldwide but dramatically in developing countries including Pakistan. It is responsible for serious consequences owing to its high risk of chronicity potentially leading to liver cirrhosis and hepatocellular carcinoma. Protective methods, such as the use of vaccination to reduce the risk of transmission have not yet been established. A better knowledge of the risk factors of HCV transmission is needed for the adoption of preventive measures by those at risk. A number of epidemiological studies have focused on the risk factors for transmission of the infection and the role of sexual and intrafamilial contact. Household contacts of HCV positive patients are considered at increased risk of HCV infection. The aim of the present study was to resolve the core region based genotypes of seven females who got the infection through intrafamilial transmission. **Methods:** In order to complete the task nested RT- PCR performed after reverse transcribing the viral RNA. Sequencing of the purified PCR samples was carried out to assign the genotypes; core region was the target site for sequencing. Neighbor Joining (NJ) method was used to study the evolutionary relationship among the studied samples and HCV strains reported from different geographical regions of the world. **Results:** Genotyping revealed four different genotypes out of which two samples could not be typed by sequencing. In addition to HCV genotype 3 (3a and 3b), two new genotypes have been reported for the first time: 4a from sub urban region of Rawalpindi and 6e from all over the Pakistan. It was observed that high genetic diversity of HCV is present in all different geographical regions of the world. Migration patterns were identified among strains of Europe, South America, Asia and Africa that have shown high diversity in their respective geographical regions. **Conclusions:** Hence the observed viral heterogeneity in intrafamilial transmission is changing very fast and there are chances that those viral strains will be more dangerous in coming years so future policies should be designed keeping in view the heterogeneity of virus.

**Board 273. Diabetes as an Underlying Health Condition among New York State Emerging Infection Program ABCs Pathogens: 2000–2009**

**K. Burzlaff**[1], G. Smith[1], K. Malloy[2], D. Abraham[3], J. Karr[1], N. Spina[2], S. Solghan[2], S. Zansky[2];
[1]NYSDOH-EIP, Rochester, NY, USA, [2]NYSDOH-EIP, Albany, NY, USA, [3]CSTE Fellow, Albany, NY, USA.

**Background:** Diabetes is a chronic disease affecting 25.8 million Americans, with a prevalence that has increased in the adult population from 6.0% in 2000 to 8.3% in 2009. As an underlying health condition, diabetes has been associated with invasive disease, including those under surveillance by the CDC Active Bacterial Core Surveillance Program (ABCs). These causative agents include: *Haemophilus influenzae*, *Neisseria meningitidis, Streptococcus pyogenes* (GAS), *Streptococcus agalactiae (GBS)*, and *Streptococcus pneumoniae.* We compared the prevalence of diabetes among adult invasive ABCs cases reported to the NYS Emerging Infections Program (EIP) to the prevalence among the NYS adult population from 2000-2009. We also describe ABCs cases reporting diabetes. **Methods:** NYS EIP conducts surveillance in 15 counties of NYS for cases of invasive illness caused by the ABCs pathogens. Underlying medical conditions (e.g., diabetes) are captured via medical record reviews and patient and provider interviews. NYS diabetes prevalence rates were obtained from the CDC's Division of Diabetes Translation. **Results:**

From 2000-2009, 5,840 invasive ABCs cases were reported to the EIP with 1,965 cases excluded from analysis due to missing underlying illness data. Although there was variability by year, overall crude diabetes prevalence was consistently higher among ABCs cases compared to NYS residents, 35.6% and 5.6%. In NYS, diabetes prevalence was highest among persons aged 65-74 years (range 13.9-18.9%) however, prevalence was highest in the 45-64 year age group for ABCs cases (range 31.0-45.5%). Among ABCs cases, no significant differences were noted for hospitalization and length of stay, but death was reported more often among cases ≥ 65 years old who reported diabetes compared to those not reporting diabetes (P < .04). **Conclusions:** Diabetes is commonly reported among ABCs cases. As diabetes rates continue to rise nationally, we are consistently observing a greater burden of disease in ABCs cases and increased mortality in older persons. Differences in baseline health status could account for variations in diabetes prevalence among age groups when comparing ABCs cases to the NYS population. Diabetes management should continue to focus on older adults, but education and prevention is also critical for younger diabetics.

**Board 274. Post-Epidemic Seroprevalence of Rift Valley Fever Virus among Somali Villages in North Eastern Kenya**

**A. LaBeaud**[1], L.J. Sutherland[2], S. Muiruri[3], S. Dahir[3], Z. Traylor[2], E. Muchiri[3], A. Hise[2], J. Kazura[2], C.H. King[2];
[1]Children's Hospital Oakland Research Institute, Oakland, CA, USA, [2]Case Western Reserve University, Cleveland, OH, USA, [3]Ministry of Health, Nairobi, Kenya.

**Background:** In endemic areas, Rift Valley fever virus (RVFV) is a significant threat to both human and animal health. Goals of this study were to measure human anti-RVFV seroprevalence in a high-risk area following the 2006-2007 Kenyan Rift Valley Fever (RVF) epidemic, to identify risk factors for RVFV exposure, and to monitor for sequelae of RVFV disease. **Methods:** We conducted a large cross-sectional village cluster survey among residents aged 1-85 years in 6 villages in Ijara District, Northeastern Province, Kenya: Tumtish (N=190, 47 households), Matarba (N=242, 70 households), Korahindi (N=289, 86 households), Gedilun (N=237, 63 households), Golalbele (N=85, 27 households), and Sabenale (N=64, 20 households). Participants underwent questionnaire administration, physical exam, vision testing, and blood collection for RVFV testing. **Results:** One thousand one hundred seven individuals were tested for RVFV exposure via RVFV IgG ELISA; 667 or 60% were women and 631 or 57% were children aged 1- 15 years. Overall, 172/1107 or 15.5% (CI95% 14.72-19.48) of local residents were RVFV seropositive. Seroprevalence varied by village: Tumtish (27/190, 14%, CI95% 9.1-19.05), Matarba (37/242, 15%, CI95% 10.65-19.83), Korahindi (53/289, 18%, CI95% 12.64-21.39), Gedilun (32/237, 13.5%, CI95% 8.93-17.60), Golalbele (13/85, 15%, CI95% 8.4-24.73), and Sabenale (10/64, 15.6%, CI95% 7.76-26.86). Visual impairment (defined as ≤20/20) was much more likely in the RVFV-seropositive group (P= 0.0001). **Conclusions:** Our results highlight significant variability in RVFV exposure in six neighboring villages having very similar climate, terrain, and Somali populations. In concordance with previous studies, RVFV seropositivity was associated with poor visual acuity. Further analysis of questionnaire data will elucidate primary risk factors for RVFV exposure.

**Board 275. Assessment of Risk Factors for Coronary Artery Abnormalities among Children with Kawasaki Syndrome in California**

**L.L. Sinden**[1], F. Tabnak[2], R.C. Holman[1], R.A. Maddox[1], L.B. Schonberger[1], J. Kim[3], D. Vugia[3], E.D. Belay[1];
[1]Centers for Disease Control and Prevention, Atlanta, GA, USA, [2]California Department of Public Health, Sacramento, CA, USA, [3]California Department of Public Health, Richmond, CA, USA.

**Background:** Kawasaki syndrome (KS) typically occurs in children less than 18 years of age and is the leading cause of acquired heart disease among children in the United States. Understanding the epidemiology of KS and risk factors for coronary artery abnormalities (CAA) can lead to timely diagnosis

and treatment of KS and can limit occurrence of CAA. **Methods:** Epidemiologic characteristics, including risk factors for the development of CAA, among KS cases less than 18 years of age during 2000-2009 reported by California to the Centers for Disease Control and Prevention's KS national surveillance system were analyzed. **Results:** California reported 2056 KS and atypical KS cases less than 18 years of age for 2000-2009. The median age of cases was 2 years; 60% of cases were male. Fifty-six percent of cases were reported as white race and 28% were reported as Asian/Pacific Islander race. Ninety-eight percent of cases received IVIG. Ninety percent of cases had testing for cardiac complication; 19% had CAA, most of which were coronary artery dilatations (82%). Eighty-seven percent of cases with CAA were less than 5 years of age. Characteristics associated with the occurrence of CAA in KS cases were male gender, Asian/Pacific Islander race, age <1 year or ≥8 years, and not receiving IVIG treatment before the fifth day of illness. **Conclusions:** This study suggests that IVIG treatment before the fifth day of illness may reduce CAA; this finding highlights that timely diagnosis and treatment of KS is important in reducing CAA among children with KS.

## Laboratory Support

Tuesday, March 13
5:00 PM – 6:00 PM
Grand Hall

**Board 276. National Survey of U.S. Laboratories Performing Testing for the Diagnosis of Tuberculosis**

**K. Wroblewski**[1], A. Starks[2], T. Dalton[2], National TB Laboratory Services Survey Workgroup;
[1]APHL, Silver Spring, MD, USA, [2]CDC, Atlanta, GA, USA.

**Background:** In response to a 2002 Federal Tuberculosis (TB) Task Force recommendation, the Association of Public Health Laboratories (APHL) and the Centers for Disease Control and Prevention (CDC) conducted the National TB Laboratory Services Survey. The survey's major objective was to assess the capability and capacity of commercial, clinical, and public health laboratories in the United States to provide TB diagnostic services. Results of the survey will be used to identify and address gaps in US TB laboratory diagnostic services. **Methods:** A 118 question survey was launched to 1444 commercial, clinical and public health laboratories on September 7, 2010 and closed in February 2011. The survey was distributed electronically via MRInterview. The questions were divided into 11 different categories including: demographics, testing methodologies; referral strategies; specimen collection, handling and transport; reporting practices; laboratory staff and training; biosafety practices; proficiency testing; public health and epidemiology; and future plans. Results were analyzed using SAS 9.2. **Results:** Forty five percent (656/ 1,444) of recipient laboratories responded to the survey. Preliminary analysis of the data shows that 18% (106/580) perform only AFB-smear microscopy while 82% (474/580) perform both AFB-smear microscopy and culture. Most laboratories use reference laboratories for MTBC identification (ID), first and second-line drug susceptibility testing (DST), and direct detection. 39% of laboratories perform fewer than 15 AFB smears per week. Of those performing culture, 40% inoculated fewer than 20 AFB cultures each week. Meeting recommended turnaround times appears to be difficult for many laboratories with only 71 of 149 (48%) reporting ≥80% of ID within 21 days and 57 of 145 (39%) reporting ≥80% of DST results within 28 days. **Conclusions:** Survey results revealed the complexity of referral networks required for TB testing services, identified training topics, and issues requiring further operational research important for strengthening U.S. laboratory systems. APHL and CDC continue to work together to identify ways to develop training tools and implement operational research topics.

**Board 277. Microbiological Characteristics of *Salmonella* Meningitis in a South African Population, 2003–2010**

**K.H. Keddy**[1,2], A. Sooka[1], P. Crowther-Gibson[1];
[1]National Institute for Communicable Diseases A Division of the National Health Laboratory Service, Johannesburg, South Africa, [2]Faculty of Health Sciences, University of the Witwatersrand, Johannesburg, South Africa.

**Background:** Laboratory-based surveillance for *Salmonella* provides invaluable information on disease trends, prevalent serotypes and antimicrobial resistance patterns, which may be used to improve disease management. *Salmonella* meningitis is an unusual, but well-documented presentation of invasive salmonellosis that may be problematic to treat: current guidelines for therapy of meningitis focus on more common pathogens. **Methods:** Between 2003 and 2010, diagnostic laboratories around South Africa referred *Salmonella* isolates to Enteric Diseases Reference Unit. Data were supplemented by systematic audits of laboratories in eight of nine provinces (2007 to 2010), through a common Laboratory Information System. Retrospective audits were conducted (2003 to 2006). Viable isolates were serotyped, according to standardised laboratory procedures and antimicrobial resistance profiles recorded, using E-tests®. **Results:** We identified 250 cases of *Salmonella* meningitis from diagnostic laboratories around South Africa. Age was recorded in 231/250 (92.4%) cases: the majority of patients, 104/231 (45.0%), were aged <1 year and were 2.7 times more likely to have meningitis than patients aged ≥1 year (P<0.001). There was no significant difference in the percentage of males and females with meningitis. Of cases, 220/250 (88.0%) isolates were available for further characterisation. *Salmonella* Typhimurium was the predominant pathogen (104/220; 47.3%), followed by *Salmonella* Enteritidis (45/220; 20.5%) and *Salmonella* Isangi (15/220; 6.8%). Three of 220 cases (1.4%) were due to *Salmonella* Typhi. Pentavalent resistance to ampicillin, chloramphenicol, streptomycin, sulfamethoxazole and tetracycline was documented in 34/220 (15.5%) isolates. Nalidixic acid resistance was observed in 52/220 (23.6%) isolates, none were resistant to ciprofloxacin. Extended-spectrum beta-lactamases were produced by 36/220 (16.4%) isolates. No isolates were resistant to imipenem. **Conclusions:** *Salmonella* meningitis is a rare, but important cause of meningitis in South Africa: children less than one year appear most susceptible to disease. Serotypes reflect predominant serotypes associated with invasive disease in this country. Management needs to include guidelines for potentially multidrug resistant strains.

**Board 278. Inhalation Anthrax, MN 2011, Laboratory Investigation**

**M.M. Sullivan**[1], L. Delaney[2], D. Henkes[3], B.A. Juni[1], D. Lonsway[2], R. Mattila[4], S. Saravia[1], R. Tiller[2], S. Tostenson[1], J. Bartkus[1];
[1]Minnesota Department of Health, St. Paul, MN, USA, [2]Centers for Disease Control and Prevention, Atlanta, GA, USA, [3]Lake Region Health Care Corporation, Fergus Falls, MN, USA, [4]National Guard, 55th Civil Support Team, St. Paul, MN, USA.

**Introduction:** State Public Health Laboratories (PHLs) play a key role in training clinical microbiology laboratories on identification, notification and referral of potential agents of bioterrorism, such as *Bacillus* sp., isolated from clinical cultures. The PHLs use CDC Laboratory Response Network (LRN) methods to confirm identification of *B. anthracis* (BA). PHLs also perform environmental cultures and molecular testing on items suspected of containing BA. On Friday August 5, the MN-PHL was notified by a sentinel lab in greater Minnesota (MN) of a suspicious *Bacillus* sp. isolate. **Methods:** The sentinel laboratory followed the ASM Sentinel Level Guidelines to attempt to rule out and refer a potential isolate of BA. Following protocol, staff immediately notified MN-PHL of this isolate and rapid transport was arranged. LRN methods were used to identify BA. MN-PHL sent the isolate to CDC for further characterization. In consultation and collaboration with CDC National Institute for Occupational Safety and Health (NIOSH) and the National Guard 55$^{th}$ Civil Support Team (CST), an environmental sampling

plan was developed. CST and MN-PHL collected samples, from the case-patient's car and items within the car. MN-PHL conducted testing. **Results:** Referred isolates from the case patient's blood cultures were identified as BA on August 6. Pleural fluid samples sent on August 8 were negative for BA. CDC determined the isolate to be pan-sensitive. The strain was identified as GT-59 by MLVA. All 54 environmental samples, which included sponge wipes and HEPA sock samples taken from inside and outside the car, air filter, cabin filter, boots, rocks, fishing lures, and antlers were negative for BA by PCR and culture. **Conclusions:** Previous investment in LRN activities enabled a rapid public health response. Training provided by MN-PHL to the sentinel lab allowed them to appropriately conduct preliminary assays, recognize a potential BA, and quickly notify and send the case-isolate to MN-PHL. Staff at MN-PHL rapidly identified BA and followed LRN notification policies. Assistance from NIOSH and CST enabled environmental sampling and testing, which were important components of the investigation. Established partnerships with the sentinel labs, CDC, CST, and others fostered an effective, thorough, and rapid response.

**Board 279. Development of RT-PCR Assays for Detection of Bacterial Virulence Factors**
**J.A. Olivas**;
Lawrence Livermore National Laboratory, Livermore, CA, USA.
**Background:** *Bacillus*, *Listeria*, *Pseudomonas*, *Escherichia,* and *Streptococcus* produce a wide array of virulence factors leading to a very large number of diseases. Knowledge of virulence factors will allow us to understand how to interrupt the pathogenetic events leading up to disease. Current culturing methods for pathogen detection are time-consuming, laborious, and fail to characterize virulence factors. In contrast, our virulence gene assays involve an inexpensive, facile, 40-minute method via TaqMan PCR, and utilize specific and sensitive virulence gene signatures that have the ability to characterize virulence factors. **Methods:** Lawrence Livermore National Laboratory (LLNL) has developed signatures for one-step TaqMan PCR detection of various virulence factors for *Bacillus, Listeria, Psuedomonas, Escherichia,* and *Streptococcus* utilizing both bioinformatics for signature identification and generation and Taqman screening for specificity and sensitivity of the generated signatures. **Results:** Through bioinformatics and Taqman PCR screening, we have developed multiple signatures to detect various virulence factors for *Bacillus*, *Listeria*, *Pseudomonas*, *Escherichia,* and *Streptococcus.* The Limit of Detection (LoD) ranges from 1-5000 copies of a given bacterial agent. There was zero cross-reactivity with near neighbor bacterial DNA, eliminating false positives. **Conclusions:** Characterization of virulence factors is an on-going process. Bacterial DNA is constantly in flux, thus a need to develop new assays is imperative LLNL has designed and developed a rapid and robust system for detection, identification and characterization of virulence factors of biothreat agents for the safety and security of the nation.

## Prevention Challenges for Respiratory Diseases

Tuesday, March 13
5:00 PM – 6:00 PM
Grand Hall

**Board 280. Trends in Legionellosis Surveillance, Connecticut, 2001–2010**
**K. Soto**, T. Rabatsky-Ehr, J. Krasnitski, J. Brockmeyer, M. Cartter;
Connecticut Department of Public Health, Hartford, CT, USA.
**Background:** Legionnaire's Disease (LD) is an important cause of community and healthcare-associated pneumonia. In Connecticut, LD has been monitored through passive physician and laboratory surveillance since 1997. The goal of this project is to examine ten years of LD trend data and to use

administrative discharge data to assess potential case under-reporting. **Methods:** A case of legionellosis was defined as a clinical diagnosis of LD with laboratory confirmation of infection through culture, urine antigen, direct fluorescent antibody, or four-fold serological titer change. Case data were obtained through case-patient and physician interviews. Discharge data were obtained from the Connecticut Health Information Management and Exchange (CHIME) for 2005-2009. Hospitalizations with a primary or secondary discharge specific for LD (ICD-9 code 482.84) had medical record reviews to assess case status and were not included in trend analysis. **Results:** In 2001-2010, 383 cases of LD were identified through passive laboratory surveillance. The majority of cases were diagnosed by urine antigen (93%). LD incidence over the past decade increased from 0.41 to 1.5 per 100,000 (p<0.001) and the case fatality rate decreased from 25% to 6% (p=0.01). Seventy-seven percent of cases occurred June-November; 14% of cases reported travel, 5% long term care facility residence, and 8% inpatient or outpatient hospital visits during the 10 days prior to symptom onset. Variation was seen in average annual incidence of LD by county (range 0.38-1.46/100,000). The highest rates of LD were consistently observed in central and southwestern Connecticut. Of the183 hospitalizations identified from CHIME data, 77 (42%) were not previously reported. Of these 77, 18 (24%) were laboratory-confirmed cases of LD; 21(27%) of hospitalizations had clinical suspicion or a single titer for LD; and 38 (49%) had either a history of LD, illness inconsistent with LD, or out of state residence. No trend was seen by year or hospital. **Conclusions:** LD is an increasingly reported cause of pneumonia in Connecticut. CHIME data indicated a 10% under-reporting of LD cases by passive surveillance. Continued surveillance validated with discharge data is recommended to better understand the changing epidemiology of LD, including its geographic variation.

**Board 281. Incidence of Hospitalization Due to Acute Respiratory Illness Associated with Respiratory Viral Infection in Children Aged <5 Years, Bangladesh, March 2010 – February 2011**

**N. Homaira**[1], S.P. Luby[1,2], K. Islam[1], M. Rahman[1], B.M. Sohel[1], A. Brooks[1], T. Azim[1], A.M. Fry[2], M.-A. Widdowson[2], M. Willby[2], G. Armstrong[2], J. Bresee[2], M. Rahman[3], E. Azziz-Baumgartner[2];
[1]ICDDR,B, Dhaka, Bangladesh, [2]CDC, Atlanta, GA, USA, [3]IEDCR, Dhaka, Bangladesh.

**Background:** Although respiratory disease is the leading cause of death in children aged < 5 years in Bangladesh, the contribution of respiratory viruses to severe disease is unknown. We combined surveillance and health utilization survey data to estimate the incidence of hospitalization associated with a respiratory viral infection in children aged < 5 years. **Methods:** During March 2010-February 2011, we conducted surveillance in four hospitals. On two random days per month, surveillance physicians collected nasal and throat swabs from hospitalized children aged < 5 years presenting with any two symptoms: fever, cough or difficulty breathing within seven days of symptom onset. We tested respiratory specimens for respiratory syncytial virus (RSV), rhinovirus, human parainfluenza viruses (HPIV 1−3), human metapneumovirus (HMPV), adenovirus, and influenza viruses using real-time RT-PCR. Investigators used hospital records to identify the primary catchment areas of the four hospitals and conducted a health utilization survey to determine the proportion of children aged <5 years in the catchment areas who sought care at the hospitals. We applied this proportion to the surveillance data to estimate the incidence of hospitalization. **Results:** Surveillance physicians enrolled 167 children. The median age of the hospitalized children was 5 months (range 2−10 months). Children were admitted a median of 2 days after symptoms onset and were hospitalized for a median of 4 days. Of the 167 respiratory specimens, 133 (80%) were laboratory-confirmed for a respiratory viral pathogen; 34% for rhinovirus, 23% for RSV, 16% for adenoviruses, 11% for HPIV3, 8% for HMPV, 2% for influenza viruses and 15% for more than one viral pathogens . The incidence of hospitalization associated with a respiratory virus was 79/1000 child-year. Specific virus-associated incidences/1000 child-years were 30 for RSV, 15 for rhinovirus, 11 for HPIV3, 9 for HMPV and adenoviruses and 2 for influenza viruses. **Conclusions:** In a setting where respiratory infections are the leading cause of child deaths,

hospitalizations for respiratory disease were common and most hospitalized children with a respiratory illness had detection of a respiratory virus. Interventions to reduce respiratory viral transmission may reduce the disease burden in Bangladesh.

**Board 282. Actual Use Analysis Results from a Randomized Controlled Trial of Non-pharmaceutical Interventions to Reduce Household Influenza Transmission in Bangkok, Thailand, 2008 to 2011**

**J. Levy**[1], P. Suntarattiwong[2], J.M. Simmerman[1], R.G. Jarman[3], S. Kaewchana[1], L. Kamimoto[4], S.J. Olsen[1], T. Chotpitayasunondh[2];
[1]Thailand MOPH-U.S. CDC Collaboration, Nonthaburi, Thailand, [2]Queen Sirikit National Institute of Child Health, Bangkok, Thailand, [3]U.S. Armed Forces Research Institute of Medical Sciences, Bangkok, Thailand, [4]Centers for Disease Control and Prevention, Atlanta, GA, USA.

**Background**: The effectiveness of non-pharmaceutical interventions in the prevention of influenza transmission is not well established. We report findings from a household (HH) randomized controlled trial in Thailand that studied interventions to reduce influenza transmission among HH members.
**Methods**: Index cases were patients <16 years-old seeking care for influenza-like illness <48 hours duration at the largest pediatric hospital in Bangkok. After a rapid antigen test (confirmed by RT-PCR), the HH of the index patient was randomized to 1) control, 2) hand washing (HW) or 3) hand washing plus surgical mask (HW + FM). Study nurses conducted home visits <24 hours of enrollment and on days 3, 7 and 21. RT-PCR was conducted on nose and throat swabs collected on all HH members at visits 0, 3 and 7. Hemagglutination inhibition (HI) assay was performed on acute/convalescent sera using antigens of circulating strains. A secondary infection was defined as a HH member that was either a RT-PCR positive or $\geq$4-fold rise in HI titers. **Results**: We analyzed data from 788 HHs (1995 HH members) that completed the 3 study visits between Apr 2008 and Feb 2011. The median index patient age was 5 years, and 724 (92%) slept in the same room with their parents. The secondary attack rate was 31% (624/1995; 95% CI: 29-33%) and 252 (41%) HHs experienced $\geq$1 secondary infection on day 3 or 7. In an intent-to-treat multivariate analysis adjusted for caregiver responsibility, proximity to the index patient and age, relative to the control arm the odds ratio of infection in a HH member was 1.13 (95%CI: 0.81-1.58) in the HW arm and 1.09 (95%CI:0.78-1.54) in the HW+FM arm. In the multivariate analysis of the actual reported frequency of HW on day 7, HW was not related to secondary infection (OR =0.95; 95% CI: 0.90 to 1.01); number of hours of FM use was positively associated (OR=1.07; 95% CI: 1.03 to 1.10).
**Conclusions**: Influenza transmission was high in Bangkok HHs. In contrast to the intent to treat estimate, the actual adjusted point estimate of reported hand washing was consistent with a mild protective effect though not statistically significant. Mask use was associated with a modestly increased risk of influenza infection; it may suggest that sharing the same bed each night is an overwhelming risk. Influenza vaccination remains the most effective means of prevention.

**Board 283. Do Kenyan Parents Want Their Children Vaccinated Against Influenza? Parental Attitudes towards Childhood Seasonal Influenza Vaccination Prior to and Following an Influenza Vaccination Campaign, Kenya, 2010–11**

**P.A. Oria**[1], J.M. Wong[2], D. Caselton[2], N.A. Otieno[1], E. Lebo[2], G. Emukule[2], P.M. Muthoka[3], D. Mutonga[3], J.A. Mott[2], R.F. Breiman[2], M.A. Katz[2];
[1]Kenya Medical Research Institute/Centers for Disease Control and Prevention (KEMRI/CDC), Kenya, Nairobi, Kenya, [2]Centers for Disease Control and Prevention (CDC), Kenya, Nairobi, Kenya, [3]Ministry of Public Health and Sanitation, Kenya, Nairobi, Kenya.

**Background:** Influenza vaccine is rarely used in Kenya, and little is known about attitudes towards the vaccine. In 2010, Kenya Medical Research Institute/Centers for Disease Control began a three-year observational seasonal influenza vaccine effectiveness study in children in two population-based infectious disease surveillance (PBIDS) communities: Kibera, an informal settlement in Nairobi; and

Lwak, a rural area in western Kenya. **Methods:** In 2010, free influenza vaccine was offered to children 6 months -10 years old enrolled in the two PBIDS communities with a combined total population of about 55,000 people. The vaccine campaign was preceded by an awareness campaign. We conducted focus group discussions with parents of children of enrollment age in the two sites prior to and following the vaccination campaign. **Results:** We conducted 4 group discussions prior to the vaccine campaign and 12 afterwards, with a total of 111 parents. Pre-vaccination, no parent had heard of the seasonal influenza vaccine. Most parents said low temperatures, dust, and smoke caused influenza, and said they preferred homemade remedies rather than hospital treatment for influenza. However, many parents were willing to have their children vaccinated provided they received more information about the safety and effectiveness of influenza vaccine. Post-vaccination, all parents knew about influenza vaccine. Out of 18,652 eligible children, 5,817(31.2%) were fully vaccinated, 2,073(11.1%) partially vaccinated and 10,762(57.7%) non-vaccinated. Most parents whose children were not vaccinated said the reason their children did not get vaccinated was that they were away during the vaccination period. In pre- and post-vaccination group discussions, some parents who did not plan to and/or did not vaccinate their children said they believed influenza was not severe enough to warrant vaccination and that it was normal to occasionally suffer from influenza. **Conclusions:** With awareness campaigns, some parents, previously unaware of influenza vaccine, were willing to have their children receive a free influenza vaccine. If seasonal influenza vaccine were to be introduced more broadly in Kenya, effective health messaging focusing on vaccine effectiveness, safety, and the potential severity of influenza could increase vaccine acceptance.

## Zoonotic and Animal Diseases

Tuesday, March 13
5:00 PM – 6:00 PM
Grand Hall

**Board 284. A Survey of Avian Influenza in Wild Birds and Domestic Ducks in Bangladesh**

**A. Islam**[1], A. Mikolon[1,2], M. Salah Uddin Khan[1], M.A. Rahman[1], S.K. Paul[1], A. Islam[3], M.Z. Rahman[1], M. Rahman[1], K. Vandegrift[3,4], J. Desmond[3], P.R. Hosseini[3], P. Daszak[3], S.P. Luby[1,2];
[1]International Centre for Diarrheal Disease Research (icddr,b), Dhaka, Bangladesh, [2]Centres for Disease Control and Prevention, Atlanta, GA, USA, [3]EcoHealth Alliance, New York, NY, USA, [4]Center for Infectious Disease Dynamics (CIDD), Penn State University, University Park, Pa, PA, USA.

**Background:** Wild waterfowl are the natural reservoirs for avian influenza viruses and responsible for persistence of these viruses in the wild. Freshwater wetlands (*haors*) in northern Bangladesh provide habitat for resident and migratory wild birds and domestic ducks during the cold season from November to March. The Bay of Bengal's coastal areas are also wintering grounds for migratory birds. We conducted a study of wild birds and domestic ducks in these areas to assess the presence of avian influenza viruses. **Methods:** We sampled wild birds from Hakaluki and Tanguar *haors* in two districts of northern Bangladesh and two coastal sandbars near Sonadia Island in southeastern Bangladesh. We used mist nets, noose traps and dazzling to capture these wild birds. We collected oropharyngeal and cloacal swabs from each bird in viral transport media (VTM). Cloacal swabs were also abtainedfrom domestic ducks (*Anas platyrhynchos*) from Hakaluki *haor*. Swab samples were tested by real time reverse transcriptase polymerase chain reaction (rRT-PCR) with specific primers to identify the influenza A Matrix (M) gene and the H5 subtype of hemagglutinin (HA) genes. **Results:** We sampled 472 wild birds of 67 species and 20 families from November 2010 to March 2011, of which 140 were resident and 332 were migratory. Among them, 275 were from Hakaluki *haor*, 130 from Tanguar *haor*, and 67 from

coastal sandbars. We detected 12 (2.5%, CI 1.3 - 4.4%) wild birds positive to influenza A by rRT-PCR. The influenza A infected birds from Hakaluki *haor* included 11 migratory waterfowl: nine northern pintails (*Anas acuta*), one ferruginous pochard (*Aythya nyroca*), and one garganey (*Anas querquedula*). From the coastal area, one lesser sand plover (*Charadrius mongolus*), a migratory wader, tested positive for influenza A. Birds sampled from Tanguar *haor* tested negative for influenza A. Among the 400 domestic ducks 32 tested positive for influenza A (8%, CI 5.5 - 11.1%). All influenza A positive birds and ducks tested negative for H5. **Conclusions:** Migratory wild birds and domestic ducks share habitat in Bangladesh and both carry and shed influenza A virus. Sampling wild birds in these areas and genotyping the viruses will allow identification of influenza virus strains that may represent a risk both to domestic poultry and to humans.

**Board 285. Vampire Bat Rabies as a Model One Health Approach towards Emerging Infectious Disease Prevention**

**A. Turmelle**[1], S. Recuenco[1], D. Alvarez[2], D. Moran[2], B. Petersen[1], J. Blanton[1], J. Ellison[1], C. Rupprecht[1]; [1]Centers for Disease Control and Prevention, Atlanta, GA, USA, [2]Universidad del Valle de Guatemala, Guatemala City, Guatemala.

**Background:** To understand the ecology of infectious disease emergence and perpetuation in wildlife and associated public health impact, it is critical to monitor pathogen diversity in reservoirs, seasonal ecology of vectors, community dynamics of multi-host systems and human interaction among at-risk populations. This study reports on the interrelationships between humans, bats and domestic animals in Guatemala, and evaluates evidence for pathogen exposure in humans and domestic animals associated with bat contact. **Methods:** In four communities experiencing vampire bat depredation, 490 people were interviewed between February and September 2010, and sera were collected from consenting respondents. The survey queried for animal bite history and associated illness, domestic animal ownership, the local availability of medical treatment and details regarding vaccination history. Enhanced surveillance among populations of bats occurred during 2009-2011 to characterize pathogen diversity among the bat fauna in eight areas, with a focus on the human survey locations. Radiotelemetry techniques were utilized to understand the dispersal and feeding behavior of hematophagous common vampire bats (*Desmodus rotundus*), whose affinity for livestock and humans contributes to their role as a major reservoir and vector of rabies virus throughout Latin America. **Results:** On average 10% of the human population in surveyed communities reported bat exposure, including bites, scratches or contact with unprotected skin, although 20% of respondents reported vampire bat depredation on domestic animals in one survey. Rabies virus neutralizing antibodies (rVNA) were detected from 10% of 291 free-ranging bats, implicating multiple bat species in rabies virus circulation. A high prevalence (48%) of *Bartonella spp.* infection was detected among 31 *D. rotundus*, a result of particular concern for public and veterinary health. Radiotelemetry monitoring of 16 vampire bats suggests philopatric feeding behavior at cattle farms, potentially leading to geographically stable population disease dynamics. **Conclusions:** Developing prevention and control measures to mitigate disease threats posed by vampire bats requires careful consideration of host and community disease dynamics to avoid potential for mismanagement.

**Board 286. Outbreak of *Salmonella enterica* serotype Enteritidis Infections Associated with Pet Guinea Pigs—Multiple States, 2010**

**M.L. Bartholomew**[1,2], R.T. Heffernan[2], J.G. Wright[1], S.A. Khan[1], R.F. Klos[2], T.A. Monson[3], J.P. Davis[2]; [1]Centers for Disease Control and Prevention, Atlanta, GA, USA, [2]Wisconsin Division of Public Health, Madison, WI, USA, [3]Wisconsin State Laboratory of Hygiene, Madison, WI, USA.

**Background:** Annually in the United States, *Salmonella* causes ~1 million infections and 400 deaths; infections can be acquired from nontraditional pocket pets. During October 2010, we investigated a

*Salmonella* serotype enteritidis (SE) sternal osteomyelitis case in a previously-healthy, 7-year-old who cared for 2 guinea pigs (1 newly purchased) that recently died. To determine a source, the investigation expanded when the patient's SE pulsed-field gel electrophoresis (PFGE) pattern matched a Michigan guinea pig isolate. **Methods:** A case was defined as a laboratory confirmed SE PFGE *XbaI* pattern JEGX01.0021 (outbreak strain) infection among persons exposed to guinea pigs during 2010. To locate outbreak strain isolates, PulseNet and USDA National Veterinary Service Laboratories (NVSL) databases were queried. States reviewed standard enteric interviews of persons with the outbreak strain for reports of guinea pig exposure. Persons reporting guinea pig exposure were re-interviewed with a detailed pocket pet questionnaire. All SE patients and guinea pig isolates underwent PFGE *XbaI* and *BlnI* testing and multilocus variable number tandem repeat analysis (MLVA). Traceback and environmental investigations were conducted at homes, pet stores, guinea pig distributors and breeders. **Results:** Cases were reported in 10 patients across 8 states. Nine illness onsets occurred May-November 2010. One patient was hospitalized; none died. Patient median age was 9.5 years (range: 1-61). Among 10 patients, 2 purchased guinea pigs from independent stores; 3 purchased guinea pigs from different locations of a national retail pet store (Chain A); 3 were Chain A employees; and 2 reported guinea pig exposures at unknown locations. Four NVSL SE guinea pig isolates matched the outbreak strain. All 14 isolates had PFGE *BlnI* pattern JEGA26.0002; MLVA revealed 3 related patterns. Tracebacks identified 4 primary guinea pig distributors and 92 sources supplying Chain A, including 1 breeder that potentially supplied guinea pigs to Chain A case stores. All environmental samples tested negative for *Salmonella*. **Conclusions:** A definitive source was not identified due to complex guinea pig distribution. Because guinea pigs and other pocket pets can harbor *Salmonella*, consumers and the pet industry should be educated regarding risks.

**Board 287. Influenza Virus Prevalence and Incidence at the Animal-Human Interface in a Rural Community in Vietnam in 2009 and 2011**

**B.K. Kapella**[1], N.T. Yen[2], N.C. Khanh[2], L.Q. Mai[3], T.N. Duong[3], N. Tung[4], N.D. Tho[4], P.T. Ha[3], J.C. Kile[1], N.T. Hien[3];
[1]U.S. CDC, Hanoi, Viet Nam, [2]National Institute of Hygiene and Epidemiology, Hanoi, Viet Nam, [3]National Institute of Hygiene and Epidemiology, Vietnam Ministry of Health, Hanoi, Viet Nam, [4]National Centre for Veterinary Diagnosis, Vietnam Ministry of Agriculture and Rural Development, Hanoi, Viet Nam.

**Background:** Several influenza pandemics in humans were caused by viruses from animals with genomic changes allowing the viruses to infect other species. In animals in Vietnam, influenza A is endemic, including H5N1 in poultry and H1N1 in pigs. In humans, Vietnam has sporadic cases of avian influenza A/H5N1, and year-round transmission of seasonal strains. Given population densities and biosecurity practices, opportunities for cross-species influenza virus transmission are frequent in Vietnam. However, there have been limited studies to identify the prevalence and incidence of influenza viruses at the animal-human interface. **Methodology:** A cross-sectional study in December 2009 and a cohort study from March to June 2011 were carried out in Thai Binh province in northern Vietnam. The 2009 study screened humans and animals in 30 households for influenza virus with swabs (throat swabs in humans, nose swabs in pig and cloacal swabs in poultry) and for antibodies to influenza with blood draws. The 2011 study followed humans and animals in 186 households for three months, swabbing humans with self-reported fever and cough or sore throat, and swabbing poultry and pigs with reported respiratory symptoms. If influenza A was found in any species, humans and animals in the same household were swabbed two weeks later. Swabs were tested for influenza by RT-PCR. Antibody titers were determined by haemagglutination inhibition, using a cutoff of 1:80. **Results:** The 2009 study identified 1 (0.8%) of 123 people infected with influenza A/H1N1/09 virus. Serologic evidence of past infection with influenza A viruses was identified in 65% of pigs, 17% of chickens, and 54% of ducks tested. In the 2011 study, two of 20 sick persons tested positive for A/H1N1/2009 and 3 for influenza B. Of two sick pigs and 13 sick

poultry, none were found positive for influenza A viruses. Of the household and animal contacts of the two A/H1N1/2009 positive persons, none of the 7 humans, 1 pig, and 4 poultry was positive for influenza. **Conclusions:** We found no clear evidence of transmission of influenza viruses between humans and animals in either study. However, evidence of past infection was high in poultry and swine. Additional studies at the animal-human interface are needed to better understand the influenza virus levels and transmission factors between species.

**Board 288. Emergence of Zoonotic Pathogens at the Human-Wildlife Interface: The Modeling Toolbox**

**K.A. Alexander**[1], B. Lewis[2], J.K. Blackburn[3,4], M. Marathe[2], S. Eubank[2];
[1]Virginia Tech, Blacksburg, VA, USA, [2]Virginia Tech, Virginia Bioinformatics Institute, Blacksburg, VA, USA, [3]University of Florida, Emerging Pathogens institute, Gainesville, FL, USA, [4]University of Florida, Spatial Epidemiology & Ecology Research Laboratory, Department of Geography, Gainesville, FL, USA.

**Introduction:** Increasingly, wildlife species have been identified as important sources of emerging zoonotic disease. These coupled systems identify complex and nonlinear interactions that have been historically difficult to evaluate, impeding our ability to manage zoonotic disease emergence. Mathematical models provide important tools to make the intractable elements of infectious disease research tractable. Explicit definitions of the interactions and parameter values of a system in an interactive and dynamic environment allow experimentation with the system. When applied to zoonotic diseases emergence, persistence and transmission, mathematical models can facilitate the evaluation and identification of important elements of the spatial and temporal features of pathogen spread and public and animal health strategy development. Often, however, there is a great divide between scientists conducting empirical studies on emerging zoonotic disease at the human-wildlife interface and the skill-base necessary to choose, develop and apply models to evaluate these systems. Which modeling approach should be employed? **Methods/Results:** Using Ebola and anthrax as comparative zoonotic disease examples, we assess the process of disease emergence, application of various modeling approaches (ecological niche models, patch models, compartmental models and agent based models), evaluate their strengths and weaknesses, and provide general guidelines for model selection in zoonotic disease investigations and public health control activities. **Conclusions:** Mathematical models are important tools in the study of zoonotic disease but there is a need for increased computational focus on the coupled dynamics of human and animal systems that underlie the process of zoonotic disease emergence. Model selection should be based on knowledge of the toolbox rather than current research practice of the scientist and/or collaborators; increased knowledge of options will help improve model application. Understanding and managing public and animal health threats associated with the emergence of infectious zoonotic disease will increasingly require scientists to fully engage modeling approaches that allow assessment across disciplines, animal communities, people and environments.

**Board 289. Seroprevalence of Leptospirosis and Toxoplasmosis in Children from Wisconsin**

**C. Munoz-Zanzi**, J. Williams-Nguyen;
University of Minnesota, Minneapolis, MN, USA.

**Introduction:** Leptospirosis and toxoplasmosis are among the most widespread and prevalent zoonoses in the world. In the U.S., overall sero-prevalence of toxoplasmosis in the population is 15%. Although no population-level sero-surveys have been carried out, leptospirosis in the continental U.S. is reportedly rare. Reported cases have commonly corresponded to sporadic clinical cases or occasional outbreaks due to exposure to contaminated water. Because of the limited knowledge of the status of these infections in the U.S., in particular in children, the objective of this study was to estimate their sero-prevalence in a group of children. **Methods:** The study population included 611 children from the state of Wisconsin who were part of the Marshfield Epidemiological Study Area and were participants of an unrelated study. Study participants were enrolled from children who were having a routine clinic visit

(well-child or other primary care visit). For the purpose of this study, we had access to banked sera and limited demographic information (age, sex, and whether they live on a farm). Serum samples were tested for *Leptospira* and *Toxoplasma* specific IgG antibodies using commercial ELISA kits. **Results:** Age of the children ranged from 1 to 18 years with a mean of 11.1 years. Fifty-six% were male and 36.5% lived on a farm. Regarding leptospirosis, 39/611 (6.4%) children were classified as positive to prior exposure to *Leptospira*. Although sample size was limited, there was no statistically significant difference between whether children living on a farm (4.5%) or not (7.5%), male (6.1%) or female (6.7%), or age (8.3% in 1-5 years old, 5.9% in 6 to 10 years old, 6.1% in >10 years old). Overall toxoplasmosis prevalence was 10.8%. Children living on a farm had a significantly higher prevalence (18.4%) than children not living on a farm (4.3%) (P < 0.001), while adjusting for age and sex. Sero-prevalence increased by age (P = 0.05) with 5.3% in 1 to 5 years old, 9.9% in 6 to 10 years old, and 13.6% in >10 years old. **Conclusions:** Results suggest that even in apparently low-risk populations or age groups, the true extent of these infections is larger than the number of clinically diagnosed cases. Pediatricians in the U.S. should be aware of these diseases, in particular toxoplasmosis in children from rural areas.

**Board 290. Zoonotic Pathogens and Blood Culture-Negative Infective Endocarditis in Northeast Thailand**

**H.C. Baggett**[1,2], O. Pachirat[3], G. Watt[1], P.-E. Fournier[4], M. Kosoy[5], S. Thamthitiwat[1], H. Lepidi[4], A. Paupairoj[3], V. Lulitanond[3], C. Paddock[2], N. Zeidner[5], L.F. Peruski[1,2], D. Raoult[4], S.A. Maloney[1,2];
[1]International Emerging Infections Program, Thailand Ministry of Public Health-US Centers for Disease Control and Prevention Collaboration, Nonthaburi, Thailand, [2]Centers for Disease Control and Prevention, Atlanta, GA, USA, [3]Faculty of Medicine, Khon Kaen University, Khon Kaen, Thailand, [4]University of the Mediterranean, Marseille, France, [5]Centers for Disease Control and Prevention, Fort Collins, CO, USA.

**Background:** Data on causes of infective endocarditis in Asia are lacking. Endocarditis cases for which routine blood cultures remain negative (blood culture negative endocarditis, BCNE) are common and severe. We examined etiologies and characteristics of infective endocarditis cases in Northeast Thailand, employing non-culture assays to enhance the standard diagnostic evaluation. **Methods:** Patients aged >15 years hospitalized in Khon Kaen during 2010-2011 who had suspected endocarditis by clinical signs and symptoms and echocardiogram were enrolled. Blood was cultured and tested by PCR for pathogens known to cause endocarditis. Serology was performed by immunofluorescent assay. Tissue obtained during cardiac surgery was cultured and tested by PCR. **Results:** The 66 patients with complete testing results enrolled to date had a median age of 46 years and 68% were male. Routine blood culture yielded a pathogen in 20 patients (30%). Among 46 (70%) patients with BCNE, enhanced testing confirmed a pathogen in 13 (28%), of which 10 (77%) were zoonotic: *Coxiella burnetii* (agent of Q fever, 5 cases), *Bartonella henselae* (2), *Streptococcus suis* (2), and *Bartonella vinsonii* (1). Other common pathogens were *Streptococcus sanguinis* (4), *Enterococcus sp*. (4), *S. agalactiae* (2), and *Staphylococcus aureus* (2). An etiology was established in 65% (24/37) of patients with surgical specimens vs. 24% (7/29) of non-surgical patients (p=0.001). No pathogen was identified in 35 (53%) cases. Seven (10.6%) patients died, one with *B. henselae*, one with *S. sanguinis*, and 5 with unknown etiology. **Conclusions:** Molecular and serologic testing for known endocarditis pathogens substantially improved diagnostic yield in a setting where 70% of cases had negative blood cultures. Zoonotic pathogens were the most frequently identified cause of BCNE cases. These first reports of Q fever and *Bartonella* endocarditis in Thailand and of *Streptococcus suis* endocarditis in Khon Kaen highlight the need to increase physician awareness and strengthen detection capacity to ensure appropriate treatment and improve understanding of the control and prevention of zoonotic endocarditis.

## Outbreak Investigation: Lab and Epi Response

Tuesday, March 13
5:00 PM – 6:00 PM
Grand Hall

### Board 291. Outbreak of Cyclosporiasis Associated with Multiple Events at a Georgia Catering Facility, July 2011

**H. Dishman**[1], P. Kolhe[2], S. Hernandez[2], K. Beidler[3], M. Tobin-D'Angelo[1], C. Drenzek[1], M. Park[1];
[1]Georgia Department of Public Health, Atlanta, GA, USA, [2]Fulton County Department of Health and Wellness, Atlanta, GA, USA, [3]Cobb and Douglas Public Health, Atlanta, GA, USA.

**Background**: On August 16, 2011, the Georgia Department of Public Health (GDPH) was notified of 2 possible outbreaks of gastrointestinal (GI) illness associated with catered events at Facility A on July 25, 2011 (Event 1) and July 30, 2011 (Event 2). Early illness reports described high attack rates, incubation periods of 1 week, and diarrhea lasting at least 3 days. **Methods**: The GDPH worked with the Fulton County Department of Health and Wellness (FCDHW) and Cobb and Douglas Public Health to distribute electronic surveys (n=150) to document attendees' symptoms and which food items were consumed. FCDHW also received reports of 5 Facility A employees with symptoms similar to attendees. FCDHW environmentalists inspected Facility A, collected menus and invoices for both events, and determined that 3 raw fresh produce items were common to both events: field mix, basil, and cherry tomatoes. **Results**: Approximately 100 people attended Event 1; of the 70 survey respondents, 66 (94%) reported illness after the event. Approximately 130 people attended Event 2; of the 39 respondents, 30 (77%) reported illness after the event. For both events, the median incubation period was 7 days and median illness duration was 10 days. The most common symptoms reported were diarrhea (median of 10 stools on worst day of symptoms), fatigue, and abdominal cramps. The Georgia Department of Agriculture visited the produce supplier and sampled the three common items; tests were negative for bacteria and parasites. On August 23, the Georgia Public Health Laboratory (GPHL) confirmed *Cyclospora* in an Event 2 attendee's stool specimen. On August 30, the GPHL identified *Cyclospora* in specimens from 2 Facility A employees who were not food handlers but consumed food prepared at Facility A. The field mix, basil, and cherry tomatoes were used to make salads served at both events. When salad consumption data were combined from both events, event attendees who ate salad (104, 97%) were 1.17 (0.93-1.48) times more likely to become ill than those who did not (3, 3%) (p=0.038). **Conclusions:** Salad was the most likely vehicle of foodborne transmission, although the contaminated ingredient was not determined. This finding is consistent with past *Cyclospora* outbreaks, which have been linked to "stealth vehicles" such as garnishes or other items not listed on menus or in recipes.

### Board 292. Mild Respiratory Illness Caused by H5N1 and H9N2 Infections among Young Children in Dhaka, Bangladesh, 2011

**A. Chakraborty**[1,2], K. Sturm-Ramirez[1,3], M. Khan[1], M. Haider[2], R. Sultana[1], N. Ali Rimi[1], M. Islam[1], A. Al Mamun[1], D. Goswami[1], A. Alamgir[2], M. Rahman[1], K. Jamil[2], A.D. Storms[3], E. Azziz-Baumgartner[3], K. Hancock[3], B. Shu[3], S. Lindstrom[3], N. Simpson[3], C. Todd Davis[3], A. Mikolon[1,3], W.A. Brooks[1], T.M. Uyeki[3], M. Rahman[2], S.P. Luby[1,3], M. Jahangir Hossain[1];
[1]International Centre for Diarrhoeal Diseases Research, Bangladesh (ICDDR,B), Dhaka, Bangladesh, [2]Institute of Epidemiology, Disease Control and Research (IEDCR), Dhaka, Bangladesh, [3]Centers for Disease Control and Prevention (CDC), Atlanta, GA, USA.

**Background:** Since 2007, highly pathogenic avian influenza A (H5N1) outbreaks in poultry have been reported each year in Bangladesh. One previous human case of H5N1 virus infection in Bangladesh was identified in Dhaka in 2008 through active surveillance for influenza among residents of Kamalapur, an

urban community. In March 2011, the same surveillance system detected 3 human cases of avian influenza A virus infection, including 2 H5N1 cases and a case of H9N2. A team of epidemiologists, veterinarians and anthropologists investigated the cases to assess transmission and to contain further spread. **Methods:** We collected clinical and exposure history on the cases. We recorded information on respiratory symptoms among persons who came within one meter of a case during 3 days before a case's illness onset to 7 days after last detection of H5N1 or H9N2 virus in follow-up nasopharyngeal wash (NPW). We collected serum samples from cases and from contacts who reported respiratory symptoms. NPW samples were tested at IEDCR and ICDDR,B for influenza viruses by RT-PCR. Serology, virus isolation and further characterization were done at CDC, Atlanta. **Results:** All 3 cases were <5 years old, previously healthy, and presented with fever, cough and/or runny nose. One H5N1 case had received oseltamivir treatment. All recovered without hospitalization and without complications. All cases had a history of poultry contact; the median time from contact to illness onset was 7 days (range: 7-10 days). Five of the 57 interviewed contacts of cases had a history of respiratory symptoms. None of the contacts had serological evidence of H5N1 virus infection. H5N1 viruses were isolated from two cases and identified as clade 2.2 viruses, closely related to highly pathogenic H5N1 viruses circulating among poultry in Bangladesh. A virus isolated from the third case was identified as a low pathogenic H9N2 virus, G1 lineage. All isolates were sensitive to 3 neuraminidase inhibitors.
**Conclusions:** Detection of mild pediatric cases of avian influenza A virus infection through active community surveillance suggests that additional cases may be undetected in other areas of Bangladesh and highlights the importance of surveillance in areas where avian influenza in poultry is endemic, in order to assess the risk of human infection with novel influenza A viruses.

**Board 293. Outbreak of Acute Encephalitis Syndrome, Muzaffarpur District, Uttar Pradesh, India**

**A. Kumar**[1], U. Singh[1], S. Aneja[2], T. Dikid[1];
[1]National Centre for Disease Control, Delhi, India, [2]Kalawati Saran Children's Hospital, Delhi, India.

**Background:** Acute encephalitis syndrome (AES) is endemic in India with outbreaks frequently reported. We conducted an epidemiological investigation of the AES outbreak in Muzaffarpur district, Bihar during June and July 2011 to identify potential risk factors. **Methods:** Hospital records were examined to identify all cases admitted between 11 June and 18 July fitting case definition of acute encephalitis (AE) and a line list created. Physicians and parents were interviewed. Questionnaires were completed on community, family and environmental factors in 26 randomly selected villages of the Muzaffarpur district; 13 villages with cases, 13 villages with no cases reported. Serum was collected from 9 cases and 8 contacts and tested for JE, Nippah and Chandipura viruses. **Results:** 147 records met case definition of AE. Of these 54 died (CFR 36.7%), 92% were <9 years with no adult cases. There was a bimodal peak distribution in mid June and at end of June showing a sharp decline thereafter. Most children were rural residents, usually 1 and rarely 2 per village. No siblings were affected. As per Interviews most patients had sudden onset of fever, without rash, depressed sensorium and convulsions progressing rapidly to unconsciousness. Lab testing was negative on samples collected. CT reports for 2 cases suggested encephalitis. 71% of deaths occurred within 48 hrs. 69% cases lived outside villages in lowlying areas near farms and litchi gardens and 77% regularly visited these. Rat burrows were seen in all the litchi gardens, farms. During June-July villagers temporarily live in litchi gardens with their children for litchi plucking early morning. More cases were seen in high litchi producing villages (OR= 7.32 95%CI- 3.9-13.7) and lowlying areas with sandy soil (OR= 5.8 95% CI- 3.7-9.3). Negative correlation seen between day of admission and daily rainfall (r= -0.2). **Conclusions:** Findings suggest an infectious cause of AE in this outbreak that occurs in children in litchi growing villages contiguous to low lying areas with sandy soil that was associated with pre-monsoon litchi plucking. We postulate that unique environment and behaviors were risk factors for transmission of this disease. Detailed studies on vectors, host reservoirs

and other environmental exposures should be conducted to further define at-risk behaviors, ecology and etiology.

**Board 294. Characteristics of Intentional Food Contamination Outbreaks: Results from the Foodborne Disease Outbreak Surveillance System, 1998–2008**

**S. Johnson**, L. Richardson, D. Cole;
Centers for Disease Control and Prevention, Atlanta, GA, USA.

**Background:** Outbreaks due to intentional food contamination are rare, but have become a greater concern among the public, public health professionals, and regulatory agencies. Outbreaks in the United States such as the 1984 Oregon salmonellosis incident in restaurants and the 1996 Texas *Shigella dysenteriae* type 2 incident among laboratory workers heightened awareness regarding the risk of bioterrorism through food contamination. **Methods:** Outbreaks due to intentional or suspected intentional introduction of a toxin, foreign substance, or pathogen in food occurring in the United States from 1998 to 2008 were ascertained from the Centers for Disease Control and Prevention's Foodborne Disease Outbreak Surveillance System (FDOSS) and from a review of published reports. Epidemiologic characteristics were analyzed using SAS version 9.2. **Results:** Of 13,405 foodborne disease outbreaks reported to FDOSS from 1998 to 2008, six outbreaks were reported to be the result of either actual or suspected intentional contamination. An additional two outbreaks were identified from published reports. Together, these eight outbreaks caused 252 illnesses and 1 death. Confirmed agents were arsenic, nicotine, and methomyl. Suspected agents were bleach, tetrahydrocannabinol, and other unidentified chemicals; all these were suspected based on supportive evidence. Symptoms and signs included nausea, diarrhea, vomiting, abdominal cramps, disorientation, dizziness, and numbness. Implicated foods were baked goods/desserts (n=4), coffee (n=1), ground beef (n=1), sauce condiment (n=1), and Thai dishes (n=1). Schools (n=4) were the most common exposure setting, and foods were reported as being prepared in the home in most of these (n=3). **Conclusions:** Outbreaks resulting from intentional contamination of food are rare. All known and suspected agents were chemical, which are known to have short incubation periods. The occurrence of half these outbreaks in school settings suggests that the index of suspicion for tampering in short incubation outbreaks should be higher in schools than in other settings.

**Board 295. Investigation of a Cryptosporidiosis Outbreak at a Summer Camp Using Traditional, Molecular, and Serology-Based Epidemiologic Methods**

**M.C. Hlavsa**[1], S.A. Collier[1], J. Priest[1], L. Xiao[1], L.S. Chang[1], G. Phillips[1], V.A. Roberts[1], A. Lowe[2], P. Hawkins[2], P. McFarland[2], P. Rocco[3], G. Bumby[3], J. Brunkard[1], J. Carpenter[1], V. Hill[1], E.L. Dunbar[1], J.S. Yoder[1], J.M. Roberts[1], B.A. Mathison[1], E.M. Meites[1], S. Smith[2], M. Salyers[3], A. Fleishauer[3], M.J. Beach[1], C. hominis Outbreak Investigation Team[1,3];
[1]Centers for Disease Control and Prevention, Atlanta, GA, USA, [2]Transylvania County Department of Public Health, Brevard, NC, USA, [3]North Carolina Division of Public Health, Raleigh, NC, USA.

**Background:** Infection by *Cryptosporidium* oocysts can occur following ingestion of contaminated food or water or contact with infected persons or animals. Symptomatic infection is less likely in those previously infected. During the week of June 21, 2009, attendees and staff at a summer camp began experiencing diarrhea. By June 30, four laboratory-confirmed cryptosporidiosis cases and >30 cases of diarrhea were identified. Potential obstacles to identifying the outbreak source included multiple possible sources (e.g., camp-grown produce and pre-weaned calves) and possible previous *Cryptosporidium* infection in staff. **Methods:** The investigation included traditional, molecular, and serology-based epidemiology. A case was defined a person who was at the camp June 20-26, 2009 and had gastrointestinal symptom onset after June 21. A retrospective cohort study of camp staff was conducted. Human and animal stool specimens were tested for *Cryptosporidium*; isolates were subtyped

using PCR assays for 18S rRNA and the GP60 gene. Serum samples were assayed for IgG antibodies to 17- and 27-kDa *Cryptosporidium* antigens. **Results:** The investigation identified 46 cases in attendees and staff; symptom onset peaked June 26-27. Ham served June 21 (adjusted prevalence ratio [aPR]=3.5; 95% confidence interval [CI] = 1.6-7.4) and cabin contact with an ill person (aPR=2.8; CI=1.3-6.2) were significantly associated with illness. Isolates from 7/7 humans and 4/5 animals were of the identical *C. parvum* subtype, IIaA17G2R1. Of 123 study participants, 64 (52%) gave serum samples. All but one ill serosurvey participant (14/15 or 93%) had serologic evidence of recent *Cryptosporidium* infection; 12/49 (24%) had an antibody response but not illness meeting the case definition. **Conclusions:** Traditional epidemiology revealed a point-source exposure and possible foodborne transmission. Molecular epidemiology indicated that *Cryptosporidium* transmission was likely zoonotic, suggesting food served as the proximate vehicle of transmission. Additionally, some serosurvey participants were recently infected with *Cryptosporidium* but did not have illness meeting the case definition. A combination of traditional, molecular, and serology-based epidemiologic methods can provide a more complete picture of *Cryptosporidium* transmission.

**Board 296. Clinical Comparison of Pediatric Hemolytic Uremic Syndrome Cases, with and without Stool Culture Confirmation of Shiga Toxin Positive *E. coli*; New York State Emerging Infections Program, 1999–2011**

**N.L. Spina**[1], K.P. Malloy[1], G.L. Smith[2], N.B. Dumas[3], S.M. Zansky[1];
[1]NYS Dept. of Health, Albany, NY, USA, [2]NYS Dept. of Health, Rochester, NY, USA, [3]Wadsworth Center Laboratory, Albany, NY, USA.

**Background:** The New York State Emerging Infections Program (NYSEIP) performs active surveillance for Shiga-toxin producing *E. coli* (STEC) and Hemolytic Uremic Syndrome (HUS). Pediatric (P) HUS is characterized by acute renal failure, hemolytic anemia and thrombocytopenia and is commonly a result of STEC infection. Cases of STEC associated HUS often present with bloody diarrhea, but identification of STEC can be difficult due to the wide variety of non O157 serotypes and limited testing capabilities in clinical laboratories. Because antibiotic usage in STEC cases may exacerbate HUS symptoms, ruling out infection is clinically important. Laboratories are encouraged to submit HUS+, STEC O157 (-) stools to the Wadsworth Center Laboratory (WCL) to rule out the presence of a non O157 STEC. **Methods:** The EIP utilizes a standardized HUS form developed by the CDC for data extraction. All medical records from HUS cases reported from the 34 county EIP catchment area were reviewed from 1999 - 2011. Clinical factors, symptoms, laboratory results, and sequelae were documented. For this analysis, we compared pediatric (< 18 years of age) HUS cases (P/HUS) with diarrhea (D+) that were STEC+ and those with unknown diarrheal illness etiologies. Analyses were performed using SAS v9.2. **Results:** Sixty-seven P/HUS, D+ cases were identified. There were no significant differences in age, gender, laboratory results or sequelae between P/HUS cases with STEC and those with unknown etiology. 45 of the 67 P/HUS cases (67%) were STEC+. 42 of these (93%) stools were bloody. Of the 22 (33%) cases with unknown etiology, 20 (91%) were culture negative at the clinical laboratory for O157, 11 (50%) of the stools were bloody and 2 (9.1%) cases had no testing. **Conclusions:** Clinicians should be aware that clinical laboratories cannot definitively identify an STEC non O157. If a child shows classic signs of HUS, further testing should be performed at the WCL to rule out a non O157 STEC. When an etiology is identified, clinical decisions may be altered, guiding appropriate treatment and may prevent unnecessary testing. Understanding temporal and geographic trends can prompt clinicians to test accordingly when STEC may be suspected. Communication between the clinicians and the laboratory could expedite pathogen identification if referral to the WCL is required.

## Vectorborne Diseases and Climate Change

Wednesday, March 14
12:30 PM – 1:30 PM
Grand Hall

**Board 297. Seroprevalence of Rickettsia-specific Antibodies in Young Male Volunteers in Azerbaijan**

**E. Garges**[1], A. Richards[1], A. Seyidov[2], E. Nasirova[3], R. Rivard[3], H. Dyson[4], T. Ahmadkhanov[2], N. Huseynov[2]; [1]Walter Reed Army Insitiute of Research, Silver Spring, MD, USA, [2]Ministry of Defense, Republic of Azerbaijan, Baku, Azerbaijan, [3]U.S. Army Research Institute of Infectious Diseases, Ft Dertirck, MD, USA, [4]Defense Science and Technology Laboratory, Porton Down, UK.

**Background:** Rickettsial diseases, to include both typhus group (TGR) and spotted fever groups (SFGR) show considerable variation in their regional epidemiology. Prior studies from the Southern Caucasus region of Eastern Europe demonstrate a variety of regional spotted fever rickettsiae to include *R. sibirica*, *R. conorii* and *R. akari*. In addition, *Rickettsia typhi*, the pathogenic agent of murine typhus is also known to be prevalent in the region. Limited specific information about the epidemiology of rickettsial disease has been reported in the country of Azerbaijan. However, recent studies of known tick vectors in the country have demonstrated the presence of human pathogenic rickettsia. We undertook an investigation to evaluate seroprevalence of prior exposure to a number of regional pathogens to include both spotted fever group (SFGR) and typhus group rickettsia (TGR) among men entering into military service under the Azeri Ministry of Defense. **Methods:** Male volunteers were enrolled in the study as part of the routine medical in-processing. Detailed questionnaires containing recent and remote medical history, relevant exposure histories, and health seeking behaviors were collected as well as a specimen to determine serologic evidence of prior exposure. Titers specific for both typhus group and spotted fever group rickettsia were determined locally using previously published protocols. **Results:** Of the 545 study participants evaluated for prior rickettsia exposure, none (0/545) demonstrated exposure to typhus group rickettsia, however, 3% (17/545) demonstrated a confirmatory titer against spotted fever group rickettsia. Demographic data, medical history, and relevant exposures to potential vector were not significantly associated with seropositivity to spotted fever group rickettsia. **Conclusions:** The total absence of serologic evidence of typhus group rickettsia was surprising and suggests a more epidemic nature of murine typhus in the study population. Further studies need to be conducted to determine which rickettsia or rickettsiae are responsible for inducing the SFGR-specific antibodies. This study demonstrates that rickettsia exposure occurs, though at a low level, among young male volunteers or is focal and regional and as such, not widespread among the population.

**Board 298. Armed Forces Health Surveillance Center's Global Febrile and Vector-borne Disease Surveillance Program**

**A.C. Thomas**, D.L. Blazes, T.E. Myers, R.L. Burke;
AFHSC-GEIS Operations, Silver Spring, MD, USA.

**Background:** Originally established by Presidential Directive NSTC-7 in 1997, the Department of Defense (DoD) Global Emerging Infections Surveillance and Responsive System (GEIS) was expanded in 2006 and subsequently incorporated as a Division of the Armed Forces Health Surveillance Center (AFHSC) in 2008. AFHSC-GEIS divides its surveillance efforts into five categories: respiratory, gastrointestinal, and febrile and vector-borne infections, as well as antimicrobial resistance and sexually transmitted infections. **Methods:** The goal of the GEIS febrile and vector-borne illness (FVBI) surveillance program is to integrate pathogen discovery, febrile illness, arthropod-vectors and animal surveillance systems that contribute to Force Health Protection and global public health. The primary objectives of the program are: 1) Characterize the geographic distribution and transmission of FVBI pathogens and their

antimicrobial susceptibilities; 2) Characterize febrile illness risk and outbreak threats based on surveillance and modeling results; 3) Provide actionable information for health professionals and DoD decision makers on patient care and outbreak prevention and control; and 4) Promote research training and capacity building collaborations. **Results:** Recent accomplishments of the program include the accurate forecasting of the 2010 Rift Valley Fever outbreaks in South Africa, the creation of an accurate model for predicting Japanese encephalitis (JE) risk in Korea, and the establishment of VectorMap and its subsidiary components. Our partners have also identified the novel Iquitos virus that has caused febrile illness outbreaks in the Amazon region of Peru, and have assisted with dengue outbreak investigations in multiple countries around the world, including Peru, Kenya and Djibouti. **Conclusions:** AFHSC-GEIS heads a centrally-coordinated worldwide network of partners comprised of US-based laboratories, which conduct FVBI surveillance in US military service members and their dependents, and DoD overseas research laboratories, which conduct surveillance among foreign military and civilian populations in partnership with the ministries of defense or health of partner nations. The FVBI pillar has funded over 50 competitive proposals in the past two years in over 30 countries.

**Board 299. Phylogenetic Analysis of Eastern Equine Encephalitis Virus Detected in New Hampshire between 2004 and 2010**

**F. Gao**[1], C. Loring[1], N. Goodyear[2], D. Bolton[1], C. Bean[1];
[1]New Hampshire Department of Health and Human Services, Concord, NH, USA, [2]University of Massachusetts Lowell, Lowell, MA, USA.

**Background:** Eastern equine encephalitis virus (EEEV) is endemic in the northeastern United States. In 2005, 7 cases of EEEV human disease were confirmed in New Hampshire with 2 of these cases proving fatal. New Hampshire Department of Health and Human Services (NH DHHS) began environmental surveillance for EEEV in 2004 through testing of mosquito batches and wild birds, and disease diagnosis in humans and domestic animals. The purpose of this study was to investigate the nature of population dynamics of the EEEV in New Hampshire through phylogenetic analysis of virus detected during 2004-2010. **Methods:** Archived EEEV positive specimens including mosquito homogenates, avian brain homogenates, equine brain homogenates and alpaca brain homogenates were used in this study. These specimens were collected from June 2004-August 2010 as part of surveillance efforts by the NH DHHS. RT-PCR was performed to amplify NSP3 region of EEEV followed by DNA sequencing. Phylogenetic analyses were conducted by neighbor joining (NJ), maximum parsimony (MP), and maximum likelihood (ML) methods using MEGA 4.0 and PHYLIP. **Results:** PCR and sequencing were successfully performed on 55 specimens representing different host species, temporal and geographical locations and all years representing 2004-2010in the state of New Hampshire. All 55 strains were collected in southern New Hampshire, primarily in the south central and southeast regions. Phylogenetic analysis of the NSP3 sequences revealed the presence of 4 different clades within the specimens isolated in NH. Clades 1 and 2 include strains from multiple years, while clade 3 includes a single strain from the year 2004 and clade 4 includes only specimens collected in 2009. All clades except clade 3 include strains collected in various locations in the state. **Conclusions:** Multiple mechanisms are involved in maintenance of EEEV in New Hampshire. Closely related sequences from specimens collected from multiple years indicated that some strains of virus are able to persist in the environment, overwintering for several years. At the same time, the identification of unique sequences each year suggests that novel EEE viruses have been introduced to the state repeatedly.

**Board 300. Sentinel Febrile Epidemiological Surveillance for Early Detection of Cases of Dengue in the City of Lima during the Years 2009–2010**

**M.J. Loayza, Sr.**, G.A. Cisneros, G. Yale, M.A. Lorenzo;
Ministry Health Peru, Lima, Peru.

**Background:** Dengue is similar to other febrile diseases especially in its early stages. Diagnosis is often delayed or confused with other diseases, delaying the clinical and epidemiological surveillance in areas where there is increased risk of dengue epidemic transmission (north of Lima) causes outbreaks such as occurred since 2005. The aim of this study is to evaluate the diagnostic usefulness of fever surveillance for early detection of cases of dengue in the city of Lima. **Methods:** Cross sectional study. We included patients reported in the Sentinel Surveillance System for Fever for the years 2009 and 2010. Was defined as "febrile dengue" any person with onset of fever (≥ 38 ° C), identified within 72 hours of onset, which comes from an endemic area for dengue (last 7 days) and that this focus apparent clinical of another disease. Data were recorded on sheets of epidemiological reporting.Patients Blood samples were taken. We determined the sensitivity, specificity, positive predictive value and negative case definition, compared with the results of IgM and IgG by ELISA and PCR. **Results:** Of 4712 febrile patients treated at facilities sentinels, 471 cases were identified with the definition of “case of dengue fever.” Of these 321 (68.15%) were positive for IgM antibody detection of specific dengue, indicating primary infection and in 108 cases (22.9%) were obtained by PCR serotypes: DEN-3 (57.4%), DEN-1 (36.1%) and DEN-4 (6.5%). With the results of serology for the case definition, we obtained a sensitivity of 95.5% (CI95: 92.6-97.4), specificity of 96.6% (CI 95: 96-97.1), positive predictive value of 68.2% (95: 63.7-72.3) and negative predictive value 99.6% (CI95: 99.4-99.8). **Conclusions:** Epidemiological surveillance for dengue fever in endemic areas shows that using epidemiological parameters and an adequate medical history can be detected early in patients with dengue, conducting clinical research and epidemiological surveillance quickly to prevent the transmission of cases.

## Outbreak Investigation: Lab and Epi Response

Wednesday, March 14
12:30 PM – 1:30 PM
Grand Hall

**Board 301. Suspected Polio Investigation—Indiana, 2011**

**A. Cierzniewski**;
Indiana State Department of Health, Indianapolis, IN, USA.

**Background:** On May 18, 2011, the Indiana State Department of Health (ISDH) Laboratory notified the State Epidemiologist that a polio virus had been isolated from a patient specimen. The patient was an 81-year-old retired female who lived in a long-term care facility. She was hospitalized May 5-9 with pneumonia and later fully recovered. **Methods:** A nasophayngeal swab collected during the patient’s hospitalization tested negative for enterovirus at a reference laboratory. The swab was sent to the ISDH Laboratory and placed in viral culture. The culture reacted with an enterovirus pan-polio antibody. The isolate also tested positive for enterovirus by PCR. However, two different molecular sequencing protocols failed to detect enterovirus. The ISDH Laboratory initially analyzed the specimen using a routine, validated enterovirus sequencing protocol. CDC Enterovirus Laboratory experts suggested that the specimen could be tested using an alternative published protocol; results were negative. The epidemiological investigation indicated the patient had no known exposure to polio. The isolate was sent to CDC for further analysis. **Results:** Preliminary RT-PCR performed on May 20 at CDC was positive for PV3-Sabin- like virus, similar to positive controls recently used at the ISDH Laboratory, suggesting laboratory contamination of the specimen. Positive polio controls used at the ISDH Laboratory were sent to CDC, and nucleic acid sequencing matched the patient specimen exactly, confirming laboratory contamination. **Conclusions:** An investigation launched to determine the source revealed that positive polio controls were not in culture while the patient specimen was tested. The most likely cause of the

contamination was inadequately decontaminated pipettes. Additional disinfection procedures were instituted as a result of the investigation. This incident underscores the importance of considering laboratory contamination when unusual results are identified, especially if the epidemiologic investigation reveals no risk factors, to prevent possible unnecessary effort and expenditure of resources. Suspected polio specimens should be forwarded to the CDC Enterovirus Laboratory.

**Board 302. All That Bugs Is not in Bed: Bedbug Infestation of an Office Building—Clarksville, Tennessee, 2011**

**J.G. Baumblatt**[1], J.R. Dunn[2], W. Schaffner[3], A.C. Moncayo[2], A. Stull-Lane[4], T.F. Jones[2];
[1]Epidemic Intelligence Service Officer at Tennessee Department of Health, Centers for Disease Control and Prevention, Atlanta, GA, USA, [2]Tennessee Department of Health, Nashville, TN, USA, [3]Department of Preventive Medicine, Vanderbilt University School of Medicine, Nashville, TN, USA, [4]Association of Public Health Laboratories and Centers for Disease Control and Prevention Emerging Infectious Diseases Fellow at Tennessee Department of Health, Nashville, TN, USA.

**Background:** Since 2000, a resurgence in bedbugs (*Cimex lectularius*) has occurred in the US; they are now considered common urban pests. Reports of infestations of homes, hospitals, hotels, and offices have been described, and substantial difficulties are involved in their detection and elimination. On September 1, 2011, complaints of itching and bug bites among workers in an office were reported to the Tennessee Department of Health. **Methods:** We performed a retrospective cohort study. A case was defined as unexplained skin lesions or itching at any time during July 9-September 9, 2011, in a worker in Office A. We administered a standardized questionnaire to assess symptoms and exposures in the work environment. Situational anxiety was assessed on a scale of 1 (low) to 10 (high). Analyses were performed by using Epi-Info™. Dogs certified to detect evidence of live bedbugs were used, and arthropod samples were collected. Some workers with skin complaints obtained a medical evaluation. **Results:** Of 76 workers in the office, 61 (80%) were interviewed; 35 (57%) cases were reported. Pruritic maculopapular lesions observed were consistent with arthropod bites. Arthropods were identified morphologically as bedbugs by two entomologists. Confirmatory genetic identification is pending. In bivariate analysis, the following exposures were associated with case status; working in a cubicle adjacent to one in which a dog identified bedbugs (risk ratio [RR], 4.3; 95% confidence interval [CI], 1.02-18.38), self-reported seasonal or other allergies (RR, 3.6; CI, 1.24-10.5), and female sex (RR, 7.6; CI, 1.38-41.94). In this cohort, the median situational anxiety score was 4.0. **Conclusions:** An ongoing bedbug infestation in this office building caused dermatologic manifestations and substantial anxiety among staff. We recommended the use of a pest control professional and provided education about bedbugs not being disease vectors. Bedbugs represent an emerging and challenging environmental problem with clinical, psychological, social, and financial impact.

**Board 303. Pulsed-field Gel Electrophoresis (PFGE) Analysis of *Clostridium botulinum* Isolates during Botulism Outbreak Investigations**

**L.A. Joseph**, J.K. Dykes, S.E. Maslanka, C. Luquez;
Centers for Disease Control and Prevention, Atlanta, GA, USA.

**Background:** Cases of foodborne botulism are considered public health emergencies because widely distributed contaminated products could affect many patients across state and international lines. Subtyping methods are necessary for separating outbreak-associated cases of botulism from sporadic cases as well as identifying the source of the outbreaks. Pulsed-field gel electrophoresis (PFGE) is the standard method for subtyping *Clostridium botulinum* isolates from outbreak investigations at the Centers for Disease Control and Prevention (CDC). To determine the efficacy of PFGE during botulism outbreak investigations, we evaluated multiple *C. botulinum* isolates taken from clinical, food, or environmental samples. **Methods:** Multiple isolates of *C. botulinum* recovered from specimens during

outbreak investigations (n=57) were analyzed by *SmaI* PFGE including isolates from human (n=204), food (n=88), and environmental (n=1) samples. The resulting PFGE patterns were compared using UPGMA clustering (tolerance=1.5%, optimization=1.5%) with the Dice coefficient in BioNumerics 5.01. **Results:** Thirty-five different patterns were observed after analysis of 293 isolates (57 outbreaks) by *SmaI* PFGE. Isolates within a majority of the epidemiologically-linked outbreaks were indistinguishable by *SmaI* PFGE (91%; 52/57). Moreover, the two most common PFGE patterns in this study were produced by isolates within 17 of the 57 outbreaks examined (30%). In addition, PFGE patterns from clinical Isolates matched those of the implicated food and environmental samples within an outbreak. **Conclusions:** *C. botulinum* PFGE can be an important tool for use during botulism outbreak investigations by linking isolates from clinical samples taken from different patients or connecting a clinical sample to an implicated food source. However, interpretation of the PFGE data should be conducted within an epidemiological context during botulism outbreak investigations.

**Board 304. An Outbreak of *Salmonella* Weltevreden Infections Detected by a Lab-based Active Surveillance System in Guangdong Province, China**

**X. Huang**, J. Liang, B. Ke, X. Deng, Q. Huang, L. Lu;
Guangdong Provincial Center for Disease Control and Prevention, Guangzhou, China.

**Background:** Salmonella is one of the leading bacterial causes of human foodborne illness worldwide. For better strengthening national and regional foodborne disease and pathogen surveillance and response, thus to reduce the global foodborne disease burden, a network named WHO Global Salm-Surv (renamed as Global Foodborne Infections Network now) was initiated by the World Health Organization (WHO), the Danish Veterinary Laboratory (DVL) and the Centre for Disease Control and Prevention(CDC) in January 2000, using Pulsed-Field Gel Electrophoresis (PFGE) for molecular subtyping of bacterial pathogens and multi-places outbreaks detecting . Guangdong joined the network in September 2008, establishing PulseNet-Guangdong, a lab-based surveillance system for non-Typhoid Salmonella and had been putting it into practice ever since. In September 20, 2010, 5 cases with the same Salmonella serotype, *Weltevreden*, a very rare type in Guangdong, as well as with the same PFGE pattern, was detected through the system, but with no outbreak or case report from clinical doctors. Based on the laboratory detection, we conducted an epidemiological investigation, and finally verified the outbreak of Salmonella *Weltervreden* infections. **Methods:** We conducted molecular subtyping to Salmonella strains collected from a total of 17 sentinel hospitals in 8 cities of Guangdong province with PFGE, and analyzed patterns with Bionumerics, to see if there were still other cases with the same Salmonella serotype and PFGE pattern, and the illness onset interval between any two of them were no longer than 14 days. Designed a uniform questionnaire for case interview, carried out an on-site investigation and collected suspected food and environmental samples from a referred restaurant for Salmonella isolation. **Results:** A restaurant related outbreak, with 5 cases infected by Salmonella *Weltevreden*, was detected by PulseNet-Guangdong, a lab-based active surveillance system, and verified by the epidemiological investigation. With no supportive laboratory results on food and environmental samples, the vehicle, suspected to be emu, and the source, speculated to be cross contamination, remained to be uncertain. **Conclusions:** This foodborne disease outbreak cause by a rare strain of Salmonella, *Weltevreden*, was the first foodborne outbreak detect by the lab-based surveillance system and later verified by the epidemiological investigation. It demonstrated that the lab-based active surveillance system did play a role in fast and sensitive detection of outbreaks, as well as strongly recommended that China should pay more attention to the application of this kind of new technology in the foodborne disease surveillance.

**Board 305. Human *Salmonella* Infections Associated with Exposure to Live Poultry from Agricultural Feed Stores and Mail-Order Hatcheries**

**J. Mitchell**[1], K. O'Connor[1], K. Machesky[2], B. Byrum[3], T. Forshey[3], L. Hausman[1], T. Gomez[4], C. Roney[5], C. Barton Behravesh[1];
[1]Centers for Disease Control and Prevention, Atlanta, GA, USA, [2]Ohio Department of Health, Columbus, OH, USA, [3]Ohio Department of Agriculture, Reynoldsburg, OH, USA, [4]United States Department of Agriculture, Animal and Plant Health Inspection Service, Atlanta, GA, USA, [5]United States Department of Agriculture, National Poultry Improvement Plan, Conyers, GA, USA.

**Background:** *Salmonella* infections from contact with live poultry (chickens, ducks, turkeys, geese) continue to be a public health problem. An increasing number of people around the country are choosing to raise live poultry in backyard flocks. Live poultry are commonly distributed through agricultural feed stores and mail-order hatcheries; approximately 50 million chicks are sold annually in the U.S. Since 1990, >30 outbreaks of human *Salmonella* infections linked to contact with live poultry from mail-order hatcheries have been reported. **Methods:** In the summer of 2011, two clusters of human *Salmonella* infections were identified through PulseNet, the national molecular subtyping network for foodborne disease surveillance. Standard outbreak investigations and product tracebacks were conducted. **Results**: Between 05/27/11-08/23/11, 65 individuals infected with the outbreak strain of *Salmonella* Altona and 27 individuals infected with the outbreak strain of *Salmonella* Johannesburg were reported from 23 states. Infected individuals ranged in age from <1 year to 92 years; 32% were =<5 years of age. Seventy-six percent (41/54) of *Salmonella* Altona and 71% of (17/24) *Salmonella* Johannesburg case-patients reported contact with live poultry in the week before illness began. Most patients reported purchasing chicks and ducklings from multiple locations of a nationwide agriculture feed store; live poultry were purchased for either backyard flocks to produce eggs or as pets. **Conclusions:** These outbreaks highlight the ongoing risk of human *Salmonella* infections associated with live poultry contact, especially to young children. In response to this ongoing public health problem, a collaborative effort among officials with the USDA's National Poultry Improvement Plan, local, state, and federal public and animal health agencies, the mail-order hatchery industry, and other partners was organized to develop and implement a comprehensive *Salmonella* control strategy. Educational materials warning customers of the risk of *Salmonella* infection from live poultry contact and measures they can take to prevent *Salmonella* infections should be distributed with all live poultry purchases. Preventing these infections will require an integrated approach at the hatchery, agricultural feed store, and consumer levels.

**Board 306. Two Large Diarrheal Disease Outbreaks Occurred in an Important Touristic Coastal City of Sao Paulo State in 2009 and 2010—What Did It Change to Improve the Public Health Practices to Prevent New Outbreaks?**

**M.B. Eduardo**[1], E. Suzuki[1], J. Fred[1], A.T. Plaça[2], L.M. Lima[2], L.A. Quitério[3], A.F. Ribeiro[1];
[1]Epidemiological Surveillance Center, State Secretary of Health of São Paulo, São Paulo, SP, Brazil, [2]Municipal Secretary of Health of Guarujá, Guarujá, SP, Brazil, [3]Group of Sanitary Surveillance of Santos, State Secretary of Health of São Paulo, Santos, SP, Brazil.

**Background:** Several diarrheal disease outbreaks have occurred in the last three years in touristic coastal cities of São Paulo associated with water or foods, especially in the Christmas season. We report two large diarrhea outbreaks that occurred in Guarujá city, from December 2009-January 2010 and December 2010-February 2011, and control and prevention measures. **Methods:** Descriptive studies were developed in both periods with diarrhea cases registered by the Sentinel Health Centers. Patients were interviewed to identify the source of transmission. Samples of stool, food and water were analyzed by conventional and molecular techniques. Investigations were conducted by the municipal team involving the Epidemiological Surveillance Center and other partners. **Results:** The first outbreak caused

by norovirus involved 6400 cases (incidence rate = 2074/100,000 inhabitants) associated with the public drinking water system. Epidemic curve showed a continuous exposition of patients to primary infection source. The second outbreak was caused by various pathogens, predominantly *E. coli* bacteria involving 5600 (incidence rate = 1261/100,000 inhabitants). Three risk factors were associated: consumption of raw fresh vegetables, drinking water and swimming in the sea. **Conclusions:** These investigations showed a disordered urbanization with population in areas without public sewer system, fecal contamination of rivers and beaches and an inadequate public drinking water system in a touristic city which triplicates its population in the Christmas season. The major sanitary measures were: implementation of the diarrhea surveillance and laboratorial tests, implantation of laboratorial techniques to improve the monitoring of the water and beaches, reconstruction of public drinking water system to guarantee the safety and quality of water and enhancement of sanitary inspections of foods. Other measures as extension of public sewer system for the population and treatment of sewer are being developed and denote the efforts to improve the public health practices to prevent new outbreaks.

## Laboratory Proficiency Testing/Quality Assurance

Wednesday, March 14
12:30 PM – 1:30 PM
Grand Hall

**Board 307. A Multidrug-Resistant Tuberculosis Exposure among Adolescents in Overseas U.S. Army Communities**

**J.V. Woodring**[1,2];
[1]US Army, Vilseck, Germany, [2]Bavaria Army Medical Department Activity (BMEDDAC), Vilseck, Germany.

**Background:** A 17 year old Kenyan immigrated to Germany, was found to have tuberculosis within three months of arrival to Germany and was started on empiric treatment. Four months into therapy, culture sensitivities showed multidrug-resistant tuberculosis (MDR TB) and the patient was subsequently inadequately treated with two drug therapy for the next eight months. Reactivation of this index case's MDR TB while attending a U.S. military high school resulted in a contact investigation to identify potential exposures who possibly might have TB and those would require treatment for multidrug resistant latent tuberculosis infection (MDR LTBI). **Methods:** Potential exposures to the reactivated MDR TB index case were screened with QuantiFERON-TB Gold In-Tube test (QFT-GIT) testing within two weeks of last exposure to the index case and nine week later. **Results:** For the initial QFT-GIT screening, 14% (19/140) were indeterminate and less than 1% (2/140) was positive. Follow up QFT-GIT testing nine weeks later revealed 10% (14/140) indeterminates and 6% (9/140) positives. Given the potential risk of seroconversion in predominantly classmates who only shared one class with the index case, follow up indeterminate results were finalized with a tuberculin skin test (TST) and resulted in 1 of these 14 exposures with a TST over 5 mm. Cultures sensitivities resulting from the reactivated index case eventually revealed solely isoniazid and rifampin resistance. Patients who had a remote history of untreated LTBI were started on isoniazid, while recent seroconversions were treated with six months of ethambutol and moxifloxacin. All patients receiving drug sensitive or drug resistant LTBI therapy have tolerated their treatment regiments well. **Conclusions:** To date, QFT-GIT and other Interferon Gamma Release Assays (IGRAs) are poorly studied among adolescents, with most children IGRA studies limited to five years olds and younger. The current study's high rate of initial and follow up indeterminates among 14 to 19 year olds questions the efficacy of using this screening tool for this age group. Further

studies are required to determine if this potential limitation of high rates of indeterminates affects other clinical studies involving adolescents.

**Board 308. Assuring the Quality of HIV Tests in Zambia**

**K.G. Musonda**[1], K.K. Musukwa[1], C.B. Ndongmo[2], S.M. Mwangala[1], G.M. Ngwele[1], C.C. Chisenga[1], M.A. Monze[1], ZANQAP Team;
[1]Virology, Immunology & Molecular Biology Unit, Dept of Pathology & Microbiology, University Teaching Hospital, Lusaka, Zambia, [2]CTS Global, Inc., Lusaka, Zambia.

**Background:** In Zambia the HIV prevalence among adults stands at 14.3%. There has been rapid expansion of HIV programs, driven by the desire to make HIV testing readily available to the citizenry. Consequently a sharp rise is observed in 15-49 year olds who have requested an HIV test, received the test and received their results from 6.3% in 2005 to 22.8% in 2009. The tester's ability to provide correct test results is paramount. The Ministry of Health through the University Teaching Hospital Virology Unit launched the National Quality Assurance Program, to monitor and improve performance of HIV tests. Herein is presented the performance of testing sites in HIV proficiency testing (PT) rounds implemented in 2009 and 2010. **Methods:** Commercial serum panels were conditioned at the NRL to the form of dried tube specimens (DTS) and retested to ensure reactivities were maintained. The DTS panels, each consisting of five blinded samples, were distributed to participating HIV testing sites across the country along with a questionnaire. Data were analysed using EpiData 3.1, MS Excel 2007 and PASW 18.0.0. **Results:** Results returned by participants increased from 53.1% (292/550) in PT cycle 1 (PT001) to 71.8% (488/680) in PT002. In PT002, 81.2% of testers achieved 100% accuracy, versus 79.1% during PT001. Adherence to the Zambia national HIV testing algorithm improved to 78.4% during PT002 from 65.1% in PT001. Lay counselors accounted for 34.6% and 38.8% of testers involved in PT001 and PT002, while nurses accounted for 42.6% and 33.5% respectively. In both PT cycles only three-quarters of testers indicated having received training in HIV rapid testing. It was notable that the proportion of participants achieving 100% accuracy was correlated with the testing experience ( 1 year, $p < 0.05$) and with the profession of tester (lab personnel vs. others, $p < 0.01$). **Conclusions:** There has been an increase in the proportion of HIV testing sites attaining 100% scores in the PT program, with improvement also seen in adherence to the national HIV testing algorithm. Lay counselors and nurses together perform nearly three quarters of HIV tests countrywide. Less than 1 year testing experience was predictive of lower performance. There is need to increase coverage of standardized training of rapid HIV testers.

## Effective and Sustainable Surveillance Platforms

Wednesday, March 14
12:30 PM – 1:30 PM
Grand Hall

**Board 309. Outbreak Detection in Resource-limited Settings: A National Media-based Surveillance System for Public Health Events in Bangladesh**

**M. Rahman**[1], T. Ao[2], A. Alamgir[1], M.S. Haider[1], F. Haque[1], A. Chakraborty[1], J. Hossain[3], J. Sobel[2], S.P. Luby[3], E. Gurley[3];
[1]Institute of Epidemiology, Disease Control and Research, Dhaka, Bangladesh, [2]Centers for Disease Control and Prevention, Atlanta, GA, USA, [3]International Center for Diarrheal Disease Research, Bangladesh, Dhaka, Bangladesh.

**Background:** In resource limited settings, sustainable disease and outbreak surveillance may not be feasible due to high cost. In some places, media-based surveillance has been a viable supplement to

conventional surveillance systems. The Institute of Epidemiology, Disease Control and Research (IEDCR) in Bangladesh has established a surveillance system to monitor national print and television media. The objective of the system is to detect outbreaks reported by the media to facilitate rapid response. We report characteristics of this surveillance system and the results of its evaluation. **Methods:** A local media scanning company was contracted by IEDCR to scan 10 daily newspapers and 8 television stations for any health-related news item. A daily report is emailed to the National Rapid Response Team (NRRT) at IEDCR for review, verification, and, if applicable, rapid response to reported outbreaks. A database of reported events was created to capture basic metrics such as number of events reported, outbreak etiology, news source, and final outcome of investigated events. We used the CDC guidelines to conduct an evaluation using key informant interviews. We calculated cost per outbreak identified using the service fee paid to the company. **Results:** Of 1757 news items reported to IEDCR, 113 (6%) mentioned new outbreaks. A majority of the reports which mentioned a potential outbreak came from Bengali-language sources (83%); 91% came from newspapers. In the same period, IEDCR investigated 34 confirmed outbreaks, 24 (71%) of which were first reported through this media-based surveillance system. These included outbreaks of cutaneous anthrax (7), Nipah virus (1), and H5N1 (1). The cost per outbreak detected was US$3.70. Key informant interviews revealed that the system is seen as simple, flexible, timely, and universally accepted among key stakeholders. **Conclusions:** Media monitoring is a highly effective, low-cost, and sustainable outbreak detection tool in Bangladesh. It identified outbreaks of emerging infections that might not have been otherwise investigated. It can be easily integrated into existing disease-specific surveillance systems, thereby strengthening outbreak detection efforts. Similarly situated countries should consider media monitoring as part of a comprehensive public health surveillance system.

**Board 310. Laboratory-based Surveillance of Non-Typhoidal *Salmonella* Infections in Guangdong Province, China**

**X. Deng**[1], L. Ran[2], S. Wu[2], B. Ke[1], Z. Liang[3], D. He[1], X. Yang[1], Y. Zhang[1], C. Ke[1], J.D. Klena[4], X. Liu[4], M. Mikoleit[4], J.K. Varma[4];

[1]Centre of Disease Control and Prevention of Guangdong Province, China, Guangzhou, China, [2]Chinese Center for Disease Control and Prevention, Beijing, China, Beijing, China, [3]Sun Yat-Sen University, Guangzhou, China, Guangzhou, China, Centers for Disease Control and Prevention, Atlanta, GA, USA.

**Background:** Surveillance for laboratory-confirmed infections has proven to be an essential tool for monitoring incidence and detecting outbreaks of enteric pathogens that are commonly transmitted by food. China is developing a national system that will compare enteric bacterial pathogens to detect common source outbreaks. To improve understanding about disease burden and to improve detection of dispersed outbreaks, we conducted laboratory-based surveillance for non-typhoidal *Salmonella* (NTS) infection in Guangdong Province, China. **Methods:** NTS isolates cultured from patients with diarrhea were sent from clinical laboratories at 22 sentinel hospitals to local public health laboratories for confirmation, serotyping, antimicrobial susceptibility testing, and pulsed-field gel electrophoresis (PFGE). PFGE patterns were analyzed to identify clusters representing potential outbreaks. **Results:** Between September 2009 and July 2011, 15,471 stool specimens were submitted to clinical laboratories; 570 (4%) yielded NTS. *Salmonella* were most commonly detected between April and October and were isolated predominantly from children less than five years old. Sixty-five serotypes were identified, of which Typhimurium (41%) and Enteritidis (13%) were the most common. The prevalence of resistance was high for several clinically important antimicrobial agents, especially for ampicillin (63%), cefotaxime (13%), ceftazidime (8%), cefepime (10%) and ciprofloxacin (3%). More than 67% of isolates, including 92% of Typhimurium, were resistant to three or more classes of antimicrobial agents. PFGE patterns of *Salmonella* isolates from Guangdong Province were found to be diverse; however, a unique PFGE pattern comprising 70 out of 236 (30%) Typhimurium isolates was found to occur almost exclusively in

infants. **Conclusions:** In Guangdong China, serotypes Typhimurium and Enteritidis are the most common *NTS* detected in human infections, multidrug resistance is prevalent, and strains are highly diverse. Epidemiologic studies are ongoing to determine whether a common exposure is responsible for a strain of Typhimurium that is frequently isolated from infants.

**Board 311. Armed Forces Health Surveillance Center Global Respiratory Disease Surveillance Program**
**A.Y. Tsai**, K.G. Vest, J.L. Sanchez, A. Eick-Cost, D. Blazes, R. Burke;
Armed Forces Health Surveillance Center, Silver Spring, MD, USA.
**Background:** Originally established by Presidential Directive NSTC-7 in 1997, the Department of Defense (DoD) Global Emerging Infections Surveillance and Response System was expanded in 2006 and subsequently incorporated as a Division of the Armed Forces Health Surveillance Center (AFHSC) in 2008. **Methods:** The goal of the AFHSC's respiratory pathogen surveillance program is to maintain awareness and monitor respiratory disease activity worldwide which contributes to Force Health Protection and global public health. The primary objectives of the program are: 1) Provide timely, accurate, and actionable data for decision makers within the DoD and global public health community; 2) Identify genotypic/phenotypic changes in circulating influenza virus subtypes which impact disease severity, transmissibility, treatment and/or prevention control efforts across time and geography; 3) Identify zoonotic transmission and risk factors for influenza infection/illness within the human-animal interface, especially as it applies to influenza-prevalent settings; and, 4) Conduct disease surveillance in high-risk military populations (*e.g.* recruits, special warfare, shipboard personnel). These goals are accomplished through a centrally-coordinated worldwide network of partners who promote, maintain, coordinate, and improve an informative surveillance system. This network is comprised of AFHSC analysis of military health system's data and US-based laboratories which monitor respiratory disease activity among service members and families as well as by DoD overseas research laboratories which conduct surveillance among foreign military and civilian populations in partnership with the local health and defense officials of partner countries. **Results:** Recent accomplishments include the initial detection of the 2009 A/H1N1 pandemic virus, selection of one specimen (A/Iraq/8529/2009) as a World Health Organization's Northern Hemisphere 2010-2011 seasonal vaccine reference strain, and development of two new influenza assay diagnostic kits with US Food and Drug Administration approval for molecular diagnosis of human influenza in deployed or field environments. **Conclusions:** With over 70 global surveillance sites, this serves as an important network in combating emerging respiratory infections.

**Board 312. Correlations between Incidence of Pediatric Post-Diarrheal Hemolytic Uremic Syndrome and Shiga Toxin-Producing *Escherichia coli* Infection in Active and Passive Surveillance in the United States, 2000–2009**
**K.E. Heiman**[1,2], M.E. Kendall[1,2], O.L. Henao[1], R.K. Mody[1];
[1]Centers for Disease Control and Prevention, Atlanta, GA, USA, [2]Atlanta Research and Education Foundation, Decatur, GA, USA.
**Background:** Post-diarrheal hemolytic uremic syndrome (D+HUS) is most often preceded by infection with Shiga toxin-producing *Escherichia coli* (STEC) O157; rates of D+HUS should correlate with STEC infection rates, especially STEC O157. No formal evaluation of this correlation has been conducted. We evaluated reporting of D+HUS and STEC infections in two surveillance systems, passive National Notifiable Diseases Surveillance System (NNDSS) and the Foodborne Diseases Active Surveillance Network (FoodNet), to assess this correlation. **Methods:** FoodNet collects reports of culture-confirmed STEC infections, while NNDSS does not require culture-confirmation and uses a less strict D+HUS case definition. For each system, we calculated annual rates of any STEC infection, STEC O157 infection, and D+HUS rates by state for children <18 years old, using US Census data. We calculated Spearman correlation coefficients to assess the relationship between D+HUS rate and both total STEC and O157.

STEC O157 rates could not be calculated for NNDSS after 2005 because serogroup information was no longer collected. For any given year, states in which HUS was not notifiable were excluded. We compared demographic characteristics of D+HUS patients between systems. **Results:** Rates of STEC infection correlated with rates of D+HUS in FoodNet (r=0.56). This correlation was weaker in NNDSS (r=0.34). NNDSS included a larger proportion of annual state D+HUS case counts of zero than FoodNet (26% vs. 3%); when these reports are excluded, the correlation between STEC infection and D+HUS in NNDSS strengthened (r=0.55). NNDSS had more high outlier D+HUS rates, but excluding these did not improve the STEC-D+HUS correlation. Restricting to STEC O157 the correlation strengthened in FoodNet (r=0.64), but weakened in NNDSS (limited to 2000-2005, r=0.24). There were no substantial differences in demographic characteristics of patients between systems. **Conclusions:** Rates of D+HUS correlate better with STEC infection rates in FoodNet than in NNDSS. The lower correlation in NNDSS appears to be due to missed HUS cases, not an overly sensitive D+HUS case definition. Correlations in FoodNet strengthen when restricted to STEC O157; such analyses cannot be performed with current NNDSS data.

**Board 313. Recent Declines in Foodborne Disease Outbreak Reporting in the United States**

**K.M. Herman**, L.H. Gould, D. Cole;
Centers for Disease Control and Prevention, Atlanta, GA, USA.

**Background:** Foodborne disease outbreaks have been voluntarily reported by local and state health departments and analyzed by CDC since 1973. The data collection form and reporting mechanism have changed over the years, and some of these were associated with increased reporting. In 2009, the system changed to allow users to report outbreaks from multiple modes of transmission, including foodborne, waterborne, enteric person-to-person, animal contact, and environmental contamination. A decline in foodborne outbreak reporting was observed following the transition to the current surveillance system. **Methods:** Data on foodborne outbreaks that occurred in 2009 were compared with foodborne outbreaks reported during the previous five years, 2004 - 2008. Outbreak characteristics including the outbreak month of onset, etiology, implicated food, and outbreak setting were evaluated. **Results:** The number of foodborne outbreaks reported in 2009 was 40% lower than the average number reported per year during 2004 - 2008. Forty states had declines in reporting (range, 5% - 100% decrease). The greatest monthly reporting declines were for outbreaks that occurred in the last quarter of the calendar year (range, 14% - 43% decrease). Among outbreaks with a known etiology, the proportion of outbreaks caused by bacteria increased 29% and those caused by viruses decreased 16%. Reporting of bacterial toxin (those of *Clostridium perfringens*, *Bacillus cereus*, *Staphylococcus aureus*) and norovirus outbreaks were 43% and 50% lower, respectively when compared with the previous five-year average. In contrast, reporting for outbreaks associated with *Salmonella* (8% decrease) and Shiga toxin-producing *E. coli* (33% increase) remained similar or increased. The proportion of foodborne disease outbreaks with a reported a food vehicle in 2009 was 13% lower than the average reported during 2004 - 2008. **Conclusions:** A large decline in the number of foodborne disease outbreaks was reported for 2009 compared with previous years. Hypotheses for the decline in reporting include the transition to a new surveillance system, local changes in guidelines for reporting foodborne outbreaks, limited resources or changed priorities at local and state health departments, or a true decline in outbreaks caused by some pathogens.

**Board 314. Early Results and Challenges in Active Surveillance for Legionellosis**

**K.L. Dooling**, K.-A. Toews, L. Hicks, G. Langley;
CDC/NCIRD/RDB, Atlanta, GA, USA.

**Background**: Legionellosis is a nationally notifiable disease currently reported through the passive National Notifiable Diseases Surveillance System (NNDSS). Based on these data, there has been an increase in legionellosis incidence in the United States from 0.39 to 1.15 cases per 100,000 between

2000 and 2009. In January 2011, legionellosis surveillance was added to population-based Active Bacterial Core surveillance (ABCs) as a pilot program to assess incidence, disease severity and opportunities for prevention. Here we evaluate surveillance implementation and data quality during the first year. **Methods**: ABCs is an active, laboratory and population-based surveillance system covering 36 million people in 10 states. The ABCs case definition for legionellosis is lab confirmation via culture, urine antigen or seroconversion. We surveyed ABCs staff in September, 2011, to determine progress of implementation. To assess data quality we analyzed case report form data collected during June-August, 2011, from sites that had achieved full implementation, defined as receiving reports from all catchment labs. **Results**: Nine of 10 sites (90%) responded to the survey. During June-August, 2011, 64 confirmed cases were reported from 6 sites with full implementation (35 completed case report forms). ABCs staff reported that locating medical records was the most time consuming surveillance task and interpretation of chest X-ray findings was the most difficult aspect of the case report form. Based on the 35 completed case reports, data on pneumonia diagnosis were 100% complete (33 of 35 had a written diagnosis of pneumonia in the chart) and requirement for intensive care was 97% complete (12 of 34 were admitted to ICU). All 35 records indicated that cases lived in a private residence during the illness incubation period. **Conclusions:** ABCs sites have accomplished rapid (6 months) implementation of a new surveillance system for legionellosis. The data quality appears good based on completeness of important variables that measure severity and explore opportunities for prevention.

## Sexually Transmitted Diseases

Wednesday, March 14
12:30 PM – 1:30 PM
Grand Hall

**Board 315. Genital Infection by Human Papillomavirus (HPV) and *Chlamydia trachomatis* (CT) in Asymptomatic Women: Prevalence, Associated Factors and Relationship with Cervical Lesions**

**C.N. Igansi**[1,2], R.B. Barcellos[1,2], V. Kubiszewski[1], E. Cortez-Herrera[2], L.C. Millius[1,3], D.M. Wilhelms[4], A. Aguiar[4], M.L. Rossetti[1,2], M.C. Bozzetti[1];
[1]Federal University of Rio Grande do Sul, Porto Alegre, Brazil, [2]Central Laboratory of Rio Grande do Sul, Porto Alegre, Brazil, [3]Hospital de Clínicas de Porto Alegre, Porto Alegre, Brazil, [4]Grupo Hospitalar Conceição, Porto Alegre, Brazil.

**Background:** Cervical cancer affects nearly 470,000 women annually in worldwide and more than 16,000 women in Brazil. Human Papillomavirus (HPV) infection is the necessary cause of cervical cancer and its immediate precursor cervical intraepithelial. However, others factors are studied in the development of cervical cancer as *Chlamydia trachomatis* (CT) co-infection. **Methods:** A cross-sectional study was developed with HPV and CT positivity as the outcome to describe the prevalence of HPV and CT among non symptomatic women and associate these infections with studied factors, including cervical lesions. Cervical smears for cytology were collected from 1217 women and Polymerase Chain Reaction (PCR) technique was performed to identify the presence of HPV and CT. Colposcopy and biopsy were performed whenever cytology was abnormal and/or PCR was positive to HPV. HPV and CT frequencies are described and the association with the studied variables and the outcomes were estimated by means of multiple logistic regression. **Results:** The observed HPV, CT and co-infection prevalence were 28.4%, 12.6%, and 6.5%, respectively. Non-white women (Odds Ratio (OR)=1.60; 95% Confidence Interval (CI):1.10-2.38), being employed (OR=1.74; 95%CI:1.17-2.60) and having a sexual partner with history of condyloma (OR=2.35; 95%CI:1.17-4.72) presented positive association with HPV infection. The CT infection presented a positive association with women beginning sexual activity before 20 years of

age (OR=1.82; 95%CI:1.05-3.15) and women employed (OR=1.93; 95%CI:1.15-3.25). Women that reported 3 or more sexual partners during their life showed a positive association with co-infection (OR=2.02; 95%CI:1.12-3.65). Both, HPV and co-infection were associated with the presence of cytological abnormalities of the cervix (p≤0.001). **Conclusions:** An elevated HPV, CT and co-infection prevalence was observed in an asymptomatic women population. The association of these infections with socioeconomic factors, sexual behavior and with cervical lesions indicates the importance of measures in health promotion and in health prevention with this specific target as a routine activity in primary care services, and may be useful in planning such strategies, including sexual transmitted diseases controls programs and HPV vaccines.

**Board 316. The Armed Forces Health Surveillance Center's Global STI Surveillance Program**

**A.Y. Tsai**[1], R.M. Chandrasekera[1], A.A. Eick-Cost[1], B.K. Agan[2], G.E. Macalino[2], M. Mbuchi[3], K.V. Chisholm[4], S.M. Montano[5], N. Puplampu[6], K.E. Saylors[7], J.L. Sanchez[1], D. Blazes[1];
[1]Armed Forces Health Surveillance Center, Silver Spring, MD, USA, [2]Uniformed Services University of the Health Sciences, Bethesda, MD, USA, [3]US Army Medical Research Unit-Kenya, Nairobi, Kenya, [4]US Naval Medical Research Unit-3, Cairo, Egypt, [5]US Naval Medical Research Unit-6, Lima, Peru, [6]US Naval Medical Research Unit-3 Detachment, Accra, Ghana, [7]Gloval Viral Forecasting Initiative, San Francisco, CA, USA.

**Background:** In the past two years, the Armed Forces Health Surveillance Center (AFHSC) has broadened its surveillance mission regarding sexually transmitted infections (STI) worldwide. The goal of this STI surveillance network is to provide the Department of Defense (DoD) with critical information regarding the geographic distribution and trends in antibiotic-resistant *N. gonorrhoeae* (GC), define the epidemiology and behavioral risk factors for *Chlamydia trachomatis* (CT) and GC, and assess the military impact that syphilis and non-HIV viral STI pathogens may have (human papilloma virus and herpes simplex virus). **Methods:** Such efforts are achieved through collaborations which include multinational military and civilian experts. The mission of this program does not include HIV, since it is extensively studied in other DoD programs. This network's main objectives include: 1) Using a defined, standardized data collection and analytic approach to provide information for policy and decision making in the DoD and global health communities with subsequent evaluation of STI interventions; 2) Identifying circulating GC strain genotypes associated with antibiotic resistance and monitoring their temporal and spatial spread in regions of military importance; 3) Determining disease burden to the military; and, 4) Conducting surveillance of military-relevant STI viral pathogens among at-risk US and foreign military and civilian personnel, especially in deployed locations. **Results:** Surveillance is currently underway in US, Peru, Djibouti and Kenya with planned expansion in 2012 to the Republic of Georgia, Cameroon and Ghana, and potential expansion in 2013 to Afghanistan, Thailand, Korea, and the Dominican Republic. In Peru, 2-6% GC rates have been identified using urine-based Nucleic Acid Amplification Testing (NAAT) among commercial sex workers and in men who have sex with men. In Djibouti, among heterosexual men and women, higher GC rates have been identified by culture (21%) and NAAT (40%). Preliminary data from Kenya among heterosexual men and women shows intermediate GC rates by culture (13%). GC drug resistance is also being actively monitored. **Conclusions:** These data will provide public health officials with relevant information to better guide disease prevention and response strategies against STIs.

**Board 317. Quadrivalent HPV Vaccine Coverage and Series Completion among US Active Servicewomen, 2006–2011**

**H. Nsouli-Maktabi**[1,2], J.C. Gaydos[1,2], S.L. Ludwig[1], U.D. Yerubandi[1,2], A. Eick-Cost[1,2];
[1]Armed Forces Health Surveillance Center, Silver Spring, MD, USA, [2]Henry M. Jackson Foundation, Bethesada, MD, USA.

**Background:** Human papillomavirus virus (HPV) is the most common sexually transmitted infection (STI) among US military Service Members. The HPV quadrivalent vaccine (HPV4) protects women from common strains of HPV (types 6, 11, 16, 18), which are responsible for about 70% of cervical cancers and 80% of genital warts. HPV vaccine utilization in the US civilian community has been low. Consistent with the Centers for Disease Control and Prevention's Advisory Committee on Immunization Practices (ACIP) recommendations, the Department of Defense recommends routine HPV vaccination of eligible Service Members aged 17-26 years with the 3-dose series administered over 6 months (0, 2 and 6 months). Despite the availability of HPV4 vaccine, utilization by active component US Servicewomen has been anecdotally reported to be low. This investigation quantified HPV4 vaccine immunization rates in eligible US military Servicewomen. **Methods:** The Defense Medical Surveillance System was used to identify active component Servicewomen eligible for the HPV4 vaccine from 2006 to 2011. Coverage rates were described for each of the three recommended HPV4 doses. Multivariate logistic regression was used to determine predictors of non-completion of the full series. **Results:** From January 2006 to June 2011, 270,216 active component Servicewomen were in the eligible age range for the HPV4 vaccine. Of 65,552 (24.2%) women who initiated the series, 25,107 (38.3%) received a single dose, 15,410 (23.5%) received two doses, and 25,035 (38.2%) received the recommended three doses. The median numbers of months between the first and second doses, and between the first and third doses were three and eight months, respectively. Of Servicewomen who initiated the series, series completion was lowest in the Navy, younger age groups, enlisted members, married members, members with less education, and among Servicewomen with a history of STIs. An increasing trend of non-completion was observed over time. **Conclusions:** The percentages of eligible US Servicewomen who initiated and completed the HPV4 series was low, despite recommendations for vaccination and availability of the vaccine. Increased education of Servicewomen and providers on the benefits of vaccination may increase coverage and enhance series completion.

## H1N1 Influenza

Wednesday, March 14
12:30 PM – 1:30 PM
Grand Hall

**Board 318. Racial/Ethnic Disparities in Hospitalized Influenza Rates Continue after the 2009 Pandemic, Minnesota 2009–2011**

**C. Morin**, M. Ayers-Johnson, S. Swanson, C. Lexau, R. Danila, R. Lynfield;
Minnesota Department of Health, St. Paul, MN, USA.

**Background:** During the 2009 pandemic, elevated rates of 2009 pandemic H1N1 (pH1N1) influenza morbidity and mortality were noted among indigenous populations in several U.S. states and other countries. In Minnesota, hospitalized case rates were significantly higher in non-whites (including Hispanics) compared to white, non-Hispanics (NHs). In order to assess whether these racial/ethnic disparities were unique to the pandemic, we compared persons hospitalized with influenza during the pandemic period to those hospitalized during the 2010-2011 influenza season. **Methods**: Statewide, population-based surveillance for influenza hospitalizations began October 2008. A pandemic case was defined as a person hospitalized with lab-confirmed pH1N1 influenza from April 2009-April 2010, and a 2010-2011 season case was defined as a person hospitalized with lab-confirmed pH1N1 or seasonal A H3 (H3) influenza from October 2010-April 2010. Race/ethnicity was obtained from disease reports, medical records, state vaccine registry, or case interviews. Provisional 2010 U.S. Census data were used to calculate rates. **Results:** A total of 2,300 cases were identified; 1,818 during the pandemic and 482

during the 2010-2011 season. Of these, 1,947 were pH1N1 and 353 were H3. A total of 1,737 (95%) pandemic period and 468 (97%) 2010-2011 season cases had a known race/ethnicity. Overall, incidence was highest among black, NHs (60.8 per 100,000), followed by Native American, NHs (42.8), Hispanics (35.3), Asian, NHs (23.7), and white, NHs (17.0). Non-whites accounted for 31% (n=524) of cases during the pandemic period compared to 23% (n=107) during the 2010-11 season. After adjustment for age, the relative rates associated with non-white vs. white, NH race was 2.46 during the pandemic period (95% CI, 2.21-2.72, p<0.001) and 2.54 during the 2010-11 season (95% CI, 2.02-3.20, p<0.001).
**Conclusions:** In Minnesota non-white persons were significantly more likely to have been hospitalized with influenza compared to white, NHs during the pandemic. This disparity was not unique to the pandemic and was again noted during the 2010-2011 influenza season. Evaluation of socioeconomic factors, vaccine coverage, chronic illnesses, and access to care is needed to develop effective public health interventions to alleviate this disparity.

**Board 319. Influenza as a Cause of Reported Severe Viral Pneumonia Cases in Vietnam, 2006–2011**

**N.T. Thu Yen, Sr.**;
National Institute of Hygiene and Epidemiology, Ha Noi, Viet Nam.

**Background:** In 2005, Vietnam had the highest numbers worldwide of human cases and deaths from avian influenza A/H5N1. Concern for respiratory viruses such as influenza A/H5N1 and other pathogens with pandemic potential led the Ministry of Health to institute a national passive surveillance system for severe viral pneumonia (SVP). This hospital-based surveillance has been in place since 2006. **Methods:** We use the following case definition used for SVP: sudden onset of fever >38°C (measured or reported), difficulty breathing, chest radiograph consistent with viral pneumonia, and no other diagnosis. For patients meeting inclusion criteria, we collected epidemiological and outcome data, as well as throat swabs which were tested for influenza using RT-PCR with WHO standardized procedures. **Results:** From January 2, 2006 to September 7, 2011, 981 SVP cases were reported, 596 male (61%), with a median age of 37 years (IQR 23-53 years), with a range of 1 month to 96 years. Influenza viruses were identified in 134 (14%). Pandemic A/H1N1/2009 (pH1N1) virus was detected in 61 patients (6.2%), A/H5N1 in 25 (2.6%), A/H3N2 in 20 (2.0%), influenza B in 19 (1.9%), and seasonal A/H1N1 in 9 (0.9%). SVP surveillance detected 25 of Vietnam's 26 reported cases of human influenza A/H5N1 since the system's initiation in 2006. Among SVP cases, the case fatality rate for patients without influenza was 26%, for patients with A/H5N1 was 68%, and for patients with other influenza viruses was 29%. For SVP case-patients with influenza, median time from onset date to admission date into hospital was 5 days (IQR 2-7 days) for fatal cases, and 2 days (IQR 1-4 days) for non-fatal cases (p<0.001 by Wilcoxon rank-sum test).
**Conclusions**: While SVP surveillance met its primary goal of detecting influenza A/H5N1 cases, most of the influenza cases detected were pandemic A/H1N1 and seasonal strains, which had a surprisingly high case fatality rate. These influenza deaths were significantly associated with delays in hospital admission. Additional testing is planned on the samples to determine the other etiologies of SVP cases. For the future of SVP surveillance, increased testing with a more standardized and sensitive case definition is being considered.

**Board 320. Genetic Diversity of HA1 Domain of Hemagglutinin Gene of Pandemic Influenza A/H1N1 2009 Viruses in Northern India**

**M.A. Mir**[1], P. Koul[2], W.M. Sullender[3], Y. Singh[1], A. Krishnan[1], R.B. Lal[4], S. Broor[1];
[1]All India Institute of Medical Sciences, New Delhi, India, [2]Sher-i-Kashmir Institute of Medical Sciences, Srinagar, India, [3]University of Alabama, Birmingham, AL, USA, [4]Centers for Disease Control and Prevention, Atlanta, GA, USA.

**Background:** Genetic analysis of HA1 domain of HA gene of 2009A/H1N1 (pH1N1) virus was done to understand virus evolution during 2009-2011 in two north parts of India having distinct patterns of

circulation: New Delhi with peak of influenza circulation during (July-Aug), and Srinagar during (Jan-Feb). **Methods:** Cases with influenza like illness were tested for influenza by rRT-PCR and isolates were generated using MDCK cells. HA1 (n=57) from randomly selected pH1N1 pandemic viruses isolated in Delhi (n=39) and Srinagar (n=18) during 2009 to 2011 were sequenced. Phylogenetic analyses were done using neighbor-joining and evolutionary distances calculated using the Maximum Composite Likelihood methods in MEGA4. Nucleotide alignment file was created and amino acid changes analyzed in BioEditversion7.0.9.0, using representative global strains from GISAID (n=151; including 72 from 2009, 88 from 2010 and 66 from 2011). **Results:** Phylogenetic analysis of 57 HA1 genes from 2009 to 2011 from north India clustered with clade 7 viruses characterized by S203T mutations. None of the viruses from 2009 to 2011 formed a monophyletic group, suggesting a continuum of independent introduction of circulating viral strains. Amino acid analysis revealed minor amino acid changes in the antigenic or receptor binding domains. RBS revealed 220-loop to be highly conserved (3-8% changes at D222N), whereas 130-loop revealed emergence of A134T change in 2010 and 2011 viruses (6-19%). The antigenic sites analysis revealed maximum changes in the 190 helix (residues 184-191) structure, where S183P was observed in 6-19% of viruses. More importantly, S185T (present in 1918 viruses) is evolving worldwide, was observed in 38% and 63% viruses from 2010 and 2011, respectively. Likewise, AA change at D97N is emerging with mutations in 12-32% and 44-67% viruses in 2010 and 2011, respectively. No changes were observed in highly conserved residues at Tyr (91,150,180, and 192) which form the base of the pocket. Likewise, the three asparagine-linked glycosylation sites at positions Asn (23, 87, 276) (2 isolates from Srinagar had N276S) remain highly conserved through 2009 to 2011. **Conclusions:** Collectively, emergence of S185T should be closely monitored; especially if antibody mediated pressure is imposing such changes.

**Board 321. Detection of 2009 Pandemic Influenza A (H1N1) RNA in Lung Pneumocytes and Lymphoid Tissues of Fatal Influenza Cases by a Novel In-Situ Hybridization Assay**

**J. Bhatnagar**, T. Jones, D.M. Blau, W.-J. Shieh, C.D. Paddock, C. Drew, A.M. Denison, D. Rollin, S.R. Zaki;
Infectious Diseases Pathology Branch, CDC, Atlanta, GA, USA.

**Background:** The sudden emergence of 2009 pandemic influenza A/H1N1 virus (pH1N1) and quick worldwide spread has highlighted the need to study the virus tropism, pathology and the pathogenesis. A few animal studies have provided insights into disease pathogenesis and a limited number of reports have described pathological findings in fatal human cases by immunohistochemistry (IHC). Although IHC can reveal viral antigens in specific cells, it does not indicate that the cells support viral replication. In-situ hybridization (ISH) allows for localization of viral and messenger RNA in infected cells and can provide insight into viral replication sites. **Methods:** Formalin-fixed, paraffin embedded (FFPE) autopsy tissues from 21 patients with RT-PCR-confirmed pH1N1 infection (illness onset in the United States during May 2009-March 2010) were evaluated by a novel ISH assay for pH1N1and IHC. For ISH, sense (detects viral RNA) and antisense (detects mRNA and cRNA) DIG-labeled RNA probes targeting the NP gene of pH1N1 were generated by in vitro transcription using the RNA Labeling Kit (Roche). ISH was performed on the Tecan system. IHC was performed by using a monoclonal antibody against the nucleoprotein of the influenza A virus. **Results:** pH1N1 RNA or antigens were detected in 81% of cases (57% positive by ISH; 76% positive by IHC). In cases with short duration of illness (1-3 days), 69% were ISH positive whereas 62% were IHC positive. Both ISH and IHC staining were observed predominantly in the nuclei and cytoplasm of infected epithelial cells in airways, mucous glands and in alveolar epithelial cells. Intra-alveolar pneumocytes showed strong hybridization signals with the antisense probe. Interestingly, in 38% of hilar and paratracheal lymphoid tissues tested staining was observed only by ISH. All the lymphoid tissues tested were IHC negative. **Conclusions:** Detection of pH1N1 RNA in alveolar pneumocytes and lymphoid tissues suggest pathological similarity of pH1N1 with H5N1 virus. Localization of mRNA in alveolar pneumocytes, as indicated by strong ISH signals with antisense probe,

suggests viral replication in these cells. The novel ISH assay could be a useful adjunct to localize pH1N1 in tissues, particularly for short duration cases and to further investigate viral replication and tissue tropism of the virus.

**Board 322. How Many Influenza Viruses Were Circulating in 1918?**

**G.D. Shanks**[1], A. MacKenzie[2], M. Waller[2], J.F. Brundage[3];
[1]Army Malaria Institute, Enoggera, Australia, [2]Center for Military and Veterans Health, University of Queensland, Brisbane, Australia, [3]Armed Forces Health Surveillance Center, Silver Spring, MD.

**Background:** Mortality rates varied widely during the 1918-19 influenza pandemic even in apparently identical military units. It has been hypothesized that the mortality differences were due to variable exposures to distinct influenza viruses either before or during 1918. Others have suggested that the early (spring-summer 1918) and late (fall-winter 1918-9) pandemic waves were not due to a single influenza A/H1N1 strain that suddenly increased in lethality in mid 1918, but different influenza strains with different clinical expressions. It remains unclear whether two or more influenza A viruses were co-circulating in 1918. **Methods:** Prospectively collected medical records of individual soldiers of the Australian Imperial Force (AIF) fighting in Europe during the First World War were examined to assess morbidity and mortality likely due to influenza. **Findings:** Acute respiratory illnesses thought to be due to influenza during the "first pandemic wave" (Apr-Jul 1918) did not protect affected soldiers against hospitalization for influenza-like illnesses, but did protect them against pneumonia/influenza mortality OR 0.37 (95% CI 0.25-0.53), during the "second and third waves" (Oct 1918-Mar 1919). Respiratory illness-related hospitalization rates in selected infantry battalions during the second wave did not significantly vary based on respiratory illness experience during the first wave: adjusted hazard ratios, 49 Infantry Battalion, 0.90 (0.61-1.34), 50 Infantry Battalion of 1.29 (0.87-1.91), nurses of 0.69 (0.37-1.31) and engineers of 1.05 (0.66-1.66). Cox proportional hazards analyses revealed that subjects with the longest military service had the lowest risk of hospitalization for a respiratory illness during Oct 1918-Mar 1919. Soldiers hospitalized with acute respiratory illnesses (including "purulent bronchitis") during the winter of 1916-17 were not protected from influenza-related hospitalization or death in late 1918. **Interpretation:** The most parsimonious interpretation of these findings is that two or more influenza A/H1N1 strains were co-circulating in humans during 1918. We conclude that the low lethality mid 1918 wave of influenza like illness was not caused by the same virus which caused the subsequent high mortality wave in late 1918.

## Surveillance of Vaccine-Preventable Diseases

Wednesday, March 14
12:30 PM – 1:30 PM
Grand Hall

**Board 323. Evaluation of the Acute Flaccid Paralysis Surveillance System in Kaduna State, January 2005 – December 2010**

**A.M. Oladimeji**[1], S.O. Gidado[1], E. Arenyeka[2], F. Zakari[2], P. Nguku[1], O. Biya[1], O. Fawole[3], M. Jacob[4], M.A. Zakari[4], A. Abubakar[1];
[1]Nigeria Field Epidemiology and Laboratory Training Programme, Abuja, Nigeria, [2]World Health Organization, Kaduna State Office, Kaduna, Nigeria, [3]Dept. of Epidemiology, Medical Statistics and Environmental Health, University of Ibadan., Ibadan, Nigeria, [4]Kaduna State Ministry of Health, Kaduna, Nigeria.

**Background**: Following the World Health Assembly (WHA) resolution in 1988 to eradicate poliomyelitis worldwide, Acute Flaccid Paralysis (AFP) surveillance system was established in 1997 in Kaduna State, northern Nigeria as one of the four strategies recommended for polio eradication. The objectives of the system are to detect AFP cases, identify areas of Wild Polio Virus (WPV) transmission and guide targeted immunization activities. However, since July 2009 till date, the system has documented absence of WPV. We evaluated the system to describe its process of operation, assess key attributes and determine whether or not the system was meeting set objectives. **Methods**: Evaluation was done using the 2001 CDC's Updated Guidelines for Evaluating Public Health Surveillance Systems. Three key informants from the State Ministry of Health and partner agencies were interviewed. A focus group discussion with the Local Government Disease Surveillance and Notification Officers was conducted. Finally, the state's AFP surveillance data from January 2005 to December 2010 was reviewed and analysed. **Results**: Active case search was used in identifying cases from AFP reporting sites and communities; employing a simple case definition. Information flowed from these sites through clearly outlined reporting channels to the three tiers of the health system and partners utilizing user-friendly reporting forms. Of the 1235 AFP cases detected between 2005 and 2010, 206 (16.6%) were laboratory confirmed poliomyelitis. Isolated WPV cases decreased with increased immunization coverage. The system's sensitivity measured by annualized non-polio AFP rate was 6.5 and 5.6 in 2005 and 2010 respectively (target >=2/100,000). The mean annual stool adequacy and timeliness were 90.2% and 88% (target >=80% for each). Positive predictive value PPV was 34.6%, 21.7% and 0% in 2005, 2008 and 2010 respectively. Partner agencies provided 90-95% of the resources. **Conclusions**: System appears simple, sensitive and meeting set objectives. However, it has a low PPV and majorly donor-driven. System requires sustained efforts in active surveillance and increased funding from the government.

**Board 324. Invasive Pneumococcal Disease in Children 2–59 Months of Age in Three San Francisco Bay Area Counties, Active Bacterial Core Surveillance Project, California Emerging Infections Program, January 1995 – June 2011**

**E.P. Garcia**, P.D. Kirley, M. Apostol;
California Emerging Infections Program, Oakland, CA, USA.

**Background:** *Streptococcus pneumoniae* (SPN) is a major cause of bacteremia, meningitis and pneumonia in children in the United States. Each year, over 4800 cases of invasive pneumococcal disease (IPD) are identified in children <5 years. The 7-valent pneumococcal conjugate vaccine (PCV7) introduced in 2000 and 13-valent pneumococcal conjugate vaccine (PCV13) introduced in 2010 offer protection against common IPD-causing strains. Here, we describe IPD incidence trends and examine demographic and clinical characteristics of IPD in the California Active Bacterial Core Surveillance (ABCs) catchment area of Alameda, Contra Costa and San Francisco counties from 1995-2011. **Methods:** An IPD case was defined as a 2-59 month old resident of the ABCs catchment area at the time of positive SPN isolate recovery from a normally sterile site. Cases were identified via ABCs active, laboratory-based surveillance. Isolates were obtained when available and submitted to the Centers for Disease Control for serotype testing. **Results:** A total of 628 IPD cases were identified from 1/1995 through 6/2011. Of the 387 sent for serotyping, 367 were successfully serotyped. IPD incidence was 50, 46, 12 and 15 cases per 100,000 in 1995, 2000, 2005 and 2010, respectively. Bacteremia was the most common type of infection (86%), followed by bacteremic pneumonia (22%). Median age was 19 months; the majority of cases were <2 years (62%) and male (60%). Of the 628 cases, 26% had at least 1 underlying medical condition. Hospitalizations occurred in 44% of cases with a median stay of 5 days. The case fatality proportion was 1%. Pre PCV7 introduction, common serotypes included 6B (15%) and 14 (40%), serotypes included in PCV7. Post PCV7 introduction, the most common serotype was 19A (40% in 2005), a non-PCV7 serotype now found in PCV13. **Conclusions:** IPD incidence in the catchment area varied during the surveillance period, but has decreased over time, and the rate is approaching the Healthy People 2020 goal of 12

cases per 100,000. Consistent with previously published reports, IPD infections were most common among children <2 years. Despite the availability of pneumococcal conjugate vaccines, children <5 years are still at risk for IPD. Efforts must be made to encourage full vaccine coverage and continued disease surveillance is warranted to monitor disease trends.

**Board 325. Predictors of Rotavirus Vaccination in the National Immunization Survey 2009**

**K.C. Allen**, V.K. Cheruvu;
Kent State University, Kent, OH, USA.

**Background:** In the US, 3 million cases of Rotavirus infection occurs annually sending 1 in 7 children under 5 to the ER for gastroenteritis. Though the Rotavirus vaccine has reduced the incidence of ER visits and hospitalizations by 94% and 96%, respectively, it is important to identify the factors influencing vaccination to further reduce transmission rates. The CDC recommends continued surveillance to determine the effects of Rotavirus vaccine on transmission. The objective of this study is to identify those factors that influence Rotavirus vaccination. **Methods:** To understand the predictors for receiving the Rotavirus vaccine nationally, the National Immunization Survey 2009 data were analyzed. Logistic regression analysis was performed to model the probability of receiving the vaccine. **Results:** Children with health insurance (p=.0054), with a hospital as their health care provider's facility (p=.0283), and whose parents have higher education (p=.0127) were more likely to have received the vaccine. **Conclusions:** Health insurance is associated with income which impacts the type of insurance and health care provider. Receipt of childhood vaccinations is significantly dependent on health insurance and parent's education attainment. This study points to the importance of developing educational strategies for parents among low educated, low income populations for improved access to health care.

**Board 326. Early Impact of 13-Valent Pneumococcal Conjugate Vaccine on Invasive Pneumococcal Disease, U.S., 2010–2011**

**C.M. Cox**[1], R. Link-Gelles[1], M.M. Farley[2], W. Schaffner[3], A. Thomas[4], A. Reingold[5], L.H. Harrison[6], C. Lexau[7], S. Zansky[8], S. Petit[9], K. Gershman[10], K. Scherzinger[11], B.W. Beall[1], C.G. Whitney[1];
[1]Centers for Disease Control and Prevention, Atlanta, GA, USA, [2]Emory University, Atlanta, GA, USA, [3]Vanderbilt University, Nashville, TN, USA, [4]Oregon Public Health Division, Portland, OR, USA, [5]University of California, Berkeley, Berkeley, CA, USA, [6]Johns Hopkins Bloomberg School of Public Health, Baltimore, MD, USA, [7]Minnesota Department of Health, St. Paul, MN, USA, [8]New York State Department of Health, Albany, OK, USA, [9]Connecticut Department of Public Health, Hartford, CT, USA, [10]Colorado Department of Health and Environment, Denver, CO, USA, [11]New Mexico Department of Health, Santa Fe, NM, USA.

**Background**: The U.S. introduction of 13-valent pneumococcal conjugate vaccine (PCV13) began in March 2010. To evaluate potential early effects of PCV13 introduction, we compared rates of invasive pneumococcal disease (IPD) after introduction of PCV13 to rates of IPD before vaccine introduction. **Methods**: IPD cases (isolation of pneumococcus from sterile sites) were identified through 10 Active Bacterial Core surveillance (ABCs) sites. We compared quarterly rates of IPD (cases per 100,000 population) in 2010 and the first quarter of 2011 with baseline rates from 2006-08. Isolates were serotyped to identify those included in PCV13; all cases were categorized as PCV13 serotypes or non-PCV13 serotypes, with sub-analyses performed for serotypes 19A, 7F, and 3. To account for multiple comparisons, we considered P<0.0025 as significant. **Results**: During 2010 and the first quarter of 2011, ABCs identified 4,964 cases of IPD of which 4,309 (87%) had serotyping results. During 2006-2008, 12,182 IPD cases were identified of which 10,707 (88%) had serotyping results. Among children <2 years old, rates of IPD due to PCV13 serotypes were significantly lower in the fourth quarter of 2010 (9.4 per 100,000) and first quarter of 2011 (7.2 per 100,000) compared with baseline rates during the fourth quarter and first quarter of 2006-08 (24.1 and 27.2 per 100,000, respectively). There was a statistically significant reduction in the rate of IPD due to serotype 7F (86%) in the fourth quarter of 2010 and in the

rate of IPD due to serotype 19A (75%) in the first quarter of 2011. Overall IPD rates were significantly lower in the fourth quarter of 2010 (18.8 per 100,000) and first quarter of 2011 (13.9 per 100,000) compared with baseline rates during the fourth quarter and first quarter of 2006-08 (40.3 and 43.4 per 100,000, respectively). We did not observe statistically significant reductions in IPD caused by serotype 3, although the number of cases was small (0-6 cases per quarter). **Conclusions**: We found significantly lower overall and PCV13-specific IPD rates during the fourth quarter of 2010 and first quarter of 2011. Reductions in IPD appear to be driven by decreases in disease due to serotypes 19A and 7F, both of which are contained in PCV13. These preliminary findings are consistent with early impact of PCV13 on IPD among young children.

## Foodborne and Waterborne Infections

Wednesday, March 14
12:30 PM – 1:30 PM
Grand Hall

**Board 327. Restaurant Characteristics Associated with Improper Food Cooling Practices**

**L.G. Brown**[1], B. Le[1], D. Ripley[2], EHS-Net Working Group;
[1]Centers for Disease Control and Prevention, Atlanta, GA, USA, [2]Metro Nashville Public Health Department, Nashville, TN, USA.

**Background:** Improper cooling of hot food is a significant source of foodborne illness and should be the focus of intervention efforts. As half of all foodborne illness outbreaks are associated with restaurants, it is particularly important to focus on restaurant cooling practices. However, little is known about restaurant cooling practices or restaurant characteristics that promote or hinder proper cooling. Thus, the purpose of this study was to collect descriptive data on restaurant cooling practices and identify restaurant characteristics associated with improper food cooling practices, as identified by the FDA. This study was conducted by the Environmental Health Specialists Network (EHS-Net), a network of federal, state, and local environmental health specialists focused on understanding the environmental causes of foodborne illness. **Methods:** EHS-Net environmental health specialists collected data in 420 randomly selected restaurants through interviews of restaurant managers and observations of food cooling practices. The interviews assessed restaurant characteristics (e.g., restaurant ownership) and the observations assessed food cooling practices (e.g., cooling food depth). **Results:** In 39% of 470 observations in which food was cooled through refrigeration, food was cooled at improper depths (> 3 inches). In 16% of these observations, food was cooled in cooling units with improper ambient temperatures (> 41 degrees F), in 14% of these observations, cooling food containers were improperly stacked on top of each other, in 34% of these observations, cooling food was ventilated improperly, and in 24% of these observations, inadequate air space was provided around the cooling food containers. The odds of improper cooling practices were greater in independent restaurants (vs. chains), restaurants that served < 200 meals daily (vs. $\geq$ 200 meals daily), and restaurants with a lack of manager and worker food safety training. **Conclusions:** This study found that improper cooling practices were not uncommon. Restaurant food cooling practices were related to restaurant type, volume (i.e., number of meals served daily), and food safety training. These data suggest that smaller, independent restaurants may be at higher risk for improper cooling, and that food safety training is important in safe food cooling.

**Board 328. Does Geographic Residence Play a Role in *Salmonella enteric*a serotype Javiana Infections? Data From FoodNet, 2005–2010**

**J. Huang**[1,2], M.E. Patrick[2], C. Jung[3], R. Meyer[4], O. Henao[2];
[1]Oak Ridge Institute for Science and Education, Oak Ridge, TN, USA, [2]Centers for Disease Control and Prevention, Atlanta, GA, USA, [3]Maryland Department of Health and Mental Hygiene, Baltimore, MD, USA, [4]Georgia Department of Public Health, Atlanta, GA, USA.

**Background:** Javiana is the fourth most common *Salmonella* serotype identified by the Foodborne Diseases Active Surveillance Network (FoodNet). Incidence has increased steadily since 2004 and rates are highest in the southeastern U.S. Because environmental and economic structures of counties vary between and within states, we examined degree of urbanization to further explain the geographic variation of *Salmonella* Javiana. **Methods:** FoodNet conducts population-based active surveillance for culture-confirmed *Salmonella* Javiana infections in ten sites. We examined demographic and geographic data and calculated incidence by dividing the number of laboratory-confirmed infections by the US Census population for the FoodNet catchment area, 2005-2010. We grouped sites into 'northern' or 'southern' using latitude 41°N and categorized counties as metropolitan (metro) or nonmetropolitan (non-metro), and as coastal or non-coastal based on criteria developed by the US Department of Agriculture's Economic Research Service and National Oceanic and Atmospheric Administration. Coastal counties are defined as counties where at least 15% of total land area is within a coastal watershed. **Results:** From 2005-2010, FoodNet identified 2,809 serotype Javiana infections (1 per 100,000 population). The incidence in southern states (1.5) was more than 10 times higher than in northern states (0.1). In southern states, the incidence in non-metro counties (3.2) was higher than in metro counties (1.2). The highest incidence in southern non-metro counties was in infants (44), persons aged 1-4 years (16), blacks (3.9), and persons living in coastal counties (4.7). Non-metro cases were more likely than metro cases to live in coastal counties (48% vs. 29%; $p<0.01$). **Conclusions:** In FoodNet, the incidence of serotype Javiana infection was highest in southern, non-metro counties, with rates highest among children, blacks, and persons living in coastal counties. This suggests that environmental factors such as temperate climate, proximity to water, and socioeconomic differences may play a role. Future studies to identify specific environmental risk factors for persons in non-metro counties are needed to create targeted prevention programs to reduce *Salmonella* Javiana infections.

**Board 329. Disease Burden of Foodborne Pathogens in the Netherlands, 2009**

**A.H. Havelaar**[1,2], J.A. Haagsma[1,3], M.-J.M. Mangen[1,4], J.M. Kemmeren[1], L.P. Verhoef[1], S.M. Vijgen[1,5], M. Wilson[6], I.H. Friesema[1], L.M. Kortbeek[1], Y.T. Van Duynhoven[1], W. Van Pelt[1];
[1]RIVM, Bilthoven, Netherlands, [2]Utrecht University, Utrecht, Netherlands, [3]Erasmus University, Rotterdam, Netherlands, [4]University Medical Center, Utrecht, Netherlands, [5]Health Care Insurance Board, Diemen, Netherlands, [6]RSI, Ottawa, ON, Canada.

**Background**: Making choices about the allocation of resources and prioritizing interventions to address health issues requires a consistent, quantitative assessment of the relative public health importance of diseases. Currently, statistics on the public health impact of foodborne diseases focus on the incidence of non-fatal illness and of fatal cases. Burden of disease metrics, such as the Disability Adjusted Life Year (DALY) integrate incidence data with indices of severity and duration. **Methods**: Fourteen pathogens that can also be transmitted by food are included in the study (four infectious bacteria, three toxin-producing bacteria, four viruses and three protozoa). The incidence of community-acquired non-consulting cases, patients consulting their general practitioner, those admitted to hospital, as well as the incidence of sequelae and fatal cases is estimated using surveillance data, cohort studies and published data. Disease burden includes estimates of duration and disability weights for non-fatal cases and loss of statistical life expectancy for fatal cases. Data are combined with results from an expert survey to assess the fraction of cases attributable to food, and the main food groups contributing to transmission.

**Results**: Among 1.8 million cases of disease (approx. 10,600 per 100,000) and 233 deaths (1.4 per 100,000) by these fourteen pathogens, approximately one-third (680,000 cases (4,100 per 100,000) and 78 deaths (0.5 per 100,000) are attributable to foodborne transmission. The total burden is 13,500 DALY (82 DALY per 100,000). On population level, *Toxoplasma gondii*, thermophilic *Campylobacter* spp., rotaviruses, noroviruses and *Salmonella* spp. cause the highest disease burden. The burden per case is highest for perinatal listeriosis and congenital toxoplasmosis. Approximately 45% of the total burden is attributed to food. **Conclusions**: The ranking of foodborne pathogens based on burden is very different compared to when only incidence is considered. The burden of acute disease is a relatively small part of the total burden. In the Netherlands, the burden of foodborne pathogens is similar to the burden of upper respiratory and urinary tract infections.

**Board 330. *Aeromonas* and Acute Diarrhea in Nepal**

**L. Bodhidatta**[1], S.K. Shrestha[2], J.R. Dhakwa[3], L.B. Thapa[4], B. Shrestha[5], B. Rayamajhi[2], A. Srijan[1], C.J. Mason[1];
[1]Armed Forces Research Institute of Medical Sciences, Bangkok, Thailand, [2]Walter Reed /AFRIMS Research Unit-Nepal (WARUN), Kathmandu, Nepal, [3]Kanti Children Hospital, Kathmandu, Nepal, [4]Sukraraj Tropical Infectious Disease Hospital, Kathmandu, Nepal, [5]Bharatpur Hospital, Bharatpur, Nepal.

**Background:** Four major serotypes of Aeromonas spp. are reported to be human pathogens, however, its etiological role in causing gastroenteritis in human is still controversial. **Methods:** A diarrhea etiology study in adults and children with diarrhea and non-diarrhea controls was conducted for 2 years in 3 hospitals in Nepal. Stool samples were tested for diarrhea etiologic agents by culture, ELISA and PCR. **Results:** A total of 1800 diarrhea and 1800 non-diarrhea stool samples were tested. Aeromonas was detected in 12% and 6% of cases versus 3% and 2% of controls in 2 pediatric populations aged 3 months to 5 years ($p<0.001$) and was strongly associated with diarrhea in the children aged 24-60 months (20% of cases versus 3% of controls, ($p<0.001$)). Aeromonas was also found in 9% and 4% of adults with and without diarrhea (p=<0.001), respectively. Of the 189 Aeromonas isolates from cases, 79 (42%), 57 (30%) and 53 (28%) were *A.caviae*, *A.hydrophila* and *A.verronii*, respectively. Of the 61 Aeromonas isolates from controls, 22 (36%), 27 (44%) and 12 (20%) were *A.caviae*, *A.hydrophila* and *A.verronii*, respectively. Despite small numbers, *A.verronii* was strongly associated with diarrhea at all three sites, *A.caviae* was associated with diarrhea in children, and *A.hydrophila* was associated with diarrhea in adults. **Conclusions:** Our findings suggest that Aeromonas is a significant cause of acute diarrhea in both children and adults in Nepal.

**Board 331. Molecular and Descriptive Epidemiology of Sporadic Cases of Cryptosporidiosis, Minnesota, 2000–2010**

**T.J. Robinson**, E.A. Cebelinski, K.E. Smith;
Minnesota Department of Health, St. Paul, MN, USA.

**Background:** Cryptosporidiosis is an acute diarrheal illness caused by the protozoan *Cryptosporidium*. Because molecular speciation/subtyping of *Cryptosporidium* is complex and resource intensive, it is not routinely performed by most US laboratories. In 1999, the Minnesota Department of Health (MDH) began developing molecular subtyping for *Cryptosporidium* to enhance outbreak detection and strengthen understanding of the pathogen. We examined the molecular and descriptive epidemiology of sporadic cryptosporidiosis cases in Minnesota from 2000-2010. **Methods:** Cryptosporidiosis is nationally notifiable and since 2006, clinical laboratories in Minnesota have been required to submit clinical materials from positive specimens to MDH. Genotyping was performed by PCR followed by restriction fragment length polymorphism analysis of either the dihydrofolate reductase gene or the small-subunit rRNA gene. Sequence analysis of the GP60 gene was performed on all *C. hominis* and *C. parvum* specimens. We compared *C. hominis* and *C. parvum* cases. **Results:** Of 2,371 sporadic cryptosporidiosis

cases reported, specimens from 774 (33%) were identified to species, including 562 (73%) *C. parvum*, 186 (24%) *C. hominis* and 26 (3%) other species/genotypes. Thirty-one *C. parvum* subtypes and 30 *C. hominis* subtypes were identified; 77% of the *C. parvum* cases had one of two subtypes, but the two most common *C. hominis* subtypes accounted for only 39% of the *C. hominis* cases. *C. parvum* cases were more likely to be hospitalized (p=0.01), have drunk raw milk (p=0.01), have drunk well water (p<0.001), or have had contact with cattle (p<0.001) in the 14 days prior to illness onset. *C. hominis* cases were more likely to be non-white (p<0.001), have an immunocompromising condition (p<0.001), have had contact with children in a daycare setting (p=0.002), have swum in a pool (p=0.01) or have travelled (p=0.004) in the 14 days prior to onset. **Conclusions:** We found significant clinical and epidemiological differences between *C. parvum* and *C. hominis* cases. While this study represents one piece of the molecular and epidemiologic picture of cryptosporidiosis in the US, it further emphasizes the value of state laboratories performing molecular characterization of *Cryptosporidium* specimens.

**Board 332. Review of Surveillance Data Sources for Food Source Attribution for *Salmonella enterica* serotype Enteritidis**

**A.R. Vieira**[1,2], S.J. Chai[1], B. Karp[1], W. Gu[1], N.J. Golden[3], P.L. White[4], J.S. Zablotsky-Kufel[3], K.G. Holt[5], K. Klontz[6], D.J. Cole[1];
[1]Centers for Disease Control and Prevention, Atlanta, GA, USA, [2]Oak Ridge Institute for Science and Education, Oak Ridge, TN, USA, [3]US Department of Agriculture, Food Safety and Inspection Service, Washington, DC, USA, [4]US Department of Agriculture, Food Safety and Inspection Service, Omaha, NE, USA, [5]US Department of Agriculture, Food Safety and Inspection Service, Atlanta, GA, USA, [6]US Food and Drug Administration, College Park, MD, USA.

**Background:** Food source attribution uses detailed pathogen information to estimate the number of human illnesses caused by specific foods. Federal agencies need reliable attribution estimates to inform regulations and to track progress toward goals. In particular, risk managers require estimates of the number of *Salmonella* serotype Enteritidis (SE) infections due to specific foods to evaluate regulatory changes made by the US Food and Drug Administration (FDA) and the US Food Safety and Inspection Service (FSIS). Data availability is a major factor that drives the selection of methods for estimating attribution, and a review of surveillance data sources for SE can provide valuable information for attribution studies. **Methods:** We examined data ranging from 1996 to 2009 from US sources, including data from: 1) FoodNet, the Foodborne Diseases Active Surveillance Network, a Centers for Disease Control and Prevention (CDC)-led project that provides annual incidence of SE infections, outbreak and international travel-related proportions, and case-control study findings, 2) the CDC Foodborne Disease Outbreak Surveillance System (FDOSS) which includes implicated foods, and 3) the FSIS which maintains data on the presence of SE in meat, poultry, and egg products at slaughter and processing. We performed a concordant review of available data to inform SE food source attribution. **Results:** FoodNet estimates that approximately 7% of SE infections are outbreak-related and 19% are travel-related. Implicated food commodity data in FDOSS show eggs to be the main food source of SE (64%), followed by poultry (18%). Two FoodNet case-control studies associated the highest estimated attributable fractions for SE infections with consumption of eggs and chicken. Similarly, SE is among the top five serotypes isolated from FSIS-regulated ground and intact chicken and pasteurized egg products. **Conclusions:** This study focusing on SE highlights how a concordant overview of different sources of surveillance data, with varying structures and uncertainties, can be used to inform food source attribution for a specific pathogen. The results suggest that future studies should focus on developing analytic methods that combine information from multiple sources to provide estimates of attribution to food sources.

**Board 333. Evaluation of the Role of Shiga Toxin 2 Subtypes in Virulence of Non-O157 Shiga Toxin-Producing *Escherichia coli* Strains in Minnesota**

**K. Smith**, E. Hedican, E. Cebelinski, V. Lappi, C. Medus, B. Koziol, C. Taylor;
Minnesota Department of Health, St. Paul, MN, USA.

**Background:** Shiga toxin-producing *E. coli* (STEC) are an important cause of hemorrhagic colitis and hemolytic uremic syndrome (HUS). As the use of non-culture diagnostics for STEC increases, it is becoming recognized that non-O157 STEC cause illness at least as commonly as does *E. coli* O157 (O157). As a group, non-O157 STEC are not as virulent as O157, but certain non-O157 STEC strains do cause HUS and death. Shiga toxin 2 (Stx2) is considered a necessary virulence factor for the most severe forms of disease, but differences in STEC virulence cannot be explained simply by the presence of Stx2. We explored whether certain subtypes of the *stx2* gene are associated with non-O157 STEC virulence. **Methods:** Two groups of clinical STEC isolates were evaluated: 1) 198 isolates (92 O157, 106 non-O157) from previously described sentinel site populations (Group 1); and 2) all non-O157 STEC isolates associated with HUS cases in Minnesota (Group 2, n=7). *Stx1* and/or *stx2* genes were detected by multiplex PCR. If *stx2* was detected, the gene was subtyped by partial sequence analysis of the $stxAB_2$ operon. Subtypes were designated *stx2*a-*stx2*d. **Results:** In Group 1, *stx2* was detected in 92 (100%) O157 isolates and 39 (37%) non-O157 isolates. Subtypes of *stx2* among O157 isolates included *stx2*a only (79%), *stx2*c only (12%), and *stx2*a + *stx2*c (9%). Subtypes of *stx2* among non-O157 isolates included *stx2*a only (54%), *stx2*b only (15%), *stx2*d only (13%), *stx2*c only (5%), *stx2*a + *stx2*c (3%), *stx2*a + *stx2*d (3%), and new variants (5%). Among Group 1 isolates with at least *stx2*a, O157 cases were slightly more likely than non-O157 cases to have bloody diarrhea (85% vs. 71%), be hospitalized (37% vs. 22%), and develop HUS (9% vs. 0%), but none of the differences were statistically significant. Among just Group 1 non-O157 isolates with *stx2*, cases with subtype *stx2*a were more likely than cases with other *stx2* subtypes to have bloody diarrhea (71% vs. 31%, p=0.03) and be hospitalized (22% vs. 0%, p=0.1). All 7 Group 2 (HUS associated) non-O157 isolates had only the *stx2*a subtype of *stx2*. **Discussion:** Non-O157 STEC isolates from sentinel populations had a higher diversity of *stx2* subtypes than O157 isolates. Subtype *stx2*a might be important in the increased virulence of some non-O157 STEC strains. However, a larger population of isolates should be tested to assess this association.

## Role of Health Communication

Wednesday, March 14
12:30 PM – 1:30 PM
Grand Hall

**Board 334. It's Not What You Say, It's How You Say It: Communicating Nipah Virus Prevention Messages during an Outbreak in Bangladesh**

**S. Parveen**[1], M. Islam[1], M. Begum[1], M.-U. Alam[1], H.M. Sazzad[1], E.S. Gurley[1], R. Sultana[1], M. Rahman[2], S.P. Luby[1,3], M. Hossain[1];
[1]International Centre for Diarrheal Diseases Research, Bangladesh (ICDDR,B), Dhaka, Bangladesh, [2]Institute of Epidemiology Disease Control and Research (IEDCR), Dhaka, Bangladesh, [3]Centers for Disease Control and Prevention, Atlanta, GA, USA.

**Background:** In Bangladesh, Nipah virus (NiV) is highly fatal (>75%) and is transmitted from bats to humans by drinking raw date palm sap. During a NiV outbreak in 2010, the local health authority used microphones and household visits to tell residents that drinking sap could cause the illness and announced that people should not drink sap. Despite these prevention efforts, we learned during the course of the investigation that the community believed that the outbreak was caused by supernatural

forces, not sap consumption, and they continued drinking the sap. We attempted to communicate the same prevention messages using interactive communication and a more meaningful causal explanation. **Methods:** We invited people to attend community meetings. In the meetings we showed photos of bats drinking raw sap from harvested date palm trees and explained to them in lay language that bats carry a virus called 'Nipah' and that they could contaminate the sap through their saliva and urine. We told them to avoid drinking raw sap because it could be contaminated with the virus. We also replied to participants' queries during the sessions. **Results:** Approximately 700 people participated in four community meetings. Participants mentioned that earlier messages did not include information about bats being the source of this virus in the sap and therefore did not convince them that drinking sap could cause the disease. Participants stated that from the community meetings they understood how the virus was transmitted in the sap and this was reinforced by their experiences of seeing bats in and around the sap collection pot. Participants responded positively to the biomedical model of disease transmission we presented with their supernatural explanation by saying that they now understood that the supernatural force causing the outbreak was called a 'virus'. They reported that they would abstain from raw sap consumption. **Conclusions:** During NiV outbreaks, one-way communication asking people to change behaviors without a meaningful causal explanation of transmission is unlikely to be effective. An interactive communication strategy in lay language with supporting evidence can make biomedical prevention messages credible to an affected community, even those who may initially invoke supernatural causal explanations.

**Board 335. Process of Neonatal Tetanus Elimination in Pakistan: Progress and Challenges**

**J.A. Lambo**, T. Nagulesapillai;
University of Calgary, Calgary, AB, Canada.

**Background:** Pakistan is one of the thirty nine countries that have not achieved maternal and neonatal tetanus elimination. Neonatal tetanus (NT), caused by *Clostridium tetani,* is a highly fatal infection of the neonatal period. NT is one of the most underreported diseases and still remains a major but preventable cause of neonatal and infant mortality in many developing countries. Since the World Health Assembly called for the elimination of NT in 1989, considerable progress has been made using the following strategies: clean delivery practices, routine tetanus toxoid (TT) immunization of pregnant women and immunization of all women of childbearing age with three doses of TT vaccine in high-risk areas during supplementary immunization campaigns. **Methods:** A systematic review was undertaken to assess the process of neonatal tetanus elimination in Pakistan. Medline, PubMed, Embase, and Cochrane databases were searched, as were reference lists of relevant publications. Inclusion criteria were randomized trials and observational studies on neonate population and neonatal tetanus in Pakistan. **Results:** Twenty-five publications were found relating to neonatal tetanus in Pakistan, excluding case series, case reports and reviews. TT vaccination coverage in Pakistan ranges from 40.4% to 65%. Low vaccination coverage, the main driver for neonatal tetanus in Pakistan, is due to many factors including demand failure for TT vaccine, resulting from inadequate knowledge of TT vaccine among reproductive age females and inadequate information about the benefits of TT provided by health care workers and media. Other factors linked with low vaccination coverage include residing in rural areas, lack of formal education, poor knowledge about place and time to get vaccinated and lack of awareness about the importance of vaccination. A disparity exists in TT vaccination coverage and antenatal care between urban and rural areas due to access and utilization of health care services. **Conclusions:** To successfully eliminate neonatal tetanus, women of reproductive age must be made aware of the benefits of TT vaccine not only to themselves, but also to their families. Effective communication strategies for TT vaccine delivery and health education focusing on increasing awareness of NT are strongly suggested.

**Board 336. Evaluation of Dengue Prevention Education in Key West, Florida**

**E.C. Zielinski-Gutierrez**[1], C. Blackmore[2], R. Blanton[3], M.S. Doyle[4], R. Eadie[5], E. Gazdick[6], A. Leal[7], J. Matthias[2], K. Pattison[8], C. Perez-Guerra[9], D. Stanek[2], C. Tittel[5], D. Tisch[6], J. Vyas[6], T. Warner[6], M. Vanderbrook[5];
[1]Centers for Disease Control and Prevention, Fort Collins, CO, USA, [2]Florida Dept of Health, Tallahassee, FL, USA, [3]Case Western Reserv e University, Cleveland, OH, USA, [4]Florida Keys Mosquito Control District, Kay West, FL, USA, [5]FL Dept Health/Monroe County, Key West, FL, USA, [6]Case Western Reserve University, Cleveland, OH, USA, [7]Florida Keys Mosquito Control District, Key West, FL, USA, [8]CDC & Colorado State University, Fort Collins, CO, USA, [9]CDC, San Juan, PR, USA.

**Background:** Key West, FL experienced autochthonous transmission of the viral mosquito-borne disease, dengue, in 2009 and 2010, with nearly 100 cases reported. This launched large-scale, multi-agency efforts to engage residents in prevention activities. **Methods:** A population-based evaluation of knowledge, attitudes and behaviors toward dengue prevention was undertaken in July 2011, consisting of 557 household interviews and property assessments to determine the presence or absence of immature *Aedes aegypti* mosquito infestations. Households were selected using a randomized cluster sampling approach that covered the entire island of Key West. **Results:** Domestic Inspectors from the Florida Keys Mosquito Control District (64.8%) and local newspaper articles (64.6%) were identified as the most frequently recalled sources of dengue information. Most respondents (83.5%) were able to identify standing water as a source for the mosquito vector and infected mosquitoes as the transmission mode for dengue (87.3%). Respondents who reported exposure to dengue educational messages scored statistically significantly higher on a scale of dengue knowledge than respondents who did not recall exposure to educational messages. For example, 42-51% of respondents who were exposed to mosquito inspectors, newspaper articles or door hangers had good or excellent knowledge compared to only 18-25% good/excellent knowledge among those who had not been exposed to these sources of information ($p<0.001$). Challenges remain however: Less than half of respondents (43%) were aware that dengue cases in Key West had been hospitalized, though 59% considered dengue a serious problem for Key West. One-third of respondents were not able to name specific symptoms of dengue illness. **Conclusions:** Results from the study will be used to quantify the impact of the communication strategies employed in Key West. Lessons learned regarding effective communication strategies for the future in Key West and other areas of the US that experience local dengue transmission will be presented.

**Board 337. Knowledge and Risk Factors of Congenital Infection among Pregnant Chilean Women**

**M.A. Rolfes**[1], F. Guerra[2], P. Fry[3], R. Tamayo[3], C.A. Munoz-Zanzi[1,3];
[1]University of Minnesota School of Public Health, Minneapolis, MN, USA, [2]Institute of Obstetrics and Gynecology, Austral University of Chile and Valdivia Regional Hospital, Valdivia, Chile, [3]Institute of Preventive Veterinary Medicine, Austral University of Chile, Valdivia, Chile.

**Background:** Congenital infections (CI) are infections transmitted from mother to fetus during pregnancy. Ample evidence of CI sequelae in offspring exists (e.g. cytomegalovirus, *T. gondii*, HIV, rubella, and influenza), even if infections are not apparent at birth. The public health significance of developmental disabilities due to CI and of appropriate prevention programs are particularly important for developing countries where prevalence of infectious diseases is higher and resources for diagnosis and treatments are often limited. Similarly, intervention programs, including education about CI, do not commonly exist. The objective of this study was i) to determine the level of awareness about CI in general, and toxoplasmosis and cytomegalovirus (CMV) in particular, among pregnant women in Los Rios region, Chile, and ii) to examine associations between CI knowledge and risk behaviors. **Methods:** Pregnant women were enrolled in the study at the time of hospitalization for delivery at either a large public hospital (Valdivia Region Hospital) or private clinic in the Los Rios Region of Chile. These settings account for 70% of the 5,000 annual deliveries for the entire Los Rios Region. An extensive questionnaire

was administered including information on socio-demographic characteristics, risk behaviors, and knowledge about CI. **Results:** Of the 991 women responding, 56.7% knew about CI and 43.5% provided an adequate example. The most common example was HIV/AIDS, though 4 women mentioned toxoplasmosis. Women who knew about CI were significantly more likely to work outside the home, have private health insurance, and were more educated than women who did not know about CI. Sixty-four women (6.4%) knew about toxoplasmosis. These women also had higher socio-economic status and education than women who were unaware. Only 4 women had heard about CMV. Despite differences in education and economic situation, very few differences existed in risk behaviors for *Toxoplasma* exposure between women who knew about infection and those who did not; including avoiding raw meat consumption and washing hands after handling raw meat or cat litter boxes. **Conclusions**: The behavior of women suggests that they intuitively know about toxoplasmosis and disease exposure even though explicit knowledge of CI was low in this population.

## Emerging Opportunistic Infections

Wednesday, March 14
12:30 PM – 1:30 PM
Grand Hall

**Board 338. Histoplasmosis as a Re-emerging Infection: Results from a Cohort of AIDS Patients in Medellín, Colombia**

**D.H. Caceres**[1,2], A.M. Tobon[1], C.M. Scheel[3], A.M. Ahlquist[3], D.Y. Berbesi[2], A. Restrepo[1], T. Chiller[3], B.L. Gomez[1,4];
[1]Medical and Experimental Mycology Unit, Corporacion para Investigaciones Biologicas, Medellin, Colombia, [2]Facultad de Medicina, Universidad CES, Medellín-Colombia, Medellin, Colombia, [3]Centers for Disease Control and Prevention, Atlanta, GA, USA, [4]School of Medicine and Health Sciency, Universidad del Rosario, Bogota, Colombia.

**Background:** Disease caused by Histoplasma capsulatum adds significant morbidity and increases mortality in HIV-infected individuals, particularly for those living in developing countries without full access to appropriate diagnostic methods or antiretroviral therapy. In Colombia certain areas are recognized as highly endemic for histoplasmosis, but little information exists on the true prevalence in HIV infected individuals. **Methods:** A prospective study in a cohort of patients consulting the Hospital La Maria in Medellín, Colombia, was conducted from May 2008 - August 2011. Inclusion criteria required that persons were HIV-infected and had three out of five of the following: fever, pancytopenia, weight loss, radiological evidence of pulmonary involvement or skin/mucosal lesions compatible with histoplasmosis. Cases were defined in two groups: proven cases were patients with positive H. capsulatum culture; probable cases that had histopathological evidence or a reactive serological test. **Results:** Eighty-nine patients met the inclusion criteria. Forty-five patients (50.6%) met the case definition (30 proven and 15 probable). Median age was 35.3 years (range, 21-55). There were 6 (13.6%) deaths among the 45 case-patients. A total of 22 (49%) case-patients had other microbial co-infections, the most common being tuberculosis (n=16, 35.6%). At time of diagnosis, the median CD4 cell count was 30 (range 3-570). **Conclusions:** This study highlights the importance of histoplasmosis as an opportunistic infection in HIV-infected persons in Colombia. Histoplasmosis was common in this cohort, with a third of patients having concurrent TB, which can complicate diagnosis in resource limiting settings. More work is needed to better define the burden of this disease and to provide guidance for better diagnosis and more rapid treatment.

**Board 339. Outbreak of *Francisella novicida* Infections among Inmates at a Correctional Facility, Louisiana—April–July, 2011**

**M. Brett**[1], S. Yendell[1], R. Ratard[2], J. Hand[2], G. Balsamo[2], C. Scott-Waldron[2], J. Carpenter[3], J. Petersen[1], B. Yockey[1], L. Respicio[1], P. Singh[4], C. O'Neal[5], V. Hill[3], P. Mead[1];
[1]Centers for Disease Control and Prevention, Fort Collins, CO, USA, [2]Louisiana Office of Public Health, New Orleans, LA, USA, [3]Centers for Disease Control and Prevention, Atlanta, GA, USA, [4]Correctional Facility A, Baton Rouge, LA, USA, [5]Louisiana State University Medical Center, Baton Rouge, LA, USA.

**Background:** *Francisella novicida* is rarely identified as a cause of human illness, with only 7 cases described in the literature. During April-July 2011, three cases of *F. novicida* bacteremia were reported among inmates at a correctional facility in Louisiana. One inmate died acutely. **Methods:** To verify the outbreak, ascertain additional cases, and identify risk factors for infection, we reviewed laboratory records, clinic visit logs, medical records, housing logs, food sources, and interviewed the surviving patients. With the assistance of facility staff, we inspected the potable water system and reviewed the production and distribution of ice within the facility. Samples of water and swabs from ice machines were collected for analysis by culture and by PCR using three targets. **Results:** Between March and July 2011, the hospital laboratory completed 1,981 blood cultures; 326 or 6.9% were from the prison. The probability that contamination would only affect samples from the prison was approximately 3 in 10,000. No further cases were identified with review of charts and logs. The three patients were male, aged 40-56 years, and African American. All were immunocompromised; two had severe liver disease and the other was taking immunosuppressive medications. Although all had bacteremia, no unifying clinical symptoms were indentified. The three patients were housed in different locations, ate at different kitchens and had no contact with each other. Common exposures included the water supply, ice, and visits to medical buildings. Although all inmates were seen in the onsite medical facilities, we found no common procedure, medication or staff exposure. Water quality at the prison was adequate when tested but may have been compromised in early 2011 due to low turnover in a water storage tank. Ice was centrally produced and distributed by inmates, with multiple opportunities for contamination. Swabs from one set of ice machines yielded evidence of *Francisella novicida* DNA by PCR. **Conclusions:** We report the first known cluster of human *F. novicida* infections. Although the ultimate cause of contamination could not be identified, ice appears to be the likely vehicle of transmission. Providers who care for immunocompromised patients should be aware of the potential for bacteremia with this unusual organism.

## Host and Microbial Genetics

Wednesday, March 14
12:30 PM – 1:30 PM
Grand Hall

**Board 340. Severe Outcomes Are Associated with Genogroup 2 Genotype 4 Norovirus Outbreaks: A Systematic Literature Review**

**R. Desai**[1], C. Hembree[2], A. Handel[2], J.E. Matthews[3], B.W. Dickey[4], S. McDonald[1], A. Hall[1], U.D. Parashar[1], J.S. Leon[3], B. Lopman[1];
[1]CDC, Atlanta, GA, USA, [2]University of Georgia, Athens, GA, USA, [3]Emory University, Atlanta, GA, USA, [4]Medical College of Wisconsin, Milwaukee, WI, USA.

**Background:** Noroviruses are the most common cause of epidemic gastroenteritis, and a major cause of foodborne illness. Currently, the relative impact of individual factors underlying severe illness are poorly understood and difficult to tease apart across a small number of outbreaks. This report reviews

published norovirus outbreak reports to quantify hospitalization and mortality rates and assess their relationship with outbreak setting, transmission route, and strain. **Methods:** 2435 non-duplicate articles were identified in PubMed, EMBASE, and Web of Knowledge using a string of terms related to "norovirus" and "outbreak". Inclusion criteria included articles with a minimum of two ill persons with a common exposure and at least one RT-PCR-confirmed case of norovirus disease. Pooled estimates of the proportion of cases that were hospitalized and died were estimated by outbreak strain, setting, and route of transmission. Univariate analyses were performed by outbreak setting, transmission route, and outbreak strain, and multivariable models were fitted to estimate the independent effect of each factor. **Results:** 843 norovirus outbreaks reported in 233 published articles from 45 countries between January 1993 and June 2011 were analyzed. Based upon a total of 71,724 illnesses, 506 hospitalizations, and 45 deaths, pooled hospitalization and mortality rates were 0.70% (95% confidence interval [CI]: 0.64% - 0.77%) and 0.066% (95% CI: 0.048% - 0.087%), respectively. In multivariate analysis, higher hospitalizations rates were associated with healthcare settings (incidence rate ratio [IRR]: 3.4, 95% CI: 1.9 - 6.2, $p < 0.001$) and GII.4 norovirus strains (IRR: 6.7, 95% CI: 3.2 - 14.0, $p < 0.001$), and higher mortality rates were associated with healthcare settings (IRR: 50, 95% CI: 9 - 279, $p < 0.001$). **Conclusions:** Our review demonstrates that hospitalizations and deaths were much more likely in healthcare associated outbreaks, independent of other factors. Surprisingly, GII.4 viruses were independently associated with higher levels of hospitalization, though we could not assess their association with mortality due to limited numbers of deaths with strain data. Our analysis underscores the importance of developing vaccines against GII.4 viruses to prevent severe disease outcomes, including death.

**Board 341. Large-Scale Candidate Gene Analysis of Infections by Hepatitis A and E Viruses and Cytomegalovirus in the US General Population: Analysis Using the Third National Health and Nutrition Examination Survey (NHANES III), 1991–1994**

**L. Zhang**[1], M.-H. Chang[2], A. Yesupriya[3], C.-G. Teo[1];
[1]Division of Viral Hepatitis, National Center for HIV/AIDS, Viral Hepatitis, STD, and TB Prevention, CDC, Atlanta, GA, USA, [2]Epidemiology and Analysis Program Office, Office of Surveillance, Epidemiology, and Laboratory Services, CDC, Atlanta, GA, USA, [3]Research Data Center, National Center for Health Statistics, CDC, Atlanta, GA, USA.

**Background:** Hepatitis A virus (HAV), hepatitis E virus (HEV), and cytomegalovirus (CMV) are prevalent in the US. Common to infection by these 3 viruses is a positive age correlation with seroprevalence. To identify human genetic variants that confer risk of infection by HAV, HEV and CMV, and using data from 6779 participants of Phase 2 of NHANES III (conducted from 1991 to1994), we evaluated phenotype-genotype associations between IgG seropositivity to HAV, HEV or CMV, or to combinations of these viruses and 396 single-nucleotide polymorphisms in 190 candidate genes. **Methods:** Specimens were analyzed from 2619 non-Hispanic White (NHW), 2095 non-Hispanic Black (NHB), and 2065 Mexican American participants. Univariate and multivariable logistic regression analyses were used to test for associations within each of the three race/ethnicity groups. **Results:** Significant associations were found primarily among Mexican Americans: *ASCL1/C12orf42* (rs10778213) and *LDLR* (rs1433099) with decreased risk of seropositivity to HAV and seropositivity to HAV and HEV combined (odds ratios [ORs] = 0.65 to 0.8, p-values adjusted for false discovery rates [FDR-Ps] = 0.01 to 0.001); *LDLR* variant rs2738450 with increased risk of CMV seropositivity (OR = 1.52, FDR-P = 0.003); and *G6PC2* rs560887 and *LDLR* rs1433099 with decreased risks of seropositivity to HAV, HEV and CMV combined (OR = 0.67, FDR-P $< 1 \times 10^{-7}$; and OR = 0.66, FDR-P = 0.001; respectively). Based on significance ($p < 0.05$) and consistent direction of effect, the association of *ASCL1/C12orf42* with HAV seropositivity was replicated in the NHB group (combined p = 0.004). No significant associations were observed among NHWs. Variants in several genes were associated (uncorrected $p < 0.05$) with seropositivity to HEV in at least two race/ethnicity

groups, but none remained significant when corrected for FDR. **Conclusions:** Susceptibility to infections by HAV, HEV and CMV appears to be associated with genetic variants and these associations may vary among race/ethnicity. Replication studies involving larger population samples are warranted.

## Respiratory Infections in Special Populations

Wednesday, March 14
12:30 PM – 1:30 PM
Grand Hall

### Board 342. Impact of New Influenza Vaccine Requirement for Children Enrolled in Day Care—Connecticut, 2011

**K. Yousey-Hindes**[1], J. Hadler[1], K. Soto[2], K. Kudish[2], J. Meek[1], R. Heimer[1];
[1]Yale School of Public Health, New Haven, CT, USA, [2]Connecticut Department of Public Health, Hartford, CT, USA.

**Background:** National estimates indicate that up to 60% of preschool aged children receive non-parental care each week, mostly in childcare centers. An influenza vaccination requirement for this group may have population level effects. Effective January 1, 2011, all children aged 6-59 months enrolled in a licensed Connecticut day care were required to show proof of influenza vaccination. We sought to determine the impact of this requirement on influenza and influenza-like illness incidence during the first influenza season since its implementation. **Methods:** We used national and state survey data to measure changes in influenza vaccination coverage in children 6 months - 4 years old. We analyzed influenza and influenza-like illness data from three sources: the Connecticut Hospital Emergency Department Syndromic Surveillance system, the Connecticut Emerging Infections Program's Influenza-Associated Hospitalization Surveillance system, and influenza reports to the state reportable disease database. We compared the percentage of visits, hospitalizations or reports in preschool aged children during October 2010 - April 2011 to those in the most recent H3N2 dominant influenza season, 2007-08. **Results:** Compared to the season prior to the vaccine requirement, influenza vaccination rates among those 6 months - 4 years increased dramatically (53.9% vs. 85%, $p<0.01$). Compared to 2008, among preschool aged children, the proportion of all emergency department visits for fever/flu decreased in 2011 (33.5% vs. 30.4%, p: 0.01). When compared to the 2007-08 season, among those hospitalized with influenza, the proportion of patients 6 months - 4 years also decreased (4.5% vs. 2.3%, p: 0.04). Finally, compared to 2007-08, the proportion of lab-confirmed influenza infections among those 6 months - 4 years decreased as well (13.2% vs. 10.3%, p: 0.03). **Conclusions:** This ecological analysis identified temporal correlations between the institution of a new influenza vaccination requirement for day care attendance and both an increase in vaccination rates among children 6 months - 4 years and a decrease in influenza illness. Continued surveillance is needed to determine if the observed decrease in influenza continues in future influenza seasons, including those in which H1N1 is the dominant circulating strain.

### Board 343. Seasonal Influenza Vaccine Effectiveness in Refugee Children in Kenya, 2010–2011: A Retrospective, Case-Control Study Using Test-Negative Controls

**M.A. Katz**[1], R. Nyoka[1], J.M. Wong[1], J. Ndirangu[2], P. Muthoka[3], E. Lebo[1], M. Nguhi[4], J. Ahmed[1], J.W. Burton[5], J. Mott[1], L. Waiboci[1], R.F. Breiman[1], R. Eidex[1];
[1]Centers for Disease Control-Kenya, Nairobi, Kenya, [2]International Rescue Committee, Kakuma Refugee Camp, Kenya, [3]Ministry of Public Health and Sanitation, Nairobi, Kenya, [4]International Rescue Committee, Nairobi, Kenya, [5]UN High Commission for Refugees, Nairobi, Kenya.

**Background:** Recent surveillance has shown that influenza is a significant cause of respiratory disease in tropical countries in Africa. However, little is known about the effectiveness of seasonal influenza immunization in Africa, including among vulnerable populations like refugees. **Methods:** Influenza surveillance has been ongoing in Kakuma Refugee Camp since 2007. Nasopharyngeal and oropharyngeal specimens are collected from inpatients with severe acute respiratory illness (SARI) and outpatients with influenza-like illness (ILI) and tested for influenza by RT-PCR. During October 2010 - November 2010, we offered two doses of free (donated by Sanofi Pasteur) injectable trivalent 2010 southern hemisphere seasonal influenza vaccine to 5,250 children, 6-24 months old and 36-59 months old residing in the Camp. Patients were asked about vaccination status as part of the routine surveillance questionnaire. We conducted a case-control study to evaluate the effectiveness of the vaccine in preventing laboratory-confirmed influenza associated with ILI and SARI among children 9 months - 59months old using surveillance data from from Kakuma from December 2010-August 2011. We used generalized linear models with a Bayesian approach to compare the effectiveness of the vaccine stratified by influenza type. We calculated the risk ratios between vaccinated and unvaccinated based on posterior summaries. **Results:** During the evaluation period, specimens from 47/454 (10%) children with respiratory illness (ILI or SARI) were positive for influenza; 27 were influenza A, and 21 were influenza B. Of all children with respiratory illness, 161 (35.5%) were vaccinated. Vaccine effectiveness against laboratory-confirmed influenza associated with respiratory illness was 37.5% [95% Credibility Interval (CI) 27.3,51.5] for any influenza, 59.5% (95% CI 37.9,93.5) for influenza A, and 9.4% (95% CI 5.9,15.0) for influenza B. **Conclusions:** A trivalent seasonal influenza vaccine was effective in reducing influenza-associated respiratory illness among refugee children in Kenya. In settings where influenza vaccine is not routinely used or available, such as refugee settings, targeted influenza vaccine mass campaigns may be effective for reducing the burden of influenza.

**Board 344. Influenza Viruses a Leading Cause of Respiratory Infection among Adults in Two Refugee Camps in Kenya**

**J.A. Ahmed**[1], M.A. Katz[1], M. Nguhi[2], A.K. Tepo[3], I. Ndege[3], R. Nyoka[1], D. Odhiambo[3], L.W. Waiboci[1], M. Abdel Kader[2], W. Burton[4], D. Mutonga[5], R.F. Breiman[1], R.B. Eidex[1];
[1]Centers for Disease Control and Prevention - Kenya, Nairobi, Kenya, [2]International Rescue Committee, Nairobi, Kenya, [3]Kenya Medical Research Institute, Nairobi, Kenya, [4]United Nations High Commissioner for Refugees, Nairobi, Kenya, [5]Kenya Ministry of Public Health and Sanitation, Nairobi, Kenya.
**Background:** Viruses likely cause a substantial proportion of respiratory infections, which are the leading cause of morbidity in refugee camps. However, specific viral etiologies of respiratory illness among adults in these settings are not well characterized. We examined data obtained from adults presenting with influenza-like illness (ILI) or severe acute respiratory infection (SARI) in two refugee camps in Kenya. **Methods:** From September 2007 to August 2011, nasopharyngeal and oropharyngeal specimens were obtained from persons ≥15 years old with symptom onset of ≤14 days and meeting case classifications for ILI and SARI. ILI was defined as fever ≥38°C and cough or sore throat, and SARI as ILI and shortness of breath or difficulty breathing requiring hospitalization. We report on the prevalence of viral infection in this population. Specimens were tested at KEMRI-CDC laboratories by RT-PCR for adenovirus (AdV), respiratory syncytial virus (RSV), human metapneumovirus (hMPV), parainfluenza viruses (PIV) and influenza A and B. **Results:** During September 2007-to-August 2011, 318 adults (female, 178 [56.0%]; median age=25, range =15-84 years)) presenting with either ILI (148 [46.5%]) or SARI (170 [53.5%]) at health facilities in Dadaab and Kakuma refugee camps in Kenya were tested. Duration of illness before seeking health care was 0 to 14 days (median=2). Viruses were detected in the following frequencies: influenza A 53(16.7%), AdV 22(6.9%), influenza B 16(5.0%), RSV 11(3.5%), PIV 10(3.1%), and hMPV 9(2.8%). The proportion positive for influenza A (18.3%), AdV (8.5%) and RSV (6.1%) was highest among 15 to 19 years old, however this trend was not statistically significant. Among the 99 (31.1%)

patients with a virus detected, >1 virus was detected in 29(29.3%). Specimens from 21/53 Influenza A positive specimens were subtyped. While 17/21 (81%) were influenza A H1N1 pandemic strain, 4 (19.0%) were identified as H3N2. There was strong variation year-to-year in proportion positive for AdV (p=0.005), RSV (p=0.004) and PIV (p=0.02).Viral etiology was not associated with either ILI or SARI. **Conclusions:** Influenza viruses, AdV and RSV were found to be the leading respiratory viruses associated with infection among adults in the refugee camps.

## Zoonotic and Animal Diseases

Wednesday, March 14
12:30 PM – 1:30 PM
Grand Hall

**Board 345. Detection of Japanese Encephalitis, Chaoyang, and Getah Viruses in Culicine Mosquitoes (Diptera: Culicidae), Republic of Korea, 2008–2010**

**T.A. Klein**[1], H.-C. Kim[2], R. Takhampunya[3], B.P. Evans[4], P. Masuoka[5], J. Grieco[5], J.S. Lee[6], J.-Y. Yeh[7], D.-K. Lee[8], W.-J. Lee[9];
[1]65th Medical Brigade, APO, AE, USA, [2]5th Medical Detachment, APO, AE, USA, [3]Armed Forces Research Institute of Medical Sciences, APO, AE, USA, [4]Armed Forces Reseach Institute of Medical Sciences, APO, AE, USA, [5]Uniformed Services University of Health Sciences, Bethesda, MD, USA, [6]US Army Medical Research Institute of Infectious Diseases, Fort Detrick, MD, USA, [7]National Veterinary Research and Quarantine Service, Anyang, Korea, Republic of, [8]Kosin University, Busan, Korea, Republic of, [9]Korea Centers for Disease Control and Prevention, Gheongwon-gun, Korea, Republic of.

**Background:** Japanese encephalitis virus (JEV) is endemic in the Republic of Korea (ROK) and a serious health threat to unvaccinated Korean and US populations. In 2010, an outbreak of 26 cases was >12 times the annual average for the last 26 years. Getah virus is of veterinary importance and has infrequently been documented from the ROK, while Chaoyang virus has not been previously reported. **Methods:** A total of 150,805 culicine female mosquitoes collected from 2008-2010 were identified to species, placed in pools of up to 30 mosquitoes by date and site of collection, and screened for flaviviruses and alphaviruses by real-time polymerase chain reaction assay. Virus positive pools were assayed for JEV, Chaoyang, and Getah viruses using species specific primers. **Results:** A total of 98 and 18 pools of 6,845 were positive for JEV and Chaoyang viruses, respectively. Three species were positive for JEV *Culex tritaeniorhynchus* (92), *Cx. bitaeniorhynchus* (2) and *Cx. pipiens* (4), with minimum field infection rates (MFIR) of 1.67, 1.02 and 0.36 respectively. The NS5 region was sequenced and both Genotype I and V (first report in the ROK) were observed. Three species were positive for Chaoyang virus, *Aedes vexans* (16), *Cx. pipiens* (1) and *Ae. albopictus* (1), with MFIR of 0.21, 2.75, and 0.09 respectively. This is the first report of Chaoyang virus in the ROK. Two of 106 pools of 2,091 *Ae. vexans* were positive for Getah virus (Alphaviridae), with a MFIR of 0.96. Partial regions of the *nsp1* gene were sequenced and characterized. **Conclusions:** While a JEV mandatory childhood vaccine program was fully implemented in 1971, JEV remains a serious health threat for the civilian population >30 years of age and those with waning immunity. It is also a concern for non-immune (unvaccinated) US soldiers, civilians, and family members deployed to the ROK. Therefore, the JEV vaccination policy for US personnel deployed to the ROK must be reexamined. There currently is no evidence of Chaoyang virus infections resulting in either human or animal disease. However, Getah virus is a serious threat to horses. Mosquito-borne virus surveillance should be continued to determine their relative human and veterinary importance, geographical distribution, and ecological impact on vector populations and disease transmission.

**Board 346. US National Surveillance for Influenza Viruses among Marine Mammals**

**T. Waltzek**;
University of Florida, Gainesville, FL, USA.

**Background:** Since the late 1970s, influenza A and B viruses have been detected by viral isolation, serologic methods, and rtPCR in wild populations of cetaceans and pinnipeds. In 1979-1980, approximately 20% of the northeastern US harbor seal (*Phoca vitulina*) population died from an H7N7 outbreak that likely arose following transmission from an avian host. This influenza A virus replicated to high titers in mammalian (e.g. ferrets, cats, pigs) but not avian hosts. Experimental transmission studies with this virus produced a lethal systemic disease in squirrel monkeys, and several investigators exposed to the affected harbor seals developed severe conjunctivitis. Despite growing evidence of influenza A and B viruses within wild marine mammal populations, surveillance for respiratory viruses resulting in marine mammal morbidity and mortality at institutions harboring managed populations of pinnipeds and cetaceans is lacking. **Methods:** We employed previously validated PCR assays to design a 7-pathogen surveillance panel to include: orthomyxoviruses (Influenza A and B viruses), paramyxoviruses, coronaviruses, reoviruses, anelloviruses, herpesviruses, and adenoviruses. **Results:** To date, we have tested approximately 100 marine mammal respiratory samples and have discovered an anellovirus and an orthoreovirus infecting bottlenose dolphin (*Tursiops truncatus)*. **Conclusions:** Here we outline the first concerted surveillance effort to detect and characterize respiratory viruses in marine mammals in zoos, aquariums, and stranded US marine mammal populations. Preliminary marine mammal testing has revealed novel DNA (anellovirus) and RNA (orthoreovirus) viruses. Given the limited respiratory samples tested to date, continued surveillance is planned to determine the prevalence of significant human pathogens, such as influenza viruses, circulating in marine mammal populations.

**Board 347. Toxoplasmosis in Ghana: A Seroprevalence Study in the Cape Coast Municipality**

**G. Kwakye-Nuako**[1], B. Morawski[2], P. Osei-Owusu[3], D. Amoako-Sakyi[1], D. Obiri-Yeboah[1], P. Ayeh-Kumi[4], C. Munoz-Zanzi[2];
[1]University of Cape Coast, Cape Coast, Ghana, [2]University of Minnesota, Minneapolis, MN, USA, [3]University of North Dakota, Grand Forks, ND, USA, [4]University of Ghana Medical School, Accra, Ghana.

**Background:** *Toxoplasma gondii*, is one of the most common parasitic infections worldwide. Felines are the definite hosts and a wide range of animals (birds and mammals) and humans can serve as intermediate hosts. Previous studies in parts of Africa have documented high prevalence of toxoplasmosis in people and animals. Information about the epidemiology and public health impact of toxoplasmosis in Ghana is limited or non-existent. **Methods:** A cross-sectional prevalence study was conducted in the Cape Coast municipality, the capital of the Central Region, as a phase one study. Study participants were enrolled from the two main hospitals in the Cape Coast municipality. Participants provided a blood sample and answered a survey containing questions on socio-demographic and clinical information, as well as general and specific risk factors for toxoplasmosis. Commercial ELISA kits were used to determine presence of anti-*Toxoplasma* specific IgG and IgM antibodies. Univariate and multivariable logistic regression models were used to assess risk factors for seropositivity. **Results:** A total of 229 subjects between the ages of 13 and 50 years including males and females were recruited into the study. The overall IgG seroprevalence in the Cape Coast municipality was 72.5%, with a very low IgM seroprevalence of 0.87%. IgG seroprevalence was higher in high school graduates compared to university graduates, and highest among study participants with no education. Prevalence of the infection significantly increased with increasing age ($P<0.001$). Presence of cat(s) in a house or gender did not show a statistically significant association with serostatus. The type of meat consumed did not show any association with toxoplasmosis, however the likelihood of seropositivity increased significantly with increasing number of different types of meat consumed ($P<0.001$). **Conclusions:** There is

considerable evidence of prior exposure in the population of Cape Coast. Findings suggest that meat as a source of toxoplasmosis in the study area should be evaluated further.

**Board 348. Prevalence of Influenza A Antibodies and Risk Factors for Exposure in Thai Free-Grazing Ducks**

**A. Beaudoin**[1], J. Sasipreeyajan[2], P. Kitikoon[3], P. Schreiner[4], R. Singer[1], A. Amonsin[2], S. Pakinsee[5], J. Bender[1];
[1]Department of Veterinary Population Medicine, University of Minnesota, Saint Paul, MN, USA, [2]Faculty of Veterinary Science, Chulalongkorn University, Bangkok, Thailand, [3]Agricultural Research Service, United States Department of Agriculture, Ames, IA, USA, [4]School of Public Health, University of Minnesota, Saint Paul, MN, USA, [5]College of Public Health Sciences, Chulalongkorn University, Bangkok, Thailand.

**Background:** Free-grazing duck (FGD) farmers graze flocks of thousands on post-harvest rice fields, where ducks feed on rice, insects and snails. Research has shown that FGD flocks and rice cropping intensity are spatially associated with highly pathogenic avian influenza (HPAI) H5N1 outbreaks in Thailand. It has also been theorized that these flocks are a reservoir for HPAI H5N1 viruses in inter-outbreak periods. Little is known about influenza infection and transmission in this population, nor about influenza virus subtypes to which FGD may be exposed. Goals of this study were to describe management practices of FGD flocks, estimate seroprevalence of influenza A virus antibodies, including H5 and H7 subtypes, and identify risk factors for exposure to influenza viruses. **Methods:** This cross-sectional serologic study was conducted in Suphanburi Province (SP), Thailand in July and August 2010. Thirty serum samples were collected from each of 201 registered FGD flocks (6,810 samples) for analysis with three commercial enzyme-linked immunosorbent assays (ELISAs) to detect influenza A nucleoprotein (NP), and hemagglutinin H5 and H7 proteins. Samples positive on H5 ELISA were further evaluated using HPAI H5N1 hemagglutination inhibition (HI). **Results:** The nonsubtype-specific assay (NP ELISA) identified antibodies to influenza A virus in 97% of flocks. Of ducks tested with subtype assays (1642 samples), 37% were seropositive on H5 ELISA and 8.2% were seropositive on H7 ELISA. Twenty-eight H5 ELISA-positive ducks also had positive antibody titers (≥1:40) by H5N1 HI assay. Flock and management characteristics were assessed as potential risk factors for influenza exposure using univariate and multivariable generalized estimating equation methods, with flock as a cluster variable. Significant factors included age, contact with other poultry, flock visitors and purchase age. **Conclusions:** This work shows that some FGD in SP have positive antibody titers to HPAI H5N1 virus, and ducks were exposed as recently as March 2010, a time when no outbreaks were detected. FGD flocks are also widely exposed to other unidentified influenza A viruses. Therefore, rigorous surveillance of these flocks is recommended and activities can be targeted towards FGD flocks at risk due to age or contact rates with other birds and visitors.

**Board 349. Does Intra-Host Virion Diversity Explain Epidemiological Patterns Among Rabies Strains?**

**T.M. Schneider**[1], R. Davis[2], M. Moore[2], S. Wisely[1], C. Hanlon[2];
[1]Kansas State University, Manhattan, KS, USA, [2]Kansas State University Rabies Lab, Manhattan, KS, USA.

The ability of rabies to readily switch hosts and form new reservoirs is a unique characteristic even among rapidly evolving RNA viruses which all depend on high mutation rates for emergence, transmission, and evolution. For example, within host virion diversity (the quasispecies) has been implicated in the rapid transmission and virulence of both HIV and Hepatitis C. In this project we examine patterns of quasispecies diversity using next generation pyrosequencing in multiple regions of the genome of two strains of skunk rabies. Virion sequence data were derived from brain tissue of infected striped skunks, *Mephitis mephitis*, and domestic cats, *Felis catus*. We tested three hypotheses regarding diversity in the quasispecies. 1) Because the Nucleoprotein (N) provides protection to the

genetic material and is, thus, under constraining selection but the glycoproteing (G) has antigenic sites under positive selection, we hypothesized that the G protein would have more variation within individuals than the N protein. 2) We compared quasispecies diversity in two strains: North Central Skunk Strain (NCSK) and South Central Skunk Strain (SCSK). We hypothesized quasispecies variation in the antigenic sites of the G gene would be higher in the SCSK strain because this strain appears to be spreading more quickly through skunk populations based on case data over time. In RNA viruses, increased variation in the G gene is correlated with increased virulence and transmission. 3) In addition, we compared quasispecies diversity in the reservoir species (n=12, striped skunks) versus a common spillover species (n=12, domestic cats). We investigated this hypothesis across the two rabies strains. We hypothesized that skunks as the reservoir species would have higher quasispecies variation than cats because the skunk immune system may provide more immunological roadblocks and, thus, the virus may require more antigenic diversity to invade host cells. Alternatively, quasispecies diversity in skunks may be lower, since the virus has adapted to skunks as a host and the virus has adapted to skunks as a host and, may require less antigenic diversity to travel the central nervous system than in cats. Using a next generation sequencing technology to look at the virion diversity in the two different hosts, two different strains, and the two different regions we examined new questions of rabies transmission.

**Board 350. Vector-Borne and Zoonotic Disease Surveillance on Military Bases in Mainland Japan**

**W.K. Reeves**[1], L. Lloyd[1], S.P. Wolf[2], W. Walker[3], R. Paul[4];
[1]US Air Force School of Aerospace Medicine/PHR, Wright-Patterson Air Force Base, OH, USA, [2]USAF Headquarters Air Mobility Command/A7OO, Scott AFB, IL, USA, [3]US Air Force School of Aerospace Medicine/DET-3, APO, AE, USA, [4]Public Health Command Region- Pacific, APO, AE, USA.

**Background:** Zoonotic diseases and parasites infect animals and circulate naturally in animal populations. They do not require a human host. The vast majority of emerging infectious diseases are zoonotic. Entomologists, pest managers, veterinarians, and veterinary practitioners are one of the first lines of defense against zoonotic diseases, because they can detect and report them to public health officials. Entomological and veterinary information is often unavailable for public health professionals and reportable event surveillance systems do not capture data from arthropod surveillance and veterinary clinics. **Methods:** An initial step in the surveillance of zoonotic disease in the Pacific Region was a physical audit of all animal records at the US military veterinary care facilities in mainland Japan. We visited the veterinary care facilities at Camp Zama, Iwakuni Marine Air Station, Sasebo Naval Base, Misawa Air Base, Yokota Air Base, and Yokouska Naval Base and manually reviewed ~6400 animal medical files to annotate any parasitic or pathogen activity reported in the last 10 years (2000-2010). We also compile the arthropod surveillance and pathogen screening results from more than 20000 arthropod-vectors of zoonotic diseases collected from 2009-2011 throughout Japan. **Results:** A wide range of pathogens including the agents of rickettsial diseases and arboviruses were detected. Our report focuses on the presence and trends in major pathogenic parasitic agents likely to infect humans or cause diseases in military working dogs. **Conclusions:** Zoonotic and animal diseases pose a threat to military, civilians, and working animals throughout Japan. These threats include scrub typhus, murine typhus, Japanese encephalitis virus, and heartworm.

**Board 351. Susceptibility of American Alligators to Avian and Human Influenza**

**J.W. Finger, Jr.**, B.L. Temple, C.A. Jones, T. Jelesijevic, E.W. Uhl, R.J. Hogan, T.C. Glenn, S.M. Tompkins;
University of Georgia, Athens, GA, USA.

**Background:** The emergence of zoonotic influenza A, including highly pathogenic avian H5N1 and pandemic H1N1 has sparked worldwide interest in identifying and understanding which and how many species can be infected and serve as reservoir or vector species. Studies have indicated that a wide array of amniotes, including mammals, birds, and reptiles, are susceptible to infection with influenza. Swine

are well-known mixing vessels for multiple strains of influenza, enabling the emergence of reassortant strains, but other species may also have this trait. The infectious diseases of reptiles are vastly understudied. Crocodilians are the closest living relatives to birds and are known to inhabit areas with influenza harboring birds. Because crocodilians frequently eat birds, especially ill or injured individuals, there is significant likelihood of contact and possible transmission of influenza from birds to crocodilians. Millions of alligators occur in the wild and on farms with hundreds of thousands per year harvested for meat and, leather. With increasing numbers of alligators on farms, in the wild, and encroachment of houses into natural habitats, interactions between humans and alligators are increasing. **Methods:** We developed two primary cell lines, an alligator fibroblast cell line and an alligator respiratory epithelial cell line. Both cell lines were infected with human and avian viruses. Embryonated alligator eggs were infected with each of the avian strains. Allantoic fluid was extracted to measure viral titer. **Results**: Our results demonstrate that alligator fibroblasts and alligator lung epithelial cells are susceptible to infection with both human and avian influenza strains. In addition, embryonated alligator eggs were also shown to be vulnerable to infection with avian strains. **Conclusions:** Additional work is needed at the whole-organism level, but our data indicate that alligators could serve as reservoir or vector species and may serve as mixing vessels between avian and human strains of influenza.

**Board 352. Assessing Uncertainty in Estimates of Nipah Encephalitis Occurrence from Surveillance at a Tertiary Care Hospital in Bangladesh (2007–2010)**

**J.R. Pulliam**[1], E.S. Gurley[2], A.M. Kilpatrick[3], M.J. Hossain[2], H.S. Sazzad[2], A.S. Ekram[4], R.C. Paul[2], P.E. Rollin[5], P. Daszak[6], S.P. Luby[2];
[1]University of Florida, Gainesville, FL, USA, [2]International Centre for Diarrhoeal Disease Research, Bangladesh, Dhaka, Bangladesh, [3]University of California, Santa Cruz, Santa Cruz, CA, USA, [4]Rajshahi Medical College Hospital, Rajshahi, Bangladesh, [5]Centers for Disease Control and Prevention, Atlanta, GA, USA, [6]EcoHealth Alliance, New York, NY, USA.

**Background:** Nipah virus (NiV) is a bat-borne virus that causes outbreaks and sporadic cases of acute encephalitis in people in rural Bangladesh, with an average of 10 cases detected per year. From 2007-2010, active, hospital-based surveillance for NiV was conducted at Rajshahi Medical College Hospital (RMCH). We assessed the risk that cases could be missed by surveillance. **Methods:** We constructed a probability model of the surveillance process and used data from RMCH and its catchment area to estimate the probabilities of an encephalitis patient not seeking care, of an eligible patient not being added to the surveillance log, of logged patients not having diagnostic samples collected, of collected samples not being tested, and of a false negative test. Together, these estimates were used to calculate the overall probability of a NiV case in the hospital's catchment area being missed by surveillance. **Results:** Data from a hospital utilization survey indicate that at least 54% of persons in the catchment area with symptoms consistent with NiV encephalitis did not seek care at RMCH. We also compared entries on the NiV surveillance log to other RMCH records to estimate that at least 7% of admitted patients who met the case definition were missing from the NiV surveillance log. Of cases on the log, 49% were not sampled, and 15% of samples were not tested. Finally, we accounted for the relationship between the timing of sample acquisition (relative to disease onset) and seroconversion. Considering expected seroconversion times, we estimated that 27% of *tested* NiV cases would have gone undiagnosed. Overall, we estimate a NiV case in the hospital's catchment area would have had at minimum an 87% chance of being missed by hospital-based surveillance. The full probability model was then used to calculate a confidence interval for the number of unrecognized NiV cases entered on the surveillance log during the putative NiV season, assuming cases lost at each step were an unbiased subset of the previous category (95% CI 0-9, December to May all years). **Conclusions:** This analysis suggests that the actual burden in areas under active surveillance may be an order of magnitude greater

than the number of detected cases. Improving surveillance could better identify potential threats from viral changes and additional opportunities for prevention.

## Laboratory Support

Wednesday, March 14
12:30 PM – 1:30 PM
Grand Hall

**Board 353. A Molecular Approach for the Diagnosis of Bacterial Meningitis in Culture-Negative CSF and Blood Specimens**

**D. Wroblewski**[1], T. Halse[1], T. Quinlan[1], J. Edwards[1], K. Hamlin[2], N. Dumas[1], D. Weiss[3], K. Musser[1];
[1]Wadsworth Center-NYSDOH, Albany, NY, USA, [2]CGH/DPDM, Atlanta, GA, USA, [3]NYC DHDM, New York City, NY, USA.

**Background:** Diagnosis of acute bacterial meningitis is difficult and while culture is the gold standard, the rate of isolation is significantly reduced once antibiotic treatment is initiated. Molecular testing can provide a rapid approach to determine the causative agent even when no viable organism is present. **Methods:** Our laboratory has developed a molecular testing algorithm which includes 17 real-time PCR targets to detect and serotype the causative agent of bacterial meningitis. Using this molecular approach, the presence of *Neisseria meningitidis* (Nm), *Streptococcus pneumoniae* (Spn), *Haemophilus influenzae* (Hi), and *Streptococcus agalactiae* DNA (GBS), as well as, identification of the serogroups of Nm (A,B, C, Y and W135) and the serotypes of Hi DNA (A through F) can be determined. **Results:** We evaluated this molecular approach on 174 whole blood and CSF specimens from 120 patients over a 37-month period; 46 patients had multiple specimen types submitted. Bacterial DNA was detected in 56 (32%) specimens of 44 patients (37%): Nm (33), Spn (17), Hi (4), and GBS (2). Of the 33 specimens positive for Nm DNA, 12, 12, 7, 1 and 1 were determined to be serogroups B, C, Y, W135, and negative, respectively. Of the 4 specimens positive for Hi DNA, 1 and 2 were determined to be serotype F and non-typeable, respectively. We found that among the 17 positive-paired specimens ~90% had a lower CT value for CSF than blood, indicating a greater bacterial load present in CSF. **Conclusions:** This algorithm was successful for providing a diagnosis in 37% of the culture-negative patients tested. When available, CSF was the preferable specimen but blood specimens were often adequate for diagnosis. This approach allows rapid identification (within 24 hr) and can be used to guide treatment, prophylaxis, and appropriate infection control. Additionally, molecular serogrouping of the causative agent of bacterial meningitis in culture-negative specimens can provide additional data to monitor.

**Board 354. T-cell Immunophenotyping in Early Lyme disease**

**L.A. Crowder**[1], J.N. Aucott[2], M. Soloski[2];
[1]Lyme Disease Research Foundation of Maryland, Lutherville, MD, USA, [2]Johns Hopkins University, School of Medicine, Baltimore, MD, USA.

**Background:** Lyme disease is the most common emerging infectious disease in the United States with 38,000 cases in 2009. *Borrelia burgdorferi (*B.b.). B.b. is thought to be an extracellular infection not known to produce toxins, suggesting that host tissue inflammatory response is likely the primary cause of symptoms and signs of the infection. The humeral immune response is thought to be the primary means of host control of infection. **Methods:** Seventy-six patients with clinically diagnosed erythema migrans and untreated early Lyme disease were enrolled in a prospective cohort study. Patients were matched to healthy controls based on gender, age, and medical comorbidities. Patients were seen for 7 study visits, controls for 4 visits over a two-year period where whole blood, serum, peripheral blood

lymphocytes (PBLs), DNA and RNA were collected. Immune phenotyping of fresh PBLs by flow cytometry was performed within 24 hours of each visit. **Results:** Cases had significantly lower lymphocyte counts before treatment (mean (m) = 1.305 vs. m= 1.916, p=0.0039), as well as higher AST and ALT liver function tests (m=48.79 vs. m=25.93, p=0.0096 and m=50.92 vs. 26.33, p=0.0076, respectively) than controls. CD4 and CD8 percentages of total T-cells were not different between patients and controls. However, patients showed increased % of CD4+ and CD8+ effector memory T cells (m= 15.3 and 23.65, respectively) compared to controls (m=7.48 p= 0.006 and m=15.22 p=0.024, respectively). Analysis of markers reflective of effector function showed increased levels of CD4+Th2 and CD8 Tc2 populations compared to controls (m=2.61 vs. m=1.36, p=0. 047 and M=3.97 vs. m=1.08, p=0.038, respectively). **Conclusions:** Immune phenotyping of T-cell subsets in early Lyme disease showed an unexpected increase in both CD4 and CD8 effector memory populations. Both CD4 and CD8 populations had increased percentages of Th2 and Tc2 subsets relative to non-Lyme controls. Future analyses of this data will focus on examining the relationship of immune response to disease outcomes, including the development of symptoms of post treatment Lyme syndrome. We are also interested in studying the elevation of CD8 Tc2 cells and how they may be associated with diminished antibody response in patients who fail to seroconvert with infection.

## Antimicrobial Resistance

Wednesday, March 14
12:30 PM – 1:30 PM
Grand Hall

**Board 355. Analysis of Antimicrobial Resistance Mechanisms in Multi Drug Resistant (MDR) *Salmonella enterica* by High-Throughput DNA Sequencing**

**J.G. Frye**, L.M. Hiott, J.B. Barrett, L.M. Glenn, M.D. Englen, C.R. Jackson, P.J. Fedorka-Cray;
USDA, Athens, GA, USA.

**Background:** Multi drug resistant (MDR) *Salmonella enterica* is found in food animals and may consequently pose a risk to humans through food borne transmission. To understand the mechanisms that drive this problem, the genetic elements that cause MDR and its spread need to be determined. These MDR elements in *Salmonella* are often associated with transmissible plasmids. **Methods:** MDR *Salmonella* isolated from healthy animals at slaughter were screened by multiplex PCR for 18 plasmid replicon types (Carattoli *et al.* 2005). The isolates were further screened by DNA microarray analysis for 1,269 resistance and plasmid genes (Glenn *et al.* 2011). From these results, 12 isolates with the most diverse plasmid replicons and antimicrobial resistance genes detected were selected for plasmid DNA analysis by 454 pyro-sequencing (Roche, Branford, CT). **Results:** Plasmid sequencing identified genes, plasmids, and genetic elements consistent with the PCR and microarray data. These included multiple alleles of resistance genes such as $bla_{CMY-2}$, $bla_{TEM-1}$, *floR*, *aac*(3), *aadA*, *aphA1*, *strA*, *strB*, *sulI*, *sulII*, *dfrA*, *dhf*, *tet*(A)(B)(C)(D), and *tet*R. Plasmid backbone genes detected were homologous to replication, maintenance, and conjugative transfer genes found in several plasmid replicon groups. Long contiguous DNA sequences were assembled from the data by further analysis. Sections of these sequences (up to 30,000 bp long) were nearly 100% homologous to antimicrobial resistance and virulence plasmids found in the NCBI database. These included members of the IncA/C, I1, HI1, F, and P plasmid replicon types. Other contiguous sections had less homology, or homology to sequences found on other genetic elements, or limited homology to any known sequences indicating high levels of variability in these *Salmonella* MDR plasmids. **Conclusions:** These data demonstrate that plasmids carrying resistance genes in *Salmonella* are highly diverse in their replicon types, gene content, and overall structure. In addition

to having high variability in the antimicrobial resistance gene cassettes, plasmid backbone regions are also variable and some plasmids may be chimeras of multiple plasmid backbones. Sequence analysis of these MDR plasmids improves our knowledge of the genetic mechanisms responsible for the spread of MDR *Salmonella* in animals.

**Board 356. Decrease in Susceptibility of Fluoroquinolone Found against *Mycobacterium tuberculosis* in Urban Settings**

**J.S. Verma**[1], U. Kumar[1], P. Raghuvanshi[1], N. Manzoor[2], D. Nair[1];
[1]Vardmann Mahaveer Medical College & Safdarjung Hospital, New Delhi, India, [2]Jamia Millia Islamia University, New Delhi, India.

**Background:** Ofloxacin (OFX) is one of the potent fluoroquinolone (FQ) recommended to treat MDR-TB. Over a decade, the pre-exposure of this drug for the treatment of other bacterial infections has resulted in acquisition of FQ resistance among *Mycobacterium tuberculosis* (*M. tuberculosis*) strains. **Methods:** A study was undertaken in a tertiary care center in the capital city (India) to assess the drug resistance trends of OFX among susceptible and multidrug resistant (MDR) strains of *M. tuberculosis*. A total of 102 *M. tuberculosis* isolates (47 susceptible to first-line drugs and 55 MDR isolates) were screened for susceptibility testing of OFX with a critical concentration of 2 µg/ml by Lowenstein Jensen (LJ) proportion method. **Results:** The results showed 40 (85.1%) isolates among 47 susceptible isolates and 34 (61.8%) isolates among 55 MDR isolates, were found to be susceptible to OFX. Fisher's exact test showed significant *P*-value (0.0136) demonstrating 1.377 fold (95% confidence interval) increased risk to become resistant to OFX than susceptible isolates. **Conclusions:** These finding shows decreased OFX susceptibility is not only limited to MDR isolates but also increasingly seen in susceptible strains as a result of drug abuse. Our finding were not alarming, but highlights the general risk of acquiring resistance to OFX, jeopardizing the potential for these drugs to be used as second-line anti-TB agents in the management of drug-resistant TB and creating incurable TB strains.

**Board 357. Antimicrobial Resistance among Isolates of Invasive Pneumococcal Disease in New York State from 1999 through August 2011**

**J.A. Nadeau**[1], S.A. Currenti[1], D.S. Abraham[1], S.M. Solghan[1], N.B. Dumas[2], N.L. Spina[1], J.B. Karr[3], S.M. Zansky[1];
[1]New York State Department of Health, Albany, NY, USA, [2]New York State Department of Health - Wadsworth Center, Albany, NY, USA, [3]New York State Department of Health, Rochester, NY, USA.

**Background:** Prior to the introduction of the 7-valent pneumococcal conjugate vaccine (PCV7), antimicrobial resistant (AMR) *Streptococcus pneumoniae* in the United States was most common in 5 serotypes (6B, 9V, 14, 19F, and 23F). To counteract the increasing incidence of invasive pneumococcal disease (IPD) and AMR seen among non-PCV7 serotypes, the 13-valent pneumococcal conjugate vaccine (PCV13) added serotypes 1, 3, 5, 6A, 7F, and 19A to the PCV7 serotypes (4, 6B, 9V, 14, 18C,19F, and 23F). As PCV13 use increases, changes in the serotype distribution and AMR among non-vaccine serotypes (NVTs) is expected. This study analyzes the AMR patterns for isolates of IPD in New York State (NYS) from 1999 through August, 2011. **Methods:** The NYS Emerging Infections Program (EIP) has conducted active, routine IPD surveillance within 15 upstate NY counties since 1999. Isolates of IPD, for all ages, were sent to the CDC for serotype and AMR testing. AMR tests included Penicillin (PEN), Cefotaxime (TAX), Vancomycin (VAN), and Amoxicillin (AMO). Testing for Ceftriaxone (CFT) AMR has been conducted since 2004. MIC values were categorized as susceptible, intermediate, and resistant according to the 2011 Clinical and Laboratory Standards Institute breakpoint definitions. Isolates were classified as part of the 7 serotypes contained in PCV7 (Sero7), the 6 additional serotypes contained in PCV13 (Sero6), or NVTs. **Results:** In total, 3432 isolates were tested. AMR to PEN, AMO, TAX, and VAN was 2.1%, 3.8%, 1.2%, and 0%, respectively. Among the 1885 isolates tested for CFT, 1.5% were

resistant. No PEN resistance was found within the Sero7 group since 2003. Sero6 isolates showed no PEN AMR from 2000 to 2003, then increased to a high of 13.2% (n=18) in 2009 (p<0.001). NVT 15A (5/22) and 35B (4/64) showed increased PEN AMR compared to other NVTs (p=0.02). Similar patterns were seen for intermediate PEN resistance and other antimicrobials tested. **Conclusions:** Antimicrobial resistance was shown to be highest among Sero6 types and lowest among NVTs. While cases of IPD caused by the Sero7 types are still detected among those over 5 years of age, there is no resistance among these isolates. As the use of PCV13 increases, current low levels of AMR among NVT IPD may increase as was seen with Sero6 isolates post PCV7 implementation.

**Board 358. Integration of Different Resistance Gene Cassettes in Variable Region of Class 1 and 2 Integrons of *Shigella* spp.**

**N. Eftekhari**[1], B. Bakhshi[2], M.R. Pourshafie[3], B. Zarbakhsh[4], F. Eghbalpour[4], M. Rahbar[5], M. Hajia[5], M. Mohammad zadeh[6], N. Amirmozaffari[1], K. Ghazvini[7];
[1]Islamic Azad University, Science and Research Branch, Tehran, Iran, Islamic Republic of, [2]Tarbiat Modares University, Faculty of Medical Sciences, Tehran, Iran, Islamic Republic of, [3]Pasteur Institute of Iran, Bacteriology Department, Tehran, Iran, Islamic Republic of, [4]Pasteur Institute of Iran, Biotechnology Department, Tehran, Iran, Islamic Republic of, [5]4Center for Disease Control, Ministry of Health and Medical Education, Tehran, Iran, Islamic Republic of, [6]Department of Microbiology, Milad Hospital, Tehran, Iran, Tehran, Iran, Islamic Republic of, [7]Avecenna Research Institute Microbiology and Virology Research Center, Tehran, Iran, Islamic Republic of.

**Background and Objective:** *Shigella* spp. is one of the major causative agents of diarrhea in both developing and developed countries. It causes gastroenteritis especially in children which is annually reported in Iran. The aim of this study was to confirm the identity of *Shigella* spp. by a species-specific PCR and to detect the diversity and resistance gene content of class 1 and 2 integrons among *Shigella* spp**. Material and Methods:** *Shigella* strains were isolated from stool samples in summer 2010 and characterized by serotyping, biochemical tests and species-spacific PCR of *ipaH* gene. Class 1 integron was detected by amplification of class-specific *integrase* gene and their resistance gene content was identified by amplification and sequencing of their variable resistance region. **Results:** Among the total 37 strains (identified as *ipaH*$^{+}$) *S.sonnei* was the most frequent serogroup (56.7%), followed by *S.flexnery* (32.4%), *S.boydii* (8.1%) and *S.dysentriae* (2.7%). Twelve strains (34%) harbored class1 integrons. Three types of class1 integrants were identified in this study including 750, 1500 and 1600 bp gene cassettes among 8.1, 2.7, and 18.9% of isolates which correspond to *dihydropholate reductase (dfrA7), aminoglycoside adenyl transferase (aadB)/ chloramphenicol acetyltransferase (catB3) and dihydropholate reductase (dfrA17)/ aminoglycoside adenyltransferase (aadA5)*, respectively, as identified by sequence analysis of the variable region of class1 integron. Two types of class2 integrons were identified harboring *dfrA1* and *sat1* (1500 bp) in 43.2% (including *S.sonnei , S.flexnery* and *S.boydii* ) and *dfrA1*, *sat1* and *aadA1* (2300 bp) in 24.3% (only *S.sonnei*) of isolates. **Conclusions:** The predominance of *dfr* and *aad* family gene cassettes reflects the stability and persistence of this group of resistance gene cassettes in class 1 integron. This finding, together with the absence of relative diversity in arrangement and organization of gene cassettes, suggests the transferability of the entire integron structure rather than individual gene cassettes.

**Board 359. Multidrug-resistant Patterns among *Salmonella* Isolates from Retail Meats in the National Antimicrobial Resistance Monitoring System (NARMS), 2002–2010**

**E. Tong**, S. Ayers, J. Abott, S. Friedman, S. Zhao, P. McDermott;
U.S. Food & Drug Administration, Center for Veterinary Medicine, Office of Research, Laurel, MD, USA.

**Background:** Non-typhoidal *Salmonella* (NTS) is frequently associated with foodborne illness, and is commonly isolated from food animals and their derived meats. Multidrug-resistant (MDR) variants have

emerged reducing therapeutic options in cases of invasive infections in both human and animals. The purpose of this study is to determine the prevalence of multidrug-resistant patterns among non-typhoidal *Salmonella* isolates from retail meats in the National Antimicrobial Resistance Monitoring System (NARMS). **Methods:** NARMS retail meat products include chicken breast (CB), ground turkey (GT), ground beef (GB) and pork chops (PC) collected by 11 state laboratories. Isolates were serotyped and tested for susceptibility to 15 antimicrobials agents by broth microdilution. We describe the MDR patterns of NTS recovered from NARMS retail meats (2002-2010) based on the public health concern. **Results:** From 2002-2010, 39.2% of NTS isolates (1,051/3,078) in retail meats were pansusceptible, while over 55% (1,693/3,078) were resistant to ≥2 antimicrobial classes. MDR *Salmonella* phenotypes differed by meat product and serotype, with Typhimurium CB isolates having the most MDR ≥3 classes. ACSSuT and MDR-AmpC are patterns of public health concern to humans. GT was most prevalent in ACSSuT accounting for 33.9% (21/62), followed by 27.4% GB (17/62), 21% CB (13/62) and 17.7% PC (11/62). Prevalence of MDR-AmpC was highest among GT and GB with 40% (16/40) and 35% (14/40) respectively, followed by 17.5% CB (7/40) and 7.5% PC (3/40). *Salmonella* Typhimurium accounted for 32.3% (20/62; PC=7, CB=7, GT=4, GB=2) of MDR ACSSuT, while 19.4% (12/62; GB=8, PC=3, GT=1) were from Newport. The highest prevalence of MDR-AmpC was detected in *S*. Newport with 30% (12/40; GB=8, PC=3, GT=1), followed by Dublin with 15% (6/40; GB=6). **Conclusions:** Results indicate that MDR patterns of critical human importance were found in NARMS retail meat and stress the need for sustained surveillance of foodborne pathogens in retail foods.

## Vulnerable Populations

Wednesday, March 14
12:30 PM – 1:30 PM
Grand Hall

### Board 360. Health Profile of International Students Studying in the United States: The American College Health Association Survey, 2008–2010

**E.A. Yanni**[1], T. Bell[1], V. Leino[2], S. Even[3], N. Cohen[1], D. Posey[1], Y. Liu[1], C. Brown[1], N. Marano[1];
[1]Centers for Disease Control, Atlanta, GA, USA, [2]American College Health Association, Linthicum, MD, USA, [3]University of Missouri, Columbia, MO, USA.

**Background:** Unlike immigrants, international students attending US colleges are considered temporary residents and are not required to undergo predeparture medical screening before coming to the United States. During 2009-2010, 3.5% of 19 million students enrolled in American universities were international. **Methods:** The American College Health Association conducts an annual national survey among university students to collect data about their health status, and perceptions about relevant health topics. The aim of this study was to compare the health knowledge and health status among international (n=16,338) with those of US students (n=157,143). Bivariate analyses were done by using Chi-square or Fisher's exact tests. **Results:** Forty percent of international students and 34% of US students were male (p<0.001). Of international students, 48% were Caucasian and 36% were Asian, versus 67% and 10% of US students, respectively (p<0.001). Forty-eight percent of international students had college health insurance, 47% were covered by their parents' insurance or another plan, 1.5% did not know if they had insurance, and 3.5% had no insurance, versus 13%, 80% , 1% , and 6% of US students, respectively (p<0.001). International students were less likely than US students to report receiving information from their colleges about different health topics (p<0.001), but were more likely to get mental health counseling (prevalence ratio [PR] =1.11, 95% confidence interval [CI] =1.07-1.16). US students were more likely than international students to report getting hepatitis B vaccine, but less

likely to have received varicella vaccine (p<0.001). International students were more likely to report having chlamydia (PR=1.22, CI=1.05-1.42), gonorrhea (PR=2.08, CI=1.68-2.59), hepatitis B (PR=2.70, CI=2.26-3.20), HIV (PR=2.47, CI=2.00-3.07), and tuberculosis (TB) (PR=2.74, CI=2.30-3.27) within the previous 12 months. **Conclusions:** Although 95% of international students had health insurance, they were less likely to have access to health information provided by universities and were significantly more likely to have STDs, hepatitis B, HIV, and TB. More culturally tailored health prevention measures are needed to reduce the burden of infectious disease among international students studying in the United States.

**Board 361. Differences by Age Group in the Prevalence of Diarrhea and Vomiting, Rates of Seeking Health Care, and Stool Sample Submission: FoodNet Population Survey, 1996–2007**

**M.E. Kendall**[1,2], E. Scallan[3], S.A. Greene[4,1], P. Ryan[5], T. Robinson[6], B. Shiferaw[7], S.L. Lathrop[8], R. Marcus[9], O.L. Henao[1];
[1]Centers for Disease Control and Prevention, Atlanta, GA, USA, [2]Atlanta Research and Education Foundation, Atlanta, GA, USA, [3]Colorado School of Public Health, Aurora, CO, USA, [4]The Carter Center, Atlanta, GA, USA, [5]Maryland Department of Health and Mental Hygiene, Baltimore, MD, USA, [6]Minnesota Department of Health, St. Paul, MN, USA, [7]Oregon State Public Health Division, Portland, OR, USA, [8]University of New Mexico, Albuquerque, NM, USA, [9]Yale University School of Public Health, New Haven, CT, USA.

**Background:** An estimated 179 million episodes of diarrhea or vomiting occur in the US each year; an important fraction of these illnesses are caused by agents transmitted by eating contaminated food. Many factors influence the occurrence of diarrhea, vomiting, and health care seeking behaviors, including age, medical insurance status, and concurrent symptoms. Understanding differences by age group is useful for improving foodborne disease burden estimates and guiding public health decision making. **Methods:** From 1996 to 2007, five 12-month population-based telephone surveys were conducted in US states participating in the Foodborne Diseases Active Surveillance Network (FoodNet). We examined, overall and by age group (<5, 5-64, and ≥65 years), the percent of respondents reporting an acute diarrhea episode or any vomiting (with or without diarrhea) in the 30 days before interview, and the percent seeking medical care and submitting a stool sample for that illness. An acute diarrhea episode (ADE) was defined as 3 or more loose stools in a 24 hour period; data were adjusted for survey design, sex, and age. **Results:** Among 67,934 respondents, 11% reported ADE in the 30 days before interview. The frequency of ADE was significantly higher in the most recent survey, compared with previous survey cycles. Among respondents with ADE, 15% sought medical care, and of these, 17% submitted a stool sample. The proportion of ADE was highest in children <5 years (16%) and lowest in adults ≥65 years (6.5%). Children <5 years (20%) and adults ≥65 years (18%) sought care more often than persons aged 5-64 years (14%). Adults ≥65 years were the most likely to submit a stool sample (27%), except in the most recent survey cycle, when frequency of submitting a stool sample was lowest in this age group (11%). Among all respondents, 5.1% reported any vomiting. The proportion of respondents vomiting in the 30 days before interview was highest in children <5 years (13%) and lowest in adults ≥65 years (2.1%). **Conclusions:** Children <5 years have the highest frequencies of ADE and vomiting, adults ≥65 years have the lowest. Adults ≥65 years with ADE were more likely to seek medical care and submit a stool sample than other age groups. These differences by age group can be used by public health professionals to estimate the burden of foodborne illness in the United States.

**Board 362. Brucellosis among Veterinary Personnel of Delhi, India–A Sero-Epidemiological Study**

**G. Singh**, U.V.S Rana, S. Venkatesh, V. Mittal, R.Sharma, L.S.Chauhan;
National Centre for Disease Control, Delhi, India.

**Background:** Brucellosis is an infectious disease caused by Gram negative bacteria of genus *Brucella*. It is an occupational direct anthropozoonosis which causes a serious reduction of quality food, especially the animal proteins. Misdiagnosis and underreporting of the disease in human have led to lack of evidence for control activities of brucellosis. This study was conducted to generate evidence of brucellosis among veterinary personnel and provide inputs for prevention and control. **Method**: A cross-sectional study was conducted in community settings at veterinarian's work place, during 1st February to 30th April 2007. Written consent was taken from the willing government Veterinary personnel for the study. A pre-tested structured questionnaire was used for interview. Blood samples were collected and tested for brucellosis antibodies by standard tube agglutination test (STAT). Sero-prevalence rate and prevalence ratio were calculated and tested for significance **Results:** The study was done among 46.57% (333/715) veterinary personnel. The sero-prevalence rate was 11.7% (39/333) (95% CI: 8.62-15.8%) and prevalence ratio for exposed rural and unexposed urban area was 3.22(95%CI 1.6-6.25, $X^2$-13.66, P<0.00022). Major symptoms among sero-positive were Joint pain 41% (16/39) backache 23.1% (9/39) and dry cough 20.5% (8/39). The mean age of sero-positive was 47.15 years. **Conclusions:** The study shows high prevalence of brucellosis among government veterinary personnel. The rate being significantly higher in rural than urban area, based on these observations, Information, Education and Communication (IEC) activities for prevention and control of brucellosis among veterinarian were recommended.

## Global Vaccine Initiatives

Wednesday, March 14
12:30 PM – 1:30 PM
Grand Hall

**Board 363. Quality of Studies in China on Invasive Bacterial Disease Burden and Serotype Distribution, 1980–2010**

**Y. Huai**[1], J.K. Varma[1], AGEDD study team from Johns Hopkins Bloomberg School of Public Health, H.L. Johnson[2];
[1]CDC (China office), Beijing, China, [2]Johns Hopkins Bloomberg School of Public Health, Baltimore, MD, USA.

**Background:** Invasive bacterial infections such as *Streptoccus pneumoniae* (Sp), *Haemophilus influenzae type B* (Hib) and *Neisseria meningitidis* (Nm) are leading causes of illness and death worldwide. China has not yet incorporated Sp and Hib vaccines into its national immunization program. One frequently cited reason is the lack of high quality data regarding disease burden and serotype distribution in China. We systematically reviewed and assessed the quality of Sp, Hib, and Nm studies conducted in China. **Objectives:** To assess the quality of Sp, Hib, and Nm disease burden and serotype distribution studies conducted in China. **Methods:** We used the China National Knowledge Infrastructure (CNKI), a database of Chinese language peer-reviewed articles, to find studies of Sp, Hib, and Nm conducted in China from 1980-2010. We defined criteria *a priori* for study relevance and quality. Articles were reviewed in three stages. First, we double-screened titles and abstracts for potential relevance. Second, we double-abstracted data from potentially relevant studies to confirm their relevance. Finally, we performed complete data abstraction and quality assessment on studies confirmed to be relevant. **Results:** Of 1,531 articles identified in the preliminary search, 397 (26%) were potentially relevant. We abstracted data from 392 (99%) potentially relevant studies and confirmed that 93 (24%) had data about disease burden and/or pathogen serotypes. Of the 93 relevant studies, 2 (2%) met our definition of high quality with sufficient data to calculate incidence and mortality. Twenty (22%) reported the number of cases or deaths caused by Sp, Hib, or Nm. Almost all studies used inadequate diagnostic methods, relying on

specimens from non-sterile sites. No study enrolled a representative sample of the population or adjusted for health care-seeking behavior of patients. Three (3%) articles reported serogroup or serotype of Nm and Sp strains isolated from cerebrospinal fluid; none included data about patient immunization status. **Conclusions:** The Sp, Hib, and Nm disease burden literature from China is limited. Further studies are needed to assess current disease burden and serotype distribution to assist with vaccine policy and practice.

## Tuberculosis

Wednesday, March 14
12:30 PM – 1:30 PM
Grand Hall

**Board 364. Computational Identification of Cell Surface Antigens in *Mycobacterium tuberculosis* (H37Rv) for Potential Vaccine Candidates.**

**P.P. Mishra**[1,2], S. Gupta[3], M. Kekare[3];
[1]Open source drug discovery, CSIR, mumbai, India, [2]SS & LS patkar college, Goregaon west, Mumbai, Mumbai, India, India, [3]SS & LS patkar college, Goregaon west, Mumbai, mumbai, India.

**Background:** Tuberculosis (TB)**,** a disease caused by the *Mycobacterium tuberculosis* is a leading cause of the death worldwide from a bacterial infectious disease. The disease affects 1.8 billion people every year which is equal to one-third of the entire world population. Tuberculosis is cured only by drugs which are several decades old. Strains that are resistant to multiple antibiotics make the treatment inefficacious and hence demands for a better prevention than cure. Therefore, there is a need of new vaccine development which will help cure the disease. **Design/Methods:** In the view of vaccine development, proteins of *Mycobacterium Tuberculosis* (H37Rv) provide us the opportunity for locating several surface antigens involving bioinformatics approach. We used the computational programs like SignalP3.0, LipoP1.0, TMHMM, and BLASTP to analyze the complete bacterial proteome taken from KEGG database. **Results:** With the above programs we searched out highly conserved surface antigens as lipoprotein and cell wall anchored proteins. We highlighted 44 prominent surface antigens which may be involved in subunit vaccine development programs. **Conclusions:** This study indicates that the selection of any of the 44 vaccine leads can be a strong vaccine candidate. Not much progress has been made in the vaccine development for tuberculosis. Therefore, this research is an important step towards an era where a doctor can immunize population with a vaccine against tuberculosis and help the world to become tuberculosis free.

**Board 365. Drug Resistance in *Mycobacterium tuberculosis* Isolated from Endometrial Biopsies Obtained from Infertile Women**

**M. Singhal**[1,2], S. Prasad[2], S. Gupta[1], S. Negi[1], S.T. Jimmy[1], D.S. Rawat[1], A. Rai[1];
[1]National Centre for Disease Control, Delhi, India, [2]Maulana Azad Medical College, new Delhi, India.

**Background:** Tuberculosis continues to plague mankind. It is one of the important causes of infertility and morbidity among women of reproductive age-group worldwide. The extent of the problem has increased with the emergence of multidrug-resistant strains of *Mycobacterium tuberculosis*. Co-infection with HIV has only exacerbated the scenario. It is a serious worldwide public health problem, and drug resistance, particularly multidrug resistance (MDR), is a critical factor involved in TB control. **Methods:** We have characterized the sequences of the loci associated with multidrug resistance in twenty nine clinical samples of *M. tuberculosis* from India to identify the respective mutations. The loci selected were *rpoB* (rifampin), *katG* and the ribosomal binding site of *inhA* (isoniazid). Sequencing was carried-

out using the Big Dye terminator kit (ABI, USA) and the samples were loaded on to the ABI 3130xl genetic analyzer (ABI, USA). The sequences were submitted to Gene Bank at www.ncbi.nlm.nih.gov. **Results:** Of the total 29 samples, 31.1% samples showed mutations in one or more genes. Six different kinds of mutations were obtained in the *rpoB* gene which included two insertions and four different substitutions. Mutation in codons 516, 531, 571, 572 and 584 were found. Mutation in codon 531 was most frequently found. Two novel mutations were found outside the RRDR. We identified the common Arg463Leu and Ser315Thr substitutions in the *katG* locus. Mutations were also mapped in the ribosomal binding site of the *inhA* gene. **Conclusions:** The study confirms the previous mutations in addition to the presence of two novel mutations in the *rpoB* gene. Rifampicin thus serves as the alternate marker for detection of MDR-TB, as 90% of rifampicin resistant isolates are also resistant to isoniazid. Knowledge of the mutations can help in the use of *rpoB* genotyping as an epidemiological tool for rifampicin resistant M. tuberculosis isolates. Detection of new mutations advocates the need for new diagnostic method to be implemented in India. Our results additionally have implications for the development of methods for multidrug resistance detection and are also relevant in the shaping of future clinical treatment regimens and drug design strategies.

**Board 366. Antimycobacterial Immune Responses in HIV-infected Children Starting Antiretroviral Therapy in Lusaka, Zambia**

**H.C. Nkamba**[1], K.R. Lovett[2], S. Siziya[3], W. Moss[2];
[1]University Teaching Hospital (UTH), Lusaka, Zambia, [2]Bloomberg School of Public Health, Johns Hopkins University, Baltimore, MD, USA, [3]Department of Community Medicine, University of Zambia, Lusaka, Zambia.

**Background:** Children infected with HIV are at risk of developing tuberculosis (TB). Antiretroviral therapy has been linked to improved immune responses to TB. With the scaling up of antiretroviral therapy (ART) in sub-Saharan Africa and Zambia in particular, more HIV infected children will have access to treatment. The objectives of the study were to determine the magnitude and quality of immune reconstitution in HIV-infected children receiving antiretroviral therapy (ART) and to determine pathogen-specific immune reconstitution to *Mycobacterium tuberculosis*. **Methods:** A total of 59 children of age 9 months to 5 years initiating ART with a history of BCG vaccination from Matero Reference Clinic in Lusaka were enrolled in a prospective cohort study. Demographic and clinical data were collected using questionnaires. Blood samples were drawn before starting ART, at 3 months and 6 months for measurement of T cell subsets and PPD stimulation for intracellular cytokine staining. **Results:** After 6 months of ART, the median CD4 T cell percentage increased from 9.4% at baseline to 25.9% ($p < 0.001$). Total CD8 T cell percentage decreased from 42.8% pre-ART to 36.5% after 6 months of ART ($p = 0.010$). However, naïve CD8 T cells increased within the same period ($p = 0.038$). Both activated CD4 and CD8 T cells decreased after 6 months of ART ($p < 0.001$). On the other hand, both central memory CD4 and CD8 T cells increased after 6 months of ART ($p = 0.029$ and 0.021, respectively), while effector memory CD8 T cells decreased ($p = 0.006$). After 3 months of ART, CD4 T cells expressing IFN-γ decreased ($p = 0.033$) but after 6 months of ART the percentage increased to pre-ART levels. **Conclusions:** ART has a positive impact on HIV-infected children, likely reducing the risk of tuberculosis as evidenced by the increases in CD4 T cells critical to an effective immune response against TB. Before starting ART, anti-mycobacterial immune responses seem to be primarily driven by effector memory T cells while after ART by central memory T cells. Therefore, central memory T cells appear to be the primary cells in restoring specific immune responses. These findings have valuable implications for TB vaccine development strategies in HIV-infected children.

# Prevention Challenges for Respiratory Diseases

Wednesday, March 14
12:30 PM – 1:30 PM
Grand Hall

**Board 367. Risk Factors Associated with Respiratory Syncytial Virus (RSV) Incidence in a Cohort of Young Children in Urban Bangladesh**

**N. Homaira**[1], K. Hossain[1], S.P. Luby[1,2], E.S. Gurley[1], W.A. Petri[3], M. Rahman[1], M. Rahman[4], F. Zesmin[1], M.M. Alam[1], R.U. Zaman[1], Z. Rahman[1], A.M. Fry[2], J. Bresee[2], M.-A. Widdowson[2], R. Haque[1], E. Azziz-Baumgartner[2];
[1]ICDDR,B, Dhaka, Bangladesh, [2]CDC, Atlanta, GA, USA, [3]University of Virginia, Virginia, MD, USA, [4]IEDCR, Dhaka, Bangladesh.

**Background:** Respiratory syncytial virus (RSV) is one of the major contributors to childhood respiratory illness. Identifying modifiable risk factors associated with RSV infection may help develop interventions to reduce disease burden. We conducted a longitudinal study of young children in a densely populated, low-income urban community in Bangladesh to examine a range of potential risk factors associated with RSV infection. **Method:** We followed a cohort of children during April 2009−March 2011. At enrollment field assistants collected information about each child's birth weight and height, household, socio-economic, demographic characteristics and environmental exposures. Subsequently field assistants visited each child at their home twice weekly and if they developed respiratory symptoms they were referred to the study clinic where nasal washings were obtained. We tested respiratory specimens for RSV using real-time RT-PCR. We stratified the cohort into four age-groups (0−6 months, 6−12 months, 12−24 months, and 24−36 months) and used Poisson regression to identify individual risk factors for developing RSV associated respiratory illness in this cohort of children. **Results:** We followed 515 children for 740 child-years; 423 (82%) developed 1,322 episodes of respiratory illness. One hundred and seventy five (13%) episodes were laboratory confirmed for RSV. The RSV incidence/100 child-years was 23 for children aged 0-6 months, 29 for children aged 6−12 months, 33 for children aged 12-24 months, and 13 for children aged 24−36 months. On multivariate analysis we did not identify any significant risk factor except for children aged 6−12 months having more than five household members (rate ratio 2.3, 95% CI 1.2−4, p-value=0.008) was associated with increased risk of disease. **Conclusions:** We identified a high incidence of RSV infection. In a setting where population density exceeds1000 people/km2 it is likely that young children are at high risk of developing RSV infection due to overall crowding rather than the individual level risk factors we measured. Development of an effective vaccine or reduction of population crowding are among the few interventions that may reduce the risk of RSV infection in young children in this population.

**Board 368. Investigating the Burden of Influenza in Pediatrics at the University Teaching Hospital, Lusaka, Zambia**

**P. Simuskia**[1], A. Theo[1], H. Nkamba[2], M. Liwewe[1], M. Monze[3];
[1]Influenza Surveillance ,Virology Laboratory, University Teaching Hospital, Lusaka, Zambia, [2]Ministry of Health, University Teaching Hospital, Lusaka, Zambia, [3]Ministry of Health, Virology Laboratory, University Teaching Hospital, Lusaka, Zambia.

**Background:** Influenza vaccination of healthy children is encouraged because children are frequently hospitalized for influenza-attributable illnesses. However, most children with influenza are treated as outpatients, and scarce data are available on the burden of influenza in these children. Typical symptoms of influenza begin 2-3 days after exposure to the virus. Influenza produces an acute febrile respiratory illness with cough, headache, and for 3-4 days, with symptoms that may persist for as long as

2 weeks. Accurately diagnosing influenza A or B infection based solely on clinical criteria is difficult because of the overlapping symptoms caused by the various viruses associated with upper respiratory tract infection. **Methods:** We performed an investigation of respiratory infections in children under the age of 5 who were admitted at the University Teaching Hospital, Lusaka Zambia during the peak season of flu ( May -August 2011). At any sign of respiratory infection, we examined the children and obtained a combined nose and throat swabs for the detection of influenza using the ABI 7500 Fast real -time PCR assay. With consent, the parents filled out the case investigation forms,150 children with a history of influenza like illness(cough, fever, headache) and other respiratory illnesses such us bronchitis or pneumonia were captured. **Results:** Of the 150 cases investigated, 8 tested positive for Flu B, 1 tested positive for Flu A and 141 tested negative for influenza A or B. In detected cases of influenza acute pneumonia developed as a complication of influenza in 6% of the children. One child with Flu A had a severe case of otitis media. **Conclusions:** Influenza causes a substantial burden of illness on children and their families. Periodic vaccination of children under 5 years old might be beneficial for reducing the direct and indirect effects of influenza in pediatrics. Even though we got 141 negative specimens, it is important to investigate all suspected cases as influenza can be fatal.

**Board 369. The Influenza Pandemic of 1918–1919 in Two Remote Island Nations: Iceland and New Zealand**

**J.A. Summers**[1], M. Gottfredsson[2,3], N. Wilson[1], M.G. Baker[1];
[1]University of Otago, Wellington, New Zealand, [2]University of Iceland, Reykjavik, Iceland, [3]Landspitali University Hospital, Reykjavik, Iceland.

**Background:** Nations varied in their experience of, and response to, the 1918-19 influenza pandemic. However, certain epidemiological characteristics of this pandemic were repeated in many locations. In addition, island communities can provide unique opportunities to study the epidemiology of infectious diseases. Therefore we compared the epidemiology and public health response to this pandemic in two remote island nations, on opposite sides of the globe: Iceland and New Zealand (NZ). **Methods:** Historical accounts in both nations were reviewed, along with more recent analysis of the pandemics impact and course. **Results:** Both nations were exposed to the first wave of the pandemic strain mid-1918 via ships with infected passengers, as neither country implemented full maritime quarantine prior to exposure. The second wave for both nations occurred in October 1918 and was more virulent in terms of mortality compared to the first wave. There were 484 Icelandic and over 6,000 NZ pandemic-attributable deaths during 1918. Amongst the exposed European populations of the two islands, Iceland experienced a significantly higher mortality rate (830 vs 550 per 100,000) compared to NZ (rate ratio: 1.5, 95%CI: 1.4-1.6).Young adults of both nations suffered disproportionately during this pandemic, which is consistent with the "W-shaped" age distributed mortality found in many other populations around the globe. The results from both nations also indicate that rurality and better socio-economic conditions may have been a protective factor in terms of mortality. Whilst influenza was a notifiable disease in Iceland before the pandemic, unlike NZ (which delayed until mid-pandemic), officials in both nations delayed in enacting public health responses such as quarantine measures. However, there is evidence that early control measures in specific areas of both nations resulted in lower mortality rates. **Conclusions:** In summary, the epidemiology of the 1918-19 influenza pandemic was fairly similar for the exposed European populations of Iceland and NZ. Nevertheless, major differences were: the uniquely high burden on Maori in NZ; the significantly higher overall mortality rate in Iceland; and the success of Iceland's use of localised ship quarantining and protective sequestration which protected 36% of the population.

## Infectious Causes of Chronic Diseases

Wednesday, March 14
12:30 PM – 1:30 PM
Grand Hall

**Board 370. Autoimmune Pathogenesis of Rift Valley Fever Retinitis**

**A. LaBeaud**[1], S. Newman-Gerhardt[1], S. Muiruri[2], E. Muchiri[2], C. Peters[3], J. Morrill[3], C.H. King[4];
[1]Children's Hospital Oakland Research Institute, Oakland, CA, USA, [2]Ministry of Health, Nairobi, Kenya, [3]University of Texas Medical Branch, Galveston, TX, USA, [4]Case Western Reserve University, Cleveland, OH, USA.

**Background:** Rift Valley Fever (RVF) is an emerging infection with significant threat to human health, because it can progress to retinitis (10%), encephalitis (8%) and hemorrhagic fever (1%). The timing of Rift Valley fever virus (RVFV)-induced retinitis, at 7-20 days after the disease onset, suggests an autoimmune origin, as that delay matches the activation period of the adaptive immune system. This study was designed to determine if RVFV-related retinitis is associated with increased levels of anti-retinal antibodies, suggesting an autoimmune role in retinitis pathogenesis. **Methods:** We used immunohistochemistry techniques to screen human sera collected from North Eastern Kenya during a previous 2009 survey. Antibodies directed against S-arrestin were tested in four groups of human sera: 1) RVFV exposed with retinitis (N=5); 2) RVFV exposed without retinitis (N=5); 3) RVFV unexposed with retinitis (N=7); and 4) RVFV unexposed without retinitis (N=8). Donated human globes were paraffinized, sectioned into slides, exposed to human sera, and developed with a secondary antibody and colorimetric substrate. Staining of samples was scored by two blinded observers from 0 to 4; Scores above 2 were considered positive. **Results:** 3/5 of those with RVFV retinitis were positive, compared to 1/8 of normal controls (p=0.22). In addition, 1/5 of those with RVFV but without retinitis and 1/7 of those with retinitis but unexposed to RVFV were positive (p=0.52, p=0.22, respectively). RVFV retinitis cases compared to all control groups trended toward significance (p=0.07). **Conclusions:** In this small pilot study, anti-retinal antibody levels trended higher in RVFV exposed individuals with retinitis. Possible mechanisms to explain this finding include: 1) antibody directed against RVFV cross reacts with retina (molecular mimicry); and/or 2) RVFV infection leads to increased orbital permeability and exposure of normallly hidden antigen to pre-formed anti-retinal antibodes. Further investigation into the pathogenesis of RVFV retinitis could lead to improved prevention of the most common complication of human RVF disease.

## O1. Outbreak Investigation: Lab and Epi Response II

Wednesday, March 14
3:15 PM – 4:45 PM
Centennial I

**Different *Salmonella* Serotypes Vary Widely in Predominant Food Source and in Range of Implicated Food Commodities: Data from Outbreaks, United States, 1998–2008**

**B.R. Jackson**[1], P.M. Griffin[2], D. Cole[2], K. Walsh[2], S. Chai[2];
[1]Centers for Disease Control and Prevention, Atlanta, GA, USA, [2]Enteric Diseases Epidemiology Branch, CDC, Atlanta, GA, USA.

**Background**: *Salmonella* is the most common bacterial cause of foodborne disease outbreaks reported to the Foodborne Disease Outbreak Surveillance System (FDOSS), which collects foodborne outbreak reports investigated by state and local health departments. Outbreaks are one of only a few settings in

which the transmission route and implicated food can be determined. We examined food vehicles implicated in outbreaks caused by different *Salmonella* serotypes. **Methods**: We reviewed foodborne outbreaks of *Salmonella* illness with a single known serotype reported to FDOSS from 1998—2008. We included only outbreaks in which a single food commodity was implicated: foods and ingredients were assigned to one of 17 standardized, mutually-exclusive commodities (*e.g.*, eggs). For each serotype, the percentage of outbreaks attributed to each commodity and the standard deviation (SD) of these percentages were calculated to assess the diversity of implicated commodities. Serotypes in which the range of percentages varied widely, *i.e.*, with a high percentage of outbreaks attributed to a few commodities and a small percentage attributed to other commodities, had larger SDs than serotypes with percentages of outbreaks that were more similar for all implicated commodities. **Results**: Of the 1,193 outbreaks with a single *Salmonella* serotype reported, 403 (34%) were attributed to a single commodity. Eggs accounted for 65% of Enteritidis outbreaks. Poultry was the most commonly implicated commodity in outbreaks associated with serotypes Typhimurium (33% of 58 outbreaks), Newport (20% of 40), Heidelberg (50% of 24), Saintpaul (33% of 9), and Hadar (71% of 7). All outbreaks caused by serotypes Litchfield (n=5) and Poona (n=4) were attributed to the fruits-nuts commodity. Among serotypes that caused >5 outbreaks, Typhimurium, Newport, and Javiana had the widest range of commodity groups (SD <10) and Enteritidis, Heidelberg, and Hadar had the lowest (SD >15). **Conclusions**: Important differences exist in the predominant food commodities implicated in outbreaks from different *Salmonella* serotypes, indicating that particular serotypes have different reservoirs. Knowledge about these differences can help guide outbreak investigations and control measures.

**Multidrug Resistant *Salmonella* Hadar Infections Associated with Turkey Burger Consumption**

**A.L. Green**[1], R. Klos[2], J. Kirkpatrick[1], A. Douris[3], B. Miller[4];
[1]USDA-FSIS, Minneapolis, MN, USA, [2]Wisconsin Division of Public Health, Madison, WI, USA, [3]USDA-FSIS, Athens, GA, USA, [4]Minnesota Department of Agriculture, St Paul, MN, USA.

**Background:** Multidrug resistant (MDR) foodborne *Salmonella* is an ongoing concern in the public health community. *Salmonella* Hadar is a serotype commonly associated with poultry and has become the most common serotype in ground turkey. In 2008, *S.* Hadar accounted for 14.8% of *Salmonella* isolated from retail meats, an increase from an average of 6.6% from 2002 to 2006.[1] **Methods:** In mid-January 2011, the Minnesota Department of Agriculture's (MDA) retail food sampling program detected *S*. Hadar in a multi-ingredient turkey product produced by Company A, a corporation with nationwide distribution. Recent turkey consumption was reported by a single Minnesota case patient, however, the product had been purchased six months prior and frozen. Further traceback information was not available. Enhanced surveillance led the Wisconsin Department of Health Services to notify FSIS on February 11, 2011 of three confirmed clinical cases of *S.* Hadar from January. The patient isolates were indistinguishable by Pulsed-Field Gel Electrophoresis (PFGE) from the MDA sample and each other. Additionally, all three samples had similar antibiograms, each having resistance to five antimicrobials on the Wisconsin State Laboratory of Hygiene (WSLH) clinical test panel. **Results:** The WSLH determined that *Salmonella* isolated from leftover intact turkey burger product from a case-patient's home was indistinguishable from the outbreak strain by PFGE and antimicrobial susceptibility testing.The Food Safety Inspection Service (FSIS) worked with other states in the cluster to determine exposures and coordinated with FSIS microbiologists and data analysts to describe current and historic Company A establishment *Salmonella* results. This report describes the investigation that resulted in the first-ever FSIS raw poultry recall due to adulteration with MDR *Salmonella*. **Conclusions:** In a time of diminishing public health resources, tracing infections to a common source requires ongoing surveillance and coordination across public health agencies.

1. *National antimicrobial resistance monitoring system, Highlights of NARMS Retail 2008 report.*

http://www.fda.gov/downloads/AnimalVeterinary/SafetyHealth/AntimicrobialResistance/NationalAntimicrobialResistanceMonitoringSystem/UCM237120.pdf

**Ground Beef Recall in the United States Associated with Non-O157 Shiga Toxin-Producing *Escherichia coli***

**B.W. Kissler**[1], A. Robbins[2], M. Anand[3], D.C. Nicholas[3], J.S. Egan[3], K. Musser[3], H. Prince[4], H.E. Beaufait[4], S.D. Sears[2], E.K. Sawyer[5], J. Borda[6], D. Dietz[7], T.R. Collaro[8], P. Evans[9], S.A. Seys[10];
[1]USDA/FSIS, Atlanta, GA, USA, [2]Maine Department of Health and Human Services, Augusta, ME, USA, [3]New York State Department of Health, Albany, NY, USA, [4]Maine Department of Agriculture, Food & Rural Resources, Augusta, ME, USA, [5]New York State Department of Agriculture and Markets, Albany, NY, USA, [6]USDA/FSIS, Philadelphia, PA, USA, [7]USDA/FSIS, Pittsburgh, PA, USA, [8]USDA/FSIS, Waltham, MA, USA, [9]USDA/FSIS, Washington, DC, USA, [10]USDA/FSIS, Minneapolis, MN, USA.

**Background:** Non-O157 Shiga toxin-producing *Escherichia coli* (STEC) is estimated to account for approximately 112, 752 episodes of foodborne illness and 271 hospitalizations in the United States each year. Although non-O157 STEC illnesses associated with consumption of beef products have occurred worldwide, this is the first recall of ground beef due to non-O157 STEC contamination in the United States. **Methods:** Between July and August 2010, Maine and New York laboratories identified and submitted three unrelated clinical isolates of *E. coli* O26 with an indistinguishable PFGE pattern combination to PulseNet. Interviews conducted by state health officials revealed that all three case-patients either consumed or handled ground beef prior to illness onset. With the consent of case-patients, shopper card information was collected by states to assist with traceback investigations. **Results:** Using purchase records and invoices, traceback investigations conducted by Maine, New York, and the Food Safety and Inspection Service (FSIS) identified Establishment A as the only common supplier of ground beef products among the three case-patients. New York health officials collected leftover ground beef from a case-patient's home, and the product tested positive for *E. coli* O26 with an indistinguishable PFGE pattern combination with the outbreak strain. On August 28, 2010, Establishment A recalled approximately 8,500 pounds of ground beef products produced on June 11, 2010. **Conclusions:** Shopper card information increases the likelihood of determining the source of the outbreak. As with previous foodborne illness investigations, shopper card information identified precise ground beef purchase dates and traced products back to the implicated establishment. This is the first non-O157 STEC investigation resulting in a recall of ground beef in the United States. FSIS has been developing a policy on non-O157 STEC in certain raw beef products, and in September 2011, the Agency declared six additional serogroups of pathogenic *E. coli* as adulterants in non-intact raw beef.

**Bronchiolitis Outbreak by Respiratory Syncytial Virus in a Southwestern District of Bangladesh, 2010**

**F. Haque**[1,2], M.M. Husain[2], K.M. Ameen[2], R. Rahima[2], M.J. Hossain[1], A. Alamgir[2], M. Rahman[1], M. Rahman[2], S.P. Luby[1,3];
[1]International Centre for Diarrhoeal Diseases Research, Bangladesh (ICDDR,B), Dhaka, Bangladesh, [2]Institute of Epidemiology, Disease Control and Research (IEDCR), Dhaka, Bangladesh, [3]Centers for Disease Control and Prevention (CDC), Atlanta, GA, USA.

**Background:** During July 2010, newspapers reported a respiratory disease outbreak in the Meherpur District in southwestern Bangladesh resulting in admission of children to a secondary care hospital. We investigated this outbreak to determine the etiology and explore known risk factors. **Methods:** The hospital's physician diagnosed children <2 years with cough, fast breathing or shortness of breath, expiratory wheeze as acute bronchiolitis cases. We reviewed hospital records and listed cases admitted between 26 June and 26 July. We organized the cases according to villages where they lived. We defined a cluster as at least 3 hospitalized cases from the same village who lived within 15 minutes walking distance from each other. We surveyed the clinical profiles and exposures of cases hospitalized during

24-26 July. We visited cluster villages to survey cases who were discharged from the hospital. We collected nasal and throat swabs to test for respiratory viruses including respiratory syncytial virus (RSV) using a real time RT-PCR viral panel assay. **Results:** We identified 101 admitted acute bronchiolitis cases including 9 cases from 2 village clusters. Among these, 59/101 (58%) were admitted between 16-20 July. Among the 29 cases surveyed, the median age was 4 months; 65% were males. The siblings or older family members suffered similar symptoms in 31% of the affected households. We identified RSV in 91% (21/23) of the samples, of which 43% (9/21) had dual viral infection (RSV and influenza A H1N1, or adenovirus, or influenza B). The majority of cases (90%) were treated with antibiotics. There were no reported deaths. **Conclusions:** The sudden increase in admitted acute bronchiolitis cases, the median age of the affected cases and identification of RSV in 91% of the samples suggest that this was an outbreak of RSV bronchiolitis. RSV, the leading cause of potentially life-threatening lower acute respiratory tract infection in infants, likely contributes significantly to childhood morbidity and mortality in low-income settings. Given the paucity of public health interventions to prevent respiratory infections including RSV in low-income settings, further research for identifying preventive strategies should be prioritized. Factors that perpetuate antibiotic use in managing this viral syndrome should also be explored.

**Molecular and Epidemiological Tracking of a Norovirus Outbreak in a Hospital**

**D.J. Allen**[1], J. Harris[1], K.N. Ward[2], D. Brown[1], M. Iturriza-Gomara[1];
[1]Health Protection Agency, London, UK, [2]University College London, London, UK.

**Background:** Outbreaks of norovirus-associated gastroenteritis in healthcare settings occur during winter months, causing disruption services and resulting in significant financial cost to the NHS. **Methods:** We conducted a retrospective analysis of a protracted outbreak of norovirus-associated gastroenteritis occurring between November 2009 and April 2010 in one hospital. Clinical samples could be recovered from 60 patients inpatients during the outbreak period, all with laboratory-confirmed diagnosis of norovirus. **Results:** Traditional genotyping analysis revealed 4 norovirus genotypes circulating during the outbreak period: GII-1 (1.4%), GII-3 (4.3%), GII-4 (92.9%), and GII-6 (1.4%). Re-analysis of the GII-4 strains by sequencing of the hypervariable P2 domain of the capsid protein (VP1) revealed 12 genetic clusters among the GII-4 strains detected. Taken together, these represent 17 introductions of norovirus from the community into the hospital during the outbreak period. However, only 5/17 introductions (29%) resulted in onward transmission events in the hospital, and the majority of cases analysed (31/60, 52%) were associated with a single GII-4 genetic cluster (GII-4/Cluster 4). Onward transmission of the virus was detected on only two wards: the acute admissions unit and the elderly care ward. Symptomatic norovirus cases were detected on other wards, such as infectious diseases wards, intensive care units and oncology wards, but these were isolated incidents, with no transmission to other patients. **Conclusions:** This study shows that transmission of norovirus varies significantly. The ability of some norovirus stains to cause large outbreaks whilst others appear to be ‘self-contained’ may be associated with virus-specific factors and/or host factors. It is also possible that external factors relating to the organisational fabric of the built environment and/or healthcare practices could be important contributors. The tools used in this study can aid significantly in further studies to elucidate factors that facilitate transmission and control measures aimed at outbreak prevention.

**A Meningococcal Disease Outbreak among the Homeless Community in Los Angeles County, March 1 – July 31, 2011**

**R.H. Civen**, M. Adeyemo, V. Ngo, M. Kim, C. Tyson, H. Rivas, S. Chu, D. Dassey, L. Mascola;
Los Angeles County Public health Department, Los Angeles, CA, USA.

**Background:** National and Los Angeles County (LAC) surveillance data show declining meningococcal disease (MD) incidence in the past 15 years. Outbreaks of MD are rare, with the most recent LAC outbreak (OB) in 2001. In March 2011, the LAC Department of Public Health (DPH) became aware of 2 MD cases from a downtown homeless shelter within a 2 week period. We describe our investigation and control measures of a MD OB among the homeless community. **Methods:** LAC DPH distributed a health advisory to homeless shelters, local law enforcement agencies, infection preventionists, and emergency departments requesting all suspect meningitis cases be reported immediately. A confirmed MD case had a positive culture for *Neisseria meningitides* from a sterile site from March 1- July 31, 2011, the investigation period (IP). In additional to a standardized MD case history, a supplemental questionnaire was used to address risk factors such as homelessness, drug use, use of public transportation and other behaviors. All case isolates were analyzed by slide agglutination for serogroup, multiple- locus variable tandem repeat analysis (MLVA), and PFGE using the Nhe-1 enzyme by the CDC/ LAC DPH. Antibiotic prophylaxis was offered to close contacts of each case. **Results:** Twenty cases of MD were identified during the IP. Twelve (60%) were male; 9 (45%) were African American (AA), 8 (40%) smoked cigarettes, and 7 (30%) regularly used public transportation. PFGE and MLVA identified one OB of 4 cases of serogroup C MD during the first 3 weeks of the IP. OB cases included 3 AA and 1 Hispanic ranging in age from 37-61 years. At the time of illness onset, three OB cases were living in two homeless shelters; the fourth case was a public bus driver whose route had a stop at one of those shelters. Two additional clusters with 2 cases of serogroup C MD were also identified, each sharing geographic and race- ethnic commonalities. **Conclusions:** Our supplemental questionnaire collected additional MD risk factors and behaviors that permitted epidemiological linkage. PFGE and MLVA confirmed the identification of one outbreak and two additional clusters of 2 cases each. Preventive measures such as health alerts to local shelters and early prophylaxis dissemination to homeless shelter staff and close contacts may have contributed to controlling the outbreak among the homeless.

## O2. Prevention Challenges for Respiratory Diseases

Wednesday, March 14
3:15 PM – 4:45 PM
Centennial II

### Annual Laboratory Based Surveillance of Influenza and Other Respiratory Viruses in Georgia, Utilizing Molecular Based Assays

**M.M. Park**, R.T. Morales, H.N. Santiago-Alvarado,, R.Y. Modi, M.W. Berry, H.K. Ghuman, D.E. Little, E.A. Franko;
Georgia Public Health Laboratory, Decatur, GA, USA.

**Background:** Georgia Public Health Laboratory (GPHL) utilized a comprehensive test algorithm to monitor surveillance of 12 major respiratory viruses during the entire year from July 2010 to June 2011. The respiratory panel included all influenza viruses plus some of the major respiratory viruses. The purpose of this extended testing algorithm was for enhanced surveillance of all respiratory viruses through specimen submission by the participating Influenza-Like Illness (ILI) physician network and some hospitals that cared for patients in Metro Atlanta. **Method:** The procedures included real time reverse transcriptase polymerase chain reaction (rRT-PCR), provided by the Center for Disease Control and Prevention (CDC) for sub typing flu A and testing for flu B by rRT-PCR. Specimens that were negative by the above methodology for influenza were tested by xTAG™ RVP, using Luminex® microsphere technology. **Results:** A total of 1868 specimens were tested. The flu season started with flu type B in December and showed a positivity rate of 10.9%. Seasonal flu (A/H3N2) peaked in February and

dominated the season at a rate of 13.4% positivity, followed by rhinovirus at 9.1%, Pandemic (AH1/N1) flu at 7.2% and RSV at 2.4%. The surveillance data showed that most of the respiratory viruses prevailed during the same time frame as influenza viruses; however, HMPV peaked later in May and showed a higher rate of infection in Georgia than the national average rate. **Conclusions:** Testing all specimens, including the ones that were negative for influenza by the sensitive PCR methodology, provided accurate and timely results for patient management, including appropriate use of anti-viral treatment. In addition, the surveillance of 12 respiratory viruses, determined a higher positivity rate of HMPV than the U.S national rate in Georgia with a delayed peak season in May. The extensive testing algorithm used throughout the year provided important surveillance data, which helped epidemiologists in detecting the respiratory viral infections in the community and the clinicians in properly treating their patients.

**Prevalence of Respiratory Virus Infections among Hospitalized Children in a Rural Community in India**

**B.G. Pandey**[1], S. Rai[1], V. Gupta[1], A. Krishnan[1], W.M. Sullender[2], D.D. Erdman[3], R.B. Lal[3], S. Broor[1];
[1]All India Institute of Medical Sciences, New Delhi, India, [2]University of Alabama at Birmingham, Birmingham, AL, USA, [3]Centres for Disease Control and Prevention, Atlanta, GA, USA.

**Background:** The burden of disease due to respiratory viruses among children in India is not well defined, but is likely to be substantial as India accounts for 20% of global childhood deaths due to acute respiratory infections. This study was aimed to determine the prevalence of respiratory viruses in rural Indian children <5 years old hospitalized for medical illnesses. **Methods:** A total of 262 children (0-5 years of age) admitted at various private and public hospitals for acute medical care.were enrolled from August 2009 to September 2011 from an ongoing study for influenza disease burden in all persons living in the Ballabgarh Health and Demographic Surveillance System (HDSS). Clinical and epidemiologic information and nasopharyngeal swabs were collected. Real-time reverse transcription-polymerase chain reaction (rRT-PCR) assays for influenza A and B viruses (Flu), respiratory syncytial virus (RSV), parainfluenza viruses 1-3 (PIV), human metapneumovirus (hMPV), rhinovirus (RV), coronaviruses 229E, OC43, NL63 and HKU1 (CoV) and adenovirus (AdV) were carried out using CDC protocols. **Results:** Respiratory viruses were detected in 141 of 262 (54%) specimens, with a high prevalence of RSV (55; 21%), followed by RV (39; 15%) and influenza(17; 6%). All other viruses, including AdV, PIV, hMPV and CoV collectively accounted for <5% of detections. Most virus detections were from infants <1 yr of age (171; 59%) as compared with children >1-5 yr (43; 51%). RSV was the most common virus detected in infants (43/107; 24%), followed by RV (31/107; 17%) as compared with children >1-5 yr (14% and 10%, respectively). Interestingly, influenza was more often detected in children >1 yr of age (11% of 91) as compared to infants (4% of 171). Twenty-six of 262 (10%) specimens had more than one virus detected. **Conclusions:** This study provides evidence that respiratory viruses singly or in mixed infections are detected in >50% of medically attended hospitalized children from a rural community in India using sensitive detection methods like rRT-PCR. These findings will help guide efforts to reduce the disease burden due to viral ARIs in developing countries. *This study was supported in part by cooperative agreements U01 IP000206 from the Centers for Disease Control and Prevention, Atlanta, USA.*

**The Role of Facemasks and Hand Hygiene in the Prevention of Influenza Transmission in Households: Results From a Cluster Randomised Trial; Berlin, Germany, 2009–2011**

**U. Buchholz**, T. Suess, C. Remschmidt, S. Schink, B. Schweiger, A. Nitsche, K. Schroeder, J. Doellinger, J. Milde, W. Haas, I. Koehler, G. Krause;
Robert Koch-Institute, Berlin, Germany.

**Background:** Previous controlled studies on the effect of non-pharmaceutical interventions (NPI), namely use of facemasks and intensified hand hygiene, on the prevention of influenza transmission in households have not produced definitive results. We aimed to investigate acceptability, tolerability and efficacy of NPI to reduce influenza transmission in households. **Methods:** We conducted a cluster

randomized controlled trial during the pandemic season 2009/10 and the ensuing season 2010/11. We included households with an influenza positive index case and an absence of further respiratory illness within the preceding 14 days. Study arms were wearing a facemask and practicing intensified hand hygiene (MH group), wearing facemasks only (M group) and none of the two (control group). Outcome was RT-PCR confirmed influenza in a household contact. We used daily adherence protocols. **Results:** We recruited 84 households (30 control, 26 M and 28 MH households) with 82, 69 and 67 household contacts, respectively. In 2009/10 all 41 index cases had a (H1N1) 2009 infection, in 2010/11 24 had a (H1N1) 2009- and 20 had a B-infection. The total secondary attack rate was 16% (35/218). In intention-to-treat analysis there was no statistically significant effect of the M and MH interventions. When only households were analysed where intervention was implemented within 36 hours after symptom onset of the index case secondary infection in the pooled M and MH groups was significantly lower than in the control group (adjusted odds ratio 0.16, 95% CI, 0.03-0.92). In a per-protocol analysis odds for infection was significantly reduced among participants of the M group (adjusted odds ratio, 0.30, 95% CI, 0.10-0.94). With the exception of MH index cases in 2010/11 adherence was equally good for adults and children, contacts and index cases. **Conclusions:** Results suggest that household transmission of influenza can be reduced by the use of NPI, such as facemasks and intensified hand hygiene, when applied early and used diligently. Concerns about acceptability and tolerability of the interventions should not be a reason against their recommendation. *The study was registered with ClinicalTrials.gov (Identifier NCT00833885).*

**Association of Invasive Group A Streptococcal Infections with Soft Tissue Injury, United States (2005–2010)**

**K.-A. Toews**[1], S. Zansky[2], S. Petit[3], D. Aragon[4], J. Bareta[5], P.D. Kirley[6], R. Lynfield[7], M. Farley[8], M. Lindegren[9], P. Cieslak[10], R. Hollick[11], B. Beall[1], C. Van Beneden[1];
[1]Centers for Disease Control and Prevention, Atlanta, GA, USA, [2]New York State Department of Health, Albany, NY, USA, [3]Connecticut Department of Health, Hartford, CT, USA, [4]Colorado Department of Public Health and Environment, Denver, CO, USA, [5]New Mexico Department of Health, Santa Fe, NM, USA, [6]California Emerging Infections Program, Oakland, CA, USA, [7]Minnesota Department of Health, St. Paul, MN, USA, [8]Emory University School of Medicine and VAMC, Atlanta, GA, USA, [9]Vanderbilt Medical Center, Nashville, TN, USA, [10]Oregon Department of Health and Human Services, Portland, OR, USA, [11]Johns Hopkins Bloomberg School of Public Health, Baltimore, MD, USA.

**Background**: Invasive group A streptococcal (iGAS) infections can result in severe life-threatening conditions such as necrotizing fasciitis (NF) and streptococcal toxic shock syndrome (STSS). Trauma and breaks in the skin are known to increase the risk for iGAS infections. We describe the epidemiology of these risk factors as detected through population-based surveillance from 2005-2010. **Methods**: We reviewed data from CDC's Active Bacterial Core surveillance (ABCs), an active, population- and laboratory-based surveillance program ongoing in 10 US sites (2010 surveillance population: 32.1 million) from 2005-2010. An invasive case was defined as isolation of GAS from a normally sterile site with any syndrome or from wound culture if accompanied by NF or STSS in a surveillance area resident. We defined severe iGAS as a case with NF, STSS or both. Cases were reviewed for evidence of soft tissue injury (STI) in the week prior to the 1$^{st}$ positive GAS culture including: blunt or penetrating trauma, burns, surgical wounds, or varicella. **Results**: ABCs sites reported 7035 cases of iGAS [case fatality ratio (CFR) 12.2%] and 695 cases of severe iGAS (CFR 24.3%) from 2005-2010. STI was reported in 1361 cases of iGAS (19.3%) and 211 cases of severe iGAS (30.3%) (*P*<0.0001). Males more frequently had STI (21.7%) than females (16.6%) (*P*<0.0001). STI occurred in 16.6% of cases age <18 years, 26.3% of cases age 18-34 years, 22.1% of cases age 35-49 years and 17.3% of cases age ≥50 years. More specifically, blunt trauma was reported for 445 cases of iGAS (6.3%) and 81 cases of severe iGAS (11.6%) (*P*<0.0001); penetrating trauma for 524 iGAS cases (7.5%) and 89 severe iGAS cases (12.8%) (*P*<0.0001); and burns were reported

for 38 iGAS cases (0.54%) and 8 severe iGAS (1.2%) (*P*<0.05), respectively. Recent surgery (6.4%) and varicella (<1%) were not significantly higher among severe iGAS cases than among all iGAS cases. **Conclusions**: STI, particularly blunt and penetrating trauma, were associated with nearly a third of severe iGAS infections. Early provider recognition of these acute and easily recognizable risk factors may represent an opportunity for improvement in clinical outcomes and other sequelae of a subset of patients with invasive GAS disease.

**Influenza Virus Infections and Shedding among Transplant Patients, 2010–2011**

**A. Fry**[1], D. Wagner[1], F. Siveira[2], S. Husain[3], E. Dubberke[4], M. Schuser[5], R. Avery[6], P. Pappas[7], G. Storch[8], R. Buller[9], S. Bledsoe[10], S. Mason[10], L. Gubareva[1], T. Chiller[1], B. Park[1];
[1]Centers for Disease Control and Prevention, Atlanta, GA, USA, [2]U of Pittsburgh, Pittsburgh, PA, USA, [3]University Health System, Toronto, ON, Canada, [4]Wash Univ, St Louis, MO, USA, [5]UPenn, Philadelphia, PA, USA, [6]Cleveland Clinic, Cleveland, OH, USA, [7]Univ Alabama, Birmingham, AL, USA, [8]Wash U, Univ of St Louis, St Louis, MO, USA, [9]WashU, St Louis, MO, USA, [10]Wash U, St Louis, MO, USA.

**Background:** Prolonged shedding of influenza viruses and the development of influenza antiviral resistance during therapy has been described in patients who are immunosuppressed after solid organ (SOT) or stem cell transplantation (SCT). We characterize influenza-associated illness and describe virus shedding in transplant patients. **Methods:** At five transplantation centers, we enrolled patients who had laboratory-confirmed influenza infection. We collected serial nasopharyngeal or nasal swabs at presentation (day 0) and on days 3, 5, 7 and every 7 days until negative on two occasions and recorded demographic and clinical information and outcomes. Respiratory specimens were tested for influenza viruses with PCR. Virus isolation and antiviral resistance testing are pending. The interval from date of illness onset to the date of collection of the last PCR positive specimen was used to define duration of influenza virus detection. For 3 patients with illness onset date unknown, date of first diagnostic test was used. **Results:** From December 2010 to April 2011, we collected information on 35 patients with confirmed influenza infection, 30 were infected with influenza A and 5 with influenza B; 31 were enrolled into the shedding study. Median age was 54 years (interquartile range [IQR]: 38-60), 9 (26%) had SCT, and 26 (74%) SOT (20% lung, 17% kidney, 11% liver, and 11% heart). Prior to influenza virus infection, 94% were on immunosuppressive medications and 11% on chemotherapy . All initiated treatment with oseltamivir a median of 4 days after illness onset, 60% were hospitalized for their influenza illness (median length of stay 6 days (IQR: 3-12) and 6 patients were diagnosed $\geq$72 hours after hospital admission. Overall, 31% were admitted to an intensive care unit and 20% died. Among 4 SCT and 18 SOT patients with $\geq$3 serial swabs, the median duration of influenza virus detection was 15 (range 6-53) and 4.5 (range 0-21) days, respectively (p=0.28). Four transplant patients had virus detection for $\geq$14 days and 6 patients, all SOT patients, had no positive swabs after day 0. **Conclusions:** Among this cohort of immunosuppressed transplant patients, influenza infection was associated with severe illness. Identifying risk factors for severe illness and prolonged shedding may inform infection control and treatment recommendations.

**Geographic Variability in the Etiology of Influenza-Like Illness (ILI): The Acute Respiratory Infection Consortium, a Multi-state Longitudinal Study of ILI among Department of Defense (DoD) Beneficiaries**

**J. Arnold**[1], J. Maguire[2], T. Lalani[3], D. Sutter[4], M. Fairchok[5], M. Rajnik[6], A. Weintrob[6], M. Landrum[7], S. Echols[8], E. McDonough[9], P. Blair[9], G. Defang[10], A. Vancea[11], F. Seillier-Moiseiwitsch[11], M. Ridore[11], M. Ottolini[11], T. Burgess[11], M. Kortepeter[11], E. Millar[11];
[1]Naval Medical Center, San Diego, CA, USA, [2]Naval Medical Center Portsmouth, Portsmouth, VA, USA, [3]Infectious Disease Clinical Research Program, Portsmouth, VA, USA, [4]San Antonio Military Medical Center, San Antonio, TX, USA, [5]Infectious Disease Clinical Research Program, Ft Lewis, WA, USA, [6]Walter Reed Army Medical Center, Washington, DC, USA, [7]Infectious Disease Clinical Research Program, San

Antonio, TX, USA, [8]Infectious Disease Clinical Research Program, San Diego, CA, USA, [9]Naval Health Research Center, San Diego, CA, USA, [10]Naval Medical Research Center, Silver Spring, MD, USA, [11]Infectious Disease Clinical Research Program, Bethesda, MD, USA.
**Background:** The Acute Respiratory Infection Consortium (ARIC) is a Department of Defense (DoD) effort in multiple states to study the etiology, epidemiology, immunology, and clinical characteristics of influenza-like illness (ILI) among DoD beneficiaries, with the ultimate goal of reducing the impact of ILI in this population. **Methods:** A longitudinal study of beneficiaries at six US military hospitals in five regions of the U.S. began in 8/2009, and has enrolled 771 (372 adults) subjects to date. Locations included hospitals in Washington, DC; San Antonio, TX; San Diego, CA; Portsmouth, VA; and Tacoma, WA. Clinical, microbiological and immunological data are collected. Nasopharyngeal (NP) swabs collected on days 0, 3, 7 and 28 were tested by influenza- and adenovirus-specific PCR. Subsets were tested by viral culture, two multi-plex assays (Plex-ID® and Luminex®) and rhinovirus-specific PCR. Adult serum was tested by influenza HAI assay. **Results:** The median age was 14y (34d - 64y). 52 (7.4%) patients were hospitalized, with four (0.6%) ICU admissions. 614 (79.9%) patients reported receipt of any influenza vaccine in the past 5y, and 428 (55.5%) received H1N1 vaccine. NP specimens were positive for rhinovirus (18.6%), influenza (19.9%), and adenovirus (4.9%). Multiplex assay identified a pathogen in 39.3% (Plex-ID®) and 32.9% (Luminex®) of cases. Etiologies varied by geographic region of the country: influenza A (Tacoma: 14.9%; San Diego: 16.2%; San Antonio: 23.4%; Portsmouth: 8.8%; Washington DC: 18.4%). Of the influenza isolates, 77.5% were type A. Influenza B was more frequently identified in those <18y (79%), while the converse was true for influenza A (52% >18y). Among influenza A cases, more severe symptoms (e.g. chest pain, dyspnea, and dizziness) were reported among those with H1 vs. those with H3. 68 of 92 (73.9%) paired sera were positive (HAI titer ≥40) for A/H1N1pdm. **Conclusions:** An approach using multiplex nucleic acid-based detection assays increased the yield of likely causative agents, revealing geographic differences in the etiology of ILI. Young adults were significantly impacted by ILI. Further delineation of risk factors and targets for intervention is ongoing.

## O3. Populations at High Risk for Infectious Diseases

Wednesday, March 14
3:15 PM – 4:45 PM
Centennial III

### Pandemic Influenza A (H1N1) Infection and Progression to Hospitalization and Death: How Risk Is Influenced by Ethnicity and Socio-Economic Position

**M.G. Baker**[1], L. Telfar Barnard[1], M. McLeod[1], R. Harris[1], S. Huang[2], J. Stanley[1], J. Zhang[1], T. Love[1], C. McArthur[3], A. Jutel[4], T. Lanumata[1], G. Mercer[5], D. Bandaranayake[2];
[1]University of Otago, Wellington, New Zealand, [2]ESR, Wellington, New Zealand, [3]Department of Critical Care Medicine, Auckland District Health Board, Auckland, New Zealand, [4]University of Victoria, Wellington, New Zealand, [5]National Centre for Epidemiology and Population Health, Australian National University, Canberra, Australia.
**Background:** There were marked ethnic and socio-economic status (SES) inequalities in hospitalizations and deaths from pandemic influenza A(H1N1) infection in New Zealand. This study aimed to identify how these inequalities were mediated. **Methods:** We used data from multiple sources to measure and characterize the risk of pandemic influenza A(H1N1) infection and disease in New Zealand during the first pandemic wave in 2009: seroprevalence from a national serosurvey; general practitioner consultation rates for influenza like illness; and national data on hospitalizations, intensive care admissions, and deaths attributed to this infection. We calculated both the risk of infection, and the risk

of progression to serious outcomes, with adjusted rate ratios (aRR) for socio-demographic variables. **Results:** The H1N1 pandemic resulted in markedly higher rates of infection and poorer outcomes for specific socio-demographic populations. Infection rates were significantly higher for Pacific Peoples (aRR 1.49, 95%CI 1.07-2.07) relative to European/Other; and the more deprived SES quintile 2 (aRR 1.72, 95%CI 1.20-2.48) and 4 (aRR 1.69, 95%CI 1.19-2.41), relative to the low deprivation reference group (quintile 1). After adjusting for infection rates, hospitalization rates were significantly higher for Maori (aRR 2.44, 95%CI 2.07-2.88), Pacific Peoples (aRR 4.16, 95%CI 3.46-4.98), and the most deprived SES quintile 5 (aRR 1.37, 95%CI 1.08-1.73). Mortality risk was significantly higher for Pacific Peoples (aRR 3.28, 95%CI 1.44-7.49). By contrast, general practitioner consultation rates showed an inverse relationship to disease risk, with significantly lower rates for Maori and Pacific Peoples and those in more deprived quintiles after adjusting for infection rates. **Conclusions:** Reducing the impact of pandemic influenza depends on measures aimed at population groups with multiple disadvantages. These findings reinforce the importance of reducing SES inequality, improving access to primary care services, and identifying the specific factors contributing to an elevated risk for indigenous populations.

**Incidence and Etiology of Acute Lower Respiratory Infections in Hospitalized Children Under Five Years of Age in Rural Thailand**

**R. Hasan**[1,2], J. Rhodes[2], S. Thamthitiwat[2], P. Prapasiri[2], S. Naorat[2], S. Olsen[2,3], M. Chittaganpitch[4], S. Dejsirilert[4], P. Srisaengchai[2], P. Sawatwong[2], P. Jorakate[2], A. Kaewpwan[2], A. Fry[3], D. Erdman[5], L. Peruski[2,6], S. Chuananon[7], T. Amornintapichet[8], S. Maloney[2,6], H. Baggett[2,6];
[1]Centers for Disease Control and Prevention-Hubert Global Health Fellow, Nonthaburi, Thailand, [2]International Emerging Infections Program, Thailand Ministry of Public Health- US Centers for Disease Control and Prevention Collaboration, Nonthaburi, Thailand, [3]Influenza Division, CDC, Atlanta, GA, USA, [4]National Institute of Health, Nonthaburi, Thailand, [5]Division of Viral Diseases, CDC, Atlanta, GA, USA, [6]Division of Global Disease Detection and Emergency Response, CDC, Atlanta, GA, USA, [7]Nakhon Phanom Provincial Hospital, Nakhon Phanom, Thailand, [8]Crown Prince Hospital, Sa Kaeo, Thailand.

**Background**: Pneumonia is a leading cause of under-five mortality in Thailand and globally. Data on incidence, epidemiology, and etiology are needed to improve prevention and treatment strategies. **Methods**: We conducted active, hospital-based surveillance for acute lower respiratory infections (ALRI) in 2 rural Thai provinces with a population of 81,000 children aged <5 years. ALRI was defined as active infection (fever and/or abnormal WBC) plus signs or symptoms of lower respiratory illness. Chest radiographs and blood cultures were performed as clinically indicated. ALRI cases were systematically sampled to participate in an etiology study. Nasopharyngeal swab specimens were tested by PCR for 11 viruses, Mycoplasma, and Chlamydia, and during study year 1, serology was done. To account for changes in testing over time, an etiologic model was developed to estimate the relative contribution of viral and bacterial pathogens. **Results**: We identified 27,981 cases of hospitalized ALRI in children <5 years from 2005-2010. Chest radiographs were obtained in half (49%) of all cases, of which 75% had radiographically-confirmed pneumonia. Fifty-three (0.2%) children died in hospital. The overall ALRI incidence rate (IR) was 5,658 per 100,000 child-years (95% CI 5,592, 5,724), and was over twice as high in children aged 6-23 months vs. other age groups (IR Ratio (IRR) 2.45, 95% CI 2.40, 2.51). The IR for boys was 40% higher than for girls (IRR 1.39, 95% CI 1.35, 1.42). Increases in ALRI incidence occurred during February-March and there were larger increases in June-October. Based on our etiologic model, viruses were present in 63% of cases, most often respiratory syncytial virus (21%) and influenza viruses (10%). Bacterial pathogens, most commonly *Streptococcus pneumoniae,* contributed to 1.8% of cases; however, only 28% of cases had blood cultures. We estimated that 36% of ALRI cases were of unknown etiology. **Conclusions**: Our data underscore the importance of viral pathogens as etiologic agents and the large proportion of ALRI cases of unknown etiology. Influenza vaccination and interventions targeting common viral pathogens may decrease pediatric ALRI hospitalizations in Thailand. Improved

diagnostic approaches, especially for bacteria, are critical to allow more complete descriptions of childhood ALRI etiology.

**Gastroenteritis Deaths on the Rise in the United States: The Emerging Roles of *Clostridium difficile* and Norovirus**

**A.J. Hall**, A.T. Curns, L.C. McDonald, U.D. Parashar, B.A. Lopman;
Centers for Disease Control and Prevention, Atlanta, GA, USA.

**Background:** Globally, gastroenteritis is recognized as an important contributor to mortality among children, but population-based data on gastroenteritis deaths among adults and the contributions of specific pathogens have been limited. We describe the first comprehensive analysis of gastroenteritis deaths across all ages in the United States in over two decades and specifically estimate the contributions of *Clostridium difficile* and norovirus. **Methods:** Gastroenteritis-associated deaths in the United States during 1999-2007 were identified from the National Center for Health Statistics multiple cause-of-death mortality data. All deaths in which the underlying cause or any of the contributing causes listed gastroenteritis were included. Time-series regression models were used to identify cause-unspecified gastroenteritis deaths that were likely due to specific causes; seasonality of model residuals was analyzed to estimate norovirus-associated deaths. **Results:** Over the 8-year study period, all-cause gastroenteritis mortality more than doubled from 25/1,000,000 person-years to 57/1,000,000 person-years. Adults aged ≥65 years accounted for 83% of gastroenteritis deaths (258/1,000,000 person-years). *C. difficile* mortality increased 5-fold from 10/1,000,000 person-years in 1999-00 to 48/1,000,000 person-years in 2006-07. On average, norovirus contributed to nearly 800 deaths per year (3/1,000,000 person-years), though mortality rates surged by up to 50% during epidemic seasons associated with emergent viral strains. *C. difficile*-associated deaths were most frequent during March-May, while norovirus-associated deaths peaked during December-February. **Conclusions:** Gastroenteritis is an important cause of mortality in the United States, particularly in the elderly, and rates have increased over the past decade. While *C. difficile* has emerged as the leading contributor to gastroenteritis deaths, this study demonstrates for the first time that norovirus is likely the second leading infectious cause. The disease burden and distinct seasonal patterns associated with these pathogens should help guide appropriate clinical management strategies and vaccine development.

**Burden and Etiologies of Bacteremia from Population-based Surveillance in Rural and Urban Kenya**

**D.C. Burton**[1,2], G.M. Bigogo[2,1], B. Olack[2,1], A. Audi[2,1], L. Cosmas[2,1], B. Aura[2,1], M.K. Njenga[1,2], B.S. Fields[1,2], J.M. Montgomery[1,2], D.R. Feikin[1,2], R.F. Breiman[1,2];
[1]Centers for Disease Control and Prevention-Kenya, Nairobi and Kisumu, Kenya, [2]Kenya Medical Research Institute/CDC Research and Public Health Collaboration, Kisumu and Nairobi, Kenya.

**Background**: Characterizing bacteremia burden and etiologies in Africa is important to inform the selection and evaluation of preventive and therapeutic interventions. **Methods**: We estimated overall and pathogen-specific rates of bacteremia among >50,000 participants in population-based surveillance in rural western Kenya (Asembo, September 2006-August 2011) and an informal settlement in Nairobi, Kenya (Kibera, February 2007-July 2011). Blood culture specimens were obtained from participants presenting to a designated referral facility in each site with severe acute respiratory illness, jaundice, acute febrile illness, or any syndrome requiring admission. Data collected from regular (weekly or biweekly) home visits among surveillance households were used to adjust facility-based incidence rates (expressed as episodes per 100,000 person-years of observation [Pyo]), accounting for health-seeking to other area facilities for similar syndromes. **Results**: Adjusted incidence rates of bacteremia were highest in children ≤ 5 years in both sites (Asembo: 2,881 per 100,000 Pyo; Kibera: 2,155). Older children (5-17 years) had higher incidence of bacteremia than adults (≥18 years) in Kibera (older children: 1,330; adults: 623), whereas the opposite was true in Asembo (older children: 588; adults: 1,040). In Asembo,

the leading two pathogens in each age group were non-Typhi *Salmonella* (NTS) and *Streptococcus pneumoniae*, which together accounted for 72%-81% of isolates (NTS predominated in children, but *S. pneumoniae* predominated in adults). In Kibera, *Salmonella* serotype Typhi was the leading pathogen in each age group, accounting for 34% (<5 years) to 63% (5-17 years) of isolates, followed by *S. pneumoniae* and *Staphylococcus aureus* in older children and adults, and NTS and *S. aureus* in children <5 years. The number of NTS bacteremia cases increased over time in both sites (particularly among children <5 years) during most of the study period. **Conclusions**: Individuals in poor communities in Kenya experience high rates of bacteremia. Etiologies of bacteremia may differ substantially between rural and urban areas in Africa, and are dynamic over time, suggesting that prioritization of pathogen-specific interventions such as vaccinations should be informed by timely burden data for both settings.

**Emergence of Type 3 Adenovirus in US Military Trainees and in the Southern California General Population, 2011**

**A. Hawksworth**, M. Balansay, P. Kammerer, E. McDonough, P. Blair;
Naval Health Research Center, San Diego, CA, USA.

**Background:** Since production of the original live oral adenovirus (ADV) vaccine ceased and vaccination was discontinued in the late 1990s, increased respiratory illness rates and frequent outbreaks of ADV respiratory illness have occurred in US military basic training populations. Type 4 ADV has predominated among military trainees since the loss of vaccine, but Group B ADV (types 3, 7, 14, and 21) emerged in 2006 and have persisted at varying levels. A vaccine against types 4 and 7 ADV is expected to be reinstituted at all basic training centers in late 2011. ADV respiratory illness has been seen to a lesser extent in the general population and ADV vaccination has never been offered to the general public. **Methods:** Active laboratory based surveillance for febrile respiratory illness (FRI) was conducted in military and civilian populations from 2008 to 2011. A systematic sampling of FRI cases was conducted at 8 US military basic training centers, 3 DoD clinics in San Diego (non-military dependents), and community clinics near the US-Mexico border in California. PCR testing for ADV (including subtype) was performed on all specimens. **Results:** Type 4 ADV was the leading cause of FRI among military trainees during the period of observation with 54% positivity among 18,362 sampled cases. Types 3, 7, 14, and 21 were also present in varying proportions over time, with type 3 ADV showing increased prevalence in 2011. Among non-military populations, the burden of ADV was much lower, accounting for only 2.8% of FRI (31% type 3 ADV) among 3577 sampled cases in 2008-2010. Among 817 cases in 2011, however, prevalence of ADV increased to 8.6% (46% type 3 ADV). **Conclusions:** The emergence of type 3 ADV and the persistence of other group B serotypes in US military basic trainees may continue to cause ADV morbidity among recruits after vaccination is re-initiated. Increasing prevalence of ADV among FRI cases in the general population of southern California is noteworthy and may have public health implications. FRI etiology among these and other civilian populations should be followed to monitor the emergence and distribution of ADV serotypes.

**Surveillance for Dengue Infection among New York City Residents, 2010**

**B. Bregman**, S. Slavinski;
New York City Department of Health and Mental Hygiene, Long Island City, NY, USA.

**Background:** Dengue fever is the most prevalent mosquito-borne viral disease in the world, causing 50 million infections and 25,000 deaths annually. Dengue is not endemic for most of the United States. The New York City (NYC) Department of Health (DOHMH) began dengue surveillance in 2001 to monitor trends and look for potential autochthonous transmission. Between 2001 and 2009, a mean of 36 cases was reported annually (range 2-107). A global dengue epidemic in 2010 resulted in a significant increase in reported cases in NYC. NYC mosquito surveillance has shown that the primary vector of dengue, *Aedes aegypti,* is not found in NYC; however, *Aedes albopictus*, a less efficient vector, is present. We

describe surveillance findings from 2010 and implications for NYC. **Methods:** Dengue reports are received by the DOHMH via passive laboratory surveillance. Patients are interviewed using a standardized questionnaire to collect clinical information, identify the country of exposure, determine whether pre-travel healthcare advice was sought, and if mosquito bite protection was taken. Mosquito surveillance and control is conducted by the DOHMH from May through October. **Results:** In 2010, the DOHMH identified 144 cases of dengue, more than any previous year. Cases had a median age of 45 years, and 57% were female. All reported traveling to an endemic area during the incubation period. Patients most frequently reported travelling to the Caribbean (68%), Southeast Asia (13%) and South America (10%). The most commonly reported destinations were the Dominican Republic (n=46) and Puerto Rico (n=26). Visiting friends and family was the most common reason for travel (63%). Most cases recalled being bitten by mosquitoes (81%), but only 29% reported using insect repellent and 14% used a bed net. Only 10% sought medical advice before traveling. **Conclusions:** Most patients did not take precautions to prevent mosquito bites while in endemic areas. Effective strategies are needed to encourage travelers to adopt protective measures. While local transmission has not been identified in NYC, it is possible that infected persons might return to NYC while viremic. The ease and frequency of travel and presence of a competent vector put NYC residents at risk for potential autochthonous transmission, necessitating continued dengue surveillance in NYC.

## O4. Foodborne and Waterborne Infections

Wednesday, March 14
3:15 PM – 4:45 PM
Centennial IV

**Community Incidence of Pathogen-Specific Gastroenteritis: Reconstructing the Surveillance Pyramid for Seven Pathogens in Seven European Countries**

**A.H. Havelaar**[1,2], J.A. Haagsma[1,3], P.L. Geenen[1], L. Busani[4], S. Ethelberg[5], A. Fetsch[6], F. Hansdotter[7], A. Jansen[8,9], H. Korsgaard[10], S.J. O'Brien[11], G. Scavia[4], H. Spitznagel[8,6], P. Stefanoff[12];
[1]RIVM, Bilthoven, Netherlands, [2]Utrecht University, Utrecht, Netherlands, [3]Erasmus University, Rotterdam, Netherlands, [4]ISS, Rome, Italy, [5]SSI, Copenhagen, Denmark, [6]BfR, Berlin, Germany, [7]SMI, Stockholm, Sweden, [8]RKI, Berlin, Germany, [9]ECDC, Solna, Sweden, [10]Food-DTU, Soborg, Denmark, [11]University of Liverpool, South Wirral, UK, [12]PZH, Warzawa, Poland.

**Background:** We estimated the incidence of seven pathogens that cause gastroenteritis in seven European countries by building reconstruction models for a case of gastroenteritis in the general population to move through the different steps of the surveillance pyramid using data on the health care system in different countries in relation to pathogen characteristics, in particular the frequency of bloody diarrhea. **Methods**: We developed a stochastic simulation model, using several data sources. Reported cases were obtained from national surveillance systems. Data on health care usage (visiting a general practitioner, submitting a fecal specimen) were obtained by harmonized cross-sectional surveys of gastroenteritis prevalence in the community. Data on the frequency of bloody diarrhea were obtained from a literature review. Data on laboratory practices, test sensitivity and reporting of positive results were based on data complemented with expert opinion. **Results:** Germany, Denmark, Italy, the Netherlands, Poland, Sweden and the United Kingdom participated in the study. The degree of underreporting varied by pathogen and by country. Overall, multipliers were lowest for Germany and Sweden, followed by the United Kingdom, Denmark, the Netherlands, Italy and Poland. Across all countries, the incidence rate was highest for *Campylobacter* spp, followed by *Salmonella* spp. while incidence rates of *Yersinia* spp., *Shigella* spp. and Shiga toxin producing *Escherichia coli* O157 were lower

and of the same magnitude. Data on enteropathogenic *E. coli* and *Cryptosporidium* spp. were only available in few countries. The reconstruction model provides similar results as independent results from population based studies in the Netherlands and the UK for pathogens with a high proportion of bloody diarrhea, but appears to overestimate multipliers for pathogens with predominantly watery diarrhea. **Conclusions**: Reconstructing surveillance pyramids for enteric pathogens is an efficient method to estimate the population burden of gastro-intestinal infections, provided the availability of adequate empirical data. Its validity in relation to pathogens that cause predominantly watery diarrhea requires further investigation.

**Use of Population-based Surveillance to Evaluate the Burden of Shigellosis in an Urban Informal Settlement in Nairobi, Kenya**

**H.N. Njuguna**[1], L.O. Cosmas[1], J. Williamson[1], W. Mwiti[2], H. Omala[2], N. Wamola[2], J. Oundo[1], D. Feikin[1], E. Mintz[3], R. Breiman[1];
[1]Centers for Disease Control and Prevention (CDC), Nairobi, Kenya, [2]Kenya Medical Research Institute/Centers for Disease Control, Nairobi, Kenya, [3]Centers for Disease Control and Prevention (CDC), Atlanta, GA, USA.

**Background:** While *Shigella* is estimated to cause more than 160 million infections and 1 million deaths annually, limited incidence data are available from Africa, and especially from urban slums where sanitation and hygiene are sub-optimal. We investigated the epidemiology of shigellosis with ongoing population-based infectious disease surveillance among 28,500 people conducted by KEMRI-CDC in Kibera, a large urban slum in Nairobi, Kenya. **Methods:** From January 2007- Dec 2010, surveillance participants were visited in their homes every 2 weeks by community interviewers who collected data on illnesses among residents. Participants also had free access to a field clinic in the surveillance area where stool samples were collected from patients with passage of ≥3 loose stools within 24 hours or dysentery (≥1 stool with visible blood in previous 24 hours). We adjusted crude incidence rates considering: (a) those persons that met stool collection criteria during household visits but visited clinics other than the field clinic, and (b) persons who presented to the field clinic and met stool collection criteria, but were unable to provide a stool sample. **Results:** *Shigella* species were isolated from 431 (20%) of 2,210 stool specimens collected. Dysentery was strongly associated with shigellosis (OR 3.35 (95%CI 2.50-4.50)). The overall adjusted incidence rate was 1,289/100,000 person years with highest rates in adults 34-49 year old (2,988/100,000 person years) and children 12-23 months old (2,469/100,000 person years). The majority of isolates were *S. flexneri* (60%), followed by *S. dysenteriae* (non-type 1) (13%), *S. sonnei* (9%), and *S. boydii* (9%). Only 11% were susceptible to trimethoprim-sulfamethoxazole. Susceptibility to nalidixic acid and ciprofloxacin was high (97% and 99% respectively). **Conclusions:** The incidence of diarrheal disease due to *Shigella* in this urban slum is high, particularly among young children and middle-aged adults. Dysentery is a strong predictor. *S. flexneri* is the predominant isolate and susceptibility to quinolones remains high. Provision of safe drinking water and improvements of sanitation and hygiene within the rapidly expanding informal settlements are needed to reduce disease burden, and an effective shigella vaccine would likely provide substantial benefit in this setting.

**Foodborne Disease Outbreaks Associated with Food Imported into the United States, 2005–2010**

**L. Gould**, D. Morse, R.V. Tauxe;
Centers for Disease Control and Prevention, Atlanta, GA, USA.

**Introduction:** Nearly 16% of food consumed in the United States is imported from another country, including nearly 84% of fish and 32% of fruits and nuts imported into the U.S. **Methods:** We reviewed outbreaks reported to the CDC's Foodborne Disease Outbreak Surveillance System during 2005-2010 where the implicated food was reported as imported into the United States. Variables analyzed

included: number of outbreaks, implicated foods, country of origin, and etiologic agent. **Results:** During 2005-2010, 23 outbreaks with 1994 illnesses were reported where the implicated food was imported into the U.S., representing 0.4% of reported outbreaks. These outbreaks were reported in all years, with more outbreaks reported in 2009 and 2010 (n=6 and 8, respectively) than in 2005-2008. In eight (35%) outbreaks, exposure to the implicated food occurred in multiple states, compared with 1% of all outbreaks. Of the 22 outbreaks for which the implicated food could be assigned to a single commodity, the most common commodities were fish (6 outbreaks, 26%), vine-stalk vegetables (4, 17%), fruits (3, 13%), and dairy (3, 13%). The country of origin was reported for the foods in 21 outbreaks. Ten (46%) outbreaks were from foods imported from Latin America and 8 (38%) were from foods imported from Asia. Ten outbreaks (42%) were caused by *Salmonella*; the serotypes were Branderup, Carrau, Cubana, Litchfield, Newport, Paratyphi B, Rissen, Saintpaul, Typhi, and Wandsworth. Other etiologic agents included scombroid toxin/histamine (3 outbreaks, 13%), *Brucella,* norovirus, ciguatoxin (2 outbreaks, 8% each), and *Escherichia coli* O157, *Listeria, Vibrio,* and *Paragonimus* (1 outbreak, 4% each). **Conclusions:** An increased number of outbreaks due to imported foods were reported during the most recent years of surveillance. These findings underestimate the number of outbreaks due to imported foods as the origin of many foods causing outbreaks is not known or reported. The commodities associated with the most outbreaks were the same commodities most commonly imported into the U.S. Because imported foods are widely distributed, outbreaks caused by contaminated imported foods often involve multiple states. Efforts to improve the safety of the food supply should include gathering better data on the origin of implicated food items, including import status.

**An Ingredient-based Restaurant Cohort Study as Investigative Tool to Identify Sprouts as the Causative Vehicle of the Large Outbreak of O104:H4 Shiga toxin Producing *Escherichia coli;* Germany, 2011**

**U. Buchholz**, M. Boehmer, C. Remschmidt, H. Wilking, Y. Delere, M. an der Heiden, K. Stark, G. Krause; Robert Koch-Institute, Berlin, Germany.

**Background:** Within the large outbreak of Shiga-toxin-producing *Escherichia coli* (STEC) O104:H4 and hemolytic uraemic syndrome (HUS) that occurred in Germany in 2011 we conducted a focused restaurant ingredient-based cohort study. **Methods:** Several visitor groups with subsequent cases of gastroenteric disease had eaten in restaurant K between May 12 and May 16 (outbreak period). We contacted altogether ten cohorts of guest groups. A case was defined as an illness in a member of any of the cohorts with bloody diarrhea, self-reported laboratory confirmed STEC infection or HUS with onset of diarrhea within two weeks after visiting restaurant K. We interviewed the chef of the restaurant about the ingredients used in the menu items. We interviewed members of the different visitor groups and asked them about illness after the visit as well as menu items ordered. **Results:** The recipe-based study among cohorts visiting restaurant K included 10 groups with a total of 168 persons. Overall, 31 (18%) visitors developed bloody diarrhea or STEC-confirmed diarrhea. The median incubation period was 8 days (range 2, 14). Customers who were served sprouts had an attack rate of 27% (31/115) and were significantly more likely to become ill (relative risk, 14.2; 95% CI, 2.6-infinite). Sprout consumption explained 100% of cases. P-values of relative risks of all other raw food items were greater than 0.15. **Conclusions:** Our investigation identified sprouts as the outbreak vehicle. The restaurant ingredient-based study allowed for a bias-free investigation of risk factors while avoiding exposure misclassification due to ill-remembered food items.

**Outbreak of Enteroaggregative Shiga Toxin-Producing *Escherichia coli* O104:H4 in Europe and North America Associated with Sprout Consumption: Investigation of US Cases**

**A. Mba-Jonas**[1], T.-A. Nguyen[1], R. Mody[1], C. Bailey[1], C. Bopp[1], P. Gerner-Smidt[1], K. Joyce[1], N. Strockbine[1], R.V. Tauxe[1], C. Foley[2], E. Harvey[3], S.A. Bidol[4], T. Henderson[4], R. Njord[5], T. DeSalvo[6], T. Haupt[6], S.A.

Bosch[1];
[1]Centers For Disease Control and Prevention, Atlanta, GA, USA, [2]Arizona Department of Health Services, Phoenix, AZ, USA, [3]Massachusetts Department of Public Health, Boston, MA, USA, [4]Michigan Department of Community Health, Lansing, MI, USA, [5]North Carolina Department of Health and Human Services, Raleigh, NC, USA, [6]Wisconsin Division of Public Health, Madison, WI, USA.
**Background:** Enteroaggregative Shiga toxin-producing *E. coli* (STEC) O104:H4 is a novel foodborne pathogen. On May 25, 2011, CDC was notified of a rise in STEC O104:H4 infections in Germany. Illness onsets began in early May. The clinical manifestations and disease transmission dynamics have not been fully characterized for this pathogen, which contains a rare combination of virulence factors. We initiated surveillance for US cases of STEC O104:H4 infection to identify outbreak-associated cases, to assess travel and food histories of case-patients, and to obtain information about the clinical course. **Methods:** Surveillance for US cases began on May 26, 2011 and included 1) active data gathering from potential cases through direct contact with states and announcements on national public health listserves, and 2) case-finding activities based on suspected clusters. Suspect cases were defined as either hemolytic uremic syndrome (HUS) or Shiga toxin-positive diarrheal illness in persons with travel to or from Germany since April 1, 2011, or illness onset either during travel to, or ≤3 weeks after arriving in the US from Germany. Cases were confirmed when STEC O104:H4 was isolated from a clinical specimen. To obtain detailed food and travel histories, open-ended interviews were conducted with cases or, if they were too ill, their travel companions. Strains were characterized at CDC. **Results:** Between May 26 - June 16, six confirmed cases were identified in Arizona (1), Massachusetts (1), Michigan (2), North Carolina (1), and Wisconsin (1). All strains produced Stx2a and were aggR+ and eae-. Patients' ages ranged from 38-72 years, with a median of 52; 33% were female. Five traveled to Germany and one developed diarrhea after close contact with a case patient who traveled. No patients recalled consumption of sprouts, the food vehicle ultimately implicated in the outbreak. All patients had bloody diarrhea, four (66%) case-patients developed HUS and required dialysis as well as ventilator support, and one case-patient died. **Conclusions:** The severity of illness and potential for person-to-person transmission are cause for concern for future outbreaks of similar novel STEC strains. Adherence to established guidelines for STEC testing and characterization is needed to detect such pathogens routinely in the future.

**Guillain-Barre Syndrome Incidence Declines Following Successful Countrywide Control of Campylobacteriosis**

**M.G. Baker**[1], A. Kvalsvig[1], J. Zhang[1], R. Lake[2], A. Sears[1], N. Wilson[1];
[1]University of Otago, Wellington, New Zealand, [2]ESR, Christchurch, New Zealand.
**Background:** *Campylobacter* infection is a known precipitating event for Guillain-Barre syndrome (GBS). We aimed to assess the relationship between hospitalisation for campylobacteriosis and subsequent risk of GBS and test the hypothesis that GBS incidence had responded to a marked rise and then decline in campylobacteriosis rates in New Zealand (NZ). **Methods:** This study used hospitalisations records for GBS, campylobacteriosis, and selected other conditions; mortality data for GBS; and campylobacteriosis notification data for the years 1988-2010. Hospitalisation data were filtered to remove readmissions and examined for evidence of an association between the incidence of campylobacteriosis and GBS. **Results:** We identified 2056 first admissions for GBS, an average rate of 2.32/100000 population/year. Over the 1989-2008 period there were 56 deaths recorded for GBS, a case fatality proportion of 3.0%. Annual GBS hospitalisations were closely correlated with campylobacteriosis notifications (p=0.002). Those hospitalized for campylobacteriosis had a significantly increased risk of GBS hospitalization during the following month (rate ratio 319.0, 95%CI 201.5-506.4). Successful interventions to lower *Campylobacter* contamination of fresh poultry meat were followed by a 52% decline in campylobacteriosis notifications, with GBS hospitalisations declining by 13% (RR 0.87, 95%CI 0.81-0.93) in the 3-year post-intervention

period. The sub-population of GBS cases associated with campylobacteriosis had a similar age (median 54 years) to the GBS population in general (median 52.5 years). **Conclusions:** This study adds further evidence that campylobacteriosis is an important cause of GBS. Furthermore, these data suggest that regulatory measures to prevent food-borne campylobacteriosis have the health and economic co-benefit of preventing GBS.

## O5. Laboratory Support: Surveillance and Monitoring Infections

Wednesday, March 14
3:15 PM – 4:45 PM
Regency VI

**Evidence of Dobrava and Puumala Viruses Circulation in Humans and Rodents in West Ukraine, 2004-2006**

**R. Younan**;
US–NAMRU3, Cairo, Egypt.

**Background:** Hemorrhagic fever with renal syndrome (HFRS) viruses occur throughout Europe and Asia. The most common HFRS subtypes are Hantaan (HTNV), Dobrava (DOBV), Seoul (SEOV) and Puumala (PUUV). Each HFRS virus is associated with a specific rodent reservoir species. This study aimed to 1) investigate Hantaviruses (HTA) associated with acute febrile illness (AFI) in humans and 2) detect HTA subtype in rodents from West Ukraine. **Methods:** Serum samples collected from AFI cases in Zakarpathia Oblast region were tested by commercial and home-made ELISA (USAMRIID) to detect IgM to DOBV/HTNV and PUUV. Two subsets (n=12, n=15) of IgM positive samples were confirmed by plaque reduction neutralization assay (PRNT) for HTNV/ DOBV or PUUV respectively. RNA was extracted from the serum samples and tested by real time RT-PCR for PUUV. A nested RT-PCR (nRT-PCR) using primers directed to M segment/G1 (MM-G1) coding region of Hantaviruses associated with rodents (HTN, DOBV and SEOV) was also performed. PCR product was purified from gel slices, sequenced, edited and analyzed. *Apodemus agrarius rodents* (n=30) were collected from forests in the same area. Tissue (kidneys, lungs, spleen and brain) samples were pooled from individual animals, placed on RNAlater™ and homogenized. RNA was extracted and tested as described for the human samples.
**Results:** PRNT showed that 67% of HTNV/DOBV IgM positive samples had neutralizing antibodies against DOBV, while all PUUV IgM positive samples had neutralizing antibodies against PUUV. PUUV and DOBV RNA were also detected in acute sera. Sequence analysis of the DOBV RNA showed high similarity to DOBV-Belgrade virus from Slovakia (95.9 to 97.8 % nucleotide sequence identity). Ten rodents (33%) were positive by nRT-PCR for DOBV. Sequence analysis revealed a 96% identity to DOB-Belgrade virus from Slovakia.Human case and all rodent sequences for DOBV clustered together in a monophyletic group with average nucleotide identity of 98.8%. **Conclusions:** This is the first laboratory confirmation of PUUV and DOBV as etiologic agents of HFRS in West Ukraine. In addition, results of rodent testing show that DOBV circulates in *A. agrarius*. Sequence comparison between viruses isolated from the human and rodents implicates A. agrarius as an associated rodent reservoir for HFRS caused by DOBV in West Ukraine.

**Pathogens Detected during Enhanced Surveillance for Fatal Dengue-like Acute Febrile Illness—Puerto Rico, 2010–2011**

**D.M. Blau**[1], A. Rivera[2], I. Rivera[3], J. Bhatnagar[1], E. Hunsperger[2], J. Munoz-Jordan[2], C. Drew[1], C. Paddock[1], W.-J. Shieh[1], K.M. Tomashek[2], S.R. Zaki[1];

[1]Centers For Disease Control and Prevention, Atlanta, GA, USA, [2]Centers For Disease Control and Prevention, San Juan, PR, USA, [3]Institute of Forensics Sciences, San Juan, PR, USA.
**Background:** Enhanced surveillance for deaths following an acute febrile illness (AFI) was initiated in Puerto Rico in 2010 to improve detection of dengue-related deaths. In collaboration with Centers for Disease Control and Prevention (CDC) Dengue Branch, the Puerto Rico Institute of Forensics Sciences systematically collected autopsy information and tissues from all fatal AFI cases detected. **Methods:** Autopsy specimens were submitted to the CDC's Infectious Diseases Pathology Branch (IDPB) from March 2010- March 2011. All cases were tested for dengue virus (DENV) RNA in tissues by RT-PCR. Serum samples, when available, were tested by CDC Dengue Branch by DENV RT-PCR and anti-DENV IgM and IgG enzyme-linked immunosorbent assay (ELISA). Evaluation of tissues for other infectious etiologies included examination of the histopathology, special histochemical stains, and molecular and immunohistochemical assays. **Results:** Tissue specimens from 85 cases were submitted for evaluation; 79 (93%) also had serum submitted. Of all cases, 53 (62%) were male and the median age was 38 years, range of 2 months to 84 years. The median duration from onset of illness to death was 7 days. An etiologic agent was identified in 48 (56%) of these AFI cases, including bacterial pathogens (29), viral (20) and fungal (1). Of the bacterial cases 14 were found to have evidence of *Leptospira* and of the viral cases 19 were positive for DENV and in two of these there was also evidence of a bacterial coinfection. Of the 19 cases that had DENV RNA identified in their tissue, 12 of 18 (67%) with serum available had positive RT-PCR or IgM. Predominant histopathology findings were dependant on infectious agent identified. **Conclusions:** Evaluation of autopsy tissues from patients with a suspect infectious process can provide an etiologic diagnosis that was not available from routine clinical testing. The etiologic agents identified in this enhanced surveillance included reportable agents, vaccine-preventable diseases (e.g streptococcal, meningococcal) and zoonotic agents. These findings underscore not only the importance of enhanced surveillance but additionally the value of postmortem examination and autopsies in the diagnosis of fatal infectious diseases.

**CaliciNet: A Novel Norovirus Outbreak Surveillance Network in the United States**

**J. Vinjé**[1], E. Vega[1], N. Gregoricus[1], H. Shirley[2], D. Lee[2], L. Barclay[1];
[1]Centers for Disease Control and Prevention, Atlanta, GA, USA, [2]AREF, Atlanta, GA, USA.
**Background:** Human noroviruses are responsible for 58% of all foodborne illness with a known etiology and for 70-95% of all viral gastroenteritis outbreaks in the US. To increase the quality of national norovirus surveillance, the Centers for Disease Control and Prevention (CDC) has developed and implemented an electronic norovirus outbreak surveillance network (CaliciNet) with state and local public health laboratories. **Methods:** Twenty-four certified state and city public health laboratories representing 59% of the US population submitted norovirus outbreak and sequence data to CaliciNet from March 2009 - July 2011. **Results:** Since CaliciNet went live in March 2009, 1329 outbreaks were uploaded to CaliciNet, of which 143 (11%) were caused by foodborne transmission. In October 2009, a novel GII.4 strain (GII.4 New Orleans) as well as a novel GII.12 strain emerged. GII.4 New Orleans caused 333 (62%) and 455 (73%) of the outbreaks in 2009-2010 and 2010-2011, respectively. The widespread presence of GII.4 New Orleans across the US coupled with the decreasing prevalence of the GII.4 Minerva variant, which has been the major cause of outbreaks from 2006-2009, suggests gradual strain displacement. GI strains caused 9.7% of all outbreaks and the novel GII.12 strain caused 15% of the outbreaks in the 2009-2010 norovirus season. **Conclusions:** We developed a novel norovirus outbreak surveillance network in the U.S. that allows standardized genotyping of norovirus strains, comparison of sequences from outbreaks that have a common source, as well as identification of emerging strains in real-time. CaliciNet will continue to expand over the next couple of years and will be an invaluable resource to determine the most important strains causing norovirus gastroenteritis in different populations against which a future vaccine should protect.

**World Health Organization Global Surveillance Network for Invasive Bacterial Vaccine Preventable Diseases: Challenges, Opportunities, and the Role of the Laboratory**

**F. Serhan**[1], C. Talarico[2], S. Schwartz[2], M. Carvalho[2], L.W. Mayer[2], F. Pimenta[2], C. Van Beneden[2], M. Agocs[1], R. Hajjeh[2];
[1]World Health Organization, Geneva, Switzerland, [2]Centers for Disease Control and Prevention, Atlanta, GA, USA.

**Background:** Developing countries began introducing new conjugate vaccines for prevention of meningitis and pneumonia at a rapid rate over the last few years. Monitoring the impact of these new vaccines is crucial to support immunizations programs long term and to identify changes in disease trends and causative organisms. To achieve these goals, in 2009 WHO initiated the Invasive Bacterial Vaccine-Preventable Diseases (IB-VPD) network, a multi-tiered, laboratory-based surveillance network that monitors disease caused by *Streptococcus pneumoniae, Haemophilus influenzae* type b, and *Neisseria meningitidis.* This network is the first global approach to laboratory surveillance for IB-VPD organisms. **Methods:** WHO global and regional coordinators monitor laboratory performance and coordinate activities to build bacteriology capacity in countries. CDC's Division of Bacterial Diseases serves as the main Global Reference Laboratory (GRL) for this network; together the GRL and WHO provide technical epidemiologic and laboratory support to the WHO regional offices and reference laboratories (RRLs), which are directly responsible for supporting surveillance in the countries. **Results:** WHO and the GRL have trained RRLs to build their capacity for detection, isolation, and characterization of these organisms, as well as quality assurance and control and laboratory management. The GRL has held training workshops in 4 of the 6 WHO regions (training a total of 42 laboratorians from 13 countries) and maintains regular communication with the RRLs for guidance and troubleshooting. Laboratory-based surveillance for IB-VPD has been challenging in all settings due to the fragility and special microbiologic characteristics of the bacteria. In developing countries, additional challenges have been encountered due to inadequate infrastructure and resources (e.g. lack of continuous power supply, limited supply chain for perishable reagents), as well as deficiencies in the surveillance systems (e.g., inadequate specimen handling and transport, inconsistent case identification). **Conclusions:** Addressing challenges associated with global IB-VPD surveillance is crucial for improving bacterial surveillance capacity and for generating high quality data to support vaccine introduction and monitoring worldwide.

**Incident Hepatitis E in the United States**

**J. Drobeniuc**, T. Greene-Montfort, T. Hayden, L. Ganova-Raeva, T. Le, C.-G. Teo, S. Kamili;
Centers for Disease Control and Prevention, Atlanta, GA, USA.

**Background:** The epidemiology of hepatitis E in the US is largely unknown. To enable its description, we collated and analyzed virological data acquired from sera referred to CDC by health-care providers and by public-health and commercial laboratories, together with accompanying demographic, clinical and travel-history data of patients from whom the sera originated. **Methods:** Sera referred from June 2005 through August 2011 were enrolled. Samples that tested positive for IgM anti-HEV were considered to originate from patients who met the case definition for incident hepatitis E. For HEV genotyping, nucleotide-sequencing of RT-PCR products amplified from HEV RNA was conducted. **Results:** Of 1188 sera tested, IgM anti-HEV was detected in 286 (24%). Analysis of data from 33 cases has been completed; 20 cases (60%) presented with acute jaundice, and 7 (21%) were organ-transplant recipients. Their median age was 39 y (range, 11 to 67 y) and 59% were male; 18 (56%) were Caucasian; 8 (25%) were South Asian; 4 (13%) were Hispanic and 1 (3%) was African-American. Seven cases were referred from Texas, 4 each in California, Illinois and New York, 2 each in Florida, Maryland, and South Dakota, and 1 each in Alabama, Connecticut, Delaware, Maine, Massachusetts, Pennsylvania, Virginia and Wisconsin. Travel outside the US in the 2 months before the illness was recorded for 28 cases. Of 17

(61%) with no recent history of travel, 5 were infected by HEV genotype 3. Of the remaining 11 cases with a history of travel, 10 reported travel to the Indian subcontinent, 1 of whom was infected by HEV genotype 1, and 1 case, who reported travel to China, was infected by genotype 4. **Conclusions:** Hepatitis E in United States was more common than previously thought. Incident disease tended to be associated with people who were middle-aged and male. Although most patients had not recently traveled, and therefore likely to have contracted disease indigenously, a significant proportion traveled to countries that are highly HEV-endemic, so imported the disease home.

**Department of Defense (DoD) Combined Influenza Sequence Analysis at the United States Air Force School of Aeropace Medicine: A New, Coordinated Initiative**

**B.C. Connors**[1], V.H. MacIntosh[1], J.P. Smith[1], J.L. Garner[2], A.C. Guerrero[3], P.A. Sjoberg[1];
[1]US Air Force School of Aerospace Medicine, Wright-Patterson Air Force Base, OH, USA, [2]Bio Rad, San Antonio, TX, USA, [3]US Army Medical Dept. Center & School, Fort Sam Houston, TX, USA.

**Background:** USAFSAM provides a global lab-based influenza surveillance system for DoD beneficiaries and related populations. This system is a part of a larger network of DoD laboratories with support, guidance and coordination from the Armed Forces Surveillance Center, Division of GEIS Operations (formerly DoD-GEIS). DoD contributes mid-season sequence analysis and surveillance data to the Vaccine and Related Biologics Product Advisory Committee (VRBPAC) of the Food and Drug Administration (FDA). DoD's policy is to vaccinate all active duty forces for influenza annually; USAFSAM monitors vaccine effectiveness annually. **Methods:** A working group of the DoD influenza network convened April 2010 in San Antonio. The group agreed to include to submit sequences of viruses collected during the 2010-2011 influenza seasonal with agreed upon metadata. The first 1200 base pairs (bp) of HA1 subunit of hemagglutinin, delivered in standard FASTA file, were to be submitted by January 2011. Sequences would have no gaps or ambiguities. We requested submissions from the network to include 4 key labs and some medical centers within the military health system. Sequences were to be characterized, combined and compared. These data were combined with demographics, service and vaccination status. Phylogenetic trees were created for A/2009 H1N, A/ H3N2, and B. **Results:** Over 500 sequences were included in the analysis from USAFSAM, the 4 labs (2 Army 2 Navy), and some medical centers. Characterized results were combined in phylogenetic trees as described above and presented at the 2011 VRBPAC meeting [Russell, K.L., Burke, R., Influenza Surveillance and Vaccine Effectiveness in the DoD, 25 Feb 2011, Bethesda, MD, available on FDA VRPAC website]. Homologies, compared to 2010-11 vaccine component strains, were: A/2009 H1N1 range 97.0% - 100%; A/H3N2 96.5% - 99.4%; B Victoria 98.2% - 100%. Vaccinated cases were noted on the trees to visualize mutations and vaccine breakthrough associations. **Conclusions:** DoD networked influenza surveillance laboratories collaborated to improve sequence analysis methods and standardize data sharing using a common sequence format for HA1 subunit to improve surveillance data shared with CDC and FDA. This work focused on vaccine considerations for the next year's vaccine and was incorporated by VRBPAC.

## O6. Late-Breakers III

Wednesday, March 13
3:15 PM – 4:45 PM
Regency VII

**Immunogenicity of One Dose of Vero Cell Culture-derived Japanese Encephalitis (JE) Vaccine in Adults Previously Vaccinated with Mouse Brain-derived JE Vaccine**

**T. Woolpert**[1], J.E. Staples[2], D.J. Faix[1], R.J. Nett[2], O. Kosoy[2], B.J. Biggerstaff[2], B.W. Johnson[2], M. Sracic[3], M. Fischer[2];
[1]Naval Health Research Center, San Diego, CA, USA, [2]Division of Vector-Borne Diseases, Centers for Disease Control and Prevention, Fort Collins, CO, USA, [3]13th Marine Expeditionary Unit, United States Marine Corps, Camp Pendleton, CA, USA.

**Background:** In 2009, new inactivated Vero cell culture-derived JE vaccine (JE-VC) was licensed in the US. An inactivated mouse brain-derived JE vaccine (JE-MB) has been licensed in the US since 1992. However, JE-MB is no longer being produced and all remaining doses expired in May 2011. There are no data on the use of JE-VC as a booster in individuals who previously received JE-MB. **Methods:** Military personnel who received ≥3 doses of JE-MB or were JE vaccine-naïve were vaccinated with 2 doses of JE-VC on days 0 and 28. Serum neutralizing antibodies were measured pre-vaccination and 28 days after each dose. Noninferiority was evaluated for seroprotection rate and geometric mean titer (GMT) between previously vaccinated participants post-dose 1 and vaccine-naïve participants post-dose 2. Solicited side effects reported for the 4 days following each JE-VC dose were compared. **Results:** Fifty-three previously vaccinated and 70 JE vaccine-naïve participants were enrolled. Previously vaccinated participants had significantly higher GMTs pre-vaccination (13 vs. 5), post-dose 1 (315 vs. 11), and post-dose 2 (414 vs. 79) (P<0.01 for each). Seroprotection rates among previously vaccinated participants post-dose 1 (44/44, 100%) were noninferior to those achieved in previously naïve participants post-dose 2 (53/57, 93%) (Difference 7%; 95% CI -1%-17%). The GMT among previously vaccinated participants post-dose 1 (GMT 315; 95% CI 191-520) also was noninferior to that of previously naïve participants post-dose 2 (GMT 79; 95% CI 54-114) (GMT ratio 3.99; 95% CI 2.16-7.36). The overall frequency, duration, and severity of adverse events did not differ between the groups. However, following dose 2 of JE-VC, previously vaccinated subjects reported a higher incidence of injection site pain (41% vs. 15%), redness (13% vs. 2%), and swelling (15% vs. 0%) than subjects who had not received JE-MB (P<0.05 for each). **Conclusions:** Among military personnel previously vaccinated with ≥3 doses of JE-MB, a single dose of JE-VC resulted in a noninferior neutralizing antibody response compared to a 2-dose primary series of JE-VC in previously unvaccinated personnel. Additional studies are needed to confirm these findings in other populations and determine the duration of protection following a single dose of JE-VC in prior recipients of JE-MB.

**Syndromic Surveillance in the Pacific: Evaluation of the First Year**

**B.J. Paterson**[1,2], D.N. Durrheim[1,3], J. Kool[4], B. Pavlin[5];
[1]University of Newcastle, Wallsend, Australia, [2]Hunter Medical Research Institute, Newcastle, Australia, [3]Hunter New England Population Health, Wallsend, Australia, [4]World Health Organization, Division of Pacific Technical Support, Suva, Fiji, [5]World Health Organization, Pohnpei, Federated States of Micronesia.

**Background:** Pacific Island Countries and Territories (PICTs) recently implemented a standardised, simple syndromic surveillance system to detect and respond to outbreaks of infectious diseases. Based on the early detection and reporting of four core syndromes (influenza-like-illness, diarrhoea, prolonged fever, and acute fever with rash) and the immediate reporting of unusual events, the system uses

standardised case definitions and processes rather than focussing on a technology platform used to collect or analyse the data. A formative evaluation was conducted in the first year to explore determinants of successful implementation. **Methods:** Data were collected between May-September 2011, using: semi-structured key informant interviews with selected PICTs; observational techniques including field inspections of raw data and data collection methods; analysis of syndromic data reported to WHO by participating PICTs; and reporting of alerts, outbreaks and outbreaks during the first year were compared pre and post implementation. Reliability of identified themes was confirmed with public health officials from additional PICTs. System components and attributes were identified and timeliness, compliance and proportion of sites reporting were analysed. **Results:** Forty three informants from five PICTs were interviewed. The number of PICTs participating increased from 6 to 20, of 22 PICTs, in the first year. The reporting of regional alerts, outbreaks and outbreak updates substantially increased from 29 in 2010 to 224 in 2011. Determinants of system success were: simplicity of the system; support from all levels of government; clearly defined roles and responsibilities; feedback to those who collect the data; harmonisation of case definitions; integration of data collection tools into existing health information systems; and availability of clinical and supportive epidemiological advice. **Conclusions:** There is a high level of system acceptance by PICTs and overwhelming agreement that the system is effectively acting as an early warning system, with data completeness improving. Simplified syndromic surveillance should be a priority in countries with limited public health system or technological capacity.

**A Novel Influenza A Virus from Bats**

**S. Tong**[1], Yan Li, Pierre Rivailler, Christina Conrardy, Li-Mei Chen, D.A. Alvarez Castillo[2], Sergio Recuenco, James A.Ellison, Charles T. Davis, Ian A. York, Amy S. Turmelle, D. Moran[2], Mang Shi, Ying Tao, M.R. Weil[3], Kevin Tang, Lori A. Rowe, Scott Sammons, Xiyan Xu, Michael Frace, Kim A. Lindblade, Nancy J. Cox, Larry J. Anderson, Charles Rupprecht, Ruben O. Donis;

[1]Centers for Disease Control and Prevention, Atlanta, GA, USA, [2]Universidad del Valle de Guatemala, Guatemala City, Guatemala, [3]Emory University, Atlanta, GA, USA.

**Background:** Influenza A virus reservoirs in animals have historically provided novel genetic elements leading to pandemics. Detection and risk assessment of viruses in their animal hosts, before they spread into humans, are critical to protect public health. Bats, as a reservoir for potential emerging infections, are of particular interest. Here, we describe a novel influenza A virus from bats. **Methods:** A total of 316 bats from 21 different species were captured from eight locations in southern Guatemala in 2009 and 2010. Fecal swabs were screened for influenza RNA using a pan-influenza RT-PCR targeting the PB1 gene. Influenza RNA-positive samples were subjected to full genome sequencing using both Next Generation and Sanger sequencing approaches, followed by phylogenetic, genomic and proteomic characterization. In addition, to assess the potential for genetic exchange between virus lineages, we examined the compatibility between cloned bat virus and human influenza virus genes using a mini-genome reporter assay. **Results:** A novel influenza A virus was identified from three little yellow-shouldered bats in Guatemala, two at El Jobo during 2009 and a third at Agüero during 2010. The virus had key structural and molecular features that were highly homologous with other influenza viruses. The hemagglutinin (HA) of the bat virus was distinct from known influenza A subtypes and merits designation as a new subtype, H17. The neuraminidase (NA) gene was highly divergent from those of all known influenza viruses NAs. The "internal" genes from this virus diverged from those of known influenza A viruses prior to the divergence of influenza A internal genes. In spite of this divergence from known influenza viruses, the replicase of the novel bat virus was compatible with human influenza virus elements in human cells suggesting that reassortment might be possible. **Conclusions:** This study documents the first new influenza subtype to be described since 2005. The identification of influenza A viruses in bats expands the repertoire of reservoirs and raises questions as to the public health and agricultural ramifications attributable to this reservoir. Active surveillance of bats and other wildlife for

influenza virus reservoirs will help to reveal the evolution of influenza virus and its potential impact on human health.

**Hospital Outbreak of Carbapenem-Resistant *Klebsiella pneumoniae*—Panama, 2011**

**R. Dantes**[1], N. Gupta[1], V. Stempliuk[2], M. Guardo[3], J. Lombardo[4], H. Castillo[4], E. Landires[4], C. Urena[4], R. Mitre[4], M. de Paredes[4], R. Molino[4], R. Lopez[4], D. Santana[4], M. Mallorca[4], N. Sosa[5], R. Bolanos[5], C. Gould[1], J. Perz[1], A. Guh[1];
[1]Centers for Disease Control and Prevention, Atlanta, GA, USA, [2]Pan American Health Organization/World Health Organization, Washington DC, DC, USA, [3]Pan American Health Organization/World Health Organization, Panama City, Panama, [4]Complejo Hospitalario Metropolitano Arnulfo Arias Madrid, Panama City, Panama, [5]Instituto Conmemorativo Gorgas de Estudios de la Salud, Panama City, Panama.

**Background:** Carbapenem-resistant *Klebsiella pneumoniae* (CRKP) is a globally emerging, multidrug-resistant bacterial pathogen that causes healthcare-associated infections with few therapeutic options. CRKP containment is difficult due to easy transmissibility of resistance genes among bacteria; the most common resistance mechanism is *K. pneumoniae* carbapenemase (KPC). Hospital A became aware of CRKP infections among patients in August 2010, with steep increase in May 2011. We investigated the first known CRKP outbreak in Panama. **Methods:** We reviewed hospital laboratory and clinical records. Cases were defined as CRKP isolates from Hospital A patients during January 2009–July 2011. Presence of the KPC gene was confirmed by polymerase chain reaction. We conducted a case-control study to assess predictors for KPC-confirmed CRKP; controls were patients with non-CRKP cultures collected the same date as cases. Infection prevention practices were also assessed. **Results:** We identified 108 cases; 17 (16%) occurred before August 2010, and 53 (49%) since May 2011. Of 83 cases tested, 69 (83%) were KPC-confirmed. Record review was completed for 43 KPC-confirmed case-patients identified during May-July, 2011. These case-patients were located throughout the hospital and had a median age of 64 years (range: <1–94 years); 38 (88%) had ≥1 indwelling device, 24 (56%) had prior intensive or step-down care, and 26 (60%) had died. Multivariable analyses did not identify significant predictors for KPC-confirmed CRKP. Hand hygiene and use of Contact Precautions were suboptimal facility-wide. **Conclusions:** CRKP emerged in Panama earlier than previously recognized, culminating in a large hospital outbreak. High levels of device utilization in the context of inadequate infection control may have facilitated transmission. Adherence to infection control practices and improved surveillance for CRKP are needed to prevent continued transmission.

**A Newly Emerged Cutaneous Leishmaniasis Focus in Northern Israel and a Promising Means of Sand Fly Control**

**R. Faiman**[1], I. Abbasi[1], C. Jaffe[1], Y. Motro[2], A. Nasseredin[1], L. Schnur[1], M. Torem[3], A. Warburg[1];
[1]The Hebrew University of Jerusalem, Jerusalem, Israel, [2]Ministry of Agriculture and Rural Development, Beit Dagan, Israel, [3]Clalit Medicare, Kibbutz Sde Eliyahu, Israel.

**Background:** In 2006-7 18 cases of cutaneous leishmaniasis (CL) from Sde Eliyahu (pop. 650), a village in the Beit She'an valley of Israel, were reported to the Israel Ministry of Health. Between 2007-2011 cases reported from the village totaled 103 (15.8% of the total population). The majority of the cases (>90%) reside along the perimeter fence of the village. **Methods and Results:** Direct parasite isolates, PCR, and RFLP from skin lesions indicated the causative agent was *Leishmania major*. This parasite is transmitted by the sand fly *Phlebotomus papatasi* which constituted up to 97% of the sand flies trapped by the peripheral houses. Marking sand flies in-situ indicated directionality in their foraging behavior, entering the village from the surrounding fields. PCR screening for *Leishmania* ribosomal DNA (ITS1) of over 1,200 female sand flies trapped within the village resulted with an 18% mean infection rate, increasing toward the end of summer. Gut removal and microscopic examination of a sample of sand flies from Sde Eliyahu

enabled us to isolate, type, and culture *L. major* promastigotes. Surveying the rodent fauna in the village surroundings revealed the absence of the documented reservoir of *L. major* - *Psammomys obesus* within a 3-4 kilometer radius. The Social (=Gunther's) vole *Microtus guentheri* was abundant throughout the region, heavily infesting the agricultural fields closely surrounding the village. Ear-tip tissue from 27 voles (16.5%) tested positive for *Leishmania* DNA. Reverse Line Blotting resulted with *L. major* as the causative parasite. Smaller numbers of *Meriones tristrami*, found further away from the village tested positive for ITS1 by PCR (14 of 27). In the lab, subdermally inoculated voles developed heavy infections. Sand flies became infected after feeding on the ears of infected voles. **Conclusions:** To our knowledge, this is the first report implicating *Mic. guentheri* and *M. tristrami* as potential reservoirs of *L. major*. With the widespread co-distribution of both vole and *P. papatasi* sand flies in Israel and the region, the potential for emerging foci of CL may increase, necessitating novel and effective control measures such as described herein and elsewhere.

**Enhanced Tuberculosis Case-finding Using Home-based Symptom Screening and Sputum Collection in Rural Western Kenya**

**D. Noyle**[1,2], G.M. Bigogo[1,2], J. Agaya[2,3], G. Aol[1,2], K. Mutai[1,2], K. Laserson[2], J.M. Montgomery[1,2], R.F. Breiman[1,2], K.P. Cain[2,3], D.C. Burton[1,2];
[1]International Emerging Infections Program-Kenya, Division of Global Disease Detection and Emergency Response, Centers for Disease Control and Prevention (CDC)-Kenya, Kisumu and Nairobi, Kenya, [2]Kenya Medical Research Institute (KEMRI)/CDC Research and Public Health Collaboration, Kisumu and Nairobi, Kenya, [3]Tuberculosis Branch, KEMRI/CDC Research and Public Health Collaboration, Kisumu, Kenya.
**Background:** Evidence suggests that accelerating reductions in tuberculosis (TB) burden and deaths requires further enhancements in TB case detection. Community-based TB case-finding strategies may increase case detection over the current facility-based passive system in rural western Kenya, where healthcare utilization is low. **Methods:** Trained community interviewers attempted to screen 24,990 participants in population-based surveillance in rural western Kenya using a standardized questionnaire to identify participants with possible TB symptoms (self-reported cough, or fever, or drenching night sweats during the past 2 weeks, or weight loss during the past 4 weeks) in October-December 2011. Symptomatic participants ≥ 15 years of age were consented for sputum collection at home. Sputum specimens underwent PCR-based TB testing using the Cepheid Xpert® MTB/RIF assay. Smear microscopy was performed on specimens with a positive PCR result. Data abstracted from TB treatment registers in the surveillance area and self-reported history of TB diagnosis in the past 2 years were used to identify cases already detected through routine facility-based case-finding. Data are reported for participants ≥ 15 years of age with a PCR-positive TB test. The national TB program was notified of all positive TB test results and participants with TB were encouraged to seek care at a nearby TB clinic. **Results:** Of 13,844 participants ≥ 15 years, 6,575 (47%) were available at home for screening; 1,804 (27%) reported symptoms compatible with TB infection and 1,541 (85%) of these provided an adequate sputum specimen for testing. Eighteen (1.2%) specimens were positive by PCR, of which 10 (56%) were smear-positive. None demonstrated rifampin resistance. Among 9 TB cases with known HIV status, 6 (67%) were HIV-infected. The most commonly reported symptom among TB cases was cough (89%), followed by night sweats (33%), weight loss (28%), and fever (17%). Thirteen (72%) of the TB cases were new diagnoses, representing 2 previously undiagnosed symptomatic TB cases per 1,000 persons ≥ 15 years of age. Eight (44%) of the TB cases visited a TB clinic for care within 45 days after receiving their test results. **Conclusions:** Community-based TB case-finding can be an effective adjunct to facility-based passive TB case-finding strategies in rural Kenya.
